# Automatic Assignment of Prokaryotic Genes to Functional Categories Using Literature Profiling

**DOI:** 10.1371/journal.pone.0047436

**Published:** 2012-10-15

**Authors:** Raul Torrieri, Francislon S. Oliveira, Guilherme Oliveira, Roney S. Coimbra

**Affiliations:** 1 Center for Excellence in Bioinformatics, FIOCRUZ-Minas, Belo Horizonte, Brasil; 2 Genomics and Computational Biology Group, FIOCRUZ-Minas, Belo Horizonte, Brasil; Kyushu Institute of Technology, Japan

## Abstract

In the last years, there was an exponential increase in the number of publicly available genomes. Once finished, most genome projects lack financial support to review annotations. A few of these gene annotations are based on a combination of bioinformatics evidence, however, in most cases, annotations are based solely on sequence similarity to a previously known gene, which was most probably annotated in the same way. As a result, a large number of predicted genes remain unassigned to any functional category despite the fact that there is enough evidence in the literature to predict their function. We developed a classifier trained with term-frequency vectors automatically disclosed from text *corpora* of an ensemble of genes representative of each functional category of the J. Craig Venter Institute Comprehensive Microbial Resource (JCVI-CMR) ontology. The classifier achieved up to 84% precision with 68% recall (for confidence≥0.4), F-measure 0.76 (recall and precision equally weighted) in an independent set of 2,220 genes, from 13 bacterial species, previously classified by JCVI-CMR into unambiguous categories of its ontology. Finally, the classifier assigned (confidence≥0.7) to functional categories a total of 5,235 out of the ∼24 thousand genes previously in categories “Unknown function” or “Unclassified” for which there is literature in MEDLINE. Two biologists reviewed the literature of 100 of these genes, randomly picket, and assigned them to the same functional categories predicted by the automatic classifier. Our results confirmed the hypothesis that it is possible to confidently assign genes of a real world repository to functional categories, based exclusively on the automatic profiling of its associated literature. The LitProf - Gene Classifier web server is accessible at: www.cebio.org/litprofGC.

## Introduction

In the last years, there was an exponential increase in the number of publicly available genomes. Once finished, most genome projects lack financial support to review annotations. Extracting knowledge from genome sequencing efforts requires the predicted genes to be functionally annotated. A few of these gene or genome annotations are based on a combination of computationally derived evidence, such as metabolic reconstruction, presence of candidate transcription factor binding sites or even the fact that functionally related genes tend to cluster on prokaryotic chromosomes [1]. However, in most cases, annotations are based solely on sequence similarity to a previously known gene, which was most probably annotated in the same way [2]. In addition, it is often difficult to find the genes that were experimentally validated to evaluate the reliability of these original annotations [3]. In cases where the reference sequence has no annotation or is annotated as “Unknown function”, or even is incorrectly annotated, then all sequences subsequently annotated based on their similarity to the references will inherit their inexactly assigned attributes. Current bioinformatics-based approaches cannot predict the function of up to one-third of sequenced genes. For prokaryote genomes deposited in the J. Craig Venter Institute Comprehensive Microbial Resource (JCVI-CMR) (http://cmr.jcvi.org/cgi-bin/CMR/shared/RoleList.cgi), genes classified as “Unknown function” account for ∼10% (26,390) of the total of deposited genes. Those assigned to the category “Unclassified” account for ∼18% (45,870). The “Unknown Function” and “Unclassified” genes represent ∼30% of all unique prokaryotic genes at JCVI-CMR. Furthermore, for some gene families, at least 60% of the gene predictions are wrong [4]. For many of these genes there is sufficient evidence in the literature to identify their function [5]. However, the costs involved in manually reviewing the literature to improve gene annotation in a whole genome sequencing project are prohibitive. Text-mining algorithms can help in this task.

We present, herein, LitProf – Gene classifier, a tool for automatically assigning prokaryote genes to functional categories of the JCVI-CMR ontology (File S1) based exclusively on the literature profiles extracted from their gene-specific collections of abstracts in MEDLINE database (http://www.pubmed.org). Using LitProf – Gene classifier we were able to propose functional categories to 5,235 out of the 69,088 genes previously assigned to categories “Unknown function” or “Unclassified” of the JCVI-CMR ontology.

## Results

### Disclosing the informative vocabulary required to describe each functional category

Starting from 2,201,517 loci from JCVI-CMR repository we filtered out genes that do not have a name (80%), and genes assigned to more than one functional category of JCVI-CMR ontology (12%). Were also genes assigned to the categories “unknown function”, “unclassified” and “disrupted reading frame”, which combined accounts for approximately 16% of all loci. For each group of homonymous genes we kept only one randomly chosen representative. The final dataset consisted of 59,830 unique canonical gene names from 117 genomes representing all prokaryote phylogenetic branches in JCVI-CMR (Table 1). After the redundancy reduction steps, the resultant gene set comprised 3,542 genes preserving the proportional contribution of each functional categories in JCVI-CMR (Table 2). LitProf retrieved 126,990 MEDLINE abstracts for the selected 3,542 genes (average = 35.8 abstracts per gene). From this text *corpus* LitProf disclosed the minimum informative vocabulary of 889 stemmed terms, and represented each gene as a term-frequency vector.

**Table 1:** Taxonomy distribution of genes in the training dataset.

Phylum	# ofspecies
Acidobacteria	1
Actinobacteria	5
Aquificae	1
Bacteroidetes	3
Chlamydiae	1
Chlorobi	1
Chloroflexi	4
Crenarchaeota	3
Cyanobacteria	9
Deinococcus-Thermus	2
Euryarchaeota	10
Fibrobacteres	1
Firmicutes	15
Fusobacteria	1
Nanoarchaeota	1
Planctomycetes	1
Proteobacteria	49
Spirochaetes	2
Tenericutes	3
Thermotogae	1
Virus	3

**Table 2:** Gene distribution in the functional categories of the JCVI-CMR ontology.

Functional category	Dataset		
	Original (%)	Training (%)	Classified (%)
Amino acid biosynthesis	4102 (2.53)	90 (2.54)	115 (2.20)
Biosynthesis of cofactors, prosthetic groups, and carriers	5482 (3,39)	148 (4.18)	179 (3.42)
Cell envelope	227 (1.40)	47 (1.33)	41 (0.78)
Cellular processes	17778 (10.99)	353 (9.97)	283 (5.41)
Central intermediar metabolism	2517 (1.56)	78 (2.20)	92 (1.76)
DNA metabolism	9238 (5.71)	287 (8.10)	344 (6.57)
Energy metabolism	27132 (16.77)	744 (21.01)	1363 (26.04)
Fatty acid and phospholipid metabolism	4823 (2.98)	118 (3.33)	253 (4.83)
Mobile and extrachromosomal element functions	7716 (4.77)	123 (3.47)	119 (2.27)
Protein fate	11611 (7.17)	359 (10.14)	550 (10.51)
Protein synthesis	9044 (5.59)	140 (3.95)	149 (2.85)
Purines, pyrimidines, nucleosides, and nucleotides	2678 (1.65)	62 (1.75)	77 (1.47)
Regulatory functions	18817 (11.63)	247 (6.97)	327 (6.25)
Transcription	2816 (1.74)	86 (2.43)	50 (0.96)
Transport and binding proteins	25368 (15.68)	363 (10.25)	757 (14.46)
Mix category	8297 (5.13)	297 (8.39)	536 (10.24)

Only categories used to train the classifier are shown. Mix category regroups the noisy subcategories. The original column refers to the complete J. Craig Venter Institute Comprehensive Microbial Resource (JCVI-CMR). The training column refers to the dataset used to train the classifier. The classified column refers to the “Unknown function” and “Unclassified” genes that were classified by LitProf- Gene Classifier with confidence≥0.7. There is no significant difference between the original and training datasets (p>0.05 in paired t-test; confidence level of 95%).

### SVM training

Genes represented by their term-frequency vectors together with the information about their respective functional categories, previously assigned to each gene by JCVI-CMR team, were used to train the SVM classifier. The “Signal transduction” category was underrepresented in our gene set, most probably because these genes are often assigned to more than one category in the JCVI-CMR. As a consequence, there were not enough genes assigned by JCVI-CMR only to the category “Signal transduction” making it impossible to train the SVM to recognize this category. For that reason this category was removed from the training dataset. During the cross-validation of the classifier we observed that the subcategories “Other” from categories “Cell envelope” and “Central intermediary metabolism” were frequently misclassified. For that reason, these subcategories were excluded. The subcategories “Biosynthesis and degradation of surface polysaccharides and lipopolysaccharides” and “Biosynthesis of murein sacculus and peptidoglycan” from the category “Cell envelope”, “Biosynthesis and degradation of polysaccharides” from the category “Energy metabolism” and “Sugar-nucleotide biosynthesis and conversions” from category “Purines, pyrimidines, nucleosides, and nucleotides”, all related to polysaccharide biosynthesis added noise to the classifier and thus were merged into a new “Mix category”. In any case subcategories were exchanged between established JCVI-CMR categories. Thus, the category-subcategory hierarchy of the original JCVI-CMR ontology was not violated. After these adjustments, the rearranged ontology had 16 categories comprising 115 sub-categories.

**Figure 1:**
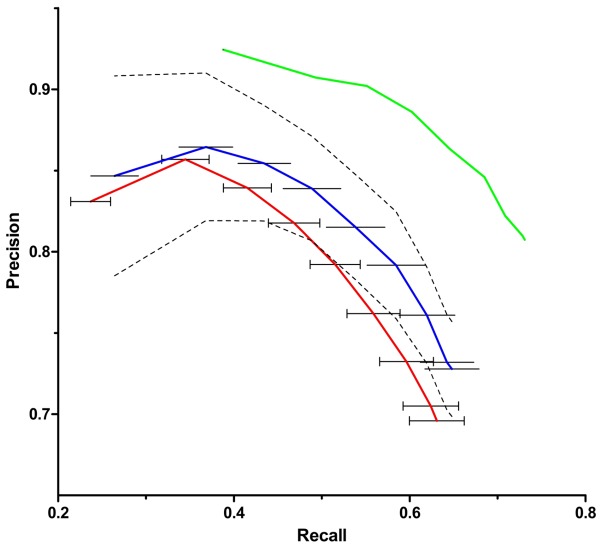
Recall vs. precision of the classifier. The red line represents the average performance of the initial classifier trained with the original categories of the JCVI-CMR ontology. The blue line, represents the average performance of the final classifier trained with a rearranged version of the ontology where noisy subcategories were merged together to create the Mix Category. For red and blue lines, the average was calculated from 100 replicates of 10-fold cross validation. The green line represents the performance of the final classifier in an independent gene set. Horizontal bars represent the standard deviations of recall. The dashed lines represent the standard deviation of precision for the blue curve.

After the ontology rearrangement, the SVM classifier was retrained and cross-validation was performed again. The estimated average precision of the resultant classifier was 80±3%, and the average recall was 60±3% (confidence≥0.4); for equally weighted recall and precision, the F-measure was 0.7. Furthermore, for the independent gene set the classifier achieved 84% precision with 68% recall (F-measure = 0.76) for confidence≥0.4, and 90% precision with 55% recall (F-measure = 0.68) for confidence≥0.7, as shown in Figure 1.

It is important to highlight that the SVM classifier predicts categories, not subcategories. At present, there are not enough genes in JCVI-CMR assigned to only one functional subcategory so that we could have used them to compose a robust example gene set required to train the SVM with enough statistical support.

### Classification of “Unknown function” and “Unclassified” genes

We used the classifier to tentatively classify the 69,088 genes previously assigned by JCVI-CMR to the categories “Unknown function” or “Unclassified”. However, only 34,033 genes had a name, and 23,973 of these genes had at least five abstracts in MEDLINE. Their text *corpora* retrieved by LitProf – Gene Classifier with the thresholds established in this experiment (min = 5 abstracts; max = 50) summed up 247,442 abstracts (average = 10.3 abstracts/gene). For a minimum confidence threshold of 0.7, LitProf – Gene Classifier unambiguously assigned 5,235 out of the ∼24 K genes with literature in MEDLINE to a functional category. Table 3 summarizes these results. For details on the classified genes see Table S3.

**Table 3:** Summary of the classification of genes previously assigned to categories “Unknown function” and “Unclassified” of the JCVI-CMR ontology.

Filters	# of genes
Total number of “Unknown function” and “Unclassified” genes	69,088
Genes that have a name	34,033
Genes with at least five abstracts in MEDLINE	23,973
Classified genes (confidence threshold≥0.7)	5,235

From the total number of “Unknown function” and “Unclassified” genes, nearly 50% have a name, with is crucial for text *corpora* retrieval. From those, ∼70% have enough literature (min = five abstracts; max = 50) for classification, and in this group, ∼22% could be assigned by LitProf - Gene Classifier to a functional category with high confidence.

JCVI-CMR = J. Craig Venter Institute Comprehensive Microbial Resource.

The classified genes span over all functional categories present in the training dataset. One-hundred randomly picked genes of this set were manually classified by two Biologists who reviewed their literature. Their manual classifications completely agreed with those of LitProf – Gene Classifier (see Table 4 for a sample of these results). No significant difference was found in the gene distribution in functional categories when comparing the classified gene set, the training gene set, and the original JCVI-CMR (Table 3).

**Table 4:** Examples of genes classified by LitProf- Gene Classifier and further validated by manually reviewing their literature.

Name	JCVI-CMR Accession	Species	Predicted category	Confidence	PubmedIDs	GO Biological process (species with GO annotated ortholog) *
ArsR protein	NT01MC4786	*Magnetococcus* sp. MC-1	Regulatory functions	0.98	20724137; 20586430	GO:0006355: regulation of transcription, DNA-dependent (*Pseudomonas aeruginosa* PAO1)
Phosphatidylserine decarboxylase, putative	GSU_1908	*Geobacter sulfurreducens* PCA	Fatty acid and phospholipids metabolism	0.96	14651609; 16667073	GO:0006660: phosphatidylserine catabolic process; GO:0004609: phosphatidylserine decarboxylase (*Geobactersulfurreducens PCA*)
UmuD protein [Contains: UmuD protein]	NT03PS1033	*Candidatus Protochlamydia amoebophila* UWE25	DNA metabolism	0.97	14651609; 16667073	GO:0009432: SOS response; GO:0009650: UV protection; GO:0008236: serine-type peptidase activity (*Colwellia psychrerythraea* 34H)
phage portal protein	NT03SP0558	*Streptococcus pyogenes* MGAS8232	Mobile and extrachromosomal element functions	0.95	20467052; 19947526	GO:0019068: virion assembly; GO:0019012: virion; GO:0005198: structural molecule activity (*Clostridium perfringens* ATCC13124)
Putative metalloprotease	pc0037	*Candidatus Protochlamydia amoebophila* UWE25	Protein fate	0.94	20838651; 20812964	GO:0006508: proteolysis; GO:0008233: peptidase activity (*Methylococcus capsulatus* str. Bath)
Lambda Kil	ECH74115_3562	*Escherichia coli* O157:H7 str. EC4115	Mobile and extrachromosomal element functions	0.98	12441108; 11470529	-
bacteriophage tail fiber assembly protein	NT06EC2684	*Erwinia carotovora* atroseptica SCRI1043	Mobile and extrachromosomal element functions	0.95	20531477; 10051617	-
staphylococcal respiratory response protein, SrrB	SAUSA300_1441	*Staphylococcus aureus* subsp. aureus USA300-FPR3757	Regulatory functions	0.92	17697253; 17198402	-
Clp amino terminal domain protein	NT01NFA0344	*Nocardia farcinica* IFM10152	Protein fate	0.99	20014030; 19843523	-
Putative malate dehydrogenase	nfa36620	*Nocardiafarcinica*IFM10152	Energy metabolism	0.94	20127467; 19405028	GO:0006108: malate metabolic process; GO:0016615: malate dehydrogenase activity (*Bacillus anthracis*)
(R)-2-hydroxyglutaryl-CoA dehydratase activator	NT01CA2639	*Clostridium acetobutylicum*ATCC824	Regulatory functions	0.76	11106419; 15374661	GO:0006520: cellular amino acid metabolic process; GO:0008047: enzyme activator activity (*Geobacter sulfurreducens* PCA)
putative beta-lactamase II	NT05LB0990	*Leptospira biflexa* serovar Patoc strain Patoc1	Cellular processes	0.58	19407375; 16452624	GO:0017001: antibiotic catabolic process; GO:0008800: beta-lactamase activity (*Bacillus anthracis*)
Carbohydrate binding protein, cbp35C	CJA_0494	*Cellvibriojaponicus* Ueda107	Transport and binding proteins	0.80	20816499; 20713592	-
Serine acetyltransferase, putative	GFRORF1528	*Prevotella ruminicola* 23	Cellular processes	0.58	20830571; 20189106	GO:0006535: cysteine biosynthetic process from serine; GO:0009001:serine O-acetyltransferase (*Campylobacter jejuni* RM1221)
CoA ligase Family protein	NT01BT3039	*Bacteroides thetaiotaomicron*VPI-5482	Fatty acid and phospholipid metabolism	0.81	20545743; 20534558	GO:0008150: biological process; GO:00165878: acid-thiol ligase activity; GO:0016208: AMP binding (*Colwellia psychrerythraea* 34H)
Modification methylase SalI	NT09RC1177	*Roseiflexus castenholzii* DSM 13941	DNA metabolism	0.81	9628360; 9130589	-

* The GO terms from Biological Process, Molecular Function and Cellular Component ontologies associated with each gene in table 4 (or, in most cases, their prokaryotic orthologs) were retrieved from AmiGO (http://amigo.geneontology.org) by querying the database with their canonical gene names. In most cases the GO terms retrieved supported the functional categorization predicted by LitProf – Gene Classifier, although there is not an exact correspondence between GO and JCVI-CMR ontologies. Six gene names out 16 tested had no match in AmiGO.

## Discussion

The current scenario of gene functional annotation (annotations based solely on sequence similarity to a previously known gene, which was most probably annotated in the same way [2]) condemns “Unknown function” and “Unclassified” genes to remain in this situation. Under this perspective this work brings significant contribution by being able to automatically capture reliable gene function categories to unknown genes. In practical terms it means that precision is a more important characteristic for a gene functional classifier than recall. Another important aspect that has to be considered is the classifier's ability to deal with real world datasets. In other words, the classifier has to present a good performance when any misclassified gene is submitted for functional assignment.

Other gene classifiers with good performance have already been proposed, but attention should be paid to the possible bias introduced in their models by the training and test datasets used. Artificial highly informative training datasets composed only of selected true positive examples (i.e., abstracts previously known to describe the gene function) will improve the performance of the classifier since the training process will not be disturbed by noise in the data. Classifiers created under such conditions tend to underperform in real-world situations when the text *corpora* associated to the test dataset contain informative and non-informative documents in unpredictable proportions.

In a previous work, Theodosiou and co-workers [6] developed a SVM classifier for assigning one of 12 selected GO categories [7] to a gene product by searching abstracts retrieved from MEDLINE for MeSH terms, previously associated to GO categories by the authors. That classifier achieved an average recall of 0.70 and precision of 0.68. For equally weighted recall and precision the F-measure was 0.69. However, both training and test datasets used in that study were composed of highly informative selected text *corpora*, retrieved based on MeSH terms used as proxy for GO categories. MeSH terms contribute to compose a highly informative training dataset that can bias the classifier.

Another work, that competed in BioCreAtIvE II [8], also presented a good performance achieving recall of 0.82 and precision of 0.67. With equally weighted recall and precision the F-measure was 0.74. The BioCreAtIvE II training dataset was mostly (64.3%) composed of true positive documents and the remaining 35.7% are known true negatives [8]. The classifier was trained with a percentage of noise documents that is unlikely to be observed in the text *corpora* of the “Unknown function” and “Unclassified” genes of any gene repository.

In the present work, the abstracts were retrieved solely by querying PubMed with the gene names. No strategy was used to enrich the text *corpus* with highly informative documents. The two datasets we used (one for training and cross-validation and one independent test dataset) were composed of text *corpora* from previously classified genes randomly picked from JCVI-CMR, covering 117 species. The informative vocabulary was then automatically disclosed by analyzing the frequency of terms in the text *corpus* of all genes. Importantly, the model was trained, tested and evaluated with datasets with a distribution of functional categories similar to that of the source (real life) repository of genes. Because we dramatically reduced the redundancy in the training dataset, when applied to an independent test dataset (2,220 previously classified genes randomly picked from JCVI-CMR), that represents the real world genes ensemble which contains a certain level of redundancy (orthologs, etc), the classifier performed even better than in the cross-validation step (Figure 1).

LitProf-Gene Classifier is species blind since we assumed that orthologs sharing the same name may also share similar functions. This assumption may be true in the majority of the cases and is also behind the popular strategy of gene annotation transfer based on sequence similarity. Our rationale in this work was that once a given gene “X” is named based on its sequence similarity to another gene “Y” which, at that time, was not yet assigned to any functional category, it will inherit this “misclassification”. Later on, new experimental studies can clarify the function of the ortholog “Y”, but its reviewed functional annotation will not be automatically transferred to gene “X” in the main public databases (we took JCVI-CMR as a case study). It is important to remark that the aim of the present work was to review gene functional annotation, not gene name assignment. Nevertheless, Litprof-Gene Classifier's performance depends on how informative is the text *corpus* of the gene to be classified. Incorrectly selecting documents due to gene name ambiguity has low impact in the training step of the SVM since the training gene set contains many examples of each functional category, the majority of them with unambiguous text *corpora*. However, individual genes with ambiguous text corpora will be classified by LitProf – Gene Classifier with low confidence. For example, querying PubMed with gene symbols *mgt*A and *mgt*B resulted in 69 and 39 abstracts respectively. Both genes are involved in magnesium transport in prokaryotes, but some abstracts indexed for gene symbol *mgt*B were clearly not related to this function (e.g. PMID: 21604018 – “male genital tuberculosis”). As a consequence, Litprof- Gene Classifier unambiguously assigned *mgt*A to functional category “Regulatory Functions” (confidence = 0.82), whereas *mgt*B could only be categorized with very low confidence (0.39), which is below the cutoff of 0.7 assumed to be sufficient for reliable classifications.

Differently from some of previous works, the main contribution of our initiative is that using the developed SVM model we could assign more than five thousand genes to functional categories, defined in a complete and well structured ontology. This represents more than 20% of the genes previously assigned to “Unknown function” or “Unclassified” categories despite having literature in MEDLINE. This number accounts for approximately two bacterial genomes completely annotated. These genes would probably never have their misannotation reviewed otherwise. From the total number of genes initially selected, only 49.3% have a name which is pre-requisite for text *corpora* retrieval. For genes with a name, 70% had enough abstracts in MEDLINE to be processed by LitProf. Table 5 shows the total number of genes classified by LitProf – Gene Classifier when no restrictions were set to confidence values. These numbers make evident the considerable amount of genes currently lacking functional annotation. Another relevant issue is the multiple category annotation of some genes. At present, only 12% of the genes in the JCVI-CMR are classified in more than one functional category. However, it is not surprising that some categories, such as the “Signal transduction”, are enriched in genes classified in more than one category. To better accommodate these cases, future work to improve LitProf – Gene Classifier will focus on predicting multiple classes instead of only the most probable class.

**Table 5:** Number of genes assigned to functional categories with different confidence thresholds.

confidence	genes
≥0.9	1804
≥0.8	3479
≥0.7	5235
≥0.6	7085
≥0.5	9055
≥0.4	11797
≥0.3	15584
≥0.2	23397
≥0.1	23973

As exemplified in table 4, the information granularity of the main categories of JCVI-CMR is similar to that of the upper levels of the GO “Biological Process” ontology, which is the only annotation available for many prokaryotic genes. As far as more genes classified in only one subcategory will be available in the JCVI-CMR database, it is conceivable that LitProf – Gene Classifier could be trained with these examples to also predict subcategories of the JCVI-CMR ontology. In principle, the same strategy could be used to train the classifier to predict functional categories of other complimentary ontologies, such as GO.

According to Blaby-Haas[2], the experimental characterization of the millions of genes sequenced is, to date, an impossible task. For that reason, automated methodologies for gene function annotation are essential in the post genomic era. Our results confirmed the hypothesis that it is possible to confidently assign genes of a real world repository to functional categories, based exclusively on the automatic profiling of its associated literature. The LitProf- Gene Classifier web server may be a valuable complimentary tool for the community involved in prokaryote gene annotation. A web server version of LitProf – Gene classifier is accessible at: www.cebio.org/litprofGC.

## Methods

### Disclosing the informative vocabulary required to describe each functional category

To compose the initial data set required to train the Support Vector Machine (SVM), canonical prokaryote gene names distributed in the 20 functional categories of the JCVI-CMR ontology (downloaded in 8^th^ June 2010), were randomly selected from the genomes of 117 prokaryote taxa represented in the JCVI-CMR database. Each of these genes had been assigned by JCVI-CMR to only one functional category. We used as gene name, the content of the field “Putative identification” from JCVI-CMR. The initial gene set was then screened to eliminate homonymous genes and genes assigned to more than one functional category that could bias the gene set. Genes assigned to categories “Disrupted reading frame”, “Hypothetical proteins”, “Unclassified” or “Unknown function” of the JCVI-CMR ontology were also excluded. For details on the rearranged ontology, see File S2.

We used the software LitProf, a homemade customized implementation of the Chaussabel & Sher algorithm [9], to identify the minimum vocabulary required to describe the function of a given gene from a collection of its gene-specific abstracts in MEDLINE. LitProf was first used by Coimbra *et al* to cluster genes differentially expressed in infant rat brain in the course of experimental meningitis [10] and later, to estimate the ambiguity level of individual aliases in large gene terminologies based exclusively in their name-specific vocabulary fingerprints automatically extracted from abstracts in MEDLINE [11].

LitProf works in three fundamental steps. In the first step, it retrieves the abstracts from MEDLINE by sequentially querying the online version of PubMed with gene names in a list provided by the user. LitProf communicates with PubMed using the Entrez Programming Utilities (E-utilities), the structured interface to the Entrez system [http://eutils.ncbi.nlm.nih.gov/]. Searches are case insensitive and approximate matches are allowed. In this study we only included genes which had at least five abstracts in MEDLINE. For these genes, up to the 50 most recent abstracts were retrieved by LitProf. In the second step, for each gene a vector of stemmed terms, suffix stripped with the Porter stemming algorithm, and their relative frequencies is constructed. The term frequency is defined as the fraction of abstracts containing the term in the text *corpus* of a given gene. In the last step LitProf reduces the dimensionality of the vectors removing all the promiscuous or gene-specific terms. To this purpose, LitProf first determines the baseline frequencies of each term occurring in a set of 7,465 MEDLINE abstracts retrieved for a set of 230 human official gene symbols randomly picked. Terms with frequencies higher than a user-defined cut-off in the baseline (five percent in this study) are eliminated from the vectors of the experimental set of genes. Then, terms for which the difference between their frequencies in the text *corpora* of the experimental gene set and in baseline exceeds an optimized cut-off are excluded. The optimized cut-off is defined applying the equation: opt_cut-off = t+(k/n) where t is the minimum threshold (in this study t = 0.15), k is a constant (in this study k = 1.5) and n is the number of abstracts retrieved for a given gene. The optimized cut-off compensates for the differences in the number of abstracts retrieved for each gene.

To further decrease redundancy in the gene set, genes were grouped by Hierarchical Clustering algorithm implemented in GenePattern[12] (Pearson correlation as distance metric and average linkage as clustering method) using as input the term-frequency vectors produced by LitProf. For each cluster of highly correlated genes (≥0.99 correlation), one representative gene was randomly chosen. The resultant gene list was resubmitted to LitProf and the minimum informative vocabulary, now adjusted to avoid the bias of genes too closely related, was recreated. For details on the training dataset, see Table S1.

### SVM training and evaluation

The term-frequency vectors together with the respective functional categories previously assigned to each gene by JCVI-CMR were used to train a gene classifier using the SVM implemented in GenePattern, designed to use a Radial Basis Function kernel function. We first assessed the classifier's performance using a 10-fold cross validation with 100 replicates. A homemade Perl script shuffled the original training dataset and divided it in training and test sets containing 90% and 10% of the genes, respectively. The SVM classifier from GenePattern was then automatically launched for each of the 100 replicas. After all the iterations, the standard deviations are calculated for recall and precision at each confidence level. We also assessed the recall and precision of classifier trained with the full training set against an independent set of 2,220 randomly picked genes, from 13 species not used in the training step, previously classified into unambiguous categories of the JCVI-CMR ontology. For details on the independent test dataset see Table S2.

### LitProf - Gene Classifier

We developed LitProf - Gene Classifier, a web-based tool composed of a set of Perl scripts that integrate the validated SVM model and all the required steps for assign a gene to a functional category based solely on its available literature.

Given a list of genes names provided by the user, the tool retrieves a minimum of five and a maximum of 50 abstracts for each gene from MEDLINE repository. These abstracts compose the text *corpora* for each gene. From these text *corpora* LitProf – Gene Classifier calculates the frequency for each term of the minimum informative vocabulary used in the previous step to training the SVM. These term-frequency vectors for each gene to be classified are automatically loaded to the SVM (implemented in GenePattern) together with the predictive model produced in the training step. The output is a web page showing the gene name, the predicted category, and the confidence.

### Statistical analysis

Gene distribution over functional categories in the classified gene set, the training gene set, and the original JCVI genome resource, were compared using a paired t-test, with a confidence level of 95%, using GraphPad Prism version 5.02 (GraphPad Software, Inc. CA, USA).

## Supporting Information

File S1 **Original JCVI-CMR ontology.**
(DOC)

File S2 **Rearranged JCVI-CMR ontology.**
(DOC)

Table S1 **Training dataset – Used to train the SVM classifier and to perform the cross-validation.**
(DOC)

Table S2 **Independent dataset – Used to test the SVM classifier.**
(DOC)

Table S3 **Genes classified by LitProf - Gene Classifier which were previously assigned to ‘Unknown function’ and ‘Unclassified’ categories of JCVI-CMR.**
(DOC)
