# Supplementary material for: Automatic Assignment of Prokaryotic Genes to Functional Categories Using Literature Profiling
Source: PLoS One. 2012 Oct 15;7(10):e47436. doi: 10.1371/journal.pone.0047436 (PMC3471813; doi:10.1371/journal.pone.0047436)
Supplement: File S2 — Rearranged JCVI-CMR ontology. (DOC) [file pone.0047436.s002.doc]

**REARRAGED JCVI-CMR ONTOLOGY**

**1. Amino acid biosynthesis**

1.1 Aromatic amino acid family

1.2 Aspartate family

1.3 Glutamate family

1.4 Pyruvate family

1.5 Serine family

1.6 Histidine family

1.7 Other

**2. Biosynthesis of cofactors, prosthetic groups, and carriers**

2.1 Biotin

2.2 Folic acid

2.3 Heme, porphyrin, and cobalamin

2.4 Lipoate

2.5 Menaquinone and ubiquinone

2.6 Molybdopterin

2.7 Pantothenate and coenzyme A

2.8 Pyridoxine

2.9 Riboflavin, FMN, and FAD

2.10 Glutathione and analogs

2.11 Thiamine

2.12 Pyridine nucleotides

2.13 Chlorophyll and bacteriochlorphyll

2.14 Siderophores

2.15 Other

2.16 Chlorophyll

2.17 Glutathione

**3. Cell envelope**

3.1 Surface structures

3.2 Biosynthesis and degradation of murein sacculus and peptidoglycan

**4. Cellular processes**

4.1 Cell division

4.2 Chemotaxis and motility

4.3 Cell adhesion

4.4 Conjugation

4.5 Detoxification

4.6 DNA transformation

4.7 Sporulation and germination

4.8 Toxin production and resistance

4.9 Pathogenesis

4.10 Adaptations to atypical conditions

4.11 Biosynthesis of natural products

4.12 Other

**5. Central intermediary metabolism**

5.1 Amino sugars

5.2 One-carbon metabolism

5.3 Phosphorus compounds

5.4 Polyamine biosynthesis

5.5 Sulfur metabolism

5.6 Nitrogen fixation

5.7 Nitrogen metabolism

5.8 Electron carrier regeneration

**6. DNA metabolism**

6.1 DNA replication, recombination, and repair

6.2 Restriction/modification

6.3 Degradation of DNA

6.4 Chromosome-associated proteins

6.5 Other

**8. Energy metabolism**

8.1 Aerobic

8.2 Amino acids and amines

8.3 Anaerobic

8.4 ATP-proton motive force interconversion

8.5 Electron transport

8.6 Entner-Doudoroff

8.7 Fermentation

8.8 Glycolysis/gluconeogenesis

8.9 Pentose phosphate pathway

8.10 Pyruvate dehydrogenase

8.11 Sugars

8.12 TCA cycle

8.13 Methanogenesis

8.15 Photosynthesis

8.16 Chemoautotrophy

8.17 Other

**9. Fatty acid and phospholipid metabolism**

9.1 Biosynthesis

9.2 Degradation

9.3 Other

**11. Mobile and extrachromosomal element functions**

11.1 Plasmid functions

11.2 Prophage functions

11.3 Transposon functions

11.4 Other

**12. Protein fate**

12.1 Protein and peptide secretion and trafficking

12.2 Protein modification and repair

12.3 Protein folding and stabilization

12.4 Degradation of proteins, peptides, and glycopeptides

12.5 Other

**13. Protein synthesis**

13.1 tRNA aminoacylation

13.2 Ribosomal proteins: synthesis and modification

13.3 tRNA and rRNA base modification

13.4 Translation factors

13.5 Other

13.6 Nucleoproteins

**14. Purines, pyrimidines, nucleosides, and nucleotides**

14.1 2'-Deoxyribonucleotide metabolism

14.2 Nucleotide and nucleoside interconversions

14.3 Purine ribonucleotide biosynthesis

14.4 Pyrimidine ribonucleotide biosynthesis

14.5 Salvage of nucleosides and nucleotides

14.7 Other

**15. Regulatory functions**

15.1 DNA interactions

15.2 RNA interactions

15.3 Protein interactions

15.4 Small molecule interactions

15.5 Other

**17. Transcription**

17.1 Degradation of RNA

17.2 DNA-dependent RNA polymerase

17.3 Transcription factors

17.4 RNA processing

17.5 Other

**18. Transport and binding proteins**

18.1 Amino acids, peptides and amines

18.2 Anions

18.3 Carbohydrates, organic alcohols, and acids

18.4 Cations and iron carrying compounds

18.5 Nucleosides, purines and pyrimidines

18.6 Porins

18.7 Other

18.8 Unknown substrate

18.9 Cations

**19. Mix category**

19.1 Biosynthesis and degradation of surface polysaccharides and

lipopolysaccharides - from the category “Cell envelope”

19.2 Biosynthesis of murein sacculus and peptidoglycan - from the category “Cell envelope”

19.3 Biosynthesis and degradation of polysaccharides - from the category “Energy metabolism”

19.4 Sugar-nucleotide biosynthesis and conversions - from category “Purines, pyrimidines, nucleosides, and nucleotides”
