# Supplementary material for: Automatic Assignment of Prokaryotic Genes to Functional Categories Using Literature Profiling
Source: PLoS One. 2012 Oct 15;7(10):e47436. doi: 10.1371/journal.pone.0047436 (PMC3471813; doi:10.1371/journal.pone.0047436)
Supplement: Table S1 — Training dataset – Used to train the SVM classifier and to perform the cross-validation. (DOC) [file pone.0047436.s003.doc]

TRAINING DATASET – USED TO TRAIN THE SVM CLASSIFIER AND TO PERFORM THE CROSS-VALIDATION

| **Gene** | **Original Category** | **PubMed IDs** |
| --- | --- | --- |
| ACIRA0001_0069 | 8 | 12087099; 6370952; 8917463; 7622474; 7663384; 8241179; 8100227; 6715379 |
| ACIRA0001_0231 | 9 | 21380499; 21378393; 21377632; 21354840; 21325744; 21320176; 21314018; 21310443; 21265823; 21264498 |
| ACIRA0001_0329 | 8 | 20979355; 20937819; 20603809; 20398675; 18454933; 19006332; 18943005; 18809504; 16803984; 18638447 |
| ACIRA0001_0640 | 6 | 20975945; 10523315; 15212893; 15136039; 11106026; 10669597; 12736; 10361305; 3011407; 9398514 |
| ACIRA0001_0772 | 8 | 21336929; 20026072; 16499623; 16023116; 12186751; 11004571; 9443811; 9417993; 8786138; 7584606 |
| ACIRA0001_1070 | 4 | 21394325; 21390913; 21383483; 21381755; 21354192; 21350490; 21350489; 21345107; 21344175; 21343350 |
| ACIRA0001_1228 | 5 | 21087930; 2138605; 15580782; 10224133; 9647837; 10200269; 17450323; 14585934; 7972072; 7493934 |
| ACIRA0001_16007 | 8 | 20414649; 20349330; 19306572; 19186822; 9371464; 14514049; 18043954; 18022671; 17986771; 17420595 |
| ACIRA0001_1664 | 12 | 21393446; 21386086; 21378315; 21372607; 21345437; 21333724; 21321316; 21319863; 21319304; 21317537 |
| ACIRA0001_1778 | 15 | 21183667; 20110293; 8051064; 19170879; 17268768; 16765837; 8486283; 10639406; 15702929; 12664264 |
| ACIRA0001_2008 | 3 | 19895819; 9844000; 11114900; 15133094; 1362446; 2551782; 9767592; 1313415; 1676385; 5432063 |
| ACIRA0001_2403 | 12 | 21378190; 21376091; 21361789; 21340736; 21325275; 21300775; 21257463; 21255863; 21208912; 21188603 |
| ACIRA0001_2691 | 12 | 20693979; 20478578; 19940503; 19913053; 9875225; 18429003; 17919550; 17385712; 17306612; 16983347 |
| ACIRA0001_2796 | 8 | 21388532; 21387398; 21366264; 21365771; 21315316; 21314614; 21299249; 21283155; 21281348; 21272045 |
| ACIRA0001_2809 | 11 | 20963614; 20665904; 19326202; 18348984; 19009320; 18479969; 18348984; 15518819; 17387010; 16364752 |
| AF_0173 | 8 | 21390322; 21378381; 21375592; 21325058; 21268349; 21265736; 21250660; 21199673; 21192786; 21179059 |
| AF_0248 | 8 | 20868295; 20675490; 20445244; 20349330; 20179327; 1324665; 20017731; 19899808; 1512208; 19725515 |
| AF_0288 | 5 | 8180221; 1332767; 1654509; 2153377; 2549047; 2828368; 3032273; 3019265 |
| AF_0329 | 12 | 20200052; 18479955; 18080018; 17367717; 15464727; 15781470; 12974639; 12805367; 6413505; 12437354 |
| AF_0358 | 6 | 15656976; 12638184; 11737776; 11732635; 10915862; 8621533; 10666338; 1373853; 9784390; 8294433 |
| AF_0508 | 4 | 20638940; 10635304; 19367118; 19273469; 15122361; 9440322; 17368731; 15706095; 17250832; 16945541 |
| AF_0537 | 13 | 21329764; 21186327; 20978909; 20949037; 20943571; 20943505; 20943150; 20618870; 20601683; 20580850 |
| AF_0725 | 2 | 19754882; 16866557; 15522295; 11215515; 11215515; 10585141; 1090845; 2077690; 9778368; 9665173 |
| AF_0727 | 2 | 11215515; 6312261; 7606163; 1731915; 6312261; 6167991 |
| AF_0745 | 14 | 20874647; 20473969; 20066562; 17910097; 17583536; 17578676; 16675542; 16255137; 16096695; 6142052 |
| AF_0853 | 12 | 10542056; 10193948; 9615190; 9503455; 7940158; 1353016; 1995641; 2576976; 3052805 |
| AF_0855 | 8 | 21236692; 20528775; 20465256; 19091740; 19020988; 18725329; 12517448; 17215140; 12962632; 12562758 |
| AF_0993 | 6 | 21363882; 21357745; 21296653; 21292986; 21283680; 21276791; 21268715; 21267443; 21266355; 21239234 |
| AF_1175 | 9 | 18258771; 16887802; 15159576; 11356167; 9490742; 9804184; 9488698; 7494405; 2318792; 7150615 |
| AF_1648 | 9 | 18579827; 17855635; 16971696; 17510911; 12117714; 17374725; 17332886; 16873929; 16873928; 10518522 |
| AF_1811 | 14 | 19299651; 18260100; 10074353; 17439323; 10716711; 17324932; 15996793; 15670151; 15566465; 14966129 |
| AF_1840 | 12 | 20716180; 20621724; 20521764; 20363127; 20334618; 20207144; 20142044; 20038112; 18781344; 17523140 |
| AF_18555 | 2 | 21393854; 21335605; 21185305; 21166461; 21098490; 21034554; 20956520; 20841425; 20656883; 20651329 |
| AF_1883 | 4 | 21241026; 21187464; 21133625; 21124441; 21115899; 21094854; 21055388; 21046154; 21040738; 21036735 |
| AF_2036 | 8 | 21109633; 20524628; 20398622; 20221790; 20018841; 19914577; 8254673; 19453276; 10487920; 19353431 |
| AFE_0022 | 18 | 21393446; 21383163; 21381722; 21376393; 21356245; 21344404; 21340031; 21339645; 21316269; 21309120 |
| AFE_00420 | 8 | 20160912; 17157320; 14638692; 11316579; 10807520; 9727038; 1908789; 9038189; 7567952; 7947791 |
| AFE_0674 | 18 | 21350490; 21299248; 21041493; 21035449; 20981744; 20865003; 20737472; 20618092; 20562277; 20545747 |
| AFE_0678 | 18 | 21156049; 21148559; 21103969; 21091508; 20977453; 20960970; 20941577; 20935164; 20934246; 20869350 |
| AFE_0929 | 18 | 20028804; 16043697; 18633280; 18272427; 8300209; 12754224; 12783268; 11350161; 2941675 |
| AFE_10942 | 4 | 21278275; 21197957; 21190852; 21097621; 21031321; 21031305; 21031302; 21028779; 20954236; 20873201 |
| AFE_1096 | 15 | 20012992; 11756427; 10829079; 16549657; 15141956; 12848431; 7756254; 10806366; 2055470; 9487693 |
| AFE_1142 | 18 | 21350490; 21249122; 21041493; 20981744; 19342493; 18849422; 20424311; 20211889; 20111865; 19843215 |
| AFE_12727 | 8 | 20160912; 19996100; 16531404; 14742428; 14644451; 12603319; 12031470; 11955070; 11935326; 11781147 |
| AFE_1387 | 4 | 20880145; 20487027; 20472796; 6328516; 19744238; 16957187; 19025575; 18848959; 17768240; 10964570 |
| AFE_1625 | 3 | 19883124; 17975091; 15632436; 12000771; 8982270; 5167087; 3549716; 14907713 |
| AFE_1671 | 15 | 20671064; 20540986; 19424634; 17094466; 15792646; 14760686; 8168923; 2265756; 2540154 |
| AFE_1704 | 11 | 19899628; 18815788; 16658221; 18253751; 18069343; 17459613; 17039376; 10852890; 15772148; 15771247 |
| AFE_1705 | 11 | 21061622; 20873200; 18615728; 20167267; 15173120; 20005797; 9254694; 19473321; 15983781; 19184476 |
| AFE_1981 | 3 | 20081027; 9767582; 1809829; 3768954; 6120442 |
| AFE_2065 | 8 | 20456655; 17981801; 328341; 1560008; 1688555; 3090936; 7440562 |
| AFE_2779 | 12 | 21258844; 14617630; 19576997; 19130269; 18702072; 18048940; 17975309; 10419525; 15161914; 15981243 |
| AFE_2843 | 3 | 21284804; 21265748; 21098028; 21097635; 21097603; 20796283; 20722599; 20656905; 20616068; 20455262 |
| AFE_2986 | 18 | 21364903; 21329797; 21327038; 21313786; 21222360; 21217201; 21212668; 21209325; 21179865; 21179864 |
| AFE_3156 | 11 | 2536143; 20154109; 20016970; 19761806; 17406229; 17406228; 16894890; 16523664; 15908923; 10448040 |
| AFE_31697 | 8 | 21207115; 20170126; 20079748; 19835359; 17746925; 19716624; 19432488; 18625203; 18281989; 10639366 |
| AFE_3183 | 8 | 20307488; 18824464; 16887609; 16819830; 16514162; 16211847; 16171391; 10907557; 15476980; 12803488 |
| AHA_0064 | 15 | 20855615; 7010115; 16166384; 2066330; 15528658; 10943406; 1370665; 9218770; 8969172; 8804390 |
| AHA_0104 | 9 | 21385872; 21343303; 21324386; 21302143; 21292322; 21281409; 21256197; 21255373; 21251176; 21223324 |
| AHA_0421 | 15 | 15342582; 11244072; 10331874; 15583165; 15018101; 9786195; 9179850; 8824586; 3243435 |
| AHA_0465 | 12 | 21391788; 21383917; 21372483; 21372438; 21362485; 21348611; 21345766; 21344476; 21329005; 21328464 |
| AHA_0501 | 12 | 19612975; 16075683; 15717599; 15605827; 15514709; 14529758; 12940146; 12325260; 12204299; 12070913 |
| AHA_0521 | 3 | 20797400; 20349311; 9227854; 20199591; 20118254; 20070257; 20004668; 19891507; 19850919; 19820722 |
| AHA_0807 | 18 | 21248342; 21097578; 21072388; 20886437; 20878478; 20820996 |
| AHA_0816 | 15 | 20656781; 20118252; 10966457; 18792681; 12453229; 10781568; 16949866; 9781871; 12123461; 10094700 |
| AHA_0854 | 18 | 21393450; 21391724; 21339577; 21330430; 21321143; 21315184; 21296967; 21268348; 21156129; 21152583 |
| AHA_0968 | 18 | 10873790; 21233811; 20969509; 20964254; 20835684; 20722469; 20623607; 20605181; 20593420; 20528568 |
| AHA_09774 | 8 | 21393864; 21393839; 21390509; 21385872; 21367878; 21360139; 21359176; 21346408; 21327387; 21310505 |
| AHA_1020 | 4 | 20980482; 20823200; 20639332; 20487271; 20199591; 9675698; 1385123; 19508558; 16923899; 19028568 |
| AHA_1165 | 8 | 17316685; 16963438; 16882299; 16298387; 15588821; 12940821; 10482494; 10692383; 10913144; 10844646 |
| AHA_1305 | 12 | 20965918; 20720417; 20705730; 20554187; 20381609; 20026068; 19960337; 19883788; 18426891; 9864012 |
| AHA_1428 | 1 | 21318117; 21216350; 21117640; 21116453; 21078554; 21076535; 21071955; 21060940; 20965581; 20880190 |
| AHA_1579 | 15 | 21031306; 16601099; 10829079; 16990134; 15916958; 15740742; 12952558; 8231809; 10844680; 10632884 |
| AHA_1753 | 12 | 21237246; 21185091; 20652663; 16798948; 19834685; 19589362; 19508204; 19405998; 8449925; 14996277 |
| AHA_1912 | 6 | 21383952; 21353395; 21319196; 21293478; 21274285; 21266189; 21251882; 21187137; 21149446; 21123297 |
| AHA_2291 | 12 | 20079837; 7141712; 7763298; 16308686; 15047720; 14760686; 12698553; 12177052; 11351281; 10756180 |
| AHA_2398 | 15 | 12060687; 16194239; 15228517; 8951818; 12826660; 2283426; 12147687; 9108148; 8624513; 9421491 |
| AHA_2847 | 4 | 10785634; 17492271; 15175291; 15817382; 12940991; 1937792; 10966110; 10320579; 8830263; 8200538 |
| AHA_29331 | 8 | 21178486; 21146533; 20515684; 20484564; 20351104; 20131326; 19998026; 18539294; 19144319; 19689355 |
| AHA_2938 | 18 | 20649844; 20628435; 20460828; 20430841; 20147439; 19833843; 19497955; 19255943; 18985798; 18717763 |
| AHA_2940 | 12 | 12950156; 12207045; 12200051; 11001812; 9019409; 15781549; 14731855; 2649495; 2943218 |
| AHA_2956 | 15 | 19011896; 16438983; 12962497; 12662307; 12269832; 8799194 |
| AHA_3120 | 12 | 14731284; 12235139; 16796689; 15550391; 14731284; 10222271; 11919638 |
| AHA_3330 | 12 | 21320715; 21156839; 21117492; 21059345; 20942967; 21288105; 20735174; 20723205; 20722626; 20694749 |
| AHA_3338 | 12 | 20688826; 20064164; 19519768; 19415239; 19013157; 18522945; 2170332; 12051947; 17322183; 17107419 |
| AHA_3376 | 18 | 17158685; 17996893; 17986083; 16844687; 3033433; 16645306; 16445940; 16225868; 14872322; 15469514 |
| AHA_3478 | 12 | 21228481; 21110062; 21090506; 21048858; 20886828; 20869404; 20662376; 20414794; 20370321; 20236165 |
| AHA_3483 | 12 | 15752701; 19291145; 10422885; 16477138; 16475002; 7006083; 15752701; 15450853; 15262231; 15183887 |
| AHA_3547 | 15 | 20662783; 18586946; 16960358; 16091943; 10517579; 12675810; 12100554 |
| AHA_3574 | 6 | 20942908; 20573047; 19077009; 5389100; 18811234; 17097293; 16204239; 16159328; 16155110; 16099603 |
| AHA_3791 | 9 | 20972840; 20932966; 20602678; 20813182; 20696151; 20687589; 20631298; 20616715; 20605907; 20860563 |
| AHA_3827 | 4 | 20722420; 20545753; 19174888; 18200850; 17904239; 17410780; 11348068; 17144297; 12694606; 16247670 |
| AHA_3908 | 12 | 11919638; 14688122; 16949246; 15943900; 9632569; 8830688; 10632892; 10589719; 6347072; 8168923 |
| AHA_4285 | 18 | 17322211; 11741847; 11527960; 12060600; 11461963; 11318953; 10799909; 10763412; 10762088; 10594060 |
| ATORI0001_0165 | 3 | 20824214; 20707404; 19825675; 18597864; 18508770; 18043952; 17955483; 17934909; 15608179; 16158237 |
| ATORI0001_0660 | 3 | 21356463; 21061834; 21057479; 21040738; 20980568; 20975069; 20940192; 20919961; 20890816; 20890421 |
| ATORI0001_08897 | 8 | 21388130; 21347668; 21279470; 21204877; 21030825; 20945834; 20869755; 20728445; 20467246; 20438129 |
| ATORI0001_1067 | 18 | 21378967; 21375947; 21339619; 21330114; 21295581; 21292012; 21270472; 21268855; 21255036; 21238459 |
| ATORI0001_1150 | 15 | 21317319; 21151985; 20817115; 20685086; 20547748; 19627503; 19324528; 18420434; 18957579; 10992472 |
| ATORI0001_1439 | 12 | 21315085; 21118527; 20947885; 20874569; 20817636; 20732391; 20639698; 20607520; 20604530; 20601056 |
| ATORI0001_1447 | 2 | 20576686; 14679236; 18186650; 11125067; 16905099; 7929447 |
| ATORI0001_15965 | 2 | 21282208; 20189102; 20064433; 16321944; 15840497; 15649375; 11468415; 9701598 |
| BAA_0061 | 13 | 20197408; 20234387; 10656263; 11388898; 18391411; 18342886; 18247350; 15317870; 17619020; 17516842 |
| BAA_0070 | 4 | 20870765; 20644139; 19635793; 18832310; 10610805; 10978550; 12457697; 15165235; 15659160; 12940998 |
| BAA_0083 | 2 | 20054118; 19389784; 10829079; 234963; 15500462; 1644759; 8096767; 1637823; 3117785; 2139795 |
| BAA_0105 | 13 | 21135240; 20357079; 17301007; 18391952; 17848100; 17982176; 18775898; 11283358; 2426258; 18442140 |
| BAA_0112 | 13 | 20399793; 11118225; 9169555; 10747797; 11160812; 14698286; 11532164; 11238976; 10476952; 8264530 |
| BAA_0113 | 13 | 12514741; 11238976; 10801481; 9657144; 8136022; 8251501 |
| BAA_0116 | 13 | 20399793; 21136979; 10937989; 17891922; 11733066; 520572; 17536671; 17199978; 17022624; 16739990 |
| BAA_0122 | 13 | 21219451; 9380668; 17027976; 16813165; 10890005; 11686933; 9331423; 9331418; 9292501; 1637863 |
| BAA_0125 | 13 | 20413480; 19111651; 10937989; 7516168; 1258377 |
| BAA_0127 | 13 | 20065060; 18757750; 16318913; 11483524; 15127365; 12946348; 11296296; 11511371; 10361087; 4620021 |
| BAA_0128 | 13 | 11733066; 15308339; 9695947; 12581648; 11716492; 8264530; 8457554; 1499563; 173425; 2191716 |
| BAA_0133 | 13 | 15473684; 1712983; 16390447; 15561149; 3297687; 3549294; 6084168 |
| BAA_0140 | 13 | 21156960; 15522293; 12914937; 12823975; 11703173; 1988674; 8805594; 7662106; 8490021; 8223574 |
| BAA_0150 | 13 | 10572302; 11168885; 8722036; 354966; 2829909 |
| BAA_0161 | 13 | 19013179; 14675435; 8536310; 8223574; 1812070; 2438658; 6818528; 2429836; 6374660 |
| BAA_0189 | 5 | 20709015; 20536206; 20506529; 20451305; 20416269; 9844142; 19957429; 19903862; 19843219; 19757168 |
| BAA_01931 | 8 | 20676631; 19560494; 18619846; 10715008; 19399347; 9135111; 187575; 6388497; 9254694; 12921536 |
| BAA_04494 | 8 | 21393839; 21307593; 21299880; 21284213; 21278295; 21243934; 21240541; 21229460; 21182289; 21139204 |
| BAA_0474 | 18 | 20975991; 20836889; 20809990; 19883117; 19207208; 10498699; 15100400; 17107946; 10347003; 16645309 |
| BAA_0478 | 12 | 21189343; 21115495; 21095572; 21075926; 12382110; 20419406; 20417202; 20207389; 20185723; 20100489 |
| BAA_0957 | 8 | 21272045; 21206028; 21178165; 21126600; 21076097; 21029324; 20861234; 20851994; 20824484; 20731795 |
| BAA_0990 | 3 | 19926656; 18720489; 18166249; 18028311; 1735716; 17005012; 16532637; 16121336; 16028518; 10658653 |
| BAA_0991 | 3 | 21178417; 7600011; 18395220; 18166249; 17005012; 16755617; 16532637; 9276929; 10658653; 15474715 |
| BAA_11217 | 8 | 20938718; 20367472; 20045141; 16350018; 19727706; 19670813; 18438874; 18424424; 17156016; 16891140 |
| BAA_12180 | 4 | 21367479; 21246233; 20977881; 20734145; 20607520; 20333498; 20233932; 20184451; 20169403; 20129048 |
| BAA_1413 | 3 | 18480329; 18322733; 18236038; 17577057; 17383694; 16549657; 16333343; 10417208; 1102538; 12641963 |
| BAA_1414 | 4 | 21345802; 21296943; 21264304; 21227395; 21205308; 21204945; 21169442; 21144051; 21126366; 21111160 |
| BAA_1536 | 18 | 21210640; 20666386; 20511233; 20459315; 19948126; 19850002; 19469573; 18189394; 18804465; 18793176 |
| BAA_17020 | 4 | 21367479; 21246233; 20607520; 20368405; 20184451; 20151761; 19778059; 19732898; 19628563; 19626709 |
| BAA_1776 | 3 | 20707404; 20335363; 20201406; 19279143; 19883124; 19857612; 19825675; 20110776; 19414810; 19298858 |
| BAA_2307 | 12 | 20630200; 7934823; 19383693; 16698798; 16678787; 15378526; 12867413; 11007775 |
| BAA_2318 | 8 | 20564560; 20192272; 18318836; 17568739; 17172314; 17042912; 16828464; 16790434; 9807838; 16132097 |
| BAA_2446 | 13 | 21098258; 21149735; 20942128; 20387531; 20018267; 19761773; 19702327; 19502729; 16754626; 19398261 |
| BAA_2553 | 12 | 20819954; 20460427; 20082075; 19915793; 19583493; 19577318; 18753673; 19301317; 18790006; 17601991 |
| BAA_27374 | 8 | 21310076; 21102653; 20957169; 20831280; 20668405; 20652693; 20419375; 20228117; 20222446; 20208168 |
| BAA_3039 | 6 | 21219854; 21040729; 20952386; 20878118; 14502125; 20598295; 20570862; 20562282; 20554056; 20543070 |
| BAA_3182 | 4 | 18694737; 18251901; 10090757; 12121459; 11169136; 10545213; 9688602; 2165985; 8279538; 8437891 |
| BAA_3251 | 12 | 14734171; 12183460; 12059959; 12054669; 10574456; 9735342 |
| BAA_3348 | 12 | 20807532; 20383020; 10508786; 15504408; 15491154; 15291820; 12586941; 12377778; 12206761; 10781538 |
| BAA_3411 | 13 | 21279988; 20942128; 20942127; 19738232; 18065539; 20655695; 20360392; 20228783; 20132829; 20128644 |
| BAA_3896 | 6 | 20843803; 20732909; 19616486; 19542005; 18992265; 15249553; 8706136; 11106395; 6997501; 12226667 |
| BAA_3948 | 12 | 17244626; 17143514; 15944156; 15681797; 12067222; 7806544; 1671040; 7819328; 8463315; 1733942 |
| BAA_3988 | 13 | 21278155; 17185548; 17051149; 15459648; 12068815; 2199796; 1386558; 4942549; 7013783 |
| BAA_39962 | 8 | 20448188; 18392745; 17640871; 1986797; 1276977 |
| BAA_39972 | 8 | 20448188; 18392745; 17640871; 10657297; 1986797; 1276977 |
| BAA_4001 | 13 | 10937989; 9491077; 14888646; 173425; 2665813; 2653827; 6381990; 792456; 773694; 6163479 |
| BAA_4077 | 3 | 20804196; 20024979; 19403924; 19369074; 19198900; 19014883; 19007109; 18704940; 18557704; 18259126 |
| BAA_4243 | 4 | 7704255; 16561900; 291033; 6801021; 3931691; 18945; 942051; 5432063 |
| BAA_4315 | 18 | 21179170; 21103971; 20817733; 20566650; 20357029; 20100283; 19828839; 19741195; 19682536; 19653651 |
| BAA_4509 | 13 | 11904182; 9294008; 9044258; 2422386; 7529559; 1379176; 1614849; 1742360; 6351726; 173425 |
| BAA_4566 | 13 | 20600110; 20149799; 18400176; 8608120; 10890005; 11530930; 1091919; 8223574; 7683367; 7916699 |
| BAA_4621 | 1 | 21047785; 20937239; 20720017; 20628880; 20529854; 15375207; 20379751; 20179139 |
| BAA_4692 | 13 | 15473684; 16285924; 3884043; 8251501; 1339289 |
| BAA_4714 | 2 | 21245367; 20978940; 20955962; 20932248; 20850424; 20580577; 20536026; 20455047; 20300940 |
| BAA_4720 | 12 | 21378284; 21372727; 21352200; 21351429; 21331046; 21327682; 21327498; 21310076; 21304989; 21303546 |
| BAA_4788 | 18 | 20643901; 16641446; 15870467; 12603748; 11544234; 18481184; 18394148; 18227063; 18086187; 8973347 |
| BAA_4827 | 13 | 20097853; 18037435; 17002278; 16739990; 15916597; 12368106; 11470155; 8628231; 1706660; 2665813 |
| BAA_4922 | 13 | 21209200; 20864539; 20956970; 20839808; 20823226; 20690656; 20676842; 20598274; 12446813; 20340150 |
| BAA_52505 | 2 | 20942799; 20460376; 19810706; 15952888; 16905347; 17941825; 17642475; 17350000; 15964837; 1400307 |
| BAA_5281 | 9 | 19213219; 18281324; 15235808; 3136142; 10387003; 9813046; 7988741; 8253773; 8399347; 8386511 |
| BAA_5474 | 5 | 21208503; 21134486; 21082203; 20735352; 20512387; 20305127; 20233922; 20095974; 20013010; 19997761 |
| BAA_A0035 | 11 | 19951359; 10801134; 19346162; 2238045; 18557832; 18349516; 16170154; 16763758; 16621844; 16150738 |
| BAA_A0157 | 11 | 21072564; 20943007; 20890269; 20737907; 20562864; 20428106; 16482214; 20227411; 17630835; 19628563 |
| BCI_0124 | 15 | 20883745; 18403126; 17449274; 16371317; 15389551; 15170488; 12948652; 12366528; 9841634; 9767719 |
| BCI_0213 | 5 | 17835129; 9838063; 16761196; 16387658; 1318389; 15065880; 15065853; 12363041; 12007424; 11921096 |
| BCI_0233 | 6 | 20978197; 20861182; 8662184; 20068132; 7906418; 18929140; 19757171; 19664552; 19129247; 9649448 |
| BCI_0395 | 14 | 20088569; 18255096; 16230416; 8385948; 11108809; 10777550; 9776180; 8798492; 7563070; 7945284 |
| BCI_0419 | 8 | 18775777; 11006847; 3141411; 1830580; 9741089; 9533458; 1555599; 9304875; 8772174; 8736535 |
| BCI_0534 | 12 | 20836086; 20512978; 19706448; 19717451; 18945679; 18268014; 10203757; 17179147; 16513633; 12183369 |
| BCI_0585 | 3 | 20190047; 20118250; 19334375; 19284999; 19063962; 18599819; 17658458; 17283383; 17125974 |
| BCI_06112 | 2 | 20180116; 16233126; 11006082; 10913707; 11006082 |
| CC_0009 | 6 | 21048928; 20661249; 19892776; 10607676; 12379112; 18432238; 19338370; 9705516; 18432238; 16430210 |
| CC_0675 | 3 | 11368909; 12674476; 12674475; 12674474; 12615971; 9788878; 9645939; 7581330; 1409695; 7931367 |
| CC_0723 | 15 | 21315771; 21284862; 21257771; 21217003; 21193607; 21078995; 21037181; 20951027; 20946847; 20946846 |
| CC_07974 | 8 | 19533311; 16333341; 17159214; 16828055; 4018385; 16307283; 15721281; 11106394; 14660638; 12963347 |
| CC_09681 | 8 | 21297118; 21244038; 21176987; 21098515; 20946831; 20886886; 20882536; 20825165; 20809622; 20727919 |
| CC_1007 | 3 | 20833802; 20657621; 20069463; 19214794; 9335282; 1735716; 16258187; 16111836; 6209263; 12832111 |
| CC_1402 | 8 | 20952576; 11334784; 18353797; 17143652; 11864982; 9880817 |
| CC_1404 | 8 | 20952576; 19889029; 11334784; 17143652; 14503876; 377280; 12196157; 12070166; 11864982; 9880817 |
| CC_1569 | 8 | 17680234; 16128611; 15112989; 7515059; 8185833; 3110160 |
| CC_1588 | 13 | 20354154; 20129918; 19925456; 19894214; 19874048; 19746363; 19627989; 7578154; 19199329; 18001138 |
| CC_1799 | 9 | 21356516; 21299081; 21284337; 21277994; 21271628; 21254598; 21191070; 21175091; 21149724; 21146149 |
| CC_1936 | 2 | 21390227; 21343300; 21214190; 21187331; 21172299; 21116996; 21087640; 20947019; 20937134; 20933261 |
| CC_2198 | 12 | 20609366; 20592285; 20154099; 19139197; 19819873; 19527767; 19508204; 18482986; 18539590; 18393803 |
| CC_2241 | 12 | 21266175; 20139072; 20073505; 18544350; 8986785; 18430135; 8899987; 15955783; 17512907; 15550391 |
| CC_2894 | 4 | 20122935; 19767828; 19711344; 19376231; 19269260; 20707217; 19101977; 19081934; 15549093; 17216057 |
| CC_3098 | 17 | 21357290; 21277915; 20971899; 20955556; 20880327; 20864473; 20811050; 20633642; 7501460 |
| CC_3106 | 8 | 21371789; 17928627; 17651438; 17504214; 17355947; 17075075; 12107052; 15044825; 12624214; 12356312 |
| CC_3381 | 12 | 21394741; 21393041; 21368222; 21347827; 21332170; 21320870; 21319259; 21315771; 21314817; 21301034 |
| CLB_0555 | 8 | 17519437; 9762080; 10525296; 7744873; 1658003; 1650362; 1905094; 16346756 |
| CLB_0739 | 8 | 18845259; 16584639; 8355992; 15826505; 12107963; 11597772; 10103005; 10094940; 2420379; 9169128 |
| CLB_0771 | 18 | 21350490; 21350473; 21349348; 21337355; 21333939; 21322478; 21308846; 21299248; 21268857; 20843249 |
| CLB_10974 | 8 | 19962135; 18671744; 18661303; 15322771; 12901010; 12030703; 11347275; 11267643; 11118651; 10222181 |
| CLB_12334 | 8 | 21369980; 21355000; 21335027; 21327413; 21315381; 21314956; 21298405; 21279650; 21229334; 21192716 |
| CLB_1466 | 12 | 19645670; 18539590; 15549168; 14965309; 11831855; 11570886; 8621556; 6321177; 1678740; 20501241 |
| CLB_1724 | 4 | 21365227; 21317325; 21092197; 21057011; 19930460; 8655545; 19616612; 11229921; 16597992; 9384377 |
| CLB_1882 | 18 | 19818021; 19234723; 18641624; 18511655; 10829230; 16879982; 11292832; 1768149 |
| CLB_2444 | 11 | 21375771; 21349326; 21339813; 21335340; 21318311; 21317336; 21285351; 21276096; 21257810; 21241792 |
| CLB_2569 | 8 | 21030403; 20884613; 20840841; 20839327; 20838636; 20837476; 20670834; 20637717; 20599533; 20592242 |
| CLB_2596 | 4 | 17572072; 16272391; 16238624; 11278070; 15491362; 6259129; 11929518; 11454205; 11260478; 1372311 |
| CLB_2830 | 2 | 20955574; 20822113; 20230506; 19942660; 12910462; 3023344; 2171433; 18500821; 7819217; 18186482 |
| CLB_3127 | 3 | 20864531; 20384682; 20073482; 20030728; 19737353; 10361286; 19729404; 19709344; 19684067; 10556321 |
| CPS_0142 | 18 | 21394896; 21394428; 21393109; 21392144; 21391781; 21389980; 21385903; 21385548; 21381611; 21373260 |
| CPS_0464 | 1 | 12594532; 10692366; 15164414; 2258657; 7297539; 7297537 |
| CPS_0630 | 5 | 21338368; 21318877; 21303766; 21208503; 21140446; 21134486; 21095459; 21082203; 21048303; 21043115 |
| CPS_0724 | 18 | 21203426; 21184851; 21126336; 20406859; 19744929; 10545125; 14960472; 12507466; 2645056; 18794801 |
| CPS_0751 | 12 | 19288442; 19116773; 12910392; 11878564; 7937158 |
| CPS_0956 | 12 | 21378058; 21206021; 21125383; 21112247; 21109561; 21071850; 20959808; 20813910; 20729362; 20727857 |
| CPS_0957 | 12 | 21378058; 21322032; 21278275; 21261628; 21216990; 21214540; 21206021; 21129201; 21125383; 21112247 |
| CPS_1508 | 4 | 20889747; 2030670; 8158647; 9234797; 18284590; 4631369; 9677288; 16677309; 16260786; 8107139 |
| CPS_1880 | 18 | 21134393; 20863830; 20720015; 20715055; 18845756; 20508181; 20470844; 7500034; 20394729; 20381597 |
| CPS_1887 | 8 | 20957036; 20559622; 20447995; 20096472; 20055505; 19711069; 19622650; 19436127; 19224199; 19202108 |
| CPS_1927 | 18 | 21338516; 21310784; 21278293; 10829079; 20601471; 20482592; 20387456; 20335169; 20299406; 11880056 |
| CPS_2344 | 8 | 21366264; 21338575; 21272045; 21252750; 21215280; 21206028; 21203928; 21148624; 21135193; 21126600 |
| CPS_2746 | 8 | 21329667; 21299212; 20889709; 20861021; 20858599; 20836848; 20697198; 20650894; 20593880; 20574584 |
| CPS_3523 | 1 | 14997577; 8809779; 7525561; 1978772; 2679804; 3894345; 6355484 |
| CPS_3580 | 12 | 21268853; 20920169; 20799977; 7501460; 20610410; 20433838; 20158612; 19797360; 19535917; 19238259 |
| CPS_3647 | 18 | 20525315; 11251106; 19088452; 7491264; 17158458; 15684415; 12816347; 12661057; 12473671; 11115132 |
| CPS_3842 | 18 | 21128790; 21097502; 21068392; 20962898; 20845974; 20812903; 20686069; 20634522; 20634426; 20619617 |
| CPS_4090 | 9 | 20937103; 19788655; 19267463; 19107395; 19103481; 18422622; 18340545; 17170116; 15170232; 10066830 |
| CPS_4164 | 15 | 21389343; 21384170; 21357539; 21346797; 21307285; 21248029; 21245479; 21224048; 21219458; 21179024 |
| CPS_4346 | 12 | 21332448; 21327044; 21036998; 20959450; 20953507; 20581826; 20581825; 20515644; 20487295; 20487271 |
| CPS_4497 | 15 | 7729414; 17916725; 10452797; 17628145; 17559414; 17434492; 10947842; 16919402; 16000707; 15670209 |
| DET_0079 | 8 | 21243405; 20530887; 19888999; 16840784; 18326677; 18044513; 17916062; 17233752; 17191498; 15294827 |
| DET_0083 | 11 | 21262357; 16181782; 18227257; 17367389; 16731525; 10722135; 10986230; 9316915; 9393713; 2548993 |
| DET_0186 | 8 | 21167813; 20688826; 20498375; 19959573; 860983; 18716757; 17975082; 16913910; 16529396; 16221580 |
| DET_0632 | 18 | 21122131; 20714133; 20693416; 20656779; 20613716; 20460825; 20204475; 20103563; 20035575; 19950388 |
| DET_0911 | 12 | 21385872; 21376699; 21376122; 21365622; 21351072; 21343297; 21342114; 21341524; 21320871; 21318132 |
| DET_0941 | 18 | 15850393; 12039966; 11299317; 9778127; 1325861; 1718037 |
| DET_1100 | 6 | 21391582; 21391199; 21391128; 21387067; 21383675; 21380558; 21377995; 21375684; 21375507; 21374707 |
| DET_1277 | 9 | 20590527; 20370610; 20178986; 20099411; 20080211; 19686777; 19571038; 19525201; 15461798; 19444866 |
| DET_1360 | 12 | 21258212; 21041443; 20862217; 20836734; 20708777; 20696889; 20616068; 20616021; 20603181 |
| DET_1400 | 12 | 21338918; 20552259; 20439464; 20036249; 19665474; 19075746; 18434193; 17565388; 17355436; 16973605 |
| DNO_0019 | 12 | 21258033; 21237246; 21212512; 20609366; 20592285; 20154099; 19819873; 19638462; 19527767; 19508204 |
| DNO_0110 | 3 | 20050159; 19507071; 18807220; 16788189; 17468768; 16452436; 20076642; 15100690; 8418033; 16624707 |
| DNO_0115 | 6 | 20937909; 20713134; 20592034; 20385564; 12560328; 2225075; 19820703; 11836238; 18822296; 17868027 |
| DNO_0119 | 18 | 20924357; 20487301; 20361665; 20361664; 20139185; 19824696; 11580842; 7592486; 18402772; 11481431 |
| DNO_0131 | 2 | 20194361; 19919179; 17178720; 16606627; 16218869; 15967800; 15450488; 11578923; 12975365; 12114526 |
| DNO_0282 | 11 | 20849300; 20509797; 16525046; 19441900; 19198779; 18473891; 17449649; 10688190; 17608468; 17326971 |
| DNO_0306 | 9 | 20956528; 20528952; 20455949; 20434430; 20185506; 19330542; 8863531; 18759116; 18691575; 11025668 |
| DNO_0330 | 4 | 21346797; 21339285; 21284980; 21272569; 21255377; 21204254; 21190525; 21182296; 21178952 |
| DNO_0446 | 12 | 21385873; 21369489; 21364893; 21330002; 21327109; 21326824; 21315077; 21296756; 21261285; 21257796 |
| DNO_0737 | 8 | 21389620; 21389104; 21388532; 21385584; 21382272; 21382109; 21378396; 21377964; 21377525; 21375472 |
| DNO_0743 | 11 | 21079776; 20056615; 17428497; 19347993; 1853558; 16377618; 6283090; 17428497; 16618107; 10944228 |
| DNO_0784 | 11 | 15165237; 12798232; 11162180; 8144474; 329561 |
| DNO_0891 | 3 | 17985115; 17468768; 17482513; 15100690; 9159491; 1683004; 8550520; 7642131; 1588814 |
| DNO_0916 | 12 | 21281954; 21245143; 21056617; 21183694; 20944414; 20920334; 20857366; 20806779; 20709345; 20682773 |
| DNO_1013 | 2 | 20955109; 20828611; 20709841; 20626653; 17399977; 20227489; 20204741; 20175988; 20170652; 7073551 |
| DNO_1038 | 11 | 21059214; 20704513; 19810044; 19370753; 19339126; 16689791; 17158767; 10358166; 1660569; 7881904 |
| DNO_1045 | 11 | 19131699; 11024184; 18160019; 17326971; 17158767; 16901968; 16674971; 15917450; 15680412; 12086672 |
| DNO_1048 | 13 | 17889642; 17194931; 7535280; 15522293; 14729335; 12823975; 11341947; 10753109; 10742169; 10523206 |
| DNO_1343 | 18 | 21227585; 18670623; 20154136; 19851727; 1500854; 19640852; 19405026; 19118354; 17030797; 11162101 |
| EF_0252 | 3 | 20795394; 20586063; 20566764; 20384696; 20338254; 8662184; 20105277; 20061761; 19818020; 19799629 |
| EF_0264 | 14 | 21383199; 21381155; 21364483; 21331772; 21315812; 21182840; 21151976; 21124792; 21074544; 21073651 |
| EF_0302 | 12 | 18946116; 15378750; 12741537; 1397329; 9074504; 11204766; 10208803; 13890599; 8620487; 8568031 |
| EF_0362 | 3 | 21357479; 21327845; 21307593; 21303815; 21282899; 21240541; 21167943; 21155389; 21149550; 21131525 |
| EF_0575 | 18 | 20658302; 15870467; 18691556; 19151332; 19067785; 18690712; 18632116; 7811392; 17953587; 3447015 |
| EF_0615 | 11 | 21156198; 20890269; 20547379; 12169598; 16923390; 11029001; 16340015; 15500249; 9869563; 2553542 |
| EF_0680 | 3 | 21390233; 21379570; 21301102; 21294378; 21276045; 21266781; 21255117; 21220359; 21216228 |
| EF_0685 | 12 | 5160755; 20005877; 16410343; 10669849; 8602167; 9426607; 8914512; 1371110; 7559654; 7544801 |
| EF_0883 | 6 | 21097613; 1903929; 16622063; 16002087; 15556628; 2536712; 11679082; 11585815 |
| EF_09015 | 2 | 20955688; 20593767; 20560533; 11180061; 19559030; 19433215; 10961912; 19129660; 19055484; 16666457 |
| EF_1002 | 4 | 20502438; 20453092; 17326815; 19903201; 19737354; 19698693; 1925029; 19654604; 8181761; 1943780 |
| EF_1072 | 15 | 20581213; 19007420; 11690652; 10679470; 16524589; 15807530; 14596799; 14596799; 10551881; 10613841 |
| EF_11301 | 8 | 20549541; 19593556; 17979299; 18436321; 17436647; 9973343; 16428816; 16289362; 11073907; 15978081 |
| EF_13550 | 8 | 21253866; 21036145; 10672375; 20160912; 20022530; 19594830; 19464573; 17532339; 9324032; 17667915 |
| EF_13560 | 8 | 21296938; 20385101; 20160912; 19383527; 17532339; 18675788; 18362922; 17960497; 17157320; 12208141 |
| EF_1597 | 4 | 21378183; 20369357; 19924887; 19917674; 2846034; 18070874; 18943053; 19000033; 18980183; 18577539 |
| EF_1660 | 8 | 12031470; 11507102; 11448970; 10745006; 10187830; 9582350; 9546032; 9375800; 9381974; 9344245 |
| EF_1661 | 8 | 17314104; 16365091; 11168412; 10832633; 8652022; 7896739; 8274006; 1959888; 2001711; 2917566 |
| EF_1663 | 9 | 16547651; 12827525; 11400055; 1828889; 2945440 |
| EF_1855 | 11 | 20543074; 19671540; 19018586; 16133337; 15643941; 10319488; 8598202; 12749837; 9352905; 11471832 |
| EF_1920 | 18 | 17643228; 11803016; 10525738; 1512189; 9278503; 9302020; 9826185; 1482126; 8955408; 9705983 |
| EF_1964 | 8 | 21210726; 20868568; 21143936; 21135193; 21112638; 21106768; 21078494; 21076097; 21073742; 21062461 |
| EF_19910 | 4 | 21367479; 20806243; 20633642; 20607520; 20232878; 19624711; 19062645; 18803257; 17516097; 17038794 |
| EF_2198 | 3 | 18499663; 18210176; 15552059; 15226299; 15165190; 14646108; 12686644; 11937332; 11846551; 10610795 |
| EF_2320 | 11 | 20646510; 12867445; 18949133; 9661666; 18579153; 11152613; 16470371; 8469723; 15737404; 15225878 |
| EF_2580 | 14 | 19890726; 19597814; 19490017; 18642247; 18636689; 18556813; 17302173; 10397864; 17124633; 16497383 |
| EF_3037 | 12 | 21128822; 20969977; 20946870; 20944418; 15980438; 20652663; 20440617; 20389065; 20346382; 20334618 |
| EF_3214 | 17 | 20472641; 15328017; 11856747; 7479068; 9388478; 9046088 |
| EF_A0003 | 11 | 21206088; 20472796; 20405119; 20238268; 20174458; 20161086; 20097915; 19917674; 19846887; 19396961 |
| EF_A0004 | 11 | 20660665; 20472796; 18245424; 21255274; 19415237; 16949607; 16672238; 18579153; 18506900; 18468916 |
| EF_A0005.12 | 4 | 18579153; 10632894; 10998166; 8755555; 9791182; 113798; 2117692; 2128961 |
| EF_A0016 | 11 | 21073510; 15073307; 11955619; 11535031; 11094276; 8820751 |
| FSU0094 | 8 | 21037069; 20884613; 20840841; 20837476; 20577771; 20529855; 20237146; 20158164; 12913005; 8385991 |
| FSU0096 | 8 | 21170890; 21063094; 21037069; 21030403; 20886440; 20884613; 20840841; 20839327; 20838636; 20837476 |
| FSU0097 | 8 | 21037069; 21030403; 20696265; 19741196; 17997985; 19213810; 19176892; 19176220; 18651318; 2025413 |
| FSU01624 | 8 | 21327449 |
| FSU02264 | 8 | 21348887; 21306947; 21302894; 21300551; 21282527; 21192641; 21174947; 21069723; 20952525; 20924850 |
| FSU0236 | 8 | 19548321; 17536929; 16027131; 12745260; 12721855; 11762159; 11523651; 11216846; 11164318; 8381043 |
| FSU0498 | 3 | 10515954; 10627048; 10574995; 10517601; 6266278 |
| FSU0503 | 8 | 21388532; 21387398; 21378116; 21366264; 21365771; 21358763; 21354629; 21354103; 21338575; 21327408 |
| FSU07790 | 4 | 20054112; 19021761; 17561111; 16363530; 15573748; 15489502; 12595266; 10331874; 11524683; 11264598 |
| FSU0909 | 5 | 20952389; 20570913; 15534175; 19738041; 19556632; 19452554; 19022216; 18614746; 18586655; 18473156 |
| FSU11684 | 8 | 20639136; 20522496; 20426480; 19884778; 19507068; 19466498; 19296676; 19205049; 19129644; 18999999 |
| FSU1191 | 15 | 19953494; 16672238; 17785916; 17550218; 17504200; 16006723; 15068623; 12689659; 12037733; 10703921 |
| FSU1222 | 18 | 21350490; 21347707; 21310763; 21299248; 21256461; 21254623; 21223946; 21219664; 21209222; 21209194 |
| FSU1289 | 8 | 15788420; 14993673; 12685039; 10880970; 9266708; 8329680; 6301828 |
| FSU1293 | 15 | 21315771; 21257771; 21050859; 21039781; 20946846; 20942908; 20929957; 20923119; 20884616; 20833806 |
| FSU13062 | 4 | 21366473; 21212283; 21138585; 21067352; 21059684; 20947007; 20934441; 20797970; 20634578; 20610383 |
| FSU1361 | 17 | 20553390; 20453093; 20025658; 19781550; 19682253; 14636572; 16469698; 18786392; 17189297; 17977831 |
| FSU1413 | 3 | 18465758; 16150696; 16141206; 12637499; 9748469; 14212979; 7371957 |
| FSU1441 | 3 | 20735851; 20198307; 18216258; 19179461; 17526849; 17403156; 17172013; 17105195; 14729720; 14645282 |
| FSU14572 | 4 | 20428083; 14713107; 19458369; 19220990; 19061259; 18798201; 18797244; 18718662; 18677293; 18609106 |
| FSU1534 | 5 | 20030377; 3026449; 16202390; 15485884; 11951047; 11084023; 11001089; 10525412; 10231529; 7559591 |
| FSU1566 | 9 | 18167308; 1657935; 17726007; 17675291; 16847310; 16620760; 16243729; 16150824; 9238851; 12524451 |
| FSU1790 | 8 | 20833871; 20498354; 19797693; 19559794; 18631294; 10652612; 17634197; 17522059; 17143338; 16383033 |
| FSU1938 | 18 | 21386888; 21372145; 21318117; 21296199; 21050017; 20979600; 20934432; 20927106; 20919447; 20861221 |
| FSU20704 | 8 | 20562312; 20190048; 19318052; 17172029; 9752722; 9483796; 14907713; 7851752; 8076254; 8360615 |
| FSU2175 | 18 | 21187401; 20363758; 19780839; 19609573; 19576997; 19543924; 19168988; 19130269; 18702072; 17909854 |
| FSU2458 | 3 | 21178073; 20124190; 10361286; 10504382; 15090547; 7655074; 11479281; 11244581; 9266718; 2644215 |
| FSU25284 | 8 | 21253720; 20944403; 20851126; 19544083; 9195887; 10069079; 17947240; 17823855; 271968; 17346264 |
| FSU2661 | 8 | 18651753; 18454933; 16973619; 15021246; 16364665; 10985736; 10982813; 10491142 |
| FSU2662 | 8 | 20979355; 20870790; 18454933; 18370011; 17894548; 11248188; 16973619; 16584177; 15021246; 16303338 |
| FSU2663 | 8 | 20979355; 20870790; 16303338; 9020134; 8797851; 8621464; 7629145; 8063721 |
| FSU2667 | 8 | 20979355; 20816962; 19815558; 19496622; 15972314; 18454933; 19118365; 16608357; 10649489; 17200125 |
| FSU2668 | 8 | 21339825; 20979355; 20870790; 20816962; 20610779; 20558724; 18651753; 20117074; 19815558; 19496622 |
| FSU2669 | 8 | 20979355; 19815558; 16608357; 15943805; 15520003; 15102833; 9718301; 8797851 |
| FSU2670 | 8 | 20979355; 20870790; 15972314; 16608357; 15943805; 15736965; 15520003; 15175326; 15102833; 12740360 |
| FSU2673 | 8 | 20979355; 20870790; 19496621; 16156794; 15175326; 15035646; 11728457; 10491142; 8536688; 1605643 |
| FSU2674 | 8 | 20979355; 20816962; 20610779; 20558724; 20117074; 19815558; 19496622; 19496621; 15972314; 18454933 |
| FSU2783 | 1 | 20651037; 17406446; 15930617; 11551466; 15033357; 10968789; 12356303; 12206759; 11501672; 10968789 |
| FSU2787 | 1 | 18062262; 16921527; 16078071; 1849480; 2824247; 2836080 |
| FSU2922 | 8 | 21365180; 21362400; 21358682; 21327988; 21323512; 21315380; 21297352; 21289039; 21269378; 21257186 |
| FSU3215 | 6 | 20601468; 18606826; 15952900; 19072585; 10890893; 16135232; 18068124; 17981150; 17919660; 17599913 |
| FSU3254 | 3 | 21331046; 21205637; 21330522; 21199899; 21178073; 21147993; 21140375; 21135123; 21129777; 21117450 |
| GGOORF_0589 | 17 | 21367972; 21224389; 21098490; 20505096; 20399182; 8092857; 20103717; 20024606; 11844870; 2579395 |
| GGOORF_0841 | 11 | 15049820; 16133337; 10077450; 1610178; 16348073; 7698675; 8065263; 8121409; 2545526; 2416475 |
| GGOORF_0843 | 11 | 19879290; 17541834; 15049820; 1480115; 11544228; 6826546; 11430408; 11322825; 10411263; 8486246 |
| GGOORF_1019 | 14 | 20693665; 9551557; 15641804; 11223254; 9551557; 8346915; 1447788; 8224889; 2075724; 2673937 |
| GGOORF_1036 | 4 | 21283116; 21244534; 20729351; 20576620; 20534810; 20543140; 20451645; 2002000; 11298291; 19542284 |
| GGOORF_1151 | 11 | 20498309; 10565924; 10515920; 10223973; 1655708; 1851643 |
| GGOORF_1407 | 4 | 12366836; 10829079; 11554792; 10832645; 3309346; 9161424; 1397334; 3039148; 8200538; 1474584 |
| GGOORF_1427 | 18 | 20439155; 9300081; 16122427; 12235121; 12612614; 11168590; 16111939; 9685263; 9325247 |
| GGOORF_1488 | 9 | 20431990; 20204203; 20131019; 20086206; 19797200; 19762802; 19760663; 19473107; 19193980; 18791145 |
| GGOORF_1608 | 1 | 20658341; 20394394; 12953117; 17348838; 10222271; 17098431; 16834327; 15891897; 16767803; 15358538 |
| GGOORF_16414 | 8 | 20412461; 18051354; 9226961; 7925416; 1354172; 3145387; 3106158; 2834081; 6311687 |
| GGOORF_18165 | 2 | 20981023; 20074091; 9254694; 18311129; 17846064; 9235882; 15361144; 8329442; 9326946 |
| GGOORF_2028 | 15 | 21291396; 21282931; 20953742 |
| GGOORF_2094 | 12 | 21307308; 21276854; 21261072; 21107362; 21075846; 21074529; 21071931; 21050005; 20874089; 20833217 |
| GGOORF_2452 | 14 | 16865708; 16544324; 1398079; 8224889; 1245197 |
| GGOORF_26054 | 8 | 20810658; 20618917; 20385610; 19559007; 18555831; 18061466; 18051363; 17623838; 4824506; 17472966 |
| GGOORF_2633 | 15 | 20835487; 20823675; 19836334; 19246238; 18942854; 18509100; 17487983; 17469799; 12390021; 17385895 |
| GGOORF_2864 | 18 | 20953553; 20682717; 20097664; 19788049; 19422811; 18809213; 18670797; 18606230; 10399944; 17489948 |
| GGOORF_2912 | 15 | 21352861; 21349798; 21325639; 21321070; 21319504; 21307321; 21306243; 21306243; 21302810; 21291524 |
| GGOORF_3312 | 9 | 20691134; 20403458; 20086206; 19884765; 19875987; 19850628; 19776640; 18400702; 19690334; 19679157 |
| GGOORF_3361 | 8 | 14658500; 14635721; 12795610; 11063610; 10331270; 9847415; 9571030; 9580189; 8745924; 7578111 |
| GGOORF_3410 | 3 | 20731789; 20035716; 19762447; 19459932; 18390543; 17974560; 17111133; 16907860; 16297878; 15116923 |
| GGOORF_3413 | 8 | 21362632; 21238579; 21187465; 20837989; 20681784; 20545351; 17381106; 20424179; 20373852; 20361926 |
| GGOORF_3659 | 3 | 21215282; 20876123; 20861269; 20808928; 20686034; 20660182; 7645249; 20457214; 11972054; 20392849 |
| GGOORF_39034 | 8 | 21281627; 20367574; 14871805; 12649157; 12393417; 10753889; 12023027; 10574769; 9536084; 1653704 |
| GGOORF_4024 | 8 | 11741948; 10387078; 9037110; 8866660; 7811234; 8468468; 5926184 |
| GGOORF_4332 | 1 | 20057066; 19307721; 19013471; 18269631; 18023379; 17889830; 16327902; 14747737; 11101437; 8875911 |
| GGOORF_5024 | 12 | 20713651; 20599668; 19853572; 1619456; 18387365; 17556688; 17269451; 16834335; 16573693; 16389450 |
| GGOORF_5066 | 6 | 21326933; 21290819; 21213587; 21124229; 20864668; 21059180; 21056083; 21055985; 21055984; 21048111 |
| GGOORF_50954 | 8 | 21394887; 21394610; 21393382; 21391670; 21389003; 21388919; 21388133; 21385604; 21384921; 21382526 |
| GGOORF_5242 | 13 | 21153516; 21151777; 20680338; 20631146; 20608544; 20600071; 20534865; 17041224; 7543677 |
| GGOORF_5777 | 3 | 20855724; 20853818; 20826347; 20385763; 8862584; 20175558; 20154134; 19917674; 17997275; 19854834 |
| GGOORF_5778 | 14 | 21058063; 19574646; 1302001; 17064924; 16026156; 15566465; 14983083; 2185839; 12450384; 11604542 |
| GGOORF_5851 | 15 | 21394810; 21394293; 21394099; 21393445; 21392887; 21392794; 21391228; 21391218; 21389680; 21388974 |
| GGOORF_5939 | 15 | 21315716; 20870410; 20827300; 20439155; 20361936; 20026163; 19712061; 9215735; 19229865; 19100260 |
| GGOORF_6067 | 4 | 20796169; 20718864; 20682460; 20625172; 20228419; 20159424; 20077070; 656521; 19021765; 177845 |
| GGOORF_6339 | 17 | 20413480; 19150431; 11183774; 16936696; 19000817; 19497268; 19409394; 19297693; 12859904; 7518246 |
| GGOORF_6490 | 14 | 21394111; 21304254; 21111048; 21035468; 20363738; 20303634; 20150536; 20101413; 20093117; 20035974 |
| GGOORF_6496 | 12 | 19490017; 19222581; 18171380; 17494995; 11150108; 17098183; 10605091; 16963786; 16808467; 16751091 |
| GGOORF_6962 | 12 | 20805355; 20410297; 20112455; 17194835; 19730897; 10931273; 19505802; 19249287; 17435169; 19152643 |
| GGOORF_7489 | 12 | 19837468; 19689275; 17543192; 10431157; 15175333; 9792533; 1727426 |
| GGOORF_7561 | 15 | 19773805; 20196803; 20026524; 19552622; 19214148; 16847874; 16168628; 15061882; 14623875; 12090300 |
| GGOORF_8897 | 11 | 21147268; 21079776; 21062824; 21035394; 20974949; 20949106; 20929816; 20875907; 20871112; 20869739 |
| GGOORF_8909 | 1 | 16941645; 9365821; 8911642; 8196062; 3413100; 3674395 |
| GGOORF_8952 | 1 | 21267457; 20445230; 20435740; 19568767; 19350404; 19253050; 19157014; 7506898; 17718503; 16141215 |
| GGOORF_9225 | 6 | 20535496; 19706603; 20453833; 20403322; 20096653; 12968183; 16439205; 19723507; 19697864; 15060144 |
| GGOORF_9311 | 3 | 20843801; 20841351; 19883124; 19639961; 16878994; 11500481; 18338572; 15857145; 15552059; 15521563 |
| GKPORF_0215 | 9 | 21120858; 21030508; 20947019; 20725044; 20616867; 20615689; 20555365; 15632194; 20379951; 20230056 |
| GKPORF_0252 | 12 | 21393164; 21390473; 21390281; 21388559; 21387162; 21384401; 21384360; 21383838; 21383693; 21383048 |
| GKPORF_A0019 | 6 | 21143323; 20936510; 20827792; 20435720; 2153220; 11214325; 16923902; 17890221; 11560509; 8300611 |
| GKPORF_B0088 | 11 | 20056615; 19347993; 4598299; 1853558; 11472933; 12999698; 6283090; 17581119; 17428497; 16807240 |
| GKPORF_B02134 | 8 | 21385872; 21367878; 21310505; 21139204; 20970120; 20817800; 20656949; 20632938; 13677464; 18602573 |
| GKPORF_B0227 | 18 | 18456666; 15063852; 10940570; 1438288; 9791180; 8449875; 9442094; 1094454; 8206159; 8411172 |
| GKPORF_B0286 | 18 | 21327044; 21204920; 21069910; 20959450; 20953507; 20947023; 20581826; 20581825; 20515934; 20515644 |
| GKPORF_B0376 | 15 | 20558511; 11244072; 15583165; 10322028; 15263010; 13129942; 10632888; 9179850; 8824586 |
| GKPORF_B0390 | 18 | 12940820; 12939362; 16030141; 18854314; 17055978; 16403413; 11404376; 15533447; 11762197; 11601843 |
| GKPORF_B0544 | 18 | 21264516; 9395087; 10192253; 17395152; 12397032; 16516867; 12906362; 12605306; 11041872 |
| GKPORF_B0567 | 19 | 19364381; 12944424; 11718936; 10493853; 1449602 |
| GKPORF_B0572 | 18 | 16734747; 15456793; 15079005; 11243954; 9299353; 10369265; 9374785 |
| GKPORF_B0591 | 6 | 21187428; 21130077; 21107019; 21067630; 21064183; 20852270; 20822508; 20716951; 20693529; 20675407 |
| GKPORF_B0597 | 6 | 21383164; 21369956; 21343337; 21240275; 21172651; 20952394; 20935048; 20885790; 20817623; 20735784 |
| GKPORF_B0605 | 6 | 322276; 15223318; 2656703; 8385604; 8673463; 1337227; 2540492 |
| GKPORF_B0780 | 15 | 10745001; 15208307; 11851334; 9223636; 7752227 |
| GKPORF_B0810 | 12 | 20570733; 20004668; 18627459; 18485865; 18369105; 18242189; 17378923; 15292133; 16294247; 10856226 |
| GKPORF_B0813 | 3 | 19507071; 17526848; 16275129; 15225319; 10322165; 1683764; 2890081; 3930469; 1676385 |
| GKPORF_B08323 | 8 | 20826164; 20639368; 19839648; 19664062; 10515912; 17373777; 17353140; 16814740; 942051; 12577265 |
| GKPORF_B09627 | 8 | 15353566; 10438748; 12135383; 942051; 7130163 |
| GKPORF_B09667 | 8 | 19580167; 8550433; 19235509; 18331335; 18283541; 17483937; 17223783; 17205041; 15583170; 8132157 |
| GKPORF_B09673 | 8 | 17618087; 16508165; 12410826; 11429466; 7926834 |
| GKPORF_B1019 | 8 | 21166653; 20889786; 20851994; 20632934; 20171064; 19921179; 19395484; 19824993; 19588068; 19506862 |
| GKPORF_B1159 | 4 | 19581367; 16621832; 16423342; 15800931; 12471500; 10504562; 1926332; 8180136; 8443347; 16666276 |
| GKPORF_B1177 | 8 | 19609583; 18281432; 17688437; 17668019; 17139509; 17030441; 11399090; 15882939; 15770479; 15548307 |
| GKPORF_B1186 | 8 | 21106065; 21068384; 20952576; 20807714; 20660112; 20656065; 20624914; 20622059; 20561910; 20429690 |
| GKPORF_B1314 | 6 | 20724389; 20675470; 20310067; 20122408; 19364474; 19154347; 10514571; 7600579; 20076650; 17584298 |
| GKPORF_B14514 | 8 | 19816782; 17944535; 18613689; 18351384; 17221229; 11773048; 12026180; 9891778; 11513090; 11414615 |
| GKPORF_B1640 | 11 | 21073510; 21046326; 19778558; 19479250; 10677279; 17889829; 17725323; 17588178; 10601258; 9867814 |
| GKPORF_B1708 | 15 | 21336656; 20935126; 20022964; 15489417; 10483722; 10339816; 17623030; 17416361; 16803593; 15882409 |
| GKPORF_B1721 | 3 | 21338421; 21209092; 20882995; 20687343; 20089862; 20038538; 19734307; 19594830; 1557269; 10329614 |
| GKPORF_B1748 | 5 | 9514861; 8621656; 8307984; 8504172; 1850088 |
| GKPORF_B1749 | 5 | 19334767; 18314963; 2037604; 15485884; 9514861; 2836706; 2615765; 2643516; 3062178; 2826155 |
| GKPORF_B1770 | 3 | 21367879; 21315310; 21272313; 21250655; 21206755; 21147851; 20958982; 20870880; 20854854; 20843801 |
| GKPORF_B18291 | 8 | 20655316; 19000608; 6684148; 17938906; 12604242; 12009906; 11514550; 393250; 4366025; 5674056 |
| GKPORF_B1863 | 18 | 21330430; 19187221; 10940570; 10696483; 7508433; 8132494 |
| GKPORF_B2168 | 6 | 21394508; 21393174; 21392991; 21390305; 21386025; 21385722; 21385662; 21383994; 21381981; 21381765 |
| GKPORF_B2236 | 13 | 21376540; 21114012; 21078604; 20873482; 20852270; 20823289; 20614151; 19232867; 20000576; 19808234 |
| GKPORF_B2326 | 18 | 20978126; 20825352; 8661505; 20529663; 20513760; 19845409; 18485707; 19640852; 19424690; 19252899 |
| GKPORF_B23681 | 8 | 16930323; 16791965; 16134116; 12511304; 3553176; 6288113 |
| GKPORF_B23721 | 8 | 9973347; 12639950; 11835514; 10203754; 9383150; 7894055; 8397187; 2134185 |
| GKPORF_B2395 | 15 | 11243806; 10600367; 8034728; 8034727; 7690854; 14259761; 2280686; 3104749; 14907713; 13395009 |
| GKPORF_B24034 | 8 | 21301215; 20878180; 20870311; 20659281; 19631314; 1416968; 19453504; 19450903; 19263048; 18769888 |
| GKPORF_B2404 | 18 | 21393220; 21339598; 21339577; 21206039; 21131484; 20880213; 20718490; 19969001; 19720018; 19689316 |
| GKPORF_B2424 | 4 | 21389634; 21389113; 21388661; 21388515; 21384159; 21383121; 21379570; 21378199; 21376104; 21375706 |
| GKPORF_B2447 | 12 | 17612778; 17506485; 17506480; 12367767; 12193063; 11566173; 10690885; 9326397; 9099671; 9108356 |
| GKPORF_B25005 | 2 | 20734996; 11290749; 10829016; 10609891; 9278392; 3994382 |
| GKPORF_B2561 | 8 | 21037291; 20832411; 20724492; 20665426; 20656883; 20228169; 20139187; 20044564; 19782502; 19765784 |
| GKPORF_B2571 | 12 | 21393164; 21392598; 21392585; 21392420; 21391832; 21390473; 21388873; 21386025; 21384516; 21384360 |
| GKPORF_B2636 | 15 | 12386007; 19889099; 1679430; 18048927; 16796685; 16549677; 10216857; 7925310; 12890032; 12657052 |
| GKPORF_B2735 | 4 | 21203349; 21187329; 21173114; 21173113; 21143314; 21142182; 21124864; 21098121; 21179056; 21078677 |
| GKPORF_B2820 | 15 | 21193607; 19352034; 17307850; 15691651; 14523115; 12135487; 10923791; 10825546; 9535079; 8804390 |
| GKPORF_B2911 | 15 | 21386817; 21385762; 21378170; 21378166; 21378021; 21360054; 21346759; 21338645; 21326909; 21326894 |
| GKPORF_B2927 | 3 | 20566764; 9555900; 18503200; 14506006; 16904404; 12787347; 9864321; 15000739; 14686916; 14661974 |
| GKPORF_B2968 | 12 | 21296967; 11919638; 14688122; 16949246; 12900013; 15943900; 9632569; 8830688; 10632892; 10589719 |
| GKPORF_B2982 | 3 | 21288732; 21252495; 21234858; 21182989; 20868513; 20814824; 20651455; 20370816; 20471994; 20308073 |
| GKPORF_B2986 | 17 | 21044566; 21038358; 20831336; 20540151; 20231484; 19751674; 18024596; 6633513; 10806383; 18393864 |
| GKPORF_B3269 | 18 | 21341512; 20952641; 20860031; 20639340; 20534338; 20526342; 17370070; 19623929; 19526856; 18763574 |
| GKPORF_B3319 | 12 | 21348480; 19383877; 18598240; 18539150; 17727464; 16611635; 15793451; 15664096; 15086519; 15048132 |
| GKPORF_B3438 | 15 | 21369825; 21167020; 20637191; 18923073; 18410347; 18027090; 17596930; 17324393; 17116241; 15987823 |
| GKPORF_B3761 | 8 | 19232357; 15211509; 16557313; 1137083; 10666464; 1945499; 8025679; 7947968 |
| GKPORF_B3858 | 5 | 19235722; 19217422; 19154719; 18449193; 18350543; 17296941; 17301780; 16245012; 2199796; 9367878 |
| GKPORF_B3941 | 9 | 21255607; 21184294; 20889740; 20714851; 16430210; 18409384; 17823664; 17201058; 10869041; 15977811 |
| GKPORF_B3944 | 6 | 21394876; 21394715; 21394227; 21390580; 21382701; 21382014; 21381885; 21381205; 21380506; 21373712 |
| GKPORF_B4007 | 15 | 21233158; 21075922; 20924357; 20660485; 20547868; 20473037; 20413502; 20374491; 19150431; 20097859 |
| GKPORF_B4079 | 15 | 21393832; 21383689; 21369825; 21364145; 21355038; 21354180; 21347438; 21339296; 21336990; 21311100 |
| GKPORF_B4080 | 6 | 21394101; 21389547; 21389348; 21389131; 21388532; 21388382; 21386659; 21385925; 21385444; 21385203 |
| GKPORF_B41347 | 8 | 21245529; 21239588; 21072345; 21037291; 20890600; 20400692; 19626321; 19137547; 18847400; 18600042 |
| GKPORF_B4136 | 8 | 20571026; 19862803; 19711960; 19628049; 17881823; 17291766; 1310545; 16917525; 16718678; 16448504 |
| GKPORF_B4259 | 8 | 21375368; 21147773; 20958264; 20935145; 21041650; 20887711; 20843823; 20702580; 20687898; 20684233 |
| GKPORF_B4262 | 15 | 18031348; 15522865; 8899716; 8282725; 8497200; 8432742; 1846145 |
| GKPORF_B42962 | 8 | 20958971; 20656943; 20185797; 20170198; 20357807; 20003708; 19800271; 9620904; 19543272; 19520720 |
| GKPORF_B4381 | 12 | 21383133; 21362190; 21339696; 21329804; 21329797; 21267063; 21252230; 21194113; 21183051; 21170394 |
| GKPORF_B4394 | 4 | 21192796; 11546864; 7783625; 12121970; 10602741; 10411261; 8559061; 6455499; 8089068; 2599989 |
| GKPORF_B4494 | 13 | 20884653; 16839256; 15888129; 8982463; 15186947; 12503083; 11780395; 10101203; 10802150; 10620119 |
| GKPORF_B4538 | 9 | 21194490; 20508139; 18718934; 17464076; 16367956; 15777950; 11911184; 10220166; 1731335; 7588786 |
| GKPORF_B4676 | 15 | 20935102; 20511298; 18346472; 17459874; 15918884; 15210349; 12909015; 11900549; 11563694; 10524758 |
| GKPORF_B4747 | 3 | 21302396; 21279994; 21273450; 21266410; 21261473; 21248167; 21243929; 21241861; 21239738; 21228366 |
| GKPORF_B4797 | 15 | 20937825; 20847442; 20796170; 20723208; 20553947; 20195830; 20149610; 20100284; 20063052; 19995400 |
| GKPORF_B4898 | 3 | 20199591; 20118254; 19850919; 12142488; 18485865; 16573686; 15561151; 8830709; 7909802 |
| GKPORF_B5055 | 18 | 20875141; 20823283; 20691393; 20660570; 20547750; 20522709; 20456012; 20398062; 20345514 |
| GKPORF_B5154 | 6 | 14637246; 18360100; 10713168; 14734554; 11554297; 9743625 |
| GKPORF_B5336 | 13 | 21394482; 21393843; 21381313; 21378135; 21373769; 21371894; 21366817; 21365350; 21359512; 21353613 |
| GKPORF_B5406 | 8 | 21306142; 20976072; 20688982; 20831589; 20847256; 20628895; 20419722; 20417637; 20186410; 20121093 |
| HP0042 | 4 | 20081027; 1807350; 9163424; 3008088; 9663688; 2198250 |
| HP0056 | 8 | 21287990; 21253822; 21168532; 20943826; 20883441; 20545884; 20143043; 19140736; 19954230; 19850488 |
| HP0275 | 6 | 21343909; 21325134; 21306995; 21131491; 21078962; 20811460; 20823236; 20352273; 20232933; 6295393 |
| HP0290 | 1 | 15652176; 12637582; 9559056; 7918636; 7948877; 2082143; 8440471; 1295484; 1368705; 2516490 |
| HP0357 | 8 | 21343423; 21329681; 21185310; 21078123; 21073854; 20931090; 20833539; 20809899; 20806931; 20736169 |
| HP0387 | 6 | 21097613; 15225322; 1667219; 3456159; 3611052; 2547799; 2824502; 3029072; 2991240; 6209275 |
| HP04320 | 4 | 21282572; 21194490; 20942908; 20045694; 7670538; 19850060; 19384577; 18691010; 17512245; 8571127 |
| HP0574 | 8 | 20950777; 20624405; 20578042; 20546899; 20463314; 20439410; 20377641; 20142768; 20027484; 19943855 |
| HP0584 | 4 | 21223978; 21165487; 20935096; 20693676; 20676082; 20586476; 16487743; 20543140; 20444091; 20439729 |
| HP0630 | 4 | 21391271; 21389767; 21371011; 21366724; 21347269; 21319882; 21309927; 21262970; 21256460; 21248261 |
| HP0653 | 18 | 16009582; 10465560; 15508404; 9460808; 8897897; 3100721 |
| HP0683 | 14 | 12465928; 19904366; 19804974; 1815783; 18996617; 18765909; 11278591; 15037198; 11118200 |
| HP0735 | 14 | 21323897; 20544508; 19390618; 7537850; 18720848; 18536021; 17039332; 16897745; 16881638; 16613999 |
| HP07792 | 8 | 21269378; 20933603; 20929953; 9231420; 18491315; 20511233; 15347752; 20051483; 20007518; 19943190 |
| HP0835 | 13 | 20889748; 20824060; 20576476; 17005013; 20497998; 20484776; 20375021; 20010798; 19995727; 19777785 |
| HP08431 | 2 | 17174261; 1141859; 9700068; 9252577; 7982968; 2542220; 791939; 14217462; 1097404 |
| HP0855 | 3 | 10781617; 1663570; 9457868; 8550474; 2838462 |
| HP0877 | 6 | 21172651; 20952394; 20735784; 7673186; 20634321; 20601468; 20512659; 20304994; 20298191; 20203129 |
| HP0887 | 4 | 21341154; 21083636; 20927590; 20684964; 20682750; 20633190; 20615415; 20568532; 20512250 |
| HP0907 | 4 | 20639318; 10785634; 20154133; 20132451; 19383688; 19332819; 18931786; 18811728; 18635679; 18483484 |
| HP0925 | 6 | 21325134; 21197557; 21166902; 21145792; 21145459; 21085632; 21048951; 21037079; 21035407; 20935631 |
| HP0950 | 9 | 21294903; 20923423; 20725044; 20690600; 20616867; 20129920; 1355089; 19926919; 10744768; 18992226 |
| HP0961 | 8 | 21126600; 20824484; 20724705; 19815826; 17360190; 18503541; 8906967; 12584194; 12758080; 12604241 |
| HP0978 | 4 | 21335646; 21216997; 19680248; 12519187; 17326815; 20345660; 20132438; 19737354; 16146521; 8181761 |
| HP1052 | 3 | 20019290; 17983217; 12324252; 17176046; 1503445; 10026271; 15705580; 15667204; 12000770; 11237337 |
| HP1134 | 8 | 21345803; 21326874; 21193405; 21106936; 20880213; 20626349; 20154086; 20056103; 20026007; 8026496 |
| HP1138 | 6 | 21185326; 20581207; 1648208; 11055941; 16494844; 16122562; 10220448; 15737404; 15528650; 9199437 |
| HP1155 | 3 | 21370307; 21244065; 21183069; 21183069; 21167155; 21129777; 21097619; 21094461; 21068409; 21048936 |
| HP1375 | 3 | 20564049; 20433200; 20400541; 19604481; 3277952; 15572779; 18675810; 9829962; 11118459; 17434525 |
| HP1386 | 8 | 21385868; 20923965; 17979299; 9694668; 16489742; 16304640; 15333955; 15240097; 15109729; 12547196 |
| HP1427 | 18 | 21194620; 20049887; 19828445; 19786305; 19714712; 19447499; 19399921; 16780604; 18698560; 18565588 |
| HP1543 | 3 | 15946665; 9915498; 10225872; 7523254; 7523253; 1482126; 2000374; 3278319 |
| HP1558 | 4 | 15001355; 11554792; 6394719; 10049798; 8757287; 8107139; 1474584; 1404383; 1370543; 1905667 |
| HP1574 | 2 | 19622649; 11399071; 7473709; 7622491; 7814407; 8055941; 2115523; 2106516; 2506544; 3502256 |
| KVP40_0350 | 11 | 21385768; 21337578; 21293970; 21267078; 21248763; 21185934; 21149573; 21144870; 20951708; 20817832 |
| KVP40_0356 | 11 | 19352408; 15158724; 12600265; 10704310; 10544047; 9668211; 2143080; 2143079; 2963141; 3878456 |
| MCA_00330 | 4 | 21123297; 20739400; 1861990; 20633226; 20511593; 1899477; 19218986; 19217408; 19134553; 7997877 |
| MCA_0204 | 5 | 21231969; 20219465; 16756317; 10506203; 17959596; 17163967; 17154541; 16137685; 15728375; 10217770 |
| MCA_0777 | 15 | 20952573; 20942908; 2265755; 20507519; 10633114; 12823806; 10806366; 13376513; 8780507; 319344 |
| MCA_0823 | 11 | 20067338; 16181782; 18375798; 17367389; 17078817; 7588618; 16731525; 10760133; 11822682; 11352577 |
| MCA_1389 | 8 | 11722563; 9756865; 2985470; 7746153; 1657982 |
| MCA_1423 | 3 | 21278296; 3611062; 20194103; 19825675; 17167475; 18499663; 18428041; 18062236; 17405771; 17234634 |
| MCA_1467 | 12 | 21036998; 20679510; 2965141; 18174130; 10792719; 17074897; 9159522; 8412698; 16376476; 11761711 |
| MCA_2625 | 12 | 20888319; 19809197; 18617521; 10564520; 17509078; 17498646; 17038124; 10564520; 12823819; 12297303 |
| MCA_2711 | 9 | 21358752; 21281405; 21272644; 21268991; 21247177; 21161791; 21161790; 21147774; 21146894; 21142044 |
| MCA_3061 | 2 | 19267692; 15522295; 14595395; 11215515; 9689094; 1856165; 1732195; 8953249; 1349019; 7606163 |
| NT01AA0180 | 8 | 21386889; 21383483; 21372790; 21370438; 21346890; 21330297; 21315900; 21306405; 21277649; 21271486 |
| NT01AA0584 | 13 | 20521084; 20352288; 18310410; 18234219; 18565340; 16914449; 8501042; 16859496; 10214965; 15547251 |
| NT01AA0684 | 14 | 16026156; 727478; 7506026; 13267987; 183204; 2687276; 2592365; 3301838; 7066348 |
| NT01AA0778 | 1 | 20184895; 17347518; 19105697; 17651682; 17506328; 4630504; 12718933; 10692366; 16218965; 16207702 |
| NT01AA0871 | 18 | 21135102; 19158428; 18941142; 18248418; 11050157; 17489102; 8932698; 16164825; 15808935; 15709779 |
| NT01AA0958 | 9 | 21389045; 21386062; 21376757; 21375498; 21368116; 21344388; 21342606; 21342605; 21327327; 21324704 |
| NT01AA1045 | 8 | 21303200; 21251101; 21112414; 20971673; 20875067; 20808932; 20643099; 20636270; 20627642; 20552226 |
| NT01AA1259 | 8 | 21193741; 21099326; 10783895; 8662184; 17336263; 18455502; 17904249; 17506728; 17413318; 16545948 |
| NT01AA1447 | 3 | 19269582; 17944363; 17874321; 16579470; 16310730; 14674749; 11267762; 1672263; 6247326; 3920477 |
| NT01AA1522 | 8 | 19916927; 17089214; 15173414; 12031252; 10666639; 10328337; 2162313; 3524849; 3896810 |
| NT01AA1539 | 17 | 21391582; 21387067; 21358283; 21350489; 21346783; 21329667; 21327085; 21326884; 21326359; 21321083 |
| NT01AA1679 | 2 | 21075928; 20814239; 20674542; 20535114; 20518024; 11079541; 20407804; 20363753; 20340124; 20194361 |
| NT01AD01474 | 8 | 21392539; 21389981; 21386062; 21385868; 21376063; 21372039; 21364661; 21349219; 21346776; 21343617 |
| NT01AD0251 | 12 | 21385872; 21383693; 21368222; 21365746; 21333600; 21330347; 21316440; 21296596; 21279997; 21277892 |
| NT01AD0739 | 12 | 21278755; 21241507; 21187898; 21112241; 21073315; 21037004; 20981744; 20977272; 20973967 |
| NT01AD1213 | 8 | 20152818; 19646264; 17302961; 17235181; 16631167; 10352308; 15149576; 12738250; 11350974; 11170752 |
| NT01AD1220 | 3 | 2580220; 19210675; 18266468; 15322031; 15014080; 18755141; 18086187; 15866924; 16099047; 2908643 |
| NT01AD1230 | 8 | 20952576; 20335176; 19659724; 18454519; 11334784; 18353797; 18338855; 17927903; 8962083; 17143652 |
| NT01AD1234 | 5 | 20118248; 17468253; 9622362; 17586631; 19132060; 12634335; 17113269; 15664919; 16426819; 16273615 |
| NT01AD1265 | 8 | 20193659; 11029691; 16202924; 12822929; 12614899; 12351620; 10940012; 11058595; 9802017; 7657670 |
| NT01AD1431 | 4 | 8071222; 15136044; 11327763; 1474584; 1551848; 1905667; 2193164; 2181149; 13770074; 2129540 |
| NT01AD1502 | 2 | 20054111; 18716757; 18704520; 16407262; 18298089; 17130127; 17442677; 8427805; 16042411; 15065880 |
| NT01AD1549 | 6 | 16009137; 16473661; 8556862; 10476039; 9657933; 2555940; 8065255; 3894006 |
| NT01AD1850 | 15 | 21393847; 21362064; 21332623; 21245528; 21175741; 21173183; 21075931; 21068394; 20971902; 20952573 |
| NT01AD2064 | 3 | 21385868; 21384454; 21346818; 21337322; 21335527; 21320570; 21307865; 21267505; 21256827 |
| NT01AD2065 | 1 | 21040473; 20924576; 21080591; 20832292; 20800052; 20736170; 5420057; 20385762; 20118241; 19066616 |
| NT01AD2141 | 8 | 20880984; 20860663; 20811295; 20724486; 20659230; 20634390; 20610532; 20601070; 20484870; 20444882 |
| NT01AD2210 | 6 | 21388532; 20622008; 20526281; 20026132; 19618961; 19343651; 19199647; 16424906; 18614530; 11390668 |
| NT01AD2402 | 4 | 21393912; 21393831; 21393450; 21393226; 21393224; 21393220; 21393211; 21393206; 21393204; 21393194 |
| NT01AD3000 | 8 | 21238579; 21187465; 21037009; 20942797; 20837989; 20802042; 20681784; 20655588; 20595031; 20545351 |
| NT01AD3099 | 12 | 17592886; 15078583; 12923392; 11461752; 11299728; 6638110; 10067796; 9709027; 1597429; 9641687 |
| NT01AD3181 | 8 | 15987803; 20433942; 20403753; 3982017; 20110695; 19119913; 18694756; 18498255; 18062777; 17904517 |
| NT01AD3467 | 8 | 1671405; 15340917; 15134648; 12787152; 12646371; 12137805; 11053423; 10498693; 9551550; 2518709 |
| NT01AT00301 | 8 | 9593306; 10557274; 15916599; 10426948; 1943760 |
| NT01AT1141 | 15 | 14512618; 10557274; 15339663; 14527278; 10426948; 12622822; 2536661; 10557274; 10619016; 1438215 |
| NT01AT1698 | 15 | 20525830; 20351295; 12824468; 9830034; 9593306; 17136100; 10199407; 14527278; 12603734; 10199407 |
| NT01AT1819 | 8 | 21107318; 20933183; 20456655; 20124719; 20089767; 19860829; 19084582; 18581728; 18549703; 17993624 |
| NT01AT1912 | 8 | 20952576; 11334784; 18353797; 8962083; 17143652; 16820758; 16211847; 12126468; 11864982; 9880817 |
| NT01AT2812 | 8 | 19888818; 9526510; 17129407; 11733507; 11181712; 10866828; 10712605; 10437825; 9565548; 9426597 |
| NT01ATA0195 | 4 | 21389006; 21314460; 21253945; 21240913; 21221750; 21216027; 21182595; 21167031; 21143936; 21094695 |
| NT01ATA1304 | 8 | 20514546; 17977854; 10952004; 10390816; 9692922; 9642221; 13566053 |
| NT01BF0158 | 18 | 19425588; 1715858; 19166984; 12107133; 14714867; 10092468; 12791142; 11104814 |
| NT01BF02294 | 8 | 21050758; 20381814; 19227841; 19145281; 20578419; 19554539; 18000065; 19031451; 17396135; 2409240 |
| NT01BF0370 | 4 | 20097452; 20096385; 12951241; 18165184; 15974590; 15492014; 14572387; 12007843; 1513876; 16887510 |
| NT01BF0377 | 4 | 21393220; 21378297; 21353964; 21335387; 21324723; 21320350; 21299838; 21291846; 21271477; 21268690 |
| NT01BF0526 | 14 | 20946652; 20601304; 19515998; 18838482; 18600548; 18538515; 18511695; 18445485; 1682344; 18256308 |
| NT01BF0703 | 4 | 21393212; 21393173; 21389261; 21382175; 21381897; 21372393; 21350795; 21348461; 21343459; 21341745 |
| NT01BF0767 | 3 | 21115895; 21106106; 21057009; 21054786; 20944090; 20854854; 20832020; 20817725; 20731661; 20727406 |
| NT01BF0772 | 6 | 20639326; 17005013; 19063900; 17890313; 10096873; 10514438; 16941243; 16257976; 15998541; 15703849 |
| NT01BF0814 | 18 | 19911131; 16286358; 1008746; 9720051; 9133319; 3896791; 2674131; 14907713 |
| NT01BF0820 | 8 | 20739305; 19880397; 18811617; 14675429; 17206375; 17050641; 15474032; 10806243; 12244450; 16668996 |
| NT01BF0822 | 5 | 20622237; 20232353; 19650410; 17998446; 16990043; 10677367; 15456941; 12932876; 12624138; 12573255 |
| NT01BF0907 | 12 | 21319597; 20159304; 21226293; 21174780; 20660625; 21097155; 21089124; 21068237; 21038299; 21033077 |
| NT01BF1008 | 5 | 16061947; 18094529; 17249214; 17080200; 16623713; 15733089; 12461688; 10334395; 10814710; 10682309 |
| NT01BF1086 | 6 | 21347417; 21347256; 21291520; 21048863; 21030504; 20921460; 20875401; 7607479; 18535086; 19474347 |
| NT01BF1302 | 8 | 21335525; 7923353; 15066170; 15005627; 9894011; 2917572 |
| NT01BF15581 | 8 | 17616597; 12081954; 10852890; 11094294; 3036772; 11532130; 10511399; 10381095; 1615064; 2837147 |
| NT01BF1564 | 6 | 20599730; 20547849; 20054126; 7891691; 19425495; 19378188 |
| NT01BF1579 | 3 | 18506900; 16233119; 3927821; 12526316; 11169113; 9765842 |
| NT01BF1601 | 4 | 21391297; 21390252; 21390146; 21390130; 21390126; 21389676; 21389279; 21387154; 21383206; 21382474 |
| NT01BF16214 | 8 | 19420989; 19224400; 12964628; 10500139; 10091328; 9933916; 6277922; 1777121; 1900250; 14775715 |
| NT01BF1948 | 18 | 21377460; 21209359; 20979388; 17302934; 20684239; 20111865; 20103563; 20028819; 19828274; 19703346 |
| NT01BF1984 | 3 | 20514382; 18506900; 16690914; 3927821; 1314450; 2282142; 3739227 |
| NT01BF2016 | 15 | 20455946; 19894045; 17639608; 18495502; 16221580; 16205910; 15455157; 10200952; 12581638; 9466267 |
| NT01BF2317 | 8 | 21377964; 21370391; 21368147; 21366233; 21359198; 21354629; 21354350; 21351540; 21345803; 21335453 |
| NT01BF2318 | 8 | 21370391; 21368147; 21359198; 21335453; 21279668; 21273631; 21209382; 21177473; 21175620; 21040791 |
| NT01BF26182 | 4 | 21148448; 20614900; 20531424; 15172782; 11076687; 10216867; 8135545; 2930783 |
| NT01BF2679 | 8 | 20599628; 2156866; 18619553; 17186219; 17046099; 16204727; 15703124 |
| NT01BF26901 | 2 | 21367973; 21268892; 21148731; 20932820; 20873853; 20844120; 20667822; 20648783; 20644337; 20473558 |
| NT01BF26971 | 2 | 1141859; 12393194; 7982968; 2542220; 791939; 14217462; 1097404 |
| NT01BF2835 | 8 | 19734178; 19584547; 19254926; 18582433; 11891220; 17305364; 17176101; 16299377; 15475358; 15013751 |
| NT01BF2839 | 1 | 20698752; 8905078; 12842015; 19200761; 18844488; 18620331; 10226042; 17925002; 17917419; 10931901 |
| NT01BF2875 | 13 | 20630999; 20403960; 7954835; 16914449; 16924465; 15485852; 15048082; 12760248; 9456322; 10833194 |
| NT01BF28924 | 8 | 21262610; 21183032; 21104367; 21077101; 21035754; 20960558; 20876143; 20841354; 20811656; 20716056 |
| NT01BF2982 | 14 | 18821049; 16349109; 10708364; 11857678; 11605013; 11519722; 10817902; 10692162; 10572963; 8969503 |
| NT01BF3047 | 18 | 21394896; 21394428; 21393109; 21392823; 21392187; 21392144; 21391781; 21389980; 21389771; 21389184 |
| NT01BF3082 | 8 | 20633346; 20399532; 19747091; 19723108; 18340447; 1621979; 17943178; 17559573; 17442734; 17294332 |
| NT01BF3388 | 18 | 20709757; 20659170; 20578725; 20573049; 20487273; 20067714; 20047987; 19819871; 19460820; 19416103 |
| NT01BF3457 | 12 | 19375145; 18550518; 18163885; 9680220; 8885277 |
| NT01BF3581 | 18 | 20981744; 20373322; 19772851; 19678554 |
| NT01BF36487 | 8 | 20949348; 19228626; 19807880; 19778366; 19597319; 19523599; 16565040; 6998727; 19141113; 19091740 |
| NT01BF3731 | 18 | 20855745; 20573661; 2017436; 20419429; 18039771; 9564569; 19373193; 19062291; 18754755; 18644451 |
| NT01BF3793 | 6 | 20441787; 15358184; 12547203; 11106437; 10049831 |
| NT01BF3986 | 4 | 20605227; 20336003; 20172962; 19819015; 19715333; 19444935; 16061947; 18821018; 18723489; 18493130 |
| NT01BF3987 | 18 | 21251309; 21030685; 20582614; 20332769; 19394256; 18989752; 18792995; 18584975; 18056156; 17876718 |
| NT01BF4052 | 6 | 20811621; 20737479; 20724832; 20457142; 20392944; 20181795; 11477108; 20045502; 20040383; 19883731 |
| NT01BF4152 | 12 | 21317884; 21304520; 21288877; 21266548; 21237243; 21203472; 20886084; 20876123; 20731850; 20696164 |
| NT01BFA0024 | 4 | 20403380; 402693; 1740662; 18176015; 17381401; 15662369; 17065365; 10446041; 16754725; 16569707 |
| NT01BL0091 | 8 | 20566639; 20392694; 2733387; 19473252; 19424622; 19296828; 17452333; 17956306; 10719238; 17430888 |
| NT01BL0175 | 6 | 20724226; 16877383; 18616603; 12404116; 14592985; 17516097; 17416902; 17042786; 9406544; 16428325 |
| NT01BL0243 | 11 | 21350632; 21310067; 21262282; 21175897; 21138556; 21115395; 20508639; 20055590; 20007331; 11932238 |
| NT01BL0272 | 3 | 18672909; 10322023; 16618123; 15752194; 12760681; 11055929; 11832520; 11700352; 9720026; 9387226 |
| NT01BL0384 | 12 | 21080956; 9529072; 12905473; 10725905; 10381609; 1540031; 1702904 |
| NT01BL0481 | 12 | 21199704; 20732470; 20540439; 20232546; 20188677; 19803404; 18542876; 11159421; 18843846; 2506916 |
| NT01BL0535 | 1 | 21348811; 21239826; 21218453; 21190711; 21185872; 21176956; 21167847; 21089167; 21089122; 21055431 |
| NT01BL0548 | 12 | 12498799; 1807350; 12890031; 10377148; 11572456; 11407914; 10716714; 10366500; 10066461; 8265585 |
| NT01BL0949 | 15 | 21315771; 21216906; 21193607; 21173175; 21030435; 20946847; 20923119; 20453099; 20404199; 20378989 |
| NT01BL1022 | 6 | 20975945; 20448041; 20022231; 11090626; 19266338; 18641947; 16981199; 7846053; 15758242; 15581892 |
| NT01BL1096 | 18 | 21084222; 20709841; 19801641; 17558532; 17074913; 10829230; 16872761; 10850805; 16547355; 16514154 |
| NT01BL1163 | 14 | 21301108; 21058063; 21036757; 20082641; 19574646; 1302001; 19394344; 11111049; 17902044; 17896107 |
| NT01BL1167 | 9 | 21393852; 21393244; 21392972; 21392566; 21392564; 21391204; 21389144; 21388805; 21388804; 21387258 |
| NT01BL11994 | 8 | 20971079; 20562284; 20333513; 18572963; 17955189; 17689155; 16831850; 16798843; 7248307; 15292273 |
| NT01BL1431 | 18 | 21393174; 21389634; 21372393; 21366542; 21362586; 21360409; 21351087; 21349151; 21347827; 21345797 |
| NT01BL1539 | 12 | 21284297; 20960204; 20659898; 20597985; 15901697; 17803780; 17188820; 17173634; 16729579; 16493696 |
| NT01BL1547 | 11 | 20846564; 20621204; 20425563; 19723611; 17114059; 19561090; 19460312; 19447461; 19197790; 16689791 |
| NT01BL1699 | 13 | 21153748; 21097889; 20832751; 20696253; 20378560; 9331260; 20554519; 20478248; 20675743; 20087403 |
| NT01BL1761 | 12 | 17382507; 17140137; 15972652; 11807068; 15680210; 15654431; 14978332; 1774244; 11158345; 10352280 |
| NT01BL1861 | 8 | 21354971; 21345800; 21159850; 21238579; 21187465; 21176082; 21252179; 21135141; 21099366; 21084197 |
| NT01BL1910 | 14 | 21228488; 21210713; 21124792; 21072045; 21058063; 20933180; 20884265; 20857210; 15034147; 20675450 |
| NT01BS00392 | 4 | 15214909; 7673233; 7665601; 7758088; 7641213; 7947212; 8229126; 8337754; 7504187; 8294029 |
| NT01BS0051 | 15 | 20100285; 18955155; 17660417; 12603732; 12224521; 12123659; 11972779; 2447063; 10411754; 10589719 |
| NT01BS02194 | 8 | 21388249; 21380805; 21376774; 21354026; 21349584; 21334356; 21331654; 21322956; 21321400; 21318391 |
| NT01BS0278 | 8 | 21376009; 21362401; 21348511; 21341514; 21334702; 21329667; 21322494; 21319715; 21319712; 21313758 |
| NT01BS0300 | 1 | 21114891; 21048403; 20861582; 20853870; 20853735; 20797482; 20724492; 20664391; 20662904; 20621770 |
| NT01BS0343 | 4 | 21378181; 20013255; 19719228; 19332816; 18779054; 3080407; 16005290; 12224521; 16219920; 18025597 |
| NT01BS0381 | 4 | 21375589; 21357464; 21350246; 21321189; 21270153; 21184741; 21087599; 20881037; 20717870; 20515657 |
| NT01BS03937 | 8 | 21376550; 21313829; 21309481; 21307852; 21302394; 21287782; 21284261; 21284260; 21270273; 21261654 |
| NT01BS0720 | 6 | 21359091; 21187418; 21165603; 21123375; 21098111; 21083801; 21070745; 21062436; 21037296; 20972394 |
| NT01BS07331 | 8 | 21899333; 21879968; 21790798; 21749217; 9923704; 21700510; 21681759; 21600397; 21600277; 21548553 |
| NT01BS0792 | 18 | 21059706; 20551214; 19911130; 19267410; 18397761; 16961923; 17462011; 17352426; 16925552; 10471562 |
| NT01BS0839 | 18 | 21252218; 20870766; 10329614; 12163465; 16221579; 15774702; 15112987; 14644552; 10966480; 12110531 |
| NT01BS10050 | 4 | 21317325; 14570271; 8561462; 9540829; 9504989; 3440704; 2083842; 2599354; 3112127; 3087950 |
| NT01BS1034 | 4 | 20709900; 20109146; 7934817; 19836336; 19625340; 10986257; 17001104; 16469691; 11328872; 12627868 |
| NT01BS1145 | 2 | 19919179; 17178720; 16606627; 15967800; 11578923; 12975365; 9767567; 4345352; 9396835; 14907713 |
| NT01BS1231 | 6 | 16011798; 18753784; 10465774; 17666433; 16887145; 10362555; 10078206; 11178902; 10878253; 10669596 |
| NT01BS1334 | 12 | 21378193; 21088825; 8102773; 16249335; 17908206; 17379715; 10320662 |
| NT01BS1412 | 8 | 21349323; 21328910; 21180368; 21125383; 21047134; 20933082; 20857167; 20854909; 20489034; 20471192 |
| NT01BS1438 | 15 | 21118702; 20133180; 19675387; 17617222; 17185552; 17003273; 17001075; 19712292; 14636076; 12875849 |
| NT01BS1441 | 3 | 21208281; 21149452; 21097580; 21073699; 20980996; 20923420; 20847047; 20844865; 20795394; 20738399 |
| NT01BS1453 | 15 | 21394097; 21394064; 21393232; 21390238; 21390154; 21389260; 21389231; 21385355; 21384063; 21381918 |
| NT01BS16181 | 8 | 11953318; 16140251; 15210713; 10610766; 15090729; 14527945; 12966919; 12778888; 11740154; 10974121 |
| NT01BS1629 | 15 | 21389112; 21306447; 21173162; 21050859; 20980623; 20966074; 20946841; 20922376; 20839945; 20817765 |
| NT01BS1683 | 15 | 21050859; 20839945; 20524093; 20502894; 20391342; 20298190; 19628563; 19924481; 6550579; 19580872 |
| NT01BS1772 | 4 | 5160720; 2408275; 7585939; 8320224; 3133358 |
| NT01BS1910 | 13 | 21126361; 21088776; 21035469; 20975056; 20943505; 20937141; 20876715; 20862326; 20809949; 20736952 |
| NT01BS2078 | 8 | 21394887; 21394573; 21394485; 21394452; 21394360; 21394328; 21394221; 21394091; 21393892; 21393734 |
| NT01BS20804 | 8 | 21266107; 21234787; 21206049; 21058068; 20724279; 20703806; 20621155; 20578650; 20462210; 20461586 |
| NT01BS2137 | 4 | 21341216; 21287229; 21253720; 21187330; 20962906; 20720380; 20627133; 20478643; 20352975; 20067152 |
| NT01BS2172 | 6 | 21242293; 21187070; 21135142; 20947453; 20876584; 20868484; 20662484; 20622147; 20562439; 7957102 |
| NT01BS22637 | 8 | 12619701; 11052676; 11052675; 10561613; 9914491; 9851033; 11902724; 9208947; 9128730; 8664258 |
| NT01BS2482 | 4 | 9384377; 16907802; 17381738; 2497051; 1527010 |
| NT01BS2582 | 4 | 21315983; 21076486; 20873796; 20729357; 20435722; 4212901; 19891996; 19914579; 19332816; 19302310 |
| NT01BS2600 | 17 | 18070067; 9683469; 14993308; 9890793; 7545758; 10937434; 9393439; 9308178; 2691330 |
| NT01BS2604 | 8 | 20050916; 19682263; 16825793; 10844653; 10386372; 9395519 |
| NT01BS2768 | 4 | 11178251; 1973824; 8825779; 1944223; 3731272; 4966830 |
| NT01BS2859 | 18 | 21394934; 21390308; 21384470; 21383179; 21377497; 21377491; 21377439; 21375801; 21374001; 21373257 |
| NT01BS2864 | 4 | 10099385; 11115120; 10447890; 9566192; 9427409; 9157240; 3158742 |
| NT01BS2895 | 3 | 8626066; 7908269; 7907356; 7902527; 1355454 |
| NT01BS3085 | 9 | 21304157; 20667918; 20603333; 20591207; 20546038; 20471473; 20171262; 20146267; 20036247; 20009345 |
| NT01BS3139 | 2 | 18680949; 10217486; 9163953; 8012594; 16590310; 3040684 |
| NT01BS34877 | 8 | 21166655; 20195658; 19754880; 16858718; 16611640; 1628166; 10480865 |
| NT01BS3526 | 15 | 21050859; 20473954; 20444088; 20391342; 19924481; 19775247; 18943197; 18410285; 18216168; 18190531 |
| NT01BS3625 | 15 | 21254160; 21176126; 21151122; 21149702; 21136601; 21135865; 21102458; 21081698; 21078983; 21075852 |
| NT01BS36335 | 8 | 16293794; 11092937; 8537361; 7803457; 7815950; 1324836; 1657982 |
| NT01BS3802 | 15 | 21375718; 12800502; 15720552; 17293407; 18292804; 17177879; 16579459; 12850135; 10094627; 9889978 |
| NT01BS3804 | 15 | 20883732; 11707303; 17183215; 18092479; 10679470; 16933036; 9922236; 10742272; 14522943; 12596232 |
| NT01BS3908 | 5 | 21389671; 21367444; 21366328; 21354154; 21330668; 21295415; 21292767; 21289427; 21268838; 21251930 |
| NT01BS3940 | 8 | 21329369; 21222470; 20873742; 20630646; 20226371; 19904973; 19715303; 19697907; 19627115; 19294434 |
| NT01BS3964 | 15 | 19836340; 19668863; 18599839; 7730271; 18051269; 17981109; 17981123; 12408815; 17590234; 15299016 |
| NT01BS3976 | 3 | 17298895; 11375403; 6150066; 7581999; 8093697; 2111808; 8093697; 271968; 1355454; 1356138 |
| NT01BS4001 | 18 | 20693325; 18524919; 18359531; 16407393; 6031495; 15287594; 10835424; 12514248; 12508063; 11408491 |
| NT01BS4048 | 15 | 21349825; 20674516; 19833762; 17121866; 18258916; 10653770; 7958867; 10721692; 18487951; 18422602 |
| NT01BS4061 | 4 | 20979349; 20199595; 20132451; 18931786; 18811728; 18483484; 10995478; 2181149; 15699192; 14960570 |
| NT01BS40770 | 4 | 12813085; 18603115; 8464402; 8299942; 10588743; 15217342; 11108953; 1943780; 119663; 1527185 |
| NT01BS4391 | 15 | 16932909; 12903241; 6364042; 3034860; 14104; 6128288 |
| NT01BS4402 | 18 | 21357424; 21309115; 21289302; 21173226; 21148490; 21148012; 21135361; 21127383; 21110306; 21052943 |
| NT01BS4434 | 15 | 19833776; 18310071; 10742046; 15183876; 10498721; 11274109; 10322165; 9887260; 2040302 |
| NT01BS44567 | 8 | 20460727; 20419500; 18375516; 19521631; 19240925; 19069847; 18950192; 18681865; 18561187; 18336351 |
| NT01CD0060 | 12 | 21385720; 21288886; 21257602; 21212608; 21190527; 21187463; 21182594; 21145937; 21135131 |
| NT01CD0136 | 6 | 20873830; 20840885; 20805875; 20701268; 20547483; 20347426; 20201599; 21364794; 19883114; 19864471 |
| NT01CD0259 | 13 | 20617848; 20606262; 20601684; 20457752; 18957446; 17533454; 19481543; 18252769; 19106621; 18817520 |
| NT01CD06885 | 8 | 16099182; 19099776; 12949377; 15059621; 12409268; 12007220; 9683601; 10329019; 9683657; 9385377 |
| NT01CD0793 | 11 | 21350632; 21224843; 21165667; 21156198; 21138556; 21115395; 21110982; 21110848; 21073510; 21046326 |
| NT01CD08215 | 2 | 10737928; 12646322; 12486521; 12435492; 12009883; 11917149; 11563824; 10666297; 11399090; 11200224 |
| NT01CD0856 | 1 | 21243152; 20956302; 20944228; 20944209; 20941392; 20690656; 20640801; 20558569; 20433850; 20426479 |
| NT01CD1002 | 18 | 18502865; 18724706; 7608087; 15268940; 4908543; 14614536; 12897968; 10390542; 11278508; 7904655 |
| NT01CD1128 | 18 | 20111865; 10377096; 18248412; 9590298; 15451112; 11136469; 10094405; 2914875; 8558445; 1459958 |
| NT01CD1260 | 12 | 21205202; 20000742; 19661058; 17038124; 15513925; 6259126; 7651187; 8051048; 8344936; 2033085 |
| NT01CD1319 | 13 | 19900465; 17868038; 17103135; 15748981; 11395405; 10387030; 8548458; 7559506; 2982857 |
| NT01CD1421 | 4 | 21378183; 20423905; 20008079; 8998998; 19453507; 19217259; 19169435; 17943138; 12712204; 17280489 |
| NT01CD1431 | 3 | 20831907; 20369853; 19900400; 19694481; 18672895; 10775458; 18177480; 18045236; 17723996; 17900548 |
| NT01CD1444 | 5 | 20379711; 19845287; 15668011; 15168860; 10913707; 10448674; 1785931; 8703950; 8639629; 8617763 |
| NT01CD1451 | 6 | 21394607; 21394480; 21393214; 21393190; 21393159; 21392927; 21392918; 21392048; 21392020; 21391286 |
| NT01CD1576 | 18 | 20858707; 20228341; 20132518; 20112288; 20074989; 19781010; 11679669; 19405101; 17072531 |
| NT01CD17280 | 8 | 21236318; 21087210; 20655923; 20160956; 20160912; 20106967; 19996100; 19801660; 17532339; 18641270 |
| NT01CD1989 | 12 | 21147144; 20726765; 20600818; 20435890; 20347820; 20213046; 6782899; 19967151; 19953344; 19917683 |
| NT01CD2014 | 12 | 18048681; 16253240; 16212962; 16091426; 15135061; 15122315; 12879215; 12150997; 10570802; 9371463 |
| NT01CD24151 | 8 | 21209328; 21077103; 20379761; 20198665; 20158392; 20154114; 20149645; 16350018; 19761777; 19715675 |
| NT01CR0022 | 12 | 20005877; 17554808; 17483319; 16451085; 16410343; 16260779; 14599350; 11943775; 7505615; 11735566 |
| NT01CR0487 | 1 | 18855421; 11751050; 10673432; 9722552; 9562556 |
| NT01CR0804 | 4 | 19809807; 17766410; 10322040; 15165230; 7049234 |
| NT01CR1098 | 18 | 19373193; 19361527; 18644451; 11120952; 11104814; 10655415; 12526851; 11709306; 12351840; 10203840 |
| NT01CS0009 | 3 | 18227251; 3038334; 1834913; 2985566; 776981; 6330111; 6296144; 325003 |
| NT01CS0012 | 3 | 21229881; 21030539; 20685834; 20335363; 20194103; 19825675; 12663220; 19653227; 2125350; 19466564 |
| NT01CS0157 | 3 | 21183069; 21074048; 20920790; 20620870; 20608745; 20497333; 20128627; 20061535; 19646181; 19462216 |
| NT01CS01767 | 8 | 21237173; 20655986; 1999397; 19895788; 8550433; 18789896; 17712554; 16728956; 16294303; 15983415 |
| NT01CS0387 | 18 | 17932225; 15287594; 7830601; 7783647; 7537337; 7929167; 8052131; 6348505; 13898172; 3100814 |
| NT01CS0394 | 2 | 18053578; 17451239; 17377573; 6452875; 12230556; 9387; 9375378; 131711; 3888976; 6547672 |
| NT01CS05867 | 8 | 17942405; 11913457; 17636255; 7984417; 10473374; 9090073 |
| NT01CS0602 | 1 | 19394346; 19041910; 18765924; 18242192; 15383717; 12570844; 3700390; 11731178; 1259145; 10508663 |
| NT01CS0657 | 3 | 19857646; 16556217; 15170403; 9157238; 7565110 |
| NT01CS0658 | 3 | 21097635; 20796283; 20738375; 10944393; 18616949; 15105427; 10094619; 17007878; 16968224; 16556217 |
| NT01CS0712 | 18 | 21143936; 21131908; 20937906; 20881245; 20879691; 20877283; 20870764; 20855745; 20828170; 20826447 |
| NT01CS0722 | 3 | 20128627; 17936593; 11121068; 3307916; 16965759; 16922630; 16789876; 16701071; 16476725; 15657055 |
| NT01CS0808 | 8 | 21253719; 21205211; 19923736; 19328460; 18640127; 18066434; 3903497; 15590681; 6295879; 7758956 |
| NT01CS0819 | 6 | 19393175; 18418087; 10397770; 11960995; 11554310; 10430892; 9852053; 8736558; 8462727; 1627644 |
| NT01CS0911 | 4 | 21288904; 21059655; 20713660; 20566764; 20435778; 20086158; 20036252; 1371846; 12654013; 19309146 |
| NT01CS09140 | 8 | 21253866; 21036145; 10672375; 20160912; 20022530; 19874026; 18787477; 19594830; 19464573; 19383527 |
| NT01CS0944 | 8 | 20514546; 17977854; 10641038; 12269813; 10952004; 10716626; 10390816; 9692922; 9642221; 13566053 |
| NT01CS0994 | 18 | 9135111; 1335716; 8159684; 1593610; 2425624 |
| NT01CS1024 | 8 | 16863643; 15910742; 15475358; 15184552; 11017202; 10544288; 10486564; 10471783; 10393339; 9737851 |
| NT01CS1026 | 8 | 15910742; 11017202; 10544288; 9737851; 9665692; 9548917; 9484234; 9443817; 9298948; 9169436 |
| NT01CS1031 | 3 | 20876532; 20855724; 20853818; 20826347; 20385763; 8862584; 20175558; 20154134; 20088879; 20028080 |
| NT01CS10901 | 8 | 19453508; 18926808; 17669536; 17150757; 16556607; 9757107; 16326697; 16242617; 15691337; 12824170 |
| NT01CS1130 | 18 | 21220482; 20947811; 18041952; 19019576; 18403237; 17600077; 10858363; 16809025; 16172537; 15499527 |
| NT01CS1154 | 18 | 21143936; 20881245; 20877283; 20870764; 20730247; 20656779; 20626869; 2017436; 20482643; 20479005 |
| NT01CS1174 | 9 | 21386822; 21381897; 21346005; 21325291; 21318485; 21310233; 21207966; 21189138; 21177752; 21159442 |
| NT01CS1183 | 12 | 19839645; 17390395; 16289685; 15882050; 15159592; 12946361; 12718528; 12297235; 5697999; 10852868 |
| NT01CS12922 | 8 | 11803023; 10446163; 10216163; 9521736; 1731062; 8550613; 7947903; 8120006; 8334158; 8419359 |
| NT01CS1447 | 6 | 21317331; 21227918; 21087076; 21030440; 20920291; 20805881; 20686482; 20675723; 19733176; 230505 |
| NT01CS1486 | 11 | 20063181; 17198713; 13434; 16457867; 7975237; 8178454; 4105995 |
| NT01CS1487 | 11 | 20056615; 17428497; 6283090; 10465774; 17428497; 16377618; 16008350; 15733918; 15063737; 12466275 |
| NT01CS1610 | 12 | 21343297; 21185091; 21128822; 20969977; 20946870; 20944418; 15980438; 20652663; 20440617; 20389065 |
| NT01CS1704 | 6 | 21109751; 16989934; 9783263; 9609952; 8627883; 7763245; 8588247; 8485224; 1302503; 1663344 |
| NT01CS1727 | 12 | 21387310; 21375706; 21375584; 21368222; 21368221; 21367592; 21362116; 21327044; 21321049; 21320870 |
| NT01CS1774 | 5 | 20053710; 20044451; 19077168; 17983588; 17450323; 8393782; 15849794; 14757162; 10224133; 7972072 |
| NT01CS1892 | 14 | 20374530; 8706691; 8569197; 7803457; 1324836 |
| NT01CS1913 | 15 | 20026409; 18455494; 19055322; 17127770; 4103787; 16125667; 15966732; 14574328; 8563639; 4079800 |
| NT01CS19327 | 8 | 21310479; 21278273; 21197843; 21169482; 20965335; 20923481; 20860559; 20855003; 20838866; 20808573 |
| NT01CS1971 | 2 | 18353784; 16624818; 17950386; 9658014; 10428033; 15792955; 12440154; 10777520; 10760477; 10419476 |
| NT01CS19900 | 4 | 9869407; 3803391; 9570121; 9325308 |
| NT01CS2030 | 3 | 20688826; 20064164; 19519768; 19415239; 12051947; 17322183; 16337610; 15667282; 15667278; 15073303 |
| NT01CS2039 | 5 | 21392199; 21233162; 21212946; 21199252; 21169452; 21134098; 21131018; 21059110; 21045297; 20934202 |
| NT01CS2076 | 4 | 18719175; 17015641; 8755888; 10468575; 8107139; 1404383; 1551848; 1905667; 2181149; 13770074 |
| NT01CS2079 | 4 | 21278755; 20421493; 20118266; 19889085; 19665005; 19174164; 18848888; 18216859; 18216858; 10564473 |
| NT01CS2092 | 4 | 20576620; 19542284; 10829079; 7608087; 10832645; 3309346; 3039148; 6360991; 1474584; 14220656 |
| NT01CS2093 | 4 | 20576620; 6259126; 1560774; 1397334; 3039148; 1474584; 3288541; 2181149; 13770074; 3076089 |
| NT01CS2101 | 17 | 20304988; 19245942; 12864862; 15916611; 16673084; 15175291; 10572114; 11934612 |
| NT01CS2116 | 4 | 21382340; 20716375; 20203055; 9722640; 11158352; 18174138; 10063642; 15687183; 15528673; 15361076 |
| NT01CS2155 | 4 | 21167274; 20581225; 16487743; 18391446; 18174138; 16806204; 11287152; 16436427; 15528673; 12958592 |
| NT01CS2165 | 8 | 21299470; 21210973; 20969756; 20870711; 20734996; 20405048; 20340124; 20067522; 9927721; 15967443 |
| NT01CS2287 | 6 | 21391739; 21383100; 21382914; 21372421; 21368764; 21351566; 21319705; 21315610; 21301105; 21300644 |
| NT01CS23580 | 4 | 19089528; 16569716; 10648517; 9168611; 12160316; 2651416; 11251828; 16349329; 8824631; 9868765 |
| NT01CS2431 | 14 | 21041498; 10074353; 11323713; 9598063; 2192230; 2687276 |
| NT01CS2527 | 8 | 21225061; 21082301; 21045201; 20947616; 20810540; 20646194; 20634336; 20629583; 20595377; 20591656 |
| NT01CS2687 | 18 | 20387456; 20335169; 19919671; 16101996; 15223320; 11895295; 19153809; 19121687; 9453631; 18564683 |
| NT01CS2704 | 14 | 20450872; 10606815; 20217113; 20176129; 20026305; 19923723; 19896456; 19749013; 19699693; 19629564 |
| NT01CS2745 | 8 | 20473443; 3545499; 19361226; 18692066; 18331844; 18093543; 17956189; 17675287; 16179963; 15894171 |
| NT01CS2878 | 8 | 20525824; 20134243; 18214971; 18060533; 17692531; 15914052; 15518536; 7828077; 15313611; 15103146 |
| NT01CS3211 | 18 | 14560025; 15797382; 15618217; 14981507; 9644977; 10367886; 7556166; 9367739 |
| NT01CS3297 | 8 | 20610779; 19904590; 18454933; 18358763; 17634376; 17244482; 16098512; 12914915; 12615344; 12515529 |
| NT01CS3302 | 8 | 21339825; 18651753; 20056102; 19664596; 19638432; 18551278; 18454933; 17634376; 10952301; 17244482 |
| NT01DG0076 | 12 | 21365622; 21351072; 21348480; 21344411; 21342114; 21330387; 21327498; 21320871; 21318132; 21314638 |
| NT01DG0085 | 13 | 20942800; 11741992; 19278642; 3902358; 18570629; 15525707; 18095712; 17541948; 1638630; 16998202 |
| NT01DG0153 | 9 | 11170837; 11032827; 10589836; 9427398; 9268358 |
| NT01DG0185 | 6 | 21081493; 21050313; 21059911; 20951312; 20929949; 20871851; 20852630; 20739465; 20734660; 20682246 |
| NT01DG0279 | 3 | 21371235; 21311024; 21304951; 21256201; 21245931; 21245534; 21234771; 21227987; 21115127; 21097635 |
| NT01DG0412 | 15 | 21068390; 20875083; 20807376; 20211130; 20079730; 19996103; 12638084; 19820703; 19749375; 19713957 |
| NT01DG0463 | 11 | 15387827; 15225314; 9829919; 2548993; 7934869; 9126837; 3029382; 7625275; 2826132; 8021940 |
| NT01DG0589 | 6 | 21301105; 21276629; 21221925; 21216167; 21151213; 20952546; 20870749; 20852981; 20844218; 20817842 |
| NT01DG06870 | 4 | 20091748; 20020303; 17437059; 7515185; 17123099; 17114922; 16963428; 9780336; 10406127; 16109414 |
| NT01DG09165 | 2 | 20807370; 3372162; 8624411; 19392686; 18757491; 18202289; 17851775; 16868079; 17039370; 16492736 |
| NT01DG09472 | 8 | 21366490; 21307286; 21262951; 21173220; 21167381; 21144829; 21084781; 21076104; 21071346; 21057854 |
| NT01DG0993 | 4 | 17229554; 15935863; 15379733; 15109736; 8119588; 12654734; 12044681; 1195397; 8985659; 3579257 |
| NT01DG0995 | 4 | 21359593; 21167031; 21059273; 20950636; 20947511; 20938527; 20851900; 20821443; 20723231; 20702768 |
| NT01DG1416 | 1 | 21394321; 21358708; 21339095; 21334398; 21193302; 21185933; 21173738; 21128869; 21070369; 21045271 |
| NT01DG15722 | 4 | 21336990; 19737354; 10692373; 14960717; 18573177; 10409682; 10048040; 15165230; 16416128; 15870446 |
| NT01DG1701 | 6 | 20412069; 19896942; 19478237; 19250833; 18776493; 17299348; 10873856; 16202983; 15983408; 15797986 |
| NT01DG1773 | 6 | 20407851; 20391772; 19597696; 19420796; 19053129; 16838276; 16835767; 11472182; 10543970; 15109812 |
| NT01DG1947 | 4 | 21333546; 21220584; 21214651; 21193611; 21187014; 21147776; 21062893; 20935174; 20921219; 20810321 |
| NT01DG1995 | 8 | 11046083; 16759640; 16672595; 1900238; 12408187; 10319590; 9932647; 9790673; 1998334; 9593868 |
| NT01DG2009 | 6 | 21386893; 21376055; 21358629; 21357421; 21355588; 21354178; 21331256; 21310295; 21307942; 21300896 |
| NT01DG2019 | 1 | 9778800; 16593885; 9639316; 7874737; 14190241 |
| NT01DG2169 | 3 | 21344880; 21344461; 21265802; 21059465; 20980482; 20954046; 20944223; 20833811; 20714501; 20705665 |
| NT01DG2191 | 6 | 21376033; 21368764; 21358820; 21347491; 21331447; 21319705; 21318561; 21304601; 21301105 |
| NT01DG2271 | 4 | 20483662; 20345590; 20032405; 19589519; 17302811; 16442856; 16361939; 15659811; 10482496; 12923898 |
| NT01DGA0016 | 3 | 19298858; 11554469; 18094231; 18029587; 15162539; 10511517; 12797748; 12619034; 11846551; 16667831 |
| NT01DGA0060 | 18 | 19717637; 1744032; 18535149; 16828282; 17297656; 16911515; 6069173; 15850393; 11442842; 11123914 |
| NT01DGA0144 | 2 | 21110975; 20955518; 12472699; 18818314; 18846290; 18846282; 11967097; 17472958; 16915519; 16021340 |
| NT01DGA0251 | 9 | 21391204; 21369973; 21347309; 21322560; 21315569; 21308848; 21256127; 21194627; 21132189; 21093408 |
| NT01DGA0336 | 8 | 18673073; 10436930; 1707310; 1689724; 2833503; 3533929 |
| NT01DO0064 | 12 | 17288554; 11076529; 10484769; 9548919; 9398304; 3512521; 3072256 |
| NT01DO0293 | 8 | 21070748; 20813094; 20046096; 20041317; 19943845; 19883924; 19678839; 10799549; 17333252; 18386080 |
| NT01DO0368 | 3 | 21330432; 21097635; 19857645; 19061404; 10944393; 18433773; 18174140; 15105427; 17468768; 15687210 |
| NT01DO0476 | 4 | 19699535; 12670992; 17683131; 16703280; 11407113 |
| NT01DO0937 | 6 | 15199175; 15843021; 9837717; 3001317; 784901 |
| NT01DO0978 | 18 | 21378750; 21377740; 21375693; 21347827; 21334374; 21298055; 21272653; 21257768; 21255413; 21251950 |
| NT01DO1017 | 15 | 17085583; 15374661; 11980491; 7607244; 8365476; 3691501 |
| NT01DO1658 | 9 | 18032383; 18407998; 18171025; 15531764; 15344554; 14550650; 12840019; 12397064; 11591162; 10841782 |
| NT01DO1714 | 11 | 20533823; 18056646; 20140532; 1948046; 18789947; 20112711; 19951359; 19943076; 19841219; 19622748 |
| NT01DO2398 | 8 | 21320626; 20962922; 20833871; 20732951; 20707314; 20686173; 20618892; 20597606; 20500561; 20484007 |
| NT01DO3271 | 13 | 18761062; 12962325; 9730282; 2606513; 3426607 |
| NT01DO3441 | 8 | 20188887; 17683131; 16851284; 12589573; 12580599; 11939777; 9930987; 9346301; 8288565; 1660303 |
| NT01DO3507 | 8 | 18289689; 8226720; 1543699; 2687259; 3214167; 6166606 |
| NT01DO3626 | 12 | 20419412; 20064164; 19799526; 19778964; 19361518; 19013157; 10747959; 18247574; 17956229; 17935691 |
| NT01DO3672 | 2 | 16844298; 14621995; 8573500; 8566759; 8335646; 6117313; 3516220; 7310534; 7026552 |
| NT01DS0214 | 12 | 21188170; 21125386; 21034747; 20960817; 20625347; 20580091; 20431301; 20334618; 20179346; 20179356 |
| NT01DS0451 | 4 | 20739453; 20519494; 20485749; 20097757; 20080101; 19958256; 17234601; 19858213; 19625248; 18505790 |
| NT01DS0494 | 5 | 21389773; 21380559; 21369987; 21357487; 21357454; 21321255; 21307384; 21303397; 21303396; 21287139 |
| NT01DS0505 | 13 | 20840452; 18566829; 2517480; 3073106; 17406794; 16121241; 16096273; 11877419; 14981896; 14504302 |
| NT01DS0525 | 8 | 21375368; 20958264; 21041650; 20702580; 20684233; 20541516; 20498354; 20453422; 20387635; 934318 |
| NT01DS0532 | 6 | 21075923; 20979348; 20889748; 20824060; 20798056; 20639326; 20576476; 20497998; 20497227; 20484776 |
| NT01DS1017 | 3 | 19804974; 5700707; 11173485; 15572779; 11278591; 11118459; 11329257; 11173485; 11118459; 11084021 |
| NT01DS1173 | 18 | 21322090; 21284755; 21283636; 21246047; 21192085; 21112065; 21095462; 21048708; 21085165; 20975902 |
| NT01DS1255 | 4 | 20558183; 19705835; 2313702; 19324687; 18427119; 17581122; 10781070; 17163981; 16856941; 11287152 |
| NT01DS13127 | 8 | 18025549; 12925808; 12821154; 12596860; 240767; 3275662 |
| NT01DS1372 | 18 | 17804250; 16679531; 16413831; 16126158; 15748819; 15568867; 4250726; 2947628; 2944541 |
| NT01DS1390 | 12 | 21329698; 20501794; 15843375; 19646995; 18519635; 18804477; 18346739; 16709864; 9767238; 16225851 |
| NT01DS1456 | 4 | 942051; 10574791; 15889412; 16819818; 16186538; 15866712; 15388921; 15048837; 12878592; 12569457 |
| NT01DS1695 | 3 | 21056984; 20824644; 20673699; 16772334; 20540529; 20492133; 20472763; 20466652; 20412017; 15270554 |
| NT01DS17057 | 2 | 17107958; 17960917; 17135273; 17107958; 16672227; 15299374; 15659781; 12207027; 11552267 |
| NT01DS1877 | 12 | 21235483; 20801097; 20444093; 19837092; 10360771; 12426336; 18073115; 10675323; 2167176; 14659543 |
| NT01DS2015 | 15 | 21394102; 21393832; 21393426; 21393367; 21393365; 21393212; 21392374; 21390913; 21390327; 21390267 |
| NT01DS20951 | 8 | 21372759; 21200028; 21176778; 21171089; 21167017; 21145450; 21129777; 21080915; 21078982; 21078906 |
| NT01DS20967 | 8 | 17585504; 16574358; 15068241; 12826263; 11720088; 11181918; 11090953; 11043604; 10640308; 10188600 |
| NT01DS2189 | 8 | 9233812; 12125824; 17639348; 7846053; 15667266; 10727938; 10924909; 6855607; 8764511; 7765318 |
| NT01DS2418 | 6 | 21363969; 21353562; 21353270; 21300774; 21297980; 21225639; 21206756; 21173161; 21156137; 21131275 |
| NT01DS2569 | 15 | 21393072; 21392374; 21383126; 21383015; 21377919; 21342550; 21365674; 21360523; 21360173; 21350773 |
| NT01DS2621 | 18 | 21299754; 21112296; 20969962; 20624476; 20593814; 20571891; 6382731; 20427291; 20141829 |
| NT01DS2637 | 4 | 21394810; 21393238; 21392824; 21392767; 21391842; 21391785; 21391781; 21391649; 21391215; 21391123 |
| NT01DS2674 | 6 | 21362549; 21348638; 21320694; 21292986; 21268715; 21267443; 21265757; 21265740; 21251110; 21242291 |
| NT01DS2843 | 6 | 10089311; 15635155; 11101606; 8913296; 7797467; 7867950; 6301692; 3069589 |
| NT01DS2916 | 2 | 20798996; 20038586; 19267692; 15522295; 14595395; 11215515; 11750128; 9689094; 1856165; 1732195 |
| NT01DS3031 | 12 | 21036998; 20943439; 20844762; 20817772; 20727465; 20691712; 20236645; 20174684; 20019078; 9618442 |
| NT01DS3080 | 4 | 20580470; 16487743; 20199603; 16857668; 11934566; 14500664; 15942694; 11985711; 11518318; 1937792 |
| NT01DS3197 | 8 | 21253866; 21177827; 21122111; 21104132; 21068339; 21060736; 21046341; 21044662; 20938718; 20862323 |
| NT01DS3242 | 13 | 19136613; 11743004; 10637234; 12667458; 2141170; 10464265; 9847405; 9315303; 8893853; 8898393 |
| NT01DS3287 | 11 | 21296759; 20487384; 10373526; 10526214; 9813069; 7791009; 7882433; 7687012; 6656801 |
| NT01DS3513 | 18 | 18845756; 20430809; 19875442; 19853036; 11600639; 10470043; 19210618; 18849006; 18713007; 1705327 |
| NT01DS3537 | 1 | 19650882; 19052366; 18503755; 18062262; 17827659; 11483005; 17451239; 17010158; 16921527; 16675503 |
| NT01DS3606 | 3 | 18755141; 11251840; 1790305; 1790304; 1720755 |
| NT01DS3730 | 15 | 20805227; 20682556; 20665263; 20665259; 20639328; 20152151; 19201803; 19800022; 19117195; 18248426 |
| NT01EC0015 | 12 | 21292744; 20953191; 20926844; 20926695; 20880591; 20865007; 20862304; 20847048; 20832865; 20813055 |
| NT01EC00721 | 8 | 17906139; 16222531; 12699688; 10625676; 11395407; 11732896; 11732895; 2540407; 10769139; 10769138 |
| NT01EC0187 | 12 | 11179216; 9853401; 8748033; 7875565; 2651442; 323852 |
| NT01EC0207 | 3 | 21392323; 21388432; 21371926; 21369988; 21368133; 21357995; 21353274; 21349152; 21347426; 21341767 |
| NT01EC0273 | 4 | 21393550; 21392139; 21390329; 21389095; 21386358; 21385991; 21385874; 21381217; 21378392; 21375397 |
| NT01EC0321 | 1 | 16567425; 10223988; 8663056; 8663055; 7771772; 7648198; 7630323; 8244991; 8226748; 8325851 |
| NT01EC0525 | 8 | 21352800; 16863643; 16040612; 15910742; 15475358; 11170426; 10393339; 9378724; 7499371; 2162836 |
| NT01EC0526 | 8 | 21352800; 21247900; 21071492; 19928831; 18953727; 16863643; 11851414; 11115638; 11017202; 10944359 |
| NT01EC0527 | 8 | 21352800; 20351111; 19928831; 19218360; 18953727; 16040612; 15475358; 11851414; 10944359; 10486564 |
| NT01EC08912 | 8 | 17891922; 16762453; 11178972; 8537344; 7748886; 4864932 |
| NT01EC08942 | 8 | 1888719; 3944115; 2365294; 6380587; 6341609 |
| NT01EC0904 | 8 | 20149100; 18673073; 18457966; 9987136; 10896219; 10436930; 8405944; 2170336; 3138232; 1685007 |
| NT01EC09351 | 8 | 21248708; 20851171; 20702586; 20696150; 20668333; 15647753; 20528923; 20434983; 3687875; 20299676 |
| NT01EC1104 | 1 | 19747453; 16289358; 14646107; 3518706; 11354602; 10607477; 10024454; 9881164; 8695645 |
| NT01EC1166 | 4 | 20302923; 16845028; 18761017; 16264189; 8409360; 15769466; 11082305; 13707010; 165169; 8226661 |
| NT01EC1190 | 8 | 21194520; 20149100; 19734178; 19577535; 19254926; 18930018; 18597483; 18457966; 18205406; 17439244 |
| NT01EC1191 | 8 | 19734178; 18930018; 16474982; 10907557; 15813741; 15347626; 12948635; 10216161; 10076013; 7608187 |
| NT01EC1216 | 8 | 20978811; 19393666; 18549208; 822747; 18311941; 18201106; 17659281; 10440380; 15598502; 15280383 |
| NT01EC1239 | 18 | 18692508; 18156179; 12923181; 12354616; 11756453; 11726714; 11248195; 10521259; 8045426; 9693722 |
| NT01EC1298 | 14 | 16819826; 10903946; 10433703; 7766613; 1979549; 6102565 |
| NT01EC1375 | 15 | 21320584; 21193607; 20937832; 20811812; 20656781; 20547815; 20435045; 20404199; 10850721; 10966457 |
| NT01EC1454 | 15 | 20134246; 17630784; 12111160; 18193310; 14527688; 8869638; 8637901; 15282798; 12207695; 12949161 |
| NT01EC2015 | 2 | 18671734; 17541777; 10411892; 11375002; 7473709; 8055941; 6816587 |
| NT01EC23052 | 4 | 20217281; 8811926; 19209886; 20139731; 10831419; 17292861; 17185556; 8626288; 16420371; 6401282 |
| NT01EC2474 | 14 | 20061477; 19969466; 9817848; 12644510; 15977277; 15159413; 15103135; 14646078; 12785757; 12468709 |
| NT01EC2476 | 3 | 15977277; 14761630; 11096116; 10521660; 10411277; 11553351; 11517612; 7574484; 11065359; 10895688 |
| NT01EC2477 | 3 | 21336933; 17532307; 17202151; 16276532; 15750655; 15695810; 15297914; 14574703; 14570895; 14505409 |
| NT01EC2480 | 14 | 21073876; 20830297; 20724435; 20687808; 7773418; 20435647; 20335578; 17393512; 20238176; 20199575 |
| NT01EC2581 | 3 | 20608745; 20601503; 20525686; 20233306; 20223213; 20025663; 6351730; 1744050; 19400771; 19374992 |
| NT01EC2614 | 6 | 21155533; 21127267; 21030352; 20974932; 20963646; 20955519; 20952393; 20889679; 20872611; 20826193 |
| NT01EC2807 | 9 | 1355089; 15155740; 16212603; 10704200; 10589718; 3556162; 1526981 |
| NT01EC3023 | 5 | 20808906; 20624911; 20221731; 20075614; 7836277; 19968123; 19845287; 19843229; 10872445; 19673421 |
| NT01EC30697 | 8 | 21310654; 20652669; 20039169; 19914581; 18685267; 10428969; 9353918; 16847457; 11092855; 16232495 |
| NT01EC3071 | 8 | 21310654; 17420585; 9353918; 2199796; 16005887; 5048285; 15215602; 12177337; 11390387; 14907713 |
| NT01EC3105 | 17 | 21306637; 21173264; 21133991; 20974746; 20932845; 20869963; 20735118; 20677811; 20660014; 20678145 |
| NT01EC3139 | 13 | 21219451; 21037010; 20817755; 20051305; 17937767; 11014182; 15215462; 17188032; 10383384; 15078091 |
| NT01EC32801 | 8 | 21254729; 21235167; 21124049; 21095085; 21090260; 21067711; 21058504; 21048401; 21031617; 20954071 |
| NT01EC33422 | 8 | 21214572; 18571506; 11369596; 11330037; 10972423; 10692424; 10683770; 10553003; 10456321; 10415121 |
| NT01EC3510 | 8 | 21107318; 20933183; 20456655; 20124719; 20089767; 19860829; 19084582; 18581728; 18549703; 17993624 |
| NT01EC3526 | 6 | 21300909; 21195169; 21131906; 20882051; 20865002; 20858288; 20841362; 20625580; 20564213; 20562326 |
| NT01EC3536 | 8 | 21187861; 21045262; 21044564; 20921368; 20923666; 20926746; 20865032; 20798498; 20731664; 20600164 |
| NT01EC41457 | 8 | 18956748; 12716415; 9220346; 8825442; 8256521; 1482691; 1288496; 1326518; 3215904; 3134890 |
| NT01EC4365 | 13 | 19136613; 11743004; 17502103; 8893853; 10637234; 15870727; 12667458; 2141170; 10464265; 11041351 |
| NT01EC4464 | 1 | 12488095; 11243831; 10581183; 8507653; 8274527; 1872874; 1108015; 6237648; 6279571 |
| NT01EC4498 | 6 | 15000738; 10026280; 11406172; 11399091; 11278077; 11150671; 11124026; 11103941; 10893304; 10826892 |
| NT01EC4500 | 6 | 8662184; 19796904; 17895578; 17696533; 16959568; 15916611; 11443091; 16325764; 16150738; 14728866 |
| NT01EC4509 | 18 | 21325831; 21307243; 21289069; 21170887; 21250979; 21242547; 21222362; 21209192; 21209188; 21270777 |
| NT01EC4520 | 18 | 17541836; 8131922; 7934851; 8405961; 2475740; 2849017; 6086106; 6111986; 4892010 |
| NT01EC4626 | 1 | 20057066; 19307721; 19013471; 18269631; 18023379; 17889830; 10331867; 16327902; 14747737; 12948639 |
| NT01EC4662 | 8 | 14551431; 16686476; 12177066; 2050627; 10373427; 8663185; 8647136; 8530465; 2254256; 8132544 |
| NT01EC4791 | 4 | 19090721; 9041419; 8990289; 19621737; 14720437; 14614594; 14600231; 14596968; 14596345; 10428945 |
| NT01EC4822 | 18 | 19025561; 11005762; 9572993; 9490701; 3943900; 3568126 |
| NT01EC4894 | 1 | 21055431; 20723595; 20651233; 20594879; 20446760; 20463181; 20381632; 20347576; 20138844; 19936712 |
| NT01EC5035 | 18 | 20363944; 19569551; 3018721; 7984417; 18305482; 12883005; 18084014; 16079298; 11524131; 11467726 |
| NT01EC52892 | 4 | 21265802; 21252279; 7991552; 9632599; 17396114; 16879977; 16280466; 15716072; 2045123; 15116313 |
| NT01FJ0010 | 9 | 1621963; 7024730; 3707134; 6749495; 7263650 |
| NT01FJ0204 | 18 | 20864546; 12007800; 20494559; 20358851; 20202283; 19759494; 18513920; 16882325; 16807861; 16426480 |
| NT01FJ0482 | 12 | 20828565; 18502756; 10085018; 17415567; 17267443; 12707267; 10426317; 11533066; 9882712; 9187245 |
| NT01FJ07787 | 8 | 21392042; 21377330; 21366559; 21361867; 21359968; 21359206; 21352963; 21328391; 21327936; 21324164 |
| NT01FJ0821 | 3 | 15292578; 10464298; 4563441; 8486688; 1826463 |
| NT01FJ1111 | 14 | 21394109; 21394105; 21214674; 21189470; 21150937; 21111048; 20939692; 20877497; 20388844; 20371678 |
| NT01FJ11851 | 8 | 20883732; 20693678; 19758330; 19459980; 18188553; 1513331; 11690652; 11919723; 17890304; 18093969 |
| NT01FJ1279 | 8 | 21078123; 21073854; 19616102; 19589965; 19307254; 19191964; 18765916; 18390572; 17188300; 17146529 |
| NT01FJ1289 | 3 | 20526724; 20158191; 19965803; 19476019; 18691671; 18335278; 10436082; 11513133 |
| NT01FJ14924 | 8 | 20602355; 20164409; 20054115; 19852484; 10082381; 19132841; 17242363; 18318536; 12764073; 16990279 |
| NT01FJ1632 | 3 | 20547785; 18809265; 8631709; 10542235; 2066344; 10037771; 14907713; 3073932; 6094494; 3897174 |
| NT01FJ1726 | 8 | 21362485; 21354471; 21346809; 21335525; 21332407; 21329881; 21322032; 21299470; 21278127; 21247928 |
| NT01FJ1825 | 2 | 20550915; 20445263; 19924845; 19631695; 19191740; 18804704; 18007032; 17362087; 17289662; 17029412 |
| NT01FJ1987 | 3 | 21151979; 20201406; 17934909; 16418723; 15341635; 15162539; 14646108; 14561509; 12928628; 12565992 |
| NT01FJ2094 | 12 | 21203457; 21118802; 20936072; 15020458; 20467052; 20441441; 20206636; 19947526; 2984171; 10585965 |
| NT01FJ22231 | 8 | 21387398; 21382395; 21377703; 21376739; 21375708; 21369920; 21358122; 21356001; 21350582; 21348473 |
| NT01FJ2622 | 1 | 21113737; 20300853; 20196394; 19924905; 19778996; 10805808; 19235232; 18054776; 17884813; 15451668 |
| NT01FJ26912 | 8 | 20957036; 20057150; 19622650; 19389776; 18720501; 18494733; 17698881; 6388497; 12097242; 16228324 |
| NT01FJ2698 | 18 | 20444417; 16932613; 16804172; 12644235; 11283292; 11136469 |
| NT01FJ2781 | 12 | 20055834; 19851712; 19802820; 19694804; 19420130; 19386397; 19007765; 18721817; 18222721; 18178555 |
| NT01FJ2803 | 6 | 11532130; 8407835; 3034859; 10564481; 10413660 |
| NT01FJ3232 | 11 | 21145536; 21037014; 20662701; 20404718; 19889873; 7665476; 19555815; 19166012; 17882282; 18256309 |
| NT01FJ3243 | 3 | 21129205; 15979388; 15501819; 15196039; 9294425; 10588048; 9501156; 9161409; 7540246; 15305587 |
| NT01FJ3260 | 18 | 21283567; 21169917; 21163569; 21143936; 21041489; 20951970; 20937890; 20807201; 20600957; 20578522 |
| NT01FJ33534 | 8 | 21386820; 21177247; 20615935; 20603889; 20197271; 20162365; 20147708; 20147709; 20138557; 20040070 |
| NT01FJ3366 | 4 | 20227693; 19695063; 18212794; 19246762; 19197989; 19049830; 19374128; 17944819; 17931347; 15378046 |
| NT01FJ36244 | 8 | 21098515; 21067711; 20882275; 20724279; 16290006; 20412461; 20015334; 19533311; 15181203; 19050861 |
| NT01FJ3655 | 12 | 20654624; 20083401; 19723579; 19714768; 9252580; 19238259; 18818204; 18524928; 17244532; 16563798 |
| NT01FJ3657 | 14 | 10747855; 2556396; 10995231; 10375643; 6160384; 8730877; 184817; 7128581 |
| NT01FJ4336 | 2 | 21296160; 21245535; 20806221; 20230506; 18534824; 18298940; 18262398; 18219123; 10438747; 17541777 |
| NT01FJ43704 | 8 | 21346408; 21248490; 21070417; 21047623; 20962906; 20884040; 20691085; 20607763; 20558896; 20549807 |
| NT01FJ4384 | 11 | 21244827; 21073237; 21062824; 20870769; 20660193; 16012093; 20118255; 20038591; 19809101; 19747486 |
| NT01FJ44805 | 2 | 21364293; 20471481; 20442400; 20405048; 19946146; 19798741; 19574216; 18606475; 19305405; 18639525 |
| NT01FJ4619 | 4 | 19674109; 19523447; 19308706; 19135891; 12218017; 17005014; 11406600; 15963662; 15827603; 11158581 |
| NT01FJ4687 | 17 | 21188417; 20855293; 18681940; 10686096; 7030739; 987906; 14507369; 12423786; 11723135; 11152597 |
| NT01FJ4693 | 6 | 21213249; 21209864; 21203948; 21167302; 21123385; 21114823; 21110982; 21068843; 21030679; 20890269 |
| NT01FJ47294 | 8 | 21193067; 16926507; 12052060; 11853483; 11267646; 9511752; 7515357 |
| NT01FJ4870 | 18 | 21179087; 21068099; 20378542; 20302775; 19131115; 19006689; 19006693; 15644776; 10601998; 10229103 |
| NT01FJ4873 | 12 | 20558177; 18974993; 19921850; 19863057; 19809370; 19804409; 19699792; 19371136; 19156482; 18979624 |
| NT01FJ52834 | 8 | 20933405; 20870130; 20724279; 20722299; 20599378; 20453546; 20438857; 20438497; 20417397; 20394004 |
| NT01FN0221 | 18 | 11054294; 18297445; 10700280; 16451184; 16359323; 15896319; 15767250; 2836362; 15336398; 15118661 |
| NT01FN02954 | 8 | 21137052; 21136951; 21063414; 20946652; 3404544; 20206694; 20178988; 20145562; 20064929; 19542868 |
| NT01FN0469 | 13 | 21372178; 21115661; 20937798; 20580948; 19806386; 16113212; 17033296; 16609814; 16230336; 15750311 |
| NT01FN0679 | 12 | 20159555; 19455308; 18256511; 16973605; 16271702; 16233770; 15130131; 15023062; 10025914; 10893253 |
| NT01FN0699 | 9 | 19843251; 19694421; 19082744; 18824113; 18310028; 15797603; 17092943; 2853689; 12604210; 16040614 |
| NT01FN0729 | 15 | 21165645; 16526955; 19665022; 11731473; 9878637; 10545328; 18177941; 16620205; 16386432; 20565676 |
| NT01FN0868 | 12 | 21307284; 21295323; 21285375; 21278787; 21236367; 21235734; 21185362; 21183720; 21144839; 21127511 |
| NT01FN0957 | 14 | 20047307; 19683539; 18536021; 17425969; 17384901; 17191912; 999839; 17143579; 16928426; 16714288 |
| NT01FN10704 | 8 | 21205672; 21188072; 21092180; 21054963; 21030539; 20724435; 15695395; 20473714; 20388532; 20331963 |
| NT01FN1777 | 17 | 21183650; 20841381; 20833730; 20625716; 16775306; 20378243; 20361508; 20208560; 20181721; 20087629 |
| NT01GM0116 | 12 | 21335977; 21326941; 21242965; 20889486; 20827447; 20735358; 20648511; 20583963; 20556630; 20525865 |
| NT01GM0395 | 8 | 11751810; 11418115; 10812085; 9387241; 8661925; 1625581; 14259761; 2200847; 2187144; 3332683 |
| NT01GM0700 | 5 | 20639477; 20942470; 20926829; 20884351; 20870729; 20817730; 20697102; 20693316; 20651844; 20645701 |
| NT01GM0775 | 12 | 19897920; 9729602; 8645734; 1732209; 8335078; 3120701 |
| NT01GM0966 | 12 | 21391672; 21382890; 21376320; 21369989; 21362190; 21354644; 21352602; 21335599; 21333981; 21332221 |
| NT01GM1041 | 15 | 21364902; 21315771; 21300840; 21257771; 21219466; 21217003; 21216906; 21193607; 21179017; 21124821 |
| NT01GM1165 | 8 | 19350381; 11042190; 18808366; 18242195; 9371838; 10497023; 3294097; 11098150; 10682350; 10486579 |
| NT01GM1174 | 18 | 21274617; 18796009; 10559939; 12860994; 16361263; 16307477; 16230350; 15215855; 16162497; 15803409 |
| NT01GM1193 | 12 | 20558178; 19771157; 20064458; 20045481; 19761755; 19680265; 3554251; 15491160; 19022390; 18953687 |
| NT01GM1234 | 3 | 17376078; 17038794; 12626683; 9721302; 1650815; 5432063; 8093697; 2228971 |
| NT01GM1277 | 12 | 21393419; 21389833; 21389345; 21389278; 21389123; 21388679; 21386766; 21383022; 21376466; 21376163 |
| NT01GM1373 | 11 | 20736234; 19788700; 19571721; 16403458; 4407662; 11145416; 1398942; 9202480; 7590258; 3035345 |
| NT01GM1386 | 3 | 16151406; 16416971; 16331565; 15809881; 12645255; 11606829; 11131918; 10319583; 10085528; 9760207 |
| NT01GM1559 | 3 | 21394312; 21367879; 21361872; 21356201; 21330144; 21325338; 21308395; 21303663; 21286806; 21284263 |
| NT01GM1596 | 9 | 20110695; 18709443; 18640292; 17116638; 16630629; 16657699; 15589131; 14970259; 14630990; 14605499 |
| NT01GM1606 | 8 | 21385190; 21281422; 20428224; 19799633; 10698784; 17359274; 12269751; 10515931; 9632263 |
| NT01GM1629 | 11 | 21391235; 21387172; 21367866; 21359008; 21343707; 21339331; 21333710; 21333649; 21327254; 21320060 |
| NT01GM2167 | 9 | 20110695; 16750167; 15885896; 8135828; 8253773; 8507687; 8012501; 1958319; 2297537 |
| NT01GM2171 | 8 | 16377226; 15885896; 12555941; 1909402; 3930843 |
| NT01GM2703 | 8 | 21228234; 21106768; 20809899; 20620150; 5420057; 20516620; 20479254; 20435888; 20171064; 20096472 |
| NT01GM3107 | 6 | 21187477; 21155533; 21127267; 21030352; 20974932; 20969882; 20964789; 20963646; 20955519; 20952393 |
| NT01GM3247 | 18 | 20981744; 18849422; 20035006; 15306012; 19254725; 19695261; 19574656; 19361527; 12724524; 19329985 |
| NT01GM3256 | 6 | 19332813; 16701584; 16024301; 14711809; 12172806; 11703661; 11165501; 11004560; 10880511 |
| NT01GM3352 | 8 | 21380436; 21305036; 21272750; 21220703; 21220358; 21209947; 21185447; 21106065; 21085043; 21081705 |
| NT01GM3412 | 8 | 9665741; 1332073; 2195031; 3446585; 3813556; 3930843; 6304077 |
| NT01GM3554 | 6 | 20628184; 20413787; 17766684; 20041869; 19556779; 19161349; 19148637; 10469642; 18817159; 18180777 |
| NT01GM3569 | 13 | 17187224; 12736709; 12645924; 11779468; 8455623; 9829824; 8711484 |
| NT01GV0079 | 11 | 21393370; 21375706; 21368277; 21356525; 21350632; 21335390; 21323982; 21310067; 21277379; 21262357 |
| NT01GV0457 | 14 | 18388293; 17023639; 16865708; 16544324; 15566465; 15060594; 9328467; 1398079; 8224889; 7284307 |
| NT01GV0668 | 5 | 20803367; 20679209; 20633042; 20523025; 20508633; 20422505; 20183076; 17711875; 20097723; 20086107 |
| NT01GV0759 | 15 | 15507638; 9125041; 6322134; 3023697; 6289323; 3494857; 6096556; 3018543 |
| NT01GV0879 | 2 | 21393834; 21345995; 21278388; 21241799; 21301021; 21099244; 20934804; 20876213; 21193426; 20732302 |
| NT01GV0989 | 8 | 19370345; 10438748; 12732969; 12135383; 942051; 7130163 |
| NT01GV1007 | 6 | 21314600; 20178456; 15133681; 15200950; 17540172; 7957165; 16690032; 15958184; 15610014; 15192207 |
| NT01GV1235 | 8 | 18673073; 18089934; 8621585; 7499371; 1707310; 1689724; 2544229; 2833503; 3533929 |
| NT01GV1601 | 8 | 18673073; 18089934; 1689724; 2833503; 3533929 |
| NT01GV1685 | 11 | 20547379; 16181782; 17078817; 16731525; 10722135; 10986230; 10556026; 9393713; 2548993; 10760133 |
| NT01GV1762 | 12 | 21365808; 21309657; 21265340; 21244406; 21244412; 21226699; 20212195; 20707801; 21195875; 21195836 |
| NT01GV2178 | 15 | 20863231; 20380929; 19167902; 19153821; 17701900; 18765922; 10395796; 18051762; 17095013; 10336488 |
| NT01GV2234 | 4 | 21311889; 21194629; 20930278; 20822460; 20819221; 20800516; 20728562; 20688956; 20657939; 20575514 |
| NT01GV23577 | 8 | 21227686; 21177104; 21110068; 21074990; 20972919; 20869471; 20835480; 20655745; 20632934; 20533943 |
| NT01GV2400 | 3 | 21311938; 21251258; 21151985; 21078344; 20729317; 20726810; 20726806; 20604574; 20547748; 20497501 |
| NT01GV2428 | 1 | 20601064; 19931317; 19921396; 16857674; 19249065; 18342636; 18030681; 942051; 17937657; 17869212 |
| NT01GV2662 | 12 | 21324206; 21295606; 21252346; 21246269; 21235526; 21205794; 21205672; 21195058; 21194850; 21151101 |
| NT01GV3437 | 2 | 19646414; 19291068; 11172074; 15634204; 10437802; 393803; 5432063 |
| NT01GV3511 | 14 | 21302675; 21268919; 21248836; 21217689; 21217679; 21170047; 21199573; 21217678; 21137981; 21111108 |
| NT01GV3900 | 1 | 21311031; 17975734; 15564524; 12972346; 8774714; 7766043; 8107678; 8223630; 16662653; 16659452 |
| NT01HB0209 | 8 | 20610779; 10952301; 16756485; 15943805; 15747169; 12914915; 11695833; 11245799; 13382877; 8033096 |
| NT01HB0515 | 12 | 18818768; 12032318; 9447962; 10362520; 9255067; 9211982; 8034598; 2898766 |
| NT01HB0541 | 12 | 18846552; 15808850; 12796498; 8521970; 7677788 |
| NT01HB05681 | 8 | 21325606; 21299565; 20926376; 19053141; 18717738; 2118958; 17329775; 17303759; 17074752; 16530868 |
| NT01HB0929 | 8 | 20738089; 19725515; 19118348; 7547908; 11125067; 8425532; 16390443; 16213671; 15720393; 10052943 |
| NT01HB0931 | 2 | 21166638; 21147059; 21081498; 20944207; 20709651; 20675294; 20669954; 20415463; 20375021; 20346907 |
| NT01HB1058 | 12 | 17092293; 15808850; 12796498; 11381270; 10064137; 8521970; 7677788 |
| NT01HB1117 | 6 | 21388532; 21378185; 21365542; 21350762; 21343909; 21336027; 21325134; 21321231; 21216906; 21145896 |
| NT01HB1203 | 8 | 18716757; 17185539; 14576151; 12489779; 12409197; 11926996; 10482517; 10826693; 9546395; 9531636 |
| NT01HD0137 | 12 | 21393853; 21372823; 21371608; 21358050; 21356266; 21355050; 21352928; 21343428; 21342622; 21340517 |
| NT01HD0668 | 8 | 20624914; 20622059; 20594980; 7640264; 21136923; 18439144; 16850161; 16807885; 16436434; 16343536 |
| NT01HD0852 | 4 | 21325274; 19432486; 19254725; 17214741; 16162665; 10529352 |
| NT01HD0858 | 18 | 19130255; 18394146; 17206384; 17005994; 10231495; 16640594; 12379679 |
| NT01HD0929 | 9 | 21172869; 20593270; 20385763; 19853312; 19754149; 17118978; 17318230; 18807027; 7961456; 12183584 |
| NT01HD0963 | 2 | 19890837; 12628916; 16857941; 16109844; 1710853; 10601252; 12564938; 8755716; 8110778; 7687288 |
| NT01HD1263 | 4 | 20006310; 18208388; 17518418; 17408592; 16638632; 16563799; 16563797; 16483314; 15037246; 14514680 |
| NT01HD1426 | 6 | 21347417; 21347256; 21310716; 21291520; 21288492; 21223548; 21048863; 21030504; 20809776; 20637278 |
| NT01HD1481 | 12 | 8087850; 10582129; 6384729; 8462840; 2679887; 8387148; 1588819; 2088168; 2041472; 2170107 |
| NT01HD1517 | 4 | 20600507; 10592175; 16790025; 16309817; 12927080; 12521307; 12354237; 10361282; 9491412; 9258332 |
| NT01LC0019 | 11 | 20418401; 15635004; 19416360; 18632228; 18065168; 18022196; 17367392; 15500249; 15469518; 10540284 |
| NT01LC0140 | 4 | 21195227; 21183019; 21141468; 21040511; 20979348; 20971906; 20935130; 20870796; 20842358; 20798585 |
| NT01LC0174 | 8 | 20739284; 20693675; 20606278; 19566721; 19052639; 18430452; 16678422; 16086247; 11976112; 15899413 |
| NT01LC02020 | 4 | 17531373; 17505307; 17438333; 16878031; 16775571; 16135970; 15513317; 12213923; 10923398; 10889354 |
| NT01LC0422 | 15 | 20946850; 20662766; 20660346; 20097862; 19766560; 9048379; 19571676; 8520220; 14630926; 19194483 |
| NT01LC0497 | 11 | 20406289; 17616596; 18761693; 18048923; 10438592; 9988480; 9799632; 9454717; 9201223; 7669337 |
| NT01LC06244 | 8 | 8309940; 17504469; 2446923; 2256682; 14606945; 11191810; 8238872; 10816581; 10361281; 9682471 |
| NT01LC0652 | 4 | 20846957; 18479053; 8824637; 7704255; 6801021; 3038682 |
| NT01LC0711 | 15 | 21193612; 21124051; 20965063; 20952652; 20447997; 20413552; 20192973; 20158521; 20122918; 19159310 |
| NT01LC07652 | 4 | 20875395; 19682914; 18335939; 18930130; 18483760; 16630813; 17014073; 16686542; 16436421; 15751951 |
| NT01LC0795 | 3 | 19138560; 10572138; 12134257; 12028417; 11849532; 9593300; 10197997; 6223998; 9927713; 10694878 |
| NT01LC0807 | 18 | 14500688; 19768783; 18923064; 18692463; 10995236; 11292341; 9705332; 2545248; 6606179 |
| NT01LC0899 | 12 | 9194175; 18373437; 18331844; 14688122; 17726569; 10214959; 16150729; 15291821; 15163408; 12766342 |
| NT01LC1093 | 12 | 19583797; 10406960; 10050287; 8661695; 6350155 |
| NT01LC11171 | 8 | 20924357; 11580842; 9882659; 14514681; 12963714; 12829297; 12777497; 12724394; 11426055; 11327758 |
| NT01LC1128 | 3 | 8662184; 19740347; 19686739; 19441232; 6852916; 9158760; 18490448; 18369905; 18602342; 18305117 |
| NT01LC1294 | 15 | 21289859; 21245161; 21205660; 21164006; 21131906; 21097719; 21084449; 21070771; 21055941; 21075303 |
| NT01LC1299 | 4 | 21359956; 21255117; 21224850; 21224215; 21183069; 20870765; 20863890; 20847002; 20729359; 20723778 |
| NT01LC1525 | 6 | 21071401; 9092551; 8254319; 10754547; 10756102; 9781875; 9004227; 8885269; 14292994; 8387145 |
| NT01LC1610 | 4 | 21112337; 21081474; 20713661; 20675578; 15946826; 19925844; 19486940; 19010402; 19368559; 19154787 |
| NT01LC1698 | 5 | 21265041; 20600019; 20440620; 20095974; 19960435; 19910419; 19817703; 19723471; 19590824; 1939141 |
| NT01LC1862 | 18 | 21104182; 21030610; 20947298; 20558765; 20150244; 17854384; 19557803; 19549526; 12034734; 18094991 |
| NT01LC21614 | 3 | 21393196; 21389348; 21380641; 21376539; 21369988; 21354401; 21350802; 21341767; 21338683; 21338414 |
| NT01LC2324 | 6 | 21357745; 21354867; 21350489; 21336027; 21297161; 21293191; 21274582; 21263027; 21236302; 21235642 |
| NT01LC2885 | 15 | 21219466; 19303806; 18792692; 18789936; 9829949; 17994770; 10331874; 16430694; 15306019; 15015732 |
| NT01MC0158 | 15 | 20947637; 20562302; 20385176; 19882182; 17959646; 19047357; 18851912; 14512618; 17988687; 17697992 |
| NT01MC0204 | 18 | 21369825; 21244849; 21191107; 21103968; 20962061; 20938698; 20851900; 20804587; 20821001; 20649839 |
| NT01MC0393 | 13 | 21152561; 21030659; 20727857; 20429542; 20097853; 19761689; 10937989; 19454243; 19329136; 18573842 |
| NT01MC1100 | 8 | 21392490; 21391497; 21388535; 21386971; 21384159; 21382478; 21382338; 21378254; 21378180; 21378103 |
| NT01MC1365 | 4 | 21255111; 21179017; 21108067; 21091513; 21062468; 20946845; 20869221; 20808885; 20833311; 20525091 |
| NT01MC1513 | 17 | 14993308; 11763973; 9159399; 9891799; 9680198 |
| NT01MC16724 | 8 | 21199910; 20056699; 13898350; 18594899; 8336105; 17719035; 15672820; 12855718; 12026175; 10473455 |
| NT01MC2674 | 12 | 21394548; 21392585; 21391832; 21380751; 21374789; 21364634; 21355049; 21347624; 21344160; 21341713 |
| NT01MC2915 | 15 | 8939696; 16732458; 15989228; 2126168; 14697518; 12848429; 10357851; 11816976; 11768529; 10383988 |
| NT01MC2990 | 12 | 19454621; 11953442; 18342411; 17374126; 17239953; 16573693; 16076848; 15170231; 12081643; 11860552 |
| NT01MC3022 | 6 | 4942327; 16904387; 12415303; 12793747; 11700277; 11810266; 10645945; 9141693; 338918 |
| NT01MC3126 | 4 | 20889747; 2030670; 18284590; 4631369; 9677288; 16677309; 16260786; 8107139; 12135374; 10629180 |
| NT01MC3661 | 1 | 21281627; 20684326; 20629101; 20522323; 19497206; 17868417; 17661288; 17094902; 16997511; 16978364 |
| NT01MK0069 | 6 | 20457749; 19442247; 19094995; 2320109; 11418610; 10393201; 14235546; 9857048; 1561835; 9368652 |
| NT01MK0183 | 6 | 21393853; 21383087; 21368151; 21327072; 21310715; 21289127; 21283762; 21264994; 21248841; 21248837 |
| NT01MK0436 | 12 | 21254169; 21098295; 20937828; 20812720; 20675386; 20632995; 20505154; 20478922; 20460155; 20355018 |
| NT01MK0561 | 12 | 21390545; 21385872; 21376699; 21376122; 21370482; 21369703; 21366301; 21365622; 21351072; 21348480 |
| NT01MK05833 | 8 | 9325428; 9151968; 1314088; 7649177; 7851382; 8319675; 8481089; 8215796; 1953299; 1772346 |
| NT01MK0729 | 8 | 21375721; 21278411; 20835839; 20334431; 20173004; 10903367; 19667393; 19480946; 16699521; 19719599 |
| NT01MK07461 | 8 | 19683415; 19744166; 2466049; 18318679; 17456317; 17006990; 16521218; 10637234; 16381642; 15747502 |
| NT01MK0958 | 4 | 17601489; 1528267; 19447111; 10607659; 12649276; 18275156; 18179418; 12324359; 17068335; 10831447 |
| NT01MK0974 | 12 | 19812900; 19137198; 11959102; 17565987; 10571178; 16906146; 16623833; 16583313; 15907469; 15221960 |
| NT01MK1214 | 14 | 21035731; 20934342; 20848821; 20808934; 20592645; 20097157; 20061166; 18774912; 19810816; 19798499 |
| NT01MK1246 | 6 | 21236302; 20943892; 20216337; 3571220; 19951946; 19442243; 19435918; 15750208; 19143592; 15829967 |
| NT01MK1445 | 4 | 19283105; 3772980; 18807377; 18365618; 17618290; 17339317; 15272387; 16496175; 10637627; 15615786 |
| NT01MK1551 | 13 | 21393382; 21392573; 21390309; 21387185; 21386885; 21385875; 21385376; 21383700; 21383078; 21382012 |
| NT01MK1672 | 14 | 20515945; 20439498; 15197771; 17167475; 19399913; 19007947; 12686640; 18004711; 17604275; 17425674 |
| NT01MK1692 | 5 | 10903367; 6945871; 17220269; 19006326; 16461668; 18323780; 18252716; 17991025; 17911316; 17472633 |
| NT01MK1713 | 6 | 20637419; 15800630; 16650908; 10504724; 14510073; 10449720; 9421495; 11733530; 11398460; 10430892 |
| NT01MK17713 | 8 | 21375721; 21278411; 20835839; 20173004; 10903367; 19667393; 19480946; 16699521; 19719599; 18398197 |
| NT01MK18143 | 8 | 20944230; 20795494; 20534465; 6501214; 14966965; 12492476; 12102556; 11916257; 11913137; 11532013 |
| NT01MK1821 | 12 | 21393445; 21393328; 21392167; 21391210; 21390246; 21390206; 21390183; 21389986; 21389870; 21389074 |
| NT01MK1887 | 13 | 20694005; 20643101; 20188109; 12105207; 10937989; 11752189; 7677746; 17092626; 1690811; 16076220 |
| NT01MK1978 | 6 | 21394438; 21367571; 21365649; 21362626; 21324423; 21310161; 21307207; 21300884; 21300033; 21271695 |
| NT01MP0002 | 12 | 20803087; 16531398; 15195998; 15184371; 12565881; 8654361; 10543453; 9585178; 9516432; 9521861 |
| NT01MP0026 | 17 | 20068132; 18725266; 1719229; 17383019; 16863505; 16234237; 15641079; 9334325; 12414988; 12242593 |
| NT01MP00711 | 8 | 20920368; 20661555; 20638314; 20589904; 20512975; 20507884; 17101645; 19566723; 19556079; 19235233 |
| NT01MP0164 | 3 | 21209170; 7545182; 8948633; 19502372; 16790744; 18852931; 18287292; 17158214; 10234833; 2516114 |
| NT01MP02230 | 8 | 1741458; 17568655; 16364320; 15839401; 3156376; 8214582; 37402; 10447888; 6546423 |
| NT01MP0419 | 4 | 16707501; 15911093; 15266015; 15123672; 14602719; 12763637; 12538595; 11779148; 9430692; 1987161 |
| NT01MP0502 | 9 | 21392972; 21388805; 21388804; 21387258; 21378159; 21378051; 21377138; 21376217; 21375886; 21374579 |
| NT01MT0102 | 14 | 19403191; 16154080; 15660995; 11751055; 8631867; 8393068; 7030616 |
| NT01MT0141 | 18 | 21389771; 21139218; 20647051; 19824072; 19455515; 12907708; 15262278; 18457969; 12207649; 17257268 |
| NT01MT0185 | 6 | 21388532; 21378185; 21343909; 21321231; 21216906; 21205840; 21173576; 21145896; 21139222; 21126315 |
| NT01MT0195 | 15 | 21338418; 21131439; 21097627; 21075923; 20980392; 20952578; 20941352; 20805337; 20678145; 20573962 |
| NT01MT0229 | 8 | 9381173; 11995829; 11882715; 9973345; 3131330 |
| NT01MT0249 | 13 | 20669187; 16113766; 18400176; 19479258; 18257223; 7690036; 11137292; 15885495; 15642198; 11425264 |
| NT01MT0444 | 2 | 20543028; 19646414; 18693239; 19825533; 17875396; 16463102; 11478896; 8602155; 3042779; 1351299 |
| NT01MT0551 | 2 | 20955518; 18846290; 16915519; 16663535; 3042779; 1351299; 9418040 |
| NT01MT0800 | 8 | 19968326; 17293867; 19022301; 18601652; 18249092; 17977945; 10629216; 16865772; 15809743; 14630917 |
| NT01MT0891 | 12 | 21378193; 21368759; 21357290; 21347827; 21333546; 21324212; 21317324; 21305036; 21289292; 21266658 |
| NT01MT09840 | 4 | 20541640; 20377272; 19615523; 19206465; 8626809; 18807117; 18457413; 19636161; 19825547; 17720689 |
| NT01MT1004 | 12 | 21393844; 21388709; 21385615; 21381077; 21377964; 21372178; 21372129; 21364651; 21362486; 21360576 |
| NT01MT1176 | 5 | 21156797; 16924483; 18429691; 18096847; 17163967; 12471504; 16131219; 11823455; 11781802; 9671511 |
| NT01MT1267 | 18 | 20656904; 19349423; 20067187; 19911131; 16661681; 16550463; 16286358; 1008746; 15642368; 15581616 |
| NT01MT1316 | 12 | 18755190; 18089836; 11726508; 15632183; 14569414; 11696367; 11306572; 1331806; 9920766; 9630516 |
| NT01MT1343 | 8 | 21268349; 21167817; 21135168; 21123941; 21112832; 21112296; 21111734; 21111046; 21082862; 21081474 |
| NT01MT1388 | 2 | 21221530; 19623929; 18388975; 17211544; 17033719; 16758158; 15979273; 12069193; 11006847; 16347868 |
| NT01MT1398 | 2 | 9665173; 9660189; 9331403; 9224567; 7606163; 7842859; 1731915; 8405386; 1451790; 1731915 |
| NT01MT1422 | 4 | 21036154; 20713661; 20643901; 20606066; 20547748; 20220788; 20091610; 20057163; 16641446; 18568851 |
| NT01MT14691 | 8 | 20938979; 20549541; 19593556; 18436321; 17520247; 17436647; 9973343; 16428816; 16075199; 15978081 |
| NT01MT1532 | 14 | 21302675; 21268919; 21248836; 21217689; 21217679; 21170047; 21199573; 21217678; 21137981; 21111108 |
| NT01MT1538 | 14 | 8621608; 8636106; 8310883; 3052277; 1460049; 2833197; 12980976 |
| NT01MT1766 | 12 | 21248859; 21078976; 20921378; 20833188; 20696460; 20667509; 20603082; 20421420; 20181951; 20180598 |
| NT01MT1798 | 15 | 21183667; 20442958; 19926656; 19456862; 11831459; 8051064; 9466913; 17726022; 15299374; 10910347 |
| NT01MT18691 | 2 | 18311927; 17174261; 15614489; 9188462; 2551298; 2474283; 6311826 |
| NT01NE0120 | 8 | 21380436; 21058669; 20666367; 20629638; 20606266; 20572664; 20472787; 20460700; 20422435; 20421652 |
| NT01NE0144 | 18 | 21304833; 20952578; 12563288; 20585060; 20418143; 20335169; 20147287; 15347752; 20095157; 20008538 |
| NT01NE0304 | 8 | 21178169; 20715760; 20388716; 20359200; 20154126; 19764800; 19697907; 18838782; 18757819; 18282009 |
| NT01NE0393 | 18 | 21276097; 10209752; 4196588; 17927700; 17628143; 17578453; 17268821; 17238922; 11967085; 15993072 |
| NT01NE0394 | 18 | 21354401; 21338516; 21329359; 21278293; 21276097; 21190440; 21189348; 21183643; 21179522; 21157514 |
| NT01NE0509 | 18 | 20805402; 20410291; 20061477; 19036729; 5323018; 17644517; 16756484; 10574995; 15980069; 10637639 |
| NT01NE0555 | 18 | 21281657; 21248166; 21161089; 21131297; 21123949; 21097500; 21072379; 21038480; 20956561; 20943816 |
| NT01NE0598 | 15 | 21368655; 21326386; 21245135; 21217772; 21122287; 21102427; 21078560; 21071440; 21070208; 21062507 |
| NT01NE0617 | 15 | 20522491; 20127467; 19919539; 19695263; 8529885; 7984417; 19220743; 11207743; 17208514; 15818467 |
| NT01NE0693 | 18 | 17370038; 10485884; 10700278; 12823822; 12700102; 10203757; 8387997; 9383187; 8144489; 7746157 |
| NT01NE0988 | 12 | 20828565; 17415567; 17267443; 11533066; 9882712; 9187245 |
| NT01NE1093 | 18 | 21151985; 20700710; 20687538; 20656779; 20600125; 20595378; 20503933; 20443931; 20369870; 20298244 |
| NT01NE1180 | 18 | 16581203; 15980069; 9755155; 8226679; 2492105; 1629152; 9368353; 9026446; 7565085 |
| NT01NE1199 | 18 | 15362847; 6768753; 9890828; 1411544; 9353297; 8939430; 3301821; 7504166; 3056908; 2266560 |
| NT01NE1303 | 18 | 20047910; 16101996; 3045756; 19397675; 363519; 15733922; 12029043; 15184553; 9457855; 11795869 |
| NT01NE1435 | 4 | 11322821; 7020540; 8753818; 8645296; 7494469; 6383412 |
| NT01NE1463 | 9 | 17564601; 12382061; 11762602; 10322030; 9204888; 16534981; 8869883 |
| NT01NE1477 | 14 | 21270255; 21123655; 21084430; 21081066; 21078494; 21076097; 21057854; 21053139; 21042731; 21041655 |
| NT01NE1512 | 15 | 20473251; 21178681; 19473555; 18566962; 18191220; 17916333; 17901711; 14557065; 14574675; 15211587 |
| NT01NE1602 | 5 | 20942799; 20302570; 20061482; 19864422; 18600049; 17941825; 17244611; 16877383; 16730357; 16298366 |
| NT01NE1827 | 9 | 20818586; 20704262; 20557983; 20099411; 19664929; 18569341; 17904577; 16275103; 15985814; 10605112 |
| NT01NE1931 | 3 | 20331639; 20145101; 17943123; 17322192; 15978078; 17121595; 16390451; 16390436; 15853882 |
| NT01NE1962 | 8 | 21061628; 20858453; 20823090; 20654728; 20643099; 20636270; 20625049; 20613764; 20603809; 20460714 |
| NT01NE1969 | 12 | 21037563; 20965178; 20885977; 20811445; 20802067; 20798673; 20636378; 20634352; 20602853; 20588025 |
| NT01NE2031 | 2 | 20970745; 18511939; 19835359; 17401144; 17168897; 16914555; 10331874; 16464106; 16343413; 16248620 |
| NT01NE2142 | 8 | 21320626; 21270901; 21258134; 21241708; 21216912; 21188974; 21117169; 21104367; 20962922; 20868748 |
| NT01NE2455 | 6 | 21290544; 21225600; 21165677; 21134127; 20933505; 20878079; 20869260; 20686522; 20677140; 20561586 |
| NT01NE2507 | 14 | 21394564; 21382339; 21377207; 21376373; 21371473; 21362453; 21360614; 21324980; 21311889; 21273417 |
| NT01NE25882 | 4 | 21275844; 20610843; 20378690; 20378007; 20335172; 20306272; 19842166; 19506095; 19424632; 10900165 |
| NT01NO00313 | 8 | 16466742; 16233244; 11532013; 11265475; 9651254; 9195883; 9000337; 8880936; 8593103; 1314088 |
| NT01NO0036 | 6 | 21144832; 21097613; 20156448; 18703019; 18206906; 8663104; 16677303; 11839499; 1718867; 8308039 |
| NT01NO0092 | 11 | 21394508; 21332975; 21277606; 21262357; 21248857; 21126577; 21117966; 21078967; 21058743; 20975940 |
| NT01NO0105 | 8 | 21335380; 20738406; 18820069; 6336730; 19941855; 19716429; 19507198; 18281432; 10896219; 17827302 |
| NT01NO0150 | 3 | 17581122; 14960717; 18599841; 15753075; 16968224; 16773281; 16556217; 16403447; 15101991; 12040098 |
| NT01NO0286 | 3 | 20870617; 20547216; 20304968; 20153338; 19953637; 19906359; 19462515; 9122246; 19013508; 18389628 |
| NT01NO0308 | 12 | 21369596; 21233208; 21148414; 21059845; 21045752; 20977881; 20969505; 20949043; 20889835; 20880591 |
| NT01NO03380 | 4 | 15819627; 15735307; 12791143; 12777805; 10931298 |
| NT01NO0368 | 18 | 21394206; 21390501; 21375693; 21352228; 21301927; 21256965; 21247063; 21214543; 21196474; 21143798 |
| NT01NO03755 | 8 | 21365653; 21311030; 21287365; 21220760; 21080078; 21049565; 20616380; 20545877; 20456047; 20424165 |
| NT01NO0441 | 12 | 19116772; 17591721; 17464060; 10684935; 9150870; 14651272; 12751333; 12741815; 7984417; 11830650 |
| NT01NO0577 | 4 | 20445251; 11430835; 19273182; 17498620; 16376524; 16014621; 11157955; 12164807; 10220403; 9929392 |
| NT01NO0611 | 15 | 8021206; 10648533; 9404056; 8693026; 7545961; 8029329; 8349566; 5432063 |
| NT01NO0666 | 18 | 17373356; 11222598; 15936994; 11567022; 11567022; 10866802 |
| NT01NO0670 | 4 | 19088431; 19002379; 18759246; 18198190; 17537991; 16815060; 16568970; 10980198; 16297653; 16253994 |
| NT01NO0671 | 4 | 21385732; 21320519; 21193388; 20971157; 20949432; 20949431; 20887743; 20571777; 20547570; 20423714 |
| NT01NO0920 | 9 | 20485265; 16838328; 11960029; 16544270; 12514242; 10373459; 8858564; 7968268; 1323035; 4608978 |
| NT01NO0933 | 9 | 19309082; 19280690; 18565285; 18274777; 18093984; 16722653; 16643907; 15967411; 15371447; 14684903 |
| NT01NO1010 | 2 | 21388209; 21322476; 20727354; 20606269; 20377263; 20231415; 20219675; 19411418; 19968274; 19816718 |
| NT01NO1153 | 18 | 21303978; 21124051; 20601097; 8702982; 20041341; 20041226; 20034571; 1878971; 19524038; 18511120 |
| NT01NO11770 | 4 | 18589814; 17552251; 7984417; 16469465; 16283251; 16156114; 10552044; 15378927; 15214638; 10202137 |
| NT01NO1434 | 8 | 21144840; 21081705; 21071633; 21042727; 21042417; 20960122; 20931558; 20876714; 20835910; 20826705 |
| NT01NO1443 | 13 | 21174459; 21093336; 21196765; 20833871; 20562876; 20510667; 20233922; 16530367; 17805690; 16061186 |
| NT01NO14450 | 8 | 21253866; 21036145; 10672375; 20160912; 20022530; 9324032; 17667915; 17635929; 8798399; 16293764 |
| NT01NO1452 | 8 | 21362551; 21243721; 21086481; 20978746; 20527922; 17349698; 20376845; 20227379; 20196537; 20190091 |
| NT01NO1503 | 11 | 20547379; 10722135; 10986230; 10320570; 9402344 |
| NT01NO15137 | 8 | 14596832; 12904299; 10518613; 10786631; 9673017; 9439692; 9127192; 8605214 |
| NT01NO1588 | 12 | 20869368; 20811692; 20633229; 20472561; 20395367; 20305065; 18515342; 20170747; 14502281; 20027625 |
| NT01NO1603 | 12 | 21392585; 21392420; 21390281; 21383917; 21383168; 21379573; 21373966; 21372483; 21342558; 21368225 |
| NT01NO1682 | 6 | 18035357; 7559580; 14508492; 9382804; 1610820; 8939893; 2334423; 3816761 |
| NT01NO1716 | 18 | 20572666; 20167620; 19130255; 2680975; 18535817; 10417152; 7984417; 9872997; 10203757; 12712204 |
| NT01NO1774 | 4 | 21388209; 21371503; 21346125; 21339943; 21322476; 21314617; 21239558; 21142169; 21085199; 21063867 |
| NT01NO1834 | 6 | 21248859; 21060849; 21034533; 20939910; 20727894; 20665498; 20533284; 20495087; 20495086; 20305446 |
| NT01NO1925 | 3 | 16000707; 15838638; 12823812; 11345521; 1955873; 9224882; 9224878; 9224872 |
| NT01NO1977 | 18 | 21349974; 21295952; 21056572; 21028901; 20553812; 20382203; 20363295; 20138126; 20026386; 19719512 |
| NT01NO20780 | 4 | 20190087; 19923715; 19574215; 19402045; 19110079; 18216065; 17313403; 17131148; 12868606; 16919403 |
| NT01NO21277 | 8 | 21165740; 21036156; 20879959; 20702080; 20692227; 20458520; 20384299; 20215780; 20190030; 20136404 |
| NT01NO2159 | 3 | 21338823; 21302368; 21144870; 21077836; 20420912; 20410612; 20144728; 19252306; 19155071; 18992255 |
| NT01NO2166 | 11 | 20689316; 20444095; 20034649; 19822340; 19493107; 18444855; 19352408; 14763988; 9367129; 10493868 |
| NT01NO2181 | 12 | 20978129; 20640600; 17028019; 19887081; 19477266; 19403345; 16446060; 18840531; 15617991; 18214855 |
| NT01NO2225 | 15 | 20403806; 20127467; 19664078; 19427285; 19381785; 19022560; 18815136; 18700747; 9349713; 17637670 |
| NT01NO2244 | 12 | 21247409; 21238933; 21098233; 21072173; 20961427; 20960971; 20950468; 20861008; 20852886; 20740538 |
| NT01NO2354 | 2 | 21092138; 20832326; 20817265; 20722615; 20711197; 20552672; 20539033; 20450493; 20393702; 20298756 |
| NT01NO2415 | 4 | 21388656; 21262281; 21155953; 20979388; 20718603; 20631438; 20624418; 20187044; 19923859; 19921512 |
| NT01NO2418 | 2 | 20693992; 11159333; 10494632; 11255013; 8969526; 1445232; 2110099 |
| NT01NO24300 | 8 | 20826660; 20160912; 19586787; 18164639; 17923481; 7569993; 9560191; 17157320; 14558820; 16219034 |
| NT01NO24941 | 8 | 20211750; 19906649; 19754426; 19652996; 19445597; 17452322; 18445036; 17253981; 10511517; 4580564 |
| NT01NO2558 | 13 | 21167168; 21149565; 21147988; 21139582; 21124459; 21098120; 21098037; 21074289; 21068376; 21056981 |
| NT01NO25595 | 2 | 21047120; 20959560; 20692224; 20458544; 18849445; 19491146; 19234759; 10075836; 18215430; 17375528 |
| NT01NO2569 | 18 | 21231964; 1498688; 15989245; 15183890; 14629018; 10224004; 1324906; 4265021; 1741624 |
| NT01NO2579 | 6 | 16908062; 11319927; 1484492; 9636374; 2156730; 1579485; 7669353; 2126155; 8246844; 8380408 |
| NT01NO2604 | 8 | 21196320; 21122809; 20869947; 20624914; 20153716; 20083086; 19805544; 19609757; 19368359; 7589436 |
| NT01NO2635 | 5 | 21075841; 20473684; 20022138; 15579666; 17600048; 19746730; 19744922; 19678707; 19548980; 19372431 |
| NT01NO2707 | 4 | 19889085; 19665005; 12631703; 16677309; 15170399; 12787361; 10712687; 2407720; 8415608 |
| NT01NO2737 | 9 | 21392585; 21173190; 20876112; 20802159; 20676045; 20561744; 20551664; 20537980; 20528790; 20522803 |
| NT01NO2762 | 4 | 7248267; 8300518; 15723539; 15301551; 15147198; 11329284; 10833393; 10600124; 10591845; 8537385 |
| NT01NO2768 | 15 | 19665595; 19438267; 17209564; 6782445; 11092928; 16672595; 16489832; 15809007; 9694845; 3709526 |
| NT01NO2785 | 1 | 11708855; 16881711; 11259585; 18398873; 18266921; 17680699; 11119723; 17261589; 10393538; 7108955 |
| NT01NO2836 | 6 | 21334452; 21075923; 20979348; 20966096; 20936276; 20889748; 20824060; 20798056; 20713450; 20711849 |
| NT01NO2901 | 4 | 19445953; 19400780; 17355860; 17140412; 17114934; 15993073; 15381417; 10964570; 10393967; 10998168 |
| NT01NO2946 | 12 | 19574214; 10569943; 17293877; 18515364; 18156635; 16619500; 16411772; 15907807; 15894523; 15449704 |
| NT01NO2955 | 12 | 20599668; 19853572; 19804735; 16307111; 17766116; 18387365; 18339324; 17980516; 16834335; 16573693 |
| NT01NO2978 | 1 | 21393430; 21369939; 21334869; 21324981; 21315854; 21308383; 21298199; 21297899; 21297164; 21296133 |
| NT01NO3097 | 1 | 20379751; 20039042; 19955263; 19383689; 17482430; 17398170; 16667839; 8904338; 16907731; 9531508 |
| NT01NO3190 | 18 | 16272399; 11916384; 11361076; 11361074; 11065368; 10589728; 1662180; 9973552; 6434522 |
| NT01NO3385 | 6 | 21254160; 21242961; 21230005; 21211013; 21171995; 21170031; 21165677; 21146499; 21139978; 21138684 |
| NT01NO3457 | 2 | 20051244; 19409940; 19103164; 18071268; 16842733; 15215101; 15809342; 15196010; 14617622; 12479413 |
| NT01NO34981 | 8 | 20511298; 18346472; 15210349; 12909015; 11900549; 11563694; 3011794; 8145647 |
| NT01OY0063 | 17 | 20624215; 18631157; 17090527; 17073752; 15469519; 10436920; 12750380; 12535070; 2661828; 11036081 |
| NT01OY0068 | 12 | 19716818; 18572881; 16249335; 17258768; 17518418; 17506728; 16638632; 16133200; 15037248; 12962509 |
| NT01OY0192 | 13 | 21142053; 20615421; 20608977; 20575532; 20227365; 20106954; 20051305; 19000309; 19664587; 19481523 |
| NT01OY0196 | 15 | 20643148; 18028310; 16762025; 16359338; 16143847; 15817794; 15667297; 10648524; 14668330; 11238986 |
| NT01OY0330 | 13 | 15215462; 10093218; 11976723; 9888802; 9843401; 8373778 |
| NT01OY0910 | 12 | 21113465; 21098295; 20937828; 20600949; 20478262; 20431057; 20195883; 20074030; 20030246; 19812900 |
| NT01OY10034 | 8 | 19783787; 19398462; 18555809; 12236604; 17056619; 2535528; 12429703; 14871663; 14740209; 12691750 |
| NT01PF0067 | 6 | 21394101; 21393072; 21392397; 21391904; 21390132; 21389894; 21389547; 21389348; 21389131; 21388532 |
| NT01PF0118 | 15 | 21383147; 21382978; 21364973; 21325103; 21324151; 21293364; 21244481; 21242318; 21239381; 21236675 |
| NT01PF05001 | 8 | 19625389; 18615662; 18187645; 2120234; 16917793; 14526081; 12536257; 12351228; 11330713; 10906347 |
| NT01PF05351 | 8 | 21384922; 21280569; 21105189; 20884367; 20683217; 20466043; 20456617; 20377688; 19949818; 19924017 |
| NT01PF0611 | 12 | 11591154; 9660070; 9311569; 7539759; 2534273; 6330082; 6325298; 4713039 |
| NT01PF06711 | 2 | 18028398; 17174261; 15967475; 15614489; 11527707; 10891066; 10383756; 10075431; 9700068; 8885414 |
| NT01PF0687 | 14 | 21311411; 21295473; 21139397; 21076373; 21070810; 21062060; 21040798; 21035731; 20934342; 20848821 |
| NT01PF1105 | 8 | 19943898; 19368556; 16962969; 16343420; 11823455; 10357231; 12605255; 12603323; 11334785; 11181712 |
| NT01PF1509 | 3 | 21068326; 20675457; 20618868; 20517106; 20492281; 20367178; 20163284; 20124438; 20113187; 19929210 |
| NT01PF1889 | 6 | 19255482; 17158792; 9501915; 12527306; 11976507; 11021967; 10925204; 1985924; 10089444; 9862976 |
| NT01PF1908 | 18 | 18470695; 17875647; 17587231; 16135241; 11080142; 15158466; 11473129; 12454482; 12426307; 11453995 |
| NT01PF1940 | 8 | 20652669; 20030377; 20022118; 19616009; 16924483; 3026449; 12859215; 19374909; 19368556; 2149743 |
| NT01PF2004 | 6 | 21392201; 21270896; 21148291; 20962039; 20864039; 20512977; 20351259; 10757782; 20106979; 20070258 |
| NT01PF2170 | 11 | 21255388; 21241813; 20563849; 16754612; 19788700; 8870269; 18191436; 17929059; 17191617; 16854536 |
| NT01PM0093 | 4 | 20702416; 20400549; 19608423; 16894341; 7982914; 10564520; 15657292; 112060; 16262792 |
| NT01PM0094 | 3 | 21299650; 20498260; 20145101; 20086145; 19151141; 4041428; 18485865; 18400183; 8820654; 18179420 |
| NT01PM0171 | 18 | 20795716; 20707314; 20471962; 1429629; 19562781; 12480818; 18983849; 15728723 |
| NT01PM0273 | 18 | 16616613; 11573939; 2005823; 10784039; 8918249; 1447208 |
| NT01PM0355 | 3 | 845124; 16339737; 1592809; 10564478; 16870450; 10542235; 16316661; 9811812; 7810996; 9791115 |
| NT01PM0445 | 8 | 21167813; 10652088; 12679550; 12605683; 11929547; 11372198; 9546395; 9808754; 9756865; 9579062 |
| NT01PM0472 | 15 | 20351136; 20000779; 18188553; 15937186; 19018589; 18194341; 11375146; 16949866; 9790834; 15576792 |
| NT01PM0528 | 9 | 19685209; 18093984; 15987908; 11544213; 11969223; 11969222; 11792456; 2125185; 11351423; 8805338 |
| NT01PM0645 | 18 | 17306760; 10377394; 12627883; 12582158; 12427139; 10201006; 12133831; 12098491; 11165372; 10825438 |
| NT01PM0657 | 4 | 21351100; 21315634; 21314230; 21297617; 21229611; 21167305; 21062913; 21042727; 21034966; 20954747 |
| NT01PM0897 | 18 | 21047787; 20878786; 20124224; 20088382; 20023402; 20103413; 11077983; 7589520; 19291859; 19272442 |
| NT01PM0939 | 6 | 21075923; 21059643; 20833130; 20798056; 20722736; 20659289; 20639326; 20639321; 20444106; 20207760 |
| NT01PM0943 | 18 | 11139621; 12436256; 11238974; 10537210; 190207; 7651347; 18613047; 1920454; 2832233 |
| NT01PM1037 | 13 | 21232144; 21126336; 20942800; 20857816; 20849936; 20729713; 20627873; 20600146; 20591428; 20531247 |
| NT01PM1045 | 4 | 20870796; 20418396; 15884659; 11001536; 10650212; 9197418; 7853390; 8050069; 2065649; 1372278 |
| NT01PM1052 | 8 | 18673073; 9987136; 10436930; 8405944; 3138232; 1685007; 1851043; 1689724; 3138232; 2833503 |
| NT01PM1061 | 14 | 21178309; 19630523; 19220460; 17892463; 3288626; 8798731; 2986688 |
| NT01PM1125 | 18 | 19922539; 10532383; 11478968; 11320129; 11162395; 8386371; 6159552; 9822819; 8936592; 8621093 |
| NT01PM1176 | 6 | 21280122; 21117234; 21097626; 20951079; 20395367; 1547773; 20118249; 2988787; 19443546; 19332822 |
| NT01PM1234 | 3 | 21178073; 20688825; 8386125; 19211098; 18833547; 17880933; 14659681; 8943265; 16559160 |
| NT01PM13074 | 8 | 21209026; 18037139; 16452068; 12721472; 9445479; 10230063; 17753861; 1647035; 9445479; 10330464 |
| NT01PM1320 | 3 | 20093260; 16339737; 9797206; 18251277; 15205448; 17709535; 7608095; 10770780; 17181066; 8606187 |
| NT01PM1493 | 13 | 21152561; 10937989; 16076220; 2504932; 11279123; 10520458; 2199796; 9299515; 9281425; 8910435 |
| NT01PM15534 | 8 | 21390580; 21382014; 21362116; 21354789; 21255309; 21244065; 21222321; 21213964; 21183322; 21082355 |
| NT01PM1597 | 8 | 21368147; 21366233; 21354629; 21351540; 21327447; 21311030; 21278125; 21257750; 21242662; 21223993 |
| NT01PM1767 | 8 | 21204849; 20866109; 20629101; 19519368; 18808312; 18510554; 18456264; 18310271; 17975734; 17559573 |
| NT01PM1872 | 17 | 20844231; 20817775; 20810777; 20802081; 20688949; 2762315; 20644308; 20631094; 20562828; 20470829 |
| NT01PM1963 | 6 | 21360615; 21276096; 21154877; 20979543; 20922738; 20851546; 20723756; 20722738; 20659894; 20603075 |
| NT01PM2097 | 4 | 11320127; 18193310; 10086843; 1328156; 8529897; 1427009; 387720; 1311113; 1779760; 942051 |
| NT01PM2109 | 4 | 20042621; 15155722; 9379906; 9143881; 2547375 |
| NT01PM2155 | 3 | 20433200; 20400541; 19604481; 3277952; 11173485; 15572779; 19121323; 18675810; 9829962; 11118459 |
| NT01RX0302 | 9 | 20656373; 20338739; 20030724; 19940943; 19708881; 16484204; 4374474; 17010159; 16882035; 11251836 |
| NT01RX0398 | 18 | 20952391; 20826817; 20552428; 20394729; 20132828; 19818021; 19400778; 19362561; 19236039; 19234723 |
| NT01RX0586 | 8 | 21296661; 21073854; 20889786; 20809899; 20807998; 20736169; 20711239; 20676631; 20552355; 20516620 |
| NT01RX1254 | 17 | 19923733; 19833764; 19737356; 19699748; 19467815; 19364128; 17661085; 18639684; 18564909; 18331403 |
| NT01RX1328 | 17 | 21381923; 21296950; 21277915; 21264304; 21193605; 21189325; 21088825; 20971916; 20935101; 20889779 |
| NT01RX1416 | 12 | 20441706; 10428928; 2377622; 17709535; 17235642; 16840781; 10223944; 8830714; 9537382; 10223944 |
| NT01RX1418 | 4 | 10222208; 15769466; 11744713; 10417653; 11298275 |
| NT01RX15110 | 4 | 21147767; 21037003; 20971899; 20870400; 20817765; 20802044; 20735480; 20709900; 20643850; 10610760 |
| NT01RX1541 | 13 | 18241796; 20472640; 20007320; 18355719; 15766524; 11882645; 11313137; 1569565; 2207153 |
| NT01RX1658 | 18 | 20719963; 16687400; 3114241; 10380629; 2459118; 6989828; 6985610 |
| NT01RX18897 | 8 | 21247899; 20965146; 20823522; 20639576; 20627725; 20596899; 20512995; 20506508; 20219403; 20116224 |
| NT01RX1892 | 8 | 20545743; 10610758; 17884091; 16645310; 16553855; 16313613; 14704707; 15289572; 15259271; 9852007 |
| NT01RX2024 | 8 | 21384803; 21286609; 21277994; 21268349; 21257536; 21159850; 21172326; 21167817; 21252179; 21144838 |
| NT01RX2131 | 4 | 20659898; 16493696; 16475788; 1846859; 11274097; 3606092; 10877767; 7836301; 8358148; 8328812 |
| NT01RX2214 | 12 | 18093586; 10531249; 16325969; 9545337; 15377669; 12972178; 12919480; 11526112; 11390277; 11208991 |
| NT01RX2226 | 9 | 21347706; 21327327; 21134903; 21235489; 21216135; 21146433; 21258118; 20966403; 20956318; 20924576 |
| NT01RX22591 | 2 | 21367973; 21342411; 21335095; 21315197; 21314613; 21288652; 21268892; 21247799; 21217003; 21214021 |
| NT01RX2288 | 5 | 20816204; 20629619; 20479257; 11533491; 2824565; 17460664; 15905855; 16139897 |
| NT01RX2386 | 8 | 8111038; 15656983; 11343129; 8486646; 10684655; 2747617 |
| NT01RX2429 | 8 | 17288562; 17213670; 5309907; 15206762; 15110088; 8541664; 12042425; 10706721 |
| NT01RX2501 | 12 | 20924840; 20414836; 18794334; 19476106; 16314466; 10606526; 16844688; 15976328; 15277786; 14625392 |
| NT01RX2705 | 12 | 20829064; 20819954; 20610394; 19744480; 19734688; 19074976; 19074975; 18550540; 18629030; 17916556 |
| NT01RX32841 | 8 | 20528923; 20345279; 20000684; 17661163; 17714437; 18420422; 17881357; 16632253; 17235685; 17202864 |
| NT01SS00584 | 8 | 21188217; 20971492; 20675451; 17692376; 20606696; 20535664; 8985182; 9194558; 20118231; 20056699 |
| NT01SS0081 | 14 | 21331030; 21217676; 21127263; 21081091; 21080915; 21059655; 21059653; 21047945; 21042744; 21042412 |
| NT01SS0091 | 14 | 17277075; 11220413; 16891376; 16810197; 15488279; 8390989; 7768789; 8390989 |
| NT01SS01725 | 8 | 19923216; 12588873; 11355627; 12232386; 8360180; 8486290 |
| NT01SS0190 | 8 | 21278125; 21193741; 21080726; 15676284; 20512929; 20339378; 20107110; 19875674; 19786367; 19759337 |
| NT01SS0191 | 8 | 21383131; 21364629; 21209382; 21193741; 21099326; 21080726; 21037180; 20975997; 20880213; 20859605 |
| NT01SS0197 | 8 | 21383131; 21364629; 21326867; 21318295; 21305254; 21303534; 21278125; 21265734; 21252653; 21247400 |
| NT01SS02704 | 8 | 20700743; 20197075; 20164147; 20045480; 20043969; 19884781; 19576307; 19428473; 19217615; 19155269 |
| NT01SS0310 | 2 | 20937134; 20725044; 20690600; 20616867; 20443544; 20379951; 19385043; 19423627; 19631611; 1355089 |
| NT01SS03295 | 8 | 17346666; 16699922; 16223737; 15233792; 15009208; 14519128; 12941874; 9665709; 11773525; 11479380 |
| NT01SS0400 | 18 | 21232060; 21135102; 21118978; 21041493; 20978004; 20888343; 20876358; 20859232; 20856239; 20855510 |
| NT01SS0552 | 15 | 21288886; 20675451; 20538620; 19883076; 19879235; 16307111; 19542007; 19430763; 16804177; 18953669 |
| NT01SS0600 | 12 | 20919698; 20676041; 20404344; 19805579; 19965696; 19356587; 19234362; 19226252; 11387331; 16855395 |
| NT01SS0645 | 8 | 19464256; 18973745; 10471806; 11576546; 11022032; 8495728; 1847135; 3111850 |
| NT01SS0646 | 8 | 10471806; 12753925; 8495595; 1847135; 1689013; 2116128 |
| NT01SS0680 | 12 | 20453133; 19000036; 20097965; 20039128; 19269748; 19596340; 10742151; 16204545; 16076845; 19054328 |
| NT01SS0929 | 13 | 16971692; 11551941; 1879566; 2037044; 2747623; 2644249; 2578124 |
| NT01SS0932 | 17 | 19648323; 19441027; 19041906; 10196160; 10493887; 11111034; 10972822; 10828392; 10079080; 8557191 |
| NT01SS1016 | 1 | 12784220; 11173479; 10360352; 2199796; 8064862; 8170389; 1554351; 1648168 |
| NT01SS1146 | 4 | 21339840; 21335646; 21330151; 21321206; 21317324; 21296931; 21276094; 21257762; 21255117; 21244597 |
| NT01SS1438 | 6 | 20693529; 20656393; 20418439; 19866355; 16713563; 19150994; 9207015 |
| NT01SS1452 | 18 | 21349151; 21265577; 21227585; 21224233; 21088257; 20686922; 20592246; 18670623; 20472565; 20423300 |
| NT01SS1516 | 6 | 20977236; 20961081; 20828134; 20705454; 20696762; 20657578; 20614917; 20600091; 20532362; 20444223 |
| NT01SS1559 | 15 | 21391093; 21389307; 21388409; 21388403; 21383633; 21343252; 21329727; 21291921; 21291865 |
| NT01SS1745 | 13 | 4927947; 20507916; 18612675; 9242913; 17199978; 1638630; 15817456; 10369922; 10359080; 1915304 |
| NT01SS1804 | 15 | 21317330; 21282331; 21216748; 21193608; 21183574; 21153675; 20938678; 20798164; 20716687; 20708625 |
| NT01SS1915 | 8 | 9233812; 17639348; 11751810; 3067084; 6855607; 3089314 |
| NT01SS2126 | 17 | 20833730; 18287027; 20460517; 20446108; 20397397; 20075562; 19951946; 12618438; 19660454 |
| NT01SS2524 | 12 | 21141460; 20964444; 20887733; 20873201; 20868232; 20851345; 20837023; 20802074; 20797683; 20637416 |
| NT01SS2569 | 9 | 15661733; 10733890; 9165098; 8300626; 1495410; 2116174; 6304089; 3900077; 6373752 |
| NT01SS27475 | 8 | 21367867; 21356599; 21311030; 21288886; 21152918; 21063907; 20960116; 20955682; 20946347; 20943284 |
| NT01SS28175 | 8 | 21365653; 21287365; 21220760; 21080078; 20616380; 20545877; 20424165; 20075906; 19734149; 19479952 |
| NT01SS3049 | 13 | 21282428; 21175197; 21144880; 21042822; 20954242; 19894713; 19847269; 19837083; 19772352; 16758500 |
| NT01SS3219 | 8 | 21224842; 20844280; 20552435; 20152184; 16026781; 18976680; 18926806; 2739735; 18406560; 17510468 |
| NT01SS3374 | 8 | 21389620; 21385584; 21382272; 21377525; 21375472; 21368419; 21365006; 21364950; 21362485; 21362081 |
| NT01SS3954 | 12 | 21210767; 20880327; 20727857; 10716932; 20460722; 20345652; 20345653; 20159564; 17262209; 20104973 |
| NT01ST0184 | 18 | 21192796; 20880425; 20594961; 19822890; 17517839; 10539994; 12824943; 12354237; 8746797; 1943705 |
| NT01ST0217 | 3 | 21299650; 20199591; 15196927; 17559395; 17378923; 8985185; 16573686; 8830709; 7909802 |
| NT01ST0465 | 3 | 7997169; 14769329; 14729737; 11062187; 2066344; 4208895 |
| NT01ST0547 | 8 | 18582433; 15910742; 14672950; 11017202; 11004462; 10544288; 10436930; 9737851; 9665692; 9548917 |
| NT01ST0587 | 18 | 15306729; 12034710; 12810987; 12441636; 7604262; 112915; 8196548; 7773394; 7711899 |
| NT01ST0941 | 18 | 20848015; 20460185; 19455513; 18396170; 12627969; 11421278; 11377046; 10409492; 10188600; 9325422 |
| NT01ST0987 | 18 | 17107946; 17074973; 16677337; 3137921; 6326592 |
| NT01ST1118 | 11 | 21327123; 21303688; 21285413; 21247655; 21241792; 21241420; 21204936; 21187415; 21129199; 20945083 |
| NT01ST1296 | 11 | 20303825; 19501196; 19493006; 10829079; 17601790; 18363236; 17526845; 17379730; 17355601; 16804187 |
| NT01ST1449 | 4 | 21393367; 21392132; 21391724; 21390252; 21390208; 21390206; 21388964; 21388880; 21386987; 21386978 |
| NT01ST1570 | 4 | 20544426; 18155882; 2291668; 8576363; 1406282; 7263566; 1654505; 6208484; 2998941; 6328217 |
| NT01ST1692 | 18 | 21357299; 21354366; 21271477; 21262925; 21254069; 21237168; 21210724; 21191769; 21182225; 21170509 |
| NT01ST1715 | 12 | 20179346; 20179356; 17581124; 15052633; 12724370; 9330676; 9004508; 8936307; 8229672; 1840314 |
| NT01ST18587 | 8 | 21377527; 21058172; 20943332; 20514433; 19053876; 18856213; 12185102; 18795862; 18432090; 18086667 |
| NT01ST1932 | 3 | 18852510; 18563901; 18322734; 18051363; 16968069; 16549662; 16421442; 16173464; 15784255; 1689153 |
| NT01ST1942 | 8 | 21167813; 16274230; 12409197; 12165429; 9546395; 9756865; 7746153; 1657982; 16345624 |
| NT01ST2201 | 18 | 20844539; 20696265; 20656864; 20624916; 15220473; 19682259; 19460820; 19410333; 15240890; 18475907 |
| NT01ST2226 | 8 | 7854413; 19928857; 17028282; 15569666; 11170458; 3067084; 2154378; 1558764; 1482271; 1849603 |
| NT01ST2420 | 18 | 9388509; 9815665; 7559654; 8402883; 7882987; 7883784; 15966102; 2836739 |
| NT01ST2464 | 4 | 20132451; 19732341; 8071222; 19383688; 8071222; 19332819; 17768253; 17542929; 12057936; 17067800 |
| NT01ST2473 | 15 | 21394488; 21394477; 21392134; 21391883; 21389270; 21389130; 21385826; 21385438; 21385035; 21383382 |
| NT01ST24920 | 4 | 17099062; 12670683; 12060973; 2496115; 10589727; 10460888; 7984109; 7954812; 7509821; 1961761 |
| NT01ST2517 | 14 | 20633228; 4874308; 942051; 18083805; 15939305; 12686640; 12101181; 11441022; 11148030; 9782487 |
| NT01ST2573 | 11 | 7790093; 15534984; 11207586; 10498940; 8406853; 9723926; 9364916 |
| NT01ST2822 | 12 | 20558234; 20180651; 19912053; 19809093; 19728173; 19396561; 10596841; 17944830; 17509894; 16895390 |
| NT01ST3050 | 8 | 21142024; 20578695; 20519496; 20516606; 19899795; 1312999; 19762342; 19553148; 8004173; 19194002 |
| NT01ST3218 | 11 | 21175897; 19788700; 18313399; 10087920; 17011731; 17257169; 7588618; 16473661; 8181061; 15883824 |
| NT01ST3460 | 18 | 20662775; 20581212; 20399272; 19891498; 19621966; 19333719; 11910013; 15255870; 18793330 |
| NT01ST3545 | 8 | 7890119; 14259761; 2200847; 2187144; 3075654 |
| NT01ST3556 | 8 | 7854413; 20542928; 19911220; 8034727; 17938909; 16756481; 18437310; 18182386; 17938909; 17574676 |
| NT01ST3938 | 5 | 21226054; 17618313; 9384533; 9398217; 9371250; 8999955; 7775463 |
| NT01ST4033 | 18 | 11916918; 17172526; 10348879; 16003938; 15661014; 15543948; 15528653; 12680778; 2973458; 12109883 |
| NT01ST4116 | 4 | 21390069; 21384160; 21369674; 21359956; 21352809; 21347706; 21336677; 21302003; 21301156; 21292024 |
| NT01ST4305 | 12 | 18604505; 17467832; 10972813; 8837412; 9668097; 9282737; 9224881; 8820654; 8817497; 7565106 |
| NT01ST4447 | 11 | 21248857 |
| NT01ST4630 | 8 | 19616102; 18071260; 17146529; 11318637; 7880831; 16559160; 8307963; 8343529; 1444446; 2104756 |
| NT01ST4957 | 2 | 21321939; 21131421; 21090173; 21048046; 21041703; 21037234; 20979658; 20972512; 20932248; 20925964 |
| NT01ST5044 | 4 | 21371264; 21357634; 21355843; 21354321; 21344118; 21334636; 21331057; 21311136; 21301875; 21300015 |
| NT01ST51591 | 2 | 20837009; 19923213; 19328201; 19166853; 19118370; 17969213; 17403671; 15271986; 14757766; 12650933 |
| NT01ST51601 | 2 | 19923213; 15456260; 17403671; 15914018; 15489164; 9987136; 15012138; 14757766; 14567704; 12650933 |
| NT01ST51621 | 2 | 21378167; 21368171; 21258042; 20444693; 19926634; 16518696; 17976513; 17403671; 17015169; 9843405 |
| NT01ST5267 | 8 | 21329464; 21294640; 21212946; 21206700; 21133623; 20878130; 20851451; 20593958; 20448207; 20200430 |
| NT01ST5369 | 13 | 9630213; 16406568; 7547908; 1333794; 3356685; 2833497 |
| NT01ST55302 | 4 | 17504272; 9237995; 12529317; 16135241; 12598694; 12426307; 9711857; 12123454; 11931562; 11191810 |
| NT01ST5537 | 15 | 19673543; 19583447; 15808504; 19124016; 18952097; 18062991; 18455186; 18186488; 5700707; 1312212 |
| NT01ST55667 | 8 | 21265764; 5432063; 17640279; 17485854; 17433574; 17137322; 17028190; 16233775; 10503078; 12604207 |
| NT01ST5600 | 12 | 19839645; 17966992; 17390395; 16289685; 15882050; 15265041; 15159592; 12946361; 12718528; 12297235 |
| NT01ST5687 | 15 | 20376102; 15937186; 18248418; 16154092; 11591683; 11703664; 1459461; 10517599; 10448677; 10089538 |
| NT01ST5689 | 4 | 20418396; 11001536; 10650212; 9197418; 7853390; 8050069; 2065649; 1372278; 1805505; 2835585 |
| NT01STA0044 | 4 | 15159578; 9829946; 11741897; 10873460; 10661868; 10358024; 9915794; 6390428; 1459960; 9426133 |
| NT01STA0130 | 4 | 21145048; 20977916; 20846734; 20815070; 20801606; 20655572; 20647941; 20592366; 20502493; 20146978 |
| NT01TD0057 | 12 | 21315388; 21255311; 21255307; 21247902; 21139195; 20853080; 20850500; 20846931; 20797615; 20615874 |
| NT01TD0096 | 4 | 21169496; 20851187; 20669241; 20544975; 20535505; 20363940; 20347402; 20061479; 19407375; 18980306 |
| NT01TD0139 | 15 | 20656781; 18793176; 20227482; 20028809; 10966457; 19237529; 19703104; 9126336; 18801989; 17997077 |
| NT01TD0147 | 18 | 10960117; 10473554; 9523453; 8991852; 8905098 |
| NT01TD0153 | 9 | 21347729; 21319141; 21288167; 21253391; 21052529; 20967558; 20503257; 20397731; 20009024; 19834870 |
| NT01TD0319 | 3 | 21394312; 21388311; 21385202; 21383969; 21378187; 21371037; 21342541; 21367879; 21366639; 21365910 |
| NT01TD03737 | 8 | 21186173; 20557574; 20227379; 20202167; 16880; 19825618; 19705487; 15685292; 19505081; 2611230 |
| NT01TD0516 | 12 | 20713620; 20679205; 20667621; 19729222; 19696109; 19553530; 19271156; 19220794; 18996356; 18835234 |
| NT01TD0570 | 8 | 16450109; 15491156; 12799376; 12427946; 12196025; 10830505; 10571051; 9748316; 9623801; 9480821 |
| NT01TD0667 | 12 | 21343297; 21330387; 21146536; 21107306; 21063220; 21047126; 21034747; 20960817; 20927641; 20850379 |
| NT01TD0673 | 1 | 21370850; 21305278; 21124060; 21076101; 21073196; 20821425; 20814792; 20627897; 20598554; 20504042 |
| NT01TD0688 | 12 | 21342558; 21368225; 21364675; 21325529; 21306151; 21299646; 21292977; 21265178; 21264511; 21254625 |
| NT01TD0762 | 6 | 2549254; 11733530; 7994576; 8395022; 2541877 |
| NT01TD08222 | 2 | 20926389; 19647806; 19270703; 18421771; 16954203; 15748981; 15581577; 12777395; 12771147; 12547821 |
| NT01TD0885 | 8 | 15338111; 15316720; 9254694; 11889481; 11820782; 10548510; 9729445; 9467914; 9425311; 9022686 |
| NT01TD0892 | 14 | 20457752; 15670165; 9333323; 12796482; 12130656; 11953431; 10966576; 9636022; 8548458; 8360919 |
| NT01TD0898 | 8 | 6355817; 12198487; 17161368; 16987314; 16866348; 15893657; 12729720; 12054871; 10591533; 9535897 |
| NT01TD1132 | 6 | 21212936; 21156049; 21148559; 21103969; 21091508; 21081109; 21040688; 20979388; 20977453; 20960970 |
| NT01TD12251 | 8 | 17497738; 16351070; 15101557; 12731863; 10861407; 10569192; 10491105; 10394618; 10200442; 7813438 |
| NT01TD1332 | 3 | 20305657; 20234135; 19825597; 14221104; 19224571; 18402606; 17768255; 17640872; 17593303; 17186209 |
| NT01TD1393 | 15 | 21383689; 21369825; 21364145; 21311100; 21307942; 21255110; 21236481; 21233348; 21223508; 21220510 |
| NT01TD1419 | 18 | 17169333; 15667293; 10913697; 9037773; 9022686; 1328332; 2860102 |
| NT01TD1519 | 18 | 21266546; 20519548; 19887446; 19619244; 18701882; 10559939; 16563799; 16169010; 10200558; 15803409 |
| NT01TD1567 | 6 | 21124948; 20861182; 20813592; 20666462; 20628184; 20334433; 20223211; 8662184; 20122408; 19931513 |
| NT01TD1585 | 12 | 20187257; 19824885; 19415426; 19685394; 9847218; 17428367; 14871029; 11927288; 11749899; 11603724 |
| NT01TD1712 | 6 | 21296952; 21257772; 21252276; 21192970; 21142182; 21123191; 20842173; 20798162; 20660743; 20659290 |
| NT01TD1809 | 8 | 15389597; 19115036; 17294170; 16027951; 14705036; 7826011; 14638414; 12855725; 11566129; 2971647 |
| NT01TD1871 | 8 | 21332923; 21321312; 21303841; 21276451; 21270637; 21178169; 21116622; 21105682; 21031026; 20959106 |
| NT01TD18815 | 2 | 16423549; 15182178; 11106484; 10966642; 9865610; 8618906; 9496786; 7743933; 8682201; 8590013 |
| NT01TD2074 | 8 | 21142117; 17034788; 16500161; 8033912; 15544340; 12634336; 12500194; 9287422; 11724568; 11704672 |
| NT01TD2105 | 18 | 21369877; 21351733; 21348301; 21321244; 21317882; 21310991; 21311022; 21295539; 21292979; 21170894 |
| NT01TD2131 | 14 | 21385868; 21323311; 21299470; 21275844; 21235502; 21222452; 21210868; 21194355; 21190518; 21094149 |
| NT01TD2134 | 15 | 2965141; 10792719; 16348476; 9387225; 8052622; 8086465; 8188579; 8412698; 8312973 |
| NT01TD2135 | 15 | 21245315; 21233159; 20971899; 20676709; 10610760; 18079742; 20232248; 20184896; 20025668; 20025669 |
| NT01TD2159 | 8 | 15047692; 2033048; 1069307; 18422485; 1310666; 15047692; 12637552 |
| NT01TD2179 | 3 | 20589904; 20512975; 18690721; 15238632; 16595672; 16091590; 15865428; 10231382; 15236574; 14725765 |
| NT01TD2189 | 4 | 21283549; 20719048; 20683135; 17900376; 16104019; 15081878; 2615761; 10640454; 6311812; 8987785 |
| NT01TD2229 | 12 | 21394906; 21393803; 21393736; 21393710; 21393674; 21393542; 21392254; 21389503; 21389402; 21389399 |
| NT01TD2280 | 15 | 21346797; 21304599; 21242066; 21229249; 21186242; 21078995; 21039781; 20876533; 20870771; 20814030 |
| NT01TD2313 | 18 | 19708689; 3054465; 18946633; 18083710; 19636864; 17997964; 10474183; 17609129; 17349657; 7642501 |
| NT01TD2577 | 3 | 19754149; 7961456; 16755996; 11679076; 10092655; 1732206; 10092655; 9197543; 9099672; 8662613 |
| NT01TD2585 | 4 | 17425668; 16390454; 16203150; 15383161; 14678166; 12209001; 10582867; 9837987; 10188253 |
| NT01TD26091 | 2 | 18028398; 11527707; 10075431; 9700068; 8885414; 791939; 14217462; 2540841 |
| NT01TD2649 | 18 | 21261644; 21187407; 21116916; 21063106; 21063093; 21057059; 21057037; 21055323; 21052084; 21046154 |
| NT01TD2745 | 15 | 19452262; 10411758; 1787796; 10347192; 8830681; 1391046; 1547494 |
| NT01TD2812 | 9 | 21370994; 21309865; 21199673; 21135931; 21113689; 21081696; 20943853; 20925342; 20662933; 20594840 |
| NT01TE0003 | 12 | 20959450; 20581826; 20581825; 20515644; 20487295; 20487271; 20437142; 20188538; 20184896; 20060910 |
| NT01TE0176 | 11 | 21156198; 20890269; 20691900; 20547379; 20303825; 1655708; 12169598; 19749446; 20948648; 18790866 |
| NT01TE0532 | 12 | 12900386; 20487019; 20363934; 17259602; 19556347; 18650432; 18412550; 9603870; 17965014; 2448968 |
| NT01TE0594 | 6 | 21283549; 20213113; 18248456; 11111029; 2615761; 11816970; 8604133 |
| NT01TE0608 | 8 | 21375368; 21362121; 21350313; 21335435; 21331250; 21316707; 21312041; 21253566; 21221938; 21171012 |
| NT01TE0707 | 3 | 1649817; 15063203; 10801168; 9235953; 5699064 |
| NT01TE0729 | 13 | 10196363; 15103084; 16712869; 15772074; 15581587; 14501142; 12736709; 9829824 |
| NT01TE0803 | 15 | 21392507; 21378195; 21364186; 21324192; 21317554; 21304819; 21303721; 21252099; 21247892; 21237251 |
| NT01TE0812 | 8 | 21315823; 21061628; 20875067; 20837795; 20676915; 20627642; 20625049; 20613764; 3934395; 20435583 |
| NT01TE0813 | 8 | 21387033; 21251101; 21152918; 21112414; 21099326; 21042417; 20958226; 20858453; 20837795; 20833784 |
| NT01TE0896 | 8 | 19928586; 18655808; 19366613; 18656488; 18593701; 18543768; 7690960; 17984565; 17971335; 17901903 |
| NT01TE0915 | 4 | 20929858; 20682242; 20620191; 20078128; 19797004; 19583492; 16060667; 18422870; 9341227; 2233312 |
| NT01TE1156 | 3 | 20851488; 19809250; 17785473; 15251431; 18177738; 17906127; 17620355; 407083; 10781617; 8530464 |
| NT01TE11765 | 8 | 20064486; 17912604; 15908597; 15358552; 12944966; 11287157; 1527503 |
| NT01TE13481 | 8 | 21267505; 20847005; 20724435; 20690587; 20525998; 20506248; 20435741; 20211750; 20035716; 19942659 |
| NT01TE1446 | 8 | 18930018; 18681943; 19356022; 17022624; 16688708; 16274230; 15035626; 14624544; 12937163; 9657676 |
| NT01TE1539 | 15 | 10966457; 19748334; 10673004; 12657056; 11844754; 11937029; 9813128; 10500846; 8969172; 9878429 |
| NT01TE1600 | 4 | 20655986; 1999397; 19895788; 18789896; 17712554; 15983415; 14676989; 3059995; 12076793; 12427935 |
| NT01TE1939 | 9 | 21272313; 15517390; 10737174; 9208946; 8621604; 8641446; 7920711; 8203167; 10976232; 5637434 |
| NT01TE21354 | 8 | 21206049; 20936136; 20622018; 20621155; 20607149; 20460730; 20384855; 9122158; 20096384; 20031435 |
| NT01TE2296 | 18 | 1763075; 11952125; 11682179; 11092250; 10214935; 16653217; 9133319; 6312838; 7108955; 3912654 |
| NT01TE2412 | 12 | 20466747; 17526840; 10320580; 16861792; 9735342; 9240461; 8954128; 7811295 |
| NT01TE2467 | 4 | 21390252; 21297164; 21296915; 21246731; 21187903; 21159796; 21136304; 21126336; 21109548; 21097705 |
| NT01TE26572 | 8 | 17505911; 8043589; 7925499; 8467079; 1901865 |
| NT01TE2676 | 9 | 21389045; 21388747; 21375498; 21371360; 21362034; 21345233; 21331774; 21330637; 21327327; 21315740 |
| NT01TE27800 | 4 | 20138126; 19826764; 19719512; 16861227; 15758237; 11683355; 9735283; 8709848 |
| NT01TV0203 | 4 | 21388418; 21314709; 21312355; 21310602; 21295988; 21269483; 21255371; 21252326; 21229377; 21228001 |
| NT01TV0253 | 8 | 21391663; 21385867; 21382109; 21355852; 21354257; 21352808; 21322032; 21320649; 21319188; 21318024 |
| NT01TV0343 | 8 | 17993624; 17981801; 15809865; 15666206; 9426612; 8830251; 8223576; 1688555 |
| NT01TV0378 | 13 | 20727857; 20429542; 20097853; 11478805; 10476961; 2103466; 409999; 4076177 |
| NT01TV0461 | 18 | 16002746; 15309927; 10700449; 12479229; 12213994; 11525232; 7534729; 1325893; 1656472; 6147184 |
| NT01TV0562 | 2 | 20627200; 3032913; 12093296; 11939774; 10758367; 14898026; 9697817; 8416664; 2278386 |
| NT01TV1025 | 6 | 21290819; 21118815; 21076032; 20972363; 20862319; 20851705; 20805875; 20795733; 12185851; 20527806 |
| NT01TV12035 | 8 | 20944748; 20882305; 17525962; 20573731; 10545161; 11985816; 18417195; 17968985; 17728351; 17659056 |
| NT01TV1258 | 4 | 20606534; 9922449; 19343071; 18776921; 10629059; 17592857; 16249118; 8939847; 12963835; 12059957 |
| NT01TV1276 | 6 | 21392732; 21388065; 21385603; 21380782; 21377274; 21377232; 21376043; 21375453; 21371741; 21370894 |
| NT01TV1673 | 6 | 21316665; 21293379; 21266482; 21262766; 21245169; 21245167; 21243710; 21187428; 21124823; 21117116 |
| NT01TV1704 | 17 | 20862319; 20647003; 20587415; 19915110; 19835160; 19653099; 19578148; 7494281; 19502319; 19493015 |
| NT01TV1722 | 8 | 21291260; 20884692; 20644934; 20615551; 20000723; 19810740; 19787416; 19655853; 19332831; 19294434 |
| NT01TV1746 | 11 | 21368277; 21262357; 21210187; 21165667; 20636974; 12446819; 20363791; 11470852; 18774337; 20017756 |
| NT01ZM0525 | 4 | 21392463; 21392344; 21390205; 21386997; 21381373; 21377509; 21373190; 21369886; 21368557; 21364978 |
| NT01ZM0657 | 18 | 21054166; 20868765; 20666458; 20656870; 20469649; 20382819; 20223653; 20201406; 19948399; 19596709 |
| NT01ZM1165 | 15 | 21242066; 17693720; 10973966; 16640581; 12045829; 11952125; 1661370 |
| NT01ZM1310 | 18 | 21310784; 18462159; 17215893; 10542235; 14731273; 12686625; 12463759; 12427941; 11722746; 11524163 |
| NT01ZM1633 | 8 | 21384159; 21344856; 21342604; 21301872; 21271858; 21229706; 21183445; 21179059; 21172391; 21143473 |
| NT01ZM17981 | 8 | 21122807; 20800309; 20511297; 20511298; 20493950; 19633866; 19335447; 12809815; 18299801; 17516079 |
| NT01ZM1924 | 5 | 12207230; 10217509; 9514861; 8307984; 8504172; 8492805; 1850088; 2615765; 2643516; 3062178 |
| NT02AB01887 | 8 | 12730200; 11863436; 15299619; 8223600; 7026235 |
| NT02AB0208 | 4 | 21347378; 21346767; 21324901; 21314639; 21240845; 21232558; 21222242; 21185596; 21178109; 21151672 |
| NT02AB0821 | 8 | 21327265; 21298162; 21185447; 21130881; 18065539; 20525829; 20498375; 20422082; 16537430; 20152799 |
| NT02AB1141 | 3 | 20828616; 20536396; 18549781; 19689275; 17322901; 18084239; 17472704; 17307140; 16316735; 15162216 |
| NT02AB1300 | 12 | 18593711; 11790776; 10096074; 15299926; 17173283; 8769645; 16342953; 15876371; 15870064; 12885939 |
| NT02AB1423 | 8 | 18454933; 18358763; 17244482; 16098512; 12615344; 11888296; 11222389; 10987141; 10549856; 2050655 |
| NT02AB1593 | 5 | 21244038; 21128148; 21090681; 21067764; 21062487; 21051237; 21047298; 20960868; 20951039; 20941725 |
| NT02AB1645 | 4 | 21322476; 20698686; 20685892; 20606269; 20377263; 19816718; 18236038; 16085708; 17918839; 17918838 |
| NT02AB1674 | 12 | 17326675; 16605251; 16098472; 9626889; 8390691 |
| NT02AB1700 | 18 | 12421831; 7592417; 1327151; 8564363; 1544462; 8384683; 8304140; 1644170; 3900065; 2820842 |
| NT02AB1908 | 9 | 20554032; 19491374; 18787057; 17645593; 17627014; 7742302; 16143518; 15928316; 15632317; 15498889 |
| NT02AB2128 | 17 | 21102410; 21157480; 21098490; 21089624; 21089620; 20921270; 20855596; 20823266; 20700605; 20552441 |
| NT02AB2632 | 3 | 20923647; 20858439; 1664983; 20179180; 19269148; 20049418; 19916514; 18439024; 19440983; 19428363 |
| NT02AB26352 | 4 | 20858439; 19232353; 19007418; 10570129; 16735729; 15993071; 16213677; 15774625; 15632425; 15632422 |
| NT02AB2884 | 12 | 20734497; 11054422; 16866358; 11246021; 16478682; 15242607; 15014068; 10945280; 12057200; 11983085 |
| NT02AB3015 | 3 | 21376013; 21367857; 21300747; 21281577; 21278078; 21209369; 21166127; 21144855; 21131637; 21114988 |
| NT02AB3345 | 18 | 20883780; 20854794; 20805305; 20734919; 20643903; 20578816; 20506327; 20495821; 20421393; 20421346 |
| NT02AB3567 | 8 | 21256113; 21181282; 21179429; 21115004; 21091955; 21031021; 20725717; 20686773; 20639133; 20616077 |
| NT02AB3812 | 15 | 21372483; 21362440; 21352201; 21347702; 21324962; 21303924; 21301095; 21293840; 21280117; 21218381 |
| NT02AB4130 | 8 | 18650926; 6861765; 17292891; 11457010; 12709052; 11926822; 10648101; 8856071; 9237682; 7619827 |
| NT02AB4451 | 3 | 21030539; 20707404; 19279143; 19857612; 19825675; 19414810; 19247962; 12582168; 19004021; 9736698 |
| NT02AB4505 | 13 | 21393037; 21392503; 21391979; 21390327; 21390211; 21390183; 21388430; 21387124; 21386816; 21386137 |
| NT02AB4691 | 12 | 18684121; 18824173; 16622848; 10426423; 15255192 |
| NT02AB4699 | 6 | 11237011; 8345525; 2360047; 11675493; 15304222; 14960380; 9207105; 9393713; 12419895; 10739867 |
| NT02AB4940 | 12 | 21394642; 21394524; 21394089; 21393611; 21393360; 21393357; 21393354; 21392933; 21391923; 21389735 |
| NT02AB4973 | 1 | 21047785; 20379751; 20039042; 19955263; 19383689; 19298998; 19011861; 11368918; 18050920; 17853367 |
| NT02AB4990 | 12 | 20456877; 18375224; 10522227; 18598777; 9726890; 16099524; 9098049; 15355531; 12890017; 3123462 |
| NT02AV0436 | 8 | 20528260; 10570187; 19923224; 19818767; 18563405; 10675553; 17120764; 17007822; 16735460; 16293432 |
| NT02AV0630 | 8 | 21205028; 21171997; 20974920; 20966083; 20960138; 20960122; 20954716; 20878669; 20851663; 20652619 |
| NT02AV0934 | 5 | 19996682; 16610802; 15566096; 14983083; 12782305; 12591109; 12484756; 8540453; 11062148; 10978156 |
| NT02AV1305 | 18 | 21372178; 21371898; 21343424; 21309982; 21309871; 21266460; 21135574; 21057222; 21051552; 20960128 |
| NT02AV1804 | 8 | 21170880; 21059656; 21048022; 21030607; 20978133; 20932953; 20923642; 20856225; 20844245; 20841472 |
| NT02AV1967 | 1 | 21152904; 21128244; 21095151; 21072851; 20673702; 20637282; 15851013; 21068576; 20227313 |
| NT02AV2195 | 15 | 21087384; 20581184; 20483622; 20467813; 20460722; 19124769; 19783475; 18535838; 19608607; 15109491 |
| NT02AV2359 | 12 | 20713620; 20679205; 19729222; 19696109; 19553530; 19271156; 19220794; 18996356; 18835234; 10531258 |
| NT02AV25184 | 8 | 19172857; 15555940; 15274915; 15207829; 12928628; 12774502; 12507688; 11763133; 11544105; 10987952 |
| NT02AV3153 | 15 | 21393632; 21383064; 21329877; 21309574; 21289099; 21266852; 21263071; 21257604; 21245191; 21239583 |
| NT02AV32335 | 8 | 20795366; 16460679; 12116371; 10388620; 1469725; 9879663; 1901566; 8168545; 3092842; 6773586 |
| NT02AV3483 | 4 | 21383852; 21310477; 21198551; 21185823; 21149590; 21126059; 21112299; 20962032; 20952678; 20946259 |
| NT02AV3713 | 3 | 15906653; 12642575; 7984417; 9538012; 8541300; 6330743 |
| NT02AV3861 | 11 | 19703395; 8945505; 15967569; 11152618; 10974534; 1544582; 2158980; 8565068; 1325658; 1779760 |
| NT02AV3863 | 11 | 21325025; 21140828; 20973220; 19703395; 20538004; 20433602; 20192979; 19579137; 18212785; 16787511 |
| NT02AV3871 | 11 | 19703395; 20538004; 10737770; 15005630; 10666445; 11554564; 11292332; 9790683; 11152618; 10704304 |
| NT02AV43285 | 8 | 21093535; 21043114; 20960144; 20829052; 20701290; 20603706; 16666412; 20450882; 20059735; 19760261 |
| NT02AV43357 | 8 | 21365653; 21220760; 21152918; 20653886; 20599235; 20561259; 20424165; 20299400; 20159572; 20097872 |
| NT02AV4425 | 8 | 20922445; 19665501; 9757107; 17191848; 16547054; 17185227; 16478474; 15906398; 15878763; 15350127 |
| NT02AV4451 | 12 | 21195058; 21047684; 20702583; 20376782; 20367757; 20040394; 19968759; 19786827; 19598126; 11043569 |
| NT02AV4688 | 9 | 21173264; 10747865; 20424004; 20349267; 20188875; 20139186; 20012191; 19937203; 19875678; 19799856 |
| NT02AV4731 | 5 | 21212358; 20221547; 20221527; 20030377; 18697949; 6384184 |
| NT02AV4822 | 4 | 20826798; 20630867; 16925440; 18027571; 9665853; 12244065; 11374583; 11306561 |
| NT02AV5152 | 5 | 20837006; 11534662; 19851340; 18436239; 3350687; 15667206; 15306023; 12965206; 12112844; 12051945 |
| NT02BB0015 | 12 | 21138844; 20920237; 18164725; 17937625; 15840585; 15230349; 14976191; 9867817; 8163020; 11162102 |
| NT02BB0025 | 12 | 21327889; 21303942; 21300786; 21282417; 21261463; 21232591; 21153812; 21147958; 21098100; 20874089 |
| NT02BB0113 | 4 | 21330432; 14960717; 10640603; 9224882; 8998979; 8830266; 8196543 |
| NT02BB0116 | NT02BB0116 | 19734688; 16791830; 16750290; 11250542; 9862466; 7881553 |
| NT02BB0230 | 6 | 21216906; 21091440; 20823921; 20622008; 20451531; 20451470; 20339440; 19208629; 19618961; 21255274 |
| NT02BB0277 | 18 | 21055952; 20852101; 20822102; 21082744; 20719367; 20698689; 20688200; 20683270; 20630813 |
| NT02BB0416 | 13 | 21113134; 20948310; 20864040; 20795652; 20723624; 20409730; 20299527; 20145189; 20097372; 20086100 |
| NT02BB0485 | 4 | 21392573; 21391306; 21389347; 21382340; 21381925; 21378260; 21372321; 21371515; 21367778; 21366422 |
| NT02BB0491 | 15 | 19118355; 8982457; 1639496; 8200538; 8235653; 1527488; 6094484 |
| NT02BB0513 | 12 | 21247409; 21238933; 21151935; 21098233; 21072173; 20961427; 20960971; 20950468; 20861008; 20852886 |
| NT02BB0673 | 8 | 17668316; 17237971; 15025931; 17126302; 12810543; 11330036; 10727111; 8195701; 7503979; 1930696 |
| NT02BB0732 | 4 | 21118484; 21118278; 21109564; 21091499; 20889747; 20819931; 2985470; 20647312; 20643117; 20576688 |
| NT02BB0753 | 18 | 9768848; 14594797; 8662767; 7713912; 2136858 |
| NT02BB0786 | 12 | 17259602; 14622420; 1809829; 8837412; 9668097; 9623911; 3768954; 9224881; 8820654; 8817497 |
| NT02BB0884 | 2 | 20823206; 20655923; 20534881; 20196890; 20136525; 20018161; 19819289; 15528186; 19715473; 19621134 |
| NT02BB10921 | 8 | 21393235; 21385318; 21384170; 21364532; 21353259; 21352236; 21349279; 21344490; 21344482; 21343667 |
| NT02BB1112 | 18 | 17950243; 17221235; 10400644; 11473129; 11237621; 8206159 |
| NT02BB1148 | 12 | 20937127; 20215707; 19655395; 18074130; 17498549; 17415567; 17348445; 17347015; 16904239; 15946568 |
| NT02BB1186 | 13 | 21317262; 21306440; 21122111; 20923667; 20889794; 20863290; 20707605; 20669901; 20650900; 20568755 |
| NT02BB1302 | 12 | 21394468; 21392420; 21389182; 21385871; 21384360; 21377848; 21373949; 21372510; 21369597; 21368896 |
| NT02BB1439 | 3 | 17600077; 16809025; 10760164; 10737641; 3237054; 3087960; 6097798; 773686 |
| NT02BB1468 | 8 | 20963615; 20886839; 20498375; 20087639; 4624447; 17139260; 19685871; 19651443; 19406771; 19387485 |
| NT02BB1492 | 12 | 21383973; 21378178; 21337395; 21321126; 21257963; 21256630; 21233413; 21209007 |
| NT02BB1724 | 15 | 19781550; 12730324; 14561776; 11179370; 17217960; 14651344; 12650455; 12460564; 11931559; 11931556 |
| NT02BB1750 | 15 | 20631154; 18442486; 17405150; 15331633; 10589826; 15019993; 14565662; 11948780; 11932251; 11870094 |
| NT02BB1829 | 12 | 21289046; 21233160; 21115658; 20884784; 19810804; 11115111; 16428391; 3203386; 19137300; 11553781 |
| NT02BB1862 | 4 | 20655466; 20375016; 20347402; 17453916; 16860732; 16290064; 7651842; 12177301; 12504013 |
| NT02BB1869 | 3 | 21074048; 20920790; 20620870; 20608745; 20128627; 20061535; 19935678; 19209901; 17965478; 17936593 |
| NT02BB2020 | 12 | 18726173; 9751889; 8940060; 9110982; 15748989; 15458417; 11743110; 15120068; 14766922; 14572649 |
| NT02BB2150 | 12 | 20956533; 20194510; 15278440; 14622290; 12490407; 11060315; 9163530; 9244184; 3139668 |
| NT02BB2202 | 15 | 15263010; 17303566; 18757816; 18239412; 10411747; 2307681; 16644280; 16530010; 11244072; 12972253 |
| NT02BB2209 | 14 | 21369979; 19683509; 19385996; 17651436; 18597482; 18164314; 17996716; 11053429; 17651436; 16014955 |
| NT02BB2290 | 4 | 21131490; 20738376; 20543066; 20471399; 16547053; 19732340; 19684067; 14688104; 19474207; 18621896 |
| NT02BB2307 | 12 | 21369703; 21302309; 21248339; 21235517; 21228126; 21205077; 21194957; 21194377; 21051527; 21048494 |
| NT02BB2329 | 12 | 21354644; 21276854; 21261463; 21261072; 21220481; 21212976; 21188150; 21173569; 21112419; 21094132 |
| NT02BB2559 | 12 | 21333981; 21310889; 21270764; 21156982; 21141460; 21108999; 21106748; 21106737; 21092349; 21090173 |
| NT02BB2961 | 12 | 20197200; 20025663; 18222721; 17222396; 16407287; 15721771; 15284538; 14630828; 12960111; 12725295 |
| NT02BB3063 | 4 | 16487743; 15731070; 19903545; 17259602; 9204707; 17979986; 19019140; 11978773; 12907559; 10496945 |
| NT02BB3085 | 15 | 21078995; 21071627; 21050859; 20979345; 20971918; 20946856; 20618698; 20593779; 20558099; 20507988 |
| NT02BB3162 | 15 | 21295603; 21229249; 21183673; 21078995; 21060253; 21057008; 20965974; 20946840; 20936511; 20919928 |
| NT02BB3378 | 2 | 21265776; 21068394; 18399988; 18323662; 18237633; 16364235; 11300770; 11213485; 10828978; 9610381 |
| NT02CH0012 | 9 | 21388747; 21330637; 21327327; 21315740; 21269353; 21257793; 21171605; 21148782; 21147110; 21075079 |
| NT02CH0053 | 12 | 21332448; 21326199; 21320870; 21252205; 21247409; 21203342; 21075890; 21036998; 21031019; 20814423 |
| NT02CH0125 | 3 | 20865175; 20210661; 11320139; 15687380; 18173801; 17426021; 12468728; 15032825; 14997492; 11748726 |
| NT02CH0177 | 12 | 21387826; 21376122; 21369703; 21344411; 21342114; 21339174; 21328067; 21307569; 21307147; 21302309 |
| NT02CH0186 | 6 | 21080016; 21169503; 20802486; 20686482; 20601468; 20534341; 20219261; 20165898; 20052594; 19649925 |
| NT02CH0206 | 17 | 21220116; 21155874; 20971899; 20937888; 20626317; 20600947; 19602144; 20304988; 20233930; 20185507 |
| NT02CH0207 | 17 | 19602144; 20154128; 19700407; 18791018; 7476207; 17766240; 16319496; 16701502; 16319496; 879738 |
| NT02CH02591 | 2 | 10383756; 10075431; 8885414; 791939; 14217462 |
| NT02CH0295 | 6 | 18342605; 11477104; 8386271; 1657645; 2841153 |
| NT02CH0363 | 4 | 21343356; 21317257; 21216994; 21097622; 20971901; 20920184; 20862217; 20831592; 20828376; 20815824 |
| NT02CH0519 | 6 | 21329494; 20978083; 20942908; 20827719; 20810907; 20947627; 20727851; 10210213; 10636868 |
| NT02CH0546 | 8 | 21386914; 21382479; 21361356; 21354339; 21352495; 21343298; 21334283; 21297919; 21279427; 21185334 |
| NT02CH0869 | 18 | 20971110; 20487300; 20562301; 20146748; 20059695; 8334704; 12622816; 10097157; 6437444; 2120234 |
| NT02CH0912 | 2 | 21259066; 21150822; 21103937; 21073196; 21048046; 20934343; 20823222; 20814629; 20603160; 20543190 |
| NT02CH0963 | 18 | 21393854; 21390132; 21388532; 21383001; 21378193; 21378185; 21372133; 21368759; 21368180; 21366233 |
| NT02CH0973 | 3 | 21112636; 21057010; 21040977; 20959443; 20871101; 20850421; 20837769; 20833156; 20832292; 20813161 |
| NT02CH1185 | 2 | 21205917; 21182989; 21051239; 20675471; 20370816; 20370816; 20412262; 20209473; 20138415; 19760906 |
| NT02CH1206 | 8 | 16669619; 16211847; 11334784; 10985736; 9257693; 8830672; 7984100 |
| NT02CH1277 | 12 | 21343292; 21333546; 21325636; 21306441; 21274957; 21258033; 21253550; 21251003; 21238935; 21229878 |
| NT02CH1319 | 9 | 21369973; 21322560; 21054894; 20028524; 19715665; 19651238; 19579033; 19453447; 18773201; 18437283 |
| NT02CH1331 | 5 | 20635345; 20631318; 20207756; 10194322; 7565414; 10368287; 7972100; 14981304; 14749331; 12471450 |
| NT02CH1369 | 6 | 21084480; 20511501; 20384788; 20202939; 20147403; 18657510; 19450506; 19143592; 7592430; 15994803 |
| NT02CH1400 | 2 | 21368171; 21255426; 21142117; 21081498; 21075928; 21072368; 20971904; 20863064; 20861021; 20843816 |
| NT02CH14827 | 8 | 21392042; 21377330; 21366559; 21361867; 21359968; 21359206; 21352963; 21328391; 21327936; 21324164 |
| NT02CH1540 | 6 | 20298190; 11106026; 10523315; 15758242; 15212893; 15136039; 10669597; 12736; 10361305; 9398514 |
| NT02CH1626 | 4 | 21330537; 21276251; 20733058; 20544960; 8458342; 6292235; 19558326; 19533031; 19497327; 18617187 |
| NT02CH1643 | 12 | 10794418; 8769645; 10455123; 345085; 7674946; 7828908; 7798200 |
| NT02CH1653 | 12 | 20023146; 8759852; 17929834; 16561900; 7894706 |
| NT02CH1769 | 8 | 19159700; 17971396; 12100551; 10631007; 11054562; 9831660; 8703024 |
| NT02CH1788 | 18 | 19023664; 9811636; 18176545; 15899945; 10966876; 11282473; 10844693; 8412663 |
| NT02CH1856 | 6 | 21278727; 21245169; 21238786; 21234761; 21229606; 21187428; 21187328; 21187070; 21141724; 21115420 |
| NT02CH1877 | 17 | 21075929; 20691896; 20678145; 20581211; 20547861; 20545843; 20530410; 20483314; 20660472; 20233932 |
| NT02CH2064 | 6 | 21354867; 21263027; 21239234; 21195320; 21115857; 21091774; 21091445; 21078976; 21047769; 21115816 |
| NT02CH2081 | 3 | 21370307; 21108133; 20495944; 12465928; 19904366; 19804974; 5700707; 1815783; 19121323; 18996617 |
| NT02CH2082 | 18 | 21061745; 20633955; 20193962; 20079335; 19819871; 19797650; 19543710; 19290886; 19161381; 19098009 |
| NT02CH2136 | 8 | 20525253; 20473714; 16806221; 16114877; 15743763; 12164810; 12095696; 8837463; 9631507; 9261082 |
| NT02CH21474 | 8 | 21342516; 21320350; 21269876; 21193828; 21131522; 21116681; 21114521; 21114504; 21071269; 20973564 |
| NT02CH2214 | 18 | 20880838; 20818668; 20177068; 20159554; 19298183; 11402001; 17689599; 18032631; 10480946; 12077419 |
| NT02CH2269 | 4 | 21183069; 20580675; 20497333; 17233825; 16146521; 1628843; 2681146; 18759781; 10671449; 18394147 |
| NT02CH2270 | 8 | 1445195; 18436321; 16633561; 16292529; 9165069 |
| NT02CH2329 | 8 | 21354790; 21329667; 21116622; 21076874; 20939108; 20839617; 20837145; 20697695; 20661821; 20559622 |
| NT02CH2567 | 15 | 21357484; 21345171; 21326909; 21326908; 21326894; 21326893; 21325277; 21288908; 21245135; 21196497 |
| NT02CH2580 | 6 | 21393072; 21304489; 21271694; 21251107; 21131907; 21131361; 21074452; 20977236; 20871655; 20705454 |
| NT02CH2581 | 3 | 20861142; 20075623; 19899805; 19298367; 8293964; 18247346; 17124631; 17030076; 17008388; 16830093 |
| NT02CH2593 | 8 | 20580433; 18028869; 17321223; 16962276; 16675853; 15036329; 12409197; 11532445; 11527963; 11405622 |
| NT02CH2687 | 12 | 21189343; 21135574; 21115495; 21095572; 21075926; 20872041; 12382110; 20566871; 20522495; 20419406 |
| NT02CH2757 | 4 | 21391306; 21378260; 21366422; 21244534; 21223978; 21223465; 21165649; 21148728; 21091499; 21076396 |
| NT02CH3071 | 6 | 20622503; 20134230; 19304752; 19232054; 19332813; 9207015; 7473738; 2055464; 17617640; 15199175 |
| NT02CH3076 | 6 | 20957358; 1741244; 9923682; 16038930; 15713456; 12879741; 8223468; 11555298; 9862476; 9742247 |
| NT02CH3132 | 8 | 21149586; 20501611; 18393803; 17103354; 16750535; 16632246; 16611144; 16309669; 16214327; 16054014 |
| NT02CH3154 | 6 | 21330432; 21321205; 21226579; 21110240; 20821352; 20713648; 21265052; 14767481; 15738392; 20371965 |
| NT02CH3333 | 4 | 21242066; 20623345; 20223832; 12118075; 9620966; 18566135; 17880425; 17707860; 17631415; 17172020 |
| NT02CH3376 | 9 | 21068393; 20979339; 20953507; 20844583; 20713103; 20145252; 20132453; 20085613; 19932173; 19797465 |
| NT02CH3383 | 8 | 20466730; 18174132; 17419738; 16920107; 16629658; 9790980; 16042600; 15465823; 8341666; 11744735 |
| NT02CH3439 | 13 | 21322634; 20805241; 20557293; 20482517; 20471434; 20306515; 20194621; 20150917; 20136139; 19940155 |
| NT02CH3610 | 1 | 21235400; 21167158; 20630732; 20592025; 20554844; 20460582; 19900465; 14576855; 12633501; 18976669 |
| NT02CH3615 | 12 | 20949898; 20924576; 20922471; 20821392; 20659418; 20557125; 20124006; 19734126; 19734121; 19309077 |
| NT02CH3667 | 6 | 21390132; 21189350; 21098490; 20921990; 20713529; 20603158; 20566477; 20443037; 20354588; 20299199 |
| NT02CH3802 | 6 | 21389581; 21388174; 21384169; 21383907; 21374657; 21372597; 21370602; 21370284; 21366272 |
| NT02CH3817 | 11 | 20107991; 2231712; 19560223; 14730025; 17719266; 16349277; 17048701; 16349277; 9139913; 15979388 |
| NT02CH3826 | 11 | 20531477; 20498502; 19913618; 6318086; 15607229; 10933510; 9023104; 11743729; 4120454; 9680195 |
| NT02CH3946 | 6 | 21365007; 21331613; 21327711; 21320783; 21318329; 21304493; 21292686; 21288129; 21287394; 21249460 |
| NT02CH3947 | 6 | 21278781; 21244040; 21103343; 21038902; 20927102; 20805875; 20714506; 20547483; 20447876; 20347426 |
| NT02DA0034 | 15 | 21039781; 20545866; 18052041; 17400438; 16154092; 16573700; 16543275; 16434396; 10684935; 15979641 |
| NT02DA0084 | 2 | 10880976; 10545188; 9756625; 9748348; 7629164; 6413254 |
| NT02DA0119 | 9 | 20966403; 20939191; 20843517; 20684826; 20523008; 20488739; 20460577; 20434185; 20404512; 20403694 |
| NT02DA0348 | 4 | 21393450; 21391724; 21350490; 21346352; 21325274; 21253903; 21249122; 21211813; 21178960; 21147064 |
| NT02DA0511 | 5 | 21281647; 21265577; 21130773; 20884330; 20810615; 20570749; 20541593; 20423300; 20415666; 20394832 |
| NT02DA05250 | 8 | 21296938; 17927566; 17635929; 17571210; 17157320; 11583990; 11285267; 10832633; 10796014; 10656808 |
| NT02DA0578 | 18 | 21393196; 21351733; 21317882; 21179061; 21176777; 21149736; 21106816; 21097502; 21075126; 21048022 |
| NT02DA0636 | 4 | 21391663; 21389200; 21385867; 21385584; 21378306; 21365742; 21365111; 21354305; 21312070; 21282621 |
| NT02DA0676 | 8 | 9613585; 16653176; 16663726; 9299297; 7796904; 8459836; 1901022; 2668282; 2826438 |
| NT02DA06964 | 8 | 20713411; 20392438; 19919544; 19708823; 11135667; 18586271; 18468620; 18331355; 17419725; 5435 |
| NT02DA0728 | 12 | 20965178; 20802067; 12603741; 19996088; 16354698; 2675964; 19101630; 9882303; 18321969; 18788473 |
| NT02DA0790 | 1 | 20961734; 20871626; 18481057; 16960959; 15526543; 12670956; 8719248; 11032894; 9565331; 8933102 |
| NT02DA0812 | 15 | 10081584; 9563844; 8858582; 8552028; 3054814; 1766374; 2046550 |
| NT02DA0849 | 4 | 21393196; 21378194; 21350490; 21347426; 21338683; 21338414; 21330374; 21329359; 21327044; 21326916 |
| NT02DA0909 | 4 | 9554854; 19306353; 10474183; 1563786; 16936294; 14636673; 15931497; 15823710; 15503857; 15480576 |
| NT02DA0946 | 2 | 21384912; 21348864; 21333380; 21282331; 21053013; 20971904; 20969648; 20870959; 20822098; 20690630 |
| NT02DA1025 | 4 | 21078967; 20487019; 20228837; 19833774; 19686342; 19562667; 19545630; 16291662; 19052865; 18996426 |
| NT02DA1149 | 18 | 21359175; 21327819; 20693447; 19962946; 19628817; 12140659; 19246762; 9210286; 17485086; 19158662 |
| NT02DA1159 | 18 | 21236254; 21103361; 21045261; 11108699; 19943161; 19803484; 19762344; 19734123; 19671009; 16382132 |
| NT02DA1167 | 11 | 21362621; 21278448; 21270160; 21257796; 21183396; 21129204; 21068246; 21068232; 20921065; 20808903 |
| NT02DA1181 | 8 | 21189647; 20957036; 20949620; 20889786; 20676631; 20559622; 20519920; 20457944; 20447995; 20399532 |
| NT02DA1283 | 18 | 21238582; 20952578; 20952391; 20855510; 20704181; 20662012; 20600116; 20596754; 20545847; 20396748 |
| NT02DA1337 | 17 | 21173851; 20937035; 20876278; 20862689; 20836043; 20832473; 20801873; 20729910; 20691182; 20616871 |
| NT02DA1356 | 8 | 20532738; 20143161; 20077566; 15865447; 15544340; 822747; 12634336; 12560602; 12482588; 9287422 |
| NT02DA1407 | 13 | 18400176; 19564499; 19446023; 7677746; 7535280; 10805779; 15522293; 14729335; 12823975; 2504932 |
| NT02DA1472 | 3 | 21375716; 21341524; 21333724; 21331046; 21205637; 21326935; 21324191; 21317344; 21306444; 21302401 |
| NT02DA1491 | 4 | 21155139; 21091498; 20922738; 20573715; 20542912; 20516200; 20298190; 20033058; 19788545; 19788546 |
| NT02DA15597 | 8 | 20795641; 20702416; 20604568; 20530482; 20022504; 19932181; 18367664; 17888883; 19615964; 19594755 |
| NT02DA1588 | 9 | 20064615; 20047918; 19900769; 17904577; 17198382; 16107329; 11583992; 9305965; 3549716; 14320488 |
| NT02DA1660 | 5 | 11823455; 9683649; 11170380; 10978548; 7559591; 8226893; 2722786 |
| NT02DA1667 | 8 | 21179059; 21068384; 20971856; 20952576; 20876281; 20862300; 20807763; 20700628; 20624914; 20622059 |
| NT02DA1785 | 18 | 17027372; 17014077; 15644918; 15225600; 11293413 |
| NT02DA18492 | 8 | 21304509; 21273120; 21228928; 21209090; 21092234; 21051878; 20978192; 20930427; 20806417; 20693323 |
| NT02DA1891 | 15 | 21078995; 19958380; 5432063; 19246748; 18694439; 18792680; 18469109; 17628138; 16434396; 14749483 |
| NT02DA1922 | 18 | 21254623; 21214274; 21212869; 21173279; 21056583; 20919451; 20888838; 20881034; 20868747; 20861358 |
| NT02DA1942 | 6 | 20889748; 11278072; 8913296; 8543037; 7867950; 2309119; 14124321; 3536664 |
| NT02DA1956 | 1 | 21044129; 19715939; 16928788; 19187205; 19402116; 11535564; 18481057; 18504828; 17701131; 17661761 |
| NT02DA2185 | 18 | 21394209; 21393546; 21391900; 21390270; 21388385; 21386094; 21383096; 21378004; 21371595; 21371431 |
| NT02DA2211 | 6 | 20571792; 2448137; 19271042; 19022275; 19015814; 18817159; 18060506; 17991030; 17907195; 17680562 |
| NT02DA2282 | 1 | 21102469; 21031542; 20888617; 20883697; 20735845; 20733534; 20653695; 20626588; 18056995; 20449652 |
| NT02DA23950 | 4 | 19162196; 17640273; 15576792; 16309817; 9675890; 10361275; 9642082; 8152377; 1453957 |
| NT02DA2532 | 6 | 21375713; 21309924; 21227917; 21124899; 21124785; 21110832; 21075347 |
| NT02DA2711 | 3 | 21379342; 21377170; 21362626; 21359182; 21349544; 21346816; 21340723; 21329659; 21327158; 21324893 |
| NT02DA2738 | 11 | 7984417; 10486244; 12413691; 12196537; 9533721; 8892635; 4927838; 8061223; 8192358; 2467933 |
| NT02DA2749 | 3 | 21229881; 21030539; 20707404; 20347067; 20188057; 20161474; 15639242; 18258263; 16040347; 11500481 |
| NT02DA2809 | 12 | 21335977; 20418496; 20418037; 8898387; 19143600; 19060069; 18785662; 18718532; 18674634; 18227252 |
| NT02DA3051 | 8 | 20964856; 20236286; 19447430; 19288973; 18073375; 17961501; 17433859; 16740275; 16137709; 15356994 |
| NT02DA32442 | 8 | 19060309; 16849807; 12374303; 11803023; 11062460; 11279023; 11178972; 10216163; 7929327; 9521736 |
| NT02DA3301 | 8 | 21389123; 21375722; 21268127; 21250602; 21225617; 21135065; 21093509; 20961851; 20922361; 20883105 |
| NT02DA3505 | 15 | 21320584; 21317318; 21315771; 21295603; 21284862; 21239493; 21217003; 21216996; 21216906; 21193607 |
| NT02DA3569 | 6 | 21358597; 21354867; 21311894; 21115420; 21040891; 21035608; 20920750; 20864418; 20858090; 20811726 |
| NT02DA3646 | 18 | 21394901; 21394897; 21394549; 21394439; 21393578; 21393193; 21392732; 21392146; 21392091; 21392047 |
| NT02DA3660 | 8 | 21144838; 20828554; 20828325; 20724146; 20676633; 20950372; 20507888; 20453146; 20224884; 20205700 |
| NT02DA37351 | 8 | 18664520; 10231382; 12081483; 9603909; 9271215; 7698662; 8119301 |
| NT02DA37771 | 8 | 2834341; 9047371; 12962479; 12206758; 11976494; 11732896; 11732895; 11054289; 10821675; 9548961 |
| NT02DA3842 | 9 | 18272295; 11803016; 11524131; 11150659; 10627041; 808529 |
| NT02DA4095 | 14 | 21321206; 21145019; 21059902; 20930138; 20856929; 20817757; 20692617; 20536734; 20616084; 20497503 |
| NT02DA4242 | 9 | 17604723; 18473732; 17898795; 14507721; 17351984; 17296494; 16824039; 16556571; 16200394; 16020265 |
| NT02DA4366 | 15 | 21106055; 20980270; 19602144; 20097789; 19880399; 19855379; 8460142; 18384044; 19224952; 15263010 |
| NT02DA4512 | 8 | 11170458; 10537212; 3067084; 2537825; 1315704; 7894709; 13449036; 8045431; 2154378; 1469733 |
| NT02DA4531 | 8 | 21305036; 21272750; 21220358; 21185447; 21085043; 21081705; 21068384; 20952576; 20922212; 20807714 |
| NT02DA4713 | 13 | 21219451; 21149572; 21142053; 21037010; 20953507; 20817755; 20615421; 20608977; 20587522; 20575532 |
| NT02DA4739 | 3 | 20976701; 20202763; 19762441; 17400891; 12397186; 18550550; 16099208; 18342249; 18314348; 17533641 |
| NT02LM0602 | 4 | 19788546; 18391964; 9495771; 17360038; 17259614; 10648549; 11973144; 9252185 |
| NT02MA0014 | 4 | 21108067; 20808885; 20833311; 20525091; 20435045; 20355710; 20138001; 20133181; 20088541; 9437425 |
| NT02MA0022 | 15 | 21091513; 20876874; 20855582; 20832320; 20558508; 20487303; 11121743; 20122866; 20061469; 20044484 |
| NT02MA0042 | 17 | 21164032; 21156400; 21156006; 21124948; 21124318; 21090588; 21073264; 21071633; 21062506; 21036999 |
| NT02MA0090 | 6 | 21389894; 21358597; 21355073; 21354867; 21348638; 21344488; 21336262; 21329650; 21313735; 21311894 |
| NT02MA0168 | 18 | 21309115; 21289302; 21173226; 21148490; 21148012; 21135361; 21127383; 21052943; 21051529; 21047781 |
| NT02MA0246 | 15 | 18433629; 17635923; 17628134; 17609131; 15687212; 17064367; 10496909; 15273097; 12654815; 10447877 |
| NT02MA03693 | 8 | 20795494; 11532013; 9076739; 8954165; 8617280; 8575452; 1772346 |
| NT02MA0430 | 15 | 21394106; 21393865; 21393365; 21392374; 21391292; 21391223; 21391093; 21390188; 21389275; 21386894 |
| NT02MA0759 | 12 | 21125796; 21094166; 21051851; 20950979; 20844218; 20631311; 20631135; 10859029; 20383012; 20299243 |
| NT02MA0816 | 6 | 20335175; 19342892; 18657502; 16840322; 11952839; 17689530; 11864567; 17495541; 10499591; 17192781 |
| NT02MA0873 | 17 | 21326933; 21290819; 21187417; 21172662; 21164032; 21124318; 21090588; 21090248; 21085632; 21076032 |
| NT02MA0957 | 18 | 21078855; 20541393; 20492358; 20141110; 18805467; 19892400; 19883597; 19702307; 1512262; 19580276 |
| NT02MA1093 | 14 | 18515330; 18082626; 17711307; 17550785; 9144792; 16819826; 16752903; 16621066; 16395552; 15381710 |
| NT02MA1471 | 5 | 12859215; 18252716; 16202390; 11823455; 11456800; 3141411; 9503607; 1328190; 4354333; 15966103 |
| NT02MA1492 | 5 | 20635345; 20631318; 20207756; 19476442; 18767150; 18682379; 11123699; 17309280; 7565414; 10368287 |
| NT02MA1561 | 3 | 21091511; 21090263; 20936123; 20929722; 20833802; 20829284; 20823255; 20802039; 12382110; 20657621 |
| NT02MA1690 | 8 | 20503342; 18950397; 18854137; 17893649; 15576047; 11827457 |
| NT02MA1851 | 3 | 20678145; 19969519; 19724122; 10715117; 17353187; 14699122; 14557260; 12561102; 11886751; 9609691 |
| NT02MA2293 | 12 | 21151101; 20817732; 20513640; 20462516; 20456597; 20364150; 20226173; 19822524; 19755481; 11855831 |
| NT02MA2331 | 6 | 21300774; 21259161; 21231972; 21194552; 21155913; 21113026; 20846957; 20829609; 20811586 |
| NT02MA2498 | 18 | 21318881; 20159658; 17578420; 14982633; 14643892; 10964926; 9045651; 1939142 |
| NT02MA2611 | 6 | 19399991; 15876366; 14756795; 11428897; 10568806; 1406951; 9714164 |
| NT02MA2658 | 3 | 16169165; 8793566; 7520113; 7322185; 6804232; 7018908; 6245876; 6775160 |
| NT02MA3021 | 8 | 21261075; 20455436; 16880; 20012884; 16299075; 19351043; 18325924; 18462832; 17977448; 11155984 |
| NT02MA3208 | 12 | 21392058; 21387310; 21383917; 21383693; 21375584; 21368222; 21368221; 21367592; 21365746; 21364954 |
| NT02MA3279 | 3 | 21080912; 20836895; 20823921; 20726530; 20550197; 20523006; 20347067; 11342591; 20217156; 20185508 |
| NT02MA3638 | 8 | 21094140; 21067524; 21056970; 21047521; 20973592; 20929212; 20873742; 20831242; 20712003; 20640799 |
| NT02MA3977 | 6 | 21336027; 21086493; 21078962; 21051354; 20956557; 20925924; 20811460; 20736290; 20730596; 20708016 |
| NT02MA3997 | 12 | 20825230; 19557407; 11860363; 18576673; 18437310; 5432063; 16270998; 15274616; 15138277; 15122907 |
| NT02MA4026 | 14 | 21386840; 21382338; 21372352; 21368461; 21362301; 21332317; 21325291; 21324566; 21301320; 21294420 |
| NT02MA4048 | 12 | 21382349; 21381030; 21345307; 21339823; 21257602; 21256111; 21222654; 21182594; 21046238 |
| NT02MA4331 | 6 | 21389046; 21147134; 20888916; 20880409; 20542142; 11048718; 20345942; 20211667; 20144704; 20019078 |
| NT02MA4697 | 3 | 18304454; 10336984; 11886840; 11886839; 10749855; 6249972 |
| NT02MA4770 | 11 | 21345955; 21289143; 21228534; 21206037; 21129201; 21127059; 21109524; 21079776; 21062506; 20963614 |
| NT02MA4828 | 12 | 21129723; 20966391; 20926780; 20812720; 20593410; 20534808; 20461058; 20304782; 20236927; 20226437 |
| NT02MA4829 | 17 | 21351518; 21330472; 21233223; 21157480; 21089620; 20926573; 20888817; 20851187; 20826615; 20634199 |
| NT02MA4851 | 8 | 21377422; 21333622; 21297352; 21192932; 21080034; 21075872; 20929256; 20889679; 20869341; 20866111 |
| NT02MA5173 | 9 | 19060391; 17263560; 16010976; 15946938; 10962286; 10405762 |
| NT02MA5249 | 15 | 21320609; 21291396; 21159950; 21149488; 21125642; 21062740; 21037200; 20870161; 20846531; 20825363 |
| NT02MA5436 | 4 | 21106757; 21069159; 20966125; 20947511; 20666229; 20666228; 20666225; 20666220; 20655873; 20638426 |
| NT02MA5512 | 12 | 21383992; 21285457; 21238935; 21220053; 21196275; 21143681; 21135106; 21116630; 21115099 |
| NT02MA56905 | 8 | 21384181; 21382037; 21365653; 21359551; 21353267; 21329207; 21325606; 21311030; 21309792; 21308384 |
| NT02MF0019 | 18 | 21315686; 21282331; 21209088; 21078855; 21075893; 20600125; 20382768; 20154136; 19745109; 11981968 |
| NT02MF0053 | 18 | 20978004; 20652498; 20643901; 18473883; 20360302; 20233304; 20053485; 19949928; 19254725; 19622348 |
| NT02MF0060 | 2 | 21179031; 2581252; 21072050; 18182978; 18045808; 19174154; 18232714; 17896913; 17845983; 17534577 |
| NT02MF0067 | 4 | 1313537; 19731316; 9774345; 3273395; 10696437; 10368791; 9837772; 9759872; 9710539; 8940364 |
| NT02MF0219 | 1 | 21290823; 21113127; 21102444; 21093415; 20948310; 20947765; 20941850; 20858867; 20843777; 20836997 |
| NT02MF0244 | 8 | 9694668; 16489742; 16304640; 15333955; 15240097; 15109729; 12547196; 11978868; 11395407; 11042472 |
| NT02MF0371 | 14 | 21295473; 21139397; 21076373; 21070810; 21062060; 21040798; 21035731; 20934342; 20848821; 20832515 |
| NT02MF0424 | 12 | 21366318; 21122159; 20946650; 20616104; 20572939; 20182770; 20002189; 11279114; 19439009; 12207705 |
| NT02MF0444 | 18 | 21367903; 21362118; 21359673; 21357485; 21325275; 21304887; 21210168; 21187326; 21103346 |
| NT02MF0644 | 6 | 21070963; 20600127; 20016583; 2466840; 19438708; 16957187; 18479467; 8663104; 12235389; 12228732 |
| NT02MF0654 | 13 | 20160120; 18611382; 12962494; 11311934; 10049785; 9016717; 7664121; 3368329; 8199244; 1105573 |
| NT02NE0203 | 13 | 21288885; 21161394; 20727857; 20429542; 20097853; 19454243; 18573842; 10075918; 15379729; 11283358 |
| NT02NE0241 | 13 | 21170087; 20878142; 18481301; 18056458; 17057733; 16185873; 2408008; 1853201; 12753362; 12703984 |
| NT02NE0334 | 13 | 21394717; 21392180; 21391976; 21389767; 21389073; 21387259; 21385875; 21385614; 21385567; 21385342 |
| NT02NE0354 | 13 | 18421856; 17647292; 10612281; 7677746; 2504932; 11279123; 10561595; 2199796; 1091919; 5432063 |
| NT02NE0402 | 6 | 21388323; 20851802; 20828312; 20693323; 15126610; 20340159; 16453745; 20037779; 19938227; 19761240 |
| NT02NE0465 | 6 | 20667794; 17873859; 17011573; 10089360; 15479781; 10486559; 9757107; 14559184; 12499561; 11697901 |
| NT02NE0539 | 17 | 21353945; 21319808; 21214526; 21183650; 21164032; 21156006; 21139582; 21117999; 21108695; 21090248 |
| NT02RE0218 | 18 | 21330432; 21284804; 21265748; 21097635; 20955240; 20722599; 20472735; 20457291; 20455262; 16950129 |
| NT02RE0234 | 12 | 21058505; 15574921; 11919638; 18067836; 16476613; 16342953; 12954635; 11147833; 10668195; 10030265 |
| NT02RE0444 | 6 | 20959412; 20889740; 20863644; 20860664; 20800707; 14976042; 19858205; 17407181; 21083131; 9557714 |
| NT02RE0447 | 9 | 21362034; 21327327; 21315740; 21300802; 21265823; 21257056; 21211954; 21179554; 21151902; 21147110 |
| NT02RE0558 | 11 | 20937911; 4598709; 15489415; 12832212; 7630711; 12399040; 10234830; 7645202; 978790; 10892750 |
| NT02RE0667 | 15 | 21242066; 21193048; 21179017; 21173231; 21169559; 21169543; 21167274; 21103944; 21098025; 20980255 |
| NT02RE1056 | 9 | 18307304; 15995648; 15988790; 12785312; 12217032 |
| NT02RE1071 | 4 | 19073264; 17308196; 17178791; 18548290; 1645287; 17238916; 1622171; 16687460; 16482431; 16000721 |
| NT02RE1090 | 4 | 21365734; 21258753; 21252275; 21154995; 21034261; 21031308; 20975958; 20121449; 16101996; 14605211 |
| NT02RE1285 | 8 | 10064717; 14735333; 11361136; 9851688; 9724544; 1311417; 9165084; 6643492; 8890912; 8001683 |
| NT02RE1581 | 15 | 21391093; 21389307; 21383852; 21383633; 21371473; 21360593; 21348830; 21344481; 21282931 |
| NT02RE1622 | 8 | 21233139; 20811810; 16624426; 17537807; 16969084; 16637013; 9582354; 14582652; 11522252; 9426197 |
| NT02RE1708 | 8 | 19638432; 17244482; 15747169; 12758076; 12678433; 12600193; 12068071; 11695833; 11295131; 11222389 |
| NT02RE1709 | 8 | 15987803; 10339593; 19128036; 16788776; 15559763; 9571020; 15115179; 15102833; 11735452; 11278828 |
| NT02RE1710 | 8 | 20610779; 20156168; 18454933; 18358763; 10952301; 11888296; 10987141; 10549856; 8536688; 2118334 |
| NT02RE2163 | 8 | 1384048; 17225146; 15313611; 15103146; 14646110; 4399881; 7873778; 6235151; 3129535 |
| NT02RE2327 | 6 | 21378185; 20944626; 20138894; 20089683; 19617314; 12214237; 10581244; 18272921; 10606516; 7568198 |
| NT02RE25750 | 4 | 21393861; 21387177; 21386087; 21385720; 21385331; 21383490; 21383152; 21383058; 21382465; 21382362 |
| NT02RE2608 | 3 | 21057009; 20576688; 20413557; 7337433; 20081033; 19929968; 19888830; 19843226; 18441060; 3862129 |
| NT02RE2658 | 2 | 20933386; 20725723; 20667786; 20630744; 20059756; 19899124; 19804570; 19719472; 19591198; 19125224 |
| NT02RE2720 | 1 | 21178309; 17804481; 8170394 |
| NT02RE2963 | 8 | 7935603; 12573288; 12091098; 12036956; 7810866 |
| NT02RE3086 | 8 | 21209382; 20554905; 20035485; 18077198; 16622056; 16601871; 16464906; 16297647; 15731894; 15558747 |
| NT02RE3267 | 17 | 10666455; 18302315; 3311729; 10551881; 14592988; 16364630; 10937990; 9501180; 12866049; 12270133 |
| NT02RE3317 | 8 | 21121258; 20930473; 20663040; 18492492; 11709194; 17989071; 17355287; 16902948; 16435204; 16379566 |
| NT02RE3589 | 18 | 21393832; 21357442; 21357299; 21354180; 21350490; 21346352; 21336797; 21328631; 21325274; 21262925 |
| NT02RE3766 | 4 | 21385992; 21382632; 21378164; 21371263; 21368902; 21364982; 21339339; 21325403; 21321120; 21317560 |
| NT02RE3882 | 15 | 10336337; 9478986; 3997821; 9353301; 7639753 |
| NT02RE39301 | 8 | 12455613; 17012386; 17929340; 17897085; 16151209; 1375309; 15362865; 15257457; 15081823; 10364471 |
| NT02RE4223 | 4 | 21378753; 21373952; 21367586; 21337413; 21335646; 21311919; 21310544; 21291501; 21262537; 21258333 |
| NT02RE4301 | 17 | 21273248; 11277442; 19202087; 8486283; 12065541; 16934437; 10361286; 15470124; 11535780; 11254138 |
| NT02RE4353 | 12 | 20802067; 18788473; 18614331; 17456188; 15726567; 15283695; 12631144; 11741588; 11408480; 11115631 |
| NT02REA0063 | 17 | 20971899; 20937888; 19906181; 19737356; 19502446; 19415331; 10966457; 19129643; 18682280; 18619465 |
| NT02REA0283 | 8 | 21324667; 21320350; 20383736; 20349184; 20198658; 15685416; 19888573; 18824465; 18813915; 17046541 |
| NT02REA0309 | 15 | 21370910; 21301757; 21188077; 21153519; 21152936; 21145993; 21135413; 21102469; 21073190; 21055431 |
| NT02REA0493 | 15 | 3897273; 19191873; 14996815; 11069241; 10906431; 18005985; 12867469; 17320105; 17173020; 17005012 |
| NT02REB0117 | 18 | 20190787; 21191377; 21187464; 21149736; 20554613; 21115899; 21104926; 21103977; 21094854; 21077936 |
| NT02REB0127 | 18 | 21369825; 21366542; 21041497; 21034832; 20888343; 20855510; 20808924; 20805402; 20662775; 20656493 |
| NT02REB0219 | 9 | 20825197; 20370610; 20045488; 19772884; 19465092; 19271216; 16400714; 9890635; 18691538; 1845298 |
| NT02REB03131 | 8 | 20977999; 20822145; 20809285; 20516598; 20088877; 19838696; 19589832; 19472978; 17890304; 17653540 |
| NT02REB0451 | 8 | 12102556; 17564616; 12492476; 10027980; 1772346; 2128800 |
| NT02REC0063 | 9 | 8406042; 19686777; 16313613; 16638529; 16297647; 15904873; 15458418; 14684903; 14669153; 12562856 |
| NT02REC0203 | 15 | 1848225; 19705488; 19692483; 19540251; 16988189; 2165385; 18655822; 10827456; 18436623; 18402905 |
| NT02REC0237 | 18 | 19396940; 18976663; 18258424; 16857013; 16092522; 15546669; 12904549; 11212326; 10071218; 9322765 |
| NT02RED0057 | 15 | 16791832; 16500039; 9574502; 8469115; 16453586 |
| NT02SS0082 | 6 | 21330836; 21247820; 21190441; 21044351; 20962099; 20833151; 20824783; 20664914; 20648054; 20635369 |
| NT02SS0110 | 3 | 20843801; 3611062; 20364833; 20188057; 20077550; 20030628; 19929855; 19720067; 19277539; 19245333 |
| NT02SS0225 | 13 | 21352546; 21262594; 21219451; 21195156; 21177880; 21151095; 21134383; 21037010; 20889753; 20884071 |
| NT02SS0232 | 14 | 21385632; 21374069; 21371039; 21354216; 21336542; 21331762; 21315198; 21295139; 21282572; 21155784 |
| NT02SS0443 | 13 | 21383132; 21311021; 21209330; 21124976; 20648755; 20455947; 20172033; 20129062; 7523114; 19896487 |
| NT02SS0450 | 13 | 20566627; 10754390; 18824261; 18426477; 17878526; 16982740; 15520025; 1729602; 10376878; 11798802 |
| NT02SS0456 | 13 | 20179335; 18172502; 17393393; 3285220; 15946190; 9207058; 15053877; 15053876; 6390679; 10508412 |
| NT02SS0548 | 8 | 21393235; 21390068; 21365755; 21364953; 21349853; 21349332; 21344599; 21336923; 21319302; 21311031 |
| NT02SS0549 | 8 | 21366264; 21296650; 20134246; 19885726; 19711061; 19694553; 19607794; 19412581; 19344636; 19153769 |
| NT02SS0685 | 8 | 21181421; 21081480; 21068384; 20971856; 20952576; 20876281; 20732359; 20624914; 20622059; 20518024 |
| NT02SS0706 | 4 | 21392491; 21381085; 21376446; 21371610; 21370388; 21367690; 21365918; 21361395; 21359575; 21341701 |
| NT02SS0726 | 13 | 21251324; 21223391; 20163651; 19641118; 15955315; 15560371; 12962325; 11601332; 11545438; 11100899 |
| NT02SS0774 | 13 | 21352546; 21303937; 21278676; 21278155; 21256985; 21219451; 21177880; 21167900; 21159796; 21156960 |
| NT02SS0992 | 13 | 15661851; 18205615; 15649767; 8166633; 14519116; 10496312; 10364253; 10084702; 6251899; 8662789 |
| NT02SS1000 | 2 | 21217007; 21036035; 20601432; 20138927; 390093; 17584754; 14663086; 12864856; 12509268; 11817648 |
| NT02SS13157 | 8 | 9397527; 15066815; 16420634; 16116657; 15901540; 15839679; 12824332; 10888848; 12423369; 8674685 |
| NT02SS1423 | 1 | 21191998; 20364396; 20028400; 19653643; 19382143; 19362563; 19041906; 18997402; 18763167; 18379776 |
| NT02SS17372 | 4 | 21069999; 20851155; 20595383; 20301166; 20154127; 20024173; 7947939; 8876144; 19435287; 3572360 |
| NT02SS1979 | 8 | 21239558; 21059110; 21044611; 20840591; 16775306; 20335176; 20194623; 20138247; 20089804; 19956901 |
| NT02SS2650 | 9 | 20470824; 19170545; 16342138; 15755870; 8631842; 8386358; 2306462 |
| NT02SS2666 | 2 | 16427313; 15380647; 11007789; 10464323; 7606163 |
| NT02SS28095 | 2 | 17335870; 16206477; 12736664; 11816029; 10094680; 9055989; 8640549 |
| NT02SS2944 | 8 | 20885930; 16922514; 16466637; 16423019; 14644435; 11727834; 11293415; 10827091; 10438486; 9395300 |
| NT02SS3035 | 8 | 9884221; 9428682; 9315721; 9298948; 9219517; 8250217; 2119806; 1322173; 3286640 |
| NT02SS3404 | 9 | 20814828; 20404339; 20194276; 20056424; 18775787; 18302342; 17473510; 15610040; 9325256; 17075828 |
| NT02SS3405 | 9 | 20971863; 20932952; 20737255; 20404339; 19653015; 19652394; 19419745; 1556116; 18624305; 17888661 |
| NT02TE0101 | 1 | 20105429; 17204552; 16807285; 16024168; 10573180; 14660177; 14600802; 11739469; 10481272 |
| NT02TE0128 | 8 | 21362485; 21346809; 21335525; 21226334; 21051543; 20979592; 20964819; 20947511; 20944230; 20932060 |
| NT02TE1395 | 17 | 21259411; 21190940; 21155874; 20944236; 20800068; 20471983; 16826544; 16754669; 10227159; 8039908 |
| NT02TE1866 | 2 | 21156797; 21147779; 21071388; 20977213; 20843816; 20690630; 20573177; 20221527; 20219465; 20164445 |
| NT02TE1939 | 17 | 21393242; 21392796; 21390306; 21390260; 21389671; 21388273; 21386978; 21386057 |
| NT02TE2360 | 5 | 21281472; 21224284; 21188624; 21187059; 21123867; 10398147; 20566371; 20554947; 20545621; 20533528 |
| NT02TE2525 | 18 | 21385703; 21303910; 21297161; 21274617; 21245269; 21148420; 21147776; 21111787; 20843792; 20654624 |
| NT02TE2730 | 8 | 21385584; 21369832; 21368150; 21364293; 21361388; 21350473; 21349979; 21348864; 21345474; 21335525 |
| NT02TE2976 | 8 | 20023011; 19693490; 19652345; 19562840; 17971854; 12466265; 13152084; 17706956; 17218476; 17098399 |
| NT02TE3796 | 11 | 20361665; 19948191; 19778558; 16418526; 19593448; 18820295; 19479250; 19365757; 19356121; 19349746 |
| NT02TE3972 | 15 | 21108067; 21098025; 21091513; 20808885; 20525091; 15572774; 20088541; 9437425; 18440816; 15716443 |
| NT02TE3981 | 4 | 21178481; 21162553; 21131490; 2002000; 14527658; 17694049; 18199454; 18001143; 1390772; 10466731 |
| NT02TE4019 | 13 | 21161331; 20949119; 20705584; 12902985; 20552438; 20538039; 3372162; 20477760; 20460582; 20404857 |
| NT02TE4051 | 6 | 20976249; 20525790; 20138014; 19818715; 18820000; 18176792; 12893777; 17397878; 17049268; 16676353 |
| NT02TE4756 | 18 | 20947202; 16639; 3520229; 17684740; 17565601; 10819976; 9888822; 16139519; 15721233; 15363386 |
| NT02TE4860 | 9 | 20398215; 20061799; 12460945; 5940632; 17885949; 10366591; 12140558; 11371521; 15948957; 15659169 |
| NT02TE5279 | 1 | 18679823; 12706338; 11814655; 2657660; 10587438; 9636022; 8418843; 1868065; 3440089; 6120060 |
| NT02TE5455 | 12 | 21394739; 21394574; 21394548; 21393843; 21393179; 21393164; 21392990; 21392598; 21392585; 21392420 |
| NT02TE5705 | 6 | 21291520; 21173184; 21092102; 21045313; 20921460; 20693529; 20691096 |
| NT02TE5760 | 11 | 15387827; 15225314; 9829919; 2548993; 9126837; 7625275; 8021940; 1334530; 1334529; 13278318 |
| NT02TE5915 | 5 | 15269796; 17227754; 16618101; 16099108; 15304505; 15060080; 12907690; 12740361; 12138164; 11375390 |
| NT02TE6292 | 8 | 20880062; 20815363; 20557022; 20112286; 20064075; 2433292; 19114071; 19100254; 17999982; 11157777 |
| NT02TT0012 | 8 | 21305143; 15772818; 12646191; 10024458; 8688087; 9667924; 1633810; 8143744; 8021261; 7878465 |
| NT02TT03173 | 8 | 21394044; 21393444; 21393246; 21393237; 21388872; 21387012; 21384861; 21384803; 21383180; 21382276 |
| NT02TT0419 | 12 | 21035733; 20818668; 20817675; 20639339; 6337632; 20417640; 20142038; 19809258; 19596042; 18713320 |
| NT02TT0553 | 9 | 9283097; 8755745; 7766696; 7756275; 8051146; 8454629; 1879422; 1712230; 13650640; 5826440 |
| NT02TT0570 | 17 | 4868216; 16825789; 15692745; 15185964; 12628255; 11824762; 10360176 |
| NT02TT0800 | 18 | 21085989; 20118641; 9787636; 19168988; 18702072; 18343670; 17251264; 16887318; 16751629; 16730200 |
| NT02TT0828 | 6 | 20457749; 20304994; 20129927; 19094995; 17600070; 10585965; 10498723; 16085468; 10829079; 10508668 |
| NT02TT1010 | 6 | 21084251; 20958054; 20876797; 20852747; 20850478; 20734660; 20695079; 20599907; 20599617; 20523341 |
| NT02TT1031 | 12 | 21031477; 20937841; 20889677; 20536384; 20512923; 20507874; 20485747; 20379219; 20146668; 20140974 |
| NT02TT1060 | 8 | 20876714; 20732359; 20381373; 8385603; 18835244; 18765295; 18649259; 18463234; 17899367; 16228398 |
| NT02TT1073 | 8 | 20532738; 20143161; 20077566; 15865447; 15544340; 15109257; 822747; 12634336; 12560602; 12482588 |
| NT02TT1141 | 18 | 21394800; 21279681; 21196952; 21157892; 20826681; 20739287; 20595047; 14963042; 20530577; 20421303 |
| NT02TT1146 | 4 | 21318355; 21300096; 21289147; 21228486; 21216994; 21211855; 21190733; 21185863; 21130480; 21085993 |
| NT02TT1490 | 18 | 20684226; 20471400; 19877430; 18156677; 19140455; 17923103; 15473317; 15225549; 15215587; 15192097 |
| NT02TT15247 | 8 | 12198487; 17014090; 16005887; 5048285; 15066784; 11571621; 11414329; 9312093; 9249026; 2714278 |
| NT02TT1575 | 12 | 21365832; 21344497; 21297029; 21288331; 21271223; 21269937; 21263404; 21224056; 21209004; 21204688 |
| NT02TT1595 | 18 | 21135361; 21103971; 21103970; 20937802; 20937770; 20857261; 20663969; 20628435; 20614158; 20609358 |
| NT02TT1993 | 2 | 21372092; 21368171; 21357619; 21357486; 21305354; 21285035; 21265779; 21255426; 21231969; 21221062 |
| NT02TTA0091 | 4 | 19214757; 17894815; 17257587; 16569761; 10065837; 11250197 |
| NT03BP0218 | 8 | 14597104; 11876708; 16347774; 9700240; 2020552; 3127651 |
| NT03BP0544 | 1 | 21312326; 21287990; 21261753; 21253822; 21152904; 21152090; 21151916; 21099300; 21082203; 21035079 |
| NT03BP05947 | 8 | 21365653; 21311030; 21310654; 21287365; 21220760; 21183637; 21134392; 21108604; 21080078; 20973793 |
| NT03BP06360 | 8 | 20675489; 20570198; 20022530; 19819302; 19596066; 19383527; 17532339; 18498061; 10913250; 7689641 |
| NT03BP0679 | 9 | 21106133; 20825197; 20463021; 20180811; 20167774; 20035485; 19903863; 19880769; 19659692; 17151231 |
| NT03BP06957 | 8 | 17449009; 2271232; 8645313; 8300532; 16345266 |
| NT03BP0731 | 4 | 20514230; 18317775; 12561874; 10202817; 8220445; 1859868; 16593270; 2129423; 6271763; 6310323 |
| NT03BP08893 | 8 | 21381155; 21372752; 21359568; 21351210; 21344614; 21323897; 21319286; 21311411; 21270794; 21258664 |
| NT03BP1667 | 3 | 20851903; 20653564; 20633230; 6100313; 19892701; 19605459; 19426809; 19153448; 19129644; 19089411 |
| NT03BP1674 | 3 | 19118341; 11454445; 13129961; 14522970; 9364918; 8842709; 8051103 |
| NT03BP1889 | 13 | 18279892; 17329242; 15611111; 12521300; 12009902 |
| NT03BP1940 | 15 | 21375706; 21342532; 21330442; 21325039; 21282650; 21264306; 21245528; 21192786; 21167274; 21149588 |
| NT03BP2060 | 18 | 20163160; 6277502; 18284334; 15527913; 11862463; 10405142; 7608187; 7997159; 8014881; 1762077 |
| NT03BP2816 | 2 | 21368171; 21255426; 21231969; 21206014; 21142117; 21081498; 21072368; 21071388; 21038112; 20971904 |
| NT03BP2839 | 8 | 18673073; 18582433; 16863643; 16299377; 15910742; 15475358; 9987136; 15184552; 14672950; 11017202 |
| NT03BP2875 | 4 | 21221750; 21143936; 14641053; 8900409; 16076113; 15998028; 15740026; 15062777; 14672935; 11542256 |
| NT03BP30397 | 8 | 21310261; 21212464; 21108999; 21056979; 20852029; 20801663; 20715794; 20712647; 20632216; 20605248 |
| NT03BP3108 | 6 | 21394759; 21394438; 21394098; 21392582; 21392201; 21390130; 21389970; 21388962; 21388951; 21386994 |
| NT03BP3358 | 8 | 20392690; 5999; 11016692; 2537825; 7608187 |
| NT03BP3617 | 6 | 20966423; 19897046; 18390561; 18191589; 16060497; 15816370; 10356995; 15324852; 14695982; 12212882 |
| NT03BP3677 | 15 | 21357483; 20133363; 18323620; 16945692; 16204505; 4352175; 18245243; 18093135; 11495988; 10679470 |
| NT03BP3710 | 12 | 21274528; 7337433; 18347791; 9882674; 17720797; 11418562; 17543409; 15489436; 16233560; 3022301 |
| NT03BP38477 | 8 | 20652619; 20640572; 20543072; 20071617; 19577910; 16754867; 19020104; 18473933; 18166197; 17997750 |
| NT03CC01706 | 2 | 15215507; 10681549; 10681549; 10409680; 9144147; 9144197; 7673149; 8336108; 1645618; 2747617 |
| NT03CC0203 | 8 | 17576516; 15388943; 10437801; 7584858; 6546423; 6288108; 6254527 |
| NT03CC0435 | 8 | 21177473; 20960122; 19577535; 10504251; 15951430; 15187270; 14717597; 10521530; 8378356; 12023025 |
| NT03CC0436 | 8 | 20513347; 19892700; 15817393; 19348906; 19348783; 8385603; 18022381; 18513324; 11259585; 17680808 |
| NT03CC0696 | 8 | 21329464; 21206700; 21133623; 21130106; 20878130; 20851451; 20833713; 20687231; 20593958; 20554899 |
| NT03CC0698 | 8 | 20858453; 20826797; 20823090; 20654728; 20643099; 20636270; 20603809; 20571113; 20528953; 20460714 |
| NT03CC0699 | 8 | 19195398; 16157265; 11307840; 11295448; 9585000; 7628554; 8001168; 1494348; 2550657 |
| NT03CC0731 | 3 | 21166998; 21073066; 20937802; 20880933; 20868650; 20805402; 20720015; 20688828; 1987054; 20633230 |
| NT03CC0767 | 6 | 20833188; 12778123; 18804481; 10606644; 9722634; 6288254; 15629722; 15503140; 15113836; 11821933 |
| NT03CC0874 | 13 | 19646947; 7731806; 18318121; 17850309; 17022624; 12003491; 15544333; 11014182; 10997906; 8730873 |
| NT03CC1021 | 6 | 12941704; 10625642; 1400219; 8422961; 2166215; 779788 |
| NT03CC1436 | 11 | 21244827; 21062824; 9858702; 10594823; 10408954; 16173391; 14651626; 10829077; 10542181; 10074068 |
| NT03CC1449 | 1 | 21229398; 19801658; 17693399; 14659018; 17557331; 10454541; 16982615; 12699629; 15211526 |
| NT03CC14535 | 8 | 21366273; 20973554; 20875391; 20521767; 20513956; 20408539; 9268671; 20182527; 20116192; 20112981 |
| NT03CC1466 | 2 | 20693681; 19766415; 17123542; 7984417; 8475124; 15855032; 11073950; 10912697; 10852929; 10842591 |
| NT03CC1484 | 8 | 11768747; 7891558; 7891558; 8130227; 8137905 |
| NT03CC1665 | 6 | 21343455; 21309064; 21300434; 21255919; 21219391; 21158931; 20977136; 20930243 |
| NT03CC2137 | 12 | 21326357; 21289038; 21148698; 21131519; 21125380; 20847219; 20812717; 20599707; 20572939; 20487024 |
| NT03CC2177 | 17 | 8034727; 18182386; 11700359; 10689164; 10206709; 9851041; 9387241; 8034727; 8034728; 8034727 |
| NT03CC2182 | 8 | 15544340; 12634336; 9287422; 222744; 11132640; 11054105; 10968624; 8880899; 10611454; 9639604 |
| NT03CC2197 | 8 | 20975997; 20433942; 19428325; 19217306; 16288777; 17668236; 16545948; 15799962; 15738637; 11768297 |
| NT03CC2207 | 1 | 17956427; 11440130; 10220897; 8290655; 942051; 3003049 |
| NT03FN0234 | 14 | 5321403; 17725561; 15256571; 1703758; 10064693; 4554017; 7500333; 8535162; 7517939; 8433966 |
| NT03FN0243 | 17 | 21382107; 21371479; 21316374; 21205867; 21143318; 20971916; 20952386; 20730534; 20700605; 9446751 |
| NT03FN0675 | 14 | 21251176; 20807196; 20724861; 20626025; 20600266; 20464463; 20424309; 20226779; 20214047; 11099506 |
| NT03FN0772 | 12 | 21390545; 21385872; 21341524; 21324345; 21047126; 20977208; 20861513; 20829225; 20828565; 20733120 |
| NT03FN1846 | 8 | 20493164; 20117074; 10339593; 19128036; 12730198; 18486594; 17681940; 16760472; 16042611; 15576052 |
| NT03FN1849 | 8 | 21329181; 21167537; 20876714; 20858453; 20643099; 20625049; 20613764; 20528953; 20459120; 20454804 |
| NT03LI0221 | 3 | 21385270; 21198360; 21178498; 21146533; 21040511; 20965199; 20851903; 20850455; 20838242; 20713621 |
| NT03LI0237 | 8 | 21181421; 21179059; 21148472; 21081480; 21068384; 21041373; 20971856; 20959514; 20952576; 20876281 |
| NT03LI0419 | 12 | 21338660; 20196537; 19702337; 17460661; 17689904; 17561415; 17383769; 16527306; 15081283; 9748450 |
| NT03LI0574 | 4 | 21282456; 17425668; 16390454; 16203150; 15995642; 15383161; 14678166; 12209001; 10582867; 9837987 |
| NT03LI0575 | 18 | 21350490; 21249122; 21187401; 21035449; 20981744; 20865003; 20600546; 20111865; 11842121; 19778646 |
| NT03LI0587 | 11 | 21224843; 20067338; 20363791; 11470852; 17251179; 19416360; 19286454; 18644963; 18474594; 10390637 |
| NT03LI0757 | 4 | 20065107; 19308703; 14980210; 15128517; 12140549; 12865312; 12805238; 11352915; 11145900; 9497382 |
| NT03LI0816 | 8 | 21378103; 21365448; 21303534; 21302592; 21295140; 21250997; 21194154; 21145387; 21107688; 21103453 |
| NT03LI0823 | 8 | 21391497; 21384159; 21382338; 21368117; 21348614; 21344856; 21342604; 21329214; 21324314; 21301872 |
| NT03LI0941 | 4 | 20304988; 19953638; 16090580; 18334551; 10995478; 17924527; 8550421; 11934612; 12672126; 10493827 |
| NT03LI1027 | 12 | 20696584; 19453269; 11836320; 19123976; 18992253; 9700155; 11223883; 2200384; 18063795; 18008160 |
| NT03LI1173 | 9 | 21362022; 21352884; 21106133; 20966403; 20843517; 20684826; 20625650; 18824113; 20523008; 20404512 |
| NT03LI1299 | 6 | 21306445; 21305083; 21264264; 21227759; 21172664; 21129204; 21107010; 21102445; 21098121; 21081488 |
| NT03LI1345 | 15 | 21385202; 21217003; 20942908; 20869221; 20480360; 20378989; 20205655; 18761686; 20081031; 19958380 |
| NT03LI1387 | 12 | 21382117; 21375692; 21365649; 21352096; 21350674; 21315759; 21307265; 21304989; 21297495; 21264226 |
| NT03LI15505 | 8 | 21294903; 20687969; 20637321; 20434185; 20371699; 20163621; 20154153; 20036965; 20024786 |
| NT03LI1813 | 12 | 21285323; 21277928; 945867; 20472716; 20171266; 16859525; 20061819; 19909369; 19789264; 19762710 |
| NT03LI1924 | 12 | 20600124; 8244018; 2254289; 12094726; 10368140; 10231573 |
| NT03LI2055 | 4 | 20203055; 20178785; 18667558; 18553406; 1764974; 2744846; 15819626; 12730325; 7806390; 11931544 |
| NT03LI2080 | 6 | 21388532; 21365542; 21350762; 21343909; 21336027; 21325134; 21321231; 21091440; 21086493; 21078962 |
| NT03LI2522 | 18 | 19840771; 2394664; 7836312; 7984417; 18385266; 18216137; 17581921; 2273446; 16030212; 16790504 |
| NT03LI2849 | 12 | 20002189; 2684968; 18045386; 15223057; 17005379; 12975369; 12584195; 11544189; 10520736; 10497172 |
| NT03LI2928 | 4 | 21108067; 21098025; 21091513; 20832320; 20808885; 20543066; 20525091; 17582720; 20487303; 20418388 |
| NT03LI3208 | 9 | 20594840; 20590527; 20562282; 20557983; 20547590; 20418430; 20370610; 20304657; 20221630; 20178986 |
| NT03LI3280 | 6 | 21229971; 20852270; 20822508; 20693529; 20656393; 20622503; 20586875; 20599730; 20587501 |
| NT03LI3756 | 12 | 13994204; 12676964; 10972815; 9737856; 9737855; 9108259; 8706922; 7866749; 2874557; 6096384 |
| NT03LI39667 | 8 | 19635449; 7022156; 19193103; 18542924; 18355280; 18338382; 17431803; 16962159; 11834298; 15531590 |
| NT03LP0003 | 6 | 20465561; 15065656; 16087733; 17364684; 10498743; 16824104; 11024023; 10390814; 9990858; 10336423 |
| NT03LP0010 | 12 | 20400571; 19824612; 19787775; 19623961; 18957606; 17646390; 18957606; 7984093; 8462872 |
| NT03LP0120 | 8 | 21047519; 9838063; 19689132; 16849792; 16377208; 14579184; 15102091; 14732287; 11940577; 12428268 |
| NT03LP0443 | 9 | 16936700; 10489373; 2801678; 17289852; 10362509; 12223468; 9085464; 8658037; 7531490; 8301317 |
| NT03LP0449 | 15 | 21125655; 20809954; 20708792; 20601375; 20301200; 16374707; 9126178; 20021432; 19861152 |
| NT03LP0571 | 1 | 21261753; 21255725; 21068339; 21055403; 20921361; 20808573; 20695527; 20661733; 20653567; 20620562 |
| NT03LP0624 | 14 | 20544509; 20382023; 20380929; 17701900; 19918830; 19754463; 19186537; 19161981; 19131690 |
| NT03LP0640 | 9 | 21394808; 21393488; 21393229; 21389119; 21386030; 21385862; 21377632; 21366233; 21361911; 21361295 |
| NT03LP0712 | 3 | 21311024; 21097635; 21078967; 20808760; 20807236; 20807222; 20796283; 20722599; 12900386; 20656905 |
| NT03LP07924 | 8 | 20703955; 20544973; 20519913; 20462210; 20031435; 19962718; 19824396; 19710471; 19556747; 19339078 |
| NT03LP09645 | 8 | 21097593; 20600382; 20167619; 10898862; 19725101; 18442171; 16339947; 8958153; 16678117; 16599556 |
| NT03LP1012 | 12 | 21394783; 21394616; 21393833; 21392515; 21391700; 21389626; 21389108; 21388959; 21388872; 21386893 |
| NT03LP1070 | 12 | 11083876; 827241; 19447052; 12815105; 15452063; 15843469; 18778119; 18601269; 11514191; 6442210 |
| NT03LP1071 | 6 | 20936072; 15020458; 20441441; 10430871; 16793381; 9406544; 16196125; 14573598; 11835278; 9822387 |
| NT03LP1072 | 6 | 15020458; 15621460; 14739987; 12717778; 9822387; 11341996; 11092836; 10753775; 10731413; 9925794 |
| NT03LP1075 | 6 | 21362621; 21342654; 21342128; 21339608; 21335605; 21333363; 21332166; 21276096; 21266475; 21263027 |
| NT03LP1237 | 15 | 20718459; 18724706; 17709748; 18313075; 17481759; 11872841; 16794934; 15629915; 9493270; 15728912 |
| NT03LP1261 | 4 | 21273249; 21109564; 19919668; 19383688; 8071222; 18719175; 10995478; 17768253; 17875662; 10516219 |
| NT03LP1322 | 12 | 21327044; 21204920; 21069910; 20959450; 20953507; 20947023; 20942652; 20581826; 20581825; 20515934 |
| NT03LP1399 | 14 | 11596651; 3053713; 18393820; 18387370; 17397129; 16549409; 16284928; 16280320; 12427759; 12068013 |
| NT03LP1467 | 2 | 20713166; 20565114; 1678137; 11500486; 11322938; 10966576; 10333520; 10089457; 8591031; 4598072 |
| NT03LP1484 | 18 | 20089863; 10700280; 10511906; 10422227; 9614079; 1424783 |
| NT03LP15470 | 8 | 21332407; 9212098; 18164639; 17923481; 7569993; 17182197; 15522269; 15082001; 14992577; 12761172 |
| NT03LP1572 | 9 | 21259003; 19740749; 19151095; 9370328; 17132865; 10858449; 16664382; 11560410; 10858449; 9370337 |
| NT03LP1576 | 12 | 21246236; 21192783; 21039608; 20950979; 20933611; 20886095; 20803144; 20720111; 20696832; 20686217 |
| NT03LP1597 | 11 | 21262357; 20562304; 20498309; 20182770; 20061472; 19332822; 20948648; 16381971; 18645960; 18280236 |
| NT03LP1631 | 13 | 18854334; 17584609; 16496097; 16144421; 15381408; 15380178; 14568070; 12620343; 12620338; 12375903 |
| NT03LP1705 | 12 | 21298394; 21219404; 21152005; 21119035; 21119035; 21119037; 21070406; 20981700; 20891022; 20843040 |
| NT03LP1737 | 13 | 21327447; 21272955; 21220307; 21219871; 21217007; 21082745; 20943400; 20888319; 20882017; 20876530 |
| NT03LP1748 | 12 | 20482315; 20196774; 20388122; 20189678; 20070259; 19942855; 17523140; 19783655; 8625414; 19688213 |
| NT03LP1783 | 4 | 2126155; 19605463; 19497327; 18451048; 17660418; 15629949; 16428326; 10692152; 15946806; 15130117 |
| NT03LP1788 | 4 | 18753783; 15817382; 10712687; 7601828; 10536136; 2407720; 2404281; 2002011; 1999391; 8415608 |
| NT03LP1789 | 4 | 20439729; 20133562; 19858188; 1447979; 10468575; 17085552; 17015641; 10731410; 16677309; 11591685 |
| NT03LP1841 | 6 | 21211720; 21209221; 21084480; 21078300; 21070792; 20971895; 20937877; 20885787; 20864032; 20851996 |
| NT03LP1872 | 6 | 21241707; 20692326; 20022231; 19586858; 19268511; 19066587; 18848986; 18370075; 17287355; 17294253 |
| NT03LP1943 | 12 | 17047052; 14659042; 12039036; 10631378; 1312681; 9646468; 7542550; 9314606; 9037761; 1339443 |
| NT03LP2101 | 17 | 10937989; 10656815; 16630817; 15654752; 12324462; 9094652; 10481020; 9836593; 1537828; 8037924 |
| NT03LP2191 | 9 | 21184844; 21173151; 21148740; 21090716; 20804717; 20696404; 20519401; 20215862; 20107206; 20097939 |
| NT03LP2230 | 8 | 21318893; 21318888; 21268586; 20839014; 20684609; 20606294; 19288015; 19567159; 19097065; 18757054 |
| NT03LP23457 | 8 | 21199677; 21140195; 21037206; 20861395; 2189777; 20622436; 20599829; 20570663; 20558818; 20484858 |
| NT03LP2722 | 4 | 21301106; 20586476; 20132451; 20118266; 20026337; 19732341; 8071222; 19332819; 14960570; 18424518 |
| NT03LP2790 | 15 | 21060253; 19602144; 20367474; 20089046; 20048056; 19843219; 19271532; 19191877; 7604262; 18565138 |
| NT03LP2815 | 12 | 19913481; 19241474; 17241197; 16805830; 12888578 |
| NT03LP2919 | 17 | 21349974; 21344039; 21324911; 21324338; 21320509; 21299217; 21151174; 21130834; 21126315; 21075929 |
| NT03LP2930 | 8 | 20025615; 15943805; 10987141; 8797851; 2256929 |
| NT03LP2933 | 8 | 20056102; 19904590; 19638432; 18358763; 17634376; 10952301; 17244482; 16756485; 16098512; 15898115 |
| NT03LP3052 | 13 | 21295137; 21282428; 21175197; 21144880; 21042822; 20954242; 20796028; 11342140; 20512599; 20510245 |
| NT03LP3152 | 4 | 1374685; 2233248 |
| NT03LPA0109 | 1 | 20064065; 17348838; 10222271; 17020768; 15358538; 8486283; 12054870; 11985590; 2674899; 1849480 |
| NT03MT0013 | 15 | 21184218; 3313728; 2040302; 13331868; 7476184; 7584049; 8000527; 1334233; 2287279; 3062173 |
| NT03MT0038 | 8 | 11502197; 10563813; 9490067; 8305427; 8537366; 8037208; 1496922; 2822716; 3571202; 2416750 |
| NT03MT00442 | 4 | 17518353; 3413113; 10781617; 10406097; 9249951; 1614514; 1823163; 2330031; 2643474 |
| NT03MT0060 | 3 | 20865175; 20210661; 11320139; 15687380; 18173801; 17426021; 12468728; 15032825; 14997492; 12421307 |
| NT03MT0102 | 5 | 20736171; 7836277; 17360677; 18976214; 18727912; 18706514; 18155044; 17785198; 17294328; 16905100 |
| NT03MT0247 | 13 | 21256985; 21177880; 21062819; 20639535; 18400176; 19006815; 11340196; 11462827; 18047567; 17895579 |
| NT03MT0254 | 8 | 21339825; 21193400; 20610779; 20156168; 18651753; 20056102; 20025615; 19904590; 19664596; 19638432 |
| NT03MT0330 | 15 | 21331046; 21276004; 21209283; 21170333; 21150289; 21106671; 21098220; 21081493; 21079790; 21062824 |
| NT03MT0410 | 6 | 10713083; 12066890; 9163424; 1508679; 9544702; 8001558; 1651938 |
| NT03MT0924 | 15 | 18662309; 17628154; 16825793; 10972836; 10931278; 8999810; 10383987; 9047301; 10972837; 1320611 |
| NT03MT1089 | 8 | 18651753; 18358763; 10952301; 16569397; 12678433; 12615344; 12515529; 11888296; 11295131; 11222389 |
| NT03MT1092 | 8 | 20610779; 10952301; 12758076; 12678433; 10987141; 8536688; 2679883 |
| NT03MT1184 | 1 | 21392498; 21385839; 21377655; 21371467; 21366658; 21348380; 21342491; 21332407; 21325018; 21324900 |
| NT03MT15461 | 8 | 20519568; 20447408; 20299676; 19942659; 19923219; 19859980; 19850285; 18795799; 12206759; 18428423 |
| NT03MT1615 | 8 | 21357619; 20960122; 20673834; 20538584; 20513347; 20498375; 20493164; 20117074; 20091229; 19879892 |
| NT03MT1760 | 15 | 20860483; 20453099; 20435045; 19789137; 19661178; 18957436; 18820688; 18725229; 3018721; 2025413 |
| NT03MT1797 | 11 | 21145325; 21124229; 21097709; 20977258; 20975831; 20974536; 20944016; 20890269; 20846395; 20844938 |
| NT03MT1816 | 15 | 10784047; 10972795; 1444391; 9642094; 6803114 |
| NT03MT1858 | 3 | 20959463; 20816176; 20447077; 20435300; 20335257; 20299332; 20147708; 20096828; 20006464; 19955174 |
| NT03MT1882 | 6 | 10745045; 11418610; 10393201; 14235546; 9857048; 14021492; 1561835; 4884815; 8404370 |
| NT03MT19937 | 8 | 21281738; 21144838; 21070510; 20958327; 20828554; 20828325; 20724146; 20676633; 20560783; 20535465 |
| NT03MT2149 | 6 | 20667914; 17542913; 16055739; 16947863; 16826228; 11331764; 8870499; 12853965; 11412842; 10801852 |
| NT03MT2371 | 18 | 21272566; 21191998; 20736162; 20445230; 20435740; 19568767; 19405093; 19350404; 10849575; 17784761 |
| NT03MT2386 | 18 | 21170889; 21257749; 21130726; 20865662; 20860483; 20845974; 20703215; 20610529; 20685824; 20594941 |
| NT03MT2413 | 11 | 15758242; 15581892; 11043984; 11827961; 10669597; 10092658; 8029014; 3011407; 8226765; 8867465 |
| NT03MT2509 | 6 | 20179150; 16109960; 11743002; 15037779; 17005014; 15201048; 15088388; 10413481; 9353259; 8692273 |
| NT03MT2518 | 6 | 21365490; 21364738; 21301438; 20805246; 19747545; 20298193; 20184309; 18835275; 19747545 |
| NT03MT2562 | 18 | 20930065; 20463172; 20588286; 20442269; 20400641; 20385770; 20223824; 20112249; 19864301; 19636250 |
| NT03MT2564 | 15 | 20415666; 19965983; 19395495; 18784352; 18055461; 16421216; 14531828; 12444019; 1662683 |
| NT03MT2715 | 12 | 20460432; 20419402; 6183169; 19703106; 19054387; 17533641; 9847218; 18179254; 9723928; 9988747 |
| NT03MT2716 | 6 | 20609918; 20154126; 19572524; 19383722; 19306853; 19303395; 18591783; 17264080; 15805601; 11844990 |
| NT03PA0125 | 8 | 21324314; 21042417; 20924414; 20854917; 20737854; 20714159; 20696095; 20691393; 20660112; 20627424 |
| NT03PA0276 | 1 | 21150112; 20815012; 20445226; 19911771; 19856378; 19816720; 18614239; 18931429; 18540103; 18200648 |
| NT03PA0404 | 12 | 20711794; 19170879; 14999401; 10749923; 16380269; 11114201; 2025413; 12939276; 12914946; 12886009 |
| NT03PA0494 | 18 | 20875141; 20691393; 20660570; 20617462; 20547750; 20522709; 20456012; 20398062; 20345514; 20192919 |
| NT03PA0503 | 8 | 21221659; 21176883; 20923787; 20851234; 20836999; 20732827; 14691600; 20507258; 20486657; 20437086 |
| NT03PA0558 | 2 | 21366233; 20961145; 20937134; 19821612; 12873140; 19736993; 19438611; 19328201; 19362514; 19245793 |
| NT03PA0844 | 17 | 20965918; 11673434; 16699585; 10792721; 9387225; 8550474; 8086465; 8188579 |
| NT03PA0863 | 18 | 19366604; 18692508; 18156179; 16722231; 12923181; 11756453; 11248195; 11248194; 10521259; 8045426 |
| NT03PA0960 | 1 | 19731276; 18705875; 15889412; 15043864; 15016471; 12768628; 12667063; 11294630; 11112527; 10671523 |
| NT03PA0980 | 18 | 21322734; 20720170; 20695527; 20652610; 20600019; 9500796; 20558222; 20441173; 20430034; 20399792 |
| NT03PA1028 | 15 | 21108067; 21097618; 21078995; 21050859; 21037181; 20979345; 20971918; 20966074; 20951027; 20946859 |
| NT03PA1047 | 6 | 21354425; 21350489; 21327042; 21235642; 21123069; 21097613; 21035377; 20890835; 20889742; 20843371 |
| NT03PA1185 | 4 | 15136044; 11327763; 11554792; 6394719; 10712687; 9161424; 1551848; 1905667; 2181149; 13770074 |
| NT03PA1189 | 4 | 17090391; 15136044; 11327763; 1474584; 1551848; 2181149; 13770074; 2129540 |
| NT03PA1194 | 4 | 20586063; 8071222; 19351587; 19153448; 8765301; 17283383; 14617143; 11327763; 12401220; 11554792 |
| NT03PA1201 | 4 | 21334822; 21273249; 21204905; 21041491; 20592113; 20555353; 20368700; 20305062; 20221731 |
| NT03PA1203 | 4 | 21143315; 16487743; 11298291; 18387898; 17433506; 10063642; 11934566; 15673516; 9140922; 1323536 |
| NT03PA1210 | 3 | 6394719; 10712687; 2056133; 8415608; 2991190 |
| NT03PA1215 | 4 | 21278755; 16487743; 20421493; 10785634; 20118266; 19260965; 19153455; 16677309; 16677309; 15170399 |
| NT03PA1271 | 4 | 21190121; 21189321; 20528877; 20594290; 8985182; 20071053; 18425112; 19184118; 18398003; 17718161 |
| NT03PA1483 | 8 | 18310026; 17116585; 12610720; 14624355; 12610720; 11755201; 10684596; 10499283; 9020792; 15299349 |
| NT03PA1525 | 9 | 20418430; 20178986; 19664929; 19472174; 2580220; 19095065; 19083031; 17555433; 18824113; 18721141 |
| NT03PA1702 | 14 | 21361328; 21257770; 21257642; 21219207; 21131548; 21059659; 20952484; 20877497; 20842824; 20829075 |
| NT03PA1707 | 4 | 21335646; 21216997; 21082789; 20345660; 20132438; 2002065; 19697711; 19571680; 15916962; 17328675 |
| NT03PA1778 | 18 | 20028004; 18378356; 18217702; 17980698; 17715404; 17504964; 17504265; 17197568; 17107928; 16684854 |
| NT03PA18037 | 8 | 21324916; 21296913; 21275244; 21085948; 21076104; 21072064; 21036936; 20965335; 20951028; 20949922 |
| NT03PA1867 | 15 | 21296958; 20965199; 20870760; 17005013; 20547748; 20529681; 20497331; 20337713; 19923747; 16923903 |
| NT03PA1995 | 13 | 21175197; 20943400; 20617848; 20601684; 20553774; 20515683; 19879905; 18957446; 17533454; 19481543 |
| NT03PA2150 | 13 | 15540167; 15528667; 12185248; 9733638; 3305487; 3856321; 3911025; 6376124; 6345153 |
| NT03PA2462 | 15 | 11956233; 19168385; 16543507; 16039071; 12788781; 10390515; 15490970; 15192021; 13278318 |
| NT03PA2555 | 15 | 21150515; 11303179; 20581202; 10399323; 20357257; 20085294; 19925818; 19826311; 19505494; 19393677 |
| NT03PA2575 | 3 | 16495632; 15802168; 15556765; 3259855; 9618443; 1083199; 11332764; 11304990; 1510447; 3545068 |
| NT03PA2866 | 8 | 8278350; 5048285; 9190809; 10438748; 11390387; 9634695; 9573183; 8002568; 1367414 |
| NT03PA2986 | 12 | 19650643; 10485712; 10383442; 9428517; 9601038; 9067252; 7713911; 8027082; 2197275; 3049606 |
| NT03PA3051 | 12 | 21168885; 20829508; 20515676; 20022338; 19896503; 19642226; 19220391; 19202909; 19141580; 18937524 |
| NT03PA3329 | 2 | 9331403; 9224567; 1731915; 8405386; 6167991 |
| NT03PA3352 | 18 | 19921465; 18359765; 17850814; 17074904; 11318367; 10866829; 9873010; 5432063; 9268321; 1625313 |
| NT03PA3419 | 3 | 20716633; 20690587; 20648548; 20634336; 20619832; 20538863; 20336367 |
| NT03PA3557 | 9 | 21377632; 21034488; 20622065; 16121256; 19395506; 18032383; 18407998; 18171025; 15904873; 15705744 |
| NT03PA3631 | 3 | 20979470; 21271486; 21254748; 21229813; 21190454; 21181614; 21178087; 21169504; 21168882; 21163439 |
| NT03PA3658 | 8 | 20383025; 20363943; 18997324; 18838820; 18186475; 16614860; 17641651; 17290794; 16834328; 16403639 |
| NT03PA3726 | 4 | 19680248; 11430835; 20132438; 19654604; 15916962; 19019154; 18976281; 17287984; 12754232; 16322744 |
| NT03PA3727 | 4 | 19680248; 11430835; 20132438; 19708980; 19654604; 15916962; 19019154; 19010291; 18976281; 18930010 |
| NT03PA3741 | 12 | 19889088; 7763298; 15047720; 14760686; 14637004; 12177052; 11351281; 11172723; 10715137; 9383148 |
| NT03PA3842 | 3 | 20506248; 17455913; 17316025; 17241201; 15932222; 15823050; 15264802; 13129607; 10623846; 11313358 |
| NT03PA3875 | 15 | 1906870; 19959660; 10708652; 10508151; 8496184; 2673534; 14455023; 2513374; 2495988; 6440948 |
| NT03PA4007 | 18 | 10049997; 19480561; 12811560; 10995844; 16936709; 10993891; 16445940; 16171403; 10361139; 15541891 |
| NT03PA4175 | 17 | 21143318; 20979331; 20935100; 20862323; 20730534; 20633642; 20583596; 20230052; 17075066; 19942657 |
| NT03PA4404 | 1 | 20937239; 20923776; 20720017; 20658158; 20550197; 20529854; 15375207; 20379751; 20179139; 20164178 |
| NT03PA4772 | 8 | 21382015; 21359512; 21357486; 21356170; 21348864; 21322643; 21315567; 21309511; 21307643; 21307642 |
| NT03PA4894 | 17 | 21058056; 21050945; 20929722; 20861329; 20851995; 20829390; 20817101; 20815816; 21088140; 20724091 |
| NT03PA4895 | 13 | 21156135; 7689052; 17346074; 6355760; 11976723; 11063598; 2110477; 2438418; 7916699; 1812070 |
| NT03PA4901 | 13 | 20176963; 19807034; 12773575; 15525707; 4273819; 16813165; 14729335; 10876152; 9538206; 9013882 |
| NT03PA4907 | 13 | 21262594; 18198843; 16861792; 11470155; 10361087; 2461520; 2461735; 3526091 |
| NT03PA4913 | 13 | 20837752; 10937989; 11952893; 16318913; 11483524; 15379729; 15135500; 12225755; 11511371; 4620021 |
| NT03PA4914 | 13 | 12824352; 10368156; 8251501; 8405418; 2207142; 3291949; 2438658; 3691516; 6818528 |
| NT03PA4924 | 13 | 17967466; 18726268; 12411440; 11823215; 17512991; 15009191; 14729335; 11863397; 8529646; 2455872 |
| NT03PA50791 | 2 | 20876192; 20180265; 19636932; 18726075; 18391442; 17305325; 10222271; 17229734; 16884311; 16042595 |
| NT03PA5092 | 4 | 20595383; 20460179; 20363951; 20345660; 19680248; 9829918; 2002065; 19737354; 19635793; 16146521 |
| NT03PA5148 | 17 | 20116460; 18977359; 17504491; 14529615; 16943044; 11179370; 17768253; 7975855; 17090527; 9148780 |
| NT03PA5163 | 4 | 21275995; 21129373; 20643083; 20417607; 20385099; 20298971; 20178826; 20015995; 16540510; 19804717 |
| NT03PA5224 | 3 | 8550520; 9224878; 8899718; 7642131; 10200977; 1588814 |
| NT03PA5268 | 13 | 21352546; 21330151; 20876569; 20830302; 20408996; 19995735; 18362051; 19763328; 19729310; 11795897 |
| NT03PA53612 | 8 | 21273120; 21209090; 21178163; 20433942; 19581367; 19701717; 19564154; 18482575; 16267304; 18358763 |
| NT03PA5433 | 3 | 21390233; 21379570; 21301102; 21294378; 21276045; 21266781; 21255117; 21220359; 21216228 |
| NT03PA5461 | 6 | 9866731; 10830496; 1512194; 8168923; 2853609 |
| NT03PA5624 | 5 | 21295415; 20606272; 2211515; 14981304; 14766306; 8648119; 10194859; 8042900; 1510567; 13785321 |
| NT03PA5715 | 13 | 17188032; 11111046; 9148919; 12226194; 3017694; 2985547 |
| NT03PA5908 | 9 | 21253498; 21036334; 20942458; 20834152; 19996150; 19332293; 19324564; 6997270; 18553138; 18400514 |
| NT03PA6051 | 3 | 21257657; 21094360; 21045169; 21040085; 21037009; 21032867; 20962519; 20870710; 20849910; 20385997 |
| NT03PA6080 | 15 | 2965141; 18277681; 1729202; 9218420; 7968519; 2493441 |
| NT03PA6118 | 6 | 19208629; 14997524; 11839499; 1706458; 1657596; 10195265; 8289342; 9425627; 8504929; 2145445 |
| NT03PA6123 | 8 | 20460724; 20154126; 18757819; 17657404; 15107237; 14977570; 12829696; 9613842; 9558336; 2843169 |
| NT03PA6144 | 6 | 19414020; 10652786; 11350954; 10652786; 3517933; 1097700; 7716189; 1374847 |
| NT03PA61471 | 8 | 15336422; 8565998; 12397015; 7488207; 10231382; 8188595; 10548468; 10481091; 10209766; 9404503 |
| NT03PA6280 | 8 | 20676631; 20608171; 20453146; 19777228; 19513611; 19443543; 16225031; 18456694; 18050911; 17640093 |
| NT03PA6403 | 8 | 21305254; 21278125; 21206008; 21118674; 20426663; 20416275; 20374939; 20372025; 20103585; 19906644 |
| NT03PA6404 | 8 | 21303534; 21209382; 21203410; 21082238; 21080726; 20931991; 20885945; 20859605; 20847343; 20811295 |
| NT03PM0292 | 13 | 20339348; 18832162; 14580344; 17172598; 16407186; 16407185; 14653819; 12941940; 10580496; 11750664 |
| NT03PM0454 | 8 | 21378103; 21365448; 21303534; 21250997; 21145387; 21114360; 21107688; 21092633; 21072576; 21036124 |
| NT03PM0472 | 2 | 18391406; 16866557; 15522295; 10400331; 9665173; 9660189; 8200543; 1451790 |
| NT03PM05635 | 8 | 19006326; 16755018; 16487060; 12602881; 10811655; 8220443; 9108142; 8001171; 2747617 |
| NT03PM1393 | 15 | 21234750; 21205103; 21166709; 21131523; 21131498; 21131490; 21087643; 21070666; 21057009; 21044320 |
| NT03PM1437 | 4 | 21311889; 21258765; 21239495; 21194629; 21117312; 21035278; 21034809; 20936826; 20930278; 20822460 |
| NT03PM1658 | 4 | 19632071; 16368958; 18424185; 14766905; 9973330; 11929561; 9076508; 10642174; 9989641; 21374488 |
| NT03PM1965 | 18 | 21204798; 20650859; 20519092; 20421284; 20411246; 20398785; 20219637; 19574231; 19393632 |
| NT03PS0069 | 4 | 21911488; 21909833; 21905122; 21904561; 21901594; 21899768; 21895868; 21887656; 21883399; 21880724 |
| NT03PS0111 | 18 | 21310763; 21170868; 21229607; 21222360; 21194371; 21190954; 21175743; 21173115; 21151119; 21135361 |
| NT03PS0142 | 9 | 15221226; 12680762; 1459244; 11099866; 10991936; 10945972; 10427752; 7607251; 6345794 |
| NT03PS0255 | 13 | 21374590; 21338421; 21323347; 21283685; 21276099; 21081952; 21036147; 20943400; 20931261; 20882017 |
| NT03PS0309 | 2 | 21394827; 21392185; 21391682; 21388192; 21382013; 21375592; 21374977; 21372787; 21371431; 21370603 |
| NT03PS0577 | 12 | 19359320; 17650073; 10700269; 16963780; 16568094; 11752255; 2670909; 12519969; 9697417; 14684302 |
| NT03PS0632 | 17 | 21345171; 20602357; 9426123; 19000817; 18280161; 10383769; 16631197; 8902817; 12029062; 14712722 |
| NT03PS0635 | 13 | 20399793; 20194507; 10679383; 17929882; 15723055; 15317997; 15199170; 17095013; 12859903; 16280383 |
| NT03PS0714 | 12 | 21210816; 21204251; 20012716; 19855938; 19683447; 19377830; 7777546; 20483277; 17203291; 16965839 |
| NT03PS0759 | 12 | 21195874; 21038355; 20960858; 20846255; 20840788; 20813067; 20604722; 20601447; 20589490; 20586028 |
| NT03PS0810 | 1 | 21332407; 21325018; 21277878; 21215796; 21199370; 21167847; 21163614; 21159064; 21147838; 21143761 |
| NT03PS0842 | 8 | 20632185; 15221385; 16659749; 10024971; 9701571; 7504555; 8325040; 2821010; 2903155 |
| NT03PS0902 | 12 | 20870712; 20857228; 20679221; 20564550; 19921250; 19801578; 19371592; 19245333; 11788305; 18980745 |
| NT03PS0973 | 3 | 20971912; 20675476; 19727946; 18192383; 19021569; 18947195; 16788171; 17609140; 17561945; 17345077 |
| NT03PS1178 | 12 | 21255864; 21148205; 21121896; 21094141; 21045315; 20973967; 20937829; 20880334; 20855515; 20833800 |
| NT03PS1279 | 4 | 15715170; 15493818; 9618448; 11064204; 9988474; 9043132; 8961550; 8709840; 7557417; 3856690 |
| NT03PS1403 | 12 | 9685993; 1671040; 7819328; 1672532; 1733942; 2293021; 3059494 |
| NT03PS1459 | 12 | 21351069; 21335373; 21152870; 20978007; 20933205; 20735721; 20690821; 20659464; 20600941; 20595383 |
| NT03PS1516 | 11 | 21145349; 20889746; 20880145; 20802082; 20675473; 19804621; 20522498; 20511430; 20499648; 20487027 |
| NT03PS1634 | 6 | 17259630; 10972811; 10655484; 56212; 1870971; 1551591; 8733229; 387806; 7517487; 2668183 |
| NT03PS1719 | 8 | 21384760; 21378254; 21378103; 21377371; 21368144; 21365448; 21365190; 21356170; 21342604; 21326867 |
| NT03PS2069 | 12 | 21383996; 21354258; 21349972; 21321956; 21294446; 21218443; 21190945; 21182350; 21166646; 21154597 |
| NT03PS2077 | 12 | 21081501; 20973156; 20876579; 20868353; 14696973; 20541397; 20506400; 20352463; 20347720; 20337595 |
| NT03RE0038 | 5 | 20194510; 19254800; 16815502; 17591443; 15935411; 11278418; 11117877; 14530966; 12735718; 7789538 |
| NT03RE0074 | 15 | 20512483; 16553876; 12867744; 10089316; 12432961; 12398213; 10094672; 9593300; 11226872; 1549509 |
| NT03RE0077 | 15 | 19204900; 9190831; 14766923; 14680944; 14636056; 10931325; 8211183; 1846779; 8875940; 8757734 |
| NT03RE01417 | 8 | 17090920; 8132157; 11073918; 11459836; 11081795; 10537203; 9611813; 9367878; 9299451; 7737515 |
| NT03RE01727 | 8 | 17508726; 16804169; 14742434; 10416260; 9722670; 9303884; 8486638; 344137 |
| NT03RE0254 | 4 | 20118256; 10447890; 14617184; 8808934; 11082305; 14171462; 1846779; 291033 |
| NT03RE0278 | 1 | 21390126; 21368056; 21355420; 21352907; 21346120; 21342468; 21319459; 21292342; 21283532; 21281601 |
| NT03RE0566 | 3 | 21256201; 18776014; 19556347; 8845350; 8330261; 5432063; 2898203 |
| NT03RE0762 | 3 | 19486161; 10947842; 16856943; 15100690 |
| NT03RE0834 | 3 | 21377170; 21362626; 21349544; 21346816; 21340723; 21329659; 21327158; 21324893; 21317879 |
| NT03RE0930 | 3 | 21057010; 20799091; 20595390; 20459596; 8862584; 20194587; 20096402; 19900730; 19844789; 19805547 |
| NT03RE1071 | 6 | 21205867; 20952386; 20923236; 20817769; 20817765; 20802044; 20729364; 20729353; 9446751; 20639538 |
| NT03RE1193 | 12 | 21392579; 21386432; 21383062; 21381651; 21380727; 21377724; 21376765; 21374562; 21371749; 21369731 |
| NT03RE1228 | 12 | 21385156; 21361873; 21270289; 21211725; 21078541; 20957402; 20937828; 20921528; 20921277; 20919990 |
| NT03RE1285 | 8 | 21152904; 20127051; 19517068; 18213407; 19306573; 18284611; 18222470; 16823984; 16794601 |
| NT03RE1309 | 8 | 21317335; 21216959; 21171573; 21039937; 20601653; 20302299; 20218714; 20162368; 20147623; 19932076 |
| NT03RE1652 | 4 | 21220369; 21143879; 21081473; 21044991; 21041549; 21037224; 20972737; 20972438; 20937634; 20932315 |
| NT03RE1653 | 18 | 21321143; 21134354; 21130927; 20952578; 20944611; 20940426; 20919635; 20888471; 20887723; 20836765 |
| NT03RE1691 | 8 | 20652669; 15865160; 9353918; 2199796; 16339959; 10692370; 11322788; 9882667; 10217753; 10460883 |
| NT03RE1964 | 13 | 20943400; 20717102; 16421450; 18279892; 16890342; 13679580; 10993083; 16385054; 18241796; 17964262 |
| NT03RE22027 | 8 | 21092725; 21075259; 20840762; 20662781; 20652542; 20642449; 20634336; 20629583; 20595377; 20570689 |
| NT03RE2212 | 12 | 21372759; 21286385; 20920355; 20735720; 20457869; 20400202; 19694617; 19143597; 18850059; 10617467 |
| NT03RE2369 | 18 | 21143198; 20952578; 20688911; 20435726; 20385096; 20053709; 9758795; 17639608; 10839820; 19054109 |
| NT03RE2444 | 8 | 20007972; 10593889; 11035020; 9331415; 9311786; 9199411; 8729796; 7588711; 7736589; 8382908 |
| NT03RE2764 | 13 | 19225060; 16052580; 7945480; 8317708; 6356984 |
| NT03RE2876 | 6 | 16430214; 15878881; 10419486; 12399474; 10873676 |
| NT03RE3000 | 18 | 19929855; 19270402; 17156194; 17113265; 11241162; 10614776; 10602043; 10458921; 10399315; 6266278 |
| NT03RE3049 | 5 | 21282323; 21212863; 21192794; 21139196; 21043115; 20876533; 20735257; 20730478; 20534592; 3124824 |
| NT03RE3091 | 18 | 2235490; 6768753; 17238922; 10485884; 10203757; 7654405; 7746157; 8391112; 3079747; 3294107 |
| NT03RE3495 | 2 | 20545743; 9658014; 17033719; 1482180; 12799002; 3085688; 10517592 |
| NT03RE3752 | 8 | 16228571; 16041151; 11695918; 11695917; 10648101; 9325424; 8856071; 7781774; 1657867; 1620156 |
| NT03REA0216 | 9 | 20923173; 20888243; 20854817; 20734961; 20730526; 20722739; 21080545; 20708005; 20645653; 20585832 |
| NT03REA0256 | 4 | 21351814; 21307277; 21289531; 21272845; 21237476; 21215828; 21187178; 21043546; 20955520; 20878241 |
| NT03REA0379 | 4 | 21147069; 20481475; 20056514; 16305206; 19683782; 19577534; 19343712; 15572765; 19199786; 18810541 |
| NT03REA03817 | 8 | 377280; 10749541; 779650; 1527502; 6321315; 7136004; 6895386 |
| NT03REA0496 | 5 | 21359825; 21299873; 21265945; 21212458; 21167507; 21080229; 21058444; 21042305; 20890085; 20650733 |
| NT03REA0511 | 13 | 20943400; 20876530; 20457752; 20306515; 16385054; 10993083; 10727213; 16385054; 17929881; 16164993 |
| NT03REA0620 | 9 | 21106941; 20554032; 20450180; 20143849; 20023637; 20057418; 19042983; 18936084; 18787057; 18589008 |
| NT03REA0701 | 8 | 20924576; 20818520; 827241; 19798672; 16857674; 17922758; 17567742; 11495997; 17185548; 17031048 |
| NT03REA07537 | 8 | 21295975; 21087382; 20879547; 20652669; 20495045; 20185233; 20182771; 19775720; 18329116; 1987160 |
| NT03REA0906 | 2 | 20806251; 16461653; 16967903; 16570505; 12909360; 15782938; 11735436; 3129571; 10933831; 10410459 |
| NT03REA1053 | 15 | 21196775; 21186955; 21176025; 21126059; 20976279; 20837642; 20813522; 20728507; 3047011; 20682782 |
| NT03REA1094 | 8 | 21141807; 21135104; 20509166; 20050916; 19659724; 19534725; 19266156; 19224199; 18420022; 18298941 |
| NT03REA1231 | 9 | 21169482; 18060402; 14570270; 12379132; 1989978; 7906398; 7813482; 8001771; 8051146; 8454629 |
| NT03REA1243 | 8 | 1616016; 19168025; 18775409; 18485888; 146039; 18175209; 15166242; 12881515; 9582354; 12560333 |
| NT03REA1269 | 18 | 21041677; 20194040; 19097166; 18367238; 18180927; 18001913; 17957383; 17874325; 16789934 |
| NT03REA1508 | 8 | 19734178; 10985736; 10896219; 10216161 |
| NT03REA1663 | 8 | 20823121; 20006620; 16962351; 16095622; 14534317; 6238732; 10077818; 9708802; 2678166; 9929386 |
| NT03REA1759 | 8 | 21329681; 21265764; 19932956; 19900530; 5432063; 19082766; 18850096; 17640279; 17485854; 17433574 |
| NT03REA1796 | 8 | 21091201; 20514241; 19011745; 12003933; 18422649; 17350704; 15680231; 14965227; 12974644; 12388585 |
| NT03REA18130 | 4 | 21296058; 21053011; 19850038; 19766587; 19647889; 19228319; 18429940; 17975734; 17955192; 17924116 |
| NT03REA1852 | 18 | 21343423; 21329681; 21300042; 21238436; 21073854; 20931090; 20809353; 20736169; 20595938; 20525870 |
| NT03REA2145 | 15 | 20942908; 20302877; 19052358; 18662309; 18293926; 16428390; 10360571; 12352954; 2764573; 10209742 |
| NT03REA22897 | 8 | 21039937; 20302299; 20218714; 20162368; 20147623; 19932076; 9287300; 19633968; 19472231; 19362514 |
| NT03REA2349 | 8 | 21265785; 21265782; 21265775; 19171117; 18719950; 10627495; 17803240; 9735294; 9786877; 15345398 |
| NT03REA2511 | 8 | 21296885; 21228234; 21166653; 20889786; 20862513; 20809073; 20632934; 20171064; 19921179; 19395484 |
| NT03REA2528 | 14 | 21118348; 16876704; 16797581; 15886037; 15627697; 15229895; 15020593; 14745534; 12036579; 11852104 |
| NT03REB0311 | 11 | 17087993; 2880835; 15836702; 12787362; 11952908; 2170327; 7781919; 2670941; 2656641; 3172224 |
| NT03SA0089 | 3 | 21352374; 21269314; 21242397; 20966088; 20962209; 20937760; 20886095; 20860785; 20692223; 20689131 |
| NT03SA0152 | 3 | 21278299; 21070266; 20889719; 20888875; 20876824; 20876287; 20817773; 20687808; 20633230; 11386933 |
| NT03SA0179 | 18 | 18368388; 17974510; 16968229; 16439362; 10411743; 9705652; 169265; 6411705; 6769709 |
| NT03SA0278 | 3 | 21388880; 21288904; 21208281; 21149452; 21097580; 21085702; 21073699; 21062783; 20980996; 20980266 |
| NT03SA0290 | 4 | 21369978; 21297888; 21292859; 21288079; 21266751; 21205005; 21147949; 21147057; 21119612; 21085949 |
| NT03SA0320 | 3 | 21317263; 20826162; 20799977; 20574043; 20562286; 20175237; 19888576; 19740656; 19707758; 19646958 |
| NT03SA0377 | 4 | 17718161; 17496964; 15383705; 10206887; 9244288; 9651373; 1756168; 2914876 |
| NT03SA0417 | 4 | 21344448; 21304478; 21253349; 21223796; 21223537; 21203842; 21189321; 21188384; 21178007; 21168710 |
| NT03SA0418 | 4 | 21294620; 21223796; 21168710; 21063965; 20926713; 20889526; 20830777; 20683117; 15966980; 20635511 |
| NT03SA0419 | 4 | 21314908; 21304478; 21294620; 21282650; 21223796; 21216961; 21190121; 21178007; 21168710; 21091636 |
| NT03SA0421 | 4 | 21338382; 21326141; 21304813; 21294620; 21223537; 21216961; 21203470; 21189321; 21178007; 21152867 |
| NT03SA0422 | 4 | 21344448; 21338382; 21309072; 21305248; 21304813; 21214629; 21223796; 21212180; 21198675; 21168710 |
| NT03SA0424 | 4 | 21344448; 21305248; 21165964; 21136248; 21121506; 21120455; 21048104; 20946065; 20920355; 20889526 |
| NT03SA0425 | 4 | 21308521; 21223796; 21223537; 21203470; 21151619; 21098229; 21059895; 21050125; 20945603; 20926713 |
| NT03SA0426 | 4 | 21034265; 21030678; 20805641; 20740056; 20686089; 20649455; 20637229; 20599534; 20528877; 20511192 |
| NT03SA0427 | 4 | 21338382; 21305248; 21304813; 21223796; 21212180; 21190121; 21189321; 21151619; 21098229; 21091636 |
| NT03SA0642 | 15 | 21378035; 21349272; 21292751; 21263373; 21236606; 21173172; 21103340; 20829395; 20727722; 20685086 |
| NT03SA0728 | 8 | 21239541; 21219252; 21051487; 20656779; 20630778; 20627950; 20545877; 20482643; 20199602; 20118408 |
| NT03SA0769 | 18 | 21392549; 21392187; 21391831; 21384276; 21376650; 21373381; 21373260; 21362484; 21359952; 21348856 |
| NT03SA0807 | 15 | 21375718; 20876289; 18310331; 17293407; 17294332; 16075200; 14523131; 12634340; 11489127; 1904524 |
| NT03SA1016 | 12 | 18762485; 16764819; 15935278; 15889931; 15834796; 15489231; 15109630; 12785843; 11750133; 11478959 |
| NT03SA1142 | 4 | 21344448; 21314908; 21304813; 21304583; 21283822; 21282650; 21264238; 21214629; 21223796; 21223537 |
| NT03SA1574 | 15 | 17697253; 17536838; 17198402; 3231114; 9891797 |
| NT03SA1852 | 15 | 20705504; 19665927; 18177376; 6350179; 14500536; 17561945; 17309633; 9829915; 11976295; 9829932 |
| NT03SA1904 | 12 | 19683795; 19175361; 18958346; 16951132; 15820142; 12798689; 12225974; 11681202; 10484618 |
| NT03SA2042 | 3 | 21388880; 21386961; 21229236; 21208281; 21189325; 21187931; 21149452; 21098238; 21098229; 21097580 |
| NT03SA2043 | 12 | 21151985; 11681202; 11567668; 2649490; 1826005; 2303822; 3058694; 3410850; 3294832; 6175640 |
| NT03SA2092 | 15 | 21384259; 21381029; 21373270; 21354106; 21345218; 21340436; 21328406; 21289437; 21287782; 21252908 |
| NT03SA2098 | 15 | 18563740; 17881745; 17158235; 16860758; 16849035; 11282977; 15358826; 12359119; 12218104; 11269729 |
| NT03SA2099 | 15 | 21371936; 21365379; 21356473; 21353401; 21344448; 21331815; 21330663; 21329998; 21325486; 21318166 |
| NT03SA2168 | 15 | 20932844; 20146748; 19850005; 1630316; 19400808; 19101563; 11373615; 1532388; 1630316; 17023259 |
| NT03SA2245 | 15 | 21389123; 21325479; 21304169; 21212282; 21168773; 21133648; 21075348; 21053310; 21050191 |
| NT03SA2418 | 18 | 21103971; 19682536; 19653651; 19633020; 19584562; 18692040; 18355966; 18032408; 17640976; 17632081 |
| NT03SA2433 | 8 | 20054114; 19328460; 16376935; 15215245; 15184043 |
| NT03SA2621 | 15 | 20935102; 20581202; 19406896; 10715008; 9254694; 16339930; 11694511; 14593252; 3038334; 9135111 |
| NT03SA2799 | 4 | 20950664; 20528258; 9880401; 18667559; 18546154; 15659658; 18343473; 7533787; 17660408; 17393025 |
| NT03SA2814 | 1 | 20724478; 20696207; 20633228; 20497496; 20299287; 20103674; 18429699; 19887595; 10360366; 18227641 |
| NT03UM0027 | 13 | 21339834; 21205822; 21151894; 21126361; 21124213; 21104369; 21098678; 21082278; 21063389; 21048991 |
| NT03UM0269 | 8 | 21332190; 20846448; 20650352; 20639112; 20558190; 20508291; 20481500; 7129027; 10663126; 20088501 |
| NT03UM0321 | 8 | 18198179; 11717276; 11080386; 9647749; 9237769; 8626276; 1404382; 2191951 |
| NT03UM0496 | 18 | 20733981; 20593757; 20096801; 18828841; 18515724; 18457450; 16283740; 16184170; 15779899; 12192868 |
| NT03UM0650 | 6 | 21315607; 21263241; 21238786; 21229606; 21187469; 21163286; 21156161; 21154337; 21151116; 21139098 |
| NT03UM1563 | 14 | 21304254; 21111048; 20303634; 20101413; 20035974; 19883222; 11322199; 19428946; 18600520; 9088342 |
| NT03UM1728 | 8 | 11976749; 2213652; 11342216; 11287154; 8381043; 10795681; 10365245; 9753654; 153704; 2205614 |
| NT03UM1813 | 12 | 21390322; 21385626; 21349823; 21347706; 21342097; 21339618; 21339577; 21338570; 21333514; 21326357 |
| NT03UM1932 | 6 | 21382338; 21293490; 21147160; 20943161; 20925482; 20821744; 20697908; 20622236; 7592953; 20601104 |
| NT03UM2081 | 3 | 21390206; 21375592; 21371889; 21352491; 21352489; 21347280; 21321072; 21285351 |
| NT03UM2524 | 4 | 21147064; 20715744; 20685578; 20630755; 20507761; 20456926; 20435137; 20429863; 20373899; 20302591 |
| NT03UM2788 | 15 | 21233390; 20231482; 20178739; 19999580; 10645945; 16079339; 17804240; 12727879; 14654842; 16880380 |
| NT03UM3210 | 12 | 21265753; 20947006; 20719249; 20117082; 19908377; 19843217; 19622546; 19402088; 10646688; 18976628 |
| NT03UM3352 | 11 | 21323982; 21310067; 21262357; 21260991; 21253567; 21233631; 21224843; 21219416; 21210187; 21205896 |
| NT03XF0176 | 12 | 21040511; 20980266; 20815828; 20026068; 18985050; 17283383; 17029770; 15842960; 15805180; 14687573 |
| NT03XF0302 | 18 | 21347217; 21189125; 20485450; 20371809; 20337660; 20193738; 20179014; 20436038 |
| NT03XF0488 | 11 | 20963614; 19326202; 18348984; 15518819; 15110522; 7873585 |
| NT03XF0696 | 6 | 20650282; 20056615; 1853558; 6283090; 16735502; 3000073; 12051907; 7799432; 7799431; 3159906 |
| NT03XF07264 | 8 | 21273488; 21067711; 21051539; 20967294; 20847102; 20823521; 20795726; 20735086; 20709852; 20708927 |
| NT03XF0757 | 8 | 20937244; 20837308; 20505899; 20370797; 20138856; 20032466; 19244509; 19783111; 19774399; 19758981 |
| NT03XF0810 | 12 | 21311752; 21181720; 21167833; 21072190; 21060843; 20978823; 20955814; 20868763; 20862260; 20807423 |
| NT03XF0894 | 2 | 20051244; 19103164; 18071268; 16842733; 15215101; 15196010; 14617622; 12479413; 11825609; 11108713 |
| NT03XF1122 | 18 | 21264094; 21205015; 21173183; 21149619; 20921306; 20883496; 19342493; 17493798; 19574656; 19558960 |
| NT03XF1141 | 6 | 21289071; 21189249; 20932515; 20884053; 20411565; 20178987; 20133944; 20124220; 20026746; 7552996 |
| NT03XF1287 | 6 | 21367887; 20851892; 3459152; 19651043; 20064458; 18344281; 19154744; 18801373; 18512914; 16606612 |
| NT03XF1657 | 15 | 21301207; 21209092; 21131597; 20954236; 20571059; 20449600; 20381366; 20038587; 19946895; 19900589 |
| NT03XF1711 | 12 | 20553980; 19000094; 18591879; 18547641; 18508466; 18286570; 17981718; 9974389; 17161345; 16782851 |
| NT03XF17237 | 8 | 20618418; 20605070; 20554032; 20460875; 19882252; 19683506; 19381393; 9420328; 19000658; 18655822 |
| NT03XF1729 | 3 | 19759006; 19299729; 20429217; 17569850; 1916089; 12165529 |
| NT03XF1825 | 3 | 20351104; 20042300; 19762441; 10992472; 8636107; 17680768; 16950533; 8743704; 10411730; 15491367 |
| NT03XF2024 | 6 | 20624397; 10540288; 17483094; 9108043; 15749022; 16354656; 15916609; 15530361; 15252043; 12694626 |
| NT03XF2228 | 3 | 12488076; 9224878; 1398996; 8988393; 8973346; 8955641; 8917091; 8899718; 271968; 1676385 |
| NT03XF2333 | 1 | 20140469; 19428471; 17168900; 18285355; 18208521; 18184691; 18050920; 16535512; 17981822; 5277076 |
| NT03XF2337 | 18 | 21295069; 20542955; 20346969; 20219531; 20214925; 18952286; 15481139; 18222405; 15953544; 8172571 |
| NT03XF23944 | 8 | 21388656; 21355995; 21298463; 21274656; 21268886; 21232646; 21223324; 21165671; 21161227; 21124065 |
| NT03XF25065 | 2 | 20942799; 20460376; 20097860; 19810706; 15018306; 11983079; 15952888; 1060112; 16603772; 16905347 |
| NT04CK0043 | 13 | 18400176; 20006901; 19101821; 18178970; 10966112; 16046447; 12393952; 12095691; 11741912; 10869465 |
| NT04CK0099 | 6 | 21263027; 21225324; 21189045; 21107010; 21098122; 21075850; 21070964; 20957187; 20885790; 20861269 |
| NT04CK0176 | 13 | 20943400; 19862811; 19478435; 10727213; 17929881; 17881821; 17114946; 14741355; 16753178; 16408313 |
| NT04CK01977 | 8 | 18695941; 16931423; 10079069; 9665726; 8547259; 2194841; 2693745; 7026235 |
| NT04CK0213 | 12 | 21233422; 21229878; 21087457; 21037108; 20936527; 20860530; 20824169; 20807771; 20719294; 20635096 |
| NT04CK0400 | 18 | 21239611; 16670083; 16652371; 15913950; 15461449; 15210124; 10889041 |
| NT04CK0539 | 8 | 18945221; 21136627; 1339433; 16292556; 15345482; 15272169; 12729017; 9171435; 9787093; 9600061 |
| NT04CK0634 | 12 | 17073462; 11929547; 11487581; 11457858; 11309116; 10766774 |
| NT04CK0825 | 6 | 21377995; 21371942; 21356311; 21329709; 21326243; 21321601; 21278727; 21222484; 21190322; 21188417 |
| NT04CK08662 | 4 | 8624513; 9847077; 3170488; 3170488; 6091052 |
| NT04CK1155 | 3 | 20833803; 20795855; 20581208; 20490454; 20458469; 20419423; 20331969; 20007322; 19765088; 18334218 |
| NT04CK1417 | 18 | 21356312; 21344312; 21305608; 21303518; 21170890; 21220218; 21164026; 21126173; 21063094; 21040791 |
| NT04CK1600 | 6 | 17921483; 15939019; 6049437; 12401175; 11529427; 11410368; 10545127; 9801313; 9121546 |
| NT04CK1610 | 12 | 21287809; 21254169; 21254094; 21223366; 21220096; 21216092; 21212609; 21211725; 21211719; 21199005 |
| NT04CK1676 | 6 | 21211725; 20978146; 20943161; 20864038; 20720002; 20705653; 20580850; 20562521; 20088966; 15935758 |
| NT04CK1747 | 8 | 16863643; 16299377; 15475358; 10348916; 10231544; 7577938; 2843179; 1707310; 1689724; 3533929 |
| NT04MF0352 | 4 | 21292743; 21242066; 21162553; 21131490; 21108067; 20923659; 20863296; 20959858; 20738376; 20693676 |
| NT04MF0503 | 8 | 21073421; 21038095; 20491941; 1906870; 19959660; 19475377; 19196761; 19074156; 18801047; 17957365 |
| NT04MF0889 | 12 | 16920629; 12475170; 11994302; 11053447; 10393315; 10393315 |
| NT04MF1449 | 12 | 20558234; 20180651; 19912053; 19809093; 19728173; 19596416; 19514861; 19396561; 10596841; 2551894 |
| NT04MF1510 | 18 | 9755155; 1282354; 3017638; 7961727; 8276779; 2839840 |
| NT04MF1605 | 4 | 21282905; 21165647; 20675049; 20528770; 20183590; 20162266; 20153634; 20023915; 19968251; 19733046 |
| NT04MF1616 | 8 | 21394876; 21392810; 21392639; 21392618; 21392520; 21392498; 21391977; 21391885; 21391540; 21389901 |
| NT04MF1654 | 12 | 20696584; 20505074; 17397944; 19504624; 19453269; 17451680; 18671782; 9700155; 16361710; 11581258 |
| NT04MF1675 | 6 | 11976102; 10582902; 11591664; 11493005; 11064191; 10395910; 9274008; 6298184; 1579110; 7844814 |
| NT04MF2012 | 6 | 21084251; 21060865; 21052677; 21042780; 20942908; 20937888; 20737003; 20691626; 20573047; 20558295 |
| NT04MF2212 | 8 | 21281738; 21144838; 21070510; 20958327; 20828554; 20828325; 20724146; 20676633; 20950372; 20507888 |
| NT04MF2232 | 3 | 17113996; 11260463; 10986272; 15727837; 11929520; 10678915; 10398745; 8595593; 3002571; 1195397 |
| NT04MF2463 | 2 | 17117872; 11514159; 11027152; 10880976; 10545188; 9756625; 9748348; 9720033; 9139683; 6768722 |
| NT04NS0195 | 18 | 21298009; 20394832; 19631523; 15757906; 19389142; 19066444; 18991398; 9934539; 17626242; 16880288 |
| NT04NS0209 | 3 | 19648040; 19428712; 3053713; 8808924; 179528; 16041130; 15235028; 12480927; 12200473; 11517666 |
| NT04NS0323 | 8 | 10775262; 18061151; 16526093; 16218961; 16023078; 15317750; 15056672; 15004020; 14727190; 12657050 |
| NT04NS0401 | 6 | 19208629; 19920138; 19618961; 17110578; 15561144; 2548993; 1706458; 8294031; 3005115; 6379604 |
| NT04NS0582 | 6 | 21115857; 21091445; 21078976; 21047769; 21115816; 21034533; 20965939; 20953938; 20923998; 20921378 |
| NT04NS0681 | 4 | 19643764; 12864862; 18060666; 15146494; 12144778; 9099861; 10832645; 10650844; 1560774; 9161424 |
| NT04NS0697 | 4 | 18599076; 16630628; 11327763; 10320579; 10712687; 10564473; 10478459; 2407720; 2181149 |
| NT04NS1081 | 17 | 20851888; 9866731; 10220166; 1512194; 8168923; 8063112; 8096622; 2853609 |
| NT04NS11125 | 2 | 20857974; 20460376; 9927721; 16905347; 10692345; 16364320; 16216272; 11158531; 10069079; 12941942 |
| NT04NS1113 | 8 | 15209384; 17768244; 14518180; 11886751; 167863 |
| NT04NS1114 | 14 | 21345279; 21285127; 21268730; 21197712; 21190906; 21181065; 21175319; 21175038; 21174947; 21159786 |
| NT04NS1180 | 18 | 17237285; 12183637; 12386321; 11827531; 11549317; 10788805; 8833320; 1587356; 10453730; 9636708 |
| NT04NS1260 | 12 | 20960971; 20649906; 20608742; 19897036; 19896963; 19646449; 19427318; 7795518; 19281249; 19175361 |
| NT04NS1390 | 1 | 21204787; 20890097; 20675578; 16301309; 9872311; 17904100; 11983164; 16604062; 16224772; 16193226 |
| NT04NS1495 | 18 | 21339607; 21044584; 20978126; 20827720; 20825352; 20600595; 20599688; 20529663; 20513760; 5527561 |
| NT04NS1682 | 17 | 20678169; 20643860; 20331633; 20015516; 19292036; 19147146; 19143563; 19010635; 18791804; 1471713 |
| NT04NS1716 | 8 | 10625447; 9693733; 9211895; 8125116; 8288589; 8138552; 8389362; 8394316; 2173951; 8435436 |
| NT04NS1889 | 5 | 21381755; 21320350; 20808906; 20703317; 20418432; 19968123; 19843229; 19138232; 19721220; 19481094 |
| NT04NS1920 | 8 | 15522295; 14595395; 11215515; 9689094; 11087941; 10585141; 10217486; 2077690; 1856165; 2156810 |
| NT04NS1976 | 8 | 16995898; 15848194; 14645228; 12196153; 10400577; 1840615; 16346383; 9563845; 16348290; 7984110 |
| NT04PA0219 | 12 | 7929230; 8513496; 8444896; 3511473; 2188978; 6656637 |
| NT04PA0223 | 4 | 21187464; 21115899; 21046154; 20935265; 20876799; 20729912; 20705604; 20608983; 20596658; 16968952 |
| NT04PA0566 | 15 | 21164006; 20939886; 20724447; 18307257; 19550110; 19230670; 19479986; 19549496; 19005923; 19005166 |
| NT04PA0572 | 6 | 21044871; 20929870; 20854105; 20643645; 20634321; 20227372; 20222011; 12154123; 10866686; 19137729 |
| NT04PA0630 | 14 | 21377422; 21278271; 20951028; 20938979; 20929557; 20813141; 20605980; 20498911; 20473969; 20452222 |
| NT04PA0658 | 12 | 21317290; 21224036; 21204251; 21190828; 21143798; 21037533; 21129723; 21098669; 21069393; 20975997 |
| NT04PA0666 | 13 | 21374069; 21300798; 21245168; 21239490; 21223507; 21214942; 21210909; 21194456; 21167155; 21148318 |
| NT04PA10402 | 4 | 20669049 |
| NT04PA1124 | 9 | 21253866; 20932952; 18824113; 20418421; 20406286; 20398792; 20046049; 20043999; 19412626; 19002527 |
| NT04PA1272 | 8 | 21168410; 21075201; 21042417; 20979355; 20978456; 20971673; 20876714; 20837795; 20826797; 20823090 |
| NT04PA1388 | 13 | 21136979; 17891922; 17536671; 9169555; 15989950; 14970378; 10805779; 15736940; 10427081; 12369843 |
| NT04PA1425 | 13 | 21390068; 21324174; 21307163; 21301853; 21298286; 21276445; 21220029; 21215957; 21193600; 21186324 |
| NT04PA1600 | 12 | 17213940; 17147425; 16799156; 16717433; 15525405; 14871656; 14642820; 12913311; 12767813; 9560160 |
| NT04PA1619 | 12 | 21237705; 21030067; 20801214; 20679238; 20618881; 20615878; 20605058; 11158746; 20502620; 20451471 |
| NT04PA2193 | 18 | 21082744; 18037401; 18559527; 10470083; 18300232; 17210706; 17074913; 11850253; 16756973; 16132345 |
| NT04PA2708 | 12 | 21189690; 19844166; 11142374; 10606266; 10899131; 10409759; 10619842; 10606651; 10357856; 1741396 |
| NT04PA2945 | 8 | 15491156; 12427946; 12196025; 10830505; 9748316; 9623801; 9480821; 9049017; 9022686; 8020468 |
| NT04RR0059 | 8 | 21368144; 21333651; 21320464; 21314602; 21181421; 21103453; 21072380; 21028883; 20958999; 20952114 |
| NT04RR0083 | 9 | 21389045; 21388747; 21362034; 21345233; 21330637; 21327327; 21315740; 21269353; 21266672; 21265823 |
| NT04RR0168 | 13 | 10937989; 9294008; 9044258; 8722036; 354966; 1742360; 173425; 2665813; 2653827; 3884043 |
| NT04RR0272 | 18 | 20634426; 20185828; 19088429; 16137722; 15571397; 9822723; 14715671; 12578372; 12384697; 11756489 |
| NT04RR0430 | 8 | 21394871; 21388576; 21386971; 21377371; 21376009; 21366780; 21366779; 21365553; 21364886; 21359624 |
| NT04RR0622 | 6 | 16328543; 16154395; 15878881; 12857738; 11976345; 11839306; 11323725; 10722730; 1309939; 10373642 |
| NT04RR0635 | 3 | 20608745; 19646181; 19209901; 16684111; 16042374; 16042373; 15895997; 15581896; 15497971; 15225046 |
| NT04RR0700 | 18 | 21129200; 21146097; 20665521; 20441961; 20121715; 20109267; 20070029; 19795348; 19786296; 19758192 |
| NT04RR0754 | 8 | 21394810; 21394489; 21392919; 21392497; 21391864; 21391587; 21391494; 21391463; 21391462; 21391437 |
| NT04RR0756 | 3 | 20570733; 18485865; 18242189; 17526848; 16573686; 8830709; 1517343; 1314210 |
| NT04RR0768 | 13 | 21078885; 20935471; 20853840; 20852128; 20805354; 6351059; 20571910; 20558763; 20553774; 20370287 |
| NT04RR0824 | 12 | 20817772; 20696419; 20348254; 20337595; 18371630; 18331590; 10386612; 17982050; 17681528; 17108265 |
| NT04RR0897 | 15 | 16430694; 11101667; 10627039; 13890303; 8969513; 7746146; 8497200; 1846145 |
| NT04RR0911 | 6 | 20822508; 20716951; 20656393; 20586875; 20599730; 20511501; 20488783; 11972788; 20471491; 18535086 |
| NT04RR10707 | 8 | 21296660; 20652411; 20606260; 20460097; 17337350; 17169596; 16765626; 15528667; 15496607; 15388917 |
| NT04RR1106 | 13 | 20037770; 18060665; 15215513; 14729335; 2199796; 1772592; 2029524; 6388639; 6186393 |
| NT04RR1368 | 4 | 21216997; 21216995; 20969647; 19680248; 20629754; 20497333; 20487275; 19843223; 15659156; 19699094 |
| NT04RR1484 | 4 | 21371996; 21048089; 20922738; 20542912; 17057717; 20375020; 20298190; 12431441; 19788545; 18363794 |
| NT04RR1508 | 6 | 21362621; 21333363; 21276096; 21263027; 21262234; 21195035; 21188165; 21156963; 21149691; 21145792 |
| NT04RR1551 | 6 | 20675375; 20223211; 19749191; 17522086; 16430690; 12940977; 12535532; 11859073; 11823461; 11809766 |
| NT04RR1566 | 6 | 20972214; 20337945; 20329707; 20222445; 20188667; 19749191; 3300806; 18065386; 17210572; 12857762 |
| NT04SE0055 | 3 | 8636107; 17229144; 17174325; 16573694; 15916614; 15321675; 11796572; 11106397; 12525152; 10792723 |
| NT04SE0084 | 8 | 12087099; 6370952; 9490067; 9030266; 8917463; 7499307; 7663384; 8241179; 8100227; 3426633 |
| NT04SE0352 | 18 | 21390329; 21388519; 21385601; 21385112; 21364900; 21364885; 21347611; 21336594; 21331373; 21330953 |
| NT04SE0516 | 15 | 20149718; 19953306; 12614149; 16394247; 15556616; 15024719; 12167640; 11734884; 11123673; 11063581 |
| NT04SE0669 | 8 | 21306142; 21221720; 21071046; 20976072; 20974920; 20966083; 20960122; 20954716; 20878669; 20851663 |
| NT04SE0849 | 18 | 21078981; 21106757; 21033470; 20937798; 20921307; 20863830; 20834157; 20815017; 20808924; 20737137 |
| NT04SE0925 | 11 | 21129200; 21041684; 20937834; 20851973; 20843802; 20826161; 20665904; 20531477; 20506290; 20351260 |
| NT04SE0951 | 11 | 15733918; 11866517; 10993723; 10336415; 9644594; 8794874; 8577244; 7799431; 8175794; 8175793 |
| NT04SE1012 | 3 | 20622068; 20056708; 19332829; 18571407; 11371519; 16963083; 15612919; 15090547; 8550483; 2492099 |
| NT04SE1026 | 6 | 21377995; 21071726; 21063071; 19616486; 19540206; 18992265; 18477668; 18473720; 8706136; 11106395 |
| NT04SE1032 | 18 | 21357477; 21339577; 21151884; 20876362; 20841472; 20808441; 20513760; 19966050 |
| NT04SE1053 | 15 | 20437037; 19596242; 19029909; 18846279; 18419278; 16079339; 17994337; 17990896; 12763020; 17804240 |
| NT04SE1107 | 9 | 21272313; 2104918; 19014901; 18555004; 18503783; 17851075; 11123329; 10737174; 8621604; 8641446 |
| NT04SE1229 | 12 | 21189343; 21095572; 21075926; 20417202; 20002189; 19696105; 19655811; 19539664; 19438721; 19362478 |
| NT04SE1362 | 1 | 21047785; 20379751; 20039042; 19955263; 19298998; 19011861; 11368918; 18050920; 17853367; 17611817 |
| NT04SE1418 | 4 | 21386816; 21367745; 21247260; 20847002; 20729359; 20497333; 20417640; 20392520; 14657404; 19828268 |
| NT04SE1469 | 4 | 19420995; 19160019; 10629181; 18456580; 18424550; 17611636; 16669774; 15072439; 14766322; 14723956 |
| NT04SE15647 | 8 | 20390382; 15784996; 14506920; 12843670; 11934574; 9520281; 11607209; 9339561; 9188535; 8534977 |
| NT04SE1643 | 15 | 21342462; 21229241; 21219458; 21193608; 21169488; 20926415; 20716687; 20708625; 20656783; 20639450 |
| NT04SE1699 | 6 | 21394103; 21393642; 21386659; 21386588; 21386030; 21383921; 21383542; 21383015; 21381664; 21376055 |
| NT04SE17831 | 8 | 11048786; 9463796; 98070; 8913721; 7551966; 8313623; 2105929; 1285890; 1647290; 2714795 |
| NT04SE1793 | 8 | 21378254; 21378103; 21368144; 21365448; 21356170; 21342604; 21326867; 21324494; 21320465; 21309218 |
| NT04SE1846 | 8 | 19577535; 19480945; 14564521; 10827276; 10706662; 9919654; 3067084; 7588754; 6331453 |
| NT04SE1849 | 8 | 18500479; 14564521; 10827276; 9919654; 3067084; 7588754; 6331453 |
| NT04SE1874 | 12 | 20159555; 19455308; 19339102; 18256511; 2670554; 17182002; 16988445; 16973605; 16233770; 10439044 |
| NT04SE2115 | 15 | 20444088; 20432948; 18673380; 12662922; 14617141; 12562816; 12756178; 11166993; 786255; 9053320 |
| NT04SE21205 | 8 | 20807881; 10859185; 19812182; 18557832; 15270677; 8676881; 9314531; 16653082 |
| NT04SE2212 | 6 | 21321206; 21288763; 21149272; 21113127; 21108798; 21097894; 21091504; 21085634; 21059643; 20979348 |
| NT04SE24675 | 8 | 19152018; 16049785; 14621292; 1577010; 1743523 |
| NT04SE2648 | 5 | 21223465; 9385560; 16301812; 16233726; 16233126; 12951510; 11488932; 11006082 |
| NT04SE2882 | 18 | 21394349; 21394088; 21393446; 21391982; 21390223; 21387375; 21383163; 21383000; 21371473; 21370032 |
| NT04SE2896 | 8 | 21366819; 21170562; 20553812; 19885993; 20379702; 20159152; 19888992; 19784828; 17485086; 10559182 |
| NT05AM0440 | 8 | 21195229; 8416294; 10715138; 9813031; 9572839; 9308890; 9166866; 8591049; 3374614; 891918 |
| NT05AM0471 | 18 | 21352605; 21251871; 21163869; 21156049; 21103969; 21091508; 21081109; 21057015; 20977453; 20960970 |
| NT05AM0507 | 4 | 21316437; 21280586; 21253945; 21216094; 21172440; 21155849; 21145545; 21075418; 20926450; 20813407 |
| NT05AM0627 | 12 | 21303942; 21261463; 20871837; 20713620; 20679205; 20667621; 19763421; 19729222; 19696109; 19553530 |
| NT05AM0746 | 18 | 21289069; 21209188; 21118530; 21112832; 21055621; 21048711; 21035764; 21034559; 21029321; 20974636 |
| NT05AM0822 | 9 | 17385315; 4623694; 15241633; 10937442; 9559566; 9231425; 12231903 |
| NT05AM1089 | 12 | 20712325; 19839645; 17453712; 17167531; 16725155; 16478121; 16443604; 16289685; 15946951; 15882050 |
| NT05AM1270 | 18 | 20888887; 20597903; 20594231; 20511703; 20219826; 19799071; 19590924; 19535423; 19116231; 19083439 |
| NT05AM15535 | 8 | 18547168; 18224279; 10708746; 16752899; 3031427 |
| NT05AM1555 | 18 | 21167126; 20798507; 20179498; 19821034; 19759922; 19753330; 19292831; 19165895; 18771934; 18668356 |
| NT05AM2093 | 12 | 21394741; 21393041; 21392067; 21390545; 21386874; 21378199; 21375177; 21371430; 21358699; 21368222 |
| NT05AM2539 | 6 | 21364005; 21203457; 21148751; 20978160; 20702603; 20691712; 4870278; 20669221; 15020458; 20336301 |
| NT05AM2577 | 8 | 20675490; 20083491; 19118348; 17244482; 16316988; 16229462; 15772944; 15210329; 12603323; 942051 |
| NT05AM2809 | 3 | 20473714; 17704015; 11038152; 11800269; 11470430; 11294642; 9193713; 9184839; 7634071; 8289782 |
| NT05AM2968 | 13 | 21301021; 21050474; 20837149; 20836875; 20739293; 20728359; 20639212; 21080555; 20471262; 20423905 |
| NT05AM3240 | 6 | 19211838; 9215887; 19470480; 17965729; 17936710; 12861005; 9811458; 12417133; 17802953; 8287473 |
| NT05AM3481 | 8 | 21393833; 21391225; 21388960; 21387410; 21386928; 21386095; 21384112; 21383242; 21383078; 21383058 |
| NT05AM3513 | 4 | 8864117; 18624796; 11679669; 8264799; 12488439; 11890537; 11751054; 10691089; 8878044; 8866480 |
| NT05AM35171 | 8 | 21224012; 21181285; 21148295; 21088901; 20934512; 20803549; 20607622; 20568228; 20520628; 3030444 |
| NT05AM3526 | 15 | 20851105; 20658980; 20142036; 1355089; 19558970; 16700552; 19271268; 9176208; 17477533; 13129936 |
| NT05AM3709 | 11 | 21144870; 20820867; 20498502; 15985826; 18547513; 11472933; 16772557; 16142226; 16023243; 15165860 |
| NT05AM3725 | 15 | 21365885; 21364741; 21331764; 21330827; 21319047; 21319022; 21311398; 21306759; 21298915; 21265880 |
| NT05AM4426 | 8 | 21394930; 21394876; 21394871; 21394845; 21394802; 21394381; 21394293; 21393488; 21393351; 21393241 |
| NT05AM4461 | 18 | 21103969; 11048718; 20188576; 20103563; 20003133; 17493798; 19373193; 18208836; 19252335; 19816142 |
| NT05AM4881 | 3 | 21209026; 20722279; 20663087; 19697141; 19645738; 18037139; 11842152; 1557130; 16452068; 15012210 |
| NT05AM4909 | 11 | 21349737; 21333546; 21238459; 21118674; 21098677; 21046147; 20978221; 20964339; 20960131; 20960127 |
| NT05AM4969 | 18 | 21134393; 20952391; 20851900; 20731460; 20712617; 20706981; 20655873; 20563633; 20553499; 9508806 |
| NT05AM5379 | 6 | 20930525; 20879535; 20729363; 20435476; 20304912; 19950673; 19888556; 19795669; 15564128; 19622418 |
| NT05AM5973 | 8 | 21128244; 21095151; 21072851; 20637282; 19932039; 19739253; 19685155; 19457086; 19294497; 19235826 |
| NT05AM6256 | 11 | 19018586; 15165887; 16581939; 15211355; 15987878; 12612829; 10077536; 1316613; 1310791 |
| NT05AMA0217 | 4 | 15628860; 12029522; 2614374; 4029816; 7391816 |
| NT05AMB0127 | 18 | 21355292; 20974273; 20828517; 19926314; 19497872; 8722628; 18972271; 18635743; 18488195; 18334180 |
| NT05AMC0004 | 3 | 9620966; 18566135; 17172020; 16769709; 14982627; 2822655; 12809607; 12596863; 11389900; 7565110 |
| NT05AMD0159 | 8 | 21278273; 20937819; 20732298; 19479006; 19636076; 19481532; 19446033; 1616016; 19390955; 15972314 |
| NT05LS0570 | 15 | 21320182; 21310260; 21293909; 21289293; 21245055; 21229249; 21225279; 21209319; 21190950; 21161211 |
| NT05LS0594 | 3 | 17110961; 10515909; 15251431; 7559346; 9133736 |
| NT05LS0862 | 6 | 21388532; 21378185; 21365542; 21350762; 21343909; 21336027; 21325134; 21321231; 21216906; 21145896 |
| NT05LS0901 | 12 | 17630120; 16625836; 12651108; 12630905; 11687837; 11118589; 10066485; 10397851; 9862843; 9268732 |
| NT05LS1003 | 1 | 21161018; 21084402; 20852179; 20847306; 20739017; 20706197; 1730614; 20667967; 20590842; 20487243 |
| NT05LS1135 | 11 | 20538004; 6328211; 10880457; 10666445; 9790683; 11152618; 11030337; 10911996; 10704304; 7024729 |
| NT05LS1194 | 9 | 12621436; 18429111; 8626468; 17652793; 17628524; 17412959; 17488650; 9334202; 11544350; 17213187 |
| NT05LS1205 | 4 | 21354109; 21297965; 21274617; 21266410; 21235734; 21224082; 21211541; 21178363; 21166830; 21159856 |
| NT05LS1206 | 3 | 20383019; 20118250; 16357223; 11069242; 19389766; 10992472; 8636107; 17298895; 17229144 |
| NT05LS1217 | 12 | 18761696; 18761694; 17581128; 10206711; 9004506; 12215599; 11164302; 105241; 10206711; 8843676 |
| NT05LS1327 | 8 | 2123834; 19628714; 11396927; 9397158; 9252455; 8665954; 8612675; 1329869; 7685596; 2222460 |
| NT05LS1485 | 8 | 20730594; 20206595; 4287829; 18294138; 17701551; 17164295; 16232504; 15907384; 15644203; 15100050 |
| NT05LS1589 | 8 | 21905665; 21874381; 21858493; 21833603; 10189289; 21732915; 21699927; 21609791; 21604744; 21515690 |
| NT05LS1697 | 12 | 21262231; 19913481; 19241474; 17616559; 17074076; 17241197; 16923819; 16805830; 15866516; 15772085 |
| NT05LS1958 | 15 | 20401585; 17223624; 18156760; 10471558; 10985787; 10754256; 10463151; 9371343; 2824439; 11902729 |
| NT05LS2046 | 11 | 21129201; 20525695; 19561086; 18687036; 19109888; 19109896; 18627466; 16377618; 18234214; 16807240 |
| NT05LS2971 | 9 | 21384891; 21382465; 21354350; 21296885; 21263204; 21187145; 21155016; 21110176; 21106133; 20971100 |
| NT05LS3007 | 8 | 19734178; 19584547; 19577535; 18582433; 17176101; 3148839; 12578386; 12530544; 12446663; 12136145 |
| NT05LS3146 | 6 | 21169503; 21183688; 17970226; 17951987; 17691945; 17116675; 17483803; 15572765; 16734777; 16704790 |
| NT05LS3225 | 12 | 21388432; 21382117; 21375692; 21365649; 21352096; 21350674; 21339476; 21339473; 21323474; 21315759 |
| NT05LS3604 | 12 | 21281692; 21058401; 20709850; 18397761; 17626354; 17458548; 17437962; 16717433; 16185928; 16061231 |
| NT05LS3795 | 3 | 21386961; 21378199; 21370307; 21358756; 21337515; 21327035; 21301102; 21290549; 21274430; 21243929 |
| NT05LS3876 | 4 | 15057485; 10762253; 11065271; 10473570; 10439395; 9274028; 8655549; 8628234; 7966271; 1469717 |
| NT05LS3926 | 11 | 10465774; 17176048; 16008350; 15755448; 15733918; 11866517; 11715858; 1830344; 11279084; 11062051 |
| NT05LS4043 | 4 | 21292750; 21037009; 20952574; 20562102; 20487300; 20059695; 1878971; 18467857; 18092466; 18072238 |
| NT05LS4184 | 4 | 21125389; 21088297; 21035738; 20805503; 20714314; 20594290; 20479271; 20413556; 8092857; 17461888 |
| NT05LS4281 | 8 | 20833277; 20622334; 20096367; 20054117; 16936696; 19650766; 15953771; 18444899; 19084557; 18088303 |
| NT05LS4760 | 15 | 21326934; 21284980; 21272569; 21106082; 21062958; 21038480; 20961603; 20886052; 20861189 |
| NT05TP0053 | 11 | 20944064; 8287692; 10547692; 19845642; 19473321; 15983781; 18815788; 10833198; 18539327; 3692483 |
| NT05TP0061 | 11 | 19396961; 11152613; 16269739; 11157923; 17259614; 16685391; 2987991; 16272378; 6644240; 12855658 |
| NT05TP0358 | 5 | 21082203; 20639325; 20381578; 18698327; 17854402; 18827348; 18495664; 18280134; 10906431; 17686999 |
| NT05TP0498 | 6 | 21390305; 21241707; 21185379; 21075927; 21035394; 20974949; 20949106; 20880997; 20875907; 20871112 |
| NT05TP0547 | 8 | 18323661; 14646139; 12627946; 9799514; 10514453; 7726577; 8001576; 7966281; 2174880; 2606914 |
| NT05TP06674 | 8 | 21382994; 21353612; 21349401; 21346032; 21333496; 21332624; 21331589; 21316941; 21305660; 21298206 |
| NT05TP0709 | 15 | 21257771; 20822510; 20572941; 20457789; 20439471; 20221736; 3525846; 19835966; 19833778; 12614149 |
| NT05TP07304 | 8 | 20393999; 20385261; 19962134; 19962130; 19834705; 19707756; 10825529; 19239186; 18756100; 18553919 |
| NT05TP1325 | 17 | 15808936; 11115109; 9701826; 9661673; 8866481; 8277242; 1350315; 2507166 |
| NT05TP1352 | 4 | 19095018; 18753783; 16897036; 16092523; 11309112; 10640597; 9426140; 8733225 |
| NT05TP1468 | 12 | 20971078; 20940304; 19916060; 10922362; 16183253; 11923872; 18664458; 15312650; 12198498; 11118137 |
| NT05TP1895 | 15 | 21378035; 21193605; 20675476; 20437273; 9534977; 19581470; 19856270; 19825410; 15808931; 17468759 |
| NT05TP1941 | 6 | 19332813; 19070898; 18663525; 15247157; 14656901; 12598537; 12507474; 11686531; 8635477; 10473086 |
| NT05TP2324 | 8 | 21220330; 20650410; 19144645; 19793082; 19580810; 19447057; 18692052; 18266866; 18260136; 18231862 |
| NT05TP2470 | 8 | 19833097; 16788055; 14981290; 9177272; 7706221; 8157629 |
| NT05TP2476 | 18 | 16113235; 14600261; 12552563; 12171517; 11850812; 11495928; 11375395 |
| NT06BA0053 | 8 | 21116622; 21081062; 21071492; 20971847; 20952576; 20937896; 20802042; 20798065; 20705660; 20697695 |
| NT06BA0060 | 8 | 14672950; 9217023; 9169436; 8663126; 8845354; 7703256; 8394111; 271968; 1332881; 1322173 |
| NT06BA0061 | 8 | 19268423; 17425940; 8800209; 8276245; 109312; 2162835 |
| NT06BA0147 | 4 | 21378185; 21321231; 21139222; 21126315; 21045543; 20956557; 20944626; 20935500; 20844015; 20705241 |
| NT06BA0234 | 5 | 20042022; 19903478; 19770499; 19664586; 11553747; 19067028; 1814508; 17540769; 17095009; 16579463 |
| NT06BA0421 | 8 | 21071046; 21068394; 21048403; 20976072; 20974920; 20954716; 20888556; 20878669; 20861582; 20851663 |
| NT06BA0443 | 2 | 20861021; 20690630; 19122276; 18006324; 7815950; 8010978; 2628174 |
| NT06BA05247 | 8 | 12093478; 11230796; 9919993; 9463820; 9463813; 7480016; 8378380; 1619617; 2757387; 2709384 |
| NT06BA0676 | 12 | 20926390; 20883728; 19916930; 19819899; 19665591; 10829079; 17530480; 18029266; 18074461; 10569938 |
| NT06BA0723 | 12 | 20979473; 21266412; 21261072; 21228166; 20818887; 21207565; 21203342; 21198752; 21191724; 21182092 |
| NT06BA0763 | 17 | 21289066; 21280007; 21268339; 21263028; 21248844; 21233849; 21211725; 21205867; 21187417; 21183718 |
| NT06BA0871 | 5 | 20736171; 20673205; 7836277; 19957302; 19580157; 18700747; 18976214; 18976214; 18706514; 18700747 |
| NT06BA0972 | 8 | 21389104; 21385868; 21377462; 21371429; 21323311; 21300479; 21299470; 21282103; 21275844; 21252495 |
| NT06BA1143 | 3 | 20870880; 20854854; 20843801; 20816175; 20816174; 20801880; 20688818; 20679665; 20602334; 20586114 |
| NT06BA1185 | 8 | 21068394; 20932062; 20513347; 19821612; 18723024; 19308466; 8385603; 6355817; 18444899; 12198487 |
| NT06BA1266 | 13 | 19627989; 10810734; 17083917; 15822125; 12731872; 12697167; 12533518; 10810734; 10739928; 9766225 |
| NT06BA1286 | 12 | 21368759; 20957402; 20637416; 20462489; 20416323; 20167799; 20068042; 20038591; 20022957; 20014030 |
| NT06BA13070 | 8 | 20835892; 20720174; 20685142; 20591708; 20487290; 20462777; 20425116; 20372858; 20106967; 20029514 |
| NT06BA1332 | 9 | 15539300; 10960477; 8388033; 3013315; 6067194; 6824716; 7236695 |
| NT06BA1356 | 3 | 21074048; 17588176; 17244817; 16476725; 16162506; 10379365; 9336672; 15130128; 14617152; 8494882 |
| NT06BA1527 | 9 | 3580384; 3550347; 6434856; 7126602; 5497665; 1058495 |
| NT06BA1587 | 18 | 20643857; 19132541; 12136096; 18160489; 10331874; 10201093; 15364914; 14514697; 12960164; 9355728 |
| NT06BA1607 | 2 | 17822383; 17217963; 15094056; 12269807; 11286891; 11200221; 10944349; 10225425; 8971718; 13778735 |
| NT06BA1710 | 18 | 21115718; 20592031; 11163470; 19556290; 18584243; 17891922; 16942819; 9234451; 15115184; 14585681 |
| NT06BA1766 | 12 | 19784642; 19426854; 15142336; 12475202; 11227212; 11067927; 10421483; 10395480; 10075426; 9079288 |
| NT06BA1775 | 9 | 10592522; 12005535; 10713420; 10709984; 9919653; 9738901; 3036654 |
| NT06BA1782 | 18 | 21034611; 20461691; 12624135; 16641205; 19220077; 18804975; 18632686; 10699106; 18306177; 17660803 |
| NT06BA2311 | 18 | 21333794; 21327044; 21314477; 20923769; 20876094; 20862217; 20828376; 20823215; 20815824; 20688817 |
| NT06BAA0197 | 18 | 21129233; 21034414; 19961827; 19332836; 19434674; 12770876; 18082614; 17976368; 9586241; 17687333 |
| NT06BAA0265 | 9 | 21105730; 21044894; 20966115; 20958018; 20931200; 20801890; 20716241; 20708801; 20678669; 20676708 |
| NT06BAA0324 | 1 | 12912839; 17944491; 11403301; 12755589; 18493744; 18201830; 19248588; 17911033; 17583882; 15486256 |
| NT06BAA04141 | 8 | 21282208; 20213667; 20208151; 20064433; 19244759; 19198899; 18668260; 10089455; 17081012; 16323658 |
| NT06BAA0485 | 9 | 16430210; 14728675; 11524729; 10869041; 15146484; 14752098; 12962497; 11872165; 11812788; 11700068 |
| NT06BAA0573 | 18 | 7651325; 2254301; 2549374; 3568126; 6376472 |
| NT06BAA06577 | 8 | 21237173; 17126562; 15489436; 16272392; 11904922; 9582432; 388356; 2020552; 7765840; 8181753 |
| NT06BAA0691 | 15 | 20522491; 19919539; 19901023; 19695263; 7984417; 19220743; 18573895; 20076707; 11207743; 17379708 |
| NT06BAA0802 | 8 | 12633849; 10622429; 9335565; 8241181; 8399240; 1309808 |
| NT06BAA0912 | 8 | 20979470; 21271486; 21131924; 21107304; 21104890; 21099121; 20847308; 21060077; 20595428; 21031626 |
| NT06BAA0992 | 12 | 21265178; 21259244; 21254625; 21248787; 21248114; 21205794; 21205672; 21205675; 21187481; 21186515 |
| NT06BAA1016 | 8 | 20127234; 11272814; 10849789; 10449046; 7590328; 7764620; 1527498; 14775715; 1796691; 3553176 |
| NT06HS0091 | 12 | 20570967; 16129398; 20188867; 19856132; 17108080; 19100870; 2426003; 16125072; 18006445; 17897943 |
| NT06HS0129 | 6 | 21394759; 21394438; 21394098; 21393997; 21393996; 21393543; 21393511; 21393330; 21393125; 21392897 |
| NT06HS0163 | 6 | 19364474; 10882079; 17107651; 15141361; 10373010; 10198119; 9609728; 6381967; 7536191; 6409898 |
| NT06HS0276 | 3 | 21383969; 21367879; 21326923; 21325338; 21317953; 21301100; 21288903; 21253797; 21243086; 21241053 |
| NT06HS0300 | 6 | 21383100; 21372421; 21351566; 21347491; 21332098; 21325737; 21319705; 21318561; 21317456; 21315610 |
| NT06HS0345 | 8 | 12601138; 11846799; 9419225; 9348086; 6894889 |
| NT06HS0433 | 6 | 21081488; 20834227; 20811461; 20811460; 20729809; 9224597; 10471747; 2659437; 18843352; 11124263 |
| NT06HS0438 | 6 | 21098118; 21088405; 21042967; 20960278; 20863555; 20860785; 20816743; 20732430; 20692642; 20677014 |
| NT06HS0491 | 8 | 21274865; 21190281; 20737532; 20660157; 20606288; 12829696; 20130997; 20091229; 20026009; 20025846 |
| NT06HS0522 | 12 | 21364044; 21259244; 21251197; 21244406; 21226391; 21207786; 21187494; 21123640; 20335564; 21106770 |
| NT06HS0527 | 8 | 17333169; 17227414; 15101982; 12079877; 238987; 3607038; 3830175; 3830163 |
| NT06HS0568 | 9 | 21134903; 21258118; 20956318; 20825197; 20601511; 20541526; 20523008; 20463027; 20460577; 20406286 |
| NT06HS0588 | 6 | 21271694; 20828134; 20573560; 20444223; 20222748; 19878455; 19074258; 18449197; 17631397; 17355959 |
| NT06HS0630 | 17 | 18004386; 19143595; 18691966; 10784442; 10212987; 16272138; 14697197; 11331764; 15743411; 15385556 |
| NT06HS0733 | 6 | 15939019; 6049437; 12401175; 11410368; 9801313; 9121546 |
| NT06HS0788 | 12 | 21394379; 21394106; 21391660; 21391436; 21390501; 21389833; 21385343; 21381897; 21376322; 21376067 |
| NT06HS0880 | 3 | 21284263; 21262610; 21261075; 21209279; 21205672; 21191069; 21183069; 21183069; 21183032; 21182591 |
| NT06HS0887 | 12 | 20976295; 20718419; 20601508; 1270419; 20458469; 20148900; 20129918; 20007322; 19900465; 19765088 |
| NT06HS0983 | 15 | 20511587; 19368556; 17307816; 12102556; 10660627; 12850130; 11073907; 16221580; 15611104; 15159566 |
| NT06HS1178 | 3 | 21076487; 21039921; 20705665; 20660674; 20530728; 10093527; 20128354; 20050159; 1328995; 15922359 |
| NT06HS1205 | 2 | 20801032; 20730526; 19721754; 17368731; 17208200; 16786318; 16698791; 16554305; 16285735; 16006204 |
| NT06HS1223 | 3 | 21372849; 21356201; 21341753; 21337515; 21327035; 21310062; 21301102; 21255117; 21247889; 21241420 |
| NT06HS13815 | 2 | 21036900; 20964339; 20960122; 20822503; 20807998; 20801922; 20711222; 20673043; 20664956; 20660125 |
| NT06HS1451 | 8 | 20888212; 20803053; 20374354; 20068286; 20043918; 19935813; 19820286; 1833582; 19169172; 18969149 |
| NT06HS1544 | 4 | 21134393; 21062372; 20616615; 19788237; 19339379; 19195736; 15885362; 19027009; 19008781; 18813846 |
| NT06HS1636 | 5 | 18947394; 17110332; 18702616; 728097; 11980912; 10348866; 12623016; 11980912 |
| NT06HS1678 | 17 | 21350489; 21326933; 21318903; 21289066; 21280007; 21268339; 21263028; 21248844; 21233849; 21211725 |
| NT06HS1700 | 6 | 19671008; 12008917; 10331238; 9829825; 9242608; 2426263; 8929390; 1915295; 7763375 |
| NT06HS1739 | 13 | 21306992; 21131277; 20836892; 20398217; 20353943; 20004207; 16245325; 19746363; 12765840; 19627989 |
| NT06HS1745 | 18 | 20696265; 20577771; 20529855; 20511227; 12913005; 19834508; 19698777; 18369135; 2139726 |
| NT06HS1748 | 8 | 20372025; 20082212; 19720061; 19663915; 19138746; 19020348; 19003877; 16897437; 16563431; 15272184 |
| NT06HS1749 | 8 | 20571891; 20382158; 20372025; 20082212; 20026332; 19900465; 19760172; 19720061; 19663915; 2744487 |
| NT06HS1767 | 8 | 21369489; 20881006; 20802145; 20460943; 19930040; 19759006; 18768894; 18706831; 18506843; 18306459 |
| NT06HS1790 | 11 | 21365356; 21274582; 21265757; 21220507; 21160097; 21151660; 21117224; 21097442; 21070798; 21070176 |
| NT06HS1923 | 15 | 21235644; 20946847; 15811514; 15170486; 11688972; 9643537; 8481091 |
| NT06HS1996 | 1 | 20805334; 19551475; 17451543; 10024454; 14699121; 10220164; 10196182; 7652206; 6339072; 1327967 |
| NT06HS2061 | 6 | 21289047; 21250545; 21116703; 21087929; 21047797; 21035729; 20943007; 20847228; 20713013; 20709025 |
| NT06HS2192 | 13 | 1281147; 11100899; 8910435; 8037726; 7287676; 7353033 |
| NT06HS2194 | 17 | 21233849; 21211725; 21183650; 21164032; 21131976; 21124318; 21095579; 21073264; 21057108; 21046623 |
| NT06HSC0059 | 18 | 21328631; 21308987; 21308737; 21302286; 21262963; 21248339; 21239536; 21227585; 21215450; 21212261 |
| NT06HSC0259 | 18 | 20966125; 20510449; 20660472; 20435896; 20375064; 16905151; 19724867; 11278375; 9537360; 12493732 |
| NT06MH0140 | 15 | 21242066; 21167274; 20693676; 20543140; 20398208; 13278318; 2002000; 20226790; 19646451; 19540260 |
| NT06MH0223 | 18 | 7983143; 8282764; 1325982; 1650377; 2561127; 16477713; 3913624 |
| NT06MH0317 | 18 | 20704181; 20147287; 16875436; 19761223; 19004000; 18793179; 18252722; 17600077; 17168567; 17148438 |
| NT06MH0475 | 3 | 20602334; 20532300; 20331963; 19912589; 19217283; 19214747; 18563288; 3053713; 18393820; 18387370 |
| NT06MH0480 | 15 | 12686116; 11158353; 1318499; 9844742; 9495022; 9467911; 9168623; 8392137 |
| NT06MH0529 | 12 | 21358633; 21085491; 21029866; 20974972; 20732327; 20674557; 20660722; 20516198; 20505323; 2539565 |
| NT06MH0579 | 12 | 20175878; 19759340; 19186947; 18092178; 7655503; 17786443; 17714804; 15194187; 16874311; 15728581 |
| NT06MH08277 | 8 | 20202935; 10878009; 10671500; 1529353; 8626532 |
| NT06MH1402 | 8 | 20026332; 19760172; 19720061; 2744487; 10900003; 18313393; 17263559; 16897437; 16627940; 15892698 |
| NT06MH1492 | 1 | 19351325; 19334594; 17942358; 18498255; 794063; 10445884; 9665716 |
| NT06MH1535 | 8 | 15987803; 19387075; 10339593; 18651753; 15264254; 17448440; 17046710; 16788776; 15317750; 15317595 |
| NT06MH1637 | 4 | 21162553; 21135094; 20802037; 11743865; 9241419; 20303644; 2002000; 20223212; 20067303; 19778064 |
| NT06MH2082 | 17 | 21383132; 21368912; 21311021; 21273289; 21209330; 21050862; 20956791; 20952379; 20803644; 7523114 |
| NT06MH2312 | 15 | 19300486; 17660417; 10476035; 15225327; 11851334; 16561900; 9425243; 8459762; 2838724 |
| NT06MH2946 | 6 | 21388532; 21365542; 21336027; 21325134; 21321231; 21091440; 20956557; 20708016; 20657659; 20655937 |
| NT06MH3249 | 15 | 21393328; 21385852; 21371195; 21362127; 21349170; 21346256; 21343614; 21322782; 21320366; 21305800 |
| NT06MH3561 | 13 | 20525789; 2265611; 10094308; 4556577; 9047363; 13221549; 7599277; 7689113; 2478711; 2471265 |
| NT06MH3586 | 17 | 20844231; 20733069; 18842848; 19492989; 19114111; 19047739; 18815118; 18786148; 18691966; 10565912 |
| NT06MH3783 | 3 | 21368380; 21361872; 21327254; 21317243; 21304485; 21286806; 21283832; 21273509; 21247092; 21233332 |
| NT06MM03221 | 4 | 20416506; 19656197; 19470640; 9168914; 11217410; 19334594; 16667981; 16187095; 16000708; 15470123 |
| NT06MM0338 | 4 | 21275339; 20962592; 20937570; 20807804; 20619629; 20571842; 20554449; 20553608; 20478922; 20433855 |
| NT06MM0397 | 11 | 21087587; 21073231; 21071804; 20967778; 20937911; 20656872; 20633563; 20630556; 20827805; 20510758 |
| NT06MM0512 | 18 | 21377658; 21360185; 21332878; 21327035; 21315829; 21314964; 21298042; 21294843; 21282101; 21277932 |
| NT06MM0716 | 4 | 21391663; 21385867; 21354257; 21322032; 21319188; 21318024; 21315197; 21288496; 21287625; 21273463 |
| NT06MM0807 | 8 | 21185447; 20878090; 20680488; 20660617; 20371361; 20046096; 20041317; 20036318; 20013193; 19923446 |
| NT06MM0865 | 18 | 21369979; 21340673; 21315360; 21300901; 21297166; 21274516; 21210194; 21167175; 21158418; 21070945 |
| NT06MM1327 | 8 | 9297468; 9020790; 8483449; 14447230; 2543298 |
| NT06MM1471 | 18 | 21054068; 21029107; 11812153; 17712582; 17239395; 16091847; 15741220; 15518568; 15476980; 15134935 |
| NT06MM1626 | 9 | 15551059; 11352749; 3129571; 9738901; 10746747; 9459298; 9177055; 8352645 |
| NT06MM1656 | 8 | 21226084; 21207533; 21051545; 20977213; 20862391; 20812788; 20593098; 20558724; 20522543; 20513347 |
| NT06MM1970 | 15 | 21278122; 20807373; 2296602; 9449833; 20514241; 18407508; 17319847; 20031914; 18088315; 16630556 |
| NT06MM2118 | 15 | 20724387; 19832907; 19780840; 14665678; 19260968; 18765794; 19021569; 17609140; 17561945; 15734656 |
| NT06MM2535 | 12 | 21372286; 21333629; 21327044; 21320469; 21309470; 21297983; 21292975; 21282188; 21281954; 21280223 |
| NT06MM2719 | 3 | 20473714; 19596226; 19056079; 17668234; 17405771; 17329246; 16670126; 15843594; 15639245; 15155763 |
| NT06MM2887 | 5 | 20878347; 20837006; 20816195; 19851340; 19507290; 16049196; 11595639; 18436239; 18214472; 18186488 |
| NT06MM3016 | 18 | 21322090; 21284755; 21246047; 21112065; 21095462; 21048708; 21085165; 20961553; 20886383; 20871425 |
| NT06MM3074 | 8 | 21265770; 21148731; 21141817; 20709842; 20649643; 20104886; 19762442; 19545139; 19458823; 10825330 |
| NT06MM3525 | 11 | 21393246; 21378602; 21373795; 21372558; 21362197; 21350632; 21347309; 21346030; 21325565; 21322032 |
| NT06MM3661 | 8 | 18396129; 18061151; 16870178; 16788776; 15559763; 15317750; 15300772; 15115179; 15056672; 14740892 |
| NT06MM3849 | 3 | 21241472; 21229881; 21205103; 21161258; 21151979; 21030539; 20841351; 20824214; 20685834; 20684602 |
| NT06MM3971 | 12 | 9661666; 2027072; 9781884; 11934614; 12417198; 12240967; 1809829; 10884397; 8693022; 1517343 |
| NT06MM4055 | 4 | 16272391; 16238624; 15491362; 11929518; 11260478; 3309346 |
| NT06MM4262 | 11 | 15353349; 20167799; 20145107; 19478801; 19937615; 12824352; 19180639; 14728676; 10087920; 17669426 |
| NT06MM4329 | 8 | 21276451; 20513347; 20398779; 20075289; 19855063; 19793027; 19188839; 18022381; 18930705; 14592463 |
| NT06MM4331 | 3 | 20394418; 20124190; 19754149; 18984595; 19201821; 17660420; 17621554; 10504382; 8370744; 15654872 |
| NT06MT00734 | 8 | 21393568; 21392970; 21389598; 21388656; 21381086; 21378034; 21377658; 21377361; 21377353; 21376555 |
| NT06MT0104 | 8 | 21187144; 18335216; 17668201; 11751810; 16713752; 12486057; 10926369; 10812085; 9546662; 1625581 |
| NT06MT02154 | 8 | 20519912; 20043150; 19756584; 19156406; 11337471; 10610766; 2993631 |
| NT06MT03517 | 8 | 17483937; 16478453; 3236220; 15965735; 14769475; 3059995; 7813460; 11405892; 9931486; 2241929 |
| NT06MT0389 | 12 | 17280684; 12369934; 11121403; 9276481; 3087629 |
| NT06MT0447 | 8 | 20075070; 18434308; 728375; 8997717; 8706724; 13812439 |
| NT06MT0488 | 17 | 2867991; 15882422; 12694623; 12031481; 10438771; 9634751; 8226681; 1518043; 1906210; 2828153 |
| NT06MT0578 | 8 | 21388532; 21382109; 21378396; 21377964; 21377525; 21372178; 21368419; 21364950; 21364670; 21362505 |
| NT06MT0611 | 3 | 20843801; 20223296; 16595677; 10657653; 18178556; 17179146; 1904554; 10531370 |
| NT06MT09017 | 2 | 11342140; 17337559; 17826740; 10760138; 16554727; 16326705; 12657046; 16132864; 15581578; 10940244 |
| NT06MT1197 | 4 | 20304659; 17337559; 18020307; 17084627; 16630724; 16533988; 10742217; 15203162; 15185746; 14556638 |
| NT06MT1293 | 6 | 20211667; 19115962; 18977316; 11158121; 9311918; 16585648; 15338545 |
| NT06MT1321 | 17 | 18248429; 8432708; 15518067; 12535070; 12527296 |
| NT06MT1355 | 17 | 21277915; 21216748; 20971916; 20802044; 11719184; 2867991; 18682280; 18657035; 14638810; 10564489 |
| NT06MT1474 | 6 | 20420913; 20109556; 12782131; 18801049; 15328017; 2138057 |
| NT06MT1609 | 8 | 20831859; 20380465; 9504925; 20233940; 20184858; 20136502; 19961168; 19576290; 19248790; 10517866 |
| NT06MT1623 | 8 | 21194188; 21151867; 20873718; 20591829; 20550962; 20549202; 20221740; 20214643; 1938951; 3039297 |
| NT06MT1673 | 3 | 20398892; 20233920; 19416450; 19407160; 18781066; 18566149; 18402607; 17965356; 14888770; 17768255 |
| NT06MT1688 | 3 | 20675481; 20405122; 20350545; 18704529; 11536144; 18390543; 18210176; 17157337; 16887235; 16257960 |
| NT06MT1788 | 15 | 20144684; 10629763; 18649864; 8402178; 1528892; 1633609; 1828858; 2449695; 1669444; 2270287 |
| NT06MT1803 | 18 | 18489257; 9918945; 11799204; 15728904; 17041047; 11027260; 18774242; 7514039; 18692158; 12096107 |
| NT06MT18337 | 8 | 18056994; 19787709; 19661995; 19434406; 10859321; 19056199; 18850694; 16500709; 18473959; 18363338 |
| NT06MT1968 | 8 | 20722380; 19819140; 12816877; 19196840; 1471713; 18081310; 17980168; 17909855; 17385561; 16843508 |
| NT06MT2310 | 12 | 20185505; 18550062; 17379330; 15845365; 11932449; 10852868; 11085286; 13278318; 10731398; 10670188 |
| NT06MT2318 | 15 | 21378313; 21358672; 21348938; 21332845; 21327099; 21323640; 21319304; 21304601; 21291890; 21283566 |
| NT06MT2334 | 12 | 21392555; 21392541; 21392503; 21392500; 21392490; 21392184; 21390546; 21390545; 21389614; 21389348 |
| NT06MT2344 | 6 | 15538360; 11952905; 10902162; 11590160; 1738203; 8843438 |
| NT06MT2464 | 2 | 20363939; 3032913; 4874308; 14898026; 9446573; 7929373; 8425548; 6312261 |
| NT06MT2568 | 4 | 11504612; 16779844; 16229464; 15703173; 14675764; 11504612; 11473257 |
| NT06MT2677 | 12 | 21376533; 21346060; 21339741; 21321311; 21304107; 21292774; 21257751; 21249316; 21238599; 21238542 |
| NT06MT2829 | 1 | 18260104; 16078071; 15596430; 15336409; 12906820; 12906831; 12624088 |
| NT06MT3006 | 18 | 16923166; 9971832; 15702067; 8910491; 8721982 |
| NT06MT3215 | 6 | 21394958; 21394906; 21394879; 21394829; 21394807; 21394759; 21394739; 21394635; 21394607; 21394548 |
| NT06MT3371 | 8 | 21210253; 20947827; 20935003; 20925195; 20846129; 20833273; 20659429; 20586862; 20587039; 20379791 |
| NT06MT3393 | 15 | 21390242; 21388403; 21383171; 21382340; 21376121; 21339227; 21333377; 21330520; 21321124; 21298052 |
| NT06MT34932 | 2 | 21296217; 19779138; 18426076; 17868200; 17537870; 17125408; 17072089; 16488120; 16406305; 16296944 |
| NT06MT3541 | 15 | 21393832; 21329667; 21124051; 20935121; 20595046; 20472642; 20466770; 20225260; 20190084; 11553605 |
| NT06MT3626 | 17 | 21220116; 19016841; 18757812; 18722345; 17485404; 16263329; 10227159; 8039908; 7840550; 7476207 |
| NT06MT3754 | 8 | 21208358; 18807715; 17651430; 9370141; 7578618; 8171273; 8214809; 2268339; 6508215; 6712147 |
| NT06MT3759 | 17 | 21277915; 20971916; 20233303; 20233302; 16804177; 10564489; 11355567; 17379215; 11018124; 16556970 |
| NT06MT3925 | 8 | 21199252; 21179059; 21068384; 21068201; 20937244; 20840839; 21198452; 20808844; 20852605; 20730594 |
| NT06MT3953 | 4 | 21085634; 20675473; 16030141; 2166035; 10715012; 18439873; 10456919; 15063064; 16763111; 10545259 |
| NT06MT4049 | 15 | 19202108; 11114919; 12686116; 11158353; 9389448; 9168623; 7582014; 4896022; 881736; 8392137 |
| NT06MT4072 | 2 | 11148030; 17212407; 11007789; 10966576; 1361170; 2234077; 1658539; 377280 |
| NT06MT4288 | 13 | 21071662; 20360175; 19745807; 20155482; 19745807; 10666455; 11283358; 18226598; 18604630; 15146073 |
| NT06SF06952 | 2 | 15645437; 14529496; 12077433; 11375500; 8430515; 7890752; 4290215 |
| NT06SF1018 | 18 | 6297798; 2125747; 14504659; 11705403; 9915792 |
| NT06SF1232 | 5 | 21393366; 21393052; 21392197; 21382037; 21375387; 21365232; 21357454; 21338415; 21334214; 21322495 |
| NT06SF1473 | 17 | 20227844; 20087629; 19936829; 19528202; 11349022; 19467815; 7501460; 18983256; 19329591; 18843597 |
| NT06SF17884 | 8 | 20187119; 18594899; 17485831; 101676; 12026175; 9782510; 10821190; 10669803; 8366066; 8244973 |
| NT06SF1827 | 4 | 17971082; 15225981; 12688630; 12672520; 11410278; 11233154; 10841776; 10681558; 10644761; 10448080 |
| NT06SF1848 | 15 | 20847010; 18511939; 19729089; 18323620; 16204505; 16672238; 10700279; 11495988; 10679470; 17194626 |
| NT06SF21117 | 8 | 21377755; 20972263; 20935143; 20799687; 20298244; 19875437; 19465440; 15287594; 4372945; 19028504 |
| NT06SF2649 | 15 | 21389327; 21376034; 21368058; 21353395; 21346244; 21343335; 21292824; 21281310; 21270259; 21268086 |
| NT06SF2682 | 3 | 20633226; 20213113; 7942316; 19096097; 18957858; 11976147; 18407824; 18323643; 18321602; 18050911 |
| NT06SF2932 | 8 | 19387485; 15972253; 15491156; 12095623; 11939777; 10368307; 11223519; 9305729; 9022686; 6541459 |
| NT06SF32207 | 8 | 21330005; 20545743; 16313613; 14704707; 15289572; 11948863; 8486283; 12054436; 6327463 |
| NT06SF33987 | 8 | 19807880; 19523599; 16565040; 18281746; 17638114; 15313216; 1311073; 12374297; 2562762; 10945221 |
| NT06SF3938 | 8 | 21350343; 21349748; 21338242; 21328435; 21300801; 21289127; 21279680; 21267634; 21248073; 21246536 |
| NT06SF40302 | 2 | 21255096; 21071441; 20954240; 20943913; 20943658; 20926655; 20926389; 20857515; 20857400; 20838591 |
| NT06SF4535 | 18 | 21278293; 20846937; 20802073; 20799747; 20696823; 20647000; 10829079; 20352420; 20335169; 20299406 |
| NT07CD0313 | 3 | 21303909; 20797482; 20661636; 20647050; 18187606; 20096375; 20045741; 20026284; 19836342; 19783652 |
| NT07CD0493 | 17 | 20924357; 11580842; 9882659; 14514681; 12829297; 12777497; 12724394; 9836599; 1382312; 5335892 |
| NT07CD0675 | 8 | 21270770; 21196308; 21125339; 21045145; 21041650; 20919991; 20863288; 20852181; 20832300; 20831857 |
| NT07CD0682 | 6 | 20667510; 20530453; 20434457; 20005284; 19968858; 18715143; 19896490; 19749384; 9766671; 19463900 |
| NT07CD1112 | 11 | 21214923; 21204936; 21041684; 20817773; 20543830; 20531477; 20160083; 2111116; 18757924; 19241380 |
| NT07CD1243 | 4 | 12614149; 16102006; 10369683; 8052128; 10708390; 1595905 |
| NT07CD2028 | 1 | 18216013; 10913262; 17490766; 12453224; 10998174; 1943695 |
| NT07CD2110 | 6 | 21390305; 21288882; 21278412; 21274659; 21241707; 21239579; 21185381; 21185379; 21165603; 21087076 |
| NT07CD2236 | 18 | 19268700; 18627004; 17541839; 16364310; 16212599 |
| NT07CD2478 | 12 | 21362190; 21281692; 21278271; 21261463; 21261072; 21234230; 21220481; 21188150; 21173569; 21124876 |
| NT07CD2939 | 18 | 20334581; 19959708; 12374799; 19692482; 18348195; 12515852; 17975670; 17595523; 16788137; 16734747 |
| NT07CD3028 | 18 | 21184302; 21129777; 21094732; 21051486; 21035733; 20944066; 20878800; 20873968; 20862359; 20850558 |
| NT07CD3705 | 6 | 21276852; 21248859; 21073053; 21060849; 21050827; 20833188; 20603082; 20421420; 9585556; 1526964 |
| NT07CD3921 | 18 | 19758487; 15740445; 16039155; 12154069; 8989880; 10388563; 3301539; 7557949; 15335687 |
| NT07ST0267 | 12 | 21320870; 21301034; 21247409; 21215656; 20671064; 20548119; 20540986; 20469960; 20140374; 19732828 |
| NT07ST0532 | 12 | 21270253; 21155711; 21094632; 20868794; 20719862; 17024418; 20617897; 19134538; 19852035; 7532381 |
| NT07ST1072 | 18 | 20666225; 20655873; 20584751; 20163552; 20026328; 20012941; 19802720; 19724867; 17589596; 12354618 |
| NT07ST11197 | 8 | 20534506; 20237288; 10576686; 18674612; 18206123; 16780921; 16033338; 15893774; 15778121; 15381403 |
| NT07ST1235 | 12 | 21173569; 21058505; 20667621; 20107983; 19845284; 19162325; 19028863; 17786425; 17630120; 17571258 |
| NT07ST1341 | 6 | 10606644; 6288254; 15629722; 15503140; 11821933; 11254128; 10431172 |
| NT07ST1456 | 8 | 21166768; 20880332; 19895419; 20560066; 7553938; 17668018; 11053379; 17074898; 16944099; 16908186 |
| NT07ST1677 | 1 | 21387027; 21385868; 21282100; 21132375; 20704760; 20667835; 20570618; 19910325; 19589834; 15286989 |
| NT07ST1745 | 4 | 21040514; 14617176; 12123453; 1729224; 165169; 2785104 |
| NT07ST1818 | 12 | 21394418; 21381035; 21366683; 21352278; 21275939; 21196708; 21182502; 21178586; 21139684 |
| NT07ST1896 | 2 | 21038112; 20606263; 20221527; 15966871; 19946146; 16632608; 19470097; 18522945; 16756317; 18092812 |
| NT07ST2004 | 3 | 20602334; 20331963; 18563288; 18387370; 18355438; 16284928; 16280320; 16055313; 15913610; 15669674 |
| NT07ST2616 | 8 | 16458324; 15668249; 12488095; 12466884; 11751050; 11243831; 11050089; 10222208; 9562556; 4296830 |
| NT07ST2779 | 3 | 21393206; 21356201; 21255117; 21240183; 21183069; 21183069; 21106176; 20956585; 20847002; 20726894 |
| NT07ST2844 | 4 | 20878432; 20807542; 19567751; 17526652; 17601516; 8137493; 16600288; 16477617; 15777620; 15191886 |
| NT07ST2865 | 12 | 11750807; 7761092; 16361710; 9015299; 10412982 |
| NT07ST2903 | 18 | 21194120; 20936291; 20858756; 20831043; 20827334; 20703447; 21226590; 20576528; 20540435; 20529095 |
| NT07ST3059 | 8 | 20980436; 20973793; 20960097; 20920510; 20705058; 20702724; 20632369; 20618733; 20600565; 20583616 |
| NT07ST31541 | 8 | 20661555; 20638314; 20589904; 20523973; 20512975; 20507884; 20216285; 20024545; 19490731; 19465673 |
| NT07ST3212 | 8 | 20937819; 20837795; 20826797; 20823090; 20816962; 20637638; 20627642; 20625049; 20610779; 20547137 |
| NT07ST3215 | 8 | 21144833; 20643099; 20571113; 20547137; 20505714; 20459120; 20398675; 20019223; 19616643; 19527690 |
| NT07ST3216 | 8 | 20846527; 20613764; 20505714; 19815558; 19181534; 18799460; 18760846; 18560889; 18501578; 18243814 |
| NT07ST3219 | 8 | 20628895; 12788651; 19815558; 18809504; 16402904; 16608357; 18291703; 17662684; 16150735; 17114648 |
| NT07ST3279 | 3 | 21183069; 21183069; 345275; 20023035; 19623961; 18539953; 18431569; 18266856; 18186792; 17501913 |
| NT07ST3462 | 3 | 20935096; 20676082; 20586476; 16487743; 20543140; 20444091; 20439729; 20421493; 20398208; 20351136 |
| NT07ST3586 | 15 | 21362064; 21360181; 21304599; 21299643; 21295415; 21267402; 21257771; 21249192; 21245528; 21243338 |
| NT07ST3600 | 15 | 21178479; 21151497; 21067162; 19901023; 16629664; 17824925; 8657312; 18279344; 17827157; 10629189 |
| NT07ST3631 | 4 | 21394603; 21392605; 21391840; 21391435; 21390543; 21390528; 21388249; 21387234; 21386728; 21385579 |
| NT07ST3651 | 8 | 20335176; 8389295; 16348282; 1850416; 6307354 |
| NT08MA0078 | 6 | 21274469; 21249131; 21229971; 21163286; 21130077; 21135864; 21045206; 20980350; 20979427; 20939822 |
| NT08MA0137 | 8 | 18681889; 18237273; 17892308; 16820168; 16491912; 15900210; 15063311; 9660187; 9374858 |
| NT08MA0198 | 12 | 21393844; 21388709; 21385615; 21383157; 21382368; 21381077; 21380642; 21378312; 21377964; 21372178 |
| NT08MA0208 | 8 | 21393246; 21292982; 21288761; 21269500; 21266547; 21257336; 21226084; 21184294; 21148731; 21138528 |
| NT08MA0306 | 2 | 20085893; 19231875; 10737935; 10395796; 17388809; 15805601; 17176045; 17017801; 16781731; 16471696 |
| NT08MA0319 | 18 | 21192796; 20713660; 20385866; 19749294; 8626331; 19151142; 18619818; 17943119; 820685; 6785365 |
| NT08MA0332 | 3 | 20731789; 20035716; 19610666; 19459932; 18795799; 19482107; 18795799; 18247575; 18045869; 9914259 |
| NT08MA0426 | 6 | 21360615; 21266475; 21263027; 21244633; 21227929; 21189691; 21173259; 21173200; 21154877 |
| NT08MA0480 | 5 | 20635345; 20631318; 20207756; 10194322; 7565414; 10368287; 16404152; 2211515; 14981304; 14749331 |
| NT08MA0590 | 14 | 21394109; 21342105; 21333030; 21300806; 21293377; 21251930; 21189470; 21176733; 21151102; 21150937 |
| NT08MA0680 | 8 | 21304897; 21153630; 21143720; 21069157; 20937805; 20880840; 20849861; 20797423; 20702405; 20688914 |
| NT08MA0734 | 2 | 20526342; 17855635; 17374725; 16873928; 16643834; 16501255; 16116126; 15604701; 2611960; 11744717 |
| NT08MA0743 | 3 | 8662184; 19416510; 10978324; 12511511; 16162494; 8112463; 405375; 25767; 3521530; 3521529 |
| NT08MA0802 | 8 | 21323311; 21299470; 21275844; 21235502; 21210868; 21194355; 21190518; 21094149; 21078302; 21044985 |
| NT08MA0859 | 8 | 21391540; 21345217; 21320349; 21270003; 21230323; 21223639; 21221542; 21191537; 21158478; 21133836 |
| NT08MA0884 | 3 | 20400549; 20139182; 18837509; 15922833; 15983044 |
| NT08MA1216 | 17 | 21124318; 20574006; 20480367; 20227504; 20219874; 19951946; 8884268; 19592490; 19418092; 19282365 |
| NT08MA13505 | 2 | 21209101; 21161331; 20705584; 12902985; 20580936; 20552454; 20552438; 20538039; 3372162; 20477760 |
| NT08MA1406 | 9 | 21081696; 20605911; 20594840; 20557983; 20418430; 20304657; 20221630; 20178986; 20158476; 20153183 |
| NT08MA14155 | 2 | 21377758; 21229377; 21208309; 21139430; 21118486; 21116859; 21112837; 20949119; 20887710; 20872012 |
| NT08MA1550 | 17 | 17977831; 9477255; 9733698; 10564489; 9116041; 2142347; 1368828 |
| NT08MA1713 | 18 | 21394349; 21394088; 21393468; 21393446; 21392380; 21391982; 21390223; 21387375; 21385718; 21383163 |
| NT08MA1722 | 3 | 21098023; 21076070; 21073956; 21073425; 20885977; 20855895; 20583802; 20559986; 20538331; 20526345 |
| NT08MA1739 | 12 | 21338918; 21338912; 21335977; 21326941; 20848881; 20827447; 20803087; 20735358; 20727857; 20525865 |
| NT08MA1775 | 11 | 20359459; 20107991; 2231712; 19560223; 14730025; 17719266; 16349277; 17048701; 16349277; 9139913 |
| NT08MA1899 | 8 | 21349599; 21308951; 21255384; 20836154; 20677779; 20487432; 20359237; 20112984; 17694358; 19625206 |
| NT08MA1937 | 12 | 20882995; 19734307; 8692985; 16342964; 15740115; 15642479; 15362861; 12860390; 10966480; 8206978 |
| NT08MA1976 | 11 | 20079432; 19623961; 10603364; 8598202; 10496929; 10489437 |
| NT08MA2103 | 8 | 21220358; 21054068; 20628047; 20429279; 20093275; 19928586; 18535010; 19758831; 19616485; 18655808 |
| NT08MA2458 | 9 | 20176020; 20057061; 19686777; 19555075; 19462023; 19118366; 19052370; 18338425; 18027023; 11373295 |
| NT08MA2826 | 12 | 20136635; 19460333; 18201985; 11600844; 17147490; 1380789; 9662192; 2476069 |
| NT08MA3031 | 4 | 17637339; 17303131; 12828367; 14556641; 10956034; 1281482 |
| NT08MA3170 | 4 | 21375698; 21365005; 21360573; 21348612; 21348606; 21345913; 21340650; 21328346; 21325249; 21320786 |
| NT08MA3242 | 5 | 19770075; 18198899; 17289450; 11893063; 12646375; 15882995; 12770504; 12445781; 11750815; 11734883 |
| NT08MA3317 | 1 | 21106925; 20817725; 20482655; 20498299; 20332210; 20213441; 19948253; 19931317; 19875435; 19710390 |
| NT08MA34692 | 4 | 21332358; 21233390; 21149676; 21113137; 20934870; 12477935; 20513390; 20299597; 20231482; 20178739 |
| NT08MA3525 | 2 | 21292977; 20823551; 20565974; 20465413; 20430628; 19216536; 19748579; 19716309; 16984393; 14019094 |
| NT08MA3539 | 12 | 21262231; 21233422; 21045016; 20849853; 20819954; 20815828; 20697605; 20691256; 20669962; 20811336 |
| NT08MA3603 | 6 | 21150334; 21128641; 20828134; 20444223; 20438097; 20418333; 20305004; 19945437; 19944689; 19888752 |
| NT08MA3624 | 17 | 11524010; 11318656; 10769129; 10529249; 9709002 |
| NT08MA3656 | 15 | 2002000; 11722727; 19646451; 10852888; 17609139; 10731426; 10647185; 9837737; 1624413; 7855435 |
| NT08MA3779 | 6 | 21327072; 21321201; 21315180; 21314619; 21245380; 21245167; 21239472; 21174446; 21168469; 21163286 |
| NT08MA39945 | 8 | 12631287; 15490883; 12461137; 7753027; 8343600; 1450384; 1885590; 1907512; 1714358; 2503398 |
| NT08MA4019 | 8 | 18781696; 11154287; 17570335; 16721829; 15174056; 12889023; 12709489; 12667485; 12489122; 11549245 |
| NT08MA4105 | 12 | 20970375; 19914209; 19291145; 15305923; 15736957; 10611680; 2545583 |
| NT08MA41142 | 4 | 11759840; 11913779; 8200522; 7683649; 11607099; 1282192; 1404376; 1956294 |
| NT08MA4129 | 8 | 21337371; 21283685; 21276816; 21270770; 21269365; 21234393; 21234284; 21196308; 21174642; 21138736 |
| NT08MA4174 | 8 | 21320626; 21270901; 21258134; 21241708; 21117169; 20962922; 20833871; 20732951; 20707314; 20655901 |
| NT08MA4265 | 3 | 11279221; 2180947; 9242624; 7481807; 7567967; 8366125; 1495425 |
| NT08MA4270 | 2 | 20359225; 20054118; 19462053; 19389784; 10639366; 10829079; 234963; 16434053; 16248620; 16042594 |
| NT08MA43825 | 2 | 11461190; 7635153; 8590013; 7763270; 8001680; 7923811; 8188266; 8444860 |
| NT08MA4481 | 8 | 21273093; 21177309; 21079553; 21078914; 20924575; 20864416; 20805371; 20704567; 20683684; 20630208 |
| NT08MA4493 | 5 | 20635345; 20207756; 19476442; 11123699; 11123699; 9712811; 7565414; 10368287; 16404152; 16152655 |
| NT08MA4497 | 6 | 21333363; 21290544; 21289624; 21289055; 21288647; 21281594; 21266537; 21254160; 21245380; 21242961 |
| NT08MA4498 | 2 | 16299185; 19761441; 18781696; 17618627; 16668905; 17978581; 17911033; 17906139; 16665411; 16524713 |
| NT08MA4547 | 12 | 20931876; 20382837; 2684968; 1847526; 15598653; 16221315; 9593850; 12940952; 12927408; 12546003 |
| NT08MA4706 | 8 | 21335525; 21196476; 21194355; 20876336; 20616155; 20511298; 20191562; 20100520; 20049866; 19825629 |
| NT08MA50687 | 8 | 20690683; 20381366; 19754880; 19508275; 16169685; 18776203; 9880484; 16518698; 17339651; 17295469 |
| NT08MA5104 | 15 | 21035448; 20589935; 19351745; 19211160; 18094251; 18025410; 17975702; 17725581; 17553500; 17263349 |
| NT08MA5157 | 5 | 21236489; 21055403; 20450939; 20394743; 19941308; 19785954; 19589348; 19286931; 10693141; 18832451 |
| NT08MA5223 | 6 | 21390132; 21389547; 21383776; 21364740; 21362621; 21362547; 21360615; 21358597; 21357745; 21355561 |
| NT08MA52375 | 8 | 20604506; 20599235; 20120238; 20059735; 15650338; 19274447; 19157743; 19076303; 19018936; 18343499 |
| NT08MA5244 | 4 | 20100873; 20100826; 19481127; 17085596; 15450492; 18232718; 17827974; 17464078; 17046525; 16377622 |
| NT08MA52637 | 8 | 21322090; 21284755; 21283636; 21246047; 21192085; 21112065; 21095462; 21048708; 21085165; 20975902 |
| NT08MA5470 | 8 | 21384180; 21378031; 21377964; 21372133; 21369811; 21366233; 21364629; 21364306; 21359198; 21357526 |
| NT08MA54905 | 2 | 21388533; 21379374; 21335601; 21296950; 21266015; 21143723; 20718073; 20660776; 20552454; 20491066 |
| NT08MA5540 | 6 | 12522255; 1427081; 12940820; 11700277; 12408833; 11207366; 10421637; 10844644; 10662672; 10543946 |
| NT08MA5622 | 6 | 21219854; 21205014; 21124948; 21079801; 20861182; 20813592; 20666462; 20628184; 20334433; 20223211 |
| NT08MA5786 | 17 | 20729364; 18757812; 17661085; 10438773; 17462019; 16909271; 15881404; 15518067; 12527296; 12111561 |
| NT08MA5956 | 17 | 12949700; 10564489; 10050033; 12501349; 9003451; 8947837; 1511015; 1368828 |
| NT08MA5985 | 12 | 21094732; 21078557; 21068254; 21029044; 20960971; 20949197; 20926390; 20883728; 20854850; 20833209 |
| NT08MA6159 | 8 | 21308129; 21110748; 21082238; 21040711; 20960123; 20628047; 20599452; 20588188; 20559622; 20079506 |
| NT08MA6176 | 8 | 21329464; 20851451; 10618710; 20554899; 20387640; 20359164; 19715479; 15489192; 19188863; 17158597 |
| NT08MA6291 | 12 | 17259602; 16751195; 2199796; 15838638; 14622420; 14600218; 12808018; 12240967; 11700363 |
| NT08MA6364 | 12 | 19788412; 19002655; 18708056; 18187560; 18023241; 17760891; 11359610; 17094011; 16179350; 12446677 |
| NT08MA6372 | 5 | 1766381; 19520724; 11595639; 17567048; 9144792; 16630633; 16568996; 16232619; 15996110; 10411743 |
| NT08MA6384 | 6 | 20975945; 20699139; 19692579; 19414019; 12529507; 16978259; 7836279; 16297462; 15165237; 15133105 |
| NT08MA6500 | 1 | 20086163; 20057066; 19559030; 19307721; 19013471; 18269631; 18023379; 17889830; 11495997; 17465479 |
| NT08MA65335 | 2 | 21318489; 20930116; 20164151; 19843012; 19264498; 18959027; 18810749; 18317702; 17996014; 17978586 |
| NT08MA6548 | 6 | 21083655; 21030440; 20889748; 20219261; 2225075; 19923746; 17905994; 19538463; 20439528; 19478444 |
| NT08MA6576 | 5 | 21167020; 21119624; 20722014; 20605227; 20206618; 17711875; 20109544; 19682969; 19458963; 19308726 |
| NT08MA67897 | 8 | 11160798; 942051; 1791759; 3350791; 1916282; 1366442; 3269389; 2982815 |
| NT08RS0018 | 4 | 21071627; 20942908; 20378989; 7989325; 7819201; 15007097; 15882426; 11432834; 11404360; 10510225 |
| NT08RS00327 | 8 | 21314134; 21294156; 21247893; 21206954; 21146812; 21124396; 21087665; 21043458; 20935102; 20857447 |
| NT08RS02847 | 8 | 19801601; 18239430; 17787008; 17684016; 17137300; 16260293; 16119842; 14706841; 14655000; 10482501 |
| NT08RS02857 | 8 | 20528775; 19807880; 17215140; 17080620; 12226404; 8300616 |
| NT08RS0741 | 8 | 20652236; 18691525; 7984417; 16958750; 9187299; 8028028; 1892826; 3061453 |
| NT08RS10345 | 8 | 18798732; 17922301; 16228472; 8460167; 10217763; 8695630; 9130598; 8636035; 8450304; 5432063 |
| NT08RS11991 | 8 | 20802079; 20667419; 20637832; 20589904; 20512975; 20507884; 383576; 20221814; 20164409; 19852484 |
| NT08RS1212 | 18 | 20031009; 15917616; 10940570; 9461423; 7922040; 9274047; 7765086; 8254318 |
| NT08RS1573 | 11 | 21394605; 21376090; 21349975; 21345356; 21320307; 21284991; 21255609; 21220524; 21215360; 21204947 |
| NT08RS17921 | 8 | 20361235; 20156148; 19558963; 19266906; 19054080; 16589675; 17619232; 17503033; 15630578; 15296170 |
| NT08RS1812 | 8 | 21372092; 20963615; 19622864; 18930018; 15632441; 12595732; 11327772; 10524262; 9809418; 8977120 |
| NT08RS1814 | 15 | 20729362; 20608176; 8474334; 20012992; 19888817; 12813079; 18616406; 17768260; 16574415; 15929010 |
| NT08RS1866 | 11 | 21129201; 20427067; 20160083; 20093122; 15837183; 17437718; 17098191; 16193532; 16932843; 16876823 |
| NT08RS1906 | 4 | 21389833; 21297337; 21292824; 21281802; 21274670; 21258824; 21256839; 21225871; 21223584; 21205088 |
| NT08RS1966 | 15 | 2835094; 3294077; 2898473; 2842595; 3689756; 3030405; 3525560; 6487597; 5167087 |
| NT08RS2000 | 8 | 20675294; 16820168; 16027125; 15946648; 15914915; 3790506; 11870062; 11330998; 7543100; 1939012 |
| NT08RS2071 | 8 | 6259625; 14020053; 1195397; 2050654; 3013855 |
| NT08RS2270 | 11 | 20890269 |
| NT08RS25451 | 8 | 20661960; 19579240; 18215050; 2834341; 9047371; 15669071; 12962479; 12206758; 11976494; 11732896 |
| NT08RS2650 | 8 | 20888212; 20378650; 19428474; 19151163; 19030736; 18969149; 17603262; 17221239; 17082757; 17031028 |
| NT08RS32294 | 8 | 21298809; 21266107; 21086456; 20976701; 20920617; 20452841; 19054103; 20144715; 20032185; 19852966 |
| NT08RS3334 | 18 | 21338480; 21333794; 21301927; 21241807; 21219471; 21182592; 21167943; 21149455; 21115718 |
| NT08RSA0017 | 8 | 10880957; 9839943; 8621665; 8443213; 1710985 |
| NT08RSA0187 | 8 | 21384215; 21348884; 21339865; 21338053; 21322643; 21240533; 21112606; 21045317; 20709842; 20675088 |
| NT08RSA0247 | 8 | 15667266; 10924909; 8764511; 869173; 2094292; 2215219; 2325631 |
| NT08RSC0011 | 9 | 8672472; 2376563; 2351678; 2186848; 2698265; 6366102; 6929858 |
| NT09HA0201 | 15 | 21205103; 19888830; 10376820; 10869437; 17254603; 10077542; 10515925; 16415592; 16415591 |
| NT09HA0224 | 12 | 20729206; 20154089; 18672675; 17646163; 17624491; 17573191; 16982626; 9454735; 16740134; 16081615 |
| NT09HA0462 | 12 | 20494587; 19915005; 19795569; 15574921; 19152630; 18673454; 10968716; 16926147; 9746589; 12904566 |
| NT09HA1983 | 18 | 18023627; 14670099; 8322516; 1784630; 3546517 |
| NT09HA2004 | 18 | 21385383; 21285061; 21270827; 20861854; 21220329; 21211658; 21193909; 21179412; 21173243; 21149604 |
| NT09HA2316 | 12 | 21369989; 21276854; 21271976; 21058505; 20306186; 20039036; 19902383; 19697018; 19669756; 19609954 |
| NT09HA2587 | 3 | 20919961; 10362118; 20540529; 20369747; 20192807; 19936627; 11119490; 15870928; 7979551; 20179984 |
| NT09HA29454 | 8 | 20659797; 20542114; 20347285; 20100576; 20036323; 18329885; 18301900; 17897213; 17487829; 17372347 |
| NT09HA31814 | 8 | 21335027; 21327413; 21315381; 21314956; 21229334; 21192716; 21138057; 21118988; 21114521; 21104698 |
| NT09HA3334 | 8 | 21383264; 21383134; 21377879; 21377519; 21373759; 21371861; 21371464; 21370258; 21360301; 21359973 |
| NT09HA3438 | 8 | 12678433; 12615344; 11695833; 11295131; 11222389; 2172645 |
| NT09HA3673 | 17 | 9384377; 17338440; 10419520; 9683469; 14993308; 7545758 |
| NT09HA3890 | 12 | 21292286; 21246632; 21236262; 21227434; 21178444; 21174470; 21150094; 21129427; 21126825; 21112953 |
| NT09HA4055 | 2 | 8873617; 1629163; 12615349; 6830756; 8626063; 8626063; 1663748; 2209599; 3516220; 942051 |
| NT09HA5136 | 8 | 21185308; 21136597; 21115336; 21104949; 20841500; 20817397; 20493950; 19682288; 19756991; 19505469 |
| NT09HAA0242 | 11 | 20562304; 15500249; 12206751; 10875286; 10822806; 10801346; 2172216; 10476039; 9742696; 895716 |
| NT09RC0007 | 18 | 21265952; 21259244; 21156276; 21135503; 21106850; 21106247; 21078874; 20858096; 21071089; 21062745 |
| NT09RC0067 | 6 | 21262799; 21223577; 20478826; 8995522; 18363794; 10829079; 17944831; 10048034; 16407074 |
| NT09RC0084 | 6 | 21276852; 20833188; 12778123; 18804481; 10606644; 12730195; 9722634; 16879643; 6288254; 15629722 |
| NT09RC0132 | 15 | 20417183; 12002822; 18279349; 9882660; 7753029; 15667993; 1718867; 11572979; 4978942; 10348851 |
| NT09RC01677 | 8 | 21380435; 21115813; 19446033; 20171005; 19406801; 19343343; 19126402; 19086182; 18955077; 12215515 |
| NT09RC0194 | 8 | 21337322; 19731367; 18819910; 18563633; 17823972; 10112; 16752391; 16697227; 15752362 |
| NT09RC0201 | 8 | 21172057; 20924576; 20798530; 20687591; 1588793; 20435898; 20412798; 20208169; 20183604; 20183598 |
| NT09RC0247 | 6 | 21219854; 20813592; 20727340; 20512791; 20337945; 20222445; 20203672; 8662184; 20122408; 19920184 |
| NT09RC0285 | 15 | 19233289; 19825547; 17259177; 12673057; 12167640; 11728710 |
| NT09RC0314 | 17 | 21221925; 20724091; 20227844; 20087629; 19936829; 19528202; 11349022; 19467815; 7501460; 18983256 |
| NT09RC03320 | 4 | 21905957; 21905665; 10407139; 21897026; 21896552; 21896551; 21895720; 21895715; 21888658; 18801906 |
| NT09RC0419 | 13 | 21357927; 21321019; 21222438; 21098258; 21149735; 20942128; 20504770; 20387531; 20160114; 20059602 |
| NT09RC0434 | 6 | 21299643; 20360008; 9640531; 19880633; 19846353; 16780573; 19399845; 19308706; 18683630; 2659796 |
| NT09RC04695 | 2 | 20017145; 19954226; 19897889; 19852513; 19699210; 18699781; 17973380; 18190517; 16632253; 17658461 |
| NT09RC0499 | 13 | 20466058; 20462952; 19917606; 19516019; 11172066; 18948083; 1793612; 10497258; 9671815; 18407920 |
| NT09RC0501 | 8 | 21385868; 21370850; 21364976; 21342411; 21288652; 21286325; 21281627; 21268892; 21233279; 21219616 |
| NT09RC0528 | 12 | 21030485; 1672770; 20209494; 17384282; 19818866; 19296605; 18389094; 18366456; 17294723; 10200556 |
| NT09RC0562 | 3 | 20799091; 20638049; 11386933; 20605777; 20599580; 20510395; 20453897; 1726852; 19556292; 8386125 |
| NT09RC0616 | 3 | 21356201; 21216228; 21183069; 21183069; 20849416; 345275; 20497333; 20302515; 20226679; 20087619 |
| NT09RC0666 | 12 | 19220400; 16330397; 10075426; 8890894; 8687979; 1808457; 2085376; 13673139; 7042754; 6368549 |
| NT09RC0728 | 6 | 10482496; 2670548; 12898065; 11700277; 8428576; 1409708 |
| NT09RC0789 | 8 | 16534744; 16268782; 12832797; 12729763; 11682175; 10691985; 10463150; 8260624; 7584858; 1535626 |
| NT09RC0811 | 15 | 17630969; 17379732; 10411727; 10851001; 12650774; 10517722 |
| NT09RC0820 | 8 | 21389620; 21385584; 21335525; 21329881; 21295157; 21210868; 21199936; 21130898; 21082862; 21081474 |
| NT09RC0843 | 2 | 20876192; 20180265; 19636932; 18391442; 17305325; 10222271; 17229734; 16884311; 16042595; 15966718 |
| NT09RC0869 | 6 | 11389467; 18606573; 17873859; 12724407; 12206670; 10097074; 11152689; 11090620; 11009612; 10610789 |
| NT09RC0895 | 14 | 21393637; 21388430; 21383964; 21378751; 21370050; 21370046; 21370045; 21370044; 21349283; 21332653 |
| NT09RC0902 | 8 | 19923443; 19519451; 19159951; 16678437; 18537677; 10376999; 17056133; 10487754; 16357825 |
| NT09RC0970 | 8 | 20534465; 19968794; 17929940; 16218868; 15757669; 15237985; 12633857; 12492476; 11852093; 11322875 |
| NT09RC0984 | 8 | 21194833; 21169450; 21166645; 21104284; 21072362; 21069149; 20858778; 20601224; 20392998; 20530407 |
| NT09RC1040 | 8 | 21104284; 19780833; 19585534; 19425498; 19420686; 18586973; 18993058; 17681537; 18051362; 12949096 |
| NT09RC1060 | 6 | 21382489; 21380757; 21365680; 21356109; 21348848; 21344949; 21338723; 21331045; 21330406; 21328508 |
| NT09RC10750 | 4 | 19247286; 19804784; 19636618; 18378708; 17408365; 18833010; 18789328; 17725563; 17106680; 10629175 |
| NT09RC1123 | 8 | 19055545; 11018134; 16634330; 10960103; 12693814; 12612960; 11293064; 10757039; 10463157; 10403335 |
| NT09RC1168 | 9 | 20924576; 20511508; 20463028; 20167774; 20110695; 20035485; 19903863; 19659692; 19452559; 19213219 |
| NT09RC1285 | 14 | 21392535; 21387833; 21387452; 21387205; 21386633; 21382338; 21376411; 21375466; 21373931; 21371294 |
| NT09RC1299 | 3 | 6295886; 17120230; 1569582; 15299374; 16236703; 15200939; 14659546; 12757953; 12628682 |
| NT09RC1306 | 3 | 20804196; 20024979; 19403924; 19369074; 19198900; 19014883; 19007109; 18704940; 18557704; 18259126 |
| NT09RC1426 | 12 | 21309513; 21182296; 20881185; 20811767; 20799977; 20688819; 20654624; 20622127; 1671555; 20453896 |
| NT09RC1438 | 9 | 20023301; 8624514; 19179086; 18808455; 18088304; 17932115; 16824521; 16762032; 16009708; 15277243 |
| NT09RC14661 | 8 | 21382014; 21255309; 20816933; 19968989; 11243887; 19733060; 19690850; 19473250 |
| NT09RC1510 | 8 | 21345803; 20974856; 20566710; 20154086; 20026007; 19785575; 9882406; 17983592; 17697991; 7929329 |
| NT09RC1511 | 8 | 21345803; 21326874; 21193405; 21106936; 21035425; 20843806; 20723362; 20663914; 20626349; 11448154 |
| NT09RC1512 | 8 | 21345803; 21326874; 21193405; 20974856; 20843806; 20170625; 20154086; 9657145; 9882406; 9397682 |
| NT09RC1514 | 8 | 17766239; 7929329; 12788493; 9632682; 16697972; 16608846; 16226039; 16120288; 16045926; 15339903 |
| NT09RC1515 | 8 | 21354629; 21106936; 5233469; 16085645; 14740887; 12887009; 12724321; 12516783; 11768302; 11768298 |
| NT09RC1516 | 8 | 20727984; 7934937; 17234633; 5233469; 18760846; 17619138; 17614033; 10998052; 17234633; 16803979 |
| NT09RC1544 | 8 | 21106986; 20832300; 20669055; 20370616; 20050917; 19966411; 19543838; 19352649; 18988465; 18983825 |
| NT09RC1637 | 12 | 19665591; 16190655; 15866709; 11694506; 10092598 |
| NT09RC1680 | 4 | 21336932; 21298015; 21271477; 21254810; 21192484; 21180705; 21177917; 21115796; 21106176; 21088182 |
| NT09RC1690 | 6 | 21347417; 21315607; 21287394; 21274469; 21263241; 21249131; 21238786; 21229971; 21229606 |
| NT09RC1732 | 2 | 9371838; 17305534; 14745177; 11574544; 10767350; 10209263; 9917370; 9285799; 9177788; 9027492 |
| NT09RC1756 | 12 | 21385762; 21384200; 21383310; 21383158; 21382889; 21381190; 21379366; 21376345; 21368137; 21364982 |
| NT09RC1760 | 1 | 20601515; 20420455; 11429604; 20080659; 19807158; 19799945; 19603180; 19583445; 19378003; 19303906 |
| NT09RC1811 | 3 | 21277385; 21253654; 21244006; 21240549; 21162542; 21072227; 21040619; 20652740; 20645127; 20561595 |
| NT09RC1841 | 6 | 21113978; 20876678; 20537055; 20299102; 20185210; 20116104; 20056120; 20043919; 19941869 |
| NT09RC1851 | 1 | 20364396; 20028400; 19653643; 19382143; 19362563; 19041906; 18997402; 18763167; 18379776; 12389038 |
| NT09RC1890 | 8 | 20387788; 12107140; 17636390; 17279449; 17002391; 16849108; 15772073; 15264822; 14505375; 3609328 |
| NT09RC1970 | 6 | 21170359; 20581116; 20465561; 20138014; 9363943; 15065656; 16780573; 9619627; 12071693; 15201400 |
| NT09RC1977 | 9 | 20953507; 20702701; 20132453; 20044027; 8314797; 12077222; 19656201; 19628254; 18426892; 17325045 |
| NT09RC20875 | 2 | 20815377; 20498089; 19477904; 17121859; 17442677; 17372774; 15379587 |
| NT09RC2118 | 5 | 21378138; 21378135; 21360114; 21356200; 21335496; 21334970; 21274656; 21256798; 21228466; 21068774 |
| NT09RC2139 | 18 | 21356312; 21345803; 21344312; 21330335; 21305608; 21303518; 21299644; 21170890; 21273509; 21263029 |
| NT09RC22094 | 8 | 20348539; 19348575; 17064369; 14669923; 14628055; 12027885; 11876426; 11472504; 10878137; 16659565 |
| NT09RC2236 | 18 | 21359601; 21356379; 21347376; 21327521; 21306300 |
| NT09RC2378 | 13 | 18658123; 19945860; 19903445; 19446023; 18205605; 17150214; 16890206; 14750525; 12883871; 10430880 |
| NT09RC2380 | 6 | 21279410; 21178304; 20600108; 20080104; 18157161; 17483455; 11340628; 19414020; 19303843; 19205349 |
| NT09RC2390 | 4 | 21354424; 21279237; 21115710; 20961683; 20680263; 20600954; 20386942; 20356038; 19361444; 19361444 |
| NT09RC2395 | 2 | 21383008; 21360077; 21334730; 21327641; 21241821; 21241596; 21183220; 21090799; 21034764; 20978940 |
| NT09RC2406 | 18 | 20881245; 19429920; 19102629; 18628769; 12622816; 11039922; 17713961; 8096630; 8849412; 11045621 |
| NT09RC2419 | 12 | 21185091; 19329603; 18501515; 9508319; 18478957; 18429106; 18318009; 17979195; 17309236; 17123646 |
| NT09RC2463 | 8 | 21206700; 21130106; 20851451; 20473585; 20378716; 20100551; 19491303; 19367690; 19360290; 15489192 |
| NT09RC2487 | 5 | 21236489; 21055403; 20607275; 20563878; 20450939; 20394743; 19941308; 19785954; 19589348; 18614654 |
| NT09RC2504 | 4 | 21239579; 20573715; 20525833; 20516200; 19788546; 19788545; 19788546; 19854907; 19243443; 11522360 |
| NT09RC2518 | 13 | 20392698; 17215866; 11118225; 16257911; 10338213; 12777815; 10997488; 10518301; 1435261; 7715456 |
| NT09RC2605 | 12 | 21309470; 21292975; 21281954; 21255722; 21255181; 21248121; 21245143; 21212461; 21209222; 21185288 |
| NT09RC2613 | 4 | 20208433; 12697329; 11179649; 10972833; 10972808; 1715856; 165169; 8825779; 7682277; 409404 |
| NT09RC2665 | 12 | 21272927; 21259244; 21245344; 21178486; 21148036; 21106820; 21056976; 21048113; 20926588; 20919742 |
| NT09RC2678 | 8 | 21209092; 20516622; 20453144; 20375021; 20364333; 18086808; 18469270; 18941301; 18845259; 9398220 |
| NT09RC2679 | 8 | 20375021; 19299230; 18941301; 16820168; 16450403; 16157495; 16051266; 15609340; 1758883; 15355973 |
| NT09RC2724 | 12 | 20462657; 19759340; 19679160; 18478320; 1472493; 16777845; 14697270; 12090464; 11829493 |
| NT09RC27744 | 8 | 15581582; 17451747; 15467207; 14982630; 3521491; 4269305; 13842941 |
| NT09RC2798 | 6 | 20467813; 16151207; 15960265; 15925473; 15519045; 11272838 |
| NT09RC2880 | 6 | 21365542; 20811460; 20451470; 20299287; 18923071; 17965729; 18588880; 15965237; 18419580; 12893777 |
| NT09RC2952 | 9 | 21116859; 20842364; 20401696; 20221629; 20013057; 19645721; 8624411; 19520763; 19459638; 18757491 |
| NT09RC29576 | 2 | 16661655; 15153108; 15051720; 10737137; 10201094; 3042779 |
| NT09RC3033 | 12 | 20640780; 19880425; 19550122; 14976264; 19149172; 16434401; 18346003; 769835; 17662691; 17950620 |
| NT09RC3072 | 12 | 21394548; 21393179; 21392598; 21392585; 21391917; 21391832; 21390281; 21389182; 21388873; 21388714 |
| NT09RC30754 | 8 | 21321400; 21301089; 21265813; 21265800; 21037533; 21071338; 21044684; 21030387; 20973526; 20825165 |
| NT09RC3238 | 5 | 20971913; 20822113; 20624911; 20075614; 19714575; 8157625; 18771922; 8200973; 18576946; 18514484 |
| NT09RC3252 | 12 | 12713490; 17242511; 15994304; 15893768; 15713475; 15678420; 15375159; 12183436; 12470958; 12437101 |
| NT09RC3293 | 12 | 21378058; 21338918; 21338912; 21335977; 21326941; 21242965; 21231916; 21151970; 21109561; 20953191 |
| NT09RC3308 | 8 | 21378161; 21123945; 21108932; 21106925; 21092215; 21081500; 21068181; 20826956; 20736318; 20421289 |
| NT09RC3320 | 12 | 19711169; 19641506; 19303503; 18655058; 18586033; 18220151; 18211368; 17937401; 17052711; 16569292 |
| NT09RC3328 | 11 | 21156198; 20197135; 19879290; 17251179; 19416360; 16822325; 17028591; 8574395; 10837455; 15268934 |
| NT09RC3344 | 9 | 18680460; 17134979; 15814455; 15188046; 2154743; 12196011; 11173475; 8399222; 1526981; 3191103 |
| NT09RC3345 | 8 | 19631611; 13764136; 19594710; 10646606; 19408028; 17659996; 15888683; 17134979; 17009333; 16765624 |
| NT09RC3372 | 1 | 20488699; 18715230; 18569340; 17948003; 15436452; 17632581; 17481905; 16103022; 14631094; 12669100 |
| NT09RC3435 | 6 | 21393627; 21391900; 21366595; 21363965; 21360615; 21359151; 21339608; 21306445; 21299643; 21295952 |
| NT09RC3456 | 8 | 21253719; 21205211; 21138563; 21132286; 20499043; 20155483; 20054114; 19923736; 19744161; 19690365 |
| NT09RC3618 | 12 | 21377942; 21334867; 21105156; 21056541; 21040421; 21035558; 20941509; 20740612; 20694970; 20673093 |
| NT09RC3663 | 9 | 20696151; 20450981; 20233940; 19887598; 19500568; 19147725; 19109000; 19085751; 18220582; 17994458 |
| NT09RC3693 | 1 | 21084312; 20305003; 20305002; 20140469; 19877050; 19761441; 12432101; 18621388; 18300225; 18272376 |
| NT09RC3870 | 2 | 20965527; 19874540; 18287016; 18504589; 11606551; 3327686; 17220201; 18006965; 17651368; 8819329 |
| NT09RC3904 | 1 | 16439663; 17316682; 14675542; 7763371; 16240442; 12554952; 9818083; 9525895; 9418242; 9043106 |
| NT09RC3935 | 8 | 20979355; 18454933; 10319462; 16973619; 16850995; 16844076; 15353043; 12948635; 12615348; 16668720 |
| NT09RC3977 | 12 | 20230833; 19526727; 19159700; 18948221; 17971396; 12437884; 16547004; 16388577; 10479292; 12729748 |
| NT09RC4028 | 8 | 20947019; 19762827; 1905927; 18272421; 19306334; 18680460; 8554526; 18297087; 17134979; 15814455 |
| NT09RC4041 | 13 | 10937989; 12787353; 4620021; 7529559; 2037044; 173425; 2665813; 2653827; 6209544; 6750359 |
| NT09RC4111 | 5 | 21185614; 20703317; 20624911; 20418432; 20221731; 19843229; 10872445; 19138232; 19673421; 19481094 |
| NT09RC4137 | 18 | 19054082; 15215502; 12851073; 12427765; 11559732; 11539400; 340461 |
| NT09RC4175 | 8 | 20870944; 20647553; 20628058; 20529839; 20492473; 20456758; 20451919; 18813375; 20121198; 20121197 |
| NT09RC4176 | 8 | 15386115; 9804883; 14702404; 6751257; 10026281; 271968; 13895406; 8599534; 8525056; 7473063 |
| NT09RC4177 | 8 | 20814302; 12521268; 15386115; 9804883; 11297752; 8599534; 1850088; 2503674; 772161; 881736 |
| NT09RC4185 | 6 | 21211725; 20943161; 20864038; 20720002; 20705653; 11972039; 20605454; 20600630; 20562521; 20534440 |
| NT09RC41871 | 2 | 21288652; 21247799; 20649840; 20484298; 20382023; 19197387; 19507290; 19490098; 19167902; 1754394 |
| NT09RC4253 | 12 | 21390227; 21343300; 21214190; 21187331; 21172299; 21147340; 21116996; 21087640; 20947019; 20943794 |
| NT09RC4316 | 15 | 21389131; 21389121; 21373931; 21362163; 21338579; 21334415; 21292297; 21285160; 21170869 |
| NT09RC4382 | 3 | 14221104; 17293176; 11672729; 11171193; 10224056; 9349281; 8286443 |
| NT09RC4423 | 12 | 21349975; 21239585; 21185288; 18942856; 18834129; 18767150; 18682379; 11123699; 17426034; 12896998 |
| NT09RC4464 | 6 | 21301105; 21216167; 20844218; 20833633; 20709846; 347446; 16740948; 19620365; 19567585; 19556347 |
| NT09RC4572 | 3 | 20828539; 20685834; 11581264; 19103164; 7741826; 18323637; 18295483; 11300872; 16420487; 15788389 |
| NT09RC4637 | 6 | 21365542; 21361871; 21350489; 21274582; 21151660; 21146520; 21138837; 21097613; 21070963; 21035377 |
| NT09RC4647 | 17 | 21183650; 20870727; 20864038; 20730136; 20724389; 20733069; 20695995; 20686083; 20606261; 20600630 |
| NT09RC4650 | 13 | 20188109; 10937989; 7677746; 1690811; 16076220; 15680962; 10890005; 10368156; 10937989; 12473202 |
| NT09RC4662 | 13 | 9757107; 10937989; 8174557; 2664451; 6178424 |
| NT09RC4665 | 13 | 10561594; 7916699; 1772592; 2438658; 3926498 |
| NT09RC4674 | 13 | 18757750; 19154332; 16762565; 10596806; 8223574; 1499563; 2461735 |
| NT09RC4692 | 3 | 21231969; 20219465; 16756317; 18314963; 10506203; 17959596; 17163967; 17154541; 16137685; 16202390 |
| NT09RC4693 | 5 | 21365232; 21206044; 21138746; 21046332; 20965680; 20875081; 20678117; 20635418; 20473958; 20456044 |
| NT09RC4704 | 8 | 20613792; 19825618; 11488591; 10394014; 15150173; 12234497; 8563639; 11167002; 10856674; 1830580 |
| NT09RC4765 | 8 | 7603409; 7925445; 2981635; 6094487; 127785; 6411649; 6792201 |
| NT09RC4926 | 8 | 19237441; 19122206; 18307973; 18293930; 17635583; 17577575; 16846214; 15853806; 11514662; 687632 |
| NT09RC49315 | 2 | 20681987; 18284573; 18258600; 17469805; 17452319; 12033984; 16455656; 15952888; 15561708; 15480755 |
| NT09RC4991 | 13 | 20399793; 9169555; 15989950; 10805779; 10747797; 8123703; 1764524; 6360687; 7049235; 6125208 |
| NT09RC5006 | 3 | 20861142; 20729083; 20392080; 20075623; 19899805; 19782148; 19656295; 19298367; 8293964; 18672863 |
| NT09RC5012 | 14 | 4077987; 10074353; 16985054; 15615517; 10701848; 14756554; 14756553; 12501179; 10985775; 10479275 |
| NT09RC5069 | 6 | 15199175; 11395472; 15699185; 1102934; 9837717; 9383194; 9000619; 8921897 |
| NT09RC5072 | 15 | 15979091; 149110; 9345313; 7504905; 1686293 |
| NT09RC5085 | 8 | 21371432; 21315380; 21248490; 21185308; 21136597; 21122807; 21115336; 21104949; 21093100; 20841500 |
| NT11FT0233 | 6 | 20972214; 20337945; 20329707; 20222445; 20188667; 19749191; 3300806; 17210572; 12857762; 16767502 |
| NT11FT0456 | 3 | 21371235; 17804791; 12791140; 7910938; 16983196 |
| NT11FT0535 | 8 | 12524212; 14758542; 12421312; 9914305; 9524269; 1309972; 7715602; 7715602; 7715601; 8231805 |
| NT11FT0608 | 9 | 17385315; 16739479; 4623694; 15241633; 10937442; 10848999; 9744093; 9559566; 9231425; 8318516 |
| NT11FT0654 | 3 | 20824106; 20638049; 20510395; 20453897; 8862584; 20075111; 19435408; 16980456; 18700154; 18673069 |
| NT11FT0738 | 8 | 21062915; 20823107; 20026072; 14504387; 18542081; 18784658; 19170109; 19245216; 18550570; 18095160 |
| NT11FT0746 | 8 | 20424179; 20399199; 20100622; 19767760; 19596433; 18502195; 18400335; 18374190; 17910598; 17762829 |
| NT11FT0773 | 3 | 18258263; 16040347; 18499663; 18325534; 16829524; 16794327; 15820665; 15552059; 15226299; 15161861 |
| NT11FT0866 | 11 | 21371235; 21311024; 21264306; 21248229; 21212019; 21183669; 21083928; 12900386; 4106489; 20487265 |
| NT11FT0983 | 1 | 20081005; 19542000; 18784913; 17624809; 16795146; 16453101; 15685598; 10420995; 2407655; 11243831 |
| NT11FT1045 | 8 | 20924198; 18467858; 18188555; 18060402; 17587673; 10867230; 12149117; 8617755; 11934292; 11931561 |
| NT11FT1180 | 18 | 21357619; 21245529; 21193613; 21167158; 21091510; 21075841; 21068385; 20944229; 20929442; 20890837 |
| NT11FT1275 | 18 | 20056707; 17096120; 16647083; 16505971; 11640984; 11160802; 10937490; 10537210; 9622354; 9484231 |
| NT11FT16504 | 3 | 21393450; 21390322; 21386968; 21378194; 21376539; 21365198; 21364896; 21359316; 21342097; 21338419 |
| NT11FT1988 | 8 | 18651753; 10952301; 15282189; 15126303; 12914915; 12678433; 11295131; 13382877; 8797851; 8536688 |
| NT11FT2057 | 8 | 19585612; 15523170; 19428809; 19062176; 17614191; 429321; 17267261; 15663956; 10806340; 7768362 |
| NT11FT2099 | 15 | 20718459; 18724706; 17709748; 18313075; 17224607; 9493270; 15728912; 10331874; 15588829; 15447145 |
| NT19SS0116 | 2 | 16367969; 15709772; 12571226; 11891227; 8514783 |
| NT19SS0441 | 6 | 10829079; 10048034; 16407074; 10716434; 13129609; 12818199; 12119115; 11545584; 9642195; 10744667 |
| NT19SS0482 | 4 | 21899456; 11454207; 21807981; 15939505; 21741112; 21709092; 21709077; 21660655; 21571624; 21563953 |
| NT19SS0497 | 18 | 21281627; 21263124; 21187111; 21094695; 21079776; 21041989; 20971155; 20878295; 20878240; 20805568 |
| NT19SS0660 | 8 | 21324604; 19519368; 18803552; 17897734; 17576516; 16534744; 16268782; 15883004; 15628446; 15555758 |
| NT19SS0769 | 6 | 21349997; 21348848; 21345950; 21344236; 21343400; 21342128; 21335604; 21330363; 21317870; 21304054 |
| NT19SS0951 | 8 | 21283685; 21270770; 21269365; 21234284; 21138736; 21125339; 21045145; 21033426; 21041650; 20919991 |
| NT19SS1020 | 9 | 21370994; 21357626; 21318901; 21301088; 21291275; 21239625; 21235239; 21185310; 21127271; 21071236 |
| NT19SS1205 | 18 | 21393450; 21391724; 21354102; 21339594; 21339577; 21330430; 21321143; 21317944; 21315184; 21296967 |
| NT19SS12843 | 8 | 21372322; 21350246; 21149701; 21135251; 21040473; 20921990; 20842695; 20835928; 20727186; 20709735 |
| NT19SS1740 | 8 | 1849603; 19904424; 9233812; 12125824; 16524901; 17903205; 17639348; 16840531; 14592709; 12752441 |
| NT19SS1840 | 18 | 20932170; 18280571; 18162938; 11401986; 17029153; 15824422; 15496397; 12518388; 10553708; 6146721 |
| NT19SS1969 | 15 | 21375693; 21371473; 21030591; 20974046; 20852360; 20370354; 20208509; 20171070; 20110498; 18725941 |
| NT19SS2018 | 15 | 21097634; 20545849; 9600888; 10676961; 16672602; 17628141; 20076637; 16866347; 10487748; 9860942 |
| NT19SS2371 | 6 | 21291520; 21234494; 21193549; 21187428; 21115708; 21094162; 21092102; 21072428; 21051524 |
| NT19SS2386 | 6 | 21187477; 21155533; 21127267; 21030352; 20974932; 20964789; 20963646; 20952393; 20929586; 20919954 |
| NT19SS2612 | 4 | 12867445; 1740117; 17259614; 8757282; 10480935; 10464182; 3008088; 9917389; 8878043; 8736534 |
| NT20NM01761 | 2 | 20484298; 19197387; 19507290; 19490098; 19167902; 1754394; 15215345; 17611796; 17295612; 17132104 |
| NT20NM0338 | 8 | 21173259; 20568458; 20381627; 19453914; 18704577; 17920788; 17487300; 17333279; 16545948; 16385111 |
| NT20NM0459 | 8 | 21241169; 21194188; 21110519; 20621065; 20615876; 20544959; 20143183; 19200363; 19906178; 19965429 |
| NT20NM0607 | 12 | 20036319; 18967155; 4475698; 12208161; 11001871; 10850619; 9787900; 9667026; 9464857; 9134732 |
| NT20NM0735 | 17 | 21038358; 4059052; 18024596; 18282007; 17922659; 17651890; 17561113; 15784265; 17306298; 16858551 |
| NT20NM0870 | 6 | 21385054; 21367748; 21366357; 21362551; 21362023; 21343428; 21339507; 21338660; 21327072; 21321201 |
| NT20NM0902 | 17 | 20923236; 19833764; 7501460; 19111618; 18995833; 10837198; 18070067; 3052291; 10348869; 16728456 |
| NT20NM1225 | 3 | 19364381; 8377180; 12944424; 12468539; 12055003; 11802720; 11718936; 11683359; 11576872; 10493853 |
| NT20NM1433 | 15 | 21068324; 20935164; 20930109; 20890837; 20699402; 20516589; 20204475; 20194531; 19850005; 18076326 |
| NT20NM1472 | 3 | 21070855; 10542091; 16475802; 7551037; 15586827; 15366937; 15358542; 15133104; 11790124; 11139297 |
| NT20NM1549 | 3 | 14769872; 15518577; 14529267; 8987972; 8961949; 8672489; 8672475 |
| NT20NM1792 | 18 | 21075893; 20421383; 20364433; 20155302; 19682417; 19655425; 10692151; 18822771; 17543335; 17192261 |
| NT20NM1806 | 11 | 10700280; 17091335; 10722135; 10491262; 12726770; 11200543; 1320187 |
| NT20NM2002 | 3 | 17517879; 10504382; 14600228; 12435504; 11836124; 10496915; 10908566; 9061365; 5804498; 8781979 |
| NT20NM2075 | 18 | 20926701; 20618438; 20467255; 20161024; 20097166; 19789413; 19716795; 18634255; 9479046; 17151919 |
| NT20NM2089 | 3 | 21149452; 20036252; 19114107; 112060; 12787347; 12787347; 11454209 |
| NT20NM2256 | 12 | 14622420; 8837412; 9668097; 9623911; 9224881; 8820654; 8817497; 7565106; 8057924; 8340405 |
| NTL01EC00014 | 12 | 21386879; 21382374; 21379326; 21373826; 21358815; 21357443; 21356360; 21351069; 21340359; 21335977 |
| NTL01EC00015 | 12 | 21378058; 21351069; 21338918; 21338912; 21335977; 21326941; 21324738; 21309513; 21295415; 21281637 |
| NTL01EC00021 | 11 | 15387827; 15225314; 9829919; 9126837; 7625275; 8021940; 1334530; 13278318; 2160405; 2553980 |
| NTL01EC00022 | 11 | 15387827; 15225314; 9829919; 9126837; 7625275; 2826132; 8021940; 1334530; 13278318; 1965605 |
| NTL01EC00029 | 15 | 21367979; 21338423; 21329802; 21176126; 21150259; 21056117; 21102605; 20950336; 20851888; 20839223 |
| NTL01EC00036 | 8 | 18077198; 16297647; 12549051; 11730586; 11551212; 11409545; 11356164; 11257539; 11244185; 10802246 |
| NTL01EC00043 | 8 | 21393444; 21374779; 21366490; 21362687; 21359175; 21347544; 21333638; 21327819; 21321959; 21314018 |
| NTL01EC00068 | 18 | 21125117; 20873853; 20471400; 18177053; 12182833; 2407294; 2676709 |
| NTL01EC00071 | 1 | 20938981; 20663849; 20170198; 17369439; 19194004; 794063; 15522288; 7789819; 8173074 |
| NTL01EC00083 | 4 | 21394897; 21394325; 21394097; 21394085; 21394081; 21393427; 21392641; 21392508; 21392495; 21392187 |
| NTL01EC00085 | 3 | 19400768; 18036201; 11090285; 10566865; 7925310; 11722566; 11398928; 11124264; 10873532; 4030693 |
| NTL01EC00095 | 4 | 21394936; 21394897; 21394776; 21394431; 21394325; 21394288; 21394108; 21394097; 21394085; 21394081 |
| NTL01EC001092 | 2 | 21206019; 21054826; 20206212; 20047307; 20047306; 19941308; 19399913; 17054470; 19165623; 18381895 |
| NTL01EC001140 | 8 | 17927566; 17715948; 12634339; 17329260; 10486147; 17157320; 17042496; 16472748; 16084384; 15378749 |
| NTL01EC001160 | 8 | 21335456; 21168410; 20960712; 20848047; 20833784; 20815787; 20739172; 20652410; 20209439; 20167543 |
| NTL01EC00121 | 5 | 21330782; 21318891; 21318875; 21318872; 21248490; 21219895; 21208503; 21187074; 21152090; 21097708 |
| NTL01EC00196 | 3 | 19212404; 18600776; 18309357; 18257680; 10498711; 17849711; 17379715; 15469511; 12084982; 11758943 |
| NTL01EC00236 | 1 | 19850488; 16439663; 18054243; 14532061; 17571216; 16958849; 10072398; 14705032; 12693104; 3540312 |
| NTL01EC00306 | 18 | 21233792; 21145918; 20923416; 20888396; 20637607; 20601463; 20599757; 20576035; 20490865; 20394050 |
| NTL01EC00328 | 18 | 20691393; 20398062; 20042597; 9074648; 19470736; 2614483; 19270429; 19240178; 19223557; 19215240 |
| NTL01EC003347 | 8 | 19445173; 18762429; 18461320; 18309280; 17497738; 17333171; 16960736; 16860571; 16707333; 15950163 |
| NTL01EC00335 | 18 | 20837701; 15209157; 10390363; 12578349; 12395284; 11969416; 10926681; 10769142; 10758173; 10727615 |
| NTL01EC003367 | 8 | 21913220; 21911974; 21910767; 21909130; 21909125; 21908788; 21908665; 21905631; 21904881; 21903747 |
| NTL01EC003407 | 8 | 20013980; 19744988; 12514037; 17051653; 15672171; 15491145; 8246842; 1791759; 9056848; 3038334 |
| NTL01EC003887 | 8 | 21394791; 21393472; 21393368; 21393332; 21390073; 21389271; 21389113; 21389019; 21386754; 21385376 |
| NTL01EC00389 | 6 | 21325134; 17924399; 16632468; 1454527; 9004227; 8390577; 1768456 |
| NTL01EC00397 | 13 | 20354154; 20129918; 19925456; 19894214; 19874048; 19746363; 19627989; 7578154; 19199329; 11001804 |
| NTL01EC00403 | 18 | 21335481; 21266914; 21251217; 21054262; 21044214; 20977457; 20946858; 20889844; 20884616; 20870805 |
| NTL01EC00407 | 2 | 18671734; 11375002; 2206482; 7473709; 7502581; 8055941; 4346248; 6816587 |
| NTL01EC00416 | 2 | 15271986; 11513588; 8610181; 7519593; 13278318; 2194094 |
| NTL01EC00425 | 18 | 21169496; 20655466; 20117966; 18333608; 19109493; 17517839; 18511069; 17900178; 10910347; 17005991 |
| NTL01EC00430 | 12 | 16682229; 10485712; 9428517; 8885261; 7900997; 8027082; 8027081; 8226769; 8407953 |
| NTL01EC00463 | 6 | 21393994; 21393049; 21388532; 21385444; 21384097; 21383964; 21383502; 21383164; 21383063; 21378394 |
| NTL01EC00465 | 14 | 21372131; 21352825; 21344481; 21265739; 21244427; 21232545; 21209212; 21209207; 21217676; 21224887 |
| NTL01EC00466 | 2 | 21158025; 12629548; 10383398; 17196160; 16870250; 1647023; 15660829; 15236569; 15123683; 12554938 |
| NTL01EC00471 | 14 | 15808855; 16349109; 8682799; 11802543; 11429465; 10331872; 8798549; 8288106; 1103973; 6265322 |
| NTL01EC00517 | 13 | 2426258; 18425141; 16385054; 17512006; 16087664; 15886196; 15790858; 15526152; 15322138; 9810464 |
| NTL01EC00538 | 11 | 17049932; 9360547; 9003531; 8355271; 6984192; 6241582; 6455332 |
| NTL01EC00550 | 11 | 21358801; 21320601; 21220301; 21206037; 21129201; 20963614; 20890845; 20684044; 20650282; 20620152 |
| NTL01EC005835 | 2 | 20079748; 16042594; 14982443; 11779629; 9795253; 8921902; 8549818; 2139795; 2154945; 3040679 |
| NTL01EC00620 | 2 | 21338421; 21338420; 21209092; 21177730; 20882995; 20870766; 20862391; 20718955; 20687343; 20508247 |
| NTL01EC00622 | 3 | 17938168; 16965759; 15051164; 10094630; 388439; 1833192; 1833192 |
| NTL01EC00642 | 18 | 11426296; 11199484; 10976971; 10692470; 10630609; 10461890; 10411993; 10366190; 9523822; 9463476 |
| NTL01EC00658 | 5 | 21342462; 21309707; 21295693; 21292975; 21278273; 21240541; 21240259; 21223562; 21200003; 21200004 |
| NTL01EC00670 | 6 | 15326179; 15225322; 12527112; 11254141; 8051709; 8011018; 822174; 6263488 |
| NTL01EC00676 | 5 | 15199544; 12115530; 11961394; 10444369; 10409134; 9688940; 9566840; 8149484; 3722206 |
| NTL01EC00684 | 11 | 20563849; 20102440; 19968874; 18440654; 17949347; 17822785; 7508433; 17222178; 17180248; 3335402 |
| NTL01EC00697 | 6 | 14522981; 12819227; 12519758; 11684456; 10079077; 8960131; 7559510; 8043588; 7516707; 8110759 |
| NTL01EC007052 | 8 | 11803023; 11692162; 11004459; 10216163; 9732445; 9521736; 9395469; 9211943; 1731062; 8626739 |
| NTL01EC007357 | 8 | 21151867; 21078123; 20717852; 20607749; 20587332; 15602552; 20519568; 20439767; 20353836; 20299676 |
| NTL01EC00786 | 18 | 17419840; 17893916; 17105494; 16516348; 15379896; 14968431; 14754920; 14668330; 12956537; 12933350 |
| NTL01EC00788 | 15 | 20656783; 20638427; 20424261; 17041587; 15733837; 18067544; 8626809; 18460915; 16264189; 17464064 |
| NTL01EC00815 | 3 | 21183069; 21074048; 20920790; 20620870; 20608745; 20497333; 20128627; 20061535; 19935678; 19646181 |
| NTL01EC00818 | 18 | 20971900; 20688487; 20606071; 20540528; 18781920; 19425588; 19166984; 19136595; 18781920; 18644451 |
| NTL01EC00851 | 18 | 21359105; 21334314; 21315850; 21307072; 21271497; 21264240; 21244858; 21244837; 21237499; 21228336 |
| NTL01EC00858 | 12 | 20510150; 19650643; 17491602; 16452686; 8242736; 12738681; 12032330; 9890793; 11278349; 11250194 |
| NTL01EC00859 | 13 | 20132820; 18951960; 18758445; 16673819; 15315759; 3313277; 11084369; 10644698; 10659855; 6383865 |
| NTL01EC00866 | 12 | 8407802; 17498646; 11994151; 10944190; 10027988 |
| NTL01EC00933 | 4 | 12730326; 7890422; 11169116; 10732276; 6300834; 9162108; 2109835; 1632986; 1367178; 3029041 |
| NTL01EC01043 | 3 | 21372321; 21363968; 21162678; 21126515; 21122159; 21118484; 21118278; 21091512; 21091499; 21076396 |
| NTL01EC01044 | 3 | 20304988; 20233930; 20199595; 18198339; 19130906; 19016841; 18757812; 10097157; 10869072; 17537210 |
| NTL01EC01048 | 3 | 17537210; 16677309; 11405627; 10564473; 10478459; 2407720; 8200538 |
| NTL01EC01049 | 3 | 20979349; 20639318; 20586476; 20581225; 10785634; 20199595; 20132451; 20026337; 19919668; 18331472 |
| NTL01EC01065 | 9 | 20418430; 20222707; 20176020; 20057061; 19136596; 19797355; 19777301; 19555075; 19550039; 19118366 |
| NTL01EC01071 | 14 | 21212540; 21128666; 21079776; 20951473; 20860090; 20833052; 20593468; 20516559; 6254974; 20497505 |
| NTL01EC01082 | 8 | 21390523; 21364629; 21358763; 21303534; 21167537; 21114358; 21081762; 21040753; 20933494; 20923772 |
| NTL01EC01112 | 11 | 21029433; 21029432; 20729353; 20545861; 19215770; 18500629; 10455110; 12626685; 17371501; 16807240 |
| NTL01EC01131 | 11 | 21255110; 21212362; 21044741; 20562305; 20542150; 20395367; 20374940; 20104556; 19889099; 10970762 |
| NTL01EC01149 | 4 | 18801989; 18275156; 9751889; 15948963; 9219999; 10411726; 8930908 |
| NTL01EC01156 | 6 | 21302943; 21300836; 21300638; 21212055; 21183573; 21168323; 21118802; 21072368; 20975940; 20936072 |
| NTL01EC01157 | 6 | 21168323; 20975940; 20727918; 15020458; 9214645; 20441441; 20435157; 20345942; 20178806; 19892776 |
| NTL01EC01163 | 8 | 19748470; 19746945; 19328247; 19155267; 19060308; 18618696; 3167024; 18023020; 17944363; 17937657 |
| NTL01EC011702 | 4 | 15803655; 16944251; 16410039; 16232965; 10816581; 9356134; 149110; 3317413; 8541310; 8081287 |
| NTL01EC01188 | 3 | 21042030; 20825995; 20577996; 20533322; 20406700; 20363943; 19891460; 19447118; 19399763; 19228070 |
| NTL01EC01220 | 9 | 19898564; 9614098; 9527846; 9442089; 8385010; 2172219; 6384177; 2982784 |
| NTL01EC01223 | 18 | 21371810; 21364120; 21360612; 21344882; 21336346; 21325051; 21321316; 21317014; 21303958; 21302947 |
| NTL01EC01236 | 1 | 20384694; 20061467; 18280161; 16839776; 10494619; 15262409; 15023340; 12855807; 10860976; 11050101 |
| NTL01EC01252 | 14 | 21187411; 20929557; 20605980; 20574830; 20498911; 20473969; 20452222; 20441167; 20424759; 20369850 |
| NTL01EC01275 | 11 | 21259006; 20595257; 19124575; 7921245; 11266368; 18833010; 10393967; 16709570; 16116291; 10216859 |
| NTL01EC01322 | 11 | 21255333; 16677300; 12886937; 12559570; 1372677; 8246844; 6313948; 6460911; 362150; 6449653 |
| NTL01EC01324 | 11 | 20545868; 15770420; 17768233; 16643978; 15476873; 12429513; 11985726; 2327075; 8335629; 8012757 |
| NTL01EC01357 | 8 | 20298739; 20012150; 20012114; 19789505; 19588076; 19266512; 11303044 |
| NTL01EC01387 | 8 | 11006331; 18382362; 17259241; 15985219; 10997131; 10211684; 7944356; 7765846 |
| NTL01EC01388 | 8 | 10323238; 11461143; 9153454; 8564964; 1934112; 450128; 6887131 |
| NTL01EC01450 | 8 | 18781696; 17447164; 11451371; 10844979; 10425713; 8342959; 2347425; 39599; 3741415; 2868716 |
| NTL01EC01478 | 3 | 21193612; 21191097; 21187288; 21115727; 21113133; 21097630; 21097627; 21076477; 21075923; 21070416 |
| NTL01EC01509 | 12 | 16221788; 17597310; 17343204; 17081790; 17051737; 16786999; 10825662; 14609329; 12901857; 12161196 |
| NTL01EC015280 | 4 | 21367479; 21362495; 21359956; 21357508; 21314836; 21298478; 21286923; 21282328; 21279407; 21262280 |
| NTL01EC01533 | 4 | 20954258; 20620241; 20549620; 20410104; 16651620; 19330811; 20201394; 19080332; 18925684; 18522493 |
| NTL01EC01545 | 4 | 21394108; 21394081; 21393427; 21393231; 21391788; 21391601; 21390206; 21390183; 21390048; 21389327 |
| NTL01EC01580 | 6 | 21394759; 21394103; 21394101; 21393861; 21393642; 21393636; 21393603; 21393127; 21393072; 21392397 |
| NTL01EC015877 | 8 | 21277764; 20969898; 20932554; 20840586; 20812981; 20723241; 20706744; 20634491; 20615998; 20607443 |
| NTL01EC01603 | 6 | 21384253; 21354178; 21353648; 21345667; 21310716; 21291520; 21278688; 21256960; 21241689; 21225639 |
| NTL01EC01607 | 13 | 20956970; 20839808; 20690656; 20676842; 20598274; 12446813; 20340150; 20204861; 16819861; 17993461 |
| NTL01EC01616 | 4 | 21239495; 21035278; 20416286; 10832909; 10497178; 19103168; 16814528; 18760622; 18755596; 18004558 |
| NTL01EC01691 | 8 | 21320626; 21270901; 20679207; 20200205; 20080177; 18808312; 8477732; 7903041; 2093896; 16087376 |
| NTL01EC01735 | 8 | 21234094; 21084304; 21083476; 20592726; 20093801; 17189693; 18996338; 16713649; 17927282; 17536015 |
| NTL01EC01772 | 17 | 20086050; 11729082; 19458942; 11201747; 12054892; 18268842; 15066284; 2156629; 15806104; 8918471 |
| NTL01EC01828 | 6 | 20670908; 18782564; 16436047; 9822387; 10359700; 10600744; 9922256; 1427081; 15078220; 12028381 |
| NTL01EC01831 | 6 | 21145792; 21085632; 20935048; 20667794; 20634321; 20603075; 20375162; 20304994; 17364684; 17965729 |
| NTL01EC01851 | 4 | 3323813; 12591864; 12022879; 11994152; 11092844; 7803815 |
| NTL01EC01867 | 18 | 17660519; 16604273; 11080142; 7582021; 8581399; 7715454; 8344909; 4899002; 2614377; 2445996 |
| NTL01EC01873 | 18 | 21047430; 20805568; 20495089; 20478358; 20439472; 20347012; 20336795; 20230873; 18826427; 20132520 |
| NTL01EC01903 | 3 | 21392573; 21372321; 21363968; 21191099; 21170308; 21162678; 21126515; 21122159; 21118484; 21118278 |
| NTL01EC01924 | 6 | 20541519; 19996303; 17951114; 98523; 17294256; 10504701; 16024043; 1722334; 15590328; 8532526 |
| NTL01EC01969 | 3 | 21161561; 11023960; 20194703; 18809265; 18723327; 2377622; 7872753; 11052419; 15155207; 17338318 |
| NTL01EC01988 | 8 | 11018134; 10960103; 12693814; 11293064; 10392540; 16663408; 9463796; 8879247; 4564719; 1537796 |
| NTL01EC019957 | 8 | 21335527; 21168771; 20850454; 20816491; 20678902; 20615386; 20237670; 20211750; 19887444; 19836401 |
| NTL01EC02037 | 17 | 18202674; 17884222; 15522865; 12354228; 8282725; 6544780 |
| NTL01EC020917 | 8 | 15913824; 15742147; 12871846; 9720023; 10817821; 8521952; 10425161; 10390821; 9721604; 9511746 |
| NTL01EC02109 | 18 | 20937906; 20599595; 20118243; 20058234; 10196117; 19961827; 19929274; 15801771; 19859921; 19766634 |
| NTL01EC02127 | 8 | 20679207; 20597606; 19889946; 17108241; 19379783; 18455501; 17609257; 16469539; 16453288; 16377227 |
| NTL01EC02152 | 8 | 20513359; 16831427; 15312975; 11955008; 9368004; 9063867 |
| NTL01EC02153 | 8 | 20188670; 10487920; 19336478; 2033048; 19021509; 17623665; 9293186; 9374473; 11967064; 12135473 |
| NTL01EC02159 | 12 | 20064972; 16233359; 1631111; 11335719; 8226679; 7635817; 1282354; 9030765; 3017638; 1317384 |
| NTL01EC02170 | 6 | 17493798; 15367703; 11023544; 8805338; 7692268; 2696875; 3047400; 3748047; 6533049 |
| NTL01EC02181 | 9 | 21377632; 21085119; 20971890; 20952531; 20885002; 20882882; 20862428; 20858440; 20843287; 20834181 |
| NTL01EC02189 | 6 | 21301105; 20383017; 347446; 20057069; 19923746; 17951987; 17116675; 17397985; 15572765; 17302148 |
| NTL01EC02220 | 2 | 21392992; 21391603; 21388965; 21385576; 21384820; 21381310; 21378135; 21373769; 21368225; 21366874 |
| NTL01EC022557 | 8 | 21253866; 21177827; 21122111; 21104132; 21086129; 21068339; 21060736; 21046341; 21044662; 20952579 |
| NTL01EC02264 | 18 | 20888343; 19308662; 18248418; 18022195; 17040912; 3403505; 16418175; 15843026; 15758229; 11916378 |
| NTL01EC02270 | 14 | 17686772; 17434429; 7683680; 14977588; 11330670; 10713115; 10593947; 10512621; 9914248; 7683680 |
| NTL01EC02271 | 4 | 15249060; 9106219; 7854125; 2714272; 2863218; 3543211; 781293 |
| NTL01EC02323 | 8 | 825075; 17507508; 2649478; 14755641; 12823810; 7326243 |
| NTL01EC02357 | 18 | 6234454; 15218545; 1593632; 1961752; 8805693; 1332978; 1706467 |
| NTL01EC02358 | 14 | 15808857; 15808857; 15784179; 10537218; 122568; 692401; 1706467; 8482076; 819302; 7007809 |
| NTL01EC02363 | 4 | 20603653; 20497503; 16420366; 10781575; 19659446; 11115884; 19447111; 11402200; 17328675; 10607659 |
| NTL01EC02519 | 12 | 21189343; 21135574; 21095572; 21075926; 12382110; 20566871; 20522495; 20419406; 20419402; 20417202 |
| NTL01EC02521 | 15 | 18619465; 17369078; 17007876; 17061571; 14700078; 12603732; 10792721; 10322172; 10027976; 9701805 |
| NTL01EC02522 | 15 | 1925561; 17061571; 2691330; 11442831; 11260465; 9159523; 10411727; 9159522 |
| NTL01EC02524 | 15 | 20802044; 10610760; 19300486; 19415331; 19400803; 2867991; 18682280; 18086212; 10384278; 3127379 |
| NTL01EC02561 | 11 | 21360615; 21154877; 20949106; 20943970; 20833633; 20829283; 20813883; 20711416; 12771385; 9158724 |
| NTL01EC02563 | 6 | 21388532; 21385444; 21383063; 21376743; 21376234; 21372041; 21368836; 21368455; 21363882; 21362621 |
| NTL01EC026457 | 8 | 19784641; 19058613; 18972399; 18856213; 18760354; 18726503; 15211509; 18483816; 18351334; 18242768 |
| NTL01EC026467 | 8 | 15211509; 1137083; 12177329; 11390381; 1945499; 3313728; 1619665; 7894055; 2287279; 3062173 |
| NTL01EC027447 | 8 | 20883732; 19608744; 10331874; 2237403; 19183274; 18488147; 18355019; 17173282; 15664934; 16987809 |
| NTL01EC02765 | 9 | 19383708; 18954360; 18641340; 18602442; 18424736; 17090541; 17041050; 17071755; 4040889; 16895402 |
| NTL01EC02829 | 3 | 21305254; 21173016; 21150130; 20922471; 20547235; 20435339; 20202930; 20070606; 20005186; 19925638 |
| NTL01EC02896 | 6 | 21178863; 21110984; 21063410; 21044965; 21044966; 20888338; 20848659; 20816984; 20725929; 20724227 |
| NTL01EC02900 | 5 | 20217058; 19399584; 19246319; 18477648; 18227970; 17510413; 17262025; 15734972; 15714464; 15510159 |
| NTL01EC02939 | 18 | 21080032; 20826777; 20737137; 20585060; 2254250; 20418143; 20335169; 20086155; 19696110; 19609963 |
| NTL01EC029667 | 8 | 21387538; 21345178; 21338600; 21317321; 21276044; 21273249; 21268903; 21117150; 21115656; 21092102 |
| NTL01EC02969 | 4 | 21390528; 21388961; 21377443; 21371926; 21365697; 21350490; 21347391; 21343394; 21342097; 21339594 |
| NTL01EC029834 | 8 | 18778276; 11571202; 101676; 15162198; 9770508; 1324388 |
| NTL01EC03000 | 6 | 21393386; 21393142; 21392374; 21390379; 21390258; 21390132; 21390131; 21389547; 21389352; 21389349 |
| NTL01EC03107 | 2 | 18482337; 17289662; 15341734; 3114239; 11007651; 16653243; 14228777; 1965313; 3123474; 7374502 |
| NTL01EC031187 | 8 | 18427742; 18310036; 18280752; 18158608; 15795232; 10330172; 10995226; 12668681; 11414203; 11063580 |
| NTL01EC03119 | 3 | 20637039; 20382772; 19782148; 17675432; 10991869; 18242192; 11118200; 17889830; 17827659; 17685588 |
| NTL01EC03132 | 15 | 21378190; 21054445; 20862323; 20678145; 20624215; 20363783; 20356457; 20067173; 12519953; 19942657 |
| NTL01EC03177 | 4 | 21362063; 21347262; 21296049; 21289288; 21255801; 21245391; 21234568; 21231508; 21224395; 21185188 |
| NTL01EC03186 | 9 | 19423627; 1355089; 15514159; 18557770; 17266990; 10716717; 16707089; 16276872 |
| NTL01EC03191 | 6 | 2988787; 19443546; 1851089; 10216857; 15223318; 14763986; 11133968; 1829453; 10610762; 2656703 |
| NTL01EC03196 | 18 | 21387000; 21378197; 21378181; 21376730; 21367437; 21361323; 21359316; 21357901; 21354311; 21352231 |
| NTL01EC03210 | 13 | 21288715; 21185180; 21146987; 20936344; 20859963; 20805355; 20713740; 20656778; 20615695; 20607487 |
| NTL01EC03258 | 12 | 21135574; 21075926; 20800706; 12382110; 20566871; 20522495; 20419406; 20419402; 20417202; 20207389 |
| NTL01EC03263 | 13 | 20855746; 19716785; 19716793; 19366171; 10528787; 19173642; 18951096; 18753147; 18213444; 993776 |
| NTL01EC03273 | 18 | 21393450; 21393229; 21392501; 21377460; 21364900; 21358125; 21343467; 21336592; 21329820; 21325441 |
| NTL01EC03291 | 2 | 19754882; 19267692; 19154787; 18680949; 18603790; 17460889; 16489629; 16289918; 15983414; 15522295 |
| NTL01EC03343 | 17 | 21367972; 21224389; 20399182; 2579395; 19478432; 18776333; 14757243; 10322435; 10673421; 10574971 |
| NTL01EC03346 | 8 | 19682247; 15328412; 11071771; 9524241; 9179845; 1372899; 2040302; 1400248; 1556120; 1372899 |
| NTL01EC03347 | 8 | 20000779; 11709175; 9393850; 2540407; 8397789; 9524241; 9179845; 9044276; 2040302; 1658569 |
| NTL01EC03349 | 8 | 20367472; 40528; 10675322; 1372899; 6462175; 1938886; 1335324; 7108955; 3323848; 3012272 |
| NTL01EC03377 | 18 | 18535149; 16828282; 15322097; 12039966; 1094454; 8811884; 2111004 |
| NTL01EC03381 | 18 | 1970641; 1429514; 2777768; 4519026; 6294056; 6381513; 7017282 |
| NTL01EC03383 | 18 | 21048841; 20943775; 20707314; 20686048; 20606011; 20599757; 20558222; 20348389; 20190041 |
| NTL01EC03384 | 15 | 20923236; 20233303; 20227844; 19936829; 19833764; 19124769; 10966639; 19467815; 7501460; 18983256 |
| NTL01EC03399 | 18 | 21281641; 20855510; 20233931; 20154103; 19782138; 19668863; 19556290; 19555049; 18830684; 18759453 |
| NTL01EC03402 | 18 | 20855510; 20662775; 20233931; 19556290; 19132541; 18385983; 14871133; 8595861; 9922258; 8609408 |
| NTL01EC034427 | 8 | 19703229; 18314671; 14734015; 8247125; 16164606; 16155230; 16155228; 11280008; 10816581; 9608522 |
| NTL01EC03538 | 8 | 21078123; 21073854; 20496859; 19616102; 19589965; 19307254; 19191964; 11950561; 18765916; 18390572 |
| NTL01EC03544 | 3 | 20938646; 20871101; 20824106; 20382766; 8862584; 20061477; 20225957; 8386125; 19522648; 19019161 |
| NTL01EC03545 | 3 | 21050842; 20861263; 20833131; 20691236; 20623219; 20487577; 20443665; 20140959; 20066963; 20060404 |
| NTL01EC03552 | 3 | 21371830; 21256827; 21220027; 21184397; 21036155; 20959463; 20954237; 20943858; 20933222; 20878800 |
| NTL01EC03553 | 3 | 21182591; 20331963; 19269748; 19875450; 19669626; 19085034; 17580962; 17546391; 17314256; 16797479 |
| NTL01EC03574 | 6 | 21047263; 9814711; 19072585; 10890893; 18606573; 16135232; 17027027; 16895921; 10636841; 8878475 |
| NTL01EC03588 | 18 | 19727945; 1482126; 11739766; 10433720; 10512697; 1569007; 9878355; 10512697; 1647022; 1489656 |
| NTL01EC03623 | 6 | 21390258; 21383955; 21349650; 21327042; 21321378; 21299647; 21292986; 21275343; 21293366; 21163839 |
| NTL01EC036427 | 8 | 20697693; 11860549; 10931908; 10878120; 9006056; 2495266; 8704981; 7855437; 5335892; 8125345 |
| NTL01EC03650 | 5 | 21367971; 20451305; 19652522; 19165584; 18279655; 17379537; 16082410; 12054050; 12044898; 11842094 |
| NTL01EC03659 | 8 | 1827992; 9614129; 7836433; 2895706; 6278247 |
| NTL01EC03661 | 6 | 21320585; 20547974; 20032314; 20008464; 16245325; 18426891; 19591841; 19446527; 19407389; 18565343 |
| NTL01EC03671 | 18 | 20707314; 1429629; 7836444; 4284300; 11320319; 10318813; 8878033; 3086314; 7582021; 7891312 |
| NTL01EC036747 | 8 | 21255330; 19118356; 17854404; 15668000; 12634340; 10658664; 9680210; 9579062; 8522138; 7596284 |
| NTL01EC03686 | 1 | 21106925; 20817725; 20482655; 20332210; 20213441; 19948253; 19931317; 19710390; 19699761; 2248769 |
| NTL01EC036957 | 2 | 21375472; 21364950; 21362081; 21352808; 21335525; 21327297; 21304598; 21296138; 21290168; 21287302 |
| NTL01EC03714 | 2 | 21272955; 19828456; 16343536; 12369042; 10617659; 6290489; 9446639; 1851483; 7487088; 7372605 |
| NTL01EC03740 | 1 | 10931901; 2203728; 4289755; 3023184; 781293 |
| NTL01EC03756 | 8 | 18765906; 15282958; 10196759; 8389549; 1601132; 2184897; 2186712; 821926; 6197902; 6452124 |
| NTL01EC03757 | 9 | 21362022; 21352884; 21257607; 21253866; 21247997; 21212688; 21209787; 21106133; 21097599; 20966403 |
| NTL01EC03792 | 13 | 12941704; 10625642; 1400219; 8422961; 1644789; 3311729; 2166215; 779788; 6182300 |
| NTL01EC03824 | 9 | 21195206; 20956528; 20798167; 20702701; 20656373; 20600072; 20528952; 20478340; 20455949; 20434430 |
| NTL01EC03835 | 2 | 21148672; 20887388; 20208449; 19946060; 19933584; 19915116; 19897617; 19880634; 19880632; 19854874 |
| NTL01EC03839 | 4 | 21392133; 21389117; 21386816; 21383145; 21383064; 21381230; 21378306; 21378260; 21378199; 21376595 |
| NTL01EC03846 | 1 | 20381632; 16216875; 7709683; 8507831; 16667223; 2496980; 3139660; 3910040; 3003511; 6383377 |
| NTL01EC03880 | 17 | 20660485; 15998636; 19111651; 16631197; 12777497; 11929549; 11796212; 9659923; 9139668; 7590257 |
| NTL01EC03904 | 14 | 20404044; 4009615; 19196760; 4077987; 10074353; 1531010; 17324932; 16988141; 16985054; 16585211 |
| NTL01EC03907 | 1 | 19596340; 10572016; 18216013; 10913262; 17546672; 17490766; 17442255; 9721288; 17302437; 16708165 |
| NTL01EC03911 | 9 | 20803137; 20549193; 17435249; 2180908; 19782152; 18344982; 19026012; 18774944; 18760332; 18586946 |
| NTL01EC039317 | 8 | 8449875; 4587612; 7005896; 3005273; 6259126 |
| NTL01EC03937 | 15 | 21299643; 21183573; 20920248; 19372162; 15968059; 14617183; 8332062; 1911945 |
| NTL01EC03956 | 15 | 21037009; 20695895; 20233930; 16452450; 19400770; 17526704; 18433630; 18179421; 17526841; 16549675 |
| NTL01EC040127 | 8 | 10613841; 1537846; 2684786; 2851497; 2852352; 2830169 |
| NTL01EC04060 | 6 | 20921378; 20444702; 20018207; 8505306; 17951114; 19451214; 8300203; 1579457; 19360696; 19100865 |
| NTL01EC04201 | 3 | 8441765; 16514161; 15758242; 10931317; 15251210; 12890032; 11065360; 10096084; 3011407; 1362447 |
| NTL01EC042137 | 8 | 3083215; 3929016; 6420508; 6363615; 6406797; 6353163; 4570160; 5432063 |
| NTL01EC04238 | 6 | 21393052; 21382108; 21375466; 21370034; 21369601; 21364495; 21362504; 21358206; 21347607; 21343449 |
| NTL01EC04239 | 6 | 21393220; 21384214; 21377424; 21358277; 21350580; 21347205; 21346871; 21345952; 21345949; 21345948 |
| NTL01EC04250 | 6 | 21393049; 21390258; 21386871; 21385355; 21383955; 21378986; 21376749; 21372283; 21371996; 21369956 |
| NTL01EC04284 | 15 | 21393367; 21388379; 21368293; 21357625; 21343340; 21342462; 21340684; 21338289; 21282331; 21282101 |
| SAK_0286 | 11 | 21387833; 21372787; 21366817; 21365916; 21365490; 21316445; 21284259; 21261079; 21261057; 21248708 |
| SAK_0384 | 17 | 20730136; 20724389; 20660219; 20512976; 19963062; 19583999; 19199915; 10913250; 18289874; 9685179 |
| SAK_0517 | 4 | 9712787; 17205473; 18078694; 18048918; 17259175; 15653821; 15753100; 10411737; 15493827; 15044471 |
| SAK_0646 | 11 | 15758239; 16009540; 11368915; 10860721; 2674324; 6210848 |
| SAK_09364 | 8 | 21241472; 21229881; 20824214; 20684602; 20707404; 20199575; 20194103; 20161474; 19825675; 12810705 |
| SAK_0944 | 8 | 17385548; 16771665; 15103625; 12866046; 11441020; 9218434 |
| SAK_1053 | 8 | 20037925; 18956756; 12702311; 10216875; 16496400; 16473032; 15502322 |
| SAK_11064 | 8 | 12731863; 11741943; 11717516; 1463743; 2294058; 1544915; 2102832 |
| SAK_1175 | 18 | 17397836; 1763075; 15604753; 7896809; 11706182; 9465122; 16667133; 16653217; 9350997 |
| SAK_1232 | 18 | 9254694; 11328886; 18592219; 18349501; 16790427; 17559518; 6336730; 16790427; 16689696; 16158222 |
| SAK_1247 | 3 | 20574043; 7961510; 19965467; 19740656; 19666032; 19081938; 18716769; 16721893; 18389116; 18378033 |
| SAK_1320 | 12 | 20966562; 19465660; 19912341; 11854229; 19152799; 11462195; 17400016; 10948119; 10531064; 8501066 |
| SAK_1476 | 18 | 20959127; 20431058; 20332621; 19794400; 19239022; 14976163; 12370264; 19049391; 18158358; 10683381 |
| SAK_1604 | 18 | 19670073; 19472210; 17350864; 14578044; 1715859; 6256652 |
| SAK_1752 | 2 | 19878958; 8910497; 19392660; 15597200; 15356000 |
| SAK_2070 | 4 | 20833330; 20516206; 20373984; 7784092; 20083156; 19934298; 19838021; 19826964; 19465682; 19269918 |
| SAK_2108 | 18 | 20643857; 19132541; 12136096; 18160489; 10331874; 17227757; 10201093; 15364914; 8595861; 14514697 |
| SP70585_0116 | 4 | 21106176; 20880145; 20733041; 18443115; 20381193; 20368405; 20308385; 20185550; 20164270; 20156257 |
| SP70585_0146 | 15 | 20172998; 19783632; 12732971; 10323238; 10436918 |
| SP70585_0152 | 18 | 19618662; 16536916; 12922132; 12895305; 4646552; 9734476; 9219725; 8663292; 8629089; 7517936 |
| SP70585_0237 | 6 | 21387278; 21357297; 21354867; 21315413; 21227005; 21205011; 21107085; 21103398; 21089674; 21060849 |
| SP70585_0314 | 8 | 21290439; 20529682; 20348906; 19554584; 19222084; 18838820; 7793070; 17536929; 18179582; 17985886 |
| SP70585_03284 | 8 | 20593184; 19735442; 19111640; 8344920; 18436565; 18183386; 10917913; 14715664; 12534822; 11354457 |
| SP70585_0351 | 2 | 21333632; 21193302; 20851095; 20536259; 20452776; 20383751; 2581252; 19631695; 19591883; 19564691 |
| SP70585_03674 | 8 | 16636444; 11420377; 10878120; 8521952; 9466926; 8125345; 2195546 |
| SP70585_0383 | 8 | 21205211; 20155483; 19328460; 18984017; 18640127; 17484020; 3903497; 15590681; 15162497; 14687575 |
| SP70585_0399 | 3 | 21161561; 20882772; 20441706; 20223804; 20194703; 19919674; 19746934; 19736537; 17488738; 19432519 |
| SP70585_0590 | 4 | 21388923; 21388140; 21384193; 21381381; 21378051; 21369871; 21350709; 21347827; 21338785; 21338780 |
| SP70585_0683 | 12 | 20819954; 18783432; 15749831; 7039409; 15368846; 12176387; 16535128; 7528082 |
| SP70585_0735 | 15 | 21197711; 21131496; 20395036; 20382770; 20384681; 20362849; 19451242; 18439357; 19705970; 19703107 |
| SP70585_0743 | 4 | 20154137; 20100826; 18232718; 15883889; 12138775 |
| SP70585_0747 | 4 | 20870765; 19635793; 8181761; 18832310; 10610805; 10978550; 18208530; 17185541; 12626683; 15165235 |
| SP70585_0960 | 5 | 20939536; 20832688; 20149107; 20036411; 1829347; 19278345; 9361443; 10768953; 18721677; 18256468 |
| SP70585_1034 | 6 | 21124948; 19635595; 19563116; 18393679; 19719636; 17452361; 2659796; 17929923; 17881053; 7574479 |
| SP70585_1148 | 6 | 21193388; 20180846; 19783470; 19332813; 18663525; 12937411; 17630395; 17180708; 11796641; 1694554 |
| SP70585_1268 | 18 | 21383000; 21358545; 21320349; 21317882; 21310991; 21311022; 21292979; 21290314; 21170894; 21170880 |
| SP70585_1520 | 3 | 8626331; 19432519; 15251431; 10753902; 15961396; 8530464; 8366124; 6774970 |
| SP70585_1629 | 18 | 19846745; 12422272; 16670299; 18310129; 17693766; 17151144; 17120766; 17120765; 17120763; 17071331 |
| SP70585_1688 | 12 | 9726890; 3123462; 14622347; 10496897; 12416795; 10348851; 10532372; 11401725; 9673015; 11004403 |
| SP70585_1772 | 15 | 21296958; 20965199; 20870760; 20580885; 17005013; 20547748; 20530443; 20529681; 20497331; 20479645 |
| SP70585_1777 | 17 | 19834340; 19199915; 17822967; 16735737; 14986719; 12727285; 7544123; 2549050 |
| SP70585_1792 | 18 | 21131949; 21125390; 20829432; 20598524; 20392300; 18647834; 20107111; 11292651; 20053979; 19825850 |
| SP70585_1812 | 3 | 20707404; 19825675; 18508770; 18043952; 17955483; 15552059; 15296732; 15161861; 15075344; 14987996 |
| SP70585_1836 | 4 | 21362550; 21149262; 21098121; 20885787; 20797633; 20797621; 20647503; 20308424; 16327805; 20237820 |
| SP70585_2200 | 12 | 20876506; 20300807; 19282216; 17434520; 15644794; 15118948; 12694309; 12169531; 11761376; 9561414 |
| SP70585_22411 | 8 | 20667964; 19457242; 20442709; 20012965; 19683668; 19521849; 18944926; 18944623; 18944547; 18838871 |
| SP70585_2269 | 3 | 21391922; 21391781; 21386820; 21380936; 21373681; 21372541; 21372250; 21369715; 21358590; 21357303 |
| SP70585_2278 | 18 | 21377750; 19732985; 18930051; 17409207; 17110979; 10085014; 10217779; 12631210; 12490324; 388356 |
| TP_0078 | 3 | 4595206; 19552981; 10816523; 15558817; 10464206; 10585466; 15470099; 12618454; 12624213; 12624193 |
| TP_0089 | 15 | 21394209; 21357734; 21347269; 21307217; 21290241; 21254629; 21245734; 21215389; 21209202; 21209198 |
| TP_0106 | 18 | 21377632; 21366542; 21364531; 21359964; 21348301; 21325265; 21308987; 21258656; 21256917; 21220704 |
| TP_01151 | 2 | 21394897; 21394810; 21394809; 21394738; 21394667; 21394603; 21394564; 21394451; 21394446; 21394385 |
| TP_0219 | 15 | 20643850; 19543710; 10932251; 14527287; 17575449; 8908813; 16483748; 15256570; 10068952; 7667267 |
| TP_0486 | 3 | 19879894; 18154079; 15147004; 14629271; 10776844; 10451122; 10048170; 1372635; 1356932; 8538575 |
| TP_0574 | 3 | 20580091; 20190044; 20097301; 18803708; 12856214; 12196546; 11442827; 11278314; 10973948; 10829017 |
| TP_0746 | 8 | 21261075; 21186173; 20557574; 20455436; 20227379; 20202167; 16880; 19825618; 19705487; 15685292 |
| TP_0852 | 12 | 19454708; 12207705; 19383708; 18954360; 18641340; 18602442; 18424736; 17090541; 17041050; 17071755 |
| TP_0872 | 4 | 21143315; 16487743; 8071222; 19153448; 12904785; 16677309; 10063642; 15196929; 15170399; 12066889 |
| TP_0950 | 17 | 21347352; 21164034; 21053045; 21045079; 20627997; 20523128; 20476778; 20473316; 12198167; 18789947 |
| TP_0971 | 4 | 21061760; 20007577; 10623848; 19380108; 19282839; 19202004; 9603964; 18205661; 10490989; 7642287 |
| trd_0002 | 6 | 20675375; 20223211; 16507358; 16430690; 15758241; 12940977; 14002700; 12535532; 11859073; 11823461 |
| trd_0031 | 3 | 20400541; 19917666; 19426133; 19421452; 11173485; 15572779; 18815882; 17704511; 17597614; 11118459 |
| trd_0052 | 6 | 21262799; 21223577; 20975945; 20197438; 8995522; 18363794; 10829079; 17720706; 17944831; 10048034 |
| trd_00790 | 4 | 21324123; 20523963; 20113006; 19616076; 19807025; 9143125; 18786786; 16385055; 17602684; 11823842 |
| trd_0135 | 18 | 20154136; 1500854; 11162101; 17957113; 2196428 |
| trd_0136 | 18 | 10427000; 16787368; 14709040; 1569007; 10702268; 9890669 |
| trd_0143 | 15 | 15900880; 1541297; 2002038; 1993703; 2211640 |
| trd_0144 | 8 | 21338421; 19961881; 8181752; 8375392; 8219277; 1802033 |
| trd_0157 | 8 | 21373768; 21209092; 20961849; 20926573; 20818167; 20731414; 20675006; 20672323; 20533364; 20516622 |
| trd_0191 | 18 | 20367639; 20232316; 12867445; 19780400; 19640849; 18830684; 15135918; 18592219; 18313788; 17980698 |
| trd_0202 | 18 | 21223322; 19664060; 18980183; 7585959; 18184765; 7030739; 17030505; 16945564; 16534626; 16505972 |
| trd_0206 | 6 | 21335340; 21316374; 21255416; 21245349; 21241792; 21216248; 21205014; 21192789; 21185254; 21147118 |
| trd_0237 | 13 | 20190091; 19754882; 17907785; 16527306; 10089390; 3579284; 12063391; 10794178; 8123787; 8385698 |
| trd_0251 | 18 | 21122131; 20855510; 20220788; 20154136; 20132828; 11162101; 18383009; 18310026; 17064364; 16445940 |
| trd_0257 | 18 | 20132828; 16665774; 3060089; 18584243; 18310026; 18252722; 11872826; 17497305; 17434534; 17062614 |
| trd_0298 | 9 | 17899070; 17891922; 15708363; 15668256; 12770824; 9388293; 9438344; 8341260; 1547954; 16665177 |
| trd_03227 | 8 | 21279661; 21227686; 21177104; 21177025; 21153851; 20970942; 20964703; 20929952; 20655745; 20639328 |
| trd_03332 | 8 | 20580433; 20149100; 19472043; 19422848; 19306371; 19240958; 18765295; 18370011; 18214990; 16685652 |
| trd_03710 | 4 | 21380777; 21252202; 21223330; 21128072; 21115813; 20954405; 20878239; 20838774; 20830297; 20825482 |
| trd_0395 | 18 | 11050157; 3301822; 15060731; 11401703; 11341969; 390313; 2664762; 1738314; 2821267 |
| trd_04291 | 8 | 20628005; 18563854; 20406851; 20215562; 20111869; 19890225; 19546240; 19487245; 19470663; 19450126 |
| trd_04420 | 4 | 21326917; 21091506; 20471115; 20185507; 10786831; 19850618; 11847086; 19634881; 18261805 |
| trd_0452 | 3 | 21229881; 21030539; 20707404; 20347067; 20188057; 20161474; 15639242; 18258263; 16040347; 11500481 |
| trd_04557 | 8 | 20118267; 20047909; 10989426; 17074891; 11234931; 16260293; 1060145; 15188393; 10591844; 12079877 |
| trd_0468 | 8 | 17929940; 16546999; 15520003; 9696761; 12605683; 12054871; 11959121; 11334785; 10216160; 9485416 |
| trd_0487 | 12 | 20709811; 20674680; 20575438; 19828817; 15158598; 8314771; 17920063; 14583620; 1613398; 12610653 |
| trd_0513 | 12 | 20420854; 20350528; 19552712; 19288213; 19118453; 10829079; 18205165; 111762; 16529946; 16188943 |
| trd_0544 | 15 | 21191097; 21124317; 21051340; 20833806; 20662065; 20577843; 12529311; 20543847; 20507260; 20471686 |
| trd_0558 | 8 | 21391497; 21384159; 21382338; 21368117; 21348614; 21344856; 21342604; 21329214; 21324314; 21301872 |
| trd_0580 | 4 | 18263765; 19022176; 18266306; 12218013; 16650858; 12940979; 12715874; 12589439; 11816965; 11707113 |
| trd_0594 | 8 | 21382338; 21324314; 21239558; 21179059; 21109418; 21092633; 21042417; 20924414; 20854917; 20737854 |
| trd_05971 | 2 | 21107687; 20593487; 20111070; 19780837; 19777229; 18538729; 17511471; 16514565; 16508696; 15966868 |
| trd_0648 | 8 | 20557447; 20369854; 20219383; 19881207; 19759613; 19552509; 19217387; 16985102; 18157663; 17881660 |
| trd_0659 | 3 | 20688825; 20622068; 20566690; 11371519; 769835; 17901900; 10699503; 16963083; 9831648; 7542800 |
| trd_0679 | 12 | 21339618; 21339577; 21338570; 21304597; 21215441; 21195087; 21139638; 21104364; 21059357; 20974141 |
| trd_0690 | 6 | 12466527; 18716622; 19614620; 19411831; 19336243; 17925038; 18643316; 10671452; 11181992; 12535540 |
| trd_0708 | 8 | 19701717; 18519036; 16299377; 16040611; 9884221; 9428682; 9315721; 9298948; 9219517; 9079667 |
| trd_0758 | 8 | 21298162; 20932751; 20589635; 20305021; 12440767; 19324096; 16491128; 19082487; 18951975; 17908933 |
| trd_0799 | 17 | 10580156; 2693905; 8346018; 7489918; 7746156 |
| trd_0804 | 11 | 21284855; 21248336; 21226769; 21193522; 21134004; 21126860; 21114236; 21106922; 21088895; 21071156 |
| trd_0854 | 4 | 21341541; 21233456; 21076891; 20956619; 20868231; 20727719; 20687968; 20655097; 20649881; 20643115 |
| trd_0865 | 5 | 6997270; 16678853; 16598824; 10872445; 15248747; 14615483; 11102525; 1987126 |
| trd_0899 | 1 | 21128869; 19549189; 17896178; 17595805; 17573062; 16820168; 15865426; 15840047; 11371182; 11169018 |
| trd_0902 | 18 | 21369825; 20855510; 20662775; 20408914; 20154136; 20132828; 10827169; 19715704; 19416927; 11805094 |
| trd_0919 | 13 | 11234015; 19625339; 19170749; 7592815; 18845387; 17439913; 16823698; 16523341; 16376935; 12815652 |
| trd_0931 | 12 | 21084296; 20941418; 19379766; 19303951; 17587577; 18930847; 18590836; 18325786; 18049927; 17350041 |
| trd_0975 | 13 | 20600110; 7731806; 7677746; 2504932; 10561595; 10561594; 2199796; 1091919; 9004854; 8722036 |
| trd_1008 | 4 | 18707611; 18421485; 18068424; 17477524; 16735739; 16637653; 16022515; 16348944; 10427081; 12377116 |
| trd_1032 | 13 | 19664587; 15135053; 9843401; 14501142; 10924141; 10529181 |
| trd_1039 | 15 | 21393212; 21369825; 20150239; 20133363; 20107022; 19904424; 19887527; 19846118; 19577910; 19438714 |
| trd_1062 | 5 | 8064853; 16396446; 15773988; 11094274; 2294058; 6223625; 1482126; 7860593; 1732230 |
| trd_1096 | 8 | 20483646; 19889946; 18762190; 18483063; 17553902; 17544406; 15988697; 15081823; 15020257; 12935880 |
| trd_1152 | 8 | 21265769; 21185507; 21174460; 21115710; 21092069; 21033146; 20971858; 20945759; 20870960; 20863064 |
| trd_1157 | 18 | 21328631; 21282456; 21134356; 21081547; 21062372; 21042264; 20955466; 20951729; 20882639; 20855745 |
| trd_1169 | 15 | 21207455; 21093452; 20804771; 16406067; 20547810; 17589500; 20012281; 16740948; 14617778; 19632156 |
| trd_1170 | 18 | 21343396; 21256461; 21248166; 21209283; 21209222; 21187326; 21179170; 21177413; 21173115; 21140912 |
| trd_1174 | 18 | 21338716; 21252226; 21244361; 21161099; 21143902; 21109292; 21084748; 21044933; 20969477; 20959918 |
| trd_12157 | 8 | 20659147; 20139318; 16104863; 10388694; 10433972; 7721710; 1067620; 2516831; 9548955; 8626276 |
| trd_1221 | 8 | 20466730; 20050916; 18687332; 18174132; 17623665; 14609319; 16920107; 12524212; 11744735; 9914305 |
| trd_1223 | 8 | 10548535; 10632883; 9524269; 7715602; 7715602; 7715601 |
| trd_1269 | 12 | 10455123; 6416164; 2692535; 3543005; 3510201; 3522590; 6378913 |
| trd_1282 | 8 | 21074581; 21074536; 20947575; 16541135; 19196274; 10810734; 10366527; 4204433; 18081191; 9396791 |
| trd_1284 | 13 | 21210840; 16945113; 11677252; 2984573; 9109519; 7989373; 2380906 |
| trd_1337 | 18 | 18767164; 14962940; 15274913; 15141299; 4290867; 1995346 |
| trd_13437 | 8 | 11747447; 9772161; 1892834; 8987982; 7703246; 7855594; 1892834; 2271624; 2215699; 2099737 |
| trd_1388 | 4 | 17038198; 17090920; 15301547; 11081795; 10727248; 10651637; 10382261; 2183008; 2183008; 13416270 |
| trd_13897 | 8 | 21370851; 12873137; 15202771; 15170325; 7411608; 10037452; 9758418; 7980602; 8432998 |
| trd_1395 | 8 | 20208179; 19837158; 17805928; 17804419; 17729270; 10633095; 17595116; 16042421; 15451173; 14646093 |
| trd_1398 | 18 | 21393174; 21392495; 21389634; 21389261; 21383772; 21382175; 21378036; 21375706; 21374643; 21372393 |
| trd_1403 | 18 | 21179170; 20849418; 20802201; 20581204; 20559624; 20395296; 20393069; 20184887; 3537305; 19706287 |
| trd_1451 | 18 | 15168617; 9534236; 8713463; 8223559; 1530922; 2158904 |
| trd_1459 | 3 | 19815704; 16945597; 16407124; 16313167; 14644550; 1943775; 11856302; 10869181; 10570974; 10393243 |
| trd_1468 | 8 | 21166653; 18505683; 10759516; 16641103; 12534290; 18096698; 17550420; 17302434; 10388564; 16633561 |
| trd_1495 | 8 | 19941855; 16817900; 11292729; 11289261; 10426958; 9234667; 6378632; 6200109; 6407398 |
| trd_1524 | 18 | 20488669; 1699669; 19522956; 19131246; 18620036; 17924465; 17887660; 17662692; 13678827; 17465479 |
| trd_1537 | 18 | 21315728; 21131908; 20881245; 20877283; 20855745; 20739103; 20706981; 20656779; 20573661; 2017436 |
| trd_1602 | 3 | 20713603; 20573954; 20444215; 20356564; 20160053; 20035319; 15448272; 19919534; 19856494; 19421452 |
| trd_1616 | 18 | 21040511; 20963614; 20377750; 19190178; 19804410; 15660156; 19735320; 19292998; 1737021; 19187219 |
| trd_1631 | 12 | 20621724; 20507874; 20410261; 20224257; 20140974; 20067154; 19580842; 19458713; 18979629; 18690782 |
| trd_1644 | 11 | 21349326; 21241792; 21214923; 21192634; 21144870; 21138844; 21097633; 21079776; 21062824; 21041684 |
| trd_1658 | 4 | 21385202; 20851903; 17028235; 18480330; 10594817; 16388033; 16151207; 2905265; 10658653; 9254694 |
| trd_1677 | 12 | 21109561; 20803137; 20087337; 19878324; 18039771; 19446023; 7501460; 18211573; 18507684; 18193200 |
| trd_1687 | 9 | 21393852; 21393244; 21392972; 21392566; 21392564; 21391204; 21389144; 21388805; 21388804; 21387258 |
| trd_1697 | 12 | 20422176; 18045787; 19552712; 18350392; 1597410; 17320992; 17223214; 12969510; 16099524; 15313226 |
| trd_1746 | 4 | 21392133; 21385724; 20675719; 17724149; 19635803; 20406405; 20348848; 20082462; 20032382; 19889886 |
| trd_17584 | 8 | 21392884; 21385562; 21384831; 21384355; 21383196; 21371041; 21369980; 21360899; 21357773; 21355557 |
| trd_1773 | 8 | 20888212; 20688172; 20675294; 20518346; 20353187; 19943898; 19899738; 19751796; 19651103; 18388293 |
| trd_1776 | 18 | 20809990; 19090784; 17910951; 17680703; 17317524; 16645309; 16616607; 10781104; 15379567; 15364580 |
| trd_1781 | 8 | 21061628; 20654728; 20636270; 20613764; 20528950; 20214873; 18651753; 20106908; 19635800; 16611942 |
| trd_1813 | 15 | 19116917; 18777041; 17287440; 16725275; 16616847; 16000707; 15680700; 15583171; 15183729; 13129619 |
| trd_18244 | 8 | 20962348; 20802066; 20605132; 20562284; 20384789; 20349929; 20304632; 20057070; 19825957; 19786581 |
| trd_1849 | 3 | 21229881; 20707404; 20347067; 15639242; 11500481; 18499663; 18210176; 18203712; 17725566; 17591617 |
| trd_1853 | 1 | 21195720; 20075610; 17873297; 15955309; 15064875; 12946365; 11993511; 11778878; 11511866; 11167013 |
| trd_1862 | 18 | 3301822; 6051349; 14568145; 9680220; 9265630 |
| trd_1868 | 8 | 21239585; 20383020; 20371327; 19904424; 19409514; 9233812; 18682379; 15103330; 11123699; 12054771 |
| trd_1877 | 8 | 11418115; 10331925; 7902351; 2265761; 1503531 |
| trd_1883 | 18 | 21282456; 21081547; 20855745; 20610168; 20573661; 20525733; 20508090; 20399647; 20111865; 20017731 |
| trd_1892 | 3 | 21385202; 21342541; 21347376; 21317159; 21203384; 21166709; 21126315; 21106106; 21102601; 21098513 |
| trd_1924 | 4 | 20428807; 11430835; 18457419; 15916962; 18310048; 16322744; 16014621; 10411726; 15636744; 11591668 |
| trd_19304 | 8 | 21354427; 21193826; 20871989; 20669918; 20581746; 20552260; 12175810; 20159465; 20124706; 20077115 |
| trd_1950 | 18 | 21059948; 20959448; 20659291; 20656779; 20655739; 20578693; 10383958; 20173761; 20147285; 20132828 |
| trd_1963 | 4 | 15819619; 17172014; 9746358; 7783616; 7968523; 4923158 |
| trd_1964 | 9 | 20956528; 20455949; 20185506; 17575454; 16778352; 16133338; 15938393; 1834913; 14593926; 10984546 |
| trd_1968 | 3 | 21301102; 20847002; 20843347; 20013255; 19924480; 19919674; 16146521; 16339737; 1765138 |
| trd_1971 | 18 | 21357486; 21319715; 21277974; 21182588; 21178073; 21070747; 21059110; 21039732; 21037180; 21036337 |
| trd_1979 | 17 | 21135120; 20966049; 20926373; 20924356; 20871972; 20818334; 20675469; 20668468; 20615954; 20553390 |
| trd_A0033 | 4 | 1372311; 9168127; 6327646; 7855428; 3555840 |
| trd_A0041 | 17 | 7592475; 7602586; 3019840; 2174860; 808854 |
| trd_A0075 | 8 | 21261075; 20455436; 16880; 20012884; 16299075; 19351043; 17977448; 16879645; 16573681; 16569613 |
| trd_A0077 | 15 | 20594941; 20133363; 16945692; 16953632; 4352175; 18245243; 18604637; 18093135; 18022383; 12358600 |
| trd_A0097 | 9 | 20851904; 19345228; 16906759; 16521160; 16378734; 12709059; 12616642; 11690636; 11477098; 11470804 |
| trd_A0125 | 4 | 20729274; 20448453; 19840121; 18452192; 18271004; 17559337; 17135279; 16396494; 15468914; 12359327 |
| trd_A0127 | 11 | 11717308; 1920611; 9023344; 9367380; 1282282 |
| trd_A0150 | 2 | 20006999; 19469573; 16305243; 12206660; 11248251; 7499269; 7672130 |
| trd_A01752 | 8 | 21303534; 21196297; 21119085; 20650322; 20649633; 20588251; 20435817; 20369289; 20079354; 20065299 |
| trd_A01794 | 8 | 21303663; 21293880; 21261081; 21252225; 21235167; 21190955; 21182826; 21094225; 21093057; 21088172 |
| trd_A0193 | 8 | 11695917; 9724544; 7945350; 2159881; 3034161 |
| trd_A0248 | 18 | 16979296; 7678431; 16549396; 8254763; 11994164; 14687586; 14572912; 12426518; 12217256; 11826290 |
| trd_A0251 | 18 | 20969648; 18024100; 2205530; 9006953; 9341163; 7767600; 8437564 |
| trd_A0253 | 14 | 16877802; 16310098; 8693839; 8504933; 7906571; 2835631; 3839509 |
| trd_A0262 | 2 | 20663063; 20532401; 20170126; 20079748; 19716624; 19462053; 17746926; 12820902; 17240979; 17190832 |
| trd_A0346 | 18 | 21317882; 20656904; 20573964; 20097755; 18690031; 17344221; 16210320; 15459199; 9525859; 10359077 |
| trd_A0349 | 8 | 21369923; 21102625; 20929591; 20885787; 20678977; 20651027; 20616095; 20598276; 20592034; 20573820 |
| trd_A0356 | 8 | 21272918; 21257036; 21247988; 21240476; 21198961; 21085948; 21073729; 20965335; 20938718; 20869341 |
| trd_A0370 | 18 | 21063407; 21063390; 20974830; 20398901; 20305123; 9431990; 11286892; 19558960; 19300998; 19258414 |
| trd_A0386 | 8 | 21343423; 21281738; 21185310; 21169901; 21104973; 21062028; 20923693; 20883155; 20876192; 20868231 |
| trd_A0393 | 8 | 4287829; 17882654; 14515334; 12095623; 11929530; 11334784; 7999130; 271968; 1332965; 1851043 |
| trd_A0415 | 3 | 21317537; 21303167; 21281703; 21266973; 21247434; 21186804; 21186189; 21097841; 21092491; 21072051 |
| trd_A0453 | 8 | 21382338; 21324314; 21179059; 21092633; 21042417; 20924414; 20854917; 20737854; 20714159; 20696095 |
| trd_A0454 | 8 | 20307490; 17676874; 327024; 16437183; 14766741; 12633849; 9396791; 11115638; 9623806; 9711295 |
| trd_A0520 | 8 | 20724480; 20359206; 20334431; 20213543; 19843169; 19737939; 19624733; 18494801; 19438211; 19424679 |
| trd_A0539 | 8 | 21364306; 21359858; 21339386; 21336645; 21332529; 21329681; 21328323; 21297347; 21295965; 21291872 |
| trd_A0594 | 15 | 20542930; 10517572; 17416591; 16546208; 12460564; 2515118; 9278503; 2020552 |
| trd_A06397 | 8 | 21305026; 19184529; 11306094; 10978349; 9772162; 2187374; 6614900 |
| trd_A0643 | 4 | 19732341; 18599076; 16630628; 15196929; 12753195; 11327763; 10320579; 10712687; 10564473; 10478459 |
| trd_A0681 | 3 | 21329659; 21212096; 20975832; 20689156; 20595232; 20426787; 20185821; 19797682; 14516279; 19289152 |
| trd_A07402 | 8 | 20353187; 4027239; 18251505; 18186483; 18179257; 16878980; 1126943; 16300392; 10368302; 12549903 |
| trd_A0750 | 15 | 21169483; 10606655; 7836297; 15819620; 10973967; 5432063; 9409772 |
| trd_A0752 | 2 | 20946885; 16922603; 12767809; 12602878; 12459457; 9267435; 942051; 1445864; 1643048; 1567858 |
| trd_A0769 | 5 | 20884691; 20100286; 17283095; 18550080; 10764768; 15796980; 15090490; 12769720; 12640627; 12559615 |
| trd_A0771 | 9 | 20725044; 19385043; 19560433; 15824287; 15514159; 15329927; 10320401; 18297087; 16823034; 16616010 |
| trd_A0795 | 18 | 21148490; 21144830; 21048710; 21058427; 20931184; 20881005; 20853509; 20849926; 20837673; 20837469 |
| trd_A0803 | 18 | 20497379; 18039771; 19105722; 11356926; 18590228; 10706290; 17304820; 15813727; 15480787; 15240840 |
| trd_A0821 | 18 | 10361286; 18551332; 18462403; 10347673; 17524545; 9679194; 1973153; 12726766; 6546423; 6416164 |
| trd_A0826 | 9 | 21106133; 20736083; 20541551; 20463021; 18281324; 18036338; 3968063; 17537807; 17395278; 16601871 |
| trd_A0836 | 8 | 21057946; 20832504; 20816746; 20589823; 20576606; 20524621; 20372030; 20221546; 20186410; 20179327 |
| trd_A0872 | 12 | 12900386; 20487019; 20363934; 20060433; 17259602; 19556347; 18650432; 9661666; 18412550; 18340545 |
| trd_A0904 | 12 | 10903946; 16513561; 16232749; 16142900; 10379365; 15913357; 12943232; 12031843; 11473256; 11069674 |
| trd_A0928 | 8 | 20487219; 19889630; 19780816; 19759330; 19624744; 6656879; 19387580; 19308642; 19301424; 19182260 |
| trd_A09307 | 8 | 19875455; 17932031; 17442675; 8449871; 7918489; 9209061; 16359327; 16233251; 16233225; 15523913 |
| VC_0018 | 12 | 21322648; 21281627; 20851121; 20730518; 20726783; 20678104; 17090218; 20647321; 20473863; 20304782 |
| VC_0224 | 3 | 21229881; 20707404; 20347067; 19720067; 19298858; 15639242; 11500481; 18518825; 18499663; 18341480 |
| VC_0236 | 3 | 20831592; 20688825; 20622068; 20566690; 20056708; 10829079; 19332829; 19201821; 17517879; 18833547 |
| VC_0305 | 17 | 21126315; 20382767; 768986; 17234211; 15554979; 15554978; 12181321; 9933592; 9642084; 8610017 |
| VC_0330 | 15 | 20675445; 11342591; 20230832; 20207760; 20116460; 19843219; 19696107; 19684063; 16339734; 11277442 |
| VC_0409 | 4 | 21317257; 20487019; 19833774; 16291662; 18819404; 18179420; 17020553; 17005969; 8757877; 16177472 |
| VC_0443 | 4 | 7859746; 18494460; 17242506; 11536333; 9865607; 3906713; 6318439; 1108004 |
| VC_0445 | 12 | 20504072; 20232248; 19112831; 12429090; 2572583; 12777438; 9159398; 2931852 |
| VC_0470 | 6 | 21093407; 20846957; 20533288; 8566804; 20712491; 19448636; 20370819; 20167522; 20145021; 20139319 |
| VC_05310 | 4 | 20975555; 20145952; 17399705; 17114583; 20054112; 19843181; 19512934; 19460918; 19052339; 19021761 |
| VC_0548 | 15 | 21173314; 21078860; 20864514; 17555441; 20100285; 17645733; 19350225; 18310331; 19047758; 18455986 |
| VC_05572 | 4 | 21129419; 21031302; 20704697; 2985470; 9466251; 20060924; 19494576; 19904385; 15743955; 19778962 |
| VC_0565 | 12 | 21245315; 21062493; 20971899; 20942652; 20739286; 20581825; 20512978; 20184896; 20070525; 19836340 |
| VC_06124 | 8 | 19879558; 18346721; 10829036; 944553; 7711751; 1597178; 2166361; 2951252; 2941300; 6174113 |
| VC_0719 | 15 | 21057008; 20834167; 20833804; 20954302; 20363229; 18052041; 12368235; 19220749; 18818215; 18789936 |
| VC_0751 | 12 | 20668094; 17453917; 4882981; 18986169; 15522305; 16843540; 17506526; 17453917; 10869428; 15485839 |
| VC_0752 | 12 | 19821612; 4882981; 18986169; 16843540; 18632665; 17506526; 17453917; 17407764; 10869428; 16920629 |
| VC_0824 | 4 | 17083375; 6874676; 16344011; 1375309; 15158282; 12637499; 12637027; 12501605; 11882717; 10784037 |
| VC_0828 | 4 | 17559395; 18179420; 17962948; 16891156; 15773401; 14678649; 12823812; 12694622; 12674350; 11873401 |
| VC_0843 | 4 | 21294238; 21072897; 21146097; 20508921; 20464759; 19996310; 19082910; 18787045; 18522411; 18493655 |
| VC_0847 | 11 | 20949106; 20875907; 20627350; 20578458; 20406289; 20298189; 17138868; 20022231; 20007650; 12902278 |
| VC_09114 | 8 | 8309940; 17504469; 17185556; 2446923; 2256682; 14606945; 11191810; 8238872; 10816581; 10361281 |
| VC_0993 | 15 | 20959806; 20720545; 2025413; 17507370; 18511939; 19961900; 19706608; 15755726; 17185323 |
| VC_1003 | 4 | 21378051; 21306448; 21195227; 21183019; 21143316; 21141468; 21118552; 21080958; 21078157; 20971906 |
| VC_1136 | 1 | 15363855; 15301532; 9333323; 12224638; 11551184; 11277623; 11264293; 7929415; 10733892; 9654139 |
| VC_1138 | 1 | 8603061; 15363855; 9333323; 11277623; 9654139; 8028028; 14190241 |
| VC_1146 | 8 | 21323311; 21299470; 21275844; 21235502; 21210868; 21194355; 21190518; 21148108; 21094149; 21078302 |
| VC_1206 | 15 | 20139185; 15130126; 19202299; 16921374; 11916378; 11401699; 1577753; 4604283; 7783641; 1846133 |
| VC_1245 | 18 | 21345797; 20173761; 19847921; 12823972; 17673622; 17302441; 16331991; 12890014; 15313236; 15308647 |
| VC_1392 | 6 | 21332028; 21296763; 21236481; 21057210; 21037589; 20978934; 20969519; 20969518; 20926618; 20856823 |
| VC_1434 | 15 | 20522491; 20156450; 12824332; 12112862; 18400007; 17303561; 10525169; 16207915; 16954205; 12546643 |
| VC_1451 | 4 | 21248099; 21220343; 18845756; 20212166; 19434753; 3293491; 18849006; 18807492; 17646359; 12356775 |
| VC_1459 | 4 | 21366345; 21079963; 19900531; 18283731; 17097005; 15916081; 15790030; 14757199; 12534264; 10074546 |
| VC_1512 | 8 | 21357619; 20498375; 17054778; 16221580; 15924426; 14610638; 12679550; 12605683; 12560990; 12409197 |
| VC_1544 | 18 | 20860484; 20643857; 3045756; 19130262; 18973471; 18629473; 11967085; 15993072; 1748657; 6464928 |
| VC_1553 | 15 | 10510236; 9393850; 9179845; 2040302; 1658569; 1556120; 1094240; 3012272; 3038334; 3316209 |
| VC_1769 | 6 | 20599730; 1741244; 16011798; 15629938; 8613993; 16368872; 16207918; 12568936; 11718555; 11555298 |
| VC_1779 | 18 | 10627041; 808529; 1445856; 1569065; 1809844 |
| VC_1836 | 18 | 21098297; 20953507; 20870776; 20816983; 20693330; 20433837; 19696740; 11501670; 19519769; 19493198 |
| VC_1837 | 18 | 21392130; 21285349; 21252278; 21110981; 21079181; 20870776; 20816983; 20693330; 20529089; 20483883 |
| VC_1839 | 18 | 21285349; 20870776; 19650773; 19519769; 19493198; 19075020; 18269247; 10474183; 3915176; 17442676 |
| VC_1950 | 2 | 19778964; 10829079; 10677347; 15366932; 12504898; 11913138; 11278798; 11170471; 10357811; 10948204 |
| VC_1972 | 2 | 15134446; 12615349; 10194342; 3516220; 942051; 6117313; 5089330 |
| VC_2066 | 17 | 10411266; 9573142; 9168617; 8866483; 8861212; 6991880; 8253389; 1453955; 2174860; 2196428 |
| VC_2072 | 12 | 19622614; 19616072; 3519316; 17557914; 17244626; 17143514; 16380485; 15944156; 15866516; 15858821 |
| VC_21452 | 4 | 21343288; 21284983; 21204929; 21194874; 20944228; 20594622; 20398676; 20374902; 20364292; 18849445 |
| VC_2241 | 8 | 20460700; 9748316; 15651867; 12580599; 11926822; 11372197; 9808046; 9613590; 9353915; 9183020 |
| VC_24120 | 8 | 21296938; 20385101; 20160912; 19383527; 17532339; 18675788; 18362922; 18253149; 17157320; 12208141 |
| VC_2415 | 15 | 20707605; 20631133; 20219606; 11350954; 18667418; 9005851; 17890844; 16140031; 16735674; 12998362 |
| VC_2425 | 3 | 20455262; 17468768; 16390451; 15170403; 9157238; 12040098; 11886754; 10026202; 11401692; 10972813 |
| VC_2501 | 12 | 21188170; 21185091; 21128822; 21034747; 20977208; 20969977; 20946870; 20944418; 20861513; 20803144 |
| VC_26910 | 4 | 21349329; 21317318; 21239493; 20799348; 20487295; 11830644; 15271926; 18293996; 13129941; 17259177 |
| VC_2693 | 15 | 21349329; 21317318; 21239493; 21179024; 20601270; 10966457; 19596441; 19445950; 15101969; 13129941 |
| VC_2713 | 15 | 20205655; 19445950; 15101969; 12453229; 16359331; 15713883; 2558046; 12672798; 10564504; 12398213 |
| VC_2724 | 4 | 11349009; 6392294; 17488729; 15533433; 15081815; 7768847; 11111909; 8406031 |
| VC_A0061 | 17 | 20935500; 20569003; 20472641; 20225163; 20225155; 20166751; 20116367; 20026132; 20016128; 18701081 |
| VC_A0218 | 4 | 21329738; 21106267; 20817802; 20817089; 20662368; 17698573; 18963158; 18062266; 17672315; 2339878 |
| VC_A0221 | 9 | 15150325; 16243284; 12536557; 10739951; 10537209; 10419290; 1917947 |
| VC_A0245 | 18 | 20870773; 19400808; 17974510; 17803963; 16079355; 10559156; 15258141; 10319814; 14766911; 12867744 |
| VC_A0387 | 4 | 21394874; 21394325; 21375687; 21369907; 21364771; 21364088; 21357389; 21323710; 21332848; 21309243 |
| VC_A0446 | 4 | 21392537; 21392488; 21392463; 21392344; 21390205; 21386997; 21385872; 21385621; 21383676; 21381373 |
| VC_A0447 | 4 | 21383676; 21353279; 21334038; 21298103; 21293099; 21291170; 21253575; 21249207; 21204021; 21168368 |
| VC_A0513 | 1 | 21380856; 21349875; 21288239; 21267457; 21227480; 21191998; 21182990; 21178262; 21163840; 21146842 |
| VC_A0519 | 15 | 21239488; 20971900; 20935102; 20462860; 20361740; 20219606; 19741270; 19406896; 16236509; 19332837 |
| VC_A0623 | 8 | 21394668; 21376665; 21319302; 21307607; 21290439; 21229467; 21227585; 21219616; 21171652; 21136951 |
| VC_A0625 | 18 | 20586423; 19159260; 10498719; 11282473; 7984417; 15708825; 9886293; 11722746; 10658665 |
| VC_A0683 | 15 | 7957888; 4978942; 8999880; 149110; 1489656 |
| VC_A07004 | 8 | 21393864; 21393839; 21390509; 21385872; 21367878; 21360139; 21359176; 21346408; 21327387; 21310505 |
| VC_A0752 | 8 | 21364950; 21355852; 21335525; 21304598; 21296138; 21295137; 21287302; 21275844; 21248835; 21239621 |
| VC_A0865 | 4 | 20307644; 10692166; 19673410; 19110602; 16381862; 18074211; 10947231; 14738797; 17431549; 17186156 |
| VC_A0910 | 18 | 20643857; 20047910; 19735464; 3045756; 18629473; 4196588; 17623027; 17385904; 11967085; 7642501 |
| VC_A10294 | 8 | 20187119; 18594899; 12026175; 9782510; 10821190; 10729189; 779849; 8366066 |
| YpAngola_0001 | 11 | 8022279; 12602326; 2553542; 11315188; 9729608; 9571135; 9141667; 8382825 |
| YpAngola_0006 | 4 | 21269993; 12423780; 10093708; 9004504; 4627922; 2811901; 745233; 3025701; 13546547 |
| YpAngola_0093 | 11 | 21360615; 21177804; 21154877; 21129200; 20844906; 20739271; 20660769; 20531477; 20447671; 19895817 |
| YpAngola_0114 | 11 | 10559158; 10438765; 11807051; 15036538; 11553538; 8556862; 12749837; 11169105; 10846225; 10217489 |
| YpAngola_A0159 | 5 | 20594968; 20013009; 19349429; 7937744; 18256468; 18042394; 17658188; 17443268; 16753448; 16439861 |
| YpAngola_A0197 | 8 | 21224843; 20067338; 17251179; 19416360; 19286454; 18644963; 18474594; 10390637; 18022196; 17617717 |
| YpAngola_A0202 | 17 | 12864862; 10411266; 10400590; 7623377; 1453955; 9168617; 8866483; 8861212; 7590326; 2174860 |
| YpAngola_A0208 | 4 | 21335384; 21334427; 21118278; 20979349; 19390839; 16349450; 17898843; 18065536; 17396015; 10629180 |
| YpAngola_A0267 | 18 | 21276097; 10209752; 4196588; 17927700; 17628143; 17578453; 17238922; 11967085; 15993072; 15918073 |
| YpAngola_A0325 | 18 | 20093292; 18611376; 17952093; 3017428; 16777062; 10464217; 16335991; 5432063; 12142471; 11081577 |
| YpAngola_A0350 | 18 | 21210849; 20888343; 20954302; 20508181; 20376793; 20233492; 20154136; 20150591; 1699669; 19856269 |
| YpAngola_A0499 | 12 | 20570733; 20378353; 19850919; 19820722; 14663080; 18953686; 18485865; 18485872; 18485076; 17966405 |
| YpAngola_A0702 | 3 | 20847047; 20418257; 18931300; 10438616; 17888883; 19468224; 19284999; 10992472 |
| YpAngola_A07461 | 8 | 20499228; 18848625; 15288928; 10099370; 9851711; 9525938; 8069783; 1730033; 6336729; 2403562 |
| YpAngola_A0751 | 6 | 17381135; 16793370; 8465198; 2549377; 2851707 |
| YpAngola_A08902 | 4 | 20417170; 20061483; 19775890; 19682914; 19493004; 18335939; 10542178; 16630813; 10678906; 18058535 |
| YpAngola_A0917 | 8 | 20937390; 20709408; 20602528; 19690715; 6633513; 12059195; 12599750; 19090787; 11021791; 18350531 |
| YpAngola_A0990 | 18 | 21345797; 21255303; 21111562; 20956785; 20948192; 20876572; 20947627; 20717016; 20712378; 20705604 |
| YpAngola_A1041 | 3 | 21256201; 21227987; 20796283; 20629752; 20444090; 20056705; 17581122; 19895819; 18776014; 19556347 |
| YpAngola_A1212 | 15 | 21097894; 21097887; 21097626; 20951079; 20949070; 20633229; 20395367; 8257110; 1547773; 20132305 |
| YpAngola_A1243 | 6 | 21263027; 21251036; 21227399; 21179576; 21124948; 21115857; 21041657; 21115420; 21112870; 21091445 |
| YpAngola_A1333 | 5 | 21324981; 21170893; 21275894; 21254393; 21228576; 21139284; 20797558; 20705081; 20545623; 20446008 |
| YpAngola_A1350 | 15 | 21094652; 2022622; 10811230; 16905149; 16520374; 12456267; 12690118; 8632012; 8907129; 7805766 |
| YpAngola_A1472 | 11 | 21081488; 10891513; 15223246; 14656464; 11818146; 6096371; 6321770; 1587473; 1492096; 2162963 |
| YpAngola_A1564 | 18 | 20937813; 20159658; 19493013; 16665774; 18690055; 9914396; 10513726; 12225850; 11976340; 11918801 |
| YpAngola_A15897 | 8 | 21392498; 21381897; 21302592; 21252179; 21147242; 21120517; 21119085; 21072576; 20929055; 20798245 |
| YpAngola_A1619 | 3 | 20622068; 20595390; 20219672; 20095950; 19530228; 19454652; 18424515; 19332829; 17984291; 17947697 |
| YpAngola_A1624 | 18 | 21293906; 21239093; 21210779; 21139976; 21115738; 21106523; 21097627; 21094556; 21056572; 21028901 |
| YpAngola_A1680 | 18 | 21336923; 21332893; 21315728; 21254783; 21161516; 20881245; 20875429; 20816060; 20696931; 20592259 |
| YpAngola_A17872 | 4 | 16944097; 16521149; 7661448; 11700361; 9244278; 8288518; 2999794; 2691873; 2716520 |
| YpAngola_A1801 | 8 | 11441462; 9151423; 8014844; 1336799; 3040894 |
| YpAngola_A2028 | 15 | 21332625; 21098025; 20935096; 20889744; 20833811; 19812899; 19703107; 19383707; 7525405; 18563621 |
| YpAngola_A2030 | 8 | 18825405; 17936114; 17853358; 17261587; 15720402; 12480900; 11527960; 10495709; 2080068; 3132906 |
| YpAngola_A2037 | 8 | 19140736; 5700707; 11294639; 16303758; 15723352; 3353366; 9857196; 7730362; 12009917; 11717519 |
| YpAngola_A2053 | 6 | 20705129; 17942115; 9372938; 1387639; 11015204; 9800196; 9464319; 9018044; 1638532; 7513059 |
| YpAngola_A2137 | 8 | 21383180; 21375592; 21295137; 21256830; 21235502; 21222452; 21210868; 21199874; 21190518; 21138988 |
| YpAngola_A2274 | 18 | 21393174; 21389634; 21372393; 21366542; 21362586; 21360409; 21352852; 21351087; 21349151; 21348297 |
| YpAngola_A2354 | 15 | 21324192; 17317137; 17928627; 17167165; 17108331; 17093945; 15385410; 8626065 |
| YpAngola_A2376 | 11 | 20660769; 19426744; 12581642; 17056065; 16476446; 11382219; 15469818; 14569303; 12083526; 11922669 |
| YpAngola_A2470 | 11 | 16731525; 2548993; 11822682; 9302014; 8917074; 8757745; 7557479; 7496528; 7628725; 1660923 |
| YpAngola_A2471 | 11 | 16731525; 2548993; 11822682; 10556026; 10760133; 9302014; 8917074; 8757745; 7557479; 7496528 |
| YpAngola_A2548 | 18 | 21366347; 21178005; 21087212; 21063094; 20937801; 20868366; 20713918; 20702583; 20698827; 20689339 |
| YpAngola_A2563 | 5 | 21387114; 21376122; 21305854; 21288095; 21284340; 21193409; 21192821; 21148296; 21116116; 21097630 |
| YpAngola_A2569 | 8 | 21299470; 21235502; 21190518; 21086077; 21029046; 20978135; 20889785; 20889129; 20888410; 18156657 |
| YpAngola_A2604 | 12 | 17570395; 16246025; 16141198; 10966480; 11106496; 10103005; 9405389 |
| YpAngola_A2652 | 3 | 21208281; 21149452; 21097580; 20980996; 20738399; 20668486; 20566764; 20383019; 20338254; 8662184 |
| YpAngola_A2673 | 4 | 9603889; 19775244; 19696109; 8081742; 18243686; 6761544; 17302795; 10419957; 16089397; 16049010 |
| YpAngola_A28171 | 2 | 19140455; 6099322; 11839308; 11642045; 11104108; 10508664; 10382260; 8619301; 2555951; 1097404 |
| YpAngola_A2860 | 5 | 1766381; 19409905; 19294702; 19006331; 18256480; 17229727; 17139615; 17010375; 16579472; 16545809 |
| YpAngola_A2896 | 3 | 8961949; 8672489; 7703227; 5324647; 8218267; 1536854; 2159466 |
| YpAngola_A2899 | 3 | 7718567; 15498941; 15518577; 16256695; 15518577; 8987972; 8672489; 7718567; 1536854; 1536853 |
| YpAngola_A2901 | 3 | 21057010; 21056982; 21044320; 20978608; 20938646; 20921143; 20871101; 20870764; 20863318; 20860010 |
| YpAngola_A2929 | 15 | 15885105; 12580319; 11160802; 10484571; 1588910 |
| YpAngola_A3018 | 18 | 20693325; 20656779; 20332504; 19332836; 19782883; 1429629; 12591882; 8849412; 1091546; 17438106 |
| YpAngola_A3034 | 4 | 12730182; 15009896; 18001134; 16891058; 271968; 9683479; 1658572; 8836921; 388439; 7934907 |
| YpAngola_A3232 | 12 | 21362403; 20622921; 20036633; 11042190; 3972783; 11179216; 16849325; 16814856; 12634421; 9827548 |
[truncated: 1,543 more chars]
