# Supplementary material for: Automatic Assignment of Prokaryotic Genes to Functional Categories Using Literature Profiling
Source: PLoS One. 2012 Oct 15;7(10):e47436. doi: 10.1371/journal.pone.0047436 (PMC3471813; doi:10.1371/journal.pone.0047436)
Supplement: Table S2 — Independent dataset – Used to test the SVM classifier. (DOC) [file pone.0047436.s004.doc]

INDEPENDENT DATASET – USED TO TEST THE SVM CLASSIFIER

| **Gene** | **Original Category** | **Category Assigned** | **Classification Confidence** | **PubMed IDs** |
| --- | --- | --- | --- | --- |
| BCA_0001 | 6 | 6 | 0.93 | 18835566; 17680349; 16036556; 15611053; 15336485; 12413665; 12366845; 11820935; 10835275; 2539359 |
| BCA_0002 | 6 | 6 | 0.97 | 20675375; 20554816; 20413500; 20357218; 20195500; 20188667; 20176899; 20157337; 20144152; 20033171 |
| BCA_0004 | 6 | 6 | 0.81 | 20138014; 19465649; 19017635; 19017273; 18644471; 18585391; 4612007; 11580844; 17609212; 17600070 |
| BCA_0006 | 6 | 6 | 0.88 | 20844218; 20833633; 20817842; 20675723; 20586870; 20383017; 20361665; 20360860; 20356737; 20348315 |
| BCA_0014 | 14 | 14 | 0.91 | 20832515; 20823538; 20817636; 20808934; 20649757; 20571059; 20524113; 20502051; 20493773; 20491506 |
| BCA_0021 | 14 | 6 | 0.61 | 18331355; 16712859; 16503632; 10481906; 12882517; 10998336; 10364165; 5432063; 9852075; 1317076 |
| BCA_0061 | 13 | 13 | 0.99 | 20598281; 20197408; 20234387; 15980571; 19463886; 18006386; 12716898; 18391411; 18342886; 16873721 |
| BCA_0065 | 19 | 19 | 0.89 | 20528914; 20388664; 20236040; 20200066; 20178374; 20174687; 20039932; 19941984; 19898977; 19880511 |
| BCA_0070 | 4 | 4 | 0.93 | 20644139; 19635793; 18832310; 10792716; 10217758; 15752189; 16005287; 15659160; 12940998; 11994149 |
| BCA_0079 | 12 | 12 | 0.89 | 19416363; 10504222; 17172676; 16432477; 16382698; 15941715; 15597150; 15211796; 10919396; 11137133 |
| BCA_0080 | 1 | 1 | 0.54 | 20826746; 20825409; 20825408; 20811048; 20803137; 20739303; 20720171; 20720017; 20716528; 20714488 |
| BCA_0083 | 2 | 2 | 0.95 | 20054118; 19389784; 365459; 2515994; 15500462; 1556094; 8096767; 1637823; 3117785; 2139795 |
| BCA_0085 | 2 | 2 | 0.94 | 20085893; 19395485; 18093090; 2862841; 17388809; 16781731; 17176045; 17017801; 16781731; 16471696 |
| BCA_0086 | 2 | 2 | 0.82 | 20550915; 20445263; 19924845; 19631695; 19191740; 18804704; 18007032; 17362087; 17289662; 17029412 |
| BCA_0115 | 2 | 2 | 0.97 | 20627615; 20189102; 19728152; 9026; 19021881; 18997428; 18923920; 11829504; 15299926; 17223221 |
| BCA_0116 | 13 | 13 | 0.99 | 20829344; 20617848; 20606262; 20601684; 20541532; 20499650; 20498012; 20479876; 20457752; 20306515 |
| BCA_0123 | 12 | 12 | 0.52 | 15007058; 10369667; 8951810; 10066835; 8197122; 10207046; 1825804; 9668058; 8951810; 8548804 |
| BCA_0124 | 17 | 17 | 0.96 | 20602357; 19801412; 19500594; 18280161; 10383769; 16631197; 11029421; 12029062; 14712722; 12600194 |
| BCA_0128 | 13 | 13 | 0.98 | 20399793; 9202191; 15989950; 10805779; 10747797; 8123703; 1764524; 6360687; 7049235; 6125208 |
| BCA_0133 | 13 | 13 | 0.31 | 15061883; 10377396; 10880769; 8586415; 1916825; 1756182; 2046660; 2874494; 2596824; 2963699 |
| BCA_0134 | 13 | 13 | 0.97 | 15995195; 18726268; 10811627; 11014182; 17512991; 15009191; 14729335; 11863397; 8529646; 2447957 |
| BCA_0136 | 13 | 13 | 0.87 | 20561528; 20338254; 20219471; 20215430; 20132446; 20033061; 20025795; 19965869; 19837086; 19833922 |
| BCA_0137 | 13 | 13 | 0.98 | 20802504; 20573490; 20534494; 20526830; 20522549; 20435138; 20348441; 20215430; 20200150; 20156451 |
| BCA_0138 | 13 | 13 | 0.98 | 20413480; 19111651; 10937989; 7516168; 6093096 |
| BCA_0140 | 13 | 13 | 0.98 | 20065060; 19752277; 16318913; 9660932; 15127365; 12946348; 11296296; 11511371; 10361087; 2036410 |
| BCA_0141 | 13 | 13 | 0.98 | 12670677; 15308339; 9695947; 12581648; 11716492; 8264530; 8457554; 1499563; 920942; 2191716 |
| BCA_0142 | 13 | 13 | 0.98 | 20727857; 20429542; 20097853; 11478805; 8722025; 6345791; 3122848; 4076177 |
| BCA_0148 | 13 | 13 | 0.98 | 10561594; 7916699; 1772592; 2438658; 3926498 |
| BCA_0150 | 13 | 13 | 0.98 | 18198843; 16861792; 11470155; 10361087; 2461520; 2461735; 3526091 |
| BCA_0163 | 13 | 13 | 0.97 | 10937989; 11168885; 8722036; 8444837; 2829909 |
| BCA_0164 | 13 | 13 | 1.00 | 20188109; 19469554; 1547498; 10411137; 16076220; 15680962; 10890005; 12429060; 10937989; 12473202 |
| BCA_0170 | 18 | 18 | 0.33 | 20598325; 20526721; 20523339; 20519162; 20426662; 20382261; 20381026; 20367639; 20353339; 20232316 |
| BCA_0173 | 13 | 13 | 0.98 | 19469554; 12787353; 2036410; 7529559; 2037044; 920942; 2665813; 2653827; 6209544; 6750359 |
| BCA_0174 | 13 | 13 | 0.97 | 19013179; 14675435; 8536310; 8223574; 1812070; 2438658; 2997731; 2429836; 6374660 |
| BCA_0178 | 4 | 4 | 0.79 | 19332816; 7523829; 10618233; 10376819; 9846746 |
| BCA_0199 | 19 | 4 | 0.29 | 19574653; 19572887; 19552711; 8550580; 18842355; 18462386; 18372071; 18266853; 16932074; 15720398 |
| BCA_0202 | 11 | 11 | 0.93 | 19225552; 17451650; 17257169; 15164716; 10613891; 10356325; 1364113; 9349711; 8384292; 2561072 |
| BCA_0217 | 18 | 18 | 0.99 | 20662775; 20408914; 20154136; 20132828; 19760129; 19715704; 19416927; 19254027; 15673787; 1926332 |
| BCA_0229 | 18 | 18 | 0.98 | 20808924; 20662775; 20655877; 20601473; 20584751; 20583551; 20557839; 20504240; 20503931; 20404332 |
| BCA_0233 | 18 | 18 | 0.96 | 20382765; 19801540; 19388077; 18273827; 18273810; 11050157; 18007034; 17600541; 11027822; 16636888 |
| BCA_0238 | 18 | 18 | 0.86 | 19594831; 18312393; 11867227; 7512872; 1733937 |
| BCA_0239 | 18 | 18 | 0.85 | 20382765; 15231816; 9579077; 14568145; 8051706 |
| BCA_0243 | 1 | 1 | 0.22 | 19954230; 19576563; 17185548; 12515465; 10037775; 7565611; 9085573; 9003320; 8621661; 7022140 |
| BCA_0249 | 2 | 2 | 0.95 | 20677756; 17425797; 19415239; 19368556; 18310429; 18607082; 2649979; 11602359; 16936034; 13412744 |
| BCA_0253 | 9 | 9 | 0.37 | 17726007; 16243729; 1751451; 7579178; 7836409 |
| BCA_0286 | 6 | 6 | 0.50 | 20100054; 19152795; 17951115; 17937906; 12223530; 12531030; 11738941; 10924509; 10835277; 10801329 |
| BCA_0287 | 9 | 9 | 0.91 | 20136099; 20121951; 20069378; 20064615; 20013982; 19954230; 19933275; 19797355; 19733180; 19636964 |
| BCA_0319 | 12 | 12 | 0.92 | 20482888; 20194785; 19916930; 19538153; 19490017; 19304828; 19222581; 18419773; 18950664; 18817188 |
| BCA_0340 | 8 | 8 | 0.77 | 18540088; 18445036; 17541831; 17462022; 17404384; 16386381; 11823219; 15221451; 1664907; 10961455 |
| BCA_0364 | 14 | 14 | 0.96 | 20693687; 20675450; 20674424; 20177786; 20175147; 20127976; 20054783; 20049533; 20005278; 20002573 |
| BCA_0368 | 14 | 8 | 0.44 | 16865708; 16544324; 1398079; 8224889; 1245197 |
| BCA_0369 | 14 | 14 | 0.99 | 19935919; 19921932; 19900465; 19575694; 18392760; 9631663; 17686772; 10593947; 17434429; 16755134 |
| BCA_0373 | 14 | 14 | 0.99 | 19946821; 19301155; 18388293; 17888004; 17146619; 15962527; 12629687; 11733076; 10449718; 10079286 |
| BCA_0379 | 6 | 6 | 0.28 | 20624965; 20443037; 20354588; 20214650; 20081198; 20021668; 19923722; 19897762; 19538130; 19150981 |
| BCA_0390 | 18 | 18 | 0.26 | 20103563; 11879800; 10490454; 3193023; 7961673 |
| BCA_0405 | 18 | 8 | 0.21 | 15870078; 15678184; 14668133; 12794928; 10087507; 8853292; 8027026 |
| BCA_0441 | 8 | 8 | 0.69 | 19464996; 10968624; 10460161; 8522507; 8910356; 8394124 |
| BCA_0454 | 2 | 2 | 0.88 | 19888457; 19851002; 18163882; 17384969; 17064688; 16871614; 12906818; 10891066; 10075431; 8885414 |
| BCA_0456 | 18 | 18 | 0.30 | 3327753; 792395; 9826185; 8455557; 1512189; 2987841 |
| BCA_0465 | 19 | 19 | 0.92 | 20843785; 20817800; 20689953; 20687339; 20674735; 20668917; 20667742; 20625731; 20591071; 20565973 |
| BCA_0477 | 13 | 13 | 0.97 | 20829344; 20479876; 19647514; 19580983; 19535251; 19351057; 17459442; 10654942; 1109585; 18406320 |
| BCA_0480 | 4 | 4 | 0.25 | 10203839; 16907731; 10196166; 10594830; 1409590; 353030 |
| BCA_0491 | 18 | 18 | 0.92 | 20809990; 19883117; 19207208; 9419228; 10649632; 17107946; 16754859; 16645309; 16616607; 12848823 |
| BCA_0514 | 1 | 19 | 0.46 | 20682705; 20675574; 20585571; 20573705; 20543557; 20525238; 20503316; 20493879; 20235147; 20233305 |
| BCA_0528 | 8 | 8 | 0.85 | 20338254; 20167619; 20118372; 20012884; 19847785; 19752030; 19703579; 19617386; 19471981; 19401227 |
| BCA_0547 | 18 | 18 | 0.95 | 20843810; 20826817; 20799350; 20683952; 20665261; 20664073; 20659465; 20651349; 20651344; 20644544 |
| BCA_0550 | 2 | 2 | 0.92 | 19786580; 17504214; 10399019; 16972865; 8906967; 16922603; 2511063; 16564539; 9251002; 8155881 |
| BCA_0556 | 15 | 15 | 0.97 | 20643656; 20055984; 20008066; 20002188; 19828451; 19690170; 18587152; 19508286; 19930151; 19169435 |
| BCA_0591 | 12 | 12 | 0.92 | 8941759; 7706536; 1934064; 1324962; 1657382; 1653238; 1646048; 1650534; 1848860; 1654139 |
| BCA_0601 | 15 | 12 | 0.67 | 20724523; 20552019; 20305656; 20181665; 20001590; 19915022; 8254721; 19435816; 19337320; 18991611 |
| BCA_0610 | 8 | 8 | 0.94 | 20705129; 20127467; 19777264; 19545523; 19523599; 19520672; 19405028; 19117946; 19110265; 18952156 |
| BCA_0627 | 15 | 15 | 0.72 | 20110293; 19170879; 17275217; 17268768; 11284699; 16941243; 15966722; 15870478; 15702929; 1311296 |
| BCA_0660 | 9 | 9 | 0.97 | 20086012; 20025994; 19898564; 19850005; 19439403; 19341704; 19001357; 18691575; 17472630; 17564601 |
| BCA_0671 | 15 | 15 | 0.95 | 19420703; 8181761; 18194340; 9000055; 17227471; 11722744; 12690416; 12471443; 10589719; 7961402 |
| BCA_0679 | 18 | 18 | 0.99 | 20808924; 20553499; 20552428; 20548943; 20548793; 20515952; 20471400; 20460823; 20454684; 20439763 |
| BCA_0687 | 2 | 8 | 0.58 | 19946345; 18927392; 18825778; 18804693; 18619818; 17510060; 17302434; 17168321; 17017850; 16842150 |
| BCA_0704 | 18 | 6 | 0.36 | 20631063; 20619549; 20455578; 20145144; 19933046; 19655414; 19633083; 19270722; 19124646; 19118356 |
| BCA_0708 | 8 | 8 | 0.87 | 20600873; 20148428; 17700540; 18801356; 10357895; 17221198; 16570322; 9883893; 10376593; 10617191 |
| BCA_0743 | 8 | 8 | 0.42 | 19818829; 16390180; 11856837; 10684608; 10525269; 8394326; 1825788 |
| BCA_0745 | 18 | 18 | 0.87 | 20797386; 18675571; 9245810; 11902719; 8031825 |
| BCA_0752 | 18 | 18 | 0.98 | 20599727; 20333304; 20152905; 20102440; 19696110; 20592808; 17042492; 10406099; 12925133; 18639631 |
| BCA_0754 | 18 | 18 | 0.85 | 18041902; 19389779; 19362642; 18356573; 8125120; 17074913; 16791740; 16780565; 16705021; 16487324 |
| BCA_0795 | 2 | 2 | 0.97 | 19923213; 19216519; 17403671; 15914018; 15489164; 15012138; 14757766; 14567704; 12650933; 8265357 |
| BCA_0803 | 18 | 18 | 0.93 | 20801885; 19962432; 19458722; 18957371; 18693246; 18627870; 12651848; 18349697; 18310123; 18303017 |
| BCA_0805 | 18 | 18 | 0.91 | 18957371; 16953591; 16412439; 15300779; 12763852; 12546731; 12482133; 11956130; 11585827; 11412108 |
| BCA_0811 | 18 | 18 | 0.91 | 20843810; 20826817; 20808924; 20799350; 20737137; 20726333; 20691148; 20684594; 20683952; 20682982 |
| BCA_0819 | 8 | 8 | 0.93 | 20841500; 20817397; 20800309; 20511298; 20493950; 20350314; 19756991; 19726178; 19633866; 19505469 |
| BCA_0820 | 19 | 19 | 0.51 | 20805025; 20711572; 20680564; 20679207; 20435476; 20427415; 20416079; 20400534; 20398236; 20391781 |
| BCA_0836 | 8 | 8 | 0.98 | 20845078; 20834161; 20833784; 20823222; 20810270; 20808932; 20808820; 20795369; 20739172; 20730761 |
| BCA_0871 | 6 | 6 | 0.26 | 20848273; 20844935; 20838331; 20837752; 20834227; 20822493; 20819415; 20818492; 20817590; 20815142 |
| BCA_0887 | 11 | 11 | 0.99 | 20625149; 20615441; 20562304; 20554775; 20553579; 20547379; 20543074; 20512353; 20502527; 20418401 |
| BCA_0899 | 18 | 18 | 0.81 | 20582607; 20382765; 19388077; 19300437; 18824545; 14761996; 18273827; 18273810; 18226303; 11050157 |
| BCA_0903 | 4 | 4 | 0.77 | 20848291; 20847055; 20846025; 20845670; 20843549; 20843149; 20843110; 20839909; 20839391; 20839055 |
| BCA_0907 | 19 | 19 | 0.19 | 20634968; 20135034; 19772853; 19552402; 19433553; 19309142; 19227983; 19097892; 18947997; 18757813 |
| BCA_0909 | 8 | 8 | 0.94 | 20824484; 20727989; 20680264; 20385943; 20237941; 20197491; 20087965; 20054117; 19940145; 19926682 |
| BCA_0915 | 4 | 4 | 0.87 | 19930460; 19837800; 19767470; 19659696; 19616612; 7783636; 19542328; 19536364; 18203828; 18506440 |
| BCA_0923 | 8 | 1 | 0.65 | 20504042; 20225259; 20218719; 20213636; 20107768; 20081005; 20043233; 20019084; 20012552; 19922622 |
| BCA_0924 | 8 | 8 | 0.79 | 20625734; 20213636; 18574730; 18563659; 18271275; 17558661; 17418311; 16237012; 10559153; 17005012 |
| BCA_0925 | 6 | 6 | 0.56 | 10406811; 10777493; 8016081; 10606815; 8589517; 2005641; 7040984 |
| BCA_0940 | 3 | 3 | 1.00 | 19915677; 18395220; 18166249; 17005012; 16755617; 16532637; 9835590; 8622910; 15474715; 15149039 |
| BCA_0952 | 8 | 8 | 0.38 | 17971860; 9473028; 16164548; 12846584; 12735293 |
| BCA_0981 | 6 | 6 | 0.96 | 20716382; 20675469; 20484375; 20130679; 19614620; 19063859; 19053250; 19001846; 16930479; 18366438 |
| BCA_1118 | 2 | 8 | 0.33 | 20553007; 20517178; 20506607; 20479301; 20331820; 20163457; 20110066; 20099833; 19948245; 19678695 |
| BCA_1120 | 2 | 8 | 0.46 | 20823222; 20814629; 20603160; 20543190; 20484676; 20417053; 20399914; 20375109; 20331452; 20194361 |
| BCA_1143 | 9 | 9 | 0.88 | 11971133; 12454267; 10828604; 9892232; 9804328; 9748261; 9165098; 7668351; 8441459 |
| BCA_1172 | 8 | 8 | 0.89 | 20800605; 20799687; 20736170; 20716528; 20709892; 20705362; 20705129; 20653971; 20645041; 20625650 |
| BCA_1182 | 6 | 6 | 0.91 | 17202163; 18753784; 11171974; 17666433; 16887145; 9461602; 8521502; 11178902; 10878253; 10669596 |
| BCA_1199 | 18 | 18 | 0.94 | 20815391; 20709853; 20684227; 20674437; 20639578; 20627949; 20610766; 20609358; 20606288; 20599686 |
| BCA_1217 | 9 | 9 | 0.98 | 20304657; 20221630; 19968566; 19884012; 19863661; 19836235; 19665020; 19664929; 19595597; 19407376 |
| BCA_1237 | 12 | 12 | 0.89 | 10794418; 7674943; 6337992; 7489923; 7674946; 7828908; 7798200 |
| BCA_1261 | 9 | 9 | 0.99 | 20818586; 20800575; 20727773; 20696503; 20583783; 20533312; 20519910; 20503440; 20410062; 20335945 |
| BCA_1277 | 1 | 1 | 0.32 | 20799944; 20532401; 20359225; 20170126; 20079748; 19943163; 19895578; 19760262; 10688190; 19694421 |
| BCA_1280 | 1 | 8 | 0.57 | 20571952; 19364491; 19032598; 18844775; 18838123; 18514538; 10535953; 17938992; 17927213; 17924454 |
| BCA_1297 | 8 | 8 | 0.93 | 20844759; 20625734; 18675788; 17367808; 17157320; 16263718; 16045627; 15279406; 15213808; 14638692 |
| BCA_1328 | 19 | 19 | 0.86 | 17588176; 17244817; 16476725; 16162506; 10727942; 8380148; 15130128; 14617152; 10471558; 11171944 |
| BCA_1333 | 15 | 15 | 0.95 | 20008174; 19898538; 16430695; 15634675; 11194879; 15165250; 15104138; 12437215; 11849533; 9712687 |
| BCA_1355 | 8 | 8 | 0.87 | 20502966; 20498089; 20035711; 19727950; 19442249; 19292455; 18959769; 18957412; 18600049; 18510925 |
| BCA_1362 | 18 | 18 | 0.86 | 19220474; 7968511; 11700350; 9720051; 8412700 |
| BCA_1368 | 9 | 8 | 0.28 | 20729355; 20214931; 20082371; 19937727; 19937726; 19879956; 19699761; 14693546; 19648370; 19582448 |
| BCA_1415 | 15 | 15 | 0.76 | 20023111; 19000822; 9393699; 12576576; 11972779; 9636707; 8001132; 1987146; 2509430 |
| BCA_1430 | 8 | 8 | 0.71 | 20831589; 20731381; 20628895; 20624490; 20613792; 20601217; 20553732; 20457259; 20419722; 20417637 |
| BCA_1451 | 1 | 1 | 0.95 | 20435740; 20065117; 19923721; 19858196; 19568767; 19405093; 19350404; 19253050; 19157014; 10750896 |
| BCA_1452 | 1 | 1 | 0.55 | 20081005; 18784913; 340888; 10338493; 11243831; 10581183; 9581571; 3907697 |
| BCA_1453 | 1 | 1 | 0.58 | 20081005; 17624809; 10188206; 16795146; 16458324; 9696751; 12488095; 12060231; 11243831; 10942289 |
| BCA_1454 | 1 | 1 | 0.79 | 20028400; 19653643; 19382143; 19362563; 19041906; 18997402; 18763167; 18379776; 12389038; 17512731 |
| BCA_1456 | 1 | 8 | 0.35 | 20735360; 20516614; 20344951; 20008079; 19897891; 19833522; 19527660; 19302372; 18067871; 19154118 |
| BCA_1460 | 1 | 14 | 0.93 | 20696207; 20633228; 20497496; 20299287; 20103674; 19936064; 19887595; 19666527; 19661458; 19486323 |
| BCA_1466 | 1 | 8 | 0.33 | 10673437; 15740738; 11006846; 8905231; 7494587; 14190241 |
| BCA_1475 | 1 | 1 | 0.90 | 20381632; 19543810; 18508763; 18470486; 17626020; 16997906; 12429091; 12570844; 16483680; 15652176 |
| BCA_1479 | 5 | 5 | 0.95 | 20382111; 20042022; 19889875; 19797606; 19770499; 19730970; 19664586; 19474428; 19420695; 19369399 |
| BCA_1480 | 5 | 5 | 0.97 | 20042022; 19903478; 19770499; 19664586; 19304933; 19067028; 18689458; 17540769; 17095009; 16579463 |
| BCA_1486 | 18 | 18 | 0.99 | 20069550; 19785001; 19531598; 19379732; 19039703; 18614623; 18311924; 18251464; 18247577; 10508723 |
| BCA_1506 | 18 | 18 | 0.63 | 20511233; 20459315; 19948126; 19850002; 19627146; 19329431; 18804465; 18793176; 18522545; 11696555 |
| BCA_1512 | 19 | 19 | 0.97 | 20847002; 20846827; 20846501; 20843347; 20836402; 20805396; 20801168; 20733036; 20729361; 20726894 |
| BCA_1520 | 14 | 14 | 0.94 | 20817362; 20810304; 20799866; 20718423; 20695479; 20603113; 20572921; 20570524; 20547356; 20544539 |
| BCA_1521 | 11 | 11 | 0.47 | 20547379; 20085626; 19936201; 11136468; 16209952; 9987107; 15500249; 7854121; 10838584; 8531886 |
| BCA_1532 | 8 | 8 | 0.87 | 20050916; 19682263; 16825793; 10844653; 10386372; 9395519 |
| BCA_1544 | 6 | 6 | 0.92 | 20451470; 20299287; 19177149; 14643435; 18588880; 10373438; 18419580; 10915769; 17911100; 11459957 |
| BCA_1550 | 1 | 8 | 0.79 | 20121452; 19956936; 19711072; 19425107; 19019160; 18546150; 17590228; 15780999; 15574419; 11989713 |
| BCA_1556 | 14 | 14 | 0.95 | 19765547; 19524543; 17542990; 4152559; 18602116; 18556440; 15450940; 18343960; 15247097; 18080217 |
| BCA_1573 | 2 | 2 | 0.95 | 10880976; 10545188; 9756625; 9748348; 7629164; 6413254 |
| BCA_1574 | 14 | 14 | 0.71 | 20801890; 20127467; 20098737; 19843229; 19638341; 19446023; 19405028; 19331130; 18625239; 18534579 |
| BCA_1575 | 1 | 8 | 0.32 | 19826808; 19323044; 19275563; 19245325; 18633449; 10517722; 18279385; 17662045; 17514677; 17348836 |
| BCA_1576 | 1 | 1 | 0.60 | 19650882; 19052366; 18503755; 18062262; 17827659; 2003924; 17451239; 17010158; 16921527; 16675503 |
| BCA_1594 | 19 | 19 | 0.95 | 18952861; 16218944; 18499663; 18325534; 18210176; 16829524; 16794327; 15820665; 15552059; 15226299 |
| BCA_1599 | 2 | 2 | 0.73 | 20568730; 20232910; 20059543; 19827080; 19780086; 19666462; 19266201; 18821554; 18726075; 18422645 |
| BCA_1600 | 2 | 1 | 0.69 | 19609288; 19366687; 19353301; 18726075; 17001646; 16525887; 15184017; 15033515; 14675432; 8197456 |
| BCA_1605 | 6 | 6 | 0.91 | 20587500; 20406289; 20071750; 19968790; 10540288; 18542861; 18703019; 18506095; 18291414; 10754552 |
| BCA_1606 | 6 | 6 | 0.95 | 20798685; 20717695; 20713509; 20695229; 20673527; 20667510; 20659169; 20656798; 20648755; 20628570 |
| BCA_1623 | 12 | 19 | 0.69 | 16717414; 1554361; 942051; 9927582; 9878520; 9747708; 7765282; 3479979; 1369078; 1597179 |
| BCA_1653 | 17 | 17 | 0.52 | 20829018; 20822667; 20805464; 20798173; 20738147; 20733040; 20731347; 20716533; 20713171; 20673218 |
| BCA_1691 | 4 | 4 | 0.88 | 20832320; 20808885; 20543066; 20525091; 20492688; 20418388; 20398208; 20355710; 20345663; 20208450 |
| BCA_1692 | 4 | 4 | 0.66 | 20180908; 10672174; 16677309; 9161425; 8627625; 8308888 |
| BCA_1695 | 4 | 4 | 0.61 | 19705835; 19606502; 19324687; 19231145; 10466731; 15496464; 10468569; 10692300; 17163981; 16413221 |
| BCA_1705 | 4 | 4 | 0.65 | 15136044; 12711330; 8943245; 3313394; 1905667; 2181149; 387724; 2129540 |
| BCA_1714 | 4 | 4 | 0.78 | 18719175; 17768253; 8943245; 2230247; 9286988; 1339421 |
| BCA_1717 | 4 | 4 | 0.88 | 20832320; 20207758; 20025667; 19494578; 19332820; 18774298; 15669087; 7603411; 16207913; 15100991 |
| BCA_1728 | 4 | 4 | 0.86 | 19703110; 16023758; 10940035; 11580247; 14617189; 12732970; 9286988; 9426140; 1551848; 2982790 |
| BCA_1729 | 4 | 4 | 0.69 | 19332819; 9426140; 10940035; 14617189; 9286988 |
| BCA_1789 | 8 | 8 | 0.92 | 20699282; 20696932; 20696004; 20691785; 20682242; 20681784; 20664735; 20621065; 20620191; 20609916 |
| BCA_1793 | 8 | 1 | 0.44 | 19921396; 18546150; 4957395; 17254537; 15830131; 12821154; 12705610; 12596860; 10601247; 9161703 |
| BCA_1806 | 18 | 18 | 0.72 | 20634426; 18761711; 17272682; 10727511; 11284199; 14715671; 9245810; 11055990; 11738647; 11454446 |
| BCA_1853 | 12 | 1 | 0.36 | 20846495; 20826268; 20815781; 20703240; 20655917; 20643759; 20634897; 20605785; 20584906; 20566694 |
| BCA_1859 | 1 | 1 | 0.95 | 20823121; 17194899; 16567425; 12684110; 8117072; 8663056; 8663055; 7771772; 7648198; 7630323 |
| BCA_1868 | 4 | 4 | 0.88 | 20848150; 20847381; 20846937; 20846935; 20846813; 20846811; 20846807; 20846357; 20826325; 20826324 |
| BCA_1877 | 2 | 4 | 0.25 | 19854891; 18973781; 18602927; 18020947; 12927541; 8806782 |
| BCA_1920 | 19 | 12 | 0.30 | 20847180; 20846183; 20845510; 20844469; 20843177; 20841584; 20835724; 20818814; 20817642; 20816009 |
| BCA_1947 | 18 | 18 | 0.74 | 20807201; 20541816; 20441230; 20228245; 20181755; 17173478; 19169648; 18663607; 18349501; 18243325 |
| BCA_1950 | 2 | 2 | 0.95 | 20180265; 19636932; 18391442; 17305325; 14466980; 17229734; 16884311; 16042595; 15966718; 14503879 |
| BCA_1961 | 18 | 14 | 0.39 | 3189820; 15870078; 15678184; 15012136; 14668133; 12794928; 7638212; 1593632; 10087507; 2197994 |
| BCA_1962 | 14 | 14 | 0.38 | 20208582; 19811548; 18347391; 18052321; 16511035; 17681069; 17629045; 9817849; 17108722; 7532308 |
| BCA_1974 | 18 | 18 | 0.91 | 19653651; 18355966; 17632081; 17171805; 12851823; 11756511; 10828993 |
| BCA_2001 | 18 | 18 | 0.96 | 19919002; 19717637; 19608612; 19597156; 19470745; 19434502; 15200952; 14576852; 11017087; 17711302 |
| BCA_2006 | 8 | 8 | 0.68 | 19923750; 10718582; 15064768; 14597104; 9582432; 2394679; 1103739; 5916391; 5916393; 7391024 |
| BCA_2031 | 1 | 1 | 0.95 | 20305003; 20305002; 19877050; 19761441; 17698629; 18621388; 18300225; 18272376; 18054772; 17979829 |
| BCA_2032 | 1 | 1 | 0.83 | 20439910; 20305003; 20305002; 19622802; 19531469; 19509296; 19509290; 17698629; 18460806; 17624493 |
| BCA_2041 | 4 | 4 | 0.57 | 19911130; 19267410; 18397761; 1706704; 17462011; 17352426; 16925552; 9387222; 16420347; 16313622 |
| BCA_2060 | 2 | 2 | 0.67 | 17642516; 15748981; 11375500; 8761662; 7980520 |
| BCA_2081 | 15 | 15 | 0.49 | 20382764; 18342372; 10698627; 12738850; 11286503; 8957002; 7796533 |
| BCA_2135 | 12 | 12 | 0.84 | 2860849; 18462752; 17372352; 15856219; 13129613; 10536007; 10567266; 1628824; 10658654; 10556070 |
| BCA_2148 | 2 | 8 | 0.41 | 20822113; 20728927; 20471397; 19947554; 19942660; 19641494; 19569646; 19558965; 19240958; 19049514 |
| BCA_2152 | 4 | 4 | 0.88 | 20465279; 20207735; 20131326; 20118189; 20026072; 19850038; 19706531; 19647889; 17332417; 19371779 |
| BCA_2155 | 8 | 8 | 0.68 | 17038198; 17497880; 4690969; 10503541; 10080921; 9611813; 4352175; 9073078; 4590480; 7496535 |
| BCA_2208 | 12 | 12 | 0.70 | 19467604; 19417147; 19239642; 19225051; 18791760; 18309355; 17959197; 17627939; 17475206; 2538661 |
| BCA_2210 | 18 | 2 | 0.87 | 20798996; 20417532; 20414806; 20223208; 20223218; 19816401; 19646414; 19640975; 15725058; 11414806 |
| BCA_2214 | 8 | 8 | 0.80 | 20116460; 19959582; 19702085; 19397750; 10896219; 19060066; 11331606; 18298089; 16791644; 15371444 |
| BCA_2216 | 8 | 8 | 0.81 | 11331606; 18298089; 15371444; 12576596; 12527376; 12165429; 12121455; 10542130; 9328645; 9305880 |
| BCA_2217 | 15 | 15 | 0.94 | 20661434; 18699868; 18672305; 9283073; 18573287; 17133823; 9952369; 15882420; 16553815; 7618865 |
| BCA_2228 | 2 | 8 | 0.55 | 19754882; 11590235; 1435259; 9493381; 7959054; 8123787 |
| BCA_2229 | 5 | 8 | 0.75 | 19387485; 16491308; 11939777; 9022686; 1830217 |
| BCA_2268 | 12 | 12 | 0.98 | 16740178; 16596389; 16442309; 10834845; 15268897; 14517614; 9989817; 11583934; 10888351; 10620182 |
| BCA_2321 | 14 | 14 | 0.58 | 20837458; 20819423; 20815815; 20811661; 20798559; 20738018; 20727673; 20725619; 20723540; 20714877 |
| BCA_2322 | 2 | 8 | 0.27 | 20845390; 20834190; 20816845; 20810806; 20799995; 20795731; 20737003; 20730777; 20726028; 20724119 |
| BCA_2328 | 12 | 12 | 0.87 | 20630200; 19395490; 19383693; 16698798; 16678787; 15378526; 12867413; 11007775 |
| BCA_2339 | 8 | 8 | 0.64 | 20564560; 20192272; 19860415; 18655158; 18318836; 16828464; 16816893; 16790434; 15046574; 16132097 |
| BCA_2346 | 18 | 18 | 0.96 | 20836083; 20696370; 19950859; 20173076; 17080616; 19462180; 18684841; 18637520; 18614208; 18485707 |
| BCA_2353 | 12 | 8 | 0.21 | 16232749; 15228527; 14612229; 10727942; 8939979 |
| BCA_2361 | 18 | 18 | 0.92 | 6148337; 17996893; 17986083; 12200319; 11481430; 16645306; 16445940; 16225868; 12003950; 15469514 |
| BCA_2419 | 8 | 8 | 0.40 | 19525419; 18048912; 16879647; 16689789; 16342930; 14575713; 15723538; 14575713; 7608087; 12706720 |
| BCA_2423 | 8 | 8 | 0.77 | 16292556; 10947204; 10686279; 9787093; 9590057; 9590035; 773366; 7609453; 3117077; 6418146 |
| BCA_2442 | 12 | 9 | 0.75 | 20038713; 19777301; 19733180; 17237222; 15990252; 15719355; 12664133; 12127488; 9520265; 8939709 |
| BCA_2462 | 18 | 18 | 0.99 | 19622348; 18983828; 18273827; 18273810; 11050157; 18204073; 17725565; 17267090; 16636888; 16024601 |
| BCA_2515 | 4 | 15 | 0.54 | 20380929; 19167902; 19153821; 2483028; 18765922; 2862841; 18051762; 17095013; 7429637; 15670150 |
| BCA_2565 | 11 | 11 | 0.98 | 20056615; 19706522; 19347993; 18775728; 8608932; 2991569; 17428497; 16618107; 10913080; 16377618 |
| BCA_2571 | 4 | 8 | 0.22 | 20138146; 16990790; 18667851; 18248455; 17952626; 15828683; 16232298; 15869971; 15828683; 2148964 |
| BCA_2580 | 12 | 12 | 0.64 | 20460427; 20082075; 19915793; 19583493; 19577318; 19373489; 19301317; 18790006; 17601991; 17562349 |
| BCA_2601 | 8 | 8 | 0.81 | 20673213; 19405028; 19236002; 19167403; 18803552; 18754683; 18688832; 17882509; 17713928; 17400540 |
| BCA_2666 | 4 | 4 | 0.47 | 20644935; 20556826; 20381010; 20231391; 20202936; 20145089; 20138928; 20099093; 20089863; 20057078 |
| BCA_2718 | 8 | 8 | 0.78 | 20848647; 20848598; 20848331; 20848186; 20848147; 20848083; 20847137; 20846929; 20846786; 20845962 |
| BCA_2742 | 8 | 8 | 0.77 | 20727852; 20547751; 20543567; 20501590; 20423462; 20410293; 20370610; 20302306; 20332039; 20138891 |
| BCA_2793 | 4 | 4 | 0.66 | 20220788; 19917758; 19863785; 12890034; 9139908; 10498733; 11229919; 14651641; 14612242; 10734070 |
| BCA_2811 | 19 | 19 | 0.77 | 20522493; 19735442; 19329633; 17259175; 17516072; 17084392; 9457884; 15153765; 9250845; 10092453 |
| BCA_2816 | 4 | 4 | 0.95 | 20712413; 20308541; 20071153; 20041768; 19928876; 19901089; 19828298; 19783991; 19747006; 19734311 |
| BCA_2851 | 8 | 8 | 0.92 | 20659890; 20405473; 20399281; 20378991; 20307095; 19367707; 19361226; 19166312; 19048328; 18945673 |
| BCA_2872 | 12 | 12 | 0.92 | 19038348; 18992803; 9770452; 17009084; 16669783; 8982462; 16705403; 16682229; 16549666; 11585843 |
| BCA_2875 | 8 | 8 | 0.85 | 20499043; 20155483; 20054114; 19923736; 19744161; 19690365; 19420771; 19328460; 19214439; 19120610 |
| BCA_2886 | 19 | 19 | 0.73 | 20686913; 10600390; 18624637; 17234634; 16122801; 10347049; 11358516; 10777493 |
| BCA_2906 | 12 | 12 | 0.93 | 18593711; 10751435; 11854196; 15299395; 17173283; 7674943; 16342953; 15876371; 15870064; 12885939 |
| BCA_2920 | 8 | 8 | 0.77 | 20675294; 16820168; 16027125; 15946648; 15914915; 10200326; 11870062; 11330998; 7543100; 1939012 |
| BCA_2954 | 4 | 4 | 0.79 | 20082702; 20077512; 19837800; 18249137; 18031270; 10322445; 12637519; 12198312; 11884407; 9515903 |
| BCA_2979 | 2 | 8 | 0.54 | 20848620; 20797618; 20729113; 20721927; 20717690; 20694531; 20629144; 20619503; 20603201; 20576686 |
| BCA_3039 | 18 | 8 | 0.44 | 20735204; 20686672; 20448665; 20420430; 20409457; 20385961; 20354774; 20237115; 20203153; 20003133 |
| BCA_3040 | 9 | 9 | 1.00 | 18060402; 14570270; 12379132; 1396693; 1416617; 7813482; 8001771; 8051146; 8454629; 8490573 |
| BCA_3046 | 18 | 18 | 0.96 | 20145144; 15231816; 15618228; 8031825; 11358842; 10941799; 8878033; 8253677 |
| BCA_3065 | 1 | 8 | 0.83 | 20525824; 20403182; 20223245; 20091669; 19401719; 19954230; 19932104; 19899082; 19850488; 19648921 |
| BCA_3103 | 8 | 8 | 0.75 | 20848588; 20848047; 20846448; 20845078; 20844459; 20844277; 20843565; 20842312; 20840069; 20838591 |
| BCA_3114 | 9 | 8 | 0.47 | 20138891; 20067617; 19825006; 19806925; 19787434; 19756631; 19429603; 18989749; 18987879; 18321191 |
| BCA_3141 | 18 | 1 | 0.30 | 10681503; 141275; 12119036; 2468367; 7551055; 7941748; 8368011; 4583244; 1346091; 3037697 |
| BCA_3171 | 4 | 4 | 0.44 | 20089863; 20057078; 16456645; 19429619; 18637789; 18259066; 18199745; 3015884; 15857293; 15827636 |
| BCA_3190 | 4 | 4 | 0.86 | 18694737; 18251901; 10090757; 12121459; 11169136; 10545213; 9688602; 3516975; 8279538; 8437891 |
| BCA_3226 | 15 | 15 | 0.99 | 20848286; 20834167; 20833814; 20833806; 20833804; 20825354; 20817764; 20817745; 20814030; 20811812 |
| BCA_3228 | 18 | 18 | 0.99 | 20639324; 20466975; 20601473; 20600546; 20578149; 20553499; 20531945; 20481466; 20631346; 20455852 |
| BCA_3239 | 12 | 12 | 0.91 | 14734171; 12183460; 12059959; 12054669; 10574456; 9735342 |
| BCA_3265 | 4 | 18 | 0.51 | 20713732; 20655466; 20594961; 20562876; 20528238; 20479256; 20466852; 20452359; 20413621; 20377457 |
| BCA_3314 | 19 | 19 | 0.96 | 20620870; 20608745; 20128627; 20061535; 19935678; 19646181; 19462216; 19458048; 19209901; 19063962 |
| BCA_3316 | 15 | 15 | 0.61 | 20404817; 10860755; 10731599; 10681342; 15978084; 14702158; 14617162; 14617152; 7854121; 10471558 |
| BCA_3320 | 11 | 11 | 1.00 | 20197135; 19879290; 19821988; 19416360; 15262929; 17028591; 15616333; 10381117; 15268934; 10545172 |
| BCA_3339 | 18 | 12 | 0.31 | 20334581; 20171238; 20159442; 20022921; 20021638; 19825806; 19767149; 19692482; 11779506; 12000790 |
| BCA_3344 | 12 | 8 | 0.65 | 20383020; 10508786; 15504408; 15491154; 15291820; 12586941; 12377778; 12206761; 10781538; 11445168 |
| BCA_3349 | 4 | 18 | 0.96 | 19947854; 18175317; 16862376; 16842214; 2341182; 16008353; 1717452; 15159567; 12823972; 11856849 |
| BCA_3352 | 1 | 8 | 0.90 | 19454243; 19446023; 18378410; 16865707; 17761677; 17693143; 17294332; 15213162; 16458324; 15668249 |
| BCA_3428 | 11 | 11 | 0.95 | 20800080; 20615953; 20603069; 20562304; 20538004; 20521842; 20512402; 20511494; 20508639; 20404201 |
| BCA_3439 | 4 | 18 | 0.77 | 12731863; 11741943; 11717516; 1463743; 1690810; 1544915; 2102832 |
| BCA_3468 | 8 | 8 | 0.89 | 20835425; 20826743; 20816840; 20693662; 20674425; 20673211; 20667822; 20647326; 20623638; 20622431 |
| BCA_3564 | 8 | 8 | 0.80 | 20002588; 19043737; 18937503; 18554861; 10411274; 18154727; 18041955; 17468884; 15181008; 11562374 |
| BCA_3566 | 6 | 6 | 0.89 | 18561205; 18379590; 18033691; 11931763; 15713769; 14871915; 14512394; 11683355; 9234704; 10469597 |
| BCA_3570 | 19 | 8 | 0.48 | 20843040; 20837036; 20835716; 20833759; 20833727; 20833236; 20830595; 20829315; 20824499; 20824389 |
| BCA_3598 | 19 | 18 | 0.51 | 20830571; 20522637; 20507232; 20103707; 20089922; 20086163; 20067831; 19808092; 19776269; 19747529 |
| BCA_3623 | 11 | 11 | 0.97 | 20554775; 20508639; 20403774; 20377427; 20331877; 20167799; 20007331; 19917199; 19901095; 19840380 |
| BCA_3649 | 9 | 9 | 0.96 | 17899070; 17891922; 15708363; 15668256; 12770824; 9388293; 9438344; 8341260; 1547954; 2583128 |
| BCA_3662 | 18 | 18 | 0.81 | 19684063; 19558963; 19542337; 17975082; 15294827; 17602684; 17185539; 17120758; 17071331; 15009894 |
| BCA_3668 | 4 | 4 | 0.86 | 20831249; 20654628; 20618708; 20593183; 20581204; 20570679; 20563625; 20562307; 20556427; 20507060 |
| BCA_3680 | 18 | 18 | 0.83 | 20363944; 19661178; 19646180; 19569551; 18957436; 18701447; 10368146; 18305482; 12813062; 18084014 |
| BCA_3691 | 6 | 6 | 0.96 | 19907650; 19500209; 19478444; 17970226; 17691945; 11089219; 5700707; 16404155; 16321509; 15897198 |
| BCA_3702 | 4 | 4 | 0.82 | 19837800; 19616612; 7783636; 7961468; 15128519; 12010551; 10864493; 10974126; 11207600; 17906142 |
| BCA_3714 | 4 | 4 | 0.65 | 19696109; 17126944; 11884407; 10906212; 10774706; 10766852; 9738899; 9692967; 8081503; 2730871 |
| BCA_3723 | 9 | 9 | 0.99 | 20348430; 20214591; 20214478; 20154153; 19634011; 19391105; 19345228; 19267463; 19218397; 18834333 |
| BCA_3795 | 15 | 15 | 0.97 | 20173059; 18511939; 16118214; 16787930; 8799114; 16132864; 11344136; 11719184; 7687247; 9004222 |
| BCA_3824 | 18 | 18 | 0.61 | 20626894; 20184747; 19663454; 18190179; 17876822; 17599800; 16806239; 16283523; 16091356; 15677459 |
| BCA_3836 | 6 | 6 | 0.93 | 19616486; 19542005; 18992265; 18682218; 8284199; 11106395; 8408062; 12226667; 17201058; 12202775 |
| BCA_3837 | 12 | 12 | 0.48 | 12549933; 11856302; 10930847; 10591406; 271968; 2543955; 6310323; 1099217; 159458; 6336737 |
| BCA_3845 | 19 | 19 | 0.98 | 20644141; 19656295; 19483088; 18081839; 15379577; 17692452; 10564498; 17501913; 17501919; 1347040 |
| BCA_3859 | 19 | 19 | 0.99 | 20708437; 20609359; 20566858; 20414771; 20386924; 20300605; 20173067; 20167623; 20127235; 20118250 |
| BCA_3866 | 6 | 6 | 0.98 | 20833188; 20696460; 20603082; 20567595; 20552889; 20495087; 20495086; 20459533; 20453896; 20438357 |
| BCA_3871 | 8 | 8 | 0.90 | 20618950; 18983854; 12875742; 11243891; 7006387 |
| BCA_3876 | 6 | 6 | 0.93 | 20817622; 20807205; 20798837; 20724091; 20723756; 20722738; 20722443; 20713120; 20711416; 20703307 |
| BCA_3893 | 12 | 12 | 0.97 | 20833629; 20688819; 20661284; 20643392; 20521950; 20462489; 20380838; 20416323; 20413834; 20388215 |
| BCA_3894 | 6 | 17 | 0.44 | 20458164; 20418391; 20025672; 20025665; 19633085; 17376733; 18713320; 9405616; 16105033; 7533264 |
| BCA_3904 | 13 | 13 | 0.97 | 17889642; 17194931; 4912319; 15522293; 14729335; 12823975; 11341947; 10753109; 10742169; 10523206 |
| BCA_3916 | 11 | 17 | 0.60 | 20620949; 20527020; 20487639; 20484498; 20392825; 20353723; 20219933; 20217897; 20215437; 20204192 |
| BCA_3926 | 13 | 13 | 0.99 | 17185548; 17051149; 15459648; 12068815; 2199796; 1386558; 6752941; 7013783 |
| BCA_3927 | 15 | 15 | 0.82 | 20363936; 19801406; 19749041; 19651859; 19302100; 19270096; 19202088; 18223088; 8830686; 17725558 |
| BCA_3932 | 6 | 6 | 0.93 | 20811674; 20576209; 20724443; 20723754; 20687496; 20662543; 20644584; 20638367; 20636260; 20634320 |
| BCA_3935 | 8 | 8 | 0.83 | 20448188; 18392745; 17640871; 11831846; 1986797; 1276977 |
| BCA_3936 | 17 | 6 | 0.22 | 20615256; 20472790; 20131292; 20131297; 20047562; 19923215; 19891952; 19139135; 19034401; 10997908 |
| BCA_3939 | 13 | 13 | 0.98 | 19469554; 9491077; 3290194; 920942; 2665813; 2653827; 6381990; 768740; 773694; 6163479 |
| BCA_3945 | 12 | 12 | 0.45 | 20828566; 20826819; 20804452; 20733058; 20729200; 20714446; 20709425; 20682346; 20682315; 20682283 |
| BCA_3948 | 4 | 4 | 0.69 | 20661436; 20525833; 20498298; 11983169; 19629621; 11017081; 19502785; 19482037; 19450506; 19450506 |
| BCA_3949 | 17 | 17 | 0.27 | 20810747; 20713509; 20705233; 20677811; 20639884; 20620951; 20616011; 20615407; 20603000; 20596726 |
| BCA_3955 | 6 | 6 | 0.94 | 338918; 10899129; 12898065; 8646771; 1544582; 3323532 |
| BCA_3958 | 13 | 13 | 0.98 | 19469554; 9294008; 9044258; 8722036; 8444837; 1742360; 920942; 2665813; 2653827; 3884043 |
| BCA_3966 | 13 | 13 | 0.92 | 19843529; 19695341; 10583945; 11259585; 14638476; 16003488; 15939024; 15731104; 10526160; 14729668 |
| BCA_3970 | 17 | 17 | 0.99 | 20512976; 19963062; 19583999; 19199915; 2886365; 18289874; 9685179; 17822967; 17233676; 15109491 |
| BCA_3971 | 14 | 15 | 0.80 | 20813141; 19941036; 19901021; 19781660; 19706536; 19642226; 19495554; 19406872; 19170836; 18635821 |
| BCA_3985 | 14 | 14 | 0.55 | 20674615; 20603160; 20399851; 20205678; 8925840; 18937438; 18932012; 16600626; 16330000; 16143532 |
| BCA_3986 | 14 | 14 | 0.60 | 19097788; 16624811; 15157083; 10956027; 16561900 |
| BCA_3990 | 14 | 14 | 0.64 | 20620149; 20602244; 20562286; 20451592; 20377130; 20219403; 20210358; 20066562; 20054825; 19860411 |
| BCA_3992 | 18 | 18 | 0.48 | 20681456; 20551056; 20424846; 20154595; 20146171; 20132429; 20042597; 20041985; 20023572; 20002522 |
| BCA_4010 | 4 | 4 | 0.94 | 20825347; 20818414; 20817757; 20807205; 20735775; 20711458; 20692617; 20670397; 20629754; 20625433 |
| BCA_4016 | 19 | 19 | 0.90 | 20024979; 19403924; 19369074; 19198900; 19014883; 19007109; 18704940; 18557704; 18259126; 17427948 |
| BCA_4021 | 4 | 4 | 0.90 | 20807205; 20644139; 20363951; 20352045; 19880597; 19635793; 19429628; 17693520; 16227976; 18832310 |
| BCA_4032 | 2 | 2 | 0.66 | 20486930; 19851003; 11994049; 10329792; 10480925; 6312972; 15642482; 10480925; 14684928; 14684898 |
| BCA_4047 | 8 | 8 | 0.98 | 20700628; 20698788; 20579319; 20525945; 20462199; 20398675; 20381373; 20357218; 20194738; 20091229 |
| BCA_4052 | 8 | 8 | 0.87 | 20838891; 20807508; 20803137; 20799944; 20720299; 20720172; 20693323; 20656883; 20655901; 20630217 |
| BCA_4067 | 5 | 5 | 0.97 | 20735257; 20730478; 20680281; 20680094; 20661733; 20643493; 20560878; 20534592; 20472577; 20470880 |
| BCA_4068 | 4 | 8 | 0.40 | 20832458; 20812289; 20810110; 20799407; 20665636; 20716179; 20684615; 20659425; 20636272; 20624404 |
| BCA_4075 | 8 | 8 | 0.89 | 20180236; 20022530; 19594830; 19464573; 19240034; 19185000; 17667915; 17635929; 10905486; 16517405 |
| BCA_4076 | 8 | 8 | 0.95 | 20160912; 17923481; 17157320; 15082001; 14992577; 14644451; 12761172; 11955070; 10767328; 10656808 |
| BCA_4077 | 8 | 8 | 0.94 | 20160912; 19996100; 16531404; 14644451; 12603319; 11955070; 11935326; 11781147; 11708858; 11673873 |
| BCA_4138 | 8 | 8 | 0.90 | 20808932; 20659552; 20625049; 20620150; 20618950; 20552226; 20516620; 20493821; 20459120; 20435911 |
| BCA_4166 | 5 | 5 | 0.66 | 20837006; 20169067; 19851340; 19700525; 19648376; 19557348; 19507290; 18436239; 18214472; 18186488 |
| BCA_4167 | 5 | 5 | 0.37 | 19648376; 19520724; 11922338; 17567048; 9143339; 16630633; 16568996; 16232619; 15996110; 10411743 |
| BCA_4173 | 12 | 12 | 0.54 | 20368803; 16920705; 16260779; 15084722; 11679083; 11054562; 9434176; 7588713; 7913447; 6238408 |
| BCA_4196 | 18 | 18 | 0.92 | 20519162; 19346249; 17700703; 10748031; 17619822; 16857941; 16595876; 11590105; 10601252; 10225906 |
| BCA_4199 | 14 | 14 | 0.97 | 20606280; 19818317; 18776493; 17804405; 16893570; 16786272; 16519676; 16283293; 16233297; 1412694 |
| BCA_4223 | 2 | 2 | 0.94 | 19622649; 11399071; 7473709; 7622491; 7814407; 8055941; 2115523; 2106516; 2506544; 3502256 |
| BCA_4225 | 2 | 2 | 0.57 | 20465413; 20430628; 19748579; 19716309; 19552377; 19486668; 19117095; 18607092; 18331058; 18298940 |
| BCA_4269 | 8 | 8 | 0.94 | 20675489; 20400973; 20180236; 20160912; 20106611; 20022530; 19874026; 19856494; 19739093; 19735955 |
| BCA_4270 | 8 | 8 | 0.66 | 20655923; 20196890; 17524396; 11839747; 11507102; 11448970; 11069910; 10764784; 10745006; 10644743 |
| BCA_4271 | 8 | 8 | 0.37 | 20655923; 20196890; 20136525; 18026828; 3947057; 17524396; 16861235; 16839657; 16581023; 15712224 |
| BCA_4286 | 2 | 2 | 0.93 | 20808315; 20634351; 20627615; 20591838; 20428043; 20364257; 20349334; 20303132; 20207709; 20188042 |
| BCA_4305 | 13 | 13 | 0.95 | 20823541; 20729861; 20670890; 20227660; 20070887; 19833922; 19542017; 17332744; 17896105; 16842744 |
| BCA_4307 | 1 | 8 | 0.34 | 20445226; 19911771; 19856378; 19816720; 18200648; 10089413; 17487901; 17487900; 17245805; 17068841 |
| BCA_4312 | 15 | 15 | 0.97 | 12668673; 18619468; 18279349; 17724773; 17592720; 12574519; 17209554; 8529874; 15667993; 10497029 |
| BCA_4350 | 4 | 4 | 0.77 | 10361283; 9723928; 6852529; 1971619; 1944223; 1676385 |
| BCA_4353 | 4 | 4 | 0.87 | 8825484; 9987128; 6852529; 1971619; 8825779; 1676385 |
| BCA_4363 | 1 | 1 | 0.87 | 20558164; 20490431; 20310006; 20151607; 20127417; 20086323; 20073048; 20044141; 20036773; 19931232 |
| BCA_4376 | 5 | 2 | 0.33 | 19738041; 19556632; 19452554; 19022216; 18614746; 18586655; 18473156; 18458567; 18452180; 8741798 |
| BCA_4380 | 18 | 18 | 0.97 | 20656779; 20600125; 20443931; 20369870; 20110355; 20083572; 20535133; 20056921; 20018934; 19919544 |
| BCA_4381 | 18 | 18 | 0.97 | 20600125; 20503933; 20443931; 20369870; 20298244; 20221674; 20110355; 20097209; 20083572; 20535133 |
| BCA_4386 | 4 | 4 | 0.93 | 20832270; 20825371; 20814075; 20732897; 20726524; 20720404; 20713653; 20713150; 20702768; 20696273 |
| BCA_4392 | 18 | 18 | 0.99 | 19141712; 18252722; 16714605; 15375207; 16288462; 16922681; 16445940; 4055741; 15817790; 10350474 |
| BCA_4399 | 2 | 2 | 0.54 | 19903472; 19708647; 19558962; 19155093; 16480720; 15650872; 12706830; 11741609 |
| BCA_4404 | 6 | 6 | 0.66 | 20808903; 20699296; 20691900; 20686482; 20686026; 20685648; 20675469; 20649463; 20643958; 20591822 |
| BCA_4424 | 12 | 12 | 0.97 | 20847048; 20803087; 20735358; 20668094; 20648511; 20583963; 20504766; 20499929; 20487289; 20448033 |
| BCA_4427 | 15 | 15 | 0.81 | 15979091; 9714706; 9345313; 7504905; 8045913 |
| BCA_4428 | 2 | 2 | 0.95 | 20194361; 19919179; 17178720; 16606627; 16218869; 15967800; 15450488; 12826405; 12975365; 12114526 |
| BCA_4433 | 13 | 13 | 0.99 | 20600110; 20149799; 20034956; 9848646; 10890005; 11530930; 1961724; 8223574; 7683367; 7916699 |
| BCA_4438 | 4 | 4 | 0.87 | 9987128; 17172014; 16209911; 15458414; 9746358; 9701804; 7565874; 7968523 |
| BCA_4445 | 1 | 1 | 0.89 | 18260104; 16078071; 15596430; 15336409; 12906820; 12906831; 12624088 |
| BCA_4485 | 1 | 1 | 0.91 | 20720017; 20699647; 20658158; 20529854; 20429919; 20379751; 20179139; 19955263; 19953300; 12650624 |
| BCA_4492 | 14 | 12 | 0.48 | 20813095; 20809980; 20798511; 20705669; 20684612; 20652929; 20634336; 20616614; 20602252; 20602229 |
| BCA_4500 | 13 | 13 | 0.99 | 20618922; 20362064; 20179335; 20156976; 20059602; 20045102; 20035439; 20010690; 19903477; 19661429 |
| BCA_4518 | 4 | 18 | 0.22 | 15958381; 12890693; 12080068; 10913128; 10480605; 9585132; 9257921; 7840804; 7918472; 1331759 |
| BCA_4519 | 14 | 14 | 0.47 | 20838591; 20606733; 20558841; 20506278; 20485294; 20392873; 20388721; 20363738; 20347206; 20303634 |
| BCA_4528 | 13 | 13 | 0.96 | 20354154; 20129918; 19925456; 19894214; 19874048; 19746363; 19627989; 19414587; 19199329; 18001138 |
| BCA_4531 | 6 | 6 | 0.93 | 20670908; 20601468; 20304994; 20157002; 19997622; 19997508; 19580006; 19369355; 19072585; 10506828 |
| BCA_4532 | 6 | 6 | 0.92 | 20601468; 19997622; 19997508; 19072585; 8331065; 10464259; 18068124; 17981150; 17919660; 17599913 |
| BCA_4542 | 2 | 8 | 0.46 | 20838591; 20600565; 18959769; 3480514; 18674537; 18226609; 13538931; 10069079; 16040350; 15967443 |
| BCA_4546 | 1 | 1 | 0.89 | 20480196; 20215586; 20137911; 20052993; 19948253; 19776011; 19508381; 19082689; 18662373; 4536832 |
| BCA_4554 | 13 | 13 | 1.00 | 19363482; 16285924; 8411168; 8251501; 1339289 |
| BCA_4566 | 6 | 6 | 0.91 | 19414020; 14617176; 11350954; 10652786; 1328155; 8106460; 7716189; 1374847 |
| BCA_4575 | 2 | 2 | 0.72 | 20798996; 20586119; 20485863; 20038586; 19965637; 19934113; 19805308; 19350426; 19336693; 19289130 |
| BCA_4577 | 2 | 8 | 0.32 | 18680949; 10217486; 9163953; 8012594; 3075378; 3040684 |
| BCA_4582 | 12 | 12 | 0.84 | 20828137; 20825413; 20818167; 20707733; 20664162; 20659109; 20658971; 20636275; 20633278; 20597098 |
| BCA_4596 | 17 | 17 | 0.71 | 19880604; 19617368; 19153445; 18050911; 17391830; 17471261; 17279367; 17173052; 11222749; 16514143 |
| BCA_4597 | 4 | 4 | 0.57 | 20615113; 20543061; 20472691; 20388667; 20222448; 20201549; 20195609; 20081210; 20060611; 20039178 |
| BCA_4607 | 14 | 14 | 0.57 | 20842901; 20836741; 20836050; 20835992; 20833716; 20828826; 20826656; 20826431; 20823761; 20823283 |
| BCA_4617 | 4 | 4 | 0.90 | 20203055; 20178785; 18667558; 18553406; 10667798; 9841668; 15819626; 12867460; 10234819; 11931544 |
| BCA_4618 | 4 | 4 | 0.90 | 20346719; 20203055; 20178785; 19520101; 19361426; 19358329; 18302792; 19202091; 10667798; 15914076 |
| BCA_4623 | 8 | 8 | 0.88 | 20583616; 20551992; 20540712; 20529087; 20379037; 20039900; 20028790; 20000467; 20213850; 19859792 |
| BCA_4628 | 8 | 8 | 0.97 | 20505714; 20421951; 20228121; 20202874; 20059546; 20053769; 20028790; 19768395; 19635800; 19584533 |
| BCA_4647 | 18 | 18 | 0.21 | 20395263; 19109523; 18273684; 17134587; 16930575; 14756421; 14744877; 10559471; 12691374; 12042324 |
| BCA_4666 | 13 | 13 | 0.97 | 20433702; 20353103; 20160120; 18784368; 18611382; 10329163; 15526031; 16939209; 16866361; 15956377 |
| BCA_4667 | 13 | 13 | 0.93 | 20353103; 20223217; 20160120; 19201563; 18611382; 16211138; 18398193; 18048724; 10329163; 17082384 |
| BCA_4683 | 13 | 13 | 0.99 | 20400559; 18037435; 12368106; 8628231; 8722036; 8504167 |
| BCA_4684 | 13 | 13 | 0.99 | 20132446; 15100988; 12068810; 9419243; 8980544; 8725011; 7739034; 8207023; 1875917; 2116407 |
| BCA_4688 | 6 | 6 | 0.91 | 20071750; 16622063; 16002087; 15556628; 1497322; 11679082; 11585815 |
| BCA_4689 | 6 | 4 | 0.16 | 12438417; 17518415; 8350400; 14750441; 11689051; 1541518; 10505416; 10466935; 9558283; 3373568 |
| BCA_4691 | 5 | 5 | 0.80 | 20124698; 20095968; 16515461; 16176926; 15150268; 15096507; 12600205; 12463749; 12403622; 12205086 |
| BCA_4696 | 6 | 6 | 0.84 | 20807462; 20713433; 20709087; 20661660; 20640548; 20640410; 20615203; 20600542; 20566850; 20561949 |
| BCA_4703 | 8 | 8 | 0.69 | 20713124; 20692206; 20661717; 20597986; 20560678; 20513808; 20473773; 20400467; 20353438; 20303774 |
| BCA_4711 | 9 | 9 | 0.99 | 19965770; 10940247; 16212603; 10704200; 10589718; 3040718; 1526981 |
| BCA_4714 | 6 | 6 | 0.83 | 20222445; 20203672; 20195500; 20122408; 19645470; 19636943; 19635595; 19591176; 19551876; 19541616 |
| BCA_4746 | 1 | 1 | 0.53 | 20696000; 20567615; 20544730; 20484304; 20466402; 20459375; 20159990; 20104527; 20067537; 19934275 |
| BCA_4752 | 8 | 8 | 0.31 | 20846475; 20832999; 20826794; 20826149; 20826143; 20826132; 20823576; 20813136; 20810567; 20797405 |
| BCA_4760 | 8 | 9 | 0.32 | 20803137; 20140210; 19418221; 19366370; 17428661; 17891922; 9250661; 10888843; 10843999; 16981708 |
| BCA_4764 | 4 | 4 | 0.38 | 18303049; 8207058; 10462526; 12665550; 9203579; 12186944; 10196225; 16559104 |
| BCA_4784 | 12 | 12 | 0.97 | 20675455; 20334618; 20179346; 20179356; 20078555; 20028436; 19556294; 19200026; 18814848; 18588815 |
| BCA_4795 | 15 | 18 | 0.31 | 20824277; 20624907; 20513959; 20495044; 20487301; 20333240; 15963225; 19202299; 17288147; 18565772 |
| BCA_4799 | 12 | 12 | 0.99 | 20833209; 20824821; 20652663; 20578976; 20437190; 20346948; 20230906; 20028450; 20004107; 19822151 |
| BCA_4922 | 4 | 4 | 0.75 | 20704697; 20498265; 19904385; 19814796; 19754952; 19748697; 19572471; 19552526; 19528215; 19493004 |
| BCA_4948 | 18 | 18 | 0.91 | 20646353; 20220129; 20208170; 20121136; 20067302; 19906658; 19894768; 19744503; 19639462; 19575528 |
| BCA_4969 | 18 | 18 | 0.91 | 20826817; 20813428; 20812902; 20733269; 20727860; 20718756; 20713354; 20691230; 20665261; 20660679 |
| BCA_4989 | 2 | 2 | 0.58 | 20485749; 20150517; 18180148; 18635006; 16131752; 15147895; 10722690; 10579488; 8218206; 271968 |
| BCA_4995 | 4 | 4 | 0.92 | 20833806; 20607520; 20184451; 19242651; 17332901; 19062645; 16512609; 11717297; 15941997; 9379903 |
| BCA_4998 | 19 | 19 | 0.79 | 20723115; 20722905; 20716056; 20716055; 20716054; 20716053; 20716052; 20716051; 20699471; 20693970 |
| BCA_5035 | 8 | 8 | 0.41 | 20839935; 20826560; 20826128; 20819951; 20810510; 20807655; 20806246; 20712627; 20696780; 20672443 |
| BCA_5037 | 4 | 4 | 0.64 | 20737003; 20678121; 20589733; 20579551; 20448043; 20357106; 20410473; 20185624; 20160144; 20136499 |
| BCA_5052 | 13 | 13 | 0.95 | 20618922; 20169446; 20152552; 19710017; 16769947; 16906134; 19430273; 19412816; 19329989; 19284994 |
| BCA_5056 | 19 | 19 | 0.64 | 20523900; 20435647; 20238176; 20160049; 20118360; 19862515; 19690976; 19683599; 19625620; 19397913 |
| BCA_5059 | 12 | 12 | 0.91 | 20824277; 20632945; 20592285; 20479960; 20359484; 20230906; 20200278; 20173027; 20170058; 20150869 |
| BCA_5113 | 2 | 2 | 0.95 | 20460376; 19810706; 19366265; 16211402; 17941825; 17642475; 17350000; 15964837; 10607655; 9881160 |
| BCA_5114 | 2 | 2 | 0.82 | 20822158; 20460376; 20097860; 19946146; 17350958; 17350000; 11578927; 16455656; 11824608; 10526354 |
| BCA_5184 | 13 | 13 | 0.89 | 19596773; 18343821; 3281834; 14992601; 10600385; 9878398; 359044; 9162081; 9090059; 8454625 |
| BCA_5189 | 12 | 12 | 0.98 | 20692405; 20580091; 20361227; 20300663; 20090473; 20087581; 20045715; 20015640; 19962413; 19913217 |
| BCA_5201 | 18 | 18 | 0.89 | 20848203; 20846699; 20844068; 20842751; 20842510; 20841455; 20837500; 20834143; 20832571; 20830780 |
| BCA_5210 | 12 | 12 | 0.77 | 20848214; 20844119; 20843370; 20842701; 20837478; 20832110; 20819124; 20802519; 20798511; 20797426 |
| BCA_5238 | 13 | 13 | 0.98 | 20348441; 20346161; 20073035; 20038631; 19883769; 19805312; 19776006; 19749382; 19540849; 10564509 |
| BCA_5247 | 8 | 8 | 0.89 | 16534744; 16268782; 12832797; 12729763; 11682175; 10691985; 10463150; 1535626; 7584858; 1535626 |
| BCA_5253 | 17 | 17 | 0.90 | 19969540; 19942657; 19854901; 19843219; 19699748; 19646180; 15175298; 18995832; 10329161; 19426742 |
| BCA_5321 | 4 | 4 | 0.90 | 20806243; 20633642; 20607520; 20232878; 19624711; 19062645; 18803257; 17516097; 17038794; 11014799 |
| BCA_5352 | 12 | 4 | 0.24 | 20593474; 20433810; 20178374; 19717590; 19459002; 19432798; 19129180; 17873043; 15155656; 18286808 |
| BCA_5375 | 19 | 19 | 0.98 | 19298858; 18499663; 18210176; 16829524; 16794327; 15820665; 15555940; 15552059; 15226299; 15161861 |
| BCA_5398 | 18 | 18 | 0.29 | 18312274; 11756511; 11069702; 9332349; 388356; 2549258; 3027504 |
| BCA_5404 | 18 | 8 | 0.31 | 20844759; 20836896; 20827474; 20815261; 20740380; 20739551; 20738841; 20734923; 20731786; 20731394 |
| BCA_5410 | 19 | 19 | 0.90 | 20356564; 20160053; 20035319; 20007685; 19919534; 19421452; 17565364; 18824121; 18757806; 18668237 |
| BCA_5411 | 19 | 19 | 0.94 | 20618940; 20523900; 20383018; 20353610; 20236943; 20226034; 20208178; 20153658; 19890361; 19825588 |
| BCA_5421 | 19 | 19 | 0.92 | 20799932; 20729352; 20717972; 20687808; 20639341; 20633230; 20633048; 20616191; 20616188; 20573954 |
| BCA_5422 | 19 | 19 | 0.60 | 20720312; 20670172; 20621375; 20601475; 20576688; 20576684; 20573193; 20543066; 20510787; 20504312 |
| BCA_5432 | 19 | 19 | 0.94 | 20392080; 20075623; 19899805; 19782148; 19656295; 19298367; 4605290; 18672863; 10529188; 10338486 |
| BCA_5440 | 8 | 8 | 0.87 | 20618950; 20459120; 20107110; 19959836; 19759337; 18784283; 19616643; 19460290; 19441803; 19393250 |
| BCA_5445 | 8 | 8 | 0.87 | 20618950; 20459120; 20107110; 19959836; 19759337; 18784283; 19616643; 19460290; 19441803; 19393250 |
| BCA_5450 | 8 | 8 | 0.85 | 20843806; 20723362; 20663914; 20591132; 20170625; 20154086; 20028336; 19841266; 19785575; 19733997 |
| BCA_5451 | 8 | 8 | 0.79 | 20843806; 20170625; 20154086; 19502237; 17620014; 9834036; 18092810; 8065448; 17983592; 17951718 |
| BCA_5452 | 8 | 8 | 0.89 | 20154086; 19841266; 19796120; 19785575; 19735955; 19733997; 19694452; 19133281; 10836500; 17983592 |
| BCA_5457 | 8 | 8 | 0.91 | 20848398; 20840863; 20827300; 20820587; 20818499; 20817397; 20817117; 20811295; 20809181; 20802119 |
| BCA_5460 | 14 | 14 | 0.92 | 20388844; 20371678; 19844197; 19837844; 19728764; 19704126; 19683539; 19567135; 19565922; 19563437 |
| BCA_5478 | 13 | 4 | 0.24 | 20726420; 20686009; 20675368; 20625716; 20591071; 20564397; 20558535; 20553496; 20533870; 20512986 |
| BCA_5479 | 14 | 14 | 0.90 | 20846129; 20819721; 20817124; 20813185; 20811669; 20810757; 20806045; 20740553; 20733037; 20727927 |
| BCA_5481 | 17 | 17 | 0.84 | 20807197; 20452362; 20413480; 20225147; 20132437; 20124724; 20075920; 20068353; 20002190; 19915588 |
| BCA_5488 | 14 | 2 | 0.29 | 20797482; 20713603; 20661636; 20680001; 20513629; 20213122; 20004571; 19815542; 19754463; 19330326 |
| BCA_5495 | 8 | 8 | 0.94 | 20705057; 20635418; 20592025; 20502966; 20429505; 20405215; 20022118; 19908864; 19817716; 19558967 |
| BCA_5520 | 5 | 5 | 0.96 | 20846141; 20844281; 20840600; 20708298; 20675472; 20666976; 20584149; 20532909; 20522643; 20512387 |
| BCA_5538 | 8 | 8 | 0.88 | 20797619; 20601085; 20535465; 20522803; 20472739; 20444946; 20439410; 20400541; 20363836; 20236319 |
| BCA_5543 | 4 | 4 | 0.62 | 19933362; 17714403; 16907802; 16751597; 9692191; 10348844; 10197998 |
| BCA_5557 | 1 | 1 | 0.98 | 19596340; 10572016; 18216013; 10913262; 17546672; 17490766; 17442255; 17302437; 16708165; 12523389 |
| BCA_5591 | 19 | 11 | 0.98 | 20671018; 20118355; 19967339; 19897657; 18944430; 18451045; 16790772; 17914239; 17897882; 17711456 |
| BCA_5614 | 4 | 15 | 0.93 | 17307848; 17628151; 10745001; 10508770; 10784052; 9016718 |
| BCA_5615 | 4 | 15 | 0.97 | 17307848; 17628151; 10745001; 10508770; 16030236; 10784052; 9016718 |
| BCA_5622 | 14 | 14 | 0.98 | 20827474; 20813141; 20654742; 20524113; 20093117; 20005278; 19603421; 19443045; 19376851; 15645418 |
| BCA_5627 | 13 | 13 | 0.98 | 20034956; 15522293; 10753109; 9370344; 3910100 |
| BCA_5642 | 17 | 17 | 0.83 | 20473316; 20215441; 20075859; 20057077; 19733182; 19376274; 19175708; 10786846; 17284611; 18984152 |
| BCA_A0060 | 3 | 3 | 0.27 | 20846837; 20844249; 20844245; 20842531; 20834154; 20834134; 20834118; 20833802; 20833017; 20822124 |
| BCA_A0101 | 11 | 11 | 0.99 | 20625149; 20615441; 20562304; 20554775; 20553579; 20547379; 20543074; 20512353; 20502527; 20418401 |
| BCA_A0112 | 19 | 19 | 0.65 | 19858184; 19416972; 18923162; 16603733; 15623518; 15459120; 15062540; 12799342; 12757764; 12399434 |
| BCA_A0143 | 6 | 18 | 0.34 | 20708584; 20628788; 20624164; 20495064; 20452131; 20410264; 20235595; 20198890; 20136702; 19966026 |
| BCA_A0162 | 15 | 15 | 0.93 | 20100285; 19995980; 17660417; 10943551; 12224521; 12123659; 11972779; 1556084; 2005875; 10589719 |
| BCA_A0168 | 15 | 15 | 0.93 | 20150239; 19919544; 19648249; 19346355; 18842096; 18375800; 11157930; 17500051; 16519689; 1712012 |
| BCA_A0206 | 6 | 6 | 0.94 | 20844218; 20843780; 20838579; 20811721; 20811674; 20811461; 20808948; 20738175; 20730852; 20719238 |
| CCV52592_0007 | 5 | 18 | 0.88 | 17027372; 17014077; 15644918; 15225600; 11293413 |
| CCV52592_0019 | 14 | 14 | 0.58 | 10766338; 8621608; 8636106; 8052308; 7961708; 7918494; 7929317; 8051113; 1310545; 1317854 |
| CCV52592_0036 | 6 | 11 | 0.64 | 20802084; 20800080; 20723231; 20543074; 20521842; 20502706; 20449818; 20143346; 20085626; 20079696 |
| CCV52592_0058 | 6 | 6 | 0.40 | 20197317; 19150358; 12791985; 17483090; 16181640; 15770476; 12758075; 11956216; 8380461; 10968280 |
| CCV52592_0063 | 6 | 6 | 0.79 | 19889099; 18048927; 17071975; 16756503; 1679430; 15493334; 15476899; 15130124; 1309527; 6330031 |
| CCV52592_0071 | 2 | 2 | 0.87 | 19720147; 19447628; 19442620; 19416104; 19309001; 19155349; 10620343; 18473904; 18466770; 18338647 |
| CCV52592_0081 | 18 | 18 | 0.94 | 20848662; 20848648; 20848555; 20848440; 20848398; 20848389; 20848387; 20848343; 20848165; 20847723 |
| CCV52592_0084 | 2 | 8 | 0.41 | 18407920; 14769888; 14686929; 12140293; 342520; 335020; 1400210; 2699328; 6134653; 6037552 |
| CCV52592_0102 | 18 | 18 | 0.38 | 20709897; 20672825; 20615984; 20610754; 20562855; 20510678; 20505107; 20505103; 20479002; 20463728 |
| CCV52592_0125 | 2 | 2 | 0.81 | 20838591; 20418485; 20182741; 19864628; 19647806; 19554628; 19408950; 19355893; 19270703; 19249205 |
| CCV52592_0147 | 5 | 5 | 0.97 | 20808906; 20418432; 19968123; 19843229; 19767467; 19721220; 19481094; 18021800; 18727912; 18643983 |
| CCV52592_0150 | 12 | 12 | 0.91 | 20552259; 20439464; 20036249; 19665474; 19075746; 18434193; 17565388; 17355436; 16973605; 16143837 |
| CCV52592_0151 | 15 | 15 | 0.95 | 20231359; 9717241; 15979091; 15861392; 11152631; 11150673; 9714706; 9345313; 7504905; 9004222 |
| CCV52592_0154 | 14 | 14 | 0.97 | 18515330; 18082626; 17711307; 17550785; 15272157; 16819826; 16752903; 16621066; 16395552; 15381710 |
| CCV52592_0162 | 6 | 6 | 0.95 | 20823514; 20798848; 20667510; 20504914; 20497557; 20309721; 20300433; 20081204; 20054139; 20036200 |
| CCV52592_0164 | 12 | 18 | 0.89 | 7934840; 12554105; 16092522; 11309117; 10076914; 10071218; 9382730; 6852022 |
| CCV52592_0166 | 15 | 15 | 0.96 | 20118371; 20093290; 20081038; 20012992; 19959580; 19735279; 19447950; 15353566; 18832298; 12448738 |
| CCV52592_0177 | 13 | 13 | 0.98 | 20705654; 20554519; 20441189; 20399793; 20392698; 20237646; 20194507; 20056613; 19773262; 19713960 |
| CCV52592_0178 | 13 | 13 | 0.75 | 20740692; 20100457; 20033061; 19717422; 19833922; 19029596; 18778715; 18471980; 2023261; 17961502 |
| CCV52592_0186 | 13 | 13 | 0.95 | 20700526; 20644643; 20554191; 20472640; 20069063; 20066047; 20056700; 20051305; 20007320; 19776006 |
| CCV52592_0191 | 4 | 4 | 0.55 | 20145127; 20086164; 10940033; 19572210; 19233553; 18945213; 18757823; 18700829; 18633717; 18239155 |
| CCV52592_0196 | 8 | 8 | 0.92 | 20589635; 20305021; 19997580; 19324096; 16491128; 19082487; 18951975; 16497924; 18505274; 18439144 |
| CCV52592_0199 | 9 | 9 | 0.97 | 20485265; 16838328; 16275786; 16544270; 12514242; 10373459; 8858564; 7968268; 1323035; 341153 |
| CCV52592_0201 | 18 | 18 | 0.93 | 6097798; 18248412; 10860977; 11024350; 15451112; 8552661; 9837945; 16348575; 8558445; 1579110 |
| CCV52592_0209 | 18 | 18 | 0.55 | 17643228; 1512189; 3327753; 792395; 1512189; 5541510; 2987841 |
| CCV52592_0213 | 1 | 1 | 0.43 | 19394346; 19041910; 18765924; 18242192; 3700390; 11731178; 1259145; 7642501; 7592354; 13267987 |
| CCV52592_0245 | 19 | 12 | 0.18 | 19923728; 17683397; 9739095; 11292689; 1372291; 1280255; 8631969 |
| CCV52592_0268 | 4 | 4 | 0.62 | 20385832; 19376877; 18391966; 16428326; 11430412; 10048040; 9218429; 8706678; 2161989; 3323846 |
| CCV52592_0284 | 13 | 13 | 0.26 | 15733854; 9286857; 2404005; 2295596; 3284463; 3536904; 3009467; 5271039; 6086058; 6309809 |
| CCV52592_0291 | 19 | 19 | 0.90 | 20412618; 20195888; 20014283; 10612409; 9720026; 9387226; 6237955; 7541787; 2134854 |
| CCV52592_0302 | 11 | 8 | 0.17 | 19946041; 10565539; 1452431; 2270082; 3405753; 2838184 |
| CCV52592_0304 | 19 | 19 | 0.77 | 17532307; 17202151; 16276532; 15750655; 15695810; 15297914; 14574703; 14570895; 14505409; 11932464 |
| CCV52592_0327 | 17 | 13 | 0.70 | 20489203; 19366704; 15764599; 10625642; 3038334; 8422961; 1704882; 2166215; 6207170; 6997720 |
| CCV52592_0335 | 8 | 8 | 0.80 | 20546754; 19821613; 16878993; 12634336; 11329284; 11132640; 10611454; 10587465; 9521665; 9079667 |
| CCV52592_0347 | 13 | 13 | 0.99 | 20601428; 20097853; 19414240; 10550212; 15652481; 18037435; 4600015; 8858588; 17002278; 2025413 |
| CCV52592_0354 | 8 | 8 | 0.89 | 20835425; 20826743; 20816840; 20693662; 20674425; 20673211; 20667822; 20647326; 20623638; 20622431 |
| CCV52592_0365 | 12 | 12 | 1.00 | 20000741; 19682610; 19336036; 18508427; 438215; 8941392; 17490920; 16914159; 11423553; 16243319 |
| CCV52592_0371 | 6 | 6 | 0.95 | 20302878; 19851738; 10373579; 17439637; 9931007; 10388557; 16125907; 16038930; 15713456; 14659743 |
| CCV52592_0400 | 18 | 18 | 0.67 | 20047910; 19854904; 19592589; 19397675; 19054085; 17118662; 15733922; 10498707; 15184553; 11222621 |
| CCV52592_0410 | 18 | 18 | 0.98 | 20805396; 20727860; 20676995; 20610168; 20608983; 20508090; 20495209; 20449741; 20419433; 20401594 |
| CCV52592_0415 | 1 | 1 | 0.87 | 20846141; 20565711; 20427272; 20056607; 19932104; 16814790; 19624748; 19449898; 19374926; 19083499 |
| CCV52592_0429 | 8 | 8 | 0.72 | 20673834; 19265402; 17007434; 16913910; 7849603; 8497190; 9546662; 10803945; 10525739; 10491142 |
| CCV52592_0432 | 18 | 12 | 0.37 | 20828170; 20709029; 20706981; 20679996; 20675712; 20631203; 20571077; 20555137; 20484932; 20405068 |
| CCV52592_0441 | 4 | 4 | 0.92 | 20132451; 19376867; 18957286; 11528005; 17090391; 16720646; 1631122; 15699192; 15276839; 15136044 |
| CCV52592_0443 | 8 | 8 | 0.87 | 20818499; 20726783; 20719858; 20705952; 20692053; 20687127; 20617129; 20614135; 20602244; 20600937 |
| CCV52592_0450 | 19 | 19 | 0.98 | 20847002; 20843347; 20801168; 20733036; 20726582; 20713676; 20704258; 20643433; 20608745; 20593835 |
| CCV52592_0491 | 18 | 18 | 0.97 | 20400641; 20385770; 20223824; 20112249; 19864301; 19636250; 19584562; 19366604; 19341747; 19307180 |
| CCV52592_0502 | 13 | 18 | 0.41 | 20848228; 20848211; 20848131; 20847300; 20847183; 20847098; 20847002; 20846957; 20846785; 20846497 |
| CCV52592_0506 | 6 | 6 | 0.84 | 20337945; 20222445; 20203672; 20195500; 20122408; 19920184; 19844165; 19783657; 19749191; 19732344 |
| CCV52592_0512 | 1 | 8 | 0.97 | 20846325; 20818499; 20684326; 20664702; 20629101; 20625650; 20621659; 20570330; 20522323; 20476564 |
| CCV52592_0521 | 4 | 19 | 0.34 | 20846779; 20846492; 20823491; 20844270; 20840469; 20838025; 20833923; 20833395; 20833149; 20828647 |
| CCV52592_0524 | 18 | 18 | 0.92 | 20387456; 20335169; 19919671; 19854904; 18940858; 19270115; 19153809; 19121687; 10087167; 18564683 |
| CCV52592_0559 | 14 | 14 | 0.94 | 19683509; 19385996; 15055759; 18597482; 18164314; 17996716; 11053429; 17651436; 8583252; 15539408 |
| CCV52592_0615 | 8 | 12 | 0.29 | 20605975; 20592392; 20592377; 20592343; 20583999; 20540604; 20526552; 20506831; 20505099; 20505084 |
| CCV52592_0638 | 8 | 8 | 0.42 | 20821442; 20533838; 18942856; 18834129; 18767150; 18393619; 14521881; 17426034; 16142921; 16061234 |
| CCV52592_0641 | 18 | 18 | 0.66 | 17023541; 8675685; 17580307; 9884020; 15300204; 12795614; 11960774; 11672685; 11210393; 10608467 |
| CCV52592_0649 | 12 | 12 | 0.96 | 20654624; 20453896; 20130682; 20130680; 20083401; 19880603; 19714768; 19476965; 19424556; 19351326 |
| CCV52592_0656 | 9 | 9 | 0.96 | 20418430; 20178986; 20158476; 20153183; 19664929; 18804030; 18824113; 18721141; 2469386; 18453702 |
| CCV52592_0661 | 18 | 18 | 0.77 | 10037698; 1482126; 1985888; 3027504; 3534792; 3023201 |
| CCV52592_0667 | 2 | 2 | 0.47 | 19266201; 18726075; 10712585; 15065678; 14675432; 12906829; 12842039; 10481043; 12773157; 12515554 |
| CCV52592_0680 | 13 | 13 | 0.98 | 19799608; 19774386; 19469554; 19245787; 19071121; 18676770; 18266979; 17157877; 16776290; 16609016 |
| CCV52592_0683 | 12 | 19 | 0.63 | 20441706; 16042374; 11254610; 17709535; 17235642; 16840781; 9588809; 14660386; 10223944; 2615650 |
| CCV52592_0696 | 2 | 2 | 0.96 | 20208151; 20064433; 18451506; 12878729; 18047786; 16452427; 17442674; 17081012; 15649375; 15466439 |
| CCV52592_0707 | 12 | 12 | 0.89 | 3049077; 10582129; 7883706; 2828030; 1830665; 8387148; 1588819; 7048314; 2041472; 2170107 |
| CCV52592_0716 | 4 | 4 | 0.77 | 20378831; 19747079; 19705835; 19606502; 19324687; 19231145; 19037090; 7995518; 10466731; 18667570 |
| CCV52592_0721 | 18 | 18 | 0.86 | 20846512; 20829454; 20826142; 20821325; 20811716; 20811452; 20739287; 20736085; 20736053; 20710118 |
| CCV52592_0729 | 9 | 9 | 0.63 | 14651609; 9370338; 8227017; 1929418; 2002005; 2007589; 2406271; 3042771; 3084471 |
| CCV52592_0731 | 18 | 18 | 0.98 | 20700443; 20696983; 20634315; 20601117; 20595681; 20595678; 20574420; 20568735; 20519162; 20518490 |
| CCV52592_0739 | 8 | 8 | 0.89 | 20845334; 20840452; 20818499; 20726783; 20695200; 20673213; 20665546; 20643807; 20630171; 20561988 |
| CCV52592_0746 | 17 | 17 | 0.93 | 20724091; 20227844; 20087629; 19936829; 19528202; 11675503; 19467815; 19443547; 19329646; 19329591 |
| CCV52592_0757 | 15 | 8 | 0.25 | 17640273; 17379835; 17085647; 11522292; 14701827; 16330552; 1404594; 15735307; 12661770; 10748138 |
| CCV52592_0770 | 13 | 13 | 0.91 | 19734148; 19721072; 19309487; 19228694; 19174549; 18818520; 18422966; 18067851; 16857674; 10430557 |
| CCV52592_0774 | 13 | 13 | 0.89 | 12736709; 1310418; 11571299; 10523634; 10428788; 16562115 |
| CCV52592_0777 | 4 | 4 | 0.93 | 20581225; 20534509; 18391446; 18174138; 16806204; 7603411; 16436427; 15528673; 12958592; 12018657 |
| CCV52592_0779 | 4 | 4 | 0.93 | 20203055; 10594820; 8454190; 18174138; 10063642; 15687183; 15528673; 15361076; 11430400; 9254694 |
| CCV52592_0786 | 4 | 4 | 0.80 | 20639318; 17492271; 12940991; 12401220; 11405627; 1915293; 1640458; 10320579; 9168617; 7770032 |
| CCV52592_0788 | 4 | 4 | 0.89 | 20829360; 20815790; 20728562; 20649474; 20627362; 20626112; 20591624; 20583283; 20582873; 20528770 |
| CCV52592_0828 | 13 | 13 | 0.98 | 19377097; 18465791; 10572144; 17310068; 10417132; 15950761; 15049826; 11849648; 11328654; 10600119 |
| CCV52592_0861 | 17 | 17 | 0.56 | 20846394; 20814424; 20810619; 20807197; 20705940; 20694660; 20673126; 20630870; 20550934; 20590996 |
| CCV52592_0862 | 6 | 6 | 0.90 | 20675375; 20223211; 16507358; 16430690; 15758241; 12940977; 9144786; 12535532; 11859073; 11823461 |
| CCV52592_0864 | 19 | 8 | 0.32 | 19946721; 17876502; 10527863; 9630539; 11222605; 11913796; 10694878; 10352233; 4200860; 3406101 |
| CCV52592_0894 | 12 | 12 | 0.96 | 19304828; 18704367; 18174150; 17011680; 16944097; 16872603; 15327959; 14662964; 12198306; 11422376 |
| CCV52592_0902 | 8 | 8 | 0.79 | 11864982; 18338855; 16669619; 16211847; 12126468; 11768747; 11334784; 10985736; 9497378; 8661920 |
| CCV52592_0911 | 13 | 13 | 0.99 | 20601428; 20498846; 19781576; 19619301; 17321546; 19469554; 10550212; 19398033; 15652481; 18313723 |
| CCV52592_0912 | 13 | 8 | 0.31 | 20579188; 20221446; 19764821; 19733997; 19559745; 19538026; 19538018; 19538016; 19520134; 19015021 |
| CCV52592_0955 | 18 | 18 | 0.98 | 20164445; 19919002; 19818021; 19748784; 10805817; 18511655; 18324396; 11602359; 17992631; 17322901 |
| CCV52592_0967 | 9 | 9 | 0.98 | 18167308; 10361297; 17850764; 17726007; 17675291; 16847310; 16620760; 16243729; 16150824; 10231527 |
| CCV52592_0971 | 1 | 8 | 0.44 | 20043233; 20080659; 19603180; 19378003; 19211556; 19154813; 19076230; 18817177; 18558720; 18368293 |
| CCV52592_0980 | 1 | 1 | 0.97 | 20086163; 19153452; 19072614; 18216013; 17927957; 12177356; 16708165; 16313180; 16232856; 16216079 |
| CCV52592_1007 | 13 | 13 | 0.98 | 20734330; 20410297; 20020415; 19468072; 7965057; 19160485; 17498616; 15849735; 10722742; 16469478 |
| CCV52592_1009 | 13 | 13 | 0.96 | 20723362; 20459660; 20386923; 19776006; 19640295; 19616559; 19386726; 4612527; 19132063; 18573842 |
| CCV52592_1010 | 13 | 13 | 0.94 | 15588818; 15522293; 15300802; 14729335; 14568531; 12937172; 12894494; 10858528; 12054647; 11735401 |
| CCV52592_1016 | 13 | 13 | 0.99 | 20176963; 20123129; 19969550; 19807034; 19706509; 19631221; 19559069; 18801746; 19405028; 19386726 |
| CCV52592_1017 | 13 | 13 | 0.97 | 20220312; 19588103; 19071121; 16195786; 14687565; 10766838; 11566355; 11524947; 11470155; 11244494 |
| CCV52592_1019 | 13 | 13 | 0.99 | 20423933; 15222900; 16990266; 18289901; 15522791; 15522293; 9468784; 11478863; 12914937; 12823975 |
| CCV52592_1024 | 13 | 13 | 0.94 | 19953637; 19616559; 18421856; 17647292; 10445873; 11796124; 11289525; 10561594; 9800350; 9169412 |
| CCV52592_1026 | 13 | 13 | 0.97 | 19477153; 19363482; 19196760; 19071121; 10619032; 17613524; 16830097; 16390447; 15621428; 15561149 |
| CCV52592_1029 | 13 | 13 | 0.98 | 20727857; 20429542; 20097853; 19454243; 18573842; 11013226; 16940070; 10338213; 17013933; 16604111 |
| CCV52592_1030 | 13 | 13 | 0.99 | 20703089; 20601428; 20554519; 20020415; 19783770; 19730979; 19581052; 19450532; 19160485; 19033382 |
| CCV52592_1035 | 18 | 18 | 0.72 | 20363944; 19661178; 19646180; 19569551; 18957436; 18701447; 10368146; 18305482; 12813062; 17643228 |
| CCV52592_1053 | 4 | 4 | 0.67 | 19682914; 19099445; 18930130; 18483760; 17311458; 17014073; 16686542; 16436421; 15751951; 15450491 |
| CCV52592_1069 | 2 | 2 | 0.96 | 18154309; 15632135; 15269205; 15247236; 15159566; 12583917; 12504674; 12006571; 11554796; 11428898 |
| CCV52592_1084 | 18 | 6 | 0.23 | 20691074; 20633111; 20616517; 20496201; 20452008; 20355123; 20338900; 20307498; 20185303; 20152959 |
| CCV52592_1092 | 19 | 19 | 0.94 | 20392080; 20075623; 19899805; 19782148; 19656295; 19298367; 4605290; 18672863; 10529188; 10338486 |
| CCV52592_1110 | 18 | 18 | 0.86 | 20387456; 20041993; 19731368; 18940858; 19451245; 19432807; 19153809; 16475801; 18043643; 17997964 |
| CCV52592_1137 | 9 | 8 | 0.45 | 19464381; 18674553; 16307764; 16299351; 15831896; 14756531; 6016335; 11211270; 11042205; 11288749 |
| CCV52592_1145 | 4 | 3 | 0.99 | 20796283; 20595000; 20487019; 20439474; 20398206; 20338182; 20018629; 20008072; 19964414; 19801658 |
| CCV52592_1159 | 8 | 8 | 0.88 | 20797619; 20601085; 20535465; 20522803; 20472739; 20444946; 20439410; 20400541; 20363836; 20236319 |
| CCV52592_1200 | 1 | 1 | 0.20 | 11551184; 3318818; 11264293; 10387030; 11170408; 8494895 |
| CCV52592_1203 | 19 | 19 | 0.62 | 10829079; 9163424; 11390676; 10865948; 9317046; 8529891 |
| CCV52592_1209 | 6 | 6 | 0.97 | 10323239; 10627049; 9479048; 3014324; 2162963 |
| CCV52592_1211 | 19 | 19 | 0.88 | 20624909; 20453127; 20435647; 20479750; 20418333; 20372031; 20363943; 20202936; 20177662; 20147450 |
| CCV52592_1218 | 19 | 19 | 0.55 | 10481091; 14533819; 9453660; 12726766; 12435504; 11981443; 11744630; 11589581; 11390676; 10627048 |
| CCV52592_1237 | 19 | 19 | 0.68 | 19346244; 19201821; 8955282; 10417132; 10196742; 11717579; 11521078; 11054112; 10981699; 10951204 |
| CCV52592_1247 | 8 | 8 | 0.94 | 20724479; 20629101; 20610654; 20561582; 20544786; 20536736; 20483191; 20481463; 20441221; 20413620 |
| CCV52592_1251 | 12 | 12 | 0.96 | 20832960; 20828778; 20824169; 20811517; 20806899; 20731414; 20702722; 20696584; 20682085; 20670659 |
| CCV52592_1311 | 6 | 6 | 0.97 | 20675375; 20554816; 20413500; 20357218; 20195500; 20188667; 20176899; 20157337; 20144152; 20033171 |
| CCV52592_1312 | 4 | 4 | 0.77 | 20848212; 20848190; 20847723; 20847445; 20846521; 20846484; 20846340; 20846025; 20845086; 20845026 |
| CCV52592_1313 | 8 | 8 | 0.82 | 19923736; 19328460; 18640127; 18066434; 1106126; 15590681; 7921236; 7758956; 1643581 |
| CCV52592_1314 | 12 | 12 | 0.63 | 20679433; 20599535; 20585451; 20512970; 20457605; 20419412; 20419406; 20419102; 20306492; 20237155 |
| CCV52592_1317 | 12 | 12 | 0.33 | 20479960; 20359484; 20150869; 19931316; 19931315; 19854289; 19724142; 19463860; 19433052; 19428710 |
| CCV52592_1331 | 4 | 4 | 0.74 | 20298562; 18697747; 18199454; 18001143; 10811894; 16157581; 14636056; 1465418; 3007436; 6748081 |
| CCV52592_1346 | 2 | 15 | 0.35 | 20847284; 20842687; 20842020; 20838263; 20818608; 20716859; 20590807; 20610621; 20498189; 20491893 |
| CCV52592_1360 | 15 | 15 | 0.68 | 11321566; 17884222; 10852879; 1884998; 1690605; 1402666; 2295883; 2427017 |
| CCV52592_1361 | 12 | 12 | 0.97 | 20847048; 20803087; 20735358; 20668094; 20648511; 20583963; 20504766; 20499929; 20487289; 20448033 |
| CCV52592_1364 | 19 | 19 | 0.79 | 10527863; 9630539; 11222605; 1447138; 4200860 |
| CCV52592_1370 | 14 | 8 | 0.90 | 2204419; 16385189; 15695810; 15606755; 9092578; 3392008 |
| CCV52592_1374 | 13 | 13 | 0.19 | 15733854; 2404005; 2295596; 3284463; 3536904; 3009467; 5271039; 6086058; 6309809; 6290471 |
| CCV52592_1383 | 15 | 18 | 0.35 | 20644138; 20571957; 20562310; 20437149; 20430815; 20372024; 20237769; 20100857; 20055984; 20046512 |
| CCV52592_1411 | 15 | 15 | 0.77 | 19894045; 18587410; 18495502; 16221580; 16205910; 15455157; 11523004; 12581638; 3301812; 11836258 |
| CCV52592_1422 | 1 | 1 | 0.94 | 20817725; 20482655; 20332210; 19948253; 19931317; 19699761; 12601748; 12818659; 19193709; 19121344 |
| CCV52592_1439 | 4 | 4 | 0.80 | 20693676; 20543140; 20439729; 20346719; 20133562; 19858188; 19767432; 19361426; 19358329; 19123032 |
| CCV52592_1475 | 6 | 6 | 0.95 | 20302878; 17439637; 16125907; 15713456; 14659743; 14600243; 12879741; 11724530; 11555298; 11136134 |
| CCV52592_1521 | 8 | 8 | 0.54 | 19815558; 19189973; 17894548; 10652103; 15996109; 15922336; 11695824; 15736965; 15175326; 15102833 |
| CCV52592_1522 | 8 | 8 | 0.45 | 19815558; 19189973; 10652103; 15996109; 15922336; 11695824; 15736965; 15175326; 15035646; 12914922 |
| CCV52592_1523 | 13 | 13 | 0.94 | 20718859; 20700687; 20596537; 20534494; 20526830; 20522549; 20471434; 20457640; 20435138; 20434456 |
| CCV52592_1544 | 13 | 13 | 0.99 | 20454621; 20037770; 18060665; 17996708; 15652215; 15215513; 15211551; 15143667; 14729335; 14623272 |
| CCV52592_1547 | 8 | 8 | 0.91 | 20807714; 20799369; 20705660; 20730854; 20660112; 20628047; 20624914; 20622059; 20555380; 20522731 |
| CCV52592_1551 | 15 | 15 | 0.84 | 20847946; 20847010; 20844005; 20843353; 20837772; 20837600; 20834230; 20834163; 20833896; 20833806 |
| CCV52592_1553 | 4 | 4 | 0.92 | 20534509; 20118266; 20096108; 19961544; 19465658; 19376867; 12366832; 18216858; 11008110; 17572072 |
| CCV52592_1555 | 13 | 13 | 0.98 | 20418391; 20206660; 19633085; 12068810; 19075940; 18610839; 17889642; 17889647; 12831880; 17194931 |
| CCV52592_1557 | 2 | 2 | 0.93 | 16922603; 12767809; 12602878; 12459457; 9267435; 13295297; 1445864; 1643048; 1567858; 1905724 |
| CCV52592_1575 | 12 | 12 | 0.90 | 19799526; 17300178; 14580344; 12813051; 12029389; 10939242; 9660752 |
| CCV52592_1583 | 17 | 17 | 0.78 | 19863111; 19805312; 19779461; 19617368; 19361424; 19215773; 17405875; 11937637; 11586360; 336626 |
| CCV52592_1585 | 2 | 2 | 0.85 | 7009558; 17033719; 1368143; 12799002; 3085688 |
| CCV52592_1587 | 13 | 13 | 0.98 | 20616875; 20091347; 20034956; 19903879; 19759058; 16021622; 10383418; 12620932; 11607112; 16939575 |
| CCV52592_1589 | 12 | 12 | 0.98 | 20828137; 20826753; 20825413; 20818167; 20812737; 20811381; 20801545; 20707733; 20696895; 20695487 |
| CCV52592_1597 | 1 | 1 | 0.93 | 20039042; 19955263; 19383689; 19298998; 19011861; 11121418; 18050920; 17853367; 17611817; 17482430 |
| CCV52592_1605 | 8 | 8 | 0.86 | 20154124; 17288147; 16880463; 15870453; 11814762; 11199849; 13076; 1730488; 8799475; 8530465 |
| CCV52592_1615 | 6 | 18 | 0.99 | 20639324; 20395595; 19775248; 19761223; 10805817; 19428657; 19046159; 18990190; 18616590; 18247478 |
| CCV52592_1652 | 19 | 19 | 0.54 | 19883124; 17975091; 15632436; 12000771; 8982270; 6370994; 1597857; 1495426; 3049593; 6296774 |
| CCV52592_1659 | 1 | 1 | 0.76 | 20805334; 19551475; 17588931; 12777757; 14699121; 10220164; 10196182; 7652206; 8458344; 1327967 |
| CCV52592_1660 | 8 | 8 | 0.86 | 20845075; 20826743; 20816840; 20600873; 20570330; 20558052; 20554191; 20471957; 20471925; 20194725 |
| CCV52592_1666 | 1 | 6 | 0.28 | 20843005; 20840606; 20833271; 20824297; 20813207; 20805291; 20726950; 20724007; 20705647; 20668089 |
| CCV52592_1668 | 4 | 4 | 0.85 | 18719175; 17015641; 10468575; 10468575; 8107139; 1404383; 2179562; 1905667; 2181149; 387724 |
| CCV52592_1677 | 6 | 6 | 0.93 | 20671152; 20682983; 20682398; 20679393; 20670142; 20668266; 20651149; 20644589; 20586875; 20613874 |
| CCV52592_1702 | 15 | 8 | 0.35 | 18155044; 15248747; 12475257; 12077442; 9261133; 8016863; 4593930; 8212131; 2849575; 6130093 |
| CCV52592_1716 | 6 | 6 | 0.95 | 20599730; 20511501; 20331425; 20204869; 20180846; 20134230; 20133978; 20132031; 20118257; 20078043 |
| CCV52592_1724 | 13 | 13 | 0.97 | 20043235; 20033390; 19477153; 18077114; 17569339; 16944589; 15634699; 16388861; 9050872; 12969427 |
| CCV52592_1734 | 1 | 8 | 0.37 | 20805334; 20652880; 20352489; 20047974; 20093640; 19963421; 19921396; 19747453; 19731381; 19596050 |
| CCV52592_1740 | 15 | 17 | 0.53 | 19854830; 17951384; 16815708; 14712703; 16337964; 15294156; 15200953; 12914699; 12914690; 12213659 |
| CCV52592_1757 | 12 | 18 | 0.42 | 20521846; 20466908; 19850876; 19815580; 19182809; 18310044; 10449368; 15851036; 3042772; 8824631 |
| CCV52592_1800 | 2 | 8 | 0.70 | 20816949; 20806221; 20655876; 20616050; 20610102; 20593779; 20553774; 19772255; 20486657; 20471397 |
| CCV52592_1814 | 8 | 8 | 0.97 | 20614874; 20498375; 20130997; 20091229; 20075289; 19577535; 19285991; 19254042; 19234303; 9872799 |
| CCV52592_1837 | 12 | 12 | 0.96 | 20847048; 20841453; 20827447; 20729526; 20727857; 20668094; 20499929; 20487289; 20448033; 20439464 |
| CCV52592_1838 | 14 | 14 | 0.97 | 9843369; 11323713; 9598063; 2192230; 2687276 |
| CCV52592_1846 | 8 | 8 | 0.83 | 20661960; 19579240; 19115296; 18215050; 10080900; 16302255; 15959893; 15669071; 12962479; 12206758 |
| CCV52592_1859 | 12 | 12 | 0.74 | 20844218; 17229438; 16403873; 16229488; 15260479; 15007058; 11336818; 11313128; 11264585; 10369667 |
| CCV52592_1873 | 8 | 8 | 0.88 | 20583616; 20551992; 20540712; 20529087; 20379037; 20039900; 20028790; 20000467; 20213850; 19859792 |
| CCV52592_1896 | 18 | 18 | 0.85 | 20438880; 20053353; 19923224; 19922410; 19448069; 19135453; 19372598; 19366604; 19363115; 19274728 |
| CCV52592_1903 | 14 | 14 | 0.88 | 20593468; 20353400; 20217185; 20065942; 19995545; 19749371; 19749250; 19749249; 19631609; 19540237 |
| CCV52592_1922 | 6 | 6 | 0.93 | 20670908; 20601468; 20304994; 20157002; 19997622; 19997508; 19580006; 19369355; 19072585; 10506828 |
| CCV52592_1967 | 1 | 1 | 0.92 | 20709017; 20503968; 20353808; 20227435; 20222969; 20122228; 20057065; 20057050; 20054116; 20025926 |
| CCV52592_1986 | 4 | 4 | 0.53 | 2680983; 15980386; 18435557; 17650960; 15189150; 10469189; 9858724; 8793871; 1331988; 1513876 |
| CCV52592_2026 | 4 | 8 | 0.35 | 20485749; 20097757; 20080101; 19924248; 19858213; 19625248; 19251824; 18818197; 18688478; 15371447 |
| CCV52592_2035 | 4 | 4 | 0.67 | 20845092; 20799341; 20708625; 20603107; 20559421; 20493879; 20424185; 20369041; 20354188; 20303133 |
| CCV52592_2042 | 8 | 8 | 0.95 | 20816850; 20697122; 20691393; 20691179; 20689707; 20660112; 20646165; 20625939; 20618079; 20599983 |
| CCV52592_2057 | 6 | 6 | 0.92 | 9135161; 18606573; 17873859; 15568977; 12206670; 7836288; 11152689; 11090620; 11009612; 10610789 |
| CCV52592_2059 | 18 | 18 | 0.90 | 10627041; 1107801; 1445856; 1569065; 1809844 |
| CCV52592_2060 | 8 | 8 | 0.94 | 20675490; 20445244; 20349330; 20179327; 20163138; 19899808; 19837837; 19725515; 19626710; 19561185 |
| CCV52592_2063 | 14 | 19 | 0.37 | 20595007; 20445247; 20413228; 19643076; 19459936; 19370021; 1097407; 18248423; 12781525; 17609801 |
| CCV52592_2070 | 8 | 8 | 0.37 | 20140718; 20075610; 19830420; 18342972; 11640987; 11525624; 11255157; 11223141; 10681054; 10194852 |
| CCV52592_2112 | 19 | 19 | 0.96 | 20615998; 20079869; 19531602; 19412576; 19245333; 18679678; 18421587; 18379842; 18318839; 17970751 |
| CCV52592_2115 | 17 | 17 | 0.47 | 20705233; 20603000; 20584897; 20569207; 20528154; 20519410; 20519371; 20504959; 20471939; 20565849 |
| CCV52592_2120 | 15 | 12 | 0.33 | 20848536; 20848509; 20848498; 20848489; 20848362; 20848307; 20848273; 20848249; 20848247; 20848229 |
| CCV52592_2204 | 8 | 8 | 0.54 | 12409197; 11850430; 11279023; 11004459; 7984417; 9533030; 8195189 |
| CCV52592_2205 | 8 | 8 | 0.88 | 20583616; 20551992; 20540712; 20529087; 20379037; 20149100; 20039900; 20028790; 20000467; 20213850 |
| MJ | 8 | 8 | 0.93 | 12860407; 20511510; 18298089; 16221580; 11197482; 10491142; 8617280; 8575452; 7649162; 1904129 |
| MJ_0005 | 8 | 8 | 0.97 | 10652088; 12679550; 12605683; 11929547; 11372198; 9546395; 9808754; 9756865; 9579062; 8723654 |
| MJ_0047 | 17 | 17 | 0.32 | 19576221; 8172903; 18703506; 16600876; 10383760; 9334264; 16207706; 16115062; 15955310; 12145212 |
| MJ_0050 | 8 | 1 | 0.40 | 20813141; 20348336; 20089922; 19842059; 19776269; 19731323; 19614976; 19376158; 19229853; 19225177 |
| MJ_0055 | 2 | 2 | 0.94 | 20806221; 20230506; 18534824; 18298940; 18262398; 18219123; 10438747; 17541777; 17400171; 7622491 |
| MJ_0057 | 18 | 18 | 0.75 | 20677981; 20650263; 20643930; 20562308; 20557427; 20507995; 20501797; 20338684; 20307259; 20298477 |
| MJ_0058 | 18 | 18 | 0.99 | 20197030; 20088899; 20067779; 20013387; 20004175; 19961846; 19857597; 19706285; 19659660; 19594831 |
| MJ_0060 | 14 | 14 | 0.55 | 20718423; 20637465; 20596646; 20513530; 20500769; 20449600; 20302706; 20194690; 20140953; 20140939 |
| MJ_0066 | 1 | 5 | 0.99 | 17993606; 17519237; 17308787; 16962072; 16049108; 12740361; 12716056; 12501187; 12072441; 10950850 |
| MJ_0085 | 18 | 18 | 0.99 | 19004000; 18252722; 11872826; 17387526; 12829271; 1393837; 15918073; 9781885; 10447888; 2172216 |
| MJ_0101 | 12 | 12 | 0.49 | 20487556; 17452350; 18755190; 18281057; 18089836; 14726591; 16024227; 10675322; 15632183; 14569414 |
| MJ_0124 | 6 | 6 | 0.95 | 20302878; 19425495; 11600708; 17439637; 9016653; 11163967; 16649659; 8887569; 16126220; 16125907 |
| MJ_0130 | 6 | 6 | 0.93 | 20302878; 20184512; 19851738; 17202163; 19425495; 19079266; 11600708; 18164032; 10373579; 17439637 |
| MJ_0139 | 18 | 18 | 0.93 | 20802201; 20671071; 20649599; 20592205; 20582984; 20571950; 20568711; 20479001; 20425031; 20405001 |
| MJ_0148 | 13 | 13 | 0.84 | 10089432; 9590285; 9590285; 10383384; 9483794; 9843401; 1561104 |
| MJ_0161 | 1 | 1 | 0.58 | 20081005; 17624809; 10188206; 16795146; 16458324; 9696751; 12488095; 12060231; 11243831; 10942289 |
| MJ_0174 | 4 | 4 | 0.28 | 20847002; 20842122; 20838207; 20829293; 20818439; 20818414; 20817757; 20815822; 20811636; 20810728 |
| MJ_0189 | 13 | 13 | 0.88 | 20667097; 20540604; 20528918; 20419091; 20392919; 20333284; 20208990; 20206592; 20176963; 20138832 |
| MJ_0197 | 17 | 17 | 0.94 | 20392699; 20088966; 19836295; 19580872; 19465661; 19208825; 19201563; 19196760; 18687781; 10565912 |
| MJ_0208 | 8 | 8 | 0.89 | 20815377; 20695524; 20669901; 20658302; 20614874; 20606288; 20564035; 20559373; 20547883; 20545868 |
| MJ_0222 | 8 | 8 | 0.73 | 20670834; 20615420; 20614849; 20591132; 20581234; 20578995; 20602994; 20518749; 20518490; 20514864 |
| MJ_0242 | 13 | 13 | 0.93 | 20580850; 20357218; 20208990; 20034997; 20011513; 19794048; 19628620; 10578177; 9916101; 19454283 |
| MJ_0247 | 6 | 6 | 0.51 | 20848439; 20848291; 20847638; 20844807; 20840871; 20840853; 20840814; 20840519; 20839281; 20839075 |
| MJ_0254 | 6 | 6 | 0.97 | 20811636; 20800503; 20705129; 20633226; 20625734; 20613862; 20531307; 20529341; 20521842; 20501624 |
| MJ_0260 | 12 | 12 | 0.95 | 20660367; 20522495; 20465851; 20385966; 20334693; 20300663; 20228117; 20159018; 20140249; 20100702 |
| MJ_0267 | 8 | 8 | 0.98 | 20618950; 20433942; 20225259; 20110695; 19767525; 19694452; 19119913; 16458324; 18983854; 18765295 |
| MJ_0272 | 15 | 15 | 0.90 | 20831592; 20727012; 20716622; 20709902; 20709099; 20624818; 20624068; 20621064; 20583753; 20577782 |
| MJ_0285 | 12 | 12 | 0.92 | 20846409; 20844575; 20843828; 20842208; 20841485; 20841355; 20829522; 20828243; 20813964; 20809504 |
| MJ_0363 | 6 | 6 | 0.97 | 20847638; 20332239; 19877719; 19696745; 18657502; 19150984; 19143592; 19001846; 9525946; 9214646 |
| MJ_0369 | 6 | 6 | 0.93 | 17921483; 15939019; 11983162; 12401175; 11820813; 11410368; 10545127; 9801313; 9121546 |
| MJ_0476 | 13 | 13 | 0.93 | 20580850; 20357218; 20208990; 20034997; 20011513; 19794048; 19628620; 10578177; 9916101; 19454283 |
| MJ_0502 | 1 | 1 | 0.63 | 20043233; 20080659; 19807158; 19799945; 19603180; 19583445; 19378003; 19303906; 19271774; 19211556 |
| MJ_0507 | 17 | 17 | 0.70 | 20353996; 19913479; 16945932; 15315821; 10591646; 9837802; 8047170; 8663456; 8902800; 3211138 |
| MJ_0544 | 19 | 19 | 0.35 | 9254694; 16284928; 15913610; 12068013; 9595667; 8813763; 6343825; 8226966; 8344954; 2162198 |
| MJ_0559 | 4 | 4 | 0.69 | 20054112; 19843181; 19021761; 18323612; 17561111; 15489502; 12595266; 9193092; 11524683; 11264598 |
| MJ_0571 | 1 | 1 | 0.86 | 20848228; 20838028; 20837041; 20830520; 20804597; 20813095; 20807241; 20806654; 20804784; 20724183 |
| MJ_0601 | 2 | 2 | 0.94 | 20739284; 20716937; 20684226; 20674574; 20648783; 20647326; 20600129; 20553497; 20519180; 20439498 |
| MJ_0613 | 6 | 6 | 0.69 | 20233728; 20018860; 19812547; 19747545; 19422832; 15358275; 19074649; 19019141; 17914173; 18956747 |
| MJ_0615 | 8 | 8 | 0.76 | 20578995; 20518490; 20511227; 20472544; 20460381; 20435646; 20188694; 20082212; 20080582; 19995987 |
| MJ_0631 | 12 | 12 | 0.55 | 20807532; 20463892; 20023111; 19720045; 9647800; 14652736; 19117946; 10795682; 17903205; 17639348 |
| MJ_0636 | 8 | 8 | 0.90 | 20675489; 20093189; 18391450; 14594853; 17237047; 16945443; 16814740; 15191624; 15110863; 12373779 |
| MJ_0637 | 1 | 1 | 0.90 | 20137911; 20052993; 19948253; 19508381; 19082689; 18662373; 4536832; 18384085; 11344458; 18171624 |
| MJ_0649 | 4 | 8 | 0.93 | 20795369; 20727352; 20675490; 20655590; 20630484; 20518072; 20496826; 20462348; 20456960; 20395268 |
| MJ_0654 | 14 | 14 | 0.73 | 20708304; 20702404; 20606073; 20603160; 20647386; 20544541; 20489727; 20473969; 20413687; 20399851 |
| MJ_0667 | 14 | 14 | 0.92 | 20837458; 20804144; 20727737; 20714877; 20700125; 20656421; 20651404; 20596627; 20593055; 20585803 |
| MJ_0671 | 2 | 8 | 0.75 | 20847280; 20847215; 20847137; 20846495; 20846490; 20846448; 20846340; 20845390; 20845308; 20844569 |
| MJ_0744 | 8 | 8 | 0.42 | 17929940; 16388601; 12167658; 11952791; 9063468; 7925445; 1772346 |
| MJ_0806 | 12 | 12 | 0.79 | 19574214; 18515364; 11423553; 1537867; 10871044; 10708856; 10095056; 7765336; 2659585 |
| MJ_0813 | 2 | 2 | 0.96 | 9665173; 9660189; 9331403; 9224567; 7606163; 7842859; 1731915; 8405386; 1451790; 1731915 |
| MJ_0822 | 3 | 3 | 0.69 | 20833802; 20650318; 20603129; 20596584; 20513375; 20488501; 20307596; 20097205; 20067320; 19851018 |
| MJ_0864 | 8 | 8 | 0.92 | 20629748; 20534465; 20235826; 20178638; 19968794; 19948802; 12604213; 10406099; 17929940; 15317781 |
| MJ_0879 | 5 | 5 | 0.95 | 20455943; 20429505; 20185682; 20173737; 20105217; 19898541; 19833949; 19783748; 19771413; 19763409 |
| MJ_0900 | 12 | 12 | 0.83 | 20681640; 20345654; 20065028; 19857645; 19829005; 19801409; 19768502; 19642226; 19586415; 19199708 |
| MJ_0918 | 1 | 1 | 0.50 | 17938992; 11856350; 11265469; 10700266; 12024219; 10688189; 6343825; 7727400; 2515110; 7687247 |
| MJ_0924 | 19 | 19 | 0.92 | 20555393; 20507232; 20382770; 20159460; 20110296; 20081029; 20023016; 19332806; 19307219; 19184146 |
| MJ_0930 | 2 | 2 | 0.95 | 16427313; 15380647; 11007789; 10464323; 7606163 |
| MJ_0961 | 6 | 6 | 0.60 | 19001846; 12837750; 14676427; 12694531; 11606589; 11278932; 9366552; 9286856; 8626784; 8566808 |
| MJ_0973 | 1 | 5 | 0.84 | 17993606; 17519237; 17308787; 16962072; 16049108; 12740361; 12716056; 12501187; 12072441; 8444841 |
| MJ_0994 | 2 | 2 | 0.72 | 20798996; 20586119; 20485863; 20038586; 19965637; 19934113; 19805308; 19350426; 19336693; 19289130 |
| MJ_0999 | 12 | 12 | 0.89 | 19908377; 18976628; 18846552; 18313393; 18154740; 17151018; 17092293; 15808850; 15305922; 12796498 |
| MJ_1009 | 18 | 15 | 0.92 | 20707314; 20435726; 20404150; 20163565; 20139185; 20067779; 19898464; 19686042; 19500074; 19396753 |
| MJ_1015 | 18 | 18 | 0.96 | 19372157; 19184417; 18383009; 11309111; 18086187; 18007034; 17644517; 17497305; 9393684; 6309138 |
| MJ_1033 | 18 | 18 | 0.96 | 20519162; 20144229; 19890837; 19780400; 19346249; 17700703; 10748031; 17651435; 17619822; 3125980 |
| MJ_1037 | 1 | 8 | 0.50 | 20370823; 19848417; 19764814; 19682283; 19622649; 19430702; 19165722; 19138746; 20409813; 19028110 |
| MJ_1039 | 17 | 17 | 0.96 | 20643958; 20615963; 20613981; 20574006; 20545305; 20534440; 20512976; 20511223; 20507608; 20432247 |
| MJ_1048 | 13 | 13 | 0.90 | 20484520; 20427644; 20181627; 20156478; 20008374; 19965467; 19828446; 19625250; 19546037; 19450648 |
| MJ_1051 | 5 | 4 | 0.25 | 20813149; 20688828; 20583998; 20539941; 20534468; 20528947; 20526835; 20525265; 20470756; 20459317 |
| MJ_1055 | 19 | 19 | 0.94 | 20639341; 20573954; 20555393; 20547868; 20547742; 20541116; 20519506; 20507232; 20498265; 20483773 |
| MJ_1056 | 1 | 13 | 0.59 | 20717102; 20648627; 20639576; 20566639; 20561401; 20559852; 20485481; 20551969; 20469910; 20457752 |
| MJ_1059 | 19 | 19 | 0.63 | 20624909; 20584286; 20571951; 20555393; 20533395; 20522722; 20507232; 20504312; 20470049; 20458144 |
| MJ_1064 | 8 | 8 | 0.63 | 19548653; 18811190; 17897839; 17691836; 12391326; 17208523; 16843895; 15999435; 15950163; 15950159 |
| MJ_1069 | 19 | 19 | 0.97 | 20832776; 20522722; 20470363; 20385555; 20154620; 20008521; 19959475; 19858195; 19584677; 17517879 |
| MJ_1091 | 2 | 18 | 0.44 | 20533221; 19910527; 20219402; 19880598; 19764821; 19472373; 19251845; 19243433; 19219535; 19021770 |
| MJ_1100 | 19 | 8 | 0.85 | 20739710; 20530968; 20523973; 20512975; 20435850; 20416511; 20403181; 20392151; 20221814; 20216285 |
| MJ_1108 | 13 | 13 | 0.97 | 20433702; 20353103; 20160120; 18784368; 18611382; 10329163; 15526031; 16939209; 16866361; 15956377 |
| MJ_1109 | 14 | 14 | 0.45 | 20664931; 20647713; 20647710; 20567103; 20494472; 19724871; 19639195; 19638079; 19473832; 19457654 |
| MJ_1146 | 8 | 8 | 0.90 | 20847047; 20838239; 20833277; 20828617; 20824484; 20820924; 20820806; 20818827; 20818499; 20801607 |
| MJ_1148 | 17 | 6 | 0.80 | 20833695; 20644220; 20479947; 20436461; 20420710; 20231364; 20184719; 20178742; 20078850; 19948888 |
| MJ_1159 | 6 | 6 | 0.50 | 20686665; 20219464; 20164225; 19902245; 19884766; 19846885; 19670070; 19450514; 19403047; 12796301 |
| MJ_1200 | 6 | 6 | 0.79 | 20071747; 18931376; 19344311; 15725673; 17914173; 6254840; 9352933; 9332521; 9931007; 16368872 |
| MJ_1229 | 9 | 9 | 0.95 | 20615689; 20555365; 20437235; 20379951; 20230056; 20140210; 19965770; 19900410; 19875284; 19585972 |
| MJ_1237 | 12 | 12 | 0.91 | 20840452; 20812720; 20666976; 20661134; 20565992; 20534808; 20499085; 20461058; 20460155; 20427654 |
| MJ_1246 | 8 | 8 | 0.50 | 20558738; 19631775; 19527071; 9396791; 18486941; 18372246; 17697679; 17640871; 16637010; 16270230 |
| MJ_1267 | 18 | 18 | 0.95 | 15850393; 12039966; 11299317; 9778127; 2007142; 1718037 |
| MJ_1296 | 2 | 2 | 0.86 | 19768473; 15003564; 8791634; 17407324; 15265740; 11862544; 16897469; 16533066; 16195795; 16042606 |
| MJ_1297 | 2 | 9 | 0.97 | 20718468; 20693281; 20651329; 20601503; 20599697; 20599696; 20516619; 20507077; 20429931; 20394362 |
| MJ_1329 | 12 | 12 | 0.92 | 20716180; 20521764; 20363127; 20334618; 20207144; 20142044; 20038112; 20017927; 19892738; 19889537 |
| MJ_1334 | 19 | 19 | 0.68 | 20830297; 20724435; 20610614; 20523900; 20482518; 20435647; 20384302; 20335578; 20238176; 20199575 |
| MJ_1339 | 13 | 13 | 0.94 | 20592073; 20570652; 20540995; 20491942; 20403759; 20350613; 20097372; 20060503; 20033460; 19958136 |
| MJ_1366 | 14 | 14 | 0.92 | 20544509; 20380929; 20021999; 19918830; 19754463; 19186537; 19161981; 19131690; 18262821; 2483028 |
| MJ_1417 | 12 | 12 | 0.91 | 20597098; 20122386; 20092629; 19956677; 19907145; 19721064; 19701660; 19400780; 8525055; 19377968 |
| MJ_1438 | 2 | 2 | 0.93 | 20627200; 4874308; 12093296; 11939774; 10758367; 14898026; 9697817; 8416664; 3528129 |
| MJ_1482 | 8 | 8 | 0.79 | 19694966; 19690808; 19665153; 19590015; 18566795; 16865707; 17563835; 16684110; 16458304; 12855169 |
| MJ_1486 | 14 | 14 | 0.98 | 20082641; 19574646; 19570845; 19394344; 17333345; 17902044; 17896107; 12057663; 17064924; 17041099 |
| MJ_1494 | 12 | 15 | 0.69 | 20067779; 19789896; 14652736; 15037239; 6134587; 10377337; 15075374; 17892869; 16148945; 16953820 |
| MJ_1505 | 13 | 17 | 0.83 | 20339440; 20026132; 19002498; 17081564; 18439859; 18418076; 18165229; 9757830; 17449473; 10667799 |
| MJ_1529 | 6 | 6 | 0.97 | 20206583; 20154338; 18483064; 17875738; 17500045; 17486081; 17088260; 17016661; 10735851; 16247226 |
| MJ_1538 | 4 | 4 | 0.95 | 20670890; 20670172; 20547176; 20346373; 20337747; 20224894; 20220167; 20206249; 20170658; 20133196 |
| MJ_1569 | 2 | 8 | 0.31 | 9224702; 11470792; 11466310; 10482499; 284366; 1107805 |
| MJ_1597 | 1 | 8 | 0.37 | 20670920; 20589080; 20518745; 20403390; 20386493; 20308400; 20152942; 20113435; 20101025; 20093739 |
| MJ_1603 | 8 | 8 | 0.85 | 20499043; 20155483; 20054114; 19923736; 19744161; 19690365; 19420771; 19328460; 19214439; 19120610 |
| MJ_1616 | 14 | 14 | 0.91 | 20832515; 20823538; 20817636; 20808934; 20649757; 20571059; 20524113; 20502051; 20493773; 20491506 |
| MJ_1647 | 6 | 6 | 0.91 | 20512977; 20351259; 20197319; 20106979; 20070258; 19943916; 19745151; 19666563; 19594448; 19563100 |
| NT01GK0069 | 4 | 4 | 0.84 | 15231773; 11283287; 1569009; 10842753; 8784208 |
| NT01GK0086 | 2 | 2 | 0.95 | 20054118; 19389784; 365459; 2515994; 15500462; 1556094; 8096767; 1637823; 3117785; 2139795 |
| NT01GK0106 | 15 | 15 | 0.87 | 17954695; 19170879; 18326199; 8982462; 9390554; 12940983; 8830234; 1943780 |
| NT01GK0109 | 12 | 12 | 0.69 | 20388574; 20206397; 20141629; 20070525; 20038589; 19998280; 19933837; 19933364; 19931487; 19899961 |
| NT01GK0115 | 1 | 4 | 0.53 | 20830571; 20720017; 20696910; 20658158; 20541568; 20501661; 20494107; 20471956; 20430892; 20379751 |
| NT01GK0121 | 13 | 13 | 0.99 | 20668449; 19648245; 19133689; 15652481; 6095062; 10572144; 11085275; 15473684; 14586115; 11904182 |
| NT01GK0150 | 13 | 13 | 0.98 | 20696253; 20596249; 20554519; 20478248; 20087403; 19773262; 19377483; 19211306; 19191325; 17726054 |
| NT01GK0156 | 13 | 13 | 0.95 | 20554519; 20038335; 18948268; 19460470; 11467768; 18259060; 12626338; 15968096; 9671054; 16363795 |
| NT01GK0159 | 14 | 15 | 0.85 | 20836529; 20831657; 20824160; 20821240; 20807109; 20803137; 20738140; 20728507; 20715173; 20699230 |
| NT01GK0161 | 13 | 13 | 0.47 | 20818934; 20813183; 20813101; 20737003; 20734419; 20713520; 20713125; 20706274; 20699665; 20690163 |
| NT01GK0162 | 13 | 13 | 0.99 | 20410138; 19318571; 11085275; 10937989; 11985888; 16246363; 16116291; 15620217; 15593329; 14668217 |
| NT01GK0193 | 17 | 17 | 0.67 | 19906181; 19737356; 19564391; 19415331; 9139908; 19129643; 18682280; 18619465; 15520285; 18086212 |
| NT01GK0214 | 11 | 11 | 0.89 | 19821988; 19416360; 15616333; 10545172; 11409542; 7830551; 10574452; 6260376; 3031427; 7988893 |
| NT01GK0217 | 18 | 18 | 0.93 | 20662775; 20583551; 20826773; 20466942; 20463017; 20430871; 20332504; 20220205; 20203161; 20188670 |
| NT01GK0218 | 14 | 14 | 0.39 | 19545523; 19447883; 19021762; 18625239; 10694211; 18427783; 17890166; 16820147; 15820655; 15698963 |
| NT01GK0308 | 12 | 12 | 0.68 | 20813910; 20729362; 20688913; 20660111; 20660109; 20655344; 20618852; 20603018; 20602461; 20600107 |
| NT01GK0352 | 11 | 11 | 0.98 | 20705129; 20652620; 20602451; 20596755; 20585120; 20573216; 20547379; 20543074; 20521842; 20449818 |
| NT01GK0358 | 11 | 11 | 0.94 | 20844938; 20842711; 20815820; 20808959; 20803777; 20803249; 20737195; 20725145; 20717103; 20716342 |
| NT01GK0365 | 11 | 11 | 0.98 | 20705129; 20652620; 20602451; 20596755; 20585120; 20573216; 20547379; 20543074; 20521842; 20449818 |
| NT01GK0375 | 4 | 4 | 0.49 | 20226063; 18340633; 16469396; 12700254; 16870344; 16797026; 16411022; 16001258; 10792761; 10540314 |
| NT01GK0376 | 4 | 12 | 0.34 | 20118368; 10427003; 14990624; 14632074; 12576066; 12066855; 18975189; 9288362; 1735728 |
| NT01GK0381 | 18 | 18 | 0.97 | 20685374; 20677367; 20637607; 20601463; 20576035; 20561293; 20510655; 20490865; 20462759; 20410607 |
| NT01GK0424 | 18 | 18 | 0.86 | 19636250; 19129177; 18032408; 16870614; 16077079; 10570036; 15066148; 12857732; 12556450; 9245810 |
| NT01GK0472 | 19 | 4 | 0.40 | 20623345; 20552685; 20546602; 20527806; 20363864; 20308548; 20223832; 20016093; 19945288; 19901009 |
| NT01GK0480 | 19 | 8 | 0.32 | 20525558; 20516619; 20499649; 20403422; 20402656; 20345915; 20307580; 20206249; 20153185; 20148235 |
| NT01GK0490 | 5 | 5 | 0.93 | 20835839; 20822098; 20811471; 20700770; 20684242; 20675578; 20666399; 20619666; 20615224; 20614905 |
| NT01GK0503 | 6 | 6 | 0.91 | 20848659; 20846956; 20846459; 20843803; 20840885; 20837600; 20819074; 20816984; 20808930; 20808729 |
| NT01GK0557 | 18 | 4 | 0.84 | 20637256; 20356456; 20173000; 20078128; 19477183; 19298085; 19229492; 18355320; 17462542; 17090391 |
| NT01GK0592 | 6 | 6 | 0.87 | 20692326; 20601421; 20532169; 20511594; 20506501; 20448041; 20378654; 20371256; 20298190; 20038937 |
| NT01GK0636 | 11 | 11 | 0.89 | 20833810; 20671018; 20659166; 20597005; 20545844; 20478535; 20366304; 20118355; 20080651; 19995962 |
| NT01GK0660 | 8 | 8 | 0.50 | 20523973; 20512975; 20216285; 20024545; 19490731; 19465673; 19357119; 19339218; 19272306; 19235233 |
| NT01GK0681 | 4 | 8 | 0.47 | 20198924; 19855961; 19840761; 19807158; 19543858; 19473001; 19420728; 19378008; 18482597; 18414799 |
| NT01GK0693 | 8 | 8 | 0.94 | 18673073; 10550680; 10436930; 1707310; 1689724; 2833503; 3533929 |
| NT01GK0748 | 15 | 15 | 0.98 | 20730247; 20716550; 20678501; 20643656; 20162616; 20012281; 19586910; 19542281; 19501097; 18041902 |
| NT01GK0804 | 1 | 1 | 0.73 | 18679823; 12706338; 11814655; 10498721; 10587438; 9636022; 8418843; 1868065; 3440089; 6120060 |
| NT01GK0807 | 15 | 15 | 0.97 | 19376850; 16194239; 15228517; 11500437; 12826660; 6388494; 12147687; 10802742; 8550521; 9724717 |
| NT01GK0871 | 2 | 8 | 0.89 | 20546308; 20491655; 20432928; 20213113; 20208375; 20148520; 20083898; 20082374; 19902179; 19861415 |
| NT01GK0898 | 18 | 15 | 0.83 | 20616047; 20494996; 20405001; 20388705; 20348254; 20233629; 20190072; 20156195; 20061446; 20056829 |
| NT01GK0912 | 9 | 9 | 0.97 | 20418430; 20178986; 20158476; 20153183; 19664929; 19472174; 18804030; 18824113; 18721141; 2469386 |
| NT01GK0920 | 18 | 18 | 0.81 | 20582607; 20382765; 19388077; 19300437; 18824545; 14761996; 18273827; 18273810; 18226303; 11050157 |
| NT01GK0923 | 18 | 18 | 0.94 | 10572136; 15060731; 11341969; 2668876; 2664762; 1738314; 2821267 |
| NT01GK0927 | 15 | 15 | 0.89 | 20233935; 11325936; 19575568; 18428716; 18643936; 18315545; 18179421; 17997097; 15978081; 16659243 |
| NT01GK0970 | 8 | 8 | 0.93 | 20832396; 20699282; 20659890; 20637729; 20618457; 20516625; 20500879; 20405473; 20399281; 20378991 |
| NT01GK0996 | 11 | 6 | 0.91 | 20506501; 19019083; 18627532; 16802857; 16009137; 2839833; 10089316; 16514161; 15848228; 15839401 |
| NT01GK0999 | 8 | 8 | 0.61 | 20153846; 19153451; 18951917; 8441378; 15952775; 15742148; 6814937; 9891798; 12096814; 11581250 |
| NT01GK1018 | 18 | 18 | 0.73 | 20363758; 19576997; 19130269; 18702072; 18048940; 17975309; 10079817; 10212214; 15981243; 15269005 |
| NT01GK1045 | 18 | 18 | 0.68 | 20797386; 20219826; 20119820; 20107111; 20101282; 20100283; 20075140; 20017296; 20010100; 19965983 |
| NT01GK1053 | 18 | 18 | 0.90 | 20843810; 20826817; 20808924; 20799350; 20726333; 20691148; 20684594; 20683952; 20682982; 20666513 |
| NT01GK1069 | 8 | 6 | 0.75 | 20604506; 20120238; 20059735; 20034395; 19274447; 19157743; 19076303; 19018936; 17640961; 18789974 |
| NT01GK1108 | 9 | 9 | 0.99 | 20370610; 20178986; 20145708; 20099411; 20080211; 19686777; 19571038; 19525201; 19454733; 19444866 |
| NT01GK1115 | 15 | 15 | 0.80 | 19633194; 15802242; 12923168; 12777497; 12724394; 12079345; 11733988; 10802183; 10610766; 10537210 |
| NT01GK1150 | 9 | 9 | 0.73 | 20599697; 20308079; 20186809; 20111596; 19920185; 19880178; 19834068; 19756806; 19737935; 19631310 |
| NT01GK1181 | 8 | 8 | 0.43 | 20720174; 20581205; 20534478; 19833896; 19653301; 19625693; 20731274; 19054088; 18472627; 18401010 |
| NT01GK1204 | 4 | 12 | 0.58 | 20848653; 20848533; 20848231; 20847583; 20847447; 20847309; 20847183; 20847139; 20847002; 20846517 |
| NT01GK1212 | 8 | 8 | 0.97 | 20799369; 20698788; 20695266; 20667482; 20666312; 20660274; 20646165; 20627424; 20618079; 20617544 |
| NT01GK1213 | 8 | 12 | 0.44 | 20108890; 16146520; 18658027; 18511221; 2144363; 18445471; 18314130; 12402391; 12536251; 15837986 |
| NT01GK1242 | 19 | 19 | 0.98 | 20847002; 20843347; 20726582; 20713676; 20696872; 20608745; 20593835; 20580675; 20547785; 20542210 |
| NT01GK1266 | 4 | 4 | 0.84 | 20502438; 20453092; 20352045; 19903201; 19737354; 19698693; 19666580; 19654604; 19429628; 18588879 |
| NT01GK1293 | 18 | 18 | 0.92 | 19883725; 19726667; 10556024; 17584678; 15644331; 15308625; 15248763; 8940039; 12974640; 12949069 |
| NT01GK1305 | 15 | 12 | 0.70 | 20836557; 20713515; 20637108; 20554077; 20488890; 20176943; 19955427; 19898420; 19889877; 19879149 |
| NT01GK1338 | 19 | 4 | 0.82 | 20703316; 20691071; 20639318; 20615986; 20610778; 20586476; 20582761; 20576688; 20547100; 20543831 |
| NT01GK1339 | 8 | 8 | 0.63 | 19526370; 18692161; 18385479; 17891922; 15851411; 17640871; 17347254; 16637010; 16270230; 15986834 |
| NT01GK1346 | 12 | 12 | 0.98 | 20837023; 19801685; 17574901; 19108586; 18838376; 3030303; 9033594; 18069965; 17522969; 17464204 |
| NT01GK1354 | 4 | 4 | 0.86 | 19889085; 19665005; 12864857; 16677309; 15170399; 10320579; 10572114; 9286988; 2982790 |
| NT01GK1360 | 4 | 4 | 0.93 | 19919668; 19383688; 19376867; 18719175; 11528005; 17768253; 17875662; 11181966; 17238927; 10633114 |
| NT01GK1364 | 4 | 4 | 0.83 | 20693676; 20676082; 20543140; 20439729; 20346719; 20304988; 20203052; 20180908; 20133562; 20003186 |
| NT01GK1365 | 4 | 4 | 0.81 | 8312476; 10698740; 104961; 1400287; 1600102; 8369293; 2442165; 2832069; 4598031; 3510184 |
| NT01GK1379 | 4 | 4 | 0.24 | 16469702; 9194713; 8866475; 7893679; 4593496; 323255 |
| NT01GK1387 | 19 | 2 | 0.48 | 20476728; 19103164; 18790692; 11076526; 18323637; 18295483; 16831938; 17345630; 16420487; 15809342 |
| NT01GK1433 | 4 | 4 | 0.42 | 10606814; 7538190; 3127374; 6297377; 6783474; 6953305 |
| NT01GK1434 | 6 | 6 | 0.99 | 20817622; 20807205; 20724091; 20723756; 20722738; 20722443; 20713120; 20711416; 20705238; 20696249 |
| NT01GK1442 | 12 | 12 | 0.93 | 20824289; 20819954; 20682762; 20679547; 20679182; 20618771; 20156752; 20610394; 20608168; 20606106 |
| NT01GK1490 | 4 | 4 | 0.59 | 20198872; 20132838; 19959472; 19834620; 19690807; 19647513; 19636967; 19465484; 18424799; 19006334 |
| NT01GK1513 | 18 | 18 | 0.93 | 19429920; 19102629; 18452587; 11309499; 8749853; 17713961; 9802337; 8647345; 9827570; 16139519 |
| NT01GK1545 | 6 | 6 | 0.28 | 3006102; 9931007; 16549669; 11555298; 10417653 |
| NT01GK1587 | 14 | 14 | 0.65 | 20837458; 20831907; 20819423; 20809970; 20803296; 20714877; 20714149; 20683621; 20664931; 20653680 |
| NT01GK1591 | 8 | 8 | 0.95 | 20696867; 18945221; 1339433; 16292556; 16212409; 16126223; 15358267; 15345482; 15272169; 15045523 |
| NT01GK1597 | 1 | 8 | 0.48 | 20105320; 18718451; 18355283; 17768251; 11358996; 11700347; 11121489; 10878127; 10785395; 10651638 |
| NT01GK1646 | 15 | 15 | 0.77 | 20656782; 20223212; 20190050; 20184894; 20511236; 19966007; 19923210; 19751239; 19638175; 19191877 |
| NT01GK1651 | 18 | 18 | 0.72 | 12925133; 18639631; 12660233; 11300785; 10748025; 6994819 |
| NT01GK1652 | 18 | 18 | 0.91 | 20646353; 20220129; 20208170; 20121136; 20067302; 19906658; 19894768; 19744503; 19639462; 19575528 |
| NT01GK1677 | 4 | 8 | 0.63 | 20812717; 20653588; 20624669; 20607231; 20599794; 20556484; 20455565; 20408892; 20405842; 20390953 |
| NT01GK1688 | 12 | 12 | 0.89 | 12111745; 11027666; 9418238; 9070839; 8733236; 1502154; 8056296; 1368764; 2182621; 3292294 |
| NT01GK1701 | 8 | 8 | 0.64 | 20652236; 20547565; 20496344; 18823329; 18691525; 16535563; 16958750; 9187299; 8592705; 8081752 |
| NT01GK1704 | 8 | 8 | 0.34 | 20306314; 20138106; 19957200; 10715138; 19468838; 19382175; 19274445; 19261318; 19132837; 19002386 |
| NT01GK1718 | 8 | 8 | 0.98 | 20816850; 20691393; 20646165; 20618079; 20599983; 20357218; 20067779; 19496430; 19324096; 19138661 |
| NT01GK1731 | 5 | 8 | 0.55 | 20663881; 20547872; 20477872; 20392697; 20387629; 20135153; 20099848; 20060433; 19891443; 19864628 |
| NT01GK1742 | 12 | 4 | 0.37 | 20818668; 20817675; 20639339; 20454456; 20417640; 20142038; 19809258; 19596042; 18713320; 18400936 |
| NT01GK1762 | 19 | 19 | 0.93 | 17588176; 17244817; 16476725; 16162506; 10727942; 8380148; 15130128; 14617152; 10471558; 11171944 |
| NT01GK1787 | 9 | 9 | 1.00 | 20511508; 20463028; 20167774; 20110695; 20035485; 19903863; 19659692; 19452559; 19213219; 19101656 |
| NT01GK1854 | 18 | 18 | 0.90 | 19919002; 19717637; 19597156; 19470745; 15200952; 11017087; 17711302; 17662307; 17597647; 8599111 |
| NT01GK1887 | 18 | 18 | 0.98 | 20802252; 20634426; 20615993; 20516077; 20472741; 20435089; 20406814; 20348389; 20126460; 20109468 |
| NT01GK1903 | 4 | 4 | 0.47 | 18980183; 12596216; 2169873; 9802010; 8621083; 3312478 |
| NT01GK1914 | 17 | 18 | 0.35 | 20545848; 20404271; 20237321; 20163657; 20134145; 20057163; 20055789; 19948795; 19901068; 19886928 |
| NT01GK1962 | 18 | 18 | 0.95 | 20815391; 20709853; 20684227; 20674437; 20639578; 20627949; 20610766; 20609358; 20606288; 20599686 |
| NT01GK2036 | 12 | 12 | 0.99 | 20507874; 20410261; 20224257; 20140974; 20067154; 19580842; 19458713; 18979629; 18690782; 18338781 |
| NT01GK2041 | 12 | 4 | 0.37 | 20818668; 20817675; 20639339; 20454456; 20417640; 20142038; 19809258; 19596042; 18713320; 18400936 |
| NT01GK2086 | 8 | 8 | 0.33 | 20809958; 20714780; 20687341; 20641017; 20519920; 20406496; 20012280; 19572128; 17280608; 19128960 |
| NT01GK2087 | 8 | 8 | 0.67 | 20809958; 20730758; 20718290; 20541500; 20541506; 20406496; 20349329; 20158272; 20126731; 20054649 |
| NT01GK2098 | 8 | 8 | 0.90 | 20845334; 20726783; 20695200; 20673213; 20643807; 20630171; 20511553; 20445268; 20392151; 20362419 |
| NT01GK2109 | 8 | 8 | 0.75 | 15678175; 11580920; 7845353; 9802033; 9322760; 1761221; 7952181; 7836662; 1761221; 3496810 |
| NT01GK2113 | 19 | 19 | 0.70 | 20803478; 20798166; 20729204; 20696204; 20659891; 20639194; 20517582; 20511219; 20504027; 20492525 |
| NT01GK2116 | 8 | 8 | 0.84 | 19346355; 19151970; 16606456; 17965859; 11690647; 17619876; 17137296; 14730129; 8435847; 12699688 |
| NT01GK2121 | 18 | 18 | 0.43 | 19633083; 10334982; 11260467; 10064713; 8878033; 2196376; 1694248; 6088507; 6358208 |
| NT01GK2138 | 5 | 5 | 0.96 | 20635345; 20631318; 20207756; 19476442; 18767150; 18682379; 14521881; 17309280; 7065255; 1624427 |
| NT01GK2167 | 1 | 8 | 0.97 | 20846325; 20818499; 20684326; 20664702; 20629101; 20625650; 20621659; 20570330; 20522323; 20476564 |
| NT01GK2191 | 18 | 18 | 0.99 | 20808924; 20553499; 20552428; 20548943; 20548793; 20515952; 20471400; 20460823; 20454684; 20439763 |
| NT01GK2200 | 9 | 9 | 0.96 | 20846857; 20845073; 20844923; 20844260; 20844007; 20843997; 20843949; 20842343; 20837870; 20836990 |
| NT01GK2225 | 18 | 18 | 0.99 | 20102440; 19484827; 18215300; 19004000; 18252722; 16714605; 11872826; 17387526; 12829271; 1393837 |
| NT01GK2247 | 8 | 8 | 0.59 | 20818827; 20702706; 20690680; 20684524; 20660274; 20660227; 20658209; 20650544; 20645828; 20643956 |
| NT01GK2251 | 9 | 8 | 0.52 | 20625717; 20195860; 18060402; 1416617; 7875566; 7409145 |
| NT01GK2304 | 8 | 8 | 0.55 | 20398623; 20332082; 20303979; 20077566; 19730970; 19652354; 10816041; 19438211; 19265402; 19096104 |
| NT01GK2327 | 8 | 8 | 0.43 | 20447408; 20394349; 20299676; 20195697; 20159657; 20106978; 19942659; 19923219; 19921396; 19888457 |
| NT01GK2328 | 4 | 8 | 0.28 | 20848677; 20848674; 20848673; 20848668; 20848659; 20848653; 20848643; 20848642; 20848615; 20848614 |
| NT01GK2338 | 18 | 12 | 0.28 | 20838844; 20832055; 20814784; 20805943; 20804978; 20801427; 20723603; 20715530; 20706061; 20701746 |
| NT01GK2351 | 15 | 15 | 0.53 | 19170879; 17275217; 17434531; 17268768; 11284699; 15870478; 15702929; 1311296 |
| NT01GK2358 | 18 | 18 | 0.55 | 17715129; 15350125; 10854277; 10744628; 9693722; 8119910; 8294906; 1567896; 2509241 |
| NT01GK2370 | 15 | 15 | 0.93 | 20581213; 19007420; 18052213; 10679470; 16524589; 15807530; 10346814; 14596799; 10551881; 6776094 |
| NT01GK2371 | 8 | 8 | 0.92 | 20567220; 20519569; 20359473; 20299676; 20335578; 20299676; 20348403; 20213376; 20151200; 20075179 |
| NT01GK2373 | 8 | 8 | 0.90 | 20830443; 20817811; 20725869; 20696150; 20528923; 20434983; 20405025; 20333764; 20299676; 20237579 |
| NT01GK2400 | 1 | 1 | 0.76 | 20441465; 20383010; 20227442; 20120471; 20072996; 19857083; 19638492; 19362203; 19334390; 19106740 |
| NT01GK2465 | 8 | 8 | 0.80 | 20836675; 20832300; 20824725; 20822851; 20737924; 20730882; 20728346; 20724951; 20693115; 20669055 |
| NT01GK2474 | 19 | 19 | 0.96 | 20497503; 20497502; 20400548; 18490448; 16475801; 14973022; 10369758; 15943817; 10094698; 14534570 |
| NT01GK2551 | 19 | 4 | 0.34 | 20434576; 19189487; 16512741; 4019413; 7892217; 8936302; 1629150; 3142866; 1848202 |
| NT01GK2624 | 8 | 8 | 0.51 | 20382813; 19847916; 19222573; 6250450; 15247105; 18725313; 17349085; 17259608; 17077990; 15966321 |
| NT01GK2629 | 9 | 9 | 0.34 | 20576599; 19923005; 19766560; 19766572; 19653693; 19596859; 19443544; 19383699; 18991142; 18831553 |
| NT01GK2660 | 12 | 4 | 0.37 | 20818668; 20817675; 20639339; 20454456; 20417640; 20142038; 19809258; 19596042; 18713320; 18400936 |
| NT01GK2682 | 4 | 4 | 0.81 | 20846513; 20837555; 20815357; 20815087; 20811880; 20809078; 20804771; 20799971; 20740651; 20732406 |
| NT01GK2684 | 4 | 4 | 0.61 | 11298275; 17630974; 16009134; 16009133; 15632435; 9931458; 15083514; 12123453; 11744713; 11298275 |
| NT01GK2691 | 8 | 9 | 0.35 | 20568559; 20487577; 20472448; 20465544; 20458967; 20413507; 20405948; 20375417; 20375199; 20363943 |
| NT01GK2736 | 17 | 17 | 0.92 | 20595001; 20545305; 20453093; 20354151; 20227844; 20087629; 20025658; 20067173; 20070531; 19951358 |
| NT01GK2746 | 15 | 18 | 0.57 | 20846107; 20839184; 20832861; 20830601; 20828370; 20828160; 20826700; 20826340; 20825181; 20821471 |
| NT01GK2749 | 4 | 4 | 0.54 | 20839945; 20833892; 20831407; 20817757; 20805502; 20803087; 20802044; 20713519; 20709900; 20709422 |
| NT01GK2774 | 4 | 8 | 0.55 | 6447794; 16209911; 11178251; 9813127; 9746358; 7565874; 7677991; 6269067 |
| NT01GK2776 | 1 | 1 | 0.22 | 19954230; 19576563; 17185548; 12515465; 10037775; 7565611; 9085573; 9003320; 8621661; 7022140 |
| NT01GK2805 | 17 | 15 | 0.61 | 20844554; 20844019; 20838584; 20828602; 20826955; 20818439; 20817769; 20816986; 20808803; 20807197 |
| NT01GK2860 | 2 | 1 | 0.36 | 18481057; 17575227; 17135341; 16534983; 16840914; 10438489; 16428494; 14742742; 12939586; 10684605 |
| NT01GK2887 | 3 | 3 | 1.00 | 20738375; 20714501; 20708667; 20616068; 20542929; 20472801; 20439474; 20349311; 20147289; 20080557 |
| NT01GK2898 | 2 | 2 | 0.76 | 20350571; 18997292; 15326348; 18613201; 7925353; 18232714; 16499613; 15705579; 15554970; 12882547 |
| NT01GK2914 | 12 | 12 | 0.65 | 20530261; 19154314; 18087630; 16199566; 12775833; 9420330; 10524213; 10551365; 9425059; 9266683 |
| NT01GK2934 | 8 | 8 | 0.88 | 20695216; 20693447; 20653233; 20696374; 20583616; 20583550; 20544938; 20544915; 20540712; 20517892 |
| NT01GK3034 | 15 | 15 | 0.70 | 20093290; 20012992; 19959580; 19447950; 15353566; 11923293; 15299374; 9301332; 9426139; 8780507 |
| NT01GK3046 | 9 | 8 | 0.67 | 10329704; 11734569; 11330072; 10660057; 2173486; 2781417; 6166327 |
| NT01GK3063 | 8 | 9 | 0.32 | 20803137; 20140210; 19418221; 19366370; 17428661; 17891922; 9250661; 10888843; 10843999; 16981708 |
| NT01GK3066 | 2 | 2 | 0.61 | 20226884; 20054882; 8286370; 18311129; 18284573; 10544286; 11524134; 15101978; 12738849; 12076669 |
| NT01GK3105 | 12 | 12 | 0.90 | 20819954; 18783432; 15749831; 7039409; 15368846; 12176387; 2231712; 7528082 |
| NT01GK3130 | 18 | 18 | 0.99 | 20662775; 20454684; 19760129; 11435397; 19594831; 19590146; 16885437; 18041902; 19398546; 19302333 |
| NT01GK3138 | 18 | 18 | 1.00 | 20808924; 20805402; 20662775; 20656493; 20454456; 20439763; 20412802; 20408914; 20399185; 20203106 |
| NT01GK3165 | 8 | 8 | 0.62 | 20143043; 20133651; 19954230; 19850488; 19635803; 19446917; 19376100; 19192390; 19140736; 19002717 |
| NT01GK3206 | 14 | 6 | 0.90 | 20519935; 20512117; 20298437; 20159164; 20146357; 20042602; 19785038; 20683475; 16251276; 10834833 |
| NT01GK3224 | 4 | 4 | 0.89 | 20829360; 20728562; 20649474; 20627362; 20626112; 20591624; 20583283; 20582873; 20528770; 20387225 |
| NT01GK3275 | 2 | 8 | 0.47 | 20707865; 20707605; 20534555; 20450902; 20089862; 19924289; 19863063; 10891285; 19684135; 19686777 |
| NT01GK3318 | 9 | 9 | 0.98 | 20825197; 20736083; 20640873; 20555380; 20541551; 20511508; 20463028; 20463021; 20452977; 20359329 |
| NT01GK3328 | 8 | 8 | 0.80 | 20803137; 20715123; 20618950; 20520732; 20463368; 20434234; 20233922; 19920149; 19828084; 19752030 |
| NT01GK3336 | 18 | 18 | 0.95 | 19717637; 19597156; 19470745; 8599111; 17297656; 16911515; 16668048; 15850393; 11442842; 11123914 |
| NT01GK3339 | 15 | 15 | 0.97 | 20133363; 18611278; 16952958; 18039772; 18022383; 14645274; 10464216; 10564501; 11191810; 1901616 |
| NT01GK3398 | 12 | 13 | 0.53 | 20505670; 20403364; 20392853; 20350931; 20097205; 20052680; 20033464; 20007323; 19933321; 19847269 |
| NT01GK3402 | 12 | 4 | 0.37 | 20818668; 20817675; 20639339; 20454456; 20417640; 20142038; 19809258; 19596042; 18713320; 18400936 |
| NT01GK3415 | 4 | 4 | 0.92 | 20545753; 20519916; 20339018; 20060643; 19575436; 19567265; 19174888; 19173857; 19120462; 18949750 |
| NT01GK3419 | 4 | 4 | 0.79 | 19965938; 19916218; 19737354; 19376877; 19246756; 18682691; 18573177; 18249907; 17642474; 16980445 |
| NT01GK3430 | 4 | 4 | 0.90 | 20663714; 20500872; 20418380; 20418156; 20363938; 20185511; 20180908; 20108326; 20047678; 19778998 |
| NT01GK3443 | 19 | 19 | 0.62 | 10829079; 9163424; 11390676; 10865948; 9317046; 8529891 |
| NT01GK3453 | 4 | 4 | 0.79 | 20639318; 17492271; 1915293; 10320579; 7770032; 8158647 |
| NT01GK3454 | 4 | 4 | 0.84 | 20581225; 18050911; 12401220; 10320579; 9742694; 9168617; 7770032; 16557718; 8407873; 2404955 |
| NT01GK3457 | 4 | 4 | 0.89 | 20845370; 20831410; 20819931; 20814576; 20808885; 20808867; 20731882; 20729351; 20728476; 20711895 |
| NT01GK3461 | 4 | 4 | 0.72 | 9426140; 16300911; 10049798; 11913797; 11204784; 10066477; 8041739; 9387224 |
| NT01GK3484 | 19 | 19 | 0.50 | 20309546; 19160029; 19129185; 17900153; 17725726; 17222909; 17174333; 17005181; 10470028; 16284181 |
| NT01GK3517 | 6 | 6 | 0.63 | 20433692; 20389284; 20305446; 20156453; 20140874; 20017296; 19945430; 19920138; 19779462; 8816793 |
| NT01GK3529 | 15 | 15 | 0.99 | 20583594; 20545348; 20466770; 20332492; 20332481; 20211838; 20190084; 20178981; 20167624; 19966017 |
| NT01GK3542 | 4 | 19 | 0.97 | 20823533; 20822875; 20812985; 20804758; 20739278; 20713411; 20709893; 20709844; 20698522; 20686915 |
| NT01GK3575 | 18 | 18 | 0.80 | 18349697; 18310123; 18303017; 16763184; 291007; 15579558; 12949075; 12546731; 11307608; 9667011 |
| NT01GK3592 | 6 | 6 | 0.75 | 20110566; 18534842; 16945962; 9234711; 16094458; 17704061; 17531976; 10716434; 9572142; 12400016 |
| NT01GK3644 | 19 | 8 | 0.85 | 20816491; 20678902; 20615386; 20237670; 20211750; 19887444; 19836401; 19751718; 19719175; 19652355 |
| NT01GK3652 | 19 | 8 | 0.27 | 20848296; 20847938; 20847272; 20847002; 20846957; 20846911; 20846368; 20823714; 20845092; 20844939 |
| NT01GK3658 | 4 | 4 | 0.91 | 20639318; 20199595; 20154133; 20132451; 19376867; 18931786; 18811728; 18483484; 11244064; 11528005 |
| NT01GK3659 | 4 | 4 | 0.92 | 20132451; 19376867; 18957286; 11528005; 17090391; 16720646; 1631122; 15699192; 15276839; 15136044 |
| NT01GK3666 | 18 | 5 | 0.42 | 16332890; 19581367; 18830684; 12001169; 17934697; 16922681; 16364322; 15065880; 7616962; 8360921 |
| NT01GK3668 | 4 | 19 | 0.82 | 20677902; 20606279; 20572278; 20454963; 20435778; 20224955; 20128906; 20080736; 20061761; 20024793 |
| NT01GK3738 | 5 | 5 | 0.98 | 20563878; 10785653; 16362287; 15752699; 15388942; 14648699; 2079625; 11804860; 8088547 |
| NT01GK3781 | 19 | 8 | 0.57 | 20711572; 20680564; 20679207; 20435476; 20427415; 20416079; 20400534; 20398236; 20391781; 20382410 |
| NT01GK3796 | 8 | 8 | 0.90 | 19734178; 19584547; 19577535; 18582433; 17176101; 6299895; 12578386; 12530544; 12446663; 12136145 |
| NT01GK3799 | 8 | 8 | 0.87 | 19734178; 6299895; 12578386; 10216161; 9737851; 9428682; 8618820; 8050583; 1322173; 1848234 |
| NT01GK3801 | 13 | 13 | 0.99 | 1058472; 17150753; 16963456; 15637073; 15062082; 12903321; 12836250; 1310418; 9660928; 7155893 |
| NT01GK3843 | 13 | 13 | 0.90 | 20463973; 20410297; 19590924; 18292779; 10572144; 10198119; 16025162; 2488477; 14585508; 14559179 |
| NT01GKA0001 | 6 | 6 | 0.61 | 20709099; 20435728; 19684126; 19640422; 19601964; 15852513; 2438417; 19481568; 19440202; 19423399 |
| NT01GKA0010 | 6 | 6 | 0.90 | 18931376; 19370021; 19286788; 19116772; 17914173; 18838147; 17126598; 18062262; 14075095; 14550943 |
| NT01NH0014 | 8 | 8 | 0.63 | 20599671; 20511298; 19486643; 15547280; 17543971; 17459874; 12496199; 16807925; 16289704; 15918884 |
| NT01NH0018 | 11 | 11 | 0.94 | 20844938; 20842711; 20815820; 20808959; 20803777; 20803249; 20737195; 20725145; 20717103; 20716342 |
| NT01NH0027 | 8 | 15 | 0.52 | 20632947; 20043234; 16839881; 19683668; 19561131; 19395490; 18565104; 18431568; 18051755; 18031348 |
| NT01NH0048 | 9 | 9 | 0.85 | 20818586; 20698542; 20666435; 20566771; 20555380; 20503440; 20452977; 20335945; 20200152; 20158608 |
| NT01NH0064 | 8 | 8 | 0.47 | 20521767; 20130996; 20077007; 19749040; 19717605; 19468858; 17981156; 19346304; 18197706; 19272352 |
| NT01NH0067 | 6 | 6 | 0.96 | 20722738; 19620647; 19542287; 19395381; 19129187; 12634335; 17499012; 10357855; 9397680; 15099822 |
| NT01NH0076 | 6 | 6 | 0.65 | 3118156; 15563835; 12628247; 12872134; 10369684; 11866515; 11183780; 10930420; 1459953; 10570977 |
| NT01NH0095 | 12 | 12 | 0.79 | 19801361; 19185782; 19038348; 15101818; 15113693; 18760830; 17850309; 17084158; 16360718; 19791424 |
| NT01NH0098 | 15 | 15 | 0.60 | 20643148; 20532736; 20493877; 20363937; 20110304; 19648236; 19542280; 19482921; 19438718; 19131333 |
| NT01NH0101 | 9 | 9 | 0.95 | 20547355; 19103602; 17428661; 18704089; 18702504; 17609944; 16061252; 15751987; 15638818; 15380819 |
| NT01NH0122 | 8 | 8 | 0.88 | 20525253; 16806221; 16114877; 15743763; 12164810; 12095696; 9406381; 9631507; 9261082 |
| NT01NH0125 | 11 | 11 | 0.94 | 20844938; 20842711; 20815820; 20808959; 20803777; 20803249; 20737195; 20725145; 20717103; 20716342 |
| NT01NH0137 | 12 | 12 | 0.86 | 20512970; 19569227; 19028503; 9605324; 16169560; 12475171; 9928475; 10807917; 11101880; 9184221 |
| NT01NH0138 | 18 | 18 | 0.88 | 12574118; 14605210; 15618217; 14981507; 9644977; 10393182; 8253836; 9367739 |
| NT01NH0142 | 12 | 12 | 0.97 | 20837023; 19801685; 17574901; 19108586; 18838376; 3030303; 9033594; 18069965; 17522969; 17464204 |
| NT01NH0176 | 18 | 18 | 0.93 | 20844142; 20839018; 20837704; 20817064; 20810899; 20739660; 20727371; 20723758; 20713020; 20711351 |
| NT01NH0190 | 8 | 8 | 0.89 | 17576516; 15388943; 10437801; 7584858; 2558656; 6288108; 6254527 |
| NT01NH0219 | 17 | 17 | 0.24 | 20810747; 20739603; 20713509; 20705233; 20677811; 20639884; 20620951; 20616011; 20615407; 20603000 |
| NT01NH0261 | 17 | 17 | 0.79 | 19542278; 19111618; 19076234; 11374870; 9393439; 8596456 |
| NT01NH0282 | 9 | 12 | 0.85 | 20346169; 9925622; 19561190; 11004196; 16030206; 17543409; 15060050; 16233560; 2644188; 10849810 |
| NT01NH0307 | 2 | 4 | 0.36 | 20809345; 20805291; 20712757; 20706790; 20696779; 20681784; 20673128; 20655259; 20637195; 20633529 |
| NT01NH0334 | 15 | 15 | 0.91 | 20093290; 9987125; 9301332; 8227007; 7236614; 1899859 |
| NT01NH0335 | 15 | 15 | 0.96 | 20378989; 20348304; 20168997; 20089442; 19958380; 13838951; 15225317; 19400808; 19191877; 19151927 |
| NT01NH0338 | 2 | 2 | 0.63 | 20798996; 20553774; 20510440; 20510016; 20490928; 20485863; 20442414; 20427485; 20406759; 20153251 |
| NT01NH0349 | 11 | 11 | 0.98 | 19899628; 18815788; 12524543; 18253751; 18069343; 17459613; 17039376; 10852890; 15772148; 15771247 |
| NT01NH0351 | 11 | 11 | 0.95 | 20709901; 20698795; 20692326; 20627350; 20603069; 20578458; 20556509; 20546690; 20494389; 20485515 |
| NT01NH0383 | 11 | 6 | 0.76 | 20007596; 19425495; 19334532; 11600708; 19013430; 18838147; 11407914; 17069852; 16649659; 15554191 |
| NT01NH0386 | 6 | 6 | 0.90 | 20178992; 20157002; 19580006; 18922464; 18606573; 10454599; 17378750; 9512524; 15738391; 10585965 |
| NT01NH0435 | 18 | 18 | 0.59 | 20074554; 19751701; 19687137; 19669810; 19667201; 19453276; 19409873; 19396940; 19366611; 19286473 |
| NT01NH0449 | 1 | 1 | 0.94 | 20821054; 20817755; 20808328; 20802128; 20801605; 20797482; 20724492; 20720171; 20718043; 20717016 |
| NT01NH0450 | 1 | 1 | 0.82 | 20831840; 20724652; 20672747; 20670473; 20637366; 20634689; 20592457; 20589880; 20580032; 20552676 |
| NT01NH0467 | 11 | 11 | 0.98 | 20659166; 20133842; 20060554; 20056615; 19962991; 19897657; 19841094; 19788336; 19776717; 19706522 |
| NT01NH0472 | 8 | 8 | 0.92 | 19220469; 16497924; 15196036; 6203738; 11751685; 10430588; 10772918; 10342853; 9237995; 9187299 |
| NT01NH0484 | 12 | 4 | 0.61 | 20605976; 20546155; 20522495; 20510344; 20463311; 20421386; 20233301; 20088373; 20056182; 19853923 |
| NT01NH0516 | 19 | 19 | 0.96 | 20347067; 20188057; 20161474; 19056279; 18952861; 16218944; 10543788; 18499663; 18325534; 18210176 |
| NT01NH0524 | 18 | 18 | 0.92 | 19187221; 10940570; 10696483; 2118530; 8132494 |
| NT01NH0534 | 18 | 18 | 0.36 | 20441802; 20430871; 20427472; 20395263; 20219227; 20217429; 20038785; 19556451; 19473983; 19419999 |
| NT01NH0535 | 18 | 18 | 0.98 | 20208152; 19818021; 19234723; 19101549; 1534409; 18511655; 18324396; 11602359; 19356060; 17322901 |
| NT01NH0545 | 15 | 15 | 0.34 | 20487301; 20436965; 20139185; 20097688; 20059691; 19858294; 19824696; 19672046; 19651770; 12837773 |
| NT01NH0547 | 15 | 15 | 0.99 | 20418395; 17462014; 10940042; 2251112; 16593648 |
| NT01NH0552 | 6 | 19 | 0.25 | 20733041; 20687505; 20646988; 20646738; 20627395; 20616014; 20569222; 20530153; 20500698; 20473331 |
| NT01NH0572 | 13 | 8 | 0.24 | 20691096; 20644147; 20633049; 20589823; 20486243; 20350137; 20348291; 20151707; 20145952; 20136357 |
| NT01NH0582 | 2 | 2 | 0.94 | 19933361; 19022396; 17950386; 7009558; 4333375; 15792955; 12440154; 10777520; 10760477; 10419476 |
| NT01NH0588 | 14 | 14 | 0.95 | 20838591; 20823277; 20817725; 20810304; 20801231; 20696207; 20693661; 20678196; 20664794; 20658215 |
| NT01NH0606 | 12 | 12 | 0.97 | 20827447; 20668094; 20615403; 20583963; 20525865; 20504766; 20499929; 20494979; 20487289; 20448033 |
| NT01NH0641 | 12 | 12 | 0.93 | 18788473; 18614331; 17456188; 15726567; 15283695; 12631144; 11741588; 11408480; 11115631; 10966643 |
| NT01NH0643 | 14 | 14 | 0.49 | 20394834; 19081844; 16900379; 16378245; 15542780; 15489502; 15291552; 10373420; 12773105; 10094930 |
| NT01NH0662 | 8 | 8 | 0.27 | 19635791; 17724028; 10617337; 16504786; 16481318; 15847154; 15597737; 14643893; 12923788; 11781092 |
| NT01NH0671 | 6 | 6 | 0.95 | 20645042; 20603158; 20530711; 20443037; 20407424; 20360008; 20354588; 20299199; 20195511; 20116856 |
| NT01NH0676 | 8 | 13 | 0.61 | 20693323; 20507077; 20448982; 20232292; 20221531; 20138838; 20105319; 20059914; 19931596; 19884769 |
| NT01NH0696 | 2 | 2 | 0.57 | 16907720; 9004500; 15311923; 10966576; 8501034; 1374146; 14251214; 6327463 |
| NT01NH0706 | 6 | 6 | 0.77 | 20179009; 20154359; 20111866; 20110566; 20075079; 19956593; 19833762; 19692572; 10686097; 18544633 |
| NT01NH0708 | 6 | 6 | 0.83 | 19332813; 19070898; 18663525; 15247157; 14656901; 12598537; 12507474; 11686531; 9480766; 10473086 |
| NT01NH0711 | 6 | 19 | 0.55 | 20832886; 20699399; 20603706; 20304912; 20040061; 19909380; 19908039; 19861655; 19703229; 19702874 |
| NT01NH0740 | 2 | 8 | 0.38 | 19649727; 16700064; 19130090; 18804703; 18801628; 18767138; 18597647; 18309272; 17417062; 16123074 |
| NT01NH0743 | 2 | 19 | 0.48 | 20017145; 19954226; 19897889; 19852513; 19699210; 18699781; 17658461; 9792721; 15722149; 12953204 |
| NT01NH0758 | 19 | 19 | 0.79 | 19304851; 18701463; 15774864; 16870450; 10542235; 10383966; 10564478; 11320055; 8772200; 8830253 |
| NT01NH0759 | 13 | 13 | 0.93 | 19184079; 15289330; 17728076; 17714511; 17430924; 17099150; 16805315; 16797058; 16516201; 16452584 |
| NT01NH0778 | 1 | 1 | 0.89 | 15882622; 11259585; 18398873; 18266921; 17680699; 17261589; 9878407; 11395417; 15889412; 11852094 |
| NT01NH0812 | 6 | 6 | 0.81 | 20845485; 20820873; 20736948; 20732909; 20729302; 20729138; 20725792; 20717836; 20686820; 20682983 |
| NT01NH0816 | 4 | 4 | 0.86 | 19376877; 19246756; 18573177; 16980445; 10048040; 2180916; 16416128; 15752189; 9864327; 11763238 |
| NT01NH0820 | 18 | 18 | 0.98 | 20832450; 20817004; 20809990; 20807802; 20798321; 20735425; 20713739; 20702707; 20699482; 20698320 |
| NT01NH0839 | 1 | 1 | 0.95 | 19428471; 11842149; 18285355; 18208521; 18184691; 18050920; 16535512; 17981822; 16007982; 17617175 |
| NT01NH0866 | 18 | 18 | 0.94 | 20817004; 20661830; 20620158; 20566566; 20530417; 20444207; 20426829; 20403880; 20360246; 20124415 |
| NT01NH0891 | 4 | 4 | 0.79 | 20846581; 20846362; 20843678; 20831651; 20831592; 20829117; 20828376; 20823200; 20815824; 20813843 |
| NT01NH0908 | 12 | 12 | 0.96 | 20709850; 20304034; 19961020; 19891527; 19624706; 236308; 19825551; 17980349; 17941091; 17482513 |
| NT01NH0917 | 18 | 18 | 0.94 | 19627500; 10485884; 17927700; 17628143; 17578453; 17238922; 8344918; 11844775; 15918073; 15612934 |
| NT01NH0937 | 2 | 2 | 0.94 | 20675471; 19858196; 19760906; 19740970; 19523068; 18388403; 18296573; 3114257; 17851041; 17224143 |
| NT01NH0946 | 12 | 12 | 0.90 | 19726681; 18835567; 17306546; 16135238; 15037248; 12235156 |
| NT01NH0951 | 13 | 12 | 0.29 | 20840813; 20644147; 20633049; 20589823; 20534395; 20528185; 20495806; 20473876; 20456288; 20378691 |
| NT01NH0958 | 9 | 9 | 1.00 | 20511508; 20463028; 20167774; 20110695; 20035485; 19903863; 19659692; 19452559; 19213219; 19101656 |
| NT01NH1022 | 11 | 11 | 0.98 | 20444095; 20034649; 19822340; 19493107; 19349519; 19352408; 16116440; 3547406; 6989837; 17850749 |
| NT01NH1024 | 11 | 11 | 1.00 | 20725145; 20603069; 20585120; 20562304; 20547379; 20543074; 20538004; 20512402; 20512353; 20502527 |
| NT01NH1065 | 9 | 9 | 0.91 | 20800575; 20555380; 20541551; 20452977; 20443682; 20428080; 20408801; 20202875; 20200152; 20158608 |
| NT01NH1100 | 11 | 11 | 0.99 | 20709901; 20467813; 20008174; 20007650; 19941653; 19921332; 19675097; 19624407; 19592535; 14617171 |
| NT01NH1129 | 8 | 8 | 0.65 | 19426674; 18824004; 18647626; 9806957; 16773741; 16376852; 16199472; 16042413; 15531838; 12852784 |
| NT01NH1130 | 8 | 8 | 0.87 | 20694448; 20648626; 20601653; 20374897; 20154043; 19889600; 19862842; 19816789; 19765774; 19624744 |
| NT01NH1134 | 8 | 8 | 0.67 | 20003495; 19811085; 19642171; 19628287; 19569981; 19422269; 19258054; 9867845; 18715687; 18666252 |
| NT01NH1149 | 8 | 11 | 0.99 | 10952608; 10413660; 10565924; 9603867; 8210675; 11064191; 8820651; 10564481; 9642176; 17794571 |
| NT01NH1154 | 4 | 4 | 0.27 | 20335172; 17552011; 17239812; 15664987; 12553441; 12621086; 12144348; 11200544 |
| NT01NH1202 | 8 | 8 | 0.97 | 20810270; 20737003; 20622440; 20516492; 20513475; 20431245; 20387665; 20371487; 20335826; 20094655 |
| NT01NH1215 | 1 | 8 | 0.85 | 20847424; 20846325; 20831907; 20829287; 20823560; 20820739; 20819649; 20813141; 20813101; 20808573 |
| NT01NH1227 | 18 | 18 | 0.83 | 20826797; 20816962; 20427472; 20015946; 19955819; 19805644; 19741015; 19460820; 19439519; 19386990 |
| NT01NH1229 | 18 | 18 | 0.99 | 20844539; 20814153; 20713739; 20709757; 20684243; 20668204; 20659170; 20657593; 20656864; 20624916 |
| NT01NH1273 | 11 | 11 | 0.57 | 20602451; 20449818; 10973072; 2174354; 18612245; 11169193; 16843620; 16164610; 15699199; 12753585 |
| NT01NH1279 | 12 | 18 | 0.47 | 20656905; 20472799; 20363945; 20362281; 20333253; 20306492; 20303353; 20199604; 20185511; 20172994 |
| NT01NH1289 | 19 | 19 | 0.87 | 15977277; 14761630; 8382158; 10521660; 778571; 11553351; 11517612; 9804772; 11065359; 10895688 |
| NT01NH1329 | 19 | 19 | 0.67 | 20609359; 19623961; 17640276; 18700763; 14595026; 7665491; 17346208; 17031048; 16499622; 16475802 |
| NT01NH1383 | 11 | 11 | 0.98 | 20547379; 17367389; 17078817; 16731525; 9987107; 8118209; 10556026; 8302219; 10556026; 7590258 |
| NT01NH1386 | 1 | 8 | 0.58 | 19468822; 19223513; 19123069; 17449498; 11128005; 10803945; 16889971; 16143852; 16034598; 15752726 |
| NT01NH1425 | 11 | 11 | 0.91 | 20803777; 20717103; 20618853; 20562864; 20547379; 20539943; 20512353; 20398357; 20361665; 20348299 |
| NT01NH1431 | 8 | 8 | 0.87 | 20460714; 20197505; 8082838; 18442173; 18022655; 8101441; 17722886; 17681940; 17643319; 17448440 |
| NT01NH1433 | 12 | 12 | 0.36 | 20835795; 20829063; 20821174; 20816804; 20815241; 20811797; 20809104; 20807231; 20804471; 20803007 |
| NT01NH1461 | 4 | 4 | 0.77 | 16980456; 17379726; 10712693; 6162715; 3049248 |
| NT01NH1573 | 4 | 4 | 0.88 | 20825347; 20818414; 20817757; 20817397; 20807205; 20740281; 20735775; 20711458; 20692617; 20685968 |
| NT01NH1574 | 19 | 19 | 0.55 | 20019290; 17176046; 15705580; 11237337; 11148046 |
| NT01NH1590 | 19 | 8 | 0.43 | 20639341; 20110296; 19535342; 19413184; 19212905; 19017271; 18552274; 12763040; 17994454; 17602156 |
| NT01NH1636 | 4 | 4 | 0.65 | 19482930; 18302792; 4373438; 16091049; 11029689; 8849449; 1991719; 2152901; 912749 |
| NT01NH1682 | 11 | 11 | 0.96 | 17464954; 17136348; 16143613; 16581939; 5634904; 9872971; 12777515; 11544527; 11169193; 1332797 |
| NT01NH1683 | 11 | 11 | 0.94 | 20844938; 20842711; 20815820; 20808959; 20803777; 20803249; 20737195; 20725145; 20717103; 20716342 |
| NT01NH1741 | 5 | 12 | 0.40 | 20846480; 20808847; 20687516; 20684510; 20639193; 20572126; 20523108; 20494994; 20470439; 20417190 |
| NT01NH1749 | 8 | 8 | 0.82 | 10841975; 14758542; 12421312; 9914305; 9524269; 1850420; 8057830; 7715602; 7715601; 8231805 |
| NT01NH1751 | 8 | 8 | 0.31 | 18174132; 17706591; 12421312; 10841975; 12427766; 11744735; 9693743 |
| NT01NH1752 | 8 | 8 | 0.91 | 20807714; 20799369; 20705660; 20730854; 20660112; 20628047; 20624914; 20622059; 20555380; 20522731 |
| NT01NH1754 | 15 | 18 | 0.52 | 20799851; 20615443; 20570303; 20435829; 20435076; 20396898; 20389078; 20132282; 20034458; 20082609 |
| NT01NH1763 | 1 | 13 | 0.41 | 19761831; 19438718; 18493948; 2876389; 18440867; 10708854; 17939683; 17488285; 17419734; 17355125 |
| NT01NH1765 | 18 | 18 | 0.82 | 19448268; 19422055; 18930916; 18592219; 18349501; 8058041; 12215815; 17559518; 7766211; 16790427 |
| NT01NH1782 | 19 | 19 | 0.96 | 20655076; 20632938; 20552260; 20528916; 20455156; 20444687; 20429910; 20358173; 20227065; 20153546 |
| NT01NH1802 | 2 | 2 | 0.91 | 19647806; 19270703; 18421771; 16954203; 15748981; 15581577; 12777395; 12771147; 12547821; 2174243 |
| NT01NH1918 | 12 | 4 | 0.44 | 20847516; 20845449; 20845448; 20833800; 20832475; 20824104; 20823511; 20823215; 20823199; 20814576 |
| NT01NH1927 | 6 | 17 | 0.33 | 18849072; 1970563; 8278350; 15565717; 15563835; 12628247; 12872134; 12754257; 10369684; 11866515 |
| NT01NH1947 | 11 | 11 | 0.98 | 20665904; 19326202; 10791775; 19009320; 18348984; 10376593; 16364752; 12142423; 12142423; 15469818 |
| NT01NH2017 | 8 | 8 | 0.51 | 20797444; 20731358; 20727352; 20723231; 20720220; 20679506; 20675471; 20652907; 20639328; 20624223 |
| NT01NH2023 | 18 | 18 | 1.00 | 20808924; 20805402; 20662775; 20656493; 20454456; 20439763; 20412802; 20408914; 20399185; 20203106 |
| NT01NH2101 | 12 | 12 | 0.49 | 15007058; 10369667; 8951810; 10066835; 8197122; 1825804; 9489663; 8951810; 8548804; 7650029 |
| NT01NH2128 | 19 | 8 | 0.19 | 20656821; 20639227; 20503105; 20495030; 20427274; 20382795; 19793136; 19766588; 19697071; 19684313 |
| NT01NH2155 | 19 | 19 | 0.88 | 19804974; 5700707; 19349513; 19237750; 16138814; 10428949; 11329257; 11173485; 11118459; 11084021 |
| NT01NH2159 | 12 | 12 | 0.39 | 19801578; 19290383; 18582870; 18318686; 18057781; 17854136; 17521179; 17216280; 16982891; 16923807 |
| NT01NH2162 | 12 | 12 | 0.89 | 20498258; 20169075; 19135451; 19383693; 9361426; 18930082; 18842584; 8298032; 18644791; 18036542 |
| NT01NH2170 | 18 | 18 | 0.56 | 20723231; 16332890; 19628586; 19581367; 19535599; 19427227; 19231868; 19118350; 18675470; 18574186 |
| NT01NH2216 | 6 | 12 | 0.25 | 20480359; 20020054; 19229565; 19228955; 8684467; 18775895; 18481231; 18392087; 18277117; 17640036 |
| NT01NH2231 | 18 | 18 | 0.86 | 20715920; 20616104; 20599691; 20583998; 20581201; 20575627; 20550927; 20545840; 20534468; 20525265 |
| NT01NH2259 | 6 | 6 | 0.95 | 20673215; 20626626; 20564464; 20523737; 20497332; 20457011; 20441441; 20403322; 20154704; 20102227 |
| NT01NH2271 | 18 | 18 | 0.58 | 12877469; 10226045; 2549011; 9523453; 16348575; 8991852; 8905098; 7968531; 1741458 |
| NT01NH2305 | 4 | 8 | 0.63 | 20843102; 20818253; 20801937; 20798904; 20739907; 20729713; 20723593; 20687231; 20684609; 20683028 |
| NT01NH2313 | 18 | 18 | 0.91 | 16889884; 15763514; 15168617; 6213620; 11841238; 9787771; 9264544 |
| NT01NH2331 | 11 | 11 | 1.00 | 20725145; 20603069; 20585120; 20562304; 20547379; 20543074; 20538004; 20512402; 20512353; 20502527 |
| NT01NH2333 | 11 | 11 | 0.99 | 17367389; 18227257; 17367389; 16731525; 9987107; 8118209; 12878520; 8302219; 10556026; 7590258 |
| NT01NH2405 | 6 | 6 | 0.71 | 20598726; 19556007; 18806297; 10322435; 17657734; 16405962; 11711545; 15528191; 9649516; 12777796 |
| NT01NH2465 | 9 | 9 | 0.98 | 20656373; 20338739; 20030724; 19940943; 19708881; 12906115; 6530599; 17010159; 16882035; 13546130 |
| NT01NH2490 | 6 | 6 | 0.63 | 20727027; 20698105; 20658474; 20547965; 20518660; 20505143; 20334924; 20299102; 20116104; 20048176 |
| NT01NH2498 | 19 | 19 | 0.84 | 20709752; 20658733; 20625206; 20608695; 20601594; 20351099; 20593276; 20593275; 20593271; 20593260 |
| NT01NH2607 | 12 | 12 | 0.63 | 17570395; 17374604; 16738799; 16246025; 16141198; 10966480; 11602263; 11427685; 11382754; 11106496 |
| NT01NH2613 | 4 | 4 | 0.85 | 20848490; 20848286; 20848225; 20848190; 20846484; 20846340; 20846259; 20846025; 20845086; 20843677 |
| NT01NH2652 | 13 | 12 | 0.32 | 19805289; 15901499; 18724928; 18724935; 15459192; 15208322; 14645504; 10202161; 11104765; 10610322 |
| NT01NH2660 | 8 | 8 | 0.83 | 20816962; 20610779; 20558724; 20117074; 19815558; 19496622; 19496621; 19189973; 19155212; 17640900 |
| NT01NH2661 | 8 | 8 | 0.82 | 20117074; 19496621; 16156794; 15175326; 15035646; 11728457; 10491142; 8536688; 1605643 |
| NT01NH2663 | 8 | 8 | 0.85 | 20117074; 19189973; 17640900; 15943805; 15736965; 15520003; 15175326; 15102833; 12740360; 12600982 |
| NT01NH2664 | 8 | 8 | 0.89 | 19815558; 17640900; 15943805; 15520003; 15102833; 9718301; 8797851 |
| NT01NH2668 | 8 | 8 | 0.79 | 19155212; 16973619; 15353043; 11245799; 10549856; 3214167 |
| NT01NH2696 | 4 | 8 | 0.68 | 20797613; 20713732; 20589823; 20520597; 20485749; 20445272; 20416506; 20375018; 20367470; 20337711 |
| NT01NH2719 | 2 | 2 | 0.95 | 17822383; 17217963; 15094056; 12269807; 11286891; 11200221; 10944349; 10225425; 8971718; 4917899 |
| NT01NH2758 | 4 | 4 | 0.43 | 20580965; 20571951; 20353426; 20336374; 20037191; 19953573; 19924247; 19801673; 19785996; 19725048 |
| NT01NH2801 | 2 | 2 | 0.67 | 20815377; 20498089; 19477904; 19021507; 17442677; 17372774; 15379587 |
| NT01NH2857 | 11 | 11 | 0.90 | 20609525; 15493821; 14745593; 10384296; 10432630; 7932704; 1878168; 2693210; 2965249; 2517491 |
| NT01NH2858 | 11 | 11 | 0.98 | 20843802; 20802084; 20531477; 20506290; 20351260; 20096705; 20026407; 19897657; 19895817; 19854925 |
| NT01NH2868 | 11 | 11 | 0.98 | 20848296; 20844906; 20843802; 20826161; 20817910; 20817773; 20806260; 20802084; 20739271; 20709901 |
| NT01NH2965 | 8 | 8 | 0.97 | 20823090; 20813901; 20807763; 20732359; 20727375; 20700628; 20659504; 20624914; 20622059; 20615115 |
| NT01NH3088 | 9 | 9 | 0.79 | 19551636; 18608229; 11418572; 17457695; 10388564; 12207032; 14728676; 14517516; 12837870; 12647275 |
| NT01NH3090 | 9 | 9 | 1.00 | 20838866; 20804609; 20825197; 20729355; 20625650; 20534558; 20488739; 20463027; 20460577; 20418421 |
| NT01NH3105 | 12 | 12 | 0.63 | 20829794; 20362275; 20677832; 20675494; 20659464; 20639890; 20156738; 20598751; 20596249; 20554781 |
| NT01NH3136 | 19 | 19 | 0.91 | 20331963; 19217283; 19214747; 18563288; 9254694; 18393820; 18387370; 18355438; 18343875; 3036870 |
| NT01NH3149 | 8 | 8 | 0.92 | 17126562; 15060050; 16272392; 11904922; 9582432; 2402433; 2394679; 7765840; 8181753; 7391023 |
| NT01NH3157 | 8 | 8 | 0.77 | 20628047; 20466730; 20382024; 20221790; 20050916; 20031978; 19796169; 19459937; 19090787; 19021509 |
| NT01NH3162 | 18 | 18 | 0.65 | 20041993; 19731368; 19153809; 16313612; 10417645; 15733922; 6792081; 12779236; 12409204; 11731159 |
| NT01NH3168 | 6 | 6 | 0.92 | 20511501; 20384788; 20202939; 20147403; 19805216; 19450506; 19143592; 10551881; 19028696; 18366438 |
| NT01NH3170 | 15 | 15 | 0.77 | 10217494; 17601173; 15263816; 10656595; 10217494; 9197412; 8045426 |
| NT01NH3232 | 2 | 1 | 0.63 | 19922747; 18007551; 17139593; 20507450; 16163487; 10533754; 11319580; 11223945; 8922917; 2681782 |
| NT01NH3292 | 12 | 12 | 0.96 | 20599668; 19853572; 19804735; 19684140; 19131330; 18387365; 18339324; 17980516; 16834335; 16573693 |
| NT01NH3304 | 6 | 6 | 0.98 | 20847942; 20842177; 20826341; 20811461; 20811460; 20808892; 20798040; 20739603; 20723756; 20711169 |
| NT01NH3343 | 19 | 8 | 0.33 | 15805590; 15023057; 11807280; 9367902; 9141694; 8664262; 8144449 |
| NT01NH3355 | 18 | 18 | 0.32 | 20410291; 10222271; 15980069; 7582013; 4882709 |
| NT01NH3356 | 19 | 19 | 0.57 | 20731789; 20035716; 19762447; 19459932; 19369395; 19482107; 18390543; 10585436; 17974560; 17884843 |
| NT01NH3367 | 11 | 11 | 0.95 | 20215437; 20167267; 20090173; 20005797; 19665056; 19473321; 11419933; 19184476; 18815788; 12524543 |
| NT01NH3512 | 18 | 18 | 0.92 | 20637256; 20562273; 20545848; 20482592; 20356456; 20173000; 20139406; 20078128; 20067302; 19968959 |
| NT01NH3542 | 11 | 11 | 1.00 | 20197135; 19879290; 19821988; 19416360; 15262929; 17028591; 15616333; 10381117; 15268934; 10545172 |
| NT01NH3588 | 15 | 15 | 0.88 | 20664079; 20559421; 20542497; 20508090; 20359331; 20345659; 20192838; 20083609; 20037159; 19858184 |
| NT01NH3598 | 5 | 8 | 0.28 | 20838768; 20449577; 20389065; 20348213; 20079473; 20053941; 19931920; 19916942; 19884277; 19705157 |
| NT01NH3599 | 5 | 5 | 0.91 | 20833878; 20816204; 20807646; 20709838; 20707913; 20679209; 20670992; 20668488; 20650733; 20646068 |
| NT01NH3603 | 5 | 5 | 0.31 | 20808938; 20716181; 20643830; 20370351; 20165970; 20040309; 20082166; 19423733; 17653048; 19029806 |
| NT01NH3636 | 11 | 11 | 0.97 | 19879290; 17541834; 3770949; 1622263; 7868610; 2830457; 11430408; 11322825; 10411263; 9696766 |
| NT01NH3650 | 11 | 11 | 0.97 | 10530453; 10339816; 8531886; 11315188; 1444394; 9571135; 9141667; 8382825 |
| NT01NH3654 | 11 | 11 | 0.99 | 17367389; 18227257; 17367389; 16731525; 9987107; 8118209; 12878520; 8302219; 10556026; 7590258 |
| NT01NH3698 | 11 | 11 | 0.97 | 3770949; 16133337; 10077450; 1610178; 2105293; 7698675; 8065263; 8121409; 2545526; 2416475 |
| NT01NH3702 | 11 | 11 | 0.98 | 20547379; 17367389; 17078817; 16731525; 9987107; 8118209; 10556026; 8302219; 10556026; 7590258 |
| NT01NH3735 | 15 | 15 | 0.40 | 20389118; 20068355; 19959396; 17470813; 8808945; 11972789; 15966722; 10686131; 11491351; 10564522 |
| NT01NH3754 | 11 | 11 | 0.96 | 20071371; 19195484; 16990289; 15384490; 10783303; 10481080; 10079521; 9858724; 9133596; 8224896 |
| NT01NH3772 | 8 | 8 | 0.81 | 20818520; 20383024; 16876450; 16133321; 10482501; 11495997; 8366043; 11294638; 11376009; 11294638 |
| NT01NH3786 | 2 | 2 | 0.94 | 18959753; 17686778; 17509837; 12777624; 17374639; 11290749; 15632135; 15269205; 15247236; 15146494 |
| NT01NH3807 | 4 | 4 | 0.92 | 20543056; 20487023; 20065060; 20056773; 19900770; 19808235; 19687245; 17704225; 18501535; 10103189 |
| NT01NH3825 | 12 | 12 | 0.99 | 19913481; 19241474; 17616559; 17074076; 17241197; 16923819; 16805830; 15866516; 15772085; 15544571 |
| NT01NH3837 | 19 | 19 | 0.86 | 12531907; 9575203; 1846149; 9268317; 6370994; 8366125; 1323052; 3027079 |
| NT01NH3874 | 12 | 12 | 0.89 | 20187257; 19824885; 9847218; 17428367; 14871029; 11927288; 11603724; 11279546; 11164190; 11073228 |
| NT01NH3875 | 13 | 6 | 0.91 | 20840878; 20833755; 20825972; 20811718; 20808872; 20805329; 20801195; 20798234; 20736948; 20729792 |
| NT01NH3877 | 2 | 2 | 0.27 | 20643967; 20559568; 20554717; 20406948; 20406854; 20402600; 20373247; 20359635; 20209896; 20206266 |
| NT01NH3932 | 18 | 18 | 0.90 | 20826467; 20817434; 20795855; 20728542; 20726534; 20713660; 20693822; 20688172; 20660709; 20633935 |
| NT01NH3934 | 4 | 4 | 0.60 | 20848490; 20848190; 20846484; 20846340; 20846025; 20845086; 20843549; 20839391; 20838926; 20838922 |
| NT01NH3942 | 2 | 4 | 0.36 | 20346714; 20054617; 20040020; 19797125; 12915875; 18719182; 18599097; 18548460; 18187174; 18067335 |
| NT01NH4037 | 13 | 13 | 0.92 | 19435325; 19285326; 17450376; 17145094; 15870694; 14726522; 12702319; 12425951; 12039996; 11999372 |
| NT01NH4069 | 4 | 12 | 0.33 | 20834237; 20832320; 20830735; 20823099; 20801929; 20738257; 20720108; 20718733; 20684781; 20683004 |
| NT01NH4072 | 5 | 5 | 0.91 | 20053710; 20044451; 19077168; 17983588; 17450323; 2420798; 15849794; 14757162; 10224133; 1729235 |
| NT01NH4094 | 8 | 8 | 0.98 | 20622059; 20546754; 20524628; 20513347; 20411714; 20226371; 20192696; 20176330; 20091229; 20067779 |
| NT01NH4120 | 12 | 12 | 0.98 | 20599761; 19658433; 19558329; 12528878; 18959767; 18844448; 18495113; 17878665; 17643392; 17597310 |
| NT01NH4123 | 5 | 5 | 0.98 | 20844550; 20844281; 20840600; 20838441; 20835736; 20832688; 20831369; 20817838; 20814750; 20811014 |
| NT01NH4128 | 5 | 5 | 0.86 | 20194510; 19545881; 19543980; 19254800; 10542236; 17591443; 17320124; 11576438; 15935411; 10381392 |
| NT01NH4169 | 8 | 8 | 0.89 | 15910742; 10544288; 9665692; 9548917; 8885847; 7961841; 6260575; 1309809; 1309808; 2168206 |
| NT01NH4170 | 8 | 8 | 0.83 | 15910742; 11017202; 10544288; 9737851; 9665692; 9548917; 9484234; 9443817; 9298948; 9169436 |
| NT01NH4202 | 12 | 12 | 0.61 | 20821392; 20659418; 20557125; 20124006; 19734126; 19734121; 19309077; 9630213; 19055324; 18991392 |
| NT01NH4203 | 9 | 8 | 0.60 | 20838866; 20625717; 20416081; 20195860; 20077426; 19674858; 19432594; 12073338; 18996107; 18461320 |
| NT01NH4204 | 8 | 8 | 0.97 | 20505714; 20421951; 20228121; 20202874; 20059546; 20053769; 20028790; 19768395; 19635800; 19584533 |
| NT01NH4210 | 6 | 6 | 0.33 | 19410520; 19004346; 11711552; 10706276; 12578369; 10677220 |
| NT01NH4214 | 6 | 6 | 0.79 | 20800503; 20734974; 20541511; 20140205; 19773422; 19747545; 19200727; 16187348; 18642930; 11316811 |
| NT01NH4268 | 8 | 8 | 0.73 | 20427286; 19405028; 19381641; 18817750; 17728250; 17713928; 17661446; 17166851; 16273368; 16233297 |
| NT01NH4295 | 5 | 5 | 0.93 | 18991405; 7042903; 18758731; 10781554; 14571369; 10592350; 17853355; 17519237; 17352498; 10613872 |
| NT01NH4297 | 5 | 5 | 0.43 | 19889875; 5922972; 16666044; 8384204; 2545683; 2824486 |
| NT01NH4359 | 4 | 4 | 0.92 | 20830297; 19812028; 19621581; 19231283; 19138450; 19017109; 12364606; 18435695; 18365248; 18351334 |
| NT01NH4363 | 8 | 8 | 0.83 | 11405622; 9521736; 9092498; 8550613; 2644269 |
| NT01NH4364 | 8 | 8 | 0.82 | 11803023; 10446163; 10216163; 1964456; 9521736; 7877484; 8550613; 7947903; 8120006; 8334158 |
| NT01NH4379 | 2 | 2 | 0.95 | 11461190; 7635153; 8590013; 7763270; 8001680; 7923811; 8188266; 8444860 |
| NT01NH4465 | 1 | 1 | 0.85 | 19394346; 19041910; 18765924; 18242192; 15383717; 12570844; 3700390; 11731178; 1259145; 10508663 |
| NT01NH4532 | 8 | 6 | 0.75 | 20604506; 20120238; 20059735; 20034395; 19274447; 19157743; 19076303; 19018936; 17640961; 18789974 |
| NT01NH4535 | 8 | 8 | 0.83 | 20799944; 20516587; 20399532; 20224939; 20217866; 19995736; 19926682; 19825612; 19578873; 19529819 |
| NT01NH4562 | 18 | 18 | 0.82 | 20815935; 20808924; 20720170; 20720110; 20713660; 20713507; 20704561; 20702707; 20695525; 20688079 |
| NT01NH4644 | 4 | 4 | 0.95 | 20573715; 20525833; 20516200; 19788546; 19818024; 19788546; 19854907; 19243443; 15952899; 18812085 |
| NT01NH4659 | 6 | 4 | 0.19 | 20530153; 20018881; 18281457; 4377758; 17947582; 10698952; 17174332; 16816388; 16540477; 16476729 |
| NT01NH4666 | 11 | 11 | 0.97 | 20709901; 20543074; 20521842; 20467813; 20449818; 20143346; 20136635; 20085626; 20071371; 20056825 |
| NT01NH4678 | 12 | 12 | 0.29 | 20842472; 20809670; 20801665; 20716389; 20705730; 20619605; 20610588; 20575287; 20554187; 20545225 |
| NT01NH4736 | 6 | 6 | 0.58 | 20603158; 20443037; 20354588; 20299199; 20116856; 20074037; 20061450; 20028838; 20021668; 19923722 |
| NT01NH4854 | 6 | 6 | 0.97 | 20228247; 20176571; 20145144; 20133050; 19956593; 19917599; 19802874; 19740104; 9311978; 10637609 |
| NT01NH4869 | 8 | 8 | 0.91 | 20839014; 20724480; 20690620; 20362274; 20359206; 20334431; 20213543; 20192260; 20179327; 20174697 |
| NT01NH4879 | 6 | 6 | 0.89 | 19616486; 18682218; 8284199; 11106395; 12226667; 16938414; 16901897; 16565170; 16290249; 15766919 |
| NT01NHA0019 | 6 | 8 | 0.55 | 20820855; 20705048; 20662379; 20656615; 20639477; 20638362; 20599764; 20595053; 20578081; 20559580 |
| NT01NHA0086 | 8 | 8 | 0.71 | 20421651; 19214757; 17894815; 16569761; 10065837; 11250197 |
| NT01NHA0142 | 4 | 4 | 0.66 | 20810736; 20810423; 20802223; 20657594; 20586190; 20585957; 20550930; 20509856; 20493903; 20435732 |
| NT01NHA0152 | 8 | 8 | 0.80 | 20846142; 20844569; 20843794; 20840762; 20839885; 20836540; 20832504; 20231231; 20830374; 20820853 |
| NT01NHA0168 | 6 | 6 | 0.93 | 17202163; 8947057; 17148461; 9931007; 9635738; 11724530; 12020947; 8420970; 10593256; 9862476 |
| NT01NHA0191 | 6 | 6 | 0.65 | 19943231; 19805312; 18441068; 19409284; 11943666; 19017876; 19183434; 18801011; 2808515; 8478078 |
| NT01NHB0005 | 4 | 18 | 0.32 | 20807449; 20716835; 20628263; 20616926; 20608614; 20594972; 20574139; 20529087; 20522498; 20467363 |
| NT01NHB0043 | 11 | 4 | 0.36 | 20822537; 20737195; 20734751; 20607625; 20515644; 20493845; 20489206; 20453031; 20403379; 20377208 |
| NT01NHB0045 | 4 | 4 | 0.74 | 19445953; 19400780; 17355860; 17140412; 17114934; 15993073; 15381417; 9717215; 2146683; 10998168 |
| NT01NHB0078 | 9 | 9 | 0.98 | 18307304; 15995648; 15988790; 12785312; 12217032 |
| NT01NHB0127 | 15 | 15 | 0.46 | 20671064; 20540986; 19424634; 17094466; 15792646; 14760686; 8034700; 6235151; 2540154 |
| NT01NHB0160 | 4 | 4 | 0.46 | 20728434; 20498855; 20487277; 20105222; 19747491; 19712680; 19297318; 19161859; 9770453; 18600776 |
| NT01PE0017 | 19 | 19 | 0.90 | 19754149; 10231527; 16755996; 11679076; 9353064; 10338486; 10092655; 9197543; 9099672; 8662613 |
| NT01PE0029 | 18 | 5 | 0.58 | 20839006; 20804587; 20819064; 20803547; 20800068; 20676911; 20649839; 20639259; 20635135; 20631178 |
| NT01PE0056 | 8 | 8 | 0.91 | 20823090; 20727375; 20624914; 20622059; 20615115; 20598281; 20524628; 20519277; 20498629; 20398675 |
| NT01PE0071 | 18 | 4 | 0.31 | 15049826; 17307845; 15472468; 12694907; 11506909; 10361292 |
| NT01PE0076 | 8 | 8 | 0.93 | 20388716; 20359200; 20154126; 19764800; 19697907; 18838782; 18757819; 18282009; 18085548; 17931594 |
| NT01PE0080 | 19 | 19 | 0.99 | 20036252; 19351587; 19210622; 19165143; 18667432; 18560152; 16790772; 17439951; 17283383; 17229144 |
| NT01PE0086 | 8 | 12 | 0.88 | 20484564; 20351104; 20131326; 19998026; 19822763; 19805168; 19689355; 19663685; 19635984; 19489607 |
| NT01PE0087 | 18 | 18 | 0.96 | 20634426; 20513761; 20483324; 20185828; 19926787; 19632193; 19496556; 19403800; 19332496; 19332356 |
| NT01PE0110 | 8 | 8 | 0.79 | 20836999; 20732827; 20514322; 20507258; 20486657; 20437086; 20361961; 20302929; 20084589; 20033292 |
| NT01PE0129 | 18 | 18 | 0.69 | 19372150; 7934840; 9521785; 2187815; 2656259; 1904860; 1791754; 1994034; 3317413; 2897067 |
| NT01PE0131 | 18 | 18 | 1.00 | 20659291; 20656493; 20628015; 20471400; 20419407; 20233931; 20212112; 20208152; 20204450; 20173761 |
| NT01PE0141 | 12 | 8 | 0.30 | 20720313; 19824402; 16280324; 9254694; 15755450; 6855597; 15632440; 12766342; 7830566; 12193604 |
| NT01PE0143 | 8 | 8 | 0.83 | 19159700; 17971396; 12100551; 10382966; 11054562; 9831660; 8703024 |
| NT01PE0154 | 14 | 8 | 0.39 | 20847421; 20843049; 20839901; 20836991; 20836136; 20835817; 20835457; 20833877; 20832111; 20831739 |
| NT01PE0170 | 8 | 8 | 0.80 | 20696867; 20639325; 20548048; 20304328; 20304089; 20060383; 20041601; 20018576; 19896403; 19667317 |
| NT01PE0174 | 15 | 15 | 0.92 | 20702715; 20513108; 20495996; 20463238; 20454693; 20416788; 20416650; 20410283; 20372880; 20346936 |
| NT01PE0189 | 8 | 8 | 0.49 | 20302299; 20147623; 19932076; 19892731; 19472231; 1680677; 15924433; 15225039; 15023059; 14570457 |
| NT01PE0204 | 15 | 15 | 0.37 | 20675476; 20639338; 20637912; 20600118; 20597005; 20512923; 20500749; 20472795; 20463078; 20446002 |
| NT01PE0239 | 8 | 5 | 0.97 | 20832688; 20149107; 20036411; 19783635; 19278345; 19270124; 19121075; 18721677; 18256468; 18248375 |
| NT01PE0251 | 15 | 18 | 0.24 | 17370038; 16102597; 16718597; 9622351; 16333751; 12948487; 16193283; 16102597; 15325046; 8602163 |
| NT01PE0253 | 18 | 18 | 0.75 | 20190429; 18989698; 18545925; 11144363; 17594241; 17541312; 17392613; 16228297; 15959851; 15763135 |
| NT01PE0269 | 8 | 8 | 0.82 | 20809073; 20171064; 19921179; 19897762; 19824993; 19506862; 19452479; 19202108; 19026644; 18926807 |
| NT01PE0341 | 4 | 8 | 0.27 | 20651166; 20538257; 20464042; 20403380; 20346134; 20302929; 20222012; 20216376; 20202763; 20190048 |
| NT01PE0343 | 15 | 15 | 0.72 | 20847010; 19208626; 19096365; 15353566; 18202699; 16689794; 15720559; 15455157; 12824394; 12826660 |
| NT01PE0359 | 8 | 8 | 0.78 | 20833709; 20829229; 20826822; 20815784; 20805327; 20734996; 20666976; 20619018; 20615833; 20604742 |
| NT01PE0371 | 12 | 12 | 0.99 | 20536447; 20444688; 19911840; 19665591; 19303951; 19240715; 18979629; 18930847; 18690782; 18336325 |
| NT01PE0373 | 2 | 8 | 0.80 | 20213113; 19411430; 19096097; 11976147; 18323643; 18050911; 17223081; 12029038; 16232604; 15556370 |
| NT01PE0399 | 5 | 8 | 0.31 | 10735872; 15805776; 11709175; 11092948; 5101773 |
| NT01PE0409 | 12 | 12 | 0.98 | 20668094; 20487289; 20159555; 19339102; 18995833; 18599817; 10397760; 18097612; 17280684; 17182002 |
| NT01PE0425 | 19 | 19 | 0.61 | 20687343; 20089862; 20038538; 19734307; 19594830; 19570983; 19520844; 19234698; 2833557; 19121696 |
| NT01PE0543 | 8 | 8 | 0.60 | 20618157; 20496989; 20233057; 20232573; 20093801; 20067563; 20057321; 20055838; 19863206; 19839645 |
| NT01PE0593 | 18 | 18 | 0.95 | 20586423; 20572666; 20299406; 19731368; 19159260; 18156677; 18990190; 18428096; 18402979; 14507365 |
| NT01PE0605 | 18 | 18 | 0.68 | 20739551; 20615971; 20609369; 20543081; 20540719; 20431056; 20538682; 20519555; 20519125; 20495143 |
| NT01PE0630 | 8 | 1 | 0.43 | 19617363; 17944491; 3071703; 3038546; 6759855; 776934; 4850204; 14168690 |
| NT01PE0634 | 15 | 15 | 0.97 | 19376850; 16194239; 15228517; 11500437; 12826660; 6388494; 12147687; 10802742; 8550521; 9724717 |
| NT01PE0636 | 15 | 15 | 0.76 | 19843215; 19557345; 9283083; 18281696; 11980663; 14580210; 12487371; 3736674; 10915804 |
| NT01PE0657 | 6 | 6 | 0.90 | 18007608; 10600744; 12667053; 11937058; 10778854; 10669596; 8383243 |
| NT01PE0658 | 6 | 6 | 0.77 | 10600744; 12667053; 11955076; 11810227; 11804608; 10377891; 1551594; 2184308; 1918028; 9062700 |
| NT01PE0695 | 8 | 1 | 0.56 | 18364348; 12044674; 11513584; 10769114; 9772162; 9772161; 9772160; 8987982; 7704254; 2187374 |
| NT01PE0735 | 15 | 18 | 0.36 | 20833896; 20805337; 20643656; 20601470; 20573962; 20571957; 20549193; 20543043; 20518707; 20472801 |
| NT01PE0752 | 12 | 12 | 0.49 | 15007058; 10369667; 8951810; 10066835; 8197122; 1825804; 9489663; 8951810; 8548804; 7650029 |
| NT01PE0764 | 18 | 8 | 0.20 | 20042597; 17520477; 10103220; 16096267; 11697902; 10629441; 7559412 |
| NT01PE0775 | 1 | 8 | 0.91 | 20736162; 20697932; 20695200; 20680264; 20662973; 20597986; 20576519; 20540954; 20459763; 20443025 |
| NT01PE0784 | 12 | 12 | 0.78 | 20230833; 19526727; 19159700; 18948221; 17971396; 11217865; 16547004; 16388577; 10479292; 2025413 |
| NT01PE0793 | 4 | 4 | 0.88 | 20660743; 20616062; 20651703; 20504960; 20479466; 20404563; 20381335; 20346677; 20308424; 20237820 |
| NT01PE0815 | 18 | 18 | 0.87 | 17103013; 10209755; 1744033; 2925670; 13192304 |
| NT01PE0823 | 12 | 12 | 0.65 | 20620146; 20419407; 20149234; 19716823; 19678842; 19546215; 18617521; 18621730; 18617521; 18503771 |
| NT01PE0839 | 6 | 6 | 0.83 | 20438097; 20227927; 20222748; 20184295; 20166732; 20133751; 20000331; 19955559; 19954157; 19945437 |
| NT01PE0840 | 15 | 15 | 0.96 | 20303978; 19843335; 19723245; 19632156; 19282236; 15453820; 10637320; 18776694; 18707611; 15205444 |
| NT01PE0872 | 15 | 15 | 0.98 | 20848286; 20833806; 20825354; 20817842; 20817764; 20705470; 20702407; 20693330; 20688824; 20659313 |
| NT01PE0896 | 8 | 8 | 0.83 | 19921179; 19897762; 19824993; 19588068; 19506862; 19452479; 19202108; 19026644; 18926807; 18751695 |
| NT01PE0913 | 8 | 8 | 0.84 | 20334669; 20097858; 19767835; 16261196; 18237635; 18237634; 18237633; 7513694; 18043907; 17956127 |
| NT01PE0916 | 8 | 8 | 0.91 | 18673073; 10550680; 14672950; 10544288; 10486564; 10471783; 10436930; 10393339; 9737851; 9665692 |
| NT01PE0918 | 8 | 8 | 0.58 | 9217023; 9169436; 8663126; 7703256; 8394111; 6260575; 1322173; 2168206 |
| NT01PE0923 | 2 | 8 | 0.21 | 20073225; 18824174; 18536467; 18194628; 18174123; 17924466; 17641833; 17520837; 16405840; 15949362 |
| NT01PE0956 | 18 | 18 | 0.92 | 19842939; 19735648; 19400747; 19361895; 19220397; 18037401; 18559527; 18398970; 12036927; 18300232 |
| NT01PE0976 | 12 | 12 | 0.93 | 16920629; 12475170; 11994302; 11053447; 10393315; 10393315 |
| NT01PE0986 | 8 | 8 | 0.86 | 20669254; 20602352; 20546308; 20491655; 20432928; 20215780; 20213113; 20208375; 20206593; 20148520 |
| NT01PE1047 | 18 | 18 | 0.68 | 20705137; 20089671; 19400752; 19847115; 19021534; 18989627; 18097638; 18038191; 17158757; 17397510 |
| NT01PE1116 | 18 | 18 | 0.71 | 20639340; 20534338; 20526342; 19623929; 19526856; 18763574; 17617696; 17322206; 377280; 11321576 |
| NT01PE1126 | 18 | 18 | 0.89 | 20826755; 20666228; 20666226; 20666220; 20655872; 20579775; 20560356; 20537585; 20499084; 20441547 |
| NT01PE1131 | 18 | 18 | 0.97 | 20812788; 20807204; 20692388; 20643857; 20601378; 20561140; 20509117; 20486179; 20447458; 20435771 |
| NT01PE1137 | 6 | 6 | 0.97 | 20811460; 20730596; 20708016; 20657823; 20655937; 20568999; 20628409; 20540339; 20526281; 20484375 |
| NT01PE1142 | 15 | 15 | 0.89 | 20056703; 19843219; 19818022; 19596773; 19570137; 9632254; 15063853; 19138193; 19076234; 17220217 |
| NT01PE1151 | 15 | 15 | 0.99 | 20577782; 17555437; 17184904; 11752325; 12089140; 9254694; 12097428; 12039043; 11204766; 2197994 |
| NT01PE1153 | 18 | 18 | 0.66 | 20844307; 20844306; 20844305; 20844304; 20844303; 20844302; 20842585; 20835844; 20833734; 20832413 |
| NT01PE1161 | 4 | 4 | 0.64 | 19735955; 17971082; 17908227; 15509585; 10521424; 9988687; 8344960 |
| NT01PE1182 | 4 | 19 | 0.78 | 19800869; 18667582; 10831430; 16790772; 17289203; 17080475; 9390549; 10601279; 15200051; 10482513 |
| NT01PE1237 | 18 | 18 | 0.93 | 15850393; 15169767; 12039966; 11299317; 9778127; 2007142; 1429514; 1718037; 6402489 |
| NT01PE1301 | 12 | 12 | 0.91 | 20847217; 20846409; 20845960; 20844575; 20844218; 20843828; 20842208; 20841485; 20841355; 20840862 |
| NT01PE1308 | 1 | 8 | 0.48 | 20179139; 8405359; 2123394; 13267987; 4764723; 6615127 |
| NT01PE1330 | 2 | 2 | 0.73 | 18083805; 4874308; 10672188; 15943815; 14898026; 9446573; 7929373; 8425548 |
| NT01PE1335 | 2 | 2 | 0.93 | 20627200; 4874308; 12093296; 11939774; 10758367; 14898026; 9697817; 8416664; 3528129 |
| NT01PE1336 | 19 | 19 | 0.81 | 20816775; 20803100; 20798166; 20720016; 20714719; 20703955; 20666458; 20656870; 20655076; 20639136 |
| NT01PE1392 | 12 | 12 | 0.97 | 20652663; 20440617; 20389065; 20346382; 20334618; 20057138; 20044953; 20032414; 20027867; 19914209 |
| NT01PE1394 | 4 | 4 | 0.86 | 20445251; 20398219; 17498620; 16376524; 16014621; 11296216; 12164807; 8709851; 9522454; 9393861 |
| NT01PE1424 | 4 | 4 | 0.43 | 20600507; 19833250; 19754432; 19715344; 19699239; 19630880; 19506802; 19454478; 19111750; 19109295 |
| NT01PE1439 | 1 | 8 | 0.43 | 20433928; 20420455; 20374530; 20043233; 20080659; 19864430; 19855961; 19807158; 19799945; 19603180 |
| NT01PE1483 | 1 | 13 | 0.88 | 20837012; 20717102; 20676734; 20658157; 20632938; 20630732; 20606262; 20601684; 20592025; 20571117 |
| NT01PE1493 | 12 | 12 | 0.71 | 20455857; 20028270; 19710096; 19391634; 19178150; 18950866; 18655790; 18477847; 18220146; 18051297 |
| NT01PE1522 | 4 | 8 | 0.56 | 20726808; 20699069; 20595409; 20593505; 20568248; 20551460; 20544508; 20525484; 20521035; 20518082 |
| NT01PE1553 | 17 | 17 | 0.58 | 20823555; 20665953; 20507976; 20106955; 20008574; 19945430; 19906695; 19809192; 19778900; 19733517 |
| NT01PE1604 | 12 | 8 | 0.22 | 20848643; 20848600; 20848532; 20848398; 20848304; 20848231; 20848218; 20848213; 20848210; 20848209 |
| NT01PE1614 | 18 | 18 | 0.90 | 19136005; 18625400; 16922681; 10547847; 15051137; 12175911; 7148736 |
| NT01PE1615 | 18 | 18 | 0.61 | 20797394; 20732439; 20668095; 20559454; 20457831; 20392798; 20380737; 19443054; 20197420; 20149227 |
| NT01PE1634 | 14 | 14 | 0.91 | 20823251; 19632197; 16952372; 16787778; 16462750; 16485000; 16098976; 12779339; 10567345; 10567345 |
| NT01PE1656 | 8 | 18 | 0.58 | 20223741; 19943161; 19803484; 19762344; 19734123; 19671009; 17942726; 16169296; 8069633; 18194661 |
| NT01PE1665 | 12 | 12 | 0.93 | 20814077; 20809561; 20723691; 20717115; 20708923; 20699358; 20687486; 20685334; 20677914; 20666852 |
| NT01PE1672 | 6 | 6 | 0.97 | 20603075; 20304994; 20144564; 19925793; 19897571; 12769856; 19047735; 19018518; 18972020; 18342610 |
| NT01PE1691 | 15 | 15 | 0.71 | 20211130; 20079730; 19996103; 19888420; 19820703; 19749375; 19713957; 19690171; 19585113; 19398578 |
| NT01PE1696 | 18 | 18 | 0.44 | 20803137; 20464999; 20433804; 20406297; 20153847; 20070893; 19887136; 19744586; 11097914; 19468029 |
| NT01PE1704 | 13 | 6 | 0.67 | 20837538; 20824077; 20679393; 20609457; 20555324; 20550164; 20492341; 20473859; 20418380; 20404330 |
| NT01PE1709 | 14 | 14 | 0.52 | 20826431; 20716488; 20481216; 20480551; 20415463; 20334620; 20225247; 20198873; 20088583; 20023034 |
| NT01PE1723 | 6 | 6 | 0.59 | 2160938; 15493334; 15476899; 15165237; 12393938; 11827961; 2721504; 2407737; 8901570; 3016704 |
| NT01PE1795 | 15 | 15 | 0.92 | 20847583; 20847043; 20847010; 20846954; 20845381; 20844019; 20844018; 20844002; 20844000; 20841501 |
| NT01PE1798 | 18 | 18 | 0.61 | 1374941; 5432063; 2033074; 5432063; 2647746; 2645283; 7050725 |
| NT01PE1800 | 8 | 8 | 0.74 | 20525824; 20134243; 18214971; 18060533; 17692531; 17364200; 10411325; 17225146; 15914052; 15518536 |
| NT01PE1801 | 4 | 4 | 0.84 | 697802; 16779844; 16229464; 15703173; 14675764; 11504612; 11473257 |
| NT01PE1850 | 8 | 8 | 0.24 | 20848592; 20848231; 20848180; 20847933; 20847312; 20847052; 20847010; 20847002; 20846997; 20846917 |
| NT01PE1904 | 4 | 4 | 0.76 | 18224398; 9379903; 12122010; 8733242; 1622933 |
| NT01PE1946 | 18 | 18 | 0.90 | 20585060; 20418143; 20335169; 20147287; 20113483; 20095157; 19737895; 19696110; 19504741; 19270115 |
| NT01PE1950 | 11 | 8 | 0.27 | 20804575; 20444836; 20126524; 19908400; 19447790; 19180175; 19074507; 18629303; 18629147; 18629028 |
| NT01PE1965 | 8 | 8 | 0.71 | 19170879; 10766746; 15064768; 16272392; 101140; 2394679 |
| NT01PE1969 | 8 | 18 | 0.42 | 20848033; 20847002; 20823710; 20841992; 20841959; 20841359; 20831617; 20831597; 20828130; 20827300 |
| NT01PE1979 | 8 | 12 | 0.37 | 20838899; 20838571; 20835851; 20831529; 20830524; 20827790; 20826098; 20809667; 20824296; 20823052 |
| NT01PE1995 | 2 | 2 | 0.82 | 17335870; 16206477; 12736664; 11816029; 10094680; 9245815; 8640549 |
| NT01PE1996 | 5 | 19 | 0.23 | 20181750; 20176146; 19923723; 19896456; 16760490; 19574167; 19448856; 19411252; 19379742; 19251690 |
| NT01PE2018 | 17 | 12 | 0.49 | 20491485; 20363939; 20080737; 19778906; 19557000; 19505533; 19505477; 19501596; 15780939; 19408959 |
| NT01PE2019 | 4 | 19 | 0.77 | 19800869; 18667582; 10831430; 16790772; 17289203; 17080475; 9390549; 10601279; 15200051; 10482513 |
| NT01PE2021 | 12 | 12 | 0.35 | 20070121; 19718045; 19185003; 15572368; 10611060; 7711042; 9344417; 2186809; 9043669; 8830660 |
| NT01PE2033 | 5 | 5 | 0.97 | 20635345; 20207756; 19476442; 14521881; 9746590; 8764511; 7065255; 1624427; 16404152; 16152655 |
| NT01PE2062 | 4 | 4 | 0.70 | 20698686; 20606269; 20377263; 19816718; 10708363; 18973837; 19346471; 19297322; 19275532; 19224923 |
| NT01PE2067 | 8 | 8 | 0.87 | 20803305; 20737532; 20714442; 20713188; 20684561; 20660157; 20652669; 20606288; 20580947; 20550791 |
| NT01PE2069 | 8 | 8 | 0.62 | 20516620; 19281248; 18576032; 16846243; 14700562; 12356321; 8901552; 10339827; 11667376; 8672817 |
| NT01PE2088 | 1 | 1 | 0.91 | 20720017; 20529854; 20429919; 20379751; 19955263; 19953300; 19296828; 8344414; 19143986; 18799456 |
| NT01PE2112 | 4 | 12 | 0.71 | 8951819; 1610182; 1365905; 1943702; 2181233; 3891742 |
| NT01PE2153 | 19 | 19 | 0.98 | 20201406; 19883124; 19825675; 19414810; 19298858; 9049429; 18508770; 18486536; 18445019; 18043952 |
| NT01PE2209 | 6 | 6 | 0.96 | 20818995; 20682265; 20659295; 20638158; 20550156; 20541502; 20541510; 20534341; 20228247; 20152159 |
| NT01PE2227 | 18 | 18 | 0.99 | 20684594; 20662321; 20614693; 20581201; 20548943; 20548793; 20533224; 20519121; 20510202; 20494648 |
| NT01PE2259 | 18 | 12 | 0.74 | 20421414; 16619263; 16618126; 15471851; 15140882; 15009896; 14697231; 12370023; 11756337; 11732624 |
| NT01PE2286 | 18 | 18 | 0.53 | 20832343; 20702712; 20694135; 20675551; 20650544; 20606642; 20563702; 20551915; 20542064; 20534885 |
| NT01PE2335 | 15 | 15 | 0.94 | 8971711; 8302847; 1938931; 15528658; 10943406; 8302847; 9218770; 8969172; 8804390; 8866479 |
| NT01PE2347 | 18 | 2 | 0.96 | 18846290; 18846282; 16915519; 12175014; 8602155; 9716491; 1351299; 9418040 |
| NT01PE2393 | 18 | 18 | 0.83 | 20530735; 20231161; 20332039; 19531598; 19776258; 19389857; 18823665; 18803303; 12011098; 18562324 |
| NT01PE2403 | 4 | 4 | 0.56 | 20679507; 20639362; 20594961; 20585120; 20573216; 20562876; 20421403; 20395214; 20363940; 20359483 |
| NT01PE2407 | 18 | 18 | 0.64 | 20147287; 19955416; 19775248; 19696110; 18990190; 17626019; 17600077; 17216354; 10601218; 16889643 |
| NT01PE2429 | 2 | 8 | 0.41 | 20716859; 20567911; 20059486; 19842439; 19830588; 19704083; 19674121; 19534905; 19395485; 19322676 |
| NT01PE2477 | 15 | 18 | 0.29 | 20727012; 20460858; 19846594; 19683048; 19619602; 19390785; 19269601; 18299909; 18052344; 17855180 |
| NT01PE2482 | 15 | 4 | 0.82 | 20723231; 18299451; 17975079; 17268768; 4568763; 16112521; 15636745; 12480902; 11980902; 1409657 |
| NT01PE2495 | 8 | 8 | 0.90 | 20195856; 19996699; 4338489; 17975081; 17720458; 17684705; 17213679; 16930152; 16802151; 16765316 |
| NT01PE2498 | 8 | 8 | 0.47 | 20727010; 20695438; 20663063; 20662380; 20363611; 20195856; 20192827; 20007967; 19959255; 19796135 |
| NT01PE2513 | 4 | 8 | 0.35 | 20209484; 19653299; 19527171; 19136719; 19074874; 18707125; 9597156; 17309689; 17270225; 17023065 |
| NT01PE2574 | 18 | 18 | 0.95 | 20583998; 20534468; 20051474; 20035006; 19805313; 16541604; 19713238; 19574656; 19432486; 17905989 |
| NT01PE2595 | 15 | 15 | 0.98 | 20833814; 20811812; 20722734; 20639324; 20571030; 20543072; 20542040; 20521955; 20487268; 20453099 |
| NT01PE2611 | 15 | 15 | 0.90 | 20831657; 20824160; 20821075; 20816895; 20814407; 20810008; 20800633; 20799635; 20738250; 20728507 |
| NT01PE2678 | 4 | 4 | 0.88 | 20738376; 20690824; 20690670; 20680094; 20625514; 20601469; 20543066; 20543140; 20453864; 20471399 |
| NT01PE2698 | 2 | 8 | 0.25 | 20730937; 20701370; 20627514; 20558329; 20557689; 20446670; 20392612; 20183124; 20148597; 20084435 |
| NT01PE2709 | 18 | 8 | 0.66 | 20844539; 20688826; 20622453; 20498375; 20211979; 20067187; 20041954; 20012540; 20051980; 20006708 |
| NT01PE2722 | 6 | 6 | 1.00 | 20689513; 20687508; 20687504; 20600125; 20564624; 20553504; 20495087; 20491904; 20444702; 20431719 |
| NT01PE2744 | 8 | 8 | 0.95 | 20816850; 20697122; 20691393; 20691179; 20689707; 20660112; 20646165; 20625939; 20618079; 20599983 |
| NT01PE2753 | 11 | 11 | 0.94 | 20844938; 20842711; 20815820; 20808959; 20803777; 20803249; 20737195; 20725145; 20717103; 20716342 |
| NT01PE2761 | 12 | 12 | 0.91 | 14731284; 15494746; 16796689; 15550391; 14731284; 10481021; 10089316 |
| NT01PE2772 | 12 | 12 | 0.89 | 16195792; 16513561; 16232749; 16142900; 10727942; 15913357; 12943232; 12031843; 11473256; 11069674 |
| NT01PE2877 | 4 | 18 | 0.35 | 20639340; 19926649; 19903372; 19411325; 18931428; 17967850; 17850346; 9603894; 15336432; 15225313 |
| NT01PE2940 | 14 | 14 | 0.92 | 20836050; 20823283; 20800313; 20735209; 20716342; 20704576; 20652015; 20623247; 20617462; 20592083 |
| NT01PE2994 | 8 | 8 | 0.98 | 10471295; 18177365; 17619188; 17549795; 5432063; 374376; 9297469; 6723659; 9109378; 9043126 |
| NT01PE3004 | 13 | 13 | 0.32 | 20824822; 20716949; 20709901; 20689233; 20684516; 20669242; 20644968; 20631690; 20605701; 20587501 |
| NT01PE3026 | 8 | 8 | 0.90 | 19580167; 19465655; 19235509; 18331335; 18283541; 17483937; 17223783; 17205041; 10433976; 697766 |
| NT01PE3030 | 8 | 8 | 0.71 | 6546423; 8795196; 10438748; 11390387; 9634695; 8795196; 2604403; 8486285 |
| NT01PE3100 | 19 | 19 | 0.98 | 20847002; 20843347; 20726582; 20713676; 20608745; 20593835; 20580675; 20547785; 20542210; 20525686 |
| NT01PE3108 | 18 | 18 | 0.98 | 20810662; 20585060; 20463304; 20445416; 20424477; 20418143; 20398325; 20392994; 20382771; 20335169 |
| NT01PE3131 | 18 | 18 | 0.92 | 20828170; 20826447; 20709735; 20705135; 20693281; 20656779; 20628049; 20610401; 20573661; 20548793 |
| NT01PE3164 | 18 | 18 | 0.53 | 8744570; 14514697; 10480876; 11827477; 11470432; 11456221; 11087367; 10893239; 10518715; 10480876 |
| NT01PE3187 | 15 | 15 | 0.99 | 20724137; 20586430; 20442958; 20178784; 20154125; 20123795; 20110293; 19995076; 19926656; 19822742 |
| NT01PE3194 | 15 | 15 | 0.90 | 20676725; 20133655; 19369699; 12824373; 18288918; 16194235; 16600681; 18451049; 10579532; 9890992 |
| NT01PE3198 | 8 | 8 | 0.86 | 20460724; 20154126; 18757819; 17657404; 15107237; 14977570; 12829696; 9613842; 9558336; 140652 |
| NT01PE3223 | 9 | 9 | 0.92 | 20727852; 20707002; 20534481; 20370610; 20334431; 19897762; 19821147; 19684143; 19639238; 19625492 |
| NT01PE3245 | 6 | 17 | 0.88 | 20847378; 20844870; 20844575; 20844014; 20833242; 20832020; 20826783; 20824087; 20823885; 20822495 |
| NT01PE3249 | 15 | 15 | 0.85 | 20594961; 20552019; 20524642; 20137101; 20059681; 19846594; 19760662; 19602148; 19539673; 19400783 |
| NT01PE3264 | 9 | 9 | 0.99 | 20797606; 20563648; 20406823; 20081823; 20038703; 20013982; 19954230; 19797355; 19779936; 19733180 |
| NT01PE3287 | 18 | 18 | 0.84 | 18485365; 18061677; 12935905; 12598694; 12383255; 1649946; 10064713; 2185220; 9367758; 8878033 |
| NT01PE3319 | 18 | 18 | 0.99 | 20585060; 20335169; 19737895; 19609963; 19432807; 18390651; 10498707; 17578453; 16973745; 9268330 |
| NT01PE3374 | 8 | 8 | 0.96 | 20025615; 15943805; 10987141; 8797851; 2256929 |
| NT01PE3427 | 9 | 8 | 0.53 | 20825197; 20814823; 20685719; 20670938; 20659813; 20650852; 20648024; 20583174; 20580542; 20532823 |
| NT01PE3437 | 6 | 6 | 0.91 | 19635595; 19563116; 19119875; 18048913; 9923595; 5327367; 17929923; 17881053; 8300534; 17343677 |
| NT01PE3476 | 15 | 15 | 0.94 | 20659289; 20562308; 20511503; 20137101; 19818019; 19539673; 19465657; 19376919; 19138192; 15937167 |
| NT01PE3518 | 12 | 12 | 0.95 | 20822545; 20820890; 20818888; 20818894; 20816329; 20812371; 20808763; 20806405; 20801431; 20740581 |
| NT01PE3622 | 14 | 8 | 0.21 | 19438719; 19246762; 6288664; 17989916; 17242516; 16603157; 15496593; 12860706; 8995266; 8397198 |
| NT01PE3632 | 8 | 8 | 0.70 | 18983169; 17019698; 11843181; 11350062; 9220005 |
| NT01PE3639 | 12 | 12 | 0.96 | 20847230; 20847055; 20846409; 20843828; 20839288; 20834233; 20833800; 20832724; 20830808; 20829355 |
| NT01PE3664 | 4 | 4 | 0.82 | 20132451; 19732341; 19648242; 19383688; 19376867; 19332819; 17768253; 17542929; 12057936; 17067800 |
| NT01PE3668 | 17 | 17 | 0.72 | 20800068; 20471983; 16826544; 16754669; 10227159; 10322161; 11731318; 10632888; 11029451; 15130135 |
| NT01PE3671 | 4 | 4 | 0.86 | 19889085; 19665005; 12864857; 16677309; 15170399; 10320579; 10572114; 9286988; 2982790 |
| NT01PE3680 | 4 | 4 | 0.89 | 20639318; 1915293; 3905770; 1640458; 10320579; 8996114; 7770032; 8407873; 2404955; 2193164 |
| NT01PE3724 | 15 | 12 | 0.41 | 19374930; 15063853; 10984043; 15561138; 14641574; 12407452; 10476040; 9218775; 7108955; 8899712 |
| NT01PE3757 | 13 | 13 | 0.54 | 19731322; 19664587; 17900615; 14566049; 16712869; 16107339; 15772074 |
| NT01PE3801 | 11 | 11 | 0.99 | 20562304; 20554775; 20553579; 20543074; 20197135; 20192694; 20085626; 20058811; 20010837; 20007650 |
| NT01PE3827 | 1 | 13 | 0.68 | 20651954; 20636328; 20355127; 20162729; 20023723; 19943898; 19878959; 19851727; 19706604; 19656298 |
| NT01PE3873 | 12 | 12 | 0.92 | 19916930; 19819899; 19665591; 18512343; 10829079; 18215358; 17530480; 18029266; 17952400; 17897481 |
| NT01PE3876 | 6 | 6 | 0.42 | 20140205; 16187348; 18642930; 11316811; 10880511; 17516660; 1584761; 17368670; 17055493; 17029241 |
| NT01PE3896 | 18 | 18 | 0.40 | 20700484; 20620867; 20614727; 20606116; 20562855; 20559552; 20544018; 20543078; 20538893; 20471983 |
| NT01PE3897 | 19 | 19 | 0.28 | 20140365; 19917711; 19753488; 19492806; 19428664; 17971860; 18044876; 12476489; 17588214; 9473028 |
| NT01PE3902 | 15 | 15 | 0.98 | 20154125; 20123795; 19632156; 19286656; 19282236; 19238575; 17275217; 17599915; 18497483; 18174141 |
| NT01PE3925 | 19 | 19 | 0.91 | 20210661; 11320139; 18174142; 18173801; 17426021; 15318951; 15032825; 14997492; 11748726; 10564478 |
| NT01PE3926 | 12 | 18 | 0.61 | 20525826; 19524550; 19199920; 18633280; 18533835; 16267305; 18565907; 12783268; 2007544 |
| NT01PE3948 | 4 | 12 | 0.49 | 20844927; 20833806; 20807494; 20806243; 20798597; 20734145; 20718006; 20713134; 20713057; 20655297 |
| NT01PE3965 | 19 | 19 | 0.33 | 20675476; 19727946; 18192383; 19021569; 18947195; 16818608; 17609140; 17561945; 17345077; 17185552 |
| NT01PE3966 | 8 | 8 | 0.96 | 20737579; 20695524; 20690702; 20632184; 20600130; 20547565; 20534338; 20471952; 20453296; 20410318 |
| NT01PE3982 | 11 | 18 | 0.23 | 20848296; 20844906; 20802084; 20709901; 20709082; 20699282; 20693318; 20684967; 20665904; 20660769 |
| NT01PE4004 | 11 | 18 | 0.45 | 20848296; 20845959; 20844906; 20844038; 20844016; 20843830; 20843802; 20841424; 20839450; 20838623 |
| NT01PE4019 | 11 | 11 | 0.97 | 20660769; 19426744; 1731246; 17056065; 16476446; 12354230; 15469818; 14569303; 12083526; 11922669 |
| NT01PE4022 | 11 | 11 | 0.95 | 20426877; 19324050; 18807113; 18051757; 17703358; 17426023; 16960352; 16932843; 12142423; 16457869 |
| NT01PE4032 | 8 | 8 | 0.55 | 20547883; 20340016; 19328793; 18646549; 18310048; 11297442; 17478552; 15803413; 12472681; 12466263 |
| NT01PE4043 | 4 | 4 | 0.55 | 20363951; 19635793; 17693520; 18832310; 10217758; 18312270; 10675323; 17185541; 2180916; 9882666 |
| NT01PE4068 | 1 | 18 | 0.33 | 20669961; 20516379; 20434517; 20111909; 20064684; 20019812; 19812031; 19784870; 19673519; 19624407 |
| NT01PE4070 | 12 | 12 | 0.93 | 17828281; 18824173; 16622848; 11246026; 15255192 |
| NT01PE4072 | 1 | 1 | 0.85 | 19394346; 19041910; 18765924; 18242192; 15383717; 12570844; 3700390; 11731178; 1259145; 10508663 |
| NT01PE4108 | 18 | 8 | 0.20 | 17264988; 15123668; 8758981; 12428729; 11479714; 16350087; 2515891; 2544457 |
| NT01PE4128 | 12 | 8 | 0.28 | 20848677; 20848674; 20848673; 20848668; 20848659; 20848653; 20848643; 20848642; 20848615; 20848614 |
| NT01PE4131 | 2 | 2 | 0.94 | 17822383; 17217963; 15094056; 12269807; 11286891; 11200221; 10944349; 10225425; 8971718; 4917899 |
| NT01PE4139 | 15 | 17 | 0.60 | 18524926; 16699585; 10792721; 9387225; 7108955; 16558051; 8188579 |
| NT01PE4185 | 18 | 18 | 0.93 | 19902387; 19894176; 19569551; 19151209; 18830567; 18815723; 18700191; 18498772; 18316319; 18088102 |
| NT01PE4209 | 18 | 18 | 0.95 | 20847098; 20836765; 20833807; 20807196; 20803137; 20801781; 20739286; 20733047; 20715920; 20667831 |
| NT01PE4225 | 15 | 15 | 0.97 | 20594941; 20133363; 18611278; 12563033; 10637320; 18039772; 18604637; 18093135; 18022383; 11728723 |
| NT01PE4238 | 14 | 14 | 0.72 | 20831412; 20421948; 20082641; 20045992; 19995917; 19900465; 19666713; 19556875; 19460093; 19368784 |
| NT01PE4275 | 8 | 1 | 0.16 | 20808309; 20806090; 20740228; 20706768; 20705665; 20660674; 20651853; 20626585; 20584675; 20569467 |
| NT01PE4292 | 12 | 12 | 0.93 | 20824277; 20632945; 20592285; 20479960; 20413723; 20359484; 20230906; 20200278; 20173027; 20170058 |
| NT01PE4293 | 6 | 6 | 0.91 | 20337945; 20329707; 20222445; 20188667; 19749191; 7630406; 18065386; 17210572; 6363409; 16767502 |
| NT01PE4297 | 6 | 6 | 0.54 | 20552019; 20457559; 20404275; 20376789; 20371694; 20350864; 20179009; 20173765; 20167605; 20157333 |
| NT01PE4304 | 15 | 8 | 0.34 | 19778357; 14974729; 10672184; 11694004; 10322176; 9324261; 10735247; 9586587; 9575470; 3031008 |
| NT01PE4314 | 6 | 6 | 0.93 | 20846371; 20841484; 20838444; 20825418; 20818161; 20804913; 20804568; 20802089; 20798175; 20720536 |
| NT01PE4322 | 6 | 6 | 0.71 | 20463878; 20019078; 18812193; 17893750; 1495996; 16824196; 16585648; 16328543; 15474408; 15109717 |
| NT01PE4331 | 19 | 19 | 0.77 | 20383009; 19052362; 9054558; 12566566; 15256026; 14659047; 9303405; 10605111; 9371343; 10066483 |
| NT01PE4350 | 8 | 8 | 0.97 | 20448037; 20448035; 20075289; 19855063; 9886074; 18930705; 18793174; 18713733; 18701458; 8387337 |
| NT01PE4351 | 8 | 8 | 0.98 | 20651150; 20544923; 20496909; 20492669; 20130997; 20091229; 20075289; 20025846; 20023020; 19892700 |
| NT01PE4366 | 5 | 5 | 0.92 | 19770499; 15065880; 15065853; 12426581; 12361716; 11389593; 8990263; 8051058; 8274013; 1730615 |
| NT01PE4370 | 18 | 18 | 0.98 | 20382768; 20376793; 19775248; 17717154; 19556290; 19544044; 19453273; 19065762; 19061401; 18394148 |
| NT01PE4372 | 18 | 18 | 0.96 | 19556290; 9244288; 8529885; 12468528; 11442842; 10564520; 10373434; 9268677; 8951382; 8411172 |
| NT01PE4373 | 9 | 8 | 0.51 | 20839914; 20836064; 20827550; 20823294; 20810539; 20801890; 20716241; 20678669; 20669015; 20653675 |
| NT01PE4376 | 2 | 2 | 0.84 | 20054111; 19946146; 16046629; 11891227; 11135669; 8514783 |
| NT01PE4387 | 19 | 12 | 0.24 | 20159460; 20097812; 19407377; 10854426; 12581364; 8936313; 3894021; 8057826; 1743507 |
| NT01PE4448 | 15 | 15 | 0.88 | 20523354; 20383199; 19965638; 19481544; 19306868; 19158184; 19062282; 18626943; 18523156; 11683498 |
| NT01PE4454 | 8 | 6 | 0.90 | 17316685; 16963438; 16882299; 16298387; 15588821; 12940821; 2830029; 10692383; 10913144; 10844646 |
| NT01PE4491 | 11 | 11 | 0.96 | 20847047; 20829283; 20803062; 20716205; 20709901; 20708437; 20705506; 20631792; 20630557; 20619867 |
| NT01PE4535 | 9 | 9 | 0.99 | 8663192; 16904369; 336620; 16762557; 6404883; 15381119; 15028711; 11421270; 14739287; 14663079 |
| NT01PE4549 | 18 | 18 | 0.97 | 20639324; 20300601; 20049509; 18215300; 18771089; 10760164; 17411076; 15918073; 10203757; 15361615 |
| NT01PE4565 | 15 | 15 | 0.96 | 20844149; 20816754; 20808885; 20801044; 20739554; 20702721; 20610765; 20593779; 20590098; 20513357 |
| NT01PE4589 | 18 | 18 | 0.91 | 20586423; 20572666; 20525826; 19731368; 19159260; 18156677; 18402979; 18205408; 18164722; 18162469 |
| NT01PE4596 | 8 | 8 | 0.94 | 20419722; 20417637; 20121093; 20070127; 20026409; 19746907; 19692330; 19666553; 19635596; 19583219 |
| NT01PE4608 | 8 | 8 | 0.84 | 15448722; 18273560; 17394573; 17205041; 12709400; 10467151; 8107120; 6323474; 5963505; 6273403 |
| NT01PE4609 | 8 | 8 | 0.94 | 17205041; 10467151; 10467151; 2061286; 8107120; 6323474; 6273403; 6794459 |
| NT01PE4623 | 14 | 5 | 0.62 | 20091765; 18338572; 16585939; 18083805; 17020555; 15296732; 9972; 10507012; 8043576; 8473316 |
| NT01PE4694 | 4 | 4 | 0.77 | 20823511; 20805337; 20507608; 20433738; 20171024; 20151316; 19955281; 19942661; 19915022; 19765590 |
| NT01PE4727 | 5 | 8 | 0.46 | 19888576; 19707758; 6389524; 19202279; 18710396; 18328504; 17604986; 17349707; 17292397; 16233198 |
| NT01PE4765 | 6 | 6 | 0.98 | 20845481; 20844976; 20833188; 20817752; 20805886; 20729302; 20725617; 20713735; 20711416; 20709753 |
| NT01PE4769 | 8 | 8 | 0.84 | 20347849; 20001966; 20113483; 20097860; 20085751; 19811920; 19744927; 19656484; 19639134; 19520720 |
| NT01PE4773 | 17 | 17 | 0.21 | 18713320; 19429620; 19215773; 18625025; 18023590; 10200269; 1758883; 17029243; 7638209; 10364165 |
| NT01PE4777 | 4 | 4 | 0.77 | 20203055; 9791175; 10052136; 9168127; 1195397 |
| NT01PE4800 | 11 | 18 | 0.95 | 19770280; 16885437; 19373193; 19208883; 18006522; 17682720; 17464069; 17417881; 17005200; 16750162 |
| NT01PE4815 | 6 | 6 | 0.27 | 20737003; 20552444; 20481745; 20409301; 20363222; 20359246; 20333467; 20329599; 20299817; 20231470 |
| NT01PE4848 | 18 | 18 | 0.24 | 20337714; 19767895; 16616885; 1498688; 15107534; 12220668; 10806256; 11686935; 11479290; 7502075 |
| NT01PE4869 | 15 | 3 | 0.98 | 20507990; 20454556; 20377643; 20074296; 20018629; 20008072; 19865481; 19592586; 19486161; 19082140 |
| NT01PE4884 | 8 | 8 | 0.23 | 18307304; 15995648; 15988790; 12785312; 12217032; 12099829; 11888314; 11815858; 11267773; 9401045 |
| NT01PE4885 | 9 | 8 | 0.41 | 20736083; 20728590; 20591565; 20565131; 20441146; 20391757; 20204456; 20031399; 19933347; 19914586 |
| NT01PE4904 | 19 | 19 | 0.90 | 20305657; 20234135; 19825597; 4269305; 19224571; 18402606; 17768255; 17640872; 17593303; 17186209 |
| NT01PE4905 | 19 | 19 | 0.31 | 20188541; 20170183; 20044250; 19711199; 19707729; 19604687; 19441226; 19399494; 19288067; 19012294 |
| NT01PE4907 | 12 | 12 | 0.92 | 20675455; 20334618; 20229202; 20188787; 20179346; 20179356; 20154109; 20078555; 20028436; 19556294 |
| NT01PE4933 | 2 | 2 | 0.94 | 20693992; 19307763; 10036175; 12527210; 11124029; 10494632; 11904168; 11320134; 11255013; 4891257 |
| NT01PE4937 | 15 | 15 | 0.68 | 18587410; 16205910; 11523004; 11259434; 11034280; 9851041; 2669674; 8564363 |
| NT01PE4960 | 8 | 8 | 0.86 | 20655923; 20160956; 20160912; 20106967; 19996100; 19801660; 19240034; 19081061; 3194009; 18675788 |
| NT01PE4966 | 15 | 15 | 0.91 | 18031348; 15522865; 8899716; 8282725; 8497200; 8432742; 2693738 |
| NT01PE4967 | 15 | 15 | 0.91 | 20719961; 20685939; 20660350; 20650961; 20624818; 20610653; 20574810; 20484458; 20472145; 20452968 |
| NT01PE4971 | 5 | 8 | 0.96 | 20516492; 20513475; 20431245; 20387665; 20371487; 20335826; 20094655; 19963048; 19806079; 19738628 |
| NT01PE4975 | 8 | 8 | 0.36 | 20675294; 16820168; 15946648; 15914915; 11330998; 7543100 |
| NT01PE4979 | 8 | 8 | 0.83 | 20514546; 17977854; 12406762; 12269813; 10952004; 10716626; 10390816; 9692922; 9642221; 13566053 |
| NT01PE5040 | 5 | 8 | 0.84 | 20481524; 20132279; 20118364; 20083495; 19938869; 19890332; 19616484; 19553202; 19194001; 19193999 |
| NT01PE5068 | 9 | 12 | 0.57 | 20823545; 20799957; 20797316; 20705608; 20703240; 20692235; 20685872; 20683962; 20662276; 20659429 |
| NT01PE5099 | 18 | 18 | 0.78 | 19805313; 19574656; 12948774; 18485072; 7759102; 11158736; 17682720; 16922505; 16840432; 15596311 |
| NT01PE5134 | 18 | 18 | 0.95 | 20843810; 20826817; 20799350; 20683952; 20665261; 20664073; 20659465; 20651349; 20651344; 20644544 |
| NT01PE5140 | 2 | 2 | 0.95 | 20545743; 7009558; 17033719; 8477729; 12799002; 3085688; 9140059 |
| NT01PE5143 | 5 | 5 | 0.98 | 20736171; 20673205; 20497125; 20067623; 19957302; 19580157; 18700747; 19323822; 19304823; 45487 |
| NT01PE5158 | 4 | 2 | 0.45 | 20848627; 20828130; 20801036; 20736169; 20722874; 20718860; 20696392; 20695229; 20693677; 20667730 |
| NT01PE5177 | 19 | 19 | 0.98 | 20829291; 20684602; 20636376; 20595206; 20530569; 20513638; 20470363; 20446311; 20427037; 20421293 |
| NT01PE5185 | 18 | 18 | 0.60 | 7860583; 8564363; 2907323; 8384683; 17770799; 2820842; 3857224; 6384184 |
| NT01PE5217 | 6 | 6 | 0.92 | 20129926; 14997524; 11839499; 3905388; 1309894; 1833878; 2542617; 9425627; 8226920; 2542611 |
| NT01PE5220 | 8 | 8 | 0.86 | 20460724; 20154126; 18757819; 17657404; 15107237; 14977570; 12829696; 9613842; 9558336; 140652 |
| NT01PE5240 | 19 | 19 | 0.81 | 20589904; 20512975; 18690721; 9761470; 16595672; 16091590; 15865428; 1829964; 15236574; 14725765 |
| NT01PE5256 | 18 | 18 | 0.96 | 20802073; 20799747; 20420522; 20227482; 19919671; 19627500; 19199920; 19119856; 18629473; 10485884 |
| NT01PE5330 | 8 | 8 | 0.22 | 17850261; 15668026; 1325563; 11410352; 7592431; 12795378; 9211896; 11410352; 10066485; 11021918 |
| NT01PE5333 | 9 | 19 | 0.63 | 20799083; 20686828; 20527376; 20037618; 19915102; 18450701; 17177517; 16935761; 15999223; 15902469 |
| NT01PE5339 | 9 | 9 | 0.99 | 20818586; 20800575; 20797606; 20727773; 20704262; 20701592; 20696503; 20693992; 20671186; 20662933 |
| NT01PE5360 | 17 | 13 | 0.46 | 20843005; 20701360; 20691896; 20661640; 20632321; 20626900; 20482805; 20480639; 20473316; 20434461 |
| NT01RU0009 | 9 | 9 | 0.73 | 20473151; 20373368; 20237999; 20188036; 20058052; 12633501; 19181555; 19029121; 10319815; 18178130 |
| NT01RU0029 | 8 | 8 | 0.83 | 18455502; 18441991; 16972982; 15497442; 12898218; 12681508; 11768297; 11295183; 10750905; 10838053 |
| NT01RU0031 | 8 | 8 | 0.80 | 19520672; 19428656; 19423706; 7637805; 17891922; 17296494; 16545948; 15892698; 12974468; 12938040 |
| NT01RU0051 | 11 | 11 | 0.95 | 19018586; 16322504; 16581939; 5634904; 15987878; 12100480; 12777515; 12612829; 11169193; 1332797 |
| NT01RU0113 | 12 | 4 | 0.35 | 10348856; 11309495; 10066835; 11069681; 10732681; 9184221; 9351821; 8087850 |
| NT01RU0139 | 6 | 6 | 0.55 | 9931007; 10388557; 14659743; 11724530; 8420970; 2159140 |
| NT01RU0146 | 6 | 12 | 0.35 | 20840432; 20720508; 20554729; 20484419; 20226194; 20130858; 20091457; 20035700; 19883288; 19545125 |
| NT01RU0155 | 13 | 13 | 0.47 | 20657015; 20450227; 20377997; 20234090; 20039704; 19933278; 19651701; 18523997; 19526041; 19446023 |
| NT01RU0196 | 9 | 9 | 0.97 | 15539300; 10960477; 8388033; 3013315; 16661658; 6824716; 7236695 |
| NT01RU0223 | 6 | 6 | 0.47 | 20846395; 20844040; 20827171; 20813962; 20708208; 20698996; 20673941; 20647674; 20637070; 20632805 |
| NT01RU0290 | 15 | 12 | 0.92 | 20841356; 20826764; 20824729; 20810657; 20807766; 20801214; 20798321; 20797611; 20732402; 20576936 |
| NT01RU0295 | 4 | 12 | 0.49 | 20844927; 20833806; 20807494; 20806243; 20798597; 20734145; 20718006; 20713134; 20713057; 20655297 |
| NT01RU0306 | 4 | 4 | 0.94 | 20825347; 20818414; 20817757; 20807205; 20735775; 20711458; 20692617; 20670397; 20629754; 20625433 |
| NT01RU0336 | 12 | 12 | 0.61 | 15007058; 10369667; 8951810; 10066835; 10446151; 8197122; 10207046; 1825804; 9668058; 8951810 |
| NT01RU0337 | 14 | 15 | 0.86 | 19661060; 19447175; 18835829; 18000314; 17416458; 16436472; 16023096; 11008000; 15106839; 12697703 |
| NT01RU0389 | 8 | 19 | 0.32 | 19700326; 19428749; 18830277; 18685277; 10526172; 9268149; 10194235; 98769; 8407802; 16713314 |
| NT01RU0460 | 13 | 13 | 0.56 | 19761774; 1987132; 8636994; 8635731; 9150870; 7539688; 7737996; 8034646; 6419024; 8176726 |
| NT01RU0531 | 1 | 6 | 0.33 | 19801658; 17053788; 19143615; 17557331; 12676087; 16982615; 10506558; 15211526 |
| NT01RU0571 | 8 | 8 | 0.84 | 20524628; 20335176; 19995914; 17672825; 18445471; 17430883; 17300187; 16631971; 14963816; 15840584 |
| NT01RU0592 | 12 | 12 | 0.88 | 16737825; 14962137; 10524213; 11179678; 8022281; 7917435; 1427098; 1389315 |
| NT01RU0648 | 13 | 12 | 0.37 | 20846368; 20845092; 20844588; 20838651; 20831632; 20830743; 20828170; 20823118; 20820770; 20818334 |
| NT01RU0671 | 12 | 12 | 0.71 | 20000742; 19661058; 12198129; 15513925; 2202727; 7651187; 8344936; 2032623 |
| NT01RU0688 | 8 | 8 | 0.90 | 20528775; 20465309; 20465256; 20447061; 20419266; 20377119; 20372025; 20363268; 20331426; 20233924 |
| NT01RU0690 | 18 | 8 | 0.31 | 20688168; 20665263; 20665259; 20585503; 20546304; 20543078; 20528917; 20457929; 20362544; 20357135 |
| NT01RU0725 | 14 | 8 | 0.38 | 19879316; 18656547; 18608754; 18586121; 16821778; 7638216; 16325515; 16161998; 12208522; 12071646 |
| NT01RU0736 | 8 | 8 | 0.76 | 20844980; 20816850; 20799369; 20734249; 20732359; 20727984; 20708435; 20701985; 20691393; 20689513 |
| NT01RU0770 | 15 | 12 | 0.25 | 20709215; 20441961; 20121715; 20109267; 20070029; 19795348; 19786296; 19758192; 19712670; 19644361 |
| NT01RU0812 | 17 | 15 | 0.61 | 20116460; 19942662; 17854828; 17456470; 7975855; 15133119; 9190808; 10658767; 10692152; 10542286 |
| NT01RU0836 | 18 | 18 | 0.98 | 20843810; 20837481; 20827262; 20826817; 20813428; 20812950; 20812904; 20812902; 20805402; 20799350 |
| NT01RU0852 | 6 | 8 | 0.44 | 18723693; 10359786; 16116479; 15554870; 12950024; 12924618; 9354614; 8810272; 8239658; 1284592 |
| NT01RU0875 | 11 | 6 | 0.50 | 18931414; 11729195; 15280020; 15158734; 15066183; 10347220; 11164500; 11053259; 10903905; 9886290 |
| NT01RU0881 | 13 | 13 | 0.99 | 20132446; 15100988; 12068810; 9419243; 8980544; 8725011; 7739034; 8207023; 1875917; 2116407 |
| NT01RU0889 | 6 | 6 | 0.47 | 20846395; 20844040; 20827171; 20813962; 20708208; 20698996; 20673941; 20647674; 20637070; 20632805 |
| NT01RU0901 | 4 | 4 | 0.88 | 20566763; 20478990; 20226333; 20124555; 20046086; 19899089; 19822894; 19805143; 19666539; 19560903 |
| NT01RU0932 | 4 | 4 | 0.25 | 20843712; 20825972; 20798328; 20668008; 20652008; 20651362; 20515662; 20511288; 20480360; 20472716 |
| NT01RU0945 | 15 | 15 | 0.93 | 16430694; 11101667; 9457886; 9098050; 8969513; 7746146; 8497200; 2693738 |
| NT01RU0946 | 15 | 15 | 0.97 | 20825354; 20622066; 20581213; 20480360; 20459101; 20453099; 20447287; 20404199; 20404817; 20378989 |
| NT01RU0982 | 2 | 2 | 0.72 | 11983074; 11952125; 10551871; 9885568; 9582371 |
| NT01RU1031 | 4 | 15 | 0.43 | 19351138; 17159215; 16400685; 10889254; 12824378; 11538077; 11759840; 4998365; 10998049; 2157156 |
| NT01RU1100 | 17 | 17 | 0.99 | 20512976; 19963062; 19583999; 19199915; 2886365; 18289874; 9685179; 17822967; 17233676; 15109491 |
| NT01RU1119 | 8 | 8 | 0.75 | 20466730; 18174132; 17419738; 16920107; 16629658; 9068642; 16042600; 15465823; 10647174; 11744735 |
| NT01RU1156 | 12 | 19 | 0.71 | 16323116; 12823621; 7854121; 11881895; 9303405; 11123924; 10564477; 7791425; 10405298; 9712687 |
| NT01RU1170 | 8 | 8 | 0.66 | 20841316; 20840756; 20839296; 20838643; 20834011; 20822403; 20814880; 20808324; 20802227; 20802042 |
| NT01RU1171 | 8 | 8 | 0.97 | 20614874; 20498375; 20130997; 20091229; 20075289; 19577535; 19285991; 19254042; 19234303; 9872799 |
| NT01RU1200 | 4 | 4 | 0.93 | 20460179; 20363951; 20345660; 20066037; 19880599; 19880597; 19737354; 19635793; 19542290; 19376877 |
| NT01RU1269 | 18 | 18 | 0.90 | 20634426; 20185828; 19088429; 16137722; 15571397; 11284199; 14715671; 12578372; 12384697; 11756489 |
| NT01RU1283 | 6 | 15 | 0.41 | 20223822; 19784811; 19269039; 19223935; 19186820; 19125815; 19067324; 18926294; 18591820; 18187416 |
| NT01RU1342 | 8 | 8 | 0.82 | 11803023; 10446163; 10216163; 1964456; 9521736; 7877484; 8550613; 7947903; 8120006; 8334158 |
| NT01RU1361 | 8 | 8 | 0.79 | 19155212; 16973619; 15353043; 11245799; 10549856; 3214167 |
| NT01RU1369 | 4 | 4 | 0.69 | 20816689; 20814890; 20713626; 20644623; 20632211; 20625514; 20618854; 20570502; 20568600; 20511497 |
| NT01RU1419 | 15 | 15 | 0.81 | 20847010; 20829344; 20798056; 20718459; 20632993; 20622062; 20587330; 20581474; 20581202; 20574521 |
| NT01RU1422 | 19 | 4 | 0.54 | 18566135; 14982627; 11163206; 12809607; 12596863; 7565110; 6322174; 6162838 |
| NT01RU1425 | 19 | 12 | 0.48 | 16475801; 11876708; 11211867; 6363127; 7031065; 7009608 |
| NT01RU1478 | 13 | 6 | 0.50 | 18931414; 11729195; 15280020; 15158734; 15066183; 10347220; 11164500; 11053259; 10903905; 9886290 |
| NT01RU1488 | 18 | 18 | 0.32 | 20842634; 20660139; 20642824; 20608168; 20592031; 20581173; 20573199; 20558901; 20544014; 20519654 |
| NT01RU1524 | 18 | 18 | 0.94 | 20548793; 20103563; 19950388; 19653298; 3782037; 9751058; 10473554; 1498688 |
| NT01RU1577 | 18 | 18 | 0.94 | 20848662; 20848648; 20848555; 20848440; 20848398; 20848389; 20848387; 20848343; 20848165; 20847723 |
| NT01RU1589 | 6 | 6 | 0.39 | 15699185; 8233776; 7607526; 1657645; 2585490 |
| NT01RUA0009 | 6 | 6 | 0.96 | 20599730; 20302878; 20054126; 11600708; 17439637; 9016653; 11163967; 9931007; 8887569; 16126220 |
| NT01RUA0044 | 11 | 6 | 0.52 | 20832426; 20829066; 20829065; 20827532; 20826783; 20824628; 20824208; 20823543; 20821187; 20818995 |
| NT01RUA0056 | 14 | 8 | 0.28 | 20848677; 20848674; 20848673; 20848668; 20848659; 20848653; 20848643; 20848642; 20848615; 20848614 |
| NT01RUA0061 | 11 | 11 | 0.94 | 20844938; 20842711; 20815820; 20808959; 20803777; 20803249; 20737195; 20725145; 20717103; 20716342 |
| NT02ER0003 | 1 | 1 | 0.22 | 19954230; 19576563; 17185548; 12515465; 10037775; 7565611; 9085573; 9003320; 8621661; 7022140 |
| NT02ER0004 | 6 | 4 | 0.37 | 20842058; 20675375; 20610564; 20559326; 20490777; 20484116; 20380588; 20223211; 20212065; 20201043 |
| NT02ER0021 | 11 | 11 | 0.96 | 19010363; 18983851; 16824089; 10417259; 10572124; 14643655; 12466275; 10993723; 10801350; 10660048 |
| NT02ER0040 | 6 | 6 | 0.94 | 19618961; 3243435; 6273399; 11515498; 15661009; 11418610; 7855597; 10986118; 10856643; 4522719 |
| NT02ER0063 | 9 | 9 | 0.28 | 17726007; 16243729; 10592242; 7569993; 8748025; 7579178 |
| NT02ER0093 | 4 | 12 | 0.32 | 19556291; 15780005; 12414154; 923667; 15612921; 16272372; 15618402; 9119487; 16469062; 9923682 |
| NT02ER0108 | 8 | 8 | 0.47 | 9524269; 8617725; 8057830; 7715602; 7715601 |
| NT02ER0133 | 6 | 6 | 0.54 | 20552019; 20457559; 20404275; 20376789; 20371694; 20350864; 20179009; 20173765; 20167605; 20157333 |
| NT02ER0182 | 13 | 13 | 0.99 | 20736336; 20722735; 20719918; 20709079; 20639535; 20639334; 20604745; 20600110; 20588254; 20571291 |
| NT02ER0186 | 8 | 18 | 0.60 | 19803484; 19762344; 19734123; 18092813; 17905810; 16996694; 15365836; 15135062; 12671089; 11681732 |
| NT02ER0214 | 12 | 12 | 0.92 | 20805355; 20410297; 20156579; 20112455; 19786597; 19730897; 19596876; 19505802; 19249287; 19236878 |
| NT02ER0222 | 8 | 8 | 0.85 | 20632369; 20600565; 20529087; 20505258; 20300940; 20222442; 19859792; 19768395; 19718054; 19664747 |
| NT02ER0254 | 6 | 6 | 0.85 | 20603082; 20140215; 20129926; 19953589; 19854909; 16464007; 19628660; 19567531; 18157157; 19192394 |
| NT02ER0255 | 4 | 4 | 0.93 | 20573715; 20516200; 20298190; 20033058; 19818024; 19788546; 19376877; 18363794; 18391964; 2170028 |
| NT02ER0304 | 18 | 18 | 0.98 | 20656779; 20466975; 20519121; 20508203; 20505021; 20502630; 20492723; 20631346; 20460728; 20460510 |
| NT02ER0345 | 2 | 2 | 0.95 | 17822383; 17217963; 15094056; 12269807; 11286891; 11200221; 10944349; 10225425; 8971718; 4917899 |
| NT02ER0359 | 8 | 8 | 0.98 | 20643099; 20625049; 20460714; 20197505; 20019878; 20019223; 19849722; 19801684; 660161; 17914900 |
| NT02ER0374 | 13 | 13 | 0.99 | 20823541; 20729861; 20670890; 20070887; 19833922; 18201202; 10545121; 16842744; 17083375; 16928980 |
| NT02ER0377 | 15 | 15 | 0.92 | 20522491; 19919539; 19901023; 19695263; 13838951; 19220743; 19210621; 20076707; 16754873; 17379708 |
| NT02ER0407 | 4 | 4 | 0.64 | 19735955; 17971082; 17908227; 15509585; 10521424; 9988687; 8344960 |
| NT02ER0429 | 13 | 12 | 0.41 | 19604517; 19066471; 18062988; 15077152; 17617471; 17573556; 16895933; 11874577; 15184368; 15177505 |
| NT02ER0461 | 13 | 8 | 0.23 | 15620217; 10989132; 10656825; 9392081; 8484789 |
| NT02ER0462 | 17 | 17 | 0.68 | 20304993; 19588252; 19443547; 19329646; 19202087; 17967949; 19076234; 19025566; 15694339; 14975451 |
| NT02ER0467 | 4 | 4 | 0.33 | 20719048; 17900376; 16104019; 15081878; 10543963; 10640454; 8591001; 8211147; 9331923; 6607932 |
| NT02ER0487 | 8 | 8 | 0.78 | 19888992; 18957412; 18713742; 18084081; 16824008; 15964837; 15211518; 15102840; 11193410; 9582371 |
| NT02ER0498 | 8 | 8 | 0.92 | 20700699; 20659552; 20643099; 20638183; 20637292; 20625049; 20620150; 20618950; 20613764; 20601013 |
| NT02ER0542 | 12 | 4 | 0.17 | 9601038; 8226770; 1429620; 2197275; 2186030; 3049606 |
| NT02ER0554 | 8 | 8 | 0.98 | 20505714; 19815558; 19181534; 18799460; 18760846; 18560889; 18501578; 18243814; 17722886; 16898010 |
| NT02ER0582 | 8 | 8 | 0.97 | 20448037; 20448035; 20075289; 19855063; 9886074; 18930705; 18793174; 18713733; 18701458; 8387337 |
| NT02ER0621 | 12 | 12 | 0.98 | 20625347; 20580091; 20431301; 20334618; 20179346; 20179356; 20150869; 19620246; 19574214; 19563735 |
| NT02ER0725 | 14 | 14 | 0.93 | 20676924; 16819826; 15826652; 10903946; 2543974; 10597272; 10433703; 8811890; 7766613; 1979549 |
| NT02ER0769 | 14 | 15 | 0.35 | 20831906; 20814065; 20813141; 20805102; 20800661; 20798549; 20732952; 20724124; 20716294; 20709865 |
| NT02ER0815 | 6 | 6 | 0.92 | 19616486; 19542005; 18682218; 8284199; 11106395; 8408062; 12226667; 12202775; 16938414; 16901897 |
| NT02ER0852 | 13 | 13 | 0.91 | 20463973; 20410297; 20154190; 19590924; 18292779; 10572144; 10198119; 16411162; 16025162; 2488477 |
| NT02ER0853 | 2 | 5 | 0.58 | 17393457; 16894907; 11006082; 16301812; 16242716; 16233726; 16233126; 15582400; 15978040; 15629142 |
| NT02ER0881 | 1 | 1 | 0.91 | 20848228; 20838028; 20837041; 20830520; 20804597; 20813095; 20807241; 20806654; 20804784; 20735175 |
| NT02ER0897 | 6 | 6 | 0.78 | 20738147; 20706627; 20663713; 20647759; 20644481; 20638361; 20601675; 20527806; 20522331; 20519779 |
| NT02ER0965 | 2 | 2 | 0.83 | 20716937; 20684227; 20684226; 20648783; 20600129; 20519180; 20427470; 20471400; 20448146; 20439498 |
| NT02ER0970 | 8 | 8 | 0.84 | 20597986; 20547565; 20435888; 20376324; 20176611; 20127344; 20077697; 20035036; 8662757; 18825971 |
| NT02NS0048 | 9 | 9 | 0.99 | 20736083; 20541551; 20463021; 18281324; 18036338; 16682457; 17537807; 17395278; 16601871; 16574148 |
| NT02NS0050 | 9 | 9 | 0.94 | 20494124; 20481475; 20463021; 20334431; 20055482; 19639238; 19470521; 19673949; 19061855; 325420 |
| NT02NS0068 | 8 | 8 | 0.38 | 15547280; 17459874; 15210349; 12909015; 12147680; 5420325 |
| NT02NS0070 | 11 | 11 | 0.98 | 20547379; 17367389; 17078817; 16731525; 9987107; 8118209; 10556026; 8302219; 10556026; 7590258 |
| NT02NS0071 | 11 | 11 | 0.94 | 20844938; 20842711; 20815820; 20808959; 20803777; 20803249; 20737195; 20725145; 20717103; 20716342 |
| NT02NS0098 | 8 | 8 | 0.88 | 20143043; 20133651; 19954230; 19850488; 19635803; 19446917; 19376100; 19192390; 19140736; 19002717 |
| NT02NS0132 | 8 | 8 | 0.69 | 20427718; 20116195; 20047814; 20045729; 19953301; 19940120; 19938610; 19933331; 19882279; 19840846 |
| NT02NS0168 | 6 | 8 | 0.26 | 20848662; 20848659; 20848643; 20848628; 20848586; 20848535; 20848441; 20848437; 20848308; 20848296 |
| NT02NS0204 | 8 | 8 | 0.82 | 20838891; 20836890; 20826956; 20801894; 20801713; 20797423; 20727971; 20725133; 20701695; 20693408 |
| NT02NS0289 | 9 | 9 | 0.77 | 20691590; 20532823; 20522731; 20480395; 20463021; 20449660; 20207594; 19896671; 19889959; 19513611 |
| NT02NS0318 | 18 | 18 | 0.90 | 20719963; 16687400; 3114241; 10380629; 1955875; 6989828; 6985610 |
| NT02NS0350 | 18 | 18 | 0.63 | 20103563; 19407337; 18784280; 18781380; 17641954; 15561767; 15777766; 12214846; 12612931; 11879800 |
| NT02NS0402 | 17 | 17 | 0.93 | 20453093; 19847776; 11418118; 15561138; 15507429; 10542167; 14659743; 10713446; 12801925; 10908318 |
| NT02NS0404 | 15 | 15 | 0.91 | 17237162; 10941799; 1588910; 8878033; 8459762; 1971620 |
| NT02NS0494 | 2 | 14 | 0.95 | 20206212; 20047307; 20047306; 19399913; 6268428; 19165623; 19138656; 18321072; 18157947; 17868694 |
| NT02NS0555 | 8 | 8 | 0.54 | 19815558; 19189973; 17894548; 10652103; 15996109; 15922336; 11842179; 15736965; 15175326; 15102833 |
| NT02NS0560 | 8 | 8 | 0.94 | 19155212; 10319462; 16973619; 16850995; 16844076; 15353043; 12948635; 12615348; 10633105; 11370674 |
| NT02NS0564 | 8 | 8 | 0.91 | 20019878; 19815558; 19308673; 18809504; 17640900; 17662684; 12644575; 16981725; 15943805; 9892013 |
| NT02NS0568 | 8 | 8 | 0.32 | 19815558; 19189973; 10652103; 15996109; 15922336; 15175326; 15102833; 15035646; 12693950; 12600982 |
| NT02NS0754 | 12 | 12 | 0.97 | 20827447; 20668094; 20499929; 20487289; 20448033; 20439464; 20335166; 20159555; 20111006; 19935715 |
| NT02NS0758 | 2 | 2 | 0.56 | 20823244; 20822501; 18846281; 10807176; 17506685; 17288560; 16787044; 16523349; 16287171; 9492312 |
| NT02NS0769 | 8 | 8 | 0.92 | 20359206; 20334431; 20213543; 19843169; 19737939; 19624733; 10816041; 19438211; 19424679; 19271777 |
| NT02NS0831 | 18 | 18 | 0.80 | 20685822; 20630939; 20616037; 20513760; 19664056; 19424690; 19252899; 19160392; 18950196; 18192351 |
| NT02NS0832 | 11 | 6 | 0.80 | 20554522; 19583771; 18305051; 17146664; 19028685; 9008363; 10694879; 17610954; 17381110; 16186386 |
| NT02NS0871 | 8 | 8 | 0.89 | 19875455; 17932031; 17442675; 16535686; 10684634; 10464214; 16359327; 16233251; 16233225; 15523913 |
| NT02NS0878 | 13 | 13 | 0.47 | 20846325; 20806080; 20802180; 20799976; 20798751; 20796214; 20739275; 20737600; 20724386; 20723225 |
| NT02NS0894 | 4 | 4 | 0.85 | 19850038; 19766587; 19647889; 19228319; 19028474; 18429940; 17975734; 17968511; 17955192; 17924116 |
| NT02NS0914 | 2 | 4 | 0.43 | 18534824; 12444973; 12036276; 11024263; 10713425; 8661944 |
| NT02NS0927 | 12 | 12 | 0.95 | 20462489; 20416323; 20167799; 20068042; 20038591; 20022957; 20014030; 19843523; 19726681; 19541655 |
| NT02NS0972 | 19 | 19 | 0.77 | 14595026; 16475802; 10874732; 15366937; 15358542; 15133104; 11790124; 11139297; 10843862; 10545329 |
| NT02NS1080 | 11 | 11 | 0.99 | 20562304; 20554775; 20553579; 20543074; 20197135; 20192694; 20085626; 20058811; 20010837; 20007650 |
| NT02NS1156 | 18 | 18 | 0.32 | 20847343; 20846144; 20844015; 20843792; 20838423; 20837659; 20837023; 20833871; 20833782; 20833099 |
| NT02NS1158 | 6 | 6 | 0.95 | 20302878; 17439637; 9931007; 16125907; 15713456; 14659743; 14600243; 12879741; 11724530; 11555298 |
| NT02NS1159 | 6 | 6 | 0.92 | 20302878; 3006102; 15987807; 11491304; 10388557; 16024301; 14744977; 12172806; 10880511; 10844674 |
| NT02NS1171 | 17 | 17 | 0.97 | 1849609; 7602586; 2498283; 1195397; 3918995 |
| NT02NS1216 | 9 | 9 | 0.97 | 20598663; 20574645; 20546309; 20391335; 19944735; 19883129; 19712754; 19629472; 19621578; 19593631 |
| NT02NS1264 | 13 | 8 | 0.34 | 20210499; 19666577; 19500305; 19400779; 19369177; 19820357; 19306130; 19286778; 19170676; 19112571 |
| NT02NS1293 | 6 | 4 | 0.19 | 20530153; 20018881; 18281457; 4377758; 17947582; 10698952; 17174332; 16816388; 16540477; 16476729 |
| NT02NS1306 | 9 | 9 | 0.73 | 18680460; 17134979; 15814455; 15188046; 9388188; 12196011; 11173475; 8399222; 1526981; 3191103 |
| NT02NS1324 | 19 | 19 | 0.30 | 19966417; 19801480; 19584557; 19482267; 19432472; 18565544; 18522649; 10547298; 18287177; 17517906 |
| NT02NS1345 | 19 | 18 | 0.63 | 20703487; 20427291; 20158191; 20093094; 20046570; 19965803; 19476019; 19364515; 19307178; 18400936 |
| NT02NS1397 | 19 | 4 | 0.24 | 20188542; 18194337; 2708131; 17289203; 12535081; 1398088; 2760018 |
| NT02NS1413 | 8 | 2 | 0.28 | 20236317; 19778964; 19295650; 17442677; 16540088; 16286471; 11075338; 15247236; 10826693; 9632249 |
| NT02NS1440 | 4 | 9 | 0.94 | 20460577; 20403694; 20398792; 20371307; 20338488; 20156697; 20137333; 20133765; 20046049; 20043999 |
| NT02NS1474 | 19 | 19 | 0.99 | 20335363; 20201406; 20032302; 19883124; 19857612; 19825675; 20110776; 19414810; 19298858; 19258392 |
| NT02NS1554 | 4 | 4 | 0.54 | 19651502; 15709779; 15090538; 11104814; 9651400; 1100846; 2681140 |
| NT02NS1591 | 6 | 6 | 0.96 | 20811460; 20628409; 20526281; 20484375; 20472641; 20451470; 20421421; 20299287; 20298192; 20225163 |
| NT02NS1660 | 6 | 6 | 0.27 | 20404922; 19875418; 19788334; 19527679; 19416864; 19206208; 19084593; 18250630; 17880426; 17286576 |
| NT02NS1704 | 8 | 8 | 0.83 | 19780833; 19585534; 19425498; 19420686; 18993058; 10331874; 18051362; 15184168; 17968539; 17704565 |
| NT02NS1778 | 12 | 12 | 0.87 | 20005877; 17554808; 17483319; 16451085; 16410343; 16260779; 14599350; 11943775; 1379319; 11735566 |
| NT02NS1799 | 17 | 17 | 0.97 | 19956571; 19390092; 7960138; 17920859; 17089148; 10588743; 16497325; 16226766; 2448286; 15978076 |
| NT02NS1811 | 4 | 4 | 0.90 | 20813407; 20730631; 20679743; 20617728; 20575514; 20561586; 20554636; 20553029; 20507097; 20507077 |
| NT02NS1854 | 11 | 11 | 0.99 | 20562304; 20554775; 20553579; 20543074; 20197135; 20192694; 20085626; 20058811; 20010837; 20007650 |
| NT02NS1856 | 15 | 15 | 0.70 | 20848231; 20825659; 20823675; 20818169; 20817929; 20816397; 20811644; 20810619; 20808903; 20807764 |
| NT02NS1869 | 18 | 8 | 0.82 | 20828614; 20678098; 20667145; 20648222; 20603179; 20600464; 20572278; 20567862; 20564563; 20564561 |
| NT02NS1895 | 8 | 8 | 0.89 | 20844927; 20699282; 20696004; 20695532; 20680488; 20677338; 20659890; 20647035; 20628047; 20626653 |
| NT02NS1903 | 19 | 19 | 0.94 | 20385610; 20045390; 19708823; 19217615; 19202084; 5578901; 18787712; 15543535; 11013345; 18331355 |
| NT02NS1910 | 1 | 1 | 0.39 | 19850488; 14705032; 18054243; 9384377; 17571216; 16958849; 12773641; 14705032; 12693104; 10762266 |
| NT02NS1922 | 9 | 9 | 1.00 | 20463027; 20110695; 19903863; 19699128; 19465092; 19271216; 18357511; 17728299; 17668200; 17189250 |
| NT02NS1926 | 5 | 4 | 0.55 | 20534468; 20459313; 20456044; 20199110; 20122221; 20192823; 20153447; 20135225; 20081033; 20081032 |
| NT02NS1955 | 8 | 8 | 0.82 | 12087099; 3519582; 9490067; 9030266; 8917463; 7499307; 7663384; 8241179; 8100227; 3426633 |
| NT02NS1973 | 18 | 18 | 0.87 | 20600957; 20563497; 20467754; 20467183; 20441230; 20429884; 20405176; 20401926; 20398313; 20394798 |
| NT02NS2141 | 12 | 12 | 0.91 | 19359320; 17650073; 10066835; 16963780; 16568094; 9298646; 2670909; 10383472; 14676204; 14684302 |
| NT02NS2160 | 6 | 18 | 1.00 | 20805396; 20713739; 20684239; 20624916; 20548793; 20534468; 20460382; 20375061; 20363758; 20362640 |
| NT02NS2184 | 19 | 19 | 0.58 | 20847006; 20823121; 20821204; 20821071; 20801033; 20735059; 20722697; 20708432; 20625851; 20604537 |
| NT02NS2235 | 9 | 8 | 0.60 | 19406896; 18834333; 18544072; 18414813; 18156794; 17612506; 17419769; 8344300; 15056912; 12172805 |
| NT02NS2267 | 8 | 8 | 0.67 | 20507062; 15125696; 12085987; 11684364; 3534890; 9524244; 9498553; 9073075; 2783417 |
| NT02NS2393 | 6 | 6 | 0.91 | 2671653; 11506820; 10375529; 2676972; 6386179 |
| NT02NS2415 | 12 | 12 | 0.89 | 19734307; 17908209; 16342964; 15740115; 15642479; 15362861; 12860390; 10966480; 8206978; 1400316 |
| NT02NS2430 | 6 | 12 | 0.18 | 20458164; 20375016; 20201569; 19915612; 19852835; 19645440; 19633085; 19540834; 15295705; 19485316 |
| NT02NS2462 | 2 | 18 | 0.25 | 20842649; 20841559; 20840345; 20838622; 20830523; 20830434; 20823897; 20818966; 20816073; 20815176 |
| NT02NS2530 | 1 | 8 | 0.71 | 20734766; 20377227; 19917104; 19565632; 19463851; 19381803; 19380176; 19376919; 19215302; 15963080 |
| NT02NS2575 | 8 | 8 | 0.90 | 20693678; 19758330; 19459980; 19346355; 18987250; 18052213; 3531172; 9254694; 18093969; 18063573 |
| NT02NS2632 | 18 | 12 | 0.49 | 20814884; 20735480; 20735375; 20675712; 20657603; 20546913; 20538720; 20400545; 20385770; 20375989 |
| NT02NS2685 | 15 | 18 | 0.20 | 18470695; 17875647; 17587231; 16790020; 16473881; 16135241; 12426307 |
| NT02NS2715 | 15 | 15 | 0.97 | 20594941; 18611278; 18039772; 18604637; 18093135; 18022383; 11728723; 10940038; 17483938; 17322187 |
| NT02NS2717 | 9 | 9 | 0.37 | 20478340; 20024669; 19915674; 19400804; 9990338; 15526036; 15577262; 15516711; 12398189; 12216837 |
| NT02NS2744 | 8 | 19 | 0.77 | 20708437; 20643653; 19493008; 18471063; 14986705; 16902754; 16530188; 12765833; 12657642; 11916960 |
| NT02NS2750 | 12 | 12 | 0.89 | 19744480; 19734688; 19074976; 19074975; 18550540; 18543330; 17916556; 17873349; 17448647; 17362992 |
| NT02NS2767 | 6 | 6 | 0.92 | 15538360; 11952905; 9389475; 11590160; 7664066; 8843438 |
| NT02NS2769 | 13 | 13 | 0.91 | 20847234; 20844523; 20844240; 20844078; 20842102; 20841486; 20839808; 20837552; 20837360; 20837025 |
| NT02NS2789 | 8 | 8 | 0.72 | 18555208; 18372402; 18305374; 17654627; 8820018; 16600650; 16494875; 15716098; 15454729; 12925586 |
| NT02NS2790 | 6 | 6 | 0.74 | 20814424; 20810619; 20739539; 20691152; 20700143; 20584987; 20571955; 20551906; 20457749; 20347402 |
| NT02NS3028 | 2 | 18 | 0.39 | 20830523; 20811585; 20171405; 20632110; 20558535; 20558542; 20558164; 20533221; 20533218; 20532744 |
| NT02NS3037 | 13 | 13 | 0.98 | 20067766; 17172768; 18069966; 11698387; 11779873 |
| NT02NS3044 | 14 | 8 | 0.38 | 19879316; 18656547; 18608754; 18586121; 16821778; 7638216; 16325515; 16161998; 12208522; 12071646 |
| NT02NS3084 | 8 | 8 | 0.84 | 15632120; 10591526; 15012298; 8647852; 8027021; 2765055; 3210228 |
| NT02NS3090 | 6 | 6 | 0.88 | 20844218; 20833633; 20817842; 20675723; 20586870; 20383017; 20361665; 20360860; 20356737; 20348315 |
| NT02NS3110 | 1 | 8 | 0.30 | 16510449; 10970743; 15211520; 12963379; 11527962; 11131019; 189692 |
| NT02NS3117 | 6 | 6 | 0.49 | 20724226; 20733069; 20639336; 20621980; 20523737; 20472798; 20448024; 20421601; 20377178; 20158916 |
| NT02NS3120 | 12 | 12 | 0.98 | 20846511; 20846373; 20845933; 20844809; 20843990; 20843040; 20843039; 20842104; 20840478; 20840338 |
| NT02NS3130 | 13 | 13 | 0.99 | 19969548; 19945860; 19903445; 19446023; 18205605; 17150214; 16890206; 14750525; 12883871; 2011495 |
| NT02NS3195 | 12 | 12 | 0.93 | 20002189; 18315846; 18045386; 16703114; 17005379; 12975369; 12584195; 11544189; 10520736; 10497172 |
| NT02NS3235 | 6 | 6 | 0.60 | 20421413; 19540249; 9207108; 19056520; 4000169; 10984059; 16604074; 16978929; 16898421; 16857169 |
| NT02NS3255 | 18 | 4 | 0.40 | 20435896; 20375064; 19966021; 19724867; 19494117; 18045414; 18477612; 18478230; 12269836; 18297791 |
| NT02NS3279 | 8 | 8 | 0.98 | 20381373; 20194738; 20042690; 19855063; 19663452; 19422848; 19268423; 18482593; 10827456; 18242646 |
| NT02NS3288 | 15 | 8 | 0.30 | 20089430; 20028345; 19861313; 19766732; 19738938; 19505464; 19501620; 18787640; 19341812; 19196697 |
| NT02NS3296 | 12 | 12 | 0.94 | 18342411; 17239953; 16573693; 16076848; 15170231; 12081643; 11860552; 2546916 |
| NT02NS3452 | 6 | 8 | 0.29 | 15319033; 3802398; 10480342; 9269990; 6287922; 271968; 3948325 |
| NT02NS3477 | 4 | 4 | 0.27 | 20595000; 19801658; 19775250; 19445953; 19400780; 17053788; 19143615; 14646342; 17557331; 17355860 |
| NT02NS3503 | 2 | 2 | 0.80 | 20738254; 16897469; 16099603; 11124029; 2110099 |
| NT02NS3504 | 18 | 18 | 0.99 | 20813428; 20704181; 20639324; 20566858; 20454684; 20412049; 20408914; 20382765; 20377823; 20363791 |
| NT02NS3517 | 1 | 1 | 0.46 | 19649769; 17333302; 15299374; 16218965; 16201833; 16122935; 386920; 12714052; 12559906; 12102508 |
| NT02NS3525 | 2 | 2 | 0.74 | 20580577; 20536026; 20300940; 20108425; 19934113; 19888908; 19861948; 19825655; 19815305; 19805308 |
| NT02NS3539 | 19 | 8 | 0.38 | 20829342; 20826810; 20816225; 20815936; 20799957; 20798201; 20708082; 20680578; 20680572; 20668520 |
| NT02NS3553 | 8 | 8 | 0.68 | 20690660; 20236387; 20145308; 19699281; 19170761; 19143568; 17504214; 17334708; 9504803; 16855450 |
| NT02NS3581 | 14 | 19 | 0.46 | 20713411; 20598292; 18586271; 3762695; 16872587; 16781473; 16243288; 16233673; 16233080; 16233079 |
| NT02NS3654 | 15 | 15 | 0.90 | 20144684; 10629763; 18649864; 8402178; 1851251; 2562826; 1828858; 722560; 1669444; 2270287 |
| NT02NS3690 | 8 | 8 | 0.74 | 20838375; 20824134; 20823107; 20806441; 20802042; 20735165; 20731752; 20697024; 20664968; 20643956 |
| NT02NS3696 | 14 | 14 | 0.12 | 9817849; 16469506; 10089427; 9817849; 1540167 |
| NT02NS3708 | 8 | 8 | 0.92 | 20201001; 19466416; 17322334; 19233206; 16269415; 19118636; 18944299; 10406803; 18310297; 18028869 |
| NT02NS3723 | 8 | 8 | 0.30 | 12573487; 8939905; 7588230; 7536666; 1937041 |
| NT02NS3724 | 15 | 15 | 0.78 | 20190084; 19389781; 19307761; 9643542; 18485072; 18219120; 16936034; 10659714; 12368433; 6423769 |
| NT02NS3744 | 2 | 8 | 0.73 | 20663876; 20620150; 20617791; 20590527; 20363939; 20058910; 20023024; 19944069; 19996167; 19897476 |
| NT02NS3775 | 9 | 8 | 0.30 | 18620026; 15589119; 11356167; 11108740; 10958805; 10827011; 9804184; 9714723; 9487154; 9003458 |
| NT02NS3782 | 8 | 8 | 0.76 | 20739284; 20693675; 19566721; 10357270; 15899413; 15158259 |
| NT02NS3795 | 8 | 8 | 0.82 | 20696867; 20695849; 20639325; 20576519; 20570675; 20548048; 20470805; 20445195; 20363598; 20304328 |
| NT02NS3834 | 4 | 4 | 0.82 | 20680263; 20600954; 20386942; 20356038; 19652348; 19361444; 19207566; 19228194; 19199051; 19138154 |
| NT02NS3924 | 8 | 8 | 0.88 | 19616102; 19589965; 19307254; 19191964; 18765916; 18390572; 17188300; 17146529; 16817900; 16733043 |
| NT02NS3937 | 8 | 8 | 0.85 | 18316329; 11412090; 12603319; 12007814; 9455733; 9454581; 8647112; 7828917; 8274006; 1851684 |
| NT02NS3951 | 8 | 8 | 0.89 | 20833709; 20829229; 20826822; 20815784; 20805327; 20619018; 20615833; 20604742; 20559571; 20547138 |
| NT02NS3979 | 6 | 17 | 0.63 | 10514496; 2413440; 1619662; 2443712; 3862875 |
| NT02NS4091 | 19 | 8 | 0.47 | 20848145; 20848132; 20846433; 20844939; 20841375; 20838231; 20837182; 20836854; 20832563; 20829890 |
| NT02NS4106 | 4 | 6 | 0.39 | 20713134; 20623179; 20599698; 20596676; 20132728; 18467468; 19258348; 19238258; 18978732; 18656956 |
| NT02NS4109 | 12 | 12 | 0.68 | 19888572; 20173418; 19383694; 19292867; 19152799; 18784072; 18713730; 12196507; 15498570; 10531064 |
| NT02NS4117 | 1 | 1 | 0.97 | 19428471; 19383689; 19370061; 18565285; 11842149; 18285355; 18208521; 18184691; 18050920; 17981822 |
| NT02NS4121 | 6 | 8 | 0.26 | 20848662; 20848659; 20848643; 20848628; 20848586; 20848535; 20848441; 20848437; 20848308; 20848296 |
| NT02NS4210 | 8 | 8 | 0.36 | 20629749; 17981479; 11466286; 9633598; 3537305; 8185833 |
| NT02NS4234 | 12 | 12 | 0.99 | 19915005; 19795569; 18083885; 19152630; 18673454; 16208033; 16926147; 8839427; 12904566; 12869197 |
| NT02NS4460 | 2 | 1 | 0.66 | 20844968; 20834145; 20830599; 20827446; 20824837; 20811276; 20806654; 20796157; 20795405; 20737539 |
| NT02NS4475 | 8 | 8 | 0.55 | 19639238; 17176108; 16466958; 15274622; 15262270; 10720438; 9711871; 3081514; 2340266 |
| NT02NS4498 | 8 | 8 | 0.24 | 16554717; 11386345; 10919324; 10797242; 10699858; 9878450; 9312120; 9278282; 9188201; 8984906 |
| NT02NS4508 | 15 | 15 | 0.92 | 18554327; 17555437; 10383959; 10430882; 10537208; 10903438 |
| NT02NS4530 | 9 | 8 | 0.29 | 20819079; 20136499; 19899769; 19744926; 9930704; 19556511; 19512879; 19112175; 18679055; 18424699 |
| NT02NS4542 | 12 | 12 | 0.96 | 20654624; 20083401; 19723579; 19714768; 19270092; 19238259; 18818204; 18524928; 17244532; 16563798 |
| NT02NS4545 | 4 | 15 | 0.84 | 20471115; 20185507; 19880603; 19850618; 19734308; 19634881; 18261805; 19377339; 19270092; 19202085 |
| NT02NS4570 | 15 | 15 | 0.91 | 20133655; 19369699; 12824373; 18288918; 16194235; 16600681; 18451049; 10579532; 9890992; 11854187 |
| NT02NS4683 | 18 | 18 | 0.97 | 20419429; 20036749; 19577533; 16885437; 19470507; 19440702; 19373193; 19171121; 19095071; 19062291 |
| NT02NS4723 | 6 | 6 | 0.95 | 20813532; 20811460; 20730596; 20708016; 20657823; 20655937; 20569003; 20568999; 20628409; 20540339 |
| NT02NS4742 | 9 | 9 | 0.97 | 20804598; 20816087; 20801191; 20798105; 20736750; 20727852; 20721692; 20707002; 20700461; 20691590 |
| NT02NS4756 | 15 | 15 | 0.88 | 20230832; 19170879; 10637320; 12560545; 17640279; 17244194; 17302809; 16960358; 10079080; 16091943 |
| NT02NS4769 | 9 | 9 | 1.00 | 20622065; 9506993; 19395506; 19028475; 18848863; 18171025; 15904873; 15705744; 15629123; 15531764 |
| NT02NS4810 | 19 | 19 | 0.96 | 20847102; 20843026; 20837514; 20835455; 20832115; 20831591; 20830564; 20828009; 20826214; 20823521 |
| NT02NS4821 | 8 | 8 | 0.94 | 1339433; 16292556; 16212409; 15358267; 15272169; 15045523; 3939535; 10947204; 10686279; 9787093 |
| NT02NS4889 | 4 | 4 | 0.63 | 20802464; 20798162; 20660743; 20659294; 20657594; 20625720; 20552356; 20435732; 20403380; 20236989 |
| NT02NSA0067 | 6 | 6 | 0.81 | 20847942; 20847899; 20844740; 20844047; 20842177; 20838603; 20837705; 20835227; 20833415; 20827200 |
| NT02NSA0112 | 8 | 8 | 0.48 | 20846142; 20809077; 20681546; 20675448; 20582783; 20574459; 20559825; 20549797; 20485834; 20455935 |
| NT02NSA0124 | 5 | 8 | 0.85 | 18825405; 17936114; 17853358; 17261587; 15720402; 12480900; 11527960; 10495709; 2080068; 3132906 |
| NT02NSA0185 | 8 | 8 | 0.59 | 20652274; 18852061; 18298409; 12270927; 15236965; 10331270; 15533878; 15470234; 12887915; 11327708 |
| NT02NSA0291 | 6 | 6 | 0.54 | 20819357; 20818334; 20798330; 20738726; 20716382; 20675469; 20668468; 20668078; 20644968; 20613862 |
| NT02XC0002 | 6 | 6 | 0.97 | 20558179; 20497334; 20297719; 20138014; 20090937; 20017360; 19944654; 19602417; 19592592; 19465649 |
| NT02XC0009 | 3 | 18 | 0.98 | 20846937; 20807744; 20805337; 20802073; 20799747; 20709895; 20696823; 20687614; 20675494; 20647000 |
| NT02XC0040 | 1 | 1 | 0.52 | 20661636; 20630732; 19823932; 15924861; 19226256; 19151600; 19144839; 19119913; 19109486; 18945221 |
| NT02XC0123 | 8 | 8 | 0.75 | 20056735; 19941873; 19774546; 19733226; 19672561; 18519673; 2026135; 18214444; 17658757; 17035713 |
| NT02XC0129 | 6 | 6 | 0.58 | 20603158; 20443037; 20354588; 20299199; 20116856; 20074037; 20061450; 20028838; 20021668; 19923722 |
| NT02XC0161 | 4 | 19 | 0.71 | 20534735; 20439478; 20383007; 20372031; 20372021; 20159575; 20131789; 20118231; 20023090; 19812028 |
| NT02XC0189 | 19 | 8 | 0.51 | 20657172; 9757128; 18249057; 10086842; 17957779; 17189174; 1934134; 14993671; 12387863; 11752785 |
| NT02XC0192 | 15 | 15 | 0.67 | 10606655; 17374605; 5551392; 15819620; 10973967; 6323997; 9409772 |
| NT02XC0197 | 15 | 15 | 0.96 | 20847010; 20846942; 20844002; 20843861; 20843353; 20842669; 20840869; 20840765; 20840068; 20838598 |
| NT02XC0201 | 8 | 8 | 0.66 | 18484998; 10431819; 17669423; 15595825; 16503646; 17116584; 17045293; 12396129; 11480181; 10451366 |
| NT02XC0211 | 18 | 8 | 0.62 | 16332890; 18996845; 18946679; 18832312; 17551845; 17322211; 17080607; 16786427; 16785425; 15906144 |
| NT02XC0218 | 8 | 8 | 0.29 | 19635449; 18801996; 19193103; 18338382; 16962159; 15531590; 2088174 |
| NT02XC0227 | 15 | 15 | 0.60 | 20643148; 20532736; 20493877; 20363937; 20110304; 19648236; 19542280; 19482921; 19438718; 19131333 |
| NT02XC0228 | 3 | 12 | 0.41 | 20847084; 20842477; 20842042; 20840278; 20840173; 20838732; 20838204; 20832196; 20831456; 20828908 |
| NT02XC0266 | 9 | 9 | 0.98 | 20304657; 20221630; 19968566; 19884012; 19863661; 19836235; 19665020; 19664929; 19595597; 19407376 |
| NT02XC0273 | 19 | 2 | 0.73 | 20553505; 20491903; 20359185; 19477415; 19247530; 19050743; 18959400; 10834845; 17557328; 17346033 |
| NT02XC0277 | 19 | 19 | 0.95 | 20445331; 20383009; 19834917; 19770507; 19229285; 19196510; 19053785; 19052362; 19025567; 18774266 |
| NT02XC0281 | 13 | 13 | 0.98 | 20587522; 18948268; 19731322; 19664587; 17900615; 10093218; 17114947; 14566049; 16943774; 15987897 |
| NT02XC0284 | 8 | 8 | 0.80 | 20836675; 20832300; 20824725; 20822851; 20737924; 20730882; 20728346; 20724951; 20693115; 20669055 |
| NT02XC0315 | 8 | 8 | 0.86 | 20460724; 20154126; 18757819; 17657404; 15107237; 14977570; 12829696; 9613842; 9558336; 140652 |
| NT02XC0316 | 6 | 6 | 0.81 | 20846957; 20846404; 20844208; 20843781; 20840787; 20840763; 20834227; 20829609; 20823545; 20823514 |
| NT02XC0318 | 18 | 8 | 0.38 | 19662400; 18663953; 18043868; 16003557; 14717710; 14572657; 14556637; 11665489; 10631329; 10569923 |
| NT02XC0396 | 4 | 4 | 0.56 | 20837997; 20835359; 20834231; 20833893; 20832320; 20828613; 20827338; 20826843; 20825419; 20823697 |
| NT02XC0414 | 8 | 8 | 0.89 | 20618418; 20460875; 19683506; 19381393; 9420328; 19000658; 18655822; 15311063; 18291663; 18252781 |
| NT02XC0415 | 8 | 8 | 0.67 | 20026072; 19823103; 19071198; 9677409; 16797007; 18096698; 16458304; 16004729; 15358267; 12102621 |
| NT02XC0423 | 8 | 8 | 0.84 | 20826223; 20824484; 20814437; 20811039; 20733374; 20728936; 20724705; 20692840; 20668515; 20666792 |
| NT02XC0467 | 4 | 4 | 0.92 | 20646152; 20510343; 20444488; 20355026; 20221680; 20193356; 20122920; 19922143; 19902951; 19809794 |
| NT02XC0471 | 11 | 11 | 0.94 | 20844938; 20842711; 20815820; 20808959; 20803777; 20803249; 20737195; 20725145; 20717103; 20716342 |
| NT02XC0475 | 4 | 19 | 0.86 | 18227251; 3038334; 330527; 2985566; 776981; 6330111; 6296144; 776981 |
| NT02XC0489 | 9 | 9 | 0.99 | 20370610; 20178986; 20145708; 20099411; 20080211; 19924289; 10891285; 19686777; 14693546; 19571038 |
| NT02XC0509 | 8 | 8 | 0.77 | 20462777; 20425116; 20346904; 20002461; 19667196; 19657231; 19586787; 19429050; 11713520; 19054088 |
| NT02XC0521 | 8 | 8 | 0.49 | 18179839; 17298373; 15742151; 15268931; 7606669; 12826056; 12099824; 11815858; 10418145; 7730274 |
| NT02XC0523 | 8 | 8 | 0.27 | 17298373; 16470664; 16175367; 15169669; 11815858; 14208983; 9774536; 9715666; 9680201; 9084662 |
| NT02XC0611 | 9 | 9 | 0.99 | 20725044; 20616867; 20443544; 20169168; 20018655; 19965770; 19560433; 19361526; 17580850; 19263251 |
| NT02XC0641 | 17 | 17 | 0.98 | 19847776; 17075066; 9843952; 12668673; 16980472; 11418118; 10384278; 11823642; 16545925; 10580008 |
| NT02XC0658 | 8 | 8 | 0.58 | 12619701; 10561613; 9851033; 11902724; 9208947; 8620876 |
| NT02XC0661 | 8 | 8 | 0.32 | 12619701; 10561613; 9851033; 9208947; 8620876; 7961952 |
| NT02XC0662 | 8 | 8 | 0.48 | 20136121; 19739187; 19486419; 18607088; 18576402; 18283449; 18258259; 18023797; 17047777; 16865343 |
| NT02XC0667 | 18 | 18 | 0.98 | 20538765; 20622267; 20453874; 20368342; 20363944; 20302929; 20210392; 20190416; 20171189; 20143158 |
| NT02XC0674 | 9 | 9 | 0.92 | 20834152; 20828383; 20815247; 20801908; 20739463; 20688330; 20673827; 20672411; 20670014; 20656812 |
| NT02XC0676 | 8 | 8 | 0.98 | 20841503; 20835892; 20833784; 20833236; 20826660; 20826089; 20824676; 20824107; 20821193; 20820751 |
| NT02XC0677 | 17 | 17 | 0.77 | 20806425; 20798317; 20735390; 20716687; 20687509; 20682311; 20681457; 20677553; 20674573; 20673505 |
| NT02XC0713 | 1 | 1 | 0.67 | 9724324; 10089371; 4981058; 7533594; 8355283; 8515238; 8210673; 1733946; 1897969; 5834235 |
| NT02XC0754 | 12 | 12 | 1.00 | 20825390; 20651228; 20627594; 20600704; 20567601; 20564017; 20560111; 20536450; 20536447; 20444688 |
| NT02XC0788 | 8 | 8 | 0.96 | 20516492; 20513475; 20431245; 20387665; 20371487; 20335826; 20094655; 19963048; 19806079; 19738628 |
| NT02XC0805 | 4 | 4 | 0.29 | 20403380; 20376355; 19956738; 18176015; 17381401; 8675301; 17065365; 11069097; 16754725; 16569707 |
| NT02XC0806 | 19 | 19 | 0.97 | 20594961; 20345654; 20300605; 20199110; 20173067; 20118281; 10708363; 19627504; 19447130; 19422893 |
| NT02XC0811 | 19 | 19 | 0.85 | 20609359; 20163137; 19623961; 17640276; 18700763; 14595026; 1885544; 18173803; 17910958; 7665491 |
| NT02XC0840 | 18 | 15 | 0.97 | 20480360; 20378989; 20168997; 20081031; 19958380; 19832907; 19618914; 19525342; 13838951; 19450606 |
| NT02XC0841 | 2 | 2 | 0.82 | 20550915; 20445263; 19924845; 19631695; 19191740; 18804704; 18007032; 17362087; 17289662; 17029412 |
| NT02XC0842 | 8 | 19 | 0.23 | 20545846; 20519355; 20219462; 20199824; 19916554; 19748480; 19647734; 15118764; 19527033; 2584186 |
| NT02XC0865 | 18 | 18 | 0.96 | 19961827; 19820143; 19434674; 8913593; 18082614; 17976368; 5542675; 17687333; 15032747; 10222271 |
| NT02XC0907 | 19 | 19 | 0.96 | 20837514; 20832115; 20823521; 20815232; 20803262; 20797788; 20734914; 20730755; 20724146; 20723980 |
| NT02XC0924 | 8 | 8 | 0.93 | 20712413; 20498481; 19289099; 9757107; 18452722; 17074835; 17385548; 16915354; 17126551; 16771665 |
| NT02XC0938 | 4 | 8 | 0.28 | 20844906; 20830571; 20799957; 20717661; 20709896; 20705129; 20687061; 20662890; 20645760; 20625734 |
| NT02XC0958 | 19 | 19 | 0.92 | 20394418; 20124190; 19754149; 19546212; 19201821; 9218777; 17621554; 10504382; 11546864; 15654872 |
| NT02XC0980 | 15 | 15 | 0.94 | 20847010; 20833712; 20825659; 20817723; 20732328; 20637800; 20635087; 20622062; 20608176; 20600850 |
| NT02XC1006 | 14 | 8 | 0.40 | 20600125; 20532430; 20512929; 20511407; 20487508; 20483345; 20473326; 20471396; 20442309; 20426806 |
| NT02XC1017 | 18 | 18 | 0.99 | 20847099; 20840536; 20837481; 20804595; 20804585; 20826815; 20826447; 20826203; 20822503; 20816053 |
| NT02XC1053 | 18 | 18 | 0.95 | 20308306; 11872840; 18402979; 19636864; 11737642; 8188372; 8951808; 1315733; 8437515; 1406279 |
| NT02XC1082 | 3 | 3 | 0.99 | 20797400; 20086145; 19891507; 18833195; 19507071; 19390146; 17302815; 19028568; 18083807; 18833195 |
| NT02XC1101 | 17 | 17 | 0.97 | 19963062; 19583999; 2886365; 17233676; 16908155; 16735737; 12426327; 2671751; 15476402; 15346766 |
| NT02XC1125 | 8 | 8 | 0.73 | 20427286; 19405028; 19381641; 18817750; 17728250; 17713928; 17661446; 17166851; 16273368; 16233297 |
| NT02XC1143 | 5 | 5 | 0.43 | 19889875; 5922972; 16666044; 8384204; 2545683; 2824486 |
| NT02XC1175 | 11 | 11 | 0.94 | 20844938; 20842711; 20815820; 20808959; 20803777; 20803249; 20737195; 20725145; 20717103; 20716342 |
| NT02XC1183 | 11 | 11 | 0.94 | 20844938; 20842711; 20815820; 20808959; 20803777; 20803249; 20737195; 20725145; 20717103; 20716342 |
| NT02XC1185 | 6 | 6 | 0.66 | 20675469; 20668078; 20403814; 20071563; 20026132; 19700773; 11839499; 14742714; 18293057; 10399912 |
| NT02XC1191 | 11 | 11 | 0.43 | 20842711; 20553579; 20449818; 20363791; 20306336; 20197135; 19897750; 19893894; 19752751; 19416360 |
| NT02XC1195 | 15 | 15 | 0.59 | 20848286; 20843317; 20833811; 20831411; 20831410; 20831408; 20829395; 20824277; 20823208; 20817772 |
| NT02XC1215 | 15 | 15 | 0.42 | 20848592; 20848558; 20848509; 20848498; 20848491; 20848470; 20848307; 20848249; 20848228; 20848180 |
| NT02XC1217 | 8 | 8 | 0.50 | 20558738; 19631775; 19527071; 9396791; 18486941; 18372246; 17697679; 17640871; 16637010; 16270230 |
| NT02XC1222 | 4 | 4 | 0.46 | 20624248; 20601473; 20109152; 20081323; 20647865; 20009034; 19753649; 19749659; 19631659; 19559652 |
| NT02XC1230 | 8 | 8 | 0.70 | 20846906; 20844868; 20843149; 20841503; 20835892; 20834161; 20833648; 20833236; 20833146; 20832465 |
| NT02XC1239 | 18 | 5 | 0.52 | 20471983; 20388494; 19728176; 19636956; 16922510; 18929666; 18355966; 18243325; 12354788; 17628207 |
| NT02XC1309 | 18 | 18 | 1.00 | 20839889; 20801915; 20796175; 20634426; 20631053; 20532241; 20518729; 20495345; 20484481; 20465544 |
| NT02XC1314 | 18 | 18 | 0.88 | 20221740; 19650773; 19519769; 19075020; 18269247; 17982123; 12193634; 9268330; 17442676; 17222427 |
| NT02XC1315 | 18 | 18 | 0.76 | 20816983; 20693330; 20433837; 20124336; 20574638; 20053141; 19995846; 19882367; 18940858; 19696740 |
| NT02XC1358 | 5 | 8 | 0.22 | 19706507; 19477184; 19131333; 18298373; 1909322; 16359338; 16243452; 15723223; 14960312; 12769681 |
| NT02XC1399 | 19 | 19 | 0.92 | 20825165; 20803478; 20680572; 20668520; 20659891; 20562312; 20545367; 20517582; 20504027; 20431262 |
| NT02XC1431 | 19 | 19 | 0.92 | 20740712; 20466731; 20458497; 20436292; 20362489; 20223486; 20164186; 20164147; 20139147; 20028468 |
| NT02XC1434 | 4 | 4 | 0.90 | 20847516; 20832475; 20823199; 20708964; 20570502; 20305020; 20177063; 20132020; 20105084; 20086084 |
| NT02XC1441 | 1 | 1 | 0.79 | 16627948; 12435751; 10700284; 9207844; 3150686; 6383377 |
| NT02XC1494 | 19 | 19 | 0.52 | 20689719; 18421552; 18045408; 17601167; 17215896; 9179841; 15462530; 15378709; 14964532; 9927663 |
| NT02XC1533 | 4 | 4 | 0.85 | 20496590; 20300516; 19767847; 19754740; 19396959; 19226326; 18978776; 18479253; 18449882; 17200670 |
| NT02XC1537 | 18 | 18 | 0.39 | 20122268; 19665022; 19561611; 12942070; 10331874; 18177941; 16620205; 16386432; 20565676; 14980017 |
| NT02XC1560 | 6 | 4 | 0.20 | 20824060; 20798056; 20713450; 20711849; 20702631; 20655619; 20639326; 20584633; 20552019; 20511495 |
| NT02XC1576 | 4 | 15 | 0.50 | 18319068; 17674066; 10713159; 17329416; 11481430; 16153174; 15251200; 15111059; 15103136; 15100992 |
| NT02XC1607 | 9 | 8 | 0.24 | 20844270; 20837014; 20685973; 20668164; 20388504; 20363896; 20359546; 20359368; 20348210; 20335986 |
| NT02XC1610 | 8 | 8 | 0.24 | 20838379; 20736485; 20733070; 20701481; 20668094; 20604542; 20602206; 20573744; 20547855; 20547757 |
| NT02XC1624 | 9 | 9 | 0.97 | 20652826; 20528952; 20086012; 20025994; 19898564; 19850005; 19656950; 19542079; 19439403; 19341704 |
| NT02XC1684 | 8 | 8 | 0.81 | 17583536; 15644203; 15619452; 11334784; 9293186; 9737851; 9718303; 9665704; 9378722; 9217023 |
| NT02XC1697 | 4 | 8 | 0.38 | 20848677; 20848676; 20848675; 20848674; 20848673; 20848672; 20848671; 20848670; 20848669; 20848668 |
| NT02XC1747 | 4 | 18 | 0.70 | 20847098; 20846813; 20844573; 20844462; 20844038; 20841453; 20838623; 20837715; 20836765; 20833873 |
| NT02XC1799 | 8 | 8 | 0.66 | 20177059; 19895788; 18789896; 17712554; 15983415; 14676989; 11208799; 10411755; 12427935; 7765840 |
| NT02XC1813 | 8 | 12 | 0.83 | 19780766; 9886074; 9750355; 10217831; 8962083; 10391898; 11822362; 10671482; 16943564; 16642333 |
| NT02XC1885 | 18 | 18 | 0.61 | 20216320; 10419969; 10641039; 10734226; 7504664 |
| NT02XC1901 | 11 | 11 | 0.94 | 20844938; 20842711; 20815820; 20808959; 20803777; 20803249; 20737195; 20725145; 20717103; 20716342 |
| NT02XC1923 | 18 | 15 | 0.84 | 20847946; 20847010; 20844005; 20843353; 20837772; 20837600; 20834230; 20834163; 20833896; 20833806 |
| NT02XC1933 | 19 | 8 | 0.27 | 20805402; 20724636; 20673862; 20803814; 20637197; 20616083; 20604568; 20604537; 20603129; 20600001 |
| NT02XC1942 | 8 | 8 | 0.88 | 20595938; 20378006; 20336003; 20215507; 20156442; 20102715; 19858736; 19715479; 10329704; 19703561 |
| NT02XC1962 | 4 | 4 | 0.82 | 20400784; 20127470; 19897753; 19752278; 19353380; 9880479; 18200532; 18096812; 11420333; 17897935 |
| NT02XC1969 | 12 | 12 | 0.58 | 20848653; 20848533; 20848231; 20847583; 20847447; 20847309; 20847183; 20847139; 20847002; 20846517 |
| NT02XC1986 | 12 | 12 | 0.92 | 20847939; 20847730; 20846516; 20824238; 20843518; 20124521; 20357281; 20841351; 20839935; 20838887 |
| NT02XC2009 | 19 | 19 | 0.99 | 20230050; 19826767; 19502744; 18177005; 17709770; 17326805; 17057348; 16473536; 16463105; 16669015 |
| NT02XC2031 | 8 | 8 | 0.94 | 16319331; 19379783; 18455501; 17609257; 16469539; 16453288; 16377227; 16352674; 15988697; 15781197 |
| NT02XC2040 | 8 | 15 | 0.90 | 20676725; 20133655; 19369699; 12824373; 18288918; 16194235; 16600681; 18451049; 10579532; 9890992 |
| NT02XC2082 | 14 | 4 | 0.40 | 20668131; 20566693; 20479198; 20440057; 20420166; 20406494; 20339016; 20086147; 20003904; 19995931 |
| NT02XC2088 | 8 | 15 | 0.98 | 20846144; 20845426; 20843349; 20842632; 20840848; 20837520; 20836077; 20835243; 20833804; 20833746 |
| NT02XC2130 | 8 | 18 | 0.23 | 17623842; 2235490; 15299926; 15503145; 9604894 |
| NT02XC2132 | 4 | 12 | 0.47 | 20368405; 20233932; 20199592; 20185542; 20151761; 20084169; 20023028; 19937033; 19900510; 19840122 |
| NT02XC2141 | 4 | 4 | 0.77 | 20848212; 20848190; 20847723; 20847445; 20846521; 20846484; 20846340; 20846025; 20845086; 20845026 |
| NT02XC2154 | 4 | 19 | 0.92 | 20823121; 20734812; 20720380; 20672305; 20669053; 20668400; 20660607; 20627133; 20567216; 20533194 |
| NT02XC2189 | 9 | 9 | 0.74 | 15221226; 12680762; 1459244; 11099866; 10991936; 10945972; 10427752; 3530497; 6345794 |
| NT02XC2201 | 8 | 8 | 0.46 | 17706591; 17298220; 16824107; 16343536; 15478216; 1850420 |
| NT02XC2220 | 4 | 4 | 0.35 | 19111750; 16790025; 16309817; 10361282; 8231804; 2254246; 323228; 7002911; 4861168 |
| NT02XC2280 | 9 | 9 | 1.00 | 20145252; 19456874; 19401462; 19072541; 17979985; 9211308; 18204094; 15461661; 8663192; 17426021 |
| NT02XC2282 | 9 | 8 | 0.35 | 20534558; 20442401; 20082371; 19925635; 19914586; 19838815; 19699761; 19582448; 19517520; 19506863 |
| NT02XC2308 | 8 | 8 | 0.82 | 19060309; 16849807; 12374303; 11803023; 10746566; 11279023; 11178972; 10216163; 1964456; 9521736 |
| NT02XC2349 | 6 | 6 | 0.59 | 20675469; 20644968; 20561953; 20531307; 20512115; 20403814; 20071563; 20026132; 19960362; 19700773 |
| NT02XC2359 | 18 | 12 | 0.51 | 20843786; 20839801; 20834232; 20813146; 20812868; 20802462; 20801878; 20796029; 20731657; 20725060 |
| NT02XC2420 | 4 | 12 | 0.31 | 20807862; 20103016; 20740013; 20686004; 20726339; 20724636; 20724635; 20718293; 20717865; 20717863 |
| NT02XC2455 | 6 | 13 | 0.42 | 20847638; 20554782; 20221432; 20080137; 20036253; 20012578; 19708815; 19550429; 19380771; 19366610 |
| NT02XC2516 | 5 | 8 | 0.79 | 20846340; 20845308; 20844569; 20833878; 20829289; 20828402; 20828021; 20822129; 20822098; 20813700 |
| NT02XC2522 | 2 | 2 | 0.96 | 19754882; 19267692; 19154787; 18603790; 17460889; 16489629; 16289918; 15983414; 15522295; 15491860 |
| NT02XC2526 | 2 | 2 | 0.97 | 20844580; 20843816; 20699408; 20677756; 20666399; 20606263; 20595262; 20562313; 20495719; 20453090 |
| NT02XC2539 | 2 | 2 | 0.98 | 20699408; 20677756; 20666399; 20606263; 20562313; 20453090; 20385644; 20378651; 20221527; 20219465 |
| NT02XC2550 | 15 | 15 | 0.95 | 20595046; 20537041; 20466770; 20225260; 20190084; 19837838; 18587152; 19389781; 19307761; 9643542 |
| NT02XC2561 | 12 | 13 | 0.21 | 19440203; 19186990; 17891155; 3049606; 10368282; 16977663; 9407082; 8798470; 7657641 |
| NT02XC2564 | 12 | 12 | 0.90 | 19726681; 18835567; 17306546; 16135238; 15037248; 12235156 |
| NT02XC2608 | 19 | 19 | 0.38 | 18499663; 16829524; 15820665; 15226299; 12758148; 11846551; 10610795 |
| NT02XC2612 | 4 | 4 | 0.86 | 19889085; 19665005; 12864857; 16677309; 15170399; 10320579; 10572114; 9286988; 2982790 |
| NT02XC2621 | 4 | 4 | 0.69 | 20439729; 10468575; 17085552; 17015641; 11279165; 16677309; 3007433; 10440379; 7928960; 6374019 |
| NT02XC2623 | 4 | 4 | 0.84 | 20639318; 20615986; 20610778; 20586476; 20543831; 20534509; 20525830; 20512252; 20511507; 20501700 |
| NT02XC2624 | 4 | 4 | 0.82 | 20703316; 20691071; 20639318; 20615986; 20610778; 20586476; 20582761; 20576688; 20547100; 20543831 |
| NT02XC2685 | 4 | 4 | 0.29 | 18514223; 15175278; 14731274; 12196531; 12119291; 11669626; 11435446; 10231491; 2168368; 7608974 |
| NT02XC2703 | 13 | 12 | 0.37 | 20668033; 20499635; 20358348; 20206339; 20159010; 20053925; 19924283; 19720044; 19692652; 16115052 |
| NT02XC2711 | 17 | 15 | 0.64 | 20844554; 20844019; 20826955; 20817065; 20816986; 20807197; 20799623; 20735431; 20729154; 20714214 |
| NT02XC2726 | 8 | 8 | 0.47 | 20077187; 19932602; 19460629; 19459965; 19242819; 19177531; 19036343; 18312412; 18080783; 17692550 |
| NT02XC2743 | 18 | 8 | 0.70 | 20847417; 20846162; 20843610; 20838584; 20837547; 20835271; 20825285; 20822828; 20815922; 20815874 |
| NT02XC2774 | 4 | 18 | 0.53 | 12650999; 9350866; 9612954; 1365905; 6394977 |
| NT02XC2779 | 11 | 4 | 0.44 | 20805406; 20797416; 20737195; 20734919; 20734751; 20731086; 20731027; 20719828; 20718342; 20718338 |
| NT02XC2813 | 4 | 4 | 0.65 | 20838602; 20838207; 20833722; 20830571; 20814187; 20803499; 20803133; 20800080; 20739506; 20738841 |
| NT02XC2917 | 4 | 4 | 0.86 | 20054112; 19021761; 17561111; 16363530; 15573748; 15489502; 12595266; 9193092; 11524683; 11264598 |
| NT02XC2947 | 19 | 8 | 0.37 | 20690427; 20684602; 20670895; 20661248; 20650040; 20648017; 20643254; 20628391; 20622068; 20620155 |
| NT02XC2949 | 19 | 19 | 0.65 | 18499663; 18325534; 16829524; 15820665; 15226299; 12758148; 11846551; 10610795; 10521532; 8878483 |
| NT02XC3001 | 6 | 6 | 0.94 | 19648246; 18682229; 17993536; 17619258; 9767087; 15223318; 14636570; 1644310; 10869041; 12686634 |
| NT02XC3008 | 8 | 12 | 0.46 | 20690544; 20615417; 20601363; 20561916; 20546141; 20538007; 20519628; 20519524; 20496344; 20487283 |
| NT02XC3043 | 1 | 1 | 0.37 | 20480196; 19948253; 19776011; 18171624; 19248588; 17506527; 17176071; 17115705; 9651254; 15753077 |
| NT02XC3183 | 14 | 14 | 0.58 | 20837458; 20819423; 20815815; 20811661; 20798559; 20738018; 20727673; 20725619; 20723540; 20714877 |
| NT02XC3191 | 1 | 8 | 0.50 | 20398676; 19279014; 16615917; 16513644; 4074072; 15662561; 12354106; 12581215; 11905963; 11790331 |
| NT02XC3203 | 15 | 18 | 0.41 | 20644138; 20571957; 20562310; 20518707; 20437149; 20430815; 20421381; 20404170; 20386693; 20372024 |
| NT02XC3207 | 13 | 13 | 0.98 | 20348441; 20346161; 20073035; 20038631; 19883769; 19805312; 19776006; 19749382; 19540849; 10564509 |
| NT02XC3261 | 8 | 8 | 0.96 | 20737579; 20695524; 20690702; 20632184; 20600130; 20547565; 20534338; 20471952; 20453296; 20410318 |
| NT02XC3278 | 18 | 18 | 0.96 | 20844762; 20834157; 20817945; 20804453; 20724170; 20722436; 20717788; 20716818; 20696879; 20691272 |
| NT02XC3287 | 19 | 19 | 0.37 | 19819716; 16914867; 18667582; 16905539; 10048339; 9087403; 11080627; 10945972; 3897200; 8021212 |
| NT02XC3299 | 8 | 12 | 0.24 | 20848498; 20848490; 20847443; 20846184; 20845810; 20845807; 20823715; 20823714; 20823711; 20845075 |
| NT02XC3328 | 6 | 6 | 0.66 | 20554496; 20546727; 20499638; 20442410; 20337945; 20212040; 20195500; 20123583; 19951465; 19943934 |
| NT02XC3336 | 15 | 15 | 0.32 | 20847938; 20847317; 20847230; 20846517; 20844147; 20843676; 20843353; 20841353; 20840906; 20839371 |
| NT02XC3347 | 18 | 18 | 0.96 | 20421301; 20090677; 20078865; 19923217; 19878597; 19806386; 19701722; 19670073; 19640852; 19605523 |
| NT02XC3356 | 18 | 8 | 0.89 | 20718446; 20696493; 20635418; 20632184; 20629748; 20614892; 20606288; 20553812; 20547883; 20538075 |
| NT02XC3368 | 13 | 8 | 0.26 | 16616438; 18230623; 17389594; 16813579; 15118328; 12946416; 11682473; 11314949 |
| NT02XC3391 | 8 | 12 | 0.91 | 20847217; 20846409; 20845960; 20844575; 20844218; 20843828; 20842208; 20841485; 20841355; 20840862 |
| NT02XC3419 | 15 | 15 | 0.49 | 20833806; 20713134; 19682264; 19646527; 18800033; 18602497; 18501443; 18051605; 18022383; 17999647 |
| NT02XC3443 | 12 | 12 | 0.99 | 20739286; 20581826; 20581825; 20515934; 20515644; 20512978; 20510014; 20487295; 20487271; 20437142 |
| NT02XC3445 | 17 | 17 | 0.91 | 20817765; 19956571; 19682265; 18713062; 10428967; 9778730; 16385111; 16343331; 10894748; 9721325 |
| NT02XC3462 | 19 | 19 | 0.72 | 20844924; 20830915; 20825165; 20809667; 20823121; 20822177; 20821520; 20821204; 20815234; 20812985 |
| NT02XC3519 | 4 | 4 | 0.86 | 20481475; 20056514; 19926855; 19683782; 19577534; 19343712; 19265437; 19199786; 18810541; 18690716 |
| NT02XC3520 | 13 | 13 | 0.90 | 20109665; 15800628; 11337501; 16859720; 15469495; 14578863; 9428306; 1672854; 2605256; 3647910 |
| NT02XC3532 | 19 | 19 | 0.88 | 20070105; 18473942; 18468997; 11988506; 10091328; 9933916; 7764221; 2311931 |
| NT02XC3555 | 15 | 15 | 0.91 | 18757816; 16788195; 14500536; 17575448; 11418146; 10358053; 15812007; 8824586; 6111556; 16171954 |
| NT02XC3565 | 19 | 12 | 0.95 | 20843040; 20832304; 20823284; 20818167; 20716448; 20689770; 20685859; 20670659; 20668084; 20660367 |
| NT02XC3568 | 8 | 8 | 0.88 | 16863643; 15910742; 15475358; 15184552; 11017202; 10544288; 10486564; 10471783; 10393339; 9737851 |
| NT02XC3569 | 8 | 8 | 0.88 | 10544288; 10486564; 10471783; 10393339; 9737851; 9665692; 9548917; 9443817; 9298948; 9217023 |
| NT02XC3584 | 9 | 8 | 0.28 | 20848677; 20848674; 20848673; 20848668; 20848659; 20848653; 20848643; 20848642; 20848615; 20848614 |
| NT02XC3591 | 12 | 8 | 0.26 | 7607232; 10368438; 6374664; 9656480; 8275013; 1416979; 2005135; 3528671; 3089323; 3844013 |
| NT02XC3636 | 18 | 18 | 0.93 | 20477749; 19698103; 19496824; 19180638; 18662319; 17485078; 17005992; 16629678; 16382870; 8494534 |
| NT02XC3644 | 4 | 4 | 0.45 | 19595787; 15371447; 17631493; 17029242; 10519556; 15260498; 15231835; 10519556; 12173933; 11376004 |
| NT02XC3679 | 6 | 8 | 0.60 | 20599730; 20544026; 20529601; 20402814; 20358345; 20302878; 20215775; 20054126; 19601747; 19490764 |
| NT02XC3699 | 12 | 12 | 0.96 | 20817636; 20732391; 20601056; 20540562; 19879248; 19821612; 19773392; 19762340; 19715703; 19703590 |
| NT02XC3725 | 3 | 3 | 1.00 | 20616068; 20348256; 20338182; 20145101; 20133577; 19717595; 19589067; 19423631; 19127589; 17873043 |
| NT02XC3737 | 9 | 9 | 0.98 | 20594840; 20557983; 20418430; 20304657; 20221630; 20153183; 20038703; 19968566; 19884012; 19863661 |
| NT02XC3752 | 6 | 6 | 0.90 | 20675375; 20223211; 16507358; 16430690; 15758241; 12940977; 9144786; 12535532; 11859073; 11823461 |
| NT02XC3758 | 2 | 2 | 0.95 | 20606263; 20038586; 19566093; 2843765; 9692897; 2025413; 9667924; 10903949; 10622725; 10411269 |
| NT02XC3787 | 8 | 8 | 0.64 | 20847235; 20805365; 20735360; 20713124; 20702649; 20692206; 20686920; 20665018; 20664702; 20661717 |
| NT02XC3797 | 13 | 13 | 0.99 | 20833785; 20631085; 20088384; 19774386; 19359250; 19344311; 19334533; 19196767; 19140323; 12082018 |
| NT02XC3815 | 8 | 8 | 0.65 | 20500527; 20490604; 20185233; 20127146; 20027149; 19803089; 19787347; 15353566; 19587775; 19034559 |
| NT02XC3872 | 13 | 13 | 0.98 | 20413480; 19111651; 10937989; 7516168; 6093096 |
| NT02XC3882 | 13 | 13 | 0.98 | 20399793; 9202191; 15989950; 10805779; 10747797; 8123703; 1764524; 6360687; 7049235; 6125208 |
| NT02XC3886 | 12 | 12 | 0.78 | 20444093; 10594836; 18683629; 17699162; 10633125; 16243836; 16242710; 12507766; 15546659; 15168501 |
| NT02XC3895 | 14 | 14 | 0.92 | 20544509; 20380929; 20021999; 19918830; 19754463; 19186537; 19161981; 19131690; 18262821; 2483028 |
| NT02XC3910 | 17 | 13 | 0.88 | 19366704; 10625642; 3038334; 8422961; 2166215; 6997720 |
| NT02XC3913 | 19 | 19 | 0.90 | 20838850; 20824487; 20821249; 20816933; 20814725; 20803244; 20736135; 20730772; 20730475; 20727822 |
| NT02XC3939 | 4 | 4 | 0.82 | 20806779; 20476795; 19735955; 19572210; 18084891; 17185549; 17141802; 17098255; 16307478; 16214169 |
| NT02XC3944 | 8 | 8 | 0.86 | 20845075; 20826743; 20816840; 20600873; 20570330; 20558052; 20554191; 20471957; 20471925; 20194725 |
| NT02XC3980 | 6 | 8 | 0.94 | 20510668; 20452977; 20435721; 20370825; 20153315; 20137101; 20110298; 20081038; 20042216; 20040374 |
| NT02XC3983 | 15 | 15 | 0.97 | 20594941; 20133363; 18611278; 12563033; 10637320; 18039772; 18604637; 18093135; 18022383; 11728723 |
| NT02XC3992 | 4 | 4 | 0.82 | 17512907; 17159217; 15846595; 12142492; 11251828; 11160799; 9324261 |
| NT02XC4016 | 8 | 8 | 0.89 | 20601653; 20302299; 20218714; 20162368; 20147623; 19932076; 19892731; 19633968; 19472231; 19362514 |
| NT02XC4031 | 18 | 14 | 0.80 | 20839414; 20829431; 20824050; 20814156; 20812847; 20807311; 20735779; 20735412; 20734919; 20732875 |
| NT02XC4059 | 8 | 8 | 0.92 | 19453508; 18926808; 17669536; 17150757; 16556607; 12824170; 16326697; 16242617; 15691337; 12824170 |
| NT02XC4083 | 19 | 19 | 0.81 | 20383009; 19052362; 9054558; 12566566; 14659047; 10605111; 9371343; 10066483; 11902729; 9054558 |
| NT02XC4136 | 19 | 19 | 0.85 | 11752577; 14740209; 12465714; 11351091; 11732051; 8901635; 8901536; 8806566; 1830823; 2549367 |
| NT02XC4180 | 12 | 12 | 0.98 | 20803144; 20706583; 20652663; 20472642; 20440617; 20389065; 20346382; 20334618; 20057138; 20032414 |
| NT02XC4186 | 19 | 19 | 0.58 | 8625428; 8830237; 8012046; 3322811; 7012838; 2215211; 3310898 |
| NT02XC4193 | 9 | 9 | 0.98 | 20844583; 20713103; 20145252; 20132453; 20085613; 19932173; 19797465; 19666474; 19456874; 19433071 |
| NT02XC4205 | 8 | 4 | 0.28 | 19297951; 18247503; 17363272; 16966321; 15528667; 11390701; 10499357; 9863770 |
| NT02XC4218 | 19 | 19 | 0.92 | 20837469; 20832776; 20817766; 20816477; 20816394; 20816222; 20816221; 20816216; 20816177; 20816175 |
| NT02XC4234 | 1 | 8 | 0.75 | 20839901; 20836991; 20828387; 20826746; 20825409; 20797558; 20821054; 20817827; 20813156; 20802128 |
| NT02XC4245 | 2 | 8 | 0.28 | 19830588; 19704083; 19674121; 19534905; 19322676; 19234759; 19172809; 19099731; 18505119; 18332253 |
| NT02XC4254 | 9 | 9 | 0.99 | 19854834; 17360388; 19550039; 19472174; 18804030; 12221980; 10222271; 16392678; 16953632; 16710404 |
| NT02XC4335 | 15 | 15 | 0.98 | 20729367; 20509863; 20139635; 20086039; 20049751; 20027290; 19906180; 19889099; 19775246; 19703979 |
| NT02XC4375 | 4 | 6 | 0.81 | 19996303; 11371527; 10504701; 16024043; 8538772; 15590328; 10497267; 15135731; 10521502; 12574322 |
| NT02XC4483 | 17 | 6 | 0.94 | 20813532; 20708016; 20705241; 20569003; 20526281; 20472641; 20452362; 20382767; 20381373; 20308527 |
| NT02XC4549 | 8 | 8 | 0.87 | 20844980; 20827217; 20816850; 20807763; 20799369; 20734249; 20732359; 20729871; 20727984; 20714159 |
| NT02XC4589 | 8 | 6 | 0.71 | 20835928; 20623640; 20594292; 20485382; 20450923; 20395215; 20385554; 20233511; 20224894; 20173781 |
| NT02XC4592 | 19 | 19 | 0.90 | 20331963; 18563288; 18387370; 18355438; 16284928; 16280320; 16055313; 15913610; 15669674; 12867081 |
| NT02XC4598 | 14 | 6 | 0.45 | 20823546; 20813141; 20601405; 20493855; 20480232; 20473284; 20466055; 20461535; 20447418; 20412059 |
| NT02XC4636 | 12 | 12 | 0.51 | 20847939; 20847730; 20847047; 20846931; 20846825; 20846516; 20824238; 20843949; 20843518; 20843040 |
| NT02XC4645 | 4 | 8 | 0.92 | 20609914; 19549597; 18991392; 18981569; 18945673; 18930846; 18835810; 18472393; 18211101; 10438728 |
| NT02XC4660 | 12 | 12 | 0.89 | 20839050; 20825230; 20824277; 20803144; 20723691; 20655071; 20632945; 20592285; 20578976; 20557572 |
| NT02XC4721 | 4 | 4 | 0.87 | 20810565; 20801895; 20637840; 20458498; 20388554; 20364116; 20347322; 20508664; 20221545; 20199868 |
| NT02XC4743 | 4 | 8 | 0.55 | 20628010; 18979475; 17876823; 17260957; 16919347; 16572225; 16544142; 16195462; 15850981; 15645721 |
| NT02XC4748 | 12 | 12 | 0.98 | 20848425; 20838651; 20837477; 20828613; 20818167; 20816329; 20812385; 20808763; 20799349; 20799311 |
| NT02XC4777 | 19 | 19 | 0.98 | 20562284; 20057070; 19626289; 19280987; 7226224; 18559265; 18337693; 18309273; 18043952; 17234634 |
| NT02XC4781 | 9 | 9 | 0.99 | 20418430; 20178986; 20158476; 20153183; 19664929; 19550039; 19472174; 19214500; 18804030; 18824113 |
| NT02XC4793 | 8 | 9 | 0.98 | 20798351; 20797606; 20690600; 20667975; 20620995; 20607591; 20601073; 20558738; 20534558; 20530735 |
| NT02XC4797 | 9 | 9 | 0.47 | 20847127; 20845345; 20843517; 20843317; 20837491; 20837115; 20837014; 20834178; 20821248; 20813182 |
| NT02XC4798 | 18 | 8 | 0.21 | 20503218; 20449476; 20194504; 20154113; 19942143; 19928853; 19860832; 19854930; 19680888; 19657693 |
| NT02XC4813 | 4 | 4 | 0.46 | 20660083; 20507997; 20472940; 20395279; 19930663; 11931551; 19578062; 19368887; 18292935; 18087702 |
| NT02XC4820 | 4 | 19 | 0.45 | 20837038; 20827470; 20810648; 20801036; 20799407; 20798855; 20795405; 20735991; 20734924; 20724527 |
| NT02XC4869 | 18 | 18 | 0.94 | 18602023; 17932225; 17559959; 17306760; 9278503; 16907860; 15358349; 15287594; 14973265; 12738772 |
| NT02XC4876 | 19 | 19 | 0.90 | 20140934; 20179984; 19384899; 10575553; 18700763; 18599029; 18596046; 17350598; 18330718; 18206726 |
| NT02XC4916 | 6 | 6 | 0.95 | 20847952; 20847817; 20847693; 20847282; 20847234; 20847082; 20847055; 20847044; 20846458; 20846433 |
| NT02XC5014 | 6 | 6 | 0.95 | 20847381; 20837039; 20806260; 20800503; 20699274; 20649618; 20595677; 20509895; 20497557; 20444879 |
| NT02XC5026 | 18 | 4 | 0.27 | 20472527; 20466484; 20459395; 20160437; 19951434; 19941879; 19940183; 19680222; 19619572; 19541925 |
| NT02XC5034 | 18 | 4 | 0.89 | 19785390; 10973494; 19543944; 19150644; 19089528; 17768259; 10489822; 18286224; 17890764; 17504492 |
| NT02XC5057 | 8 | 8 | 0.80 | 20413649; 19690808; 11071754; 18675808; 10762278; 18281624; 18207028; 15634195; 9772162; 16865707 |
| NT02XC5058 | 18 | 12 | 0.22 | 20848220; 20834163; 20831872; 20802377; 19571053; 20796192; 20689758; 20670983; 20650000; 20639461 |
| NT02XC5079 | 17 | 17 | 0.68 | 20334683; 20133747; 19430703; 17081564; 10393536; 9757830; 15708007; 7730292; 16574071; 16253270 |
| NT04SP0025 | 6 | 6 | 0.30 | 20798231; 20731826; 20720114; 20697568; 20655081; 20637039; 20609081; 20602915; 20568728; 20547102 |
| NT04SP0045 | 15 | 12 | 0.30 | 20798394; 20687872; 20582921; 20568296; 20560978; 20553486; 20534755; 20504764; 20481436; 20456369 |
| NT04SP0106 | 12 | 12 | 0.96 | 20652663; 20440617; 20389065; 20346382; 20334618; 20057138; 20032414; 20027867; 19914209; 19913106 |
| NT04SP0112 | 14 | 18 | 0.27 | 20606317; 20544526; 20544525; 20544523; 20544522; 20231284; 20208581; 20008099; 20005183; 19913594 |
| NT04SP0113 | 15 | 15 | 0.60 | 20840765; 20836032; 20834163; 20830586; 20826533; 20736066; 20730086; 20713551; 20713134; 20711226 |
| NT04SP0115 | 15 | 15 | 0.91 | 20831799; 20830236; 20805337; 20732431; 20724482; 20723231; 20709902; 20709896; 20708625; 20688829 |
| NT04SP0143 | 18 | 18 | 0.79 | 20511227; 20080582; 19834508; 19698777; 19630402; 19473972; 19344662; 19299516; 19289121; 19211918 |
| NT04SP0144 | 4 | 4 | 0.68 | 20430752; 18757817; 18067540; 17253982; 16101295; 12501248; 11401721; 10501032; 7616962; 8022272 |
| NT04SP0184 | 8 | 8 | 0.41 | 20839935; 20826560; 20826128; 20819951; 20810510; 20807655; 20806246; 20712627; 20696780; 20672443 |
| NT04SP0189 | 18 | 12 | 0.93 | 20621843; 20558178; 20523889; 20068166; 20064458; 20045481; 19862556; 19841675; 19761755; 19680265 |
| NT04SP0205 | 15 | 15 | 0.98 | 20629796; 20501872; 20447287; 20383008; 20127467; 20038702; 20019083; 19897756; 19832907; 19783633 |
| NT04SP0239 | 18 | 18 | 0.67 | 20395534; 19853682; 19063603; 18584243; 16702222; 16166347; 7845353; 12434407; 12388190; 11133151 |
| NT04SP0244 | 19 | 19 | 0.98 | 20470363; 20364833; 20188057; 20077550; 20030628; 19929855; 19720067; 19277539; 19245333; 19159314 |
| NT04SP0251 | 19 | 19 | 0.98 | 20847002; 20843347; 20726582; 20713676; 20608745; 20593835; 20580675; 20547785; 20542210; 20525686 |
| NT04SP0257 | 18 | 18 | 0.93 | 20843810; 20812950; 20658302; 20651349; 20466975; 20619549; 20600004; 20584751; 20583551; 20529607 |
| NT04SP0282 | 18 | 18 | 0.89 | 18442087; 15337171; 10886565; 10811962; 7890707 |
| NT04SP0350 | 18 | 18 | 0.24 | 20831904; 20799727; 20726513; 20711450; 20696237; 20681974; 20655381; 20626553; 20617034; 20599845 |
| NT04SP0352 | 4 | 4 | 0.67 | 19717602; 19502436; 19291237; 19291024; 19285953; 19016868; 19179513; 18156657; 17388894; 17268768 |
| NT04SP0355 | 8 | 8 | 0.83 | 20664851; 20653766; 20566279; 20518470; 20449373; 20395268; 20356276; 20350544; 20298874; 20180558 |
| NT04SP0364 | 14 | 8 | 0.40 | 20548285; 20516613; 20462199; 20448188; 20044377; 19899807; 19821570; 19724122; 19632469; 19061340 |
| NT04SP0375 | 18 | 18 | 0.86 | 20565143; 18798051; 18343875; 17079719; 9827570; 15927004; 15589830; 11443103; 12576593; 8423001 |
| NT04SP0423 | 2 | 8 | 0.40 | 19876400; 19763791; 5333155; 15472313; 15526298; 14514684; 12837541; 10089316; 10480925; 11980892 |
| NT04SP0430 | 12 | 12 | 0.98 | 19329603; 18501515; 10834952; 18478957; 18429106; 18318009; 17979195; 17309236; 17123646; 17119851 |
| NT04SP0453 | 4 | 6 | 0.27 | 20360008; 15175338; 17005570; 12654244; 15634680; 14612982; 10353244; 11707343; 11470800; 11178891 |
| NT04SP0525 | 18 | 18 | 0.97 | 20573858; 19904717; 19805341; 19706285; 19671701; 19645001; 19411703; 19389160; 19327826; 19205586 |
| NT04SP0560 | 18 | 18 | 0.98 | 20798504; 20723594; 20665694; 20592244; 20544961; 20507120; 20483324; 20385786; 20338165; 20226783 |
| NT04SP0603 | 12 | 12 | 0.96 | 20832960; 20828778; 20824169; 20811517; 20806899; 20731414; 20702722; 20696584; 20682085; 20670659 |
| NT04SP0615 | 19 | 19 | 0.87 | 20817766; 20097205; 19959572; 19796091; 19744987; 19727825; 19126547; 19052376; 19047740; 10832095 |
| NT04SP0645 | 2 | 2 | 0.89 | 20405048; 15896804; 14757766; 11443125; 10781607; 10722656; 4932656; 3133359; 9632726; 6284709 |
| NT04SP0655 | 18 | 18 | 0.96 | 20041985; 19460095; 17660412; 10676647; 17314234; 16854981; 10103220; 15820655; 12802507; 10501939 |
| NT04SP0679 | 19 | 19 | 0.93 | 20847047; 20844865; 20738399; 20642807; 20616191; 20598082; 20435778; 20418257; 20411671; 20402773 |
| NT04SP0694 | 15 | 15 | 0.85 | 9891801; 12925133; 16547644; 16000707; 13129619; 10510225 |
| NT04SP0700 | 2 | 8 | 0.44 | 20822187; 20534558; 20525309; 20508999; 20508290; 20493886; 20487541; 20395233; 20393702; 20360620 |
| NT04SP0710 | 18 | 8 | 0.50 | 20821202; 20546591; 20511421; 20453910; 20453090; 20303688; 20134249; 20084284; 20025242; 20006108 |
| NT04SP0713 | 14 | 14 | 0.99 | 8609628; 17299348; 16305529; 16000004; 15983408; 15581562; 15571394; 15561147; 12402029; 12810542 |
| NT04SP0714 | 15 | 15 | 0.94 | 20622066; 20593779; 20581213; 20565117; 20525830; 20498088; 20480360; 20471402; 20459101; 20447287 |
| NT04SP0720 | 18 | 18 | 0.98 | 20154136; 20132828; 15673787; 17322901; 17171468; 11756427; 16445940; 16135226; 9579077; 14561746 |
| NT04SP0732 | 4 | 12 | 0.50 | 20135153; 18616471; 10601861; 17428499; 16722231; 16488470; 16088218; 15805776; 9585179; 15322131 |
| NT04SP0734 | 4 | 15 | 0.37 | 20831651; 20829395; 20728315; 20686601; 20625909; 20579227; 20500497; 20467217; 20382218; 20305014 |
| NT04SP0764 | 8 | 8 | 0.91 | 20160912; 19996100; 16531404; 14644451; 12603319; 11955070; 11935326; 11781147; 11708858; 11673873 |
| NT04SP0771 | 2 | 8 | 0.29 | 11148030; 17212407; 11007789; 10966576; 1828761; 2234077; 7850430; 2247450 |
| NT04SP0799 | 11 | 6 | 0.53 | 20848253; 20798162; 20724527; 20662778; 20581737; 20552356; 20472796; 20435728; 20387457; 20383760 |
| NT04SP0855 | 15 | 15 | 0.98 | 20447287; 20038702; 19897756; 19783633; 9891801; 19460100; 16497924; 15304477; 18048908; 12515534 |
| NT04SP0882 | 18 | 8 | 0.41 | 20448665; 20420430; 20385961; 20354774; 20237115; 20203153; 20003133; 19843634; 19787704; 19654186 |
| NT04SP0932 | 4 | 4 | 0.65 | 20847516; 20837039; 20832475; 20829608; 20829288; 20823199; 20822022; 20817802; 20816689; 20814890 |
| NT04SP0942 | 8 | 8 | 0.23 | 20543879; 18081320; 15670164; 14529271; 12071964; 11802728; 11248184; 10757980; 10694397; 10521253 |
| NT04SP0969 | 2 | 2 | 0.81 | 10642176; 18241665; 14722069; 15499907; 14527557; 12829729; 10604969; 10395444; 7860596; 9274862 |
| NT04SP1007 | 9 | 14 | 0.55 | 9647837; 17340635; 17123102; 17112654; 17068342; 3447015; 16246027; 15858264; 8366139; 14699425 |
| NT04SP1038 | 8 | 8 | 0.91 | 20833871; 20732951; 20707314; 20695200; 20686672; 20686173; 20684233; 20679207; 20675600; 20655901 |
| NT04SP1091 | 11 | 6 | 0.28 | 20497505; 20197281; 19883660; 19748809; 19297330; 18834851; 17145704; 18687036; 18055467; 18033872 |
| NT04SP1108 | 6 | 11 | 0.97 | 20627350; 20578458; 20406289; 20298189; 20111606; 20022231; 20007650; 19941653; 19487729; 19465660 |
| NT04SP1114 | 18 | 18 | 0.97 | 20132828; 18957589; 17920277; 8768517; 12215814; 3023298; 11282467; 11289518; 7608087; 10817516 |
| NT04SP1115 | 18 | 18 | 0.93 | 20221889; 20173761; 20147285; 20132828; 19748784; 19636938; 19630440; 19395376; 19274733; 12893936 |
| NT04SP1183 | 14 | 8 | 0.19 | 19061340; 8181757; 2834367; 2846540; 3289734; 2829920; 6343369 |
| NT04SP1195 | 12 | 12 | 0.92 | 20591501; 20559454; 20023111; 19906959; 19892921; 19740516; 19684063; 19607812; 19598126; 19388144 |
| NT04SP1200 | 15 | 6 | 0.37 | 20845385; 20844054; 20842122; 20840511; 20831799; 20829431; 20827602; 20827587; 20826812; 20818439 |
| NT04SP1208 | 4 | 4 | 0.89 | 20583283; 20144594; 20082851; 20060057; 19938450; 19620365; 19513562; 19458032; 19403001; 19400770 |
| NT04SP1237 | 4 | 19 | 0.36 | 20847216; 20825156; 20727013; 20714354; 20665904; 20652718; 20635098; 20622130; 20610395; 20575441 |
| NT04SP1307 | 6 | 6 | 0.83 | 20438097; 20227927; 20222748; 20184295; 20166732; 20133751; 20000331; 19955559; 19954157; 19945437 |
| NT04SP1337 | 15 | 15 | 0.93 | 20150239; 19648249; 19346355; 11157930; 1712012; 1784825; 2265755; 11511866; 9846747; 9237914 |
| NT04SP1402 | 6 | 6 | 0.97 | 20846960; 20842320; 20842177; 20841565; 20837992; 20837540; 20837503; 20837020; 20835798; 20834227 |
| NT04SP1421 | 8 | 8 | 0.28 | 12421312; 9068642; 9473054; 10215894; 10844653; 10632883; 9353916; 2449095; 8253680 |
| NT04SP1460 | 3 | 12 | 0.51 | 20513375; 19304841; 8736544; 18445019; 10429941; 16944959; 16549657; 10784058; 8622910; 14527287 |
| NT04SP1545 | 15 | 15 | 0.65 | 20594961; 20552019; 20059681; 19760662; 19602148; 15937167; 18781356; 18599838; 18451049; 10831436 |
| NT04SP1562 | 12 | 12 | 0.97 | 18946116; 15378750; 12741537; 9810230; 9004508; 11204766; 10208803; 6852022; 8620487; 8568031 |
| NT04SP1581 | 6 | 19 | 0.30 | 20827334; 19157945; 12202772; 19053819; 18991290; 18674571; 18524927; 18342633; 18251396; 10482520 |
| NT04SP1614 | 11 | 11 | 0.90 | 20848296; 20844906; 20825156; 20803062; 20802084; 20709901; 20709082; 20708437; 20665904; 20662890 |
| NT04SP1616 | 6 | 6 | 0.78 | 20663713; 18615848; 17261809; 17942449; 10339623; 17419590; 17207772; 16941645; 15615704; 15494305 |
| NT04SP1659 | 19 | 8 | 0.86 | 20427286; 18215050; 10885988; 14993682; 10080900; 10322172; 11705942; 11940603; 10712619; 13331868 |
| NT04SP1675 | 13 | 13 | 0.98 | 20839046; 20797924; 20734330; 20718291; 20695782; 20675586; 20670890; 20668900; 20696925; 20657646 |
| NT04SP1714 | 12 | 12 | 0.97 | 20847048; 20841453; 20827447; 20803087; 20735358; 20729526; 20727857; 20668094; 20648511; 20615403 |
| NT04SP1715 | 12 | 12 | 0.97 | 20847048; 20832865; 20813055; 20732327; 20729526; 20727857; 20724383; 20655455; 20654624; 20648511 |
| NT04SP1799 | 15 | 15 | 0.82 | 10517579; 10915804; 1494353; 9141695; 8153625; 3156376 |
| NT04SP1807 | 15 | 15 | 0.48 | 20133363; 18611278; 12563033; 18039772; 18022383; 17483938; 17322187; 14645274; 10464216; 10564501 |
| NT04SP1849 | 19 | 8 | 0.60 | 19625389; 18615662; 18187645; 10383959; 16917793; 14526081; 12536257; 12351228; 11330713; 10906347 |
| NT04SP1901 | 4 | 8 | 0.66 | 20836298; 20825845; 20802413; 20717006; 20693135; 20690092; 20687276; 20683324; 20682069; 20680641 |
| NT04SP1949 | 4 | 4 | 0.60 | 20841492; 20708459; 20567883; 20458278; 20132182; 20109735; 19855073; 19812863; 19801665; 19740318 |
| NT04SP1956 | 4 | 19 | 0.70 | 20471679; 20195537; 20045426; 20026416; 19902966; 19801468; 19493306; 19436046; 19381366; 19291007 |
| NT04SP1976 | 12 | 12 | 0.96 | 20383012; 20352460; 20331799; 20299243; 20194785; 20159025; 20026003; 19936251; 19772795; 19706612 |
| NT04SP1994 | 15 | 15 | 0.95 | 20844764; 20843784; 20838651; 20831632; 20826812; 20823465; 20823325; 20820770; 20819073; 20817745 |
| NT04SP2003 | 9 | 15 | 0.98 | 20730247; 20716550; 20678501; 20643656; 20162616; 20012281; 19586910; 19542281; 19501097; 18041902 |
| NT04SP2035 | 15 | 15 | 0.96 | 17185537; 8446025; 16430705; 14568530; 10411736 |
| NT04SP2070 | 4 | 12 | 0.50 | 20848398; 20820878; 20671927; 20659158; 20650410; 20610477; 20610040; 20584105; 20582409; 20574026 |
| NT08MM10029 | 8 | 8 | 0.59 | 15927749; 15159592; 9274031; 7894705; 1287659; 6988019 |
| NT08MM10068 | 12 | 12 | 0.91 | 19711169; 19641506; 19303503; 18655058; 18586033; 18220151; 18211368; 17937401; 17052711; 16569292 |
| NT08MM10197 | 8 | 8 | 0.77 | 20665687; 20586489; 20516588; 20397667; 20083495; 20007000; 20000723; 19997764; 19966004; 19481814 |
| NT08MM10204 | 14 | 14 | 0.52 | 20727737; 18669159; 18043132; 16550005; 16120307; 15937643; 12128120; 12110944; 11103787; 10690514 |
| NT08MM10242 | 19 | 19 | 0.96 | 19816782; 10750896; 18613689; 18351384; 17221229; 11773048; 12026180; 9891778; 11513090; 11414615 |
| NT08MM10254 | 8 | 8 | 0.87 | 19382143; 17090920; 697766; 4690969; 11459836; 11081795; 10537203; 9611813; 9367878; 9299451 |
| NT08MM10271 | 15 | 15 | 0.91 | 20847010; 20047956; 19729089; 19307717; 10396600; 8808949; 11296236; 10860755; 10679470; 17194626 |
| NT08MM10292 | 15 | 17 | 0.22 | 18723027; 18599837; 11152613; 17526841; 7642486; 15276828; 11676534 |
| NT08MM10359 | 6 | 6 | 0.95 | 20716951; 20691096; 20606008; 20599730; 20511501; 20419059; 20331425; 20204869; 20184512; 20180846 |
| NT08MM10364 | 5 | 8 | 0.87 | 16331965; 15063311; 10860732; 8695637; 8001563; 7104302; 6285956 |
| NT08MM10412 | 12 | 12 | 0.55 | 20633281; 20525894; 20455262; 20408571; 20380418; 20176951; 20176902; 20097854; 20065028; 19946141 |
| NT08MM10501 | 12 | 12 | 0.98 | 20456877; 19948868; 10522227; 18598777; 10791781; 16099524; 11772634; 15355531; 10571076; 10493130 |
| NT08MM10526 | 4 | 8 | 0.30 | 20829884; 20827334; 20796224; 20729758; 20725060; 20713718; 20627125; 20586857; 20595071; 20538062 |
| NT08MM10728 | 4 | 4 | 0.87 | 20803137; 20420917; 20160049; 19951364; 19937156; 19673409; 19549172; 19403777; 19400840; 19400781 |
| NT08MM10902 | 18 | 18 | 0.97 | 11050157; 10572136; 15060731; 11401703; 11341969; 2668876; 2664762; 1738314; 2821267 |
| NT08MM11136 | 3 | 4 | 0.21 | 20233308; 12950929; 2558955; 8150261; 2987994 |
| NT08MM11181 | 18 | 12 | 0.30 | 20844238; 20840337; 20837483; 20827732; 20824169; 20815953; 20718734; 20617898; 20610401; 20580919 |
| NT08MM11336 | 6 | 6 | 0.48 | 20724226; 20733069; 20621980; 20472798; 20377178; 20152136; 20014805; 20010803; 19936058; 19843230 |
| NT08MM11369 | 18 | 8 | 0.45 | 20666406; 20666399; 20599753; 20599728; 20595580; 20533838; 20515113; 20494124; 20442397; 20417642 |
| NT08MM11410 | 4 | 4 | 0.89 | 20585511; 19804784; 19636618; 17725563; 17408365; 18833010; 18789328; 17725563; 17106680; 9878422 |
| NT08MM11434 | 8 | 8 | 0.52 | 19189973; 15736965; 15175326; 15102833; 12600982; 10497226; 8797851; 1899572 |
| NT08MM11515 | 18 | 18 | 0.93 | 19911131; 16286358; 8412700; 9720051; 9133319; 3279024; 2674131; 1137083 |
| NT08MM11543 | 15 | 15 | 0.67 | 10606655; 17374605; 5551392; 15819620; 10973967; 6323997; 9409772 |
| NT08MM11593 | 8 | 8 | 0.88 | 20832504; 20816746; 20589823; 20576606; 20524621; 20372030; 20221546; 20186410; 20179327; 20112454 |
| NT08MM11597 | 9 | 9 | 0.85 | 20221630; 19595597; 19407376; 19185501; 18381272; 14523010; 12624762; 12549938; 11286890; 7991698 |
| NT08MM11612 | 15 | 15 | 0.98 | 20679390; 20547753; 20544860; 20498758; 20460526; 20416298; 20023039; 19945985; 19895397; 19852844 |
| NT08MM11713 | 8 | 8 | 0.82 | 20847424; 20847004; 20844983; 20843701; 20843149; 20837563; 20836522; 20833539; 20832471; 20829305 |
| NT08MM11739 | 8 | 8 | 0.31 | 18174132; 17706591; 12421312; 10841975; 12427766; 11744735; 9693743 |
| NT08MM11840 | 19 | 19 | 0.97 | 20822948; 20807862; 20618907; 20616083; 20485560; 20447272; 20444951; 20427511; 20412465; 20409281 |
| NT08MM11912 | 18 | 18 | 0.98 | 20813428; 20803137; 20705129; 20704181; 20662775; 20639324; 20566858; 20463007; 20454684; 20412049 |
| NT08MM11952 | 19 | 6 | 0.39 | 14724630; 12126625; 1817253; 8765712; 7674303; 2968919 |
| NT08MM12150 | 6 | 6 | 0.79 | 8844145; 19291873; 2265620; 8016081; 9025093; 1924375; 7040983; 767322; 7183679; 6247993 |
| NT08MM12270 | 6 | 8 | 0.26 | 20848662; 20848659; 20848643; 20848628; 20848586; 20848535; 20848441; 20848437; 20848308; 20848296 |
| NT08MM12303 | 6 | 6 | 0.45 | 19783470; 19720053; 19175412; 19072642; 15087506; 9580708; 15073316; 6421231; 16279806; 15342609 |
| NT08MM12318 | 8 | 8 | 0.91 | 20124719; 20089767; 19860829; 19084582; 18581728; 18549703; 17993624; 18062242; 17981801; 17510459 |
| NT08MM12375 | 4 | 4 | 0.76 | 20815357; 20708040; 20707000; 20607764; 20558276; 20543141; 20513630; 20226040; 20127362; 20074058 |
| NT08MM12458 | 11 | 6 | 0.62 | 19425495; 16649659; 15554191; 12669426; 7496527 |
| NT08MM12498 | 4 | 8 | 0.54 | 20387042; 20385411; 20039613; 19834746; 19827773; 19760485; 19735113; 19687346; 19679115; 19663684 |
| NT08MM12640 | 4 | 4 | 0.90 | 20346719; 20203055; 20178785; 19520101; 19361426; 19358329; 18302792; 19202091; 10667798; 15914076 |
| NT08MM12667 | 8 | 8 | 0.97 | 20541114; 19575527; 19393218; 19382345; 18824464; 18676644; 18341582; 17538658; 10570802; 9671722 |
| NT08MM12699 | 15 | 15 | 0.46 | 20705235; 20525830; 20426835; 20363938; 20152180; 19897656; 19783630; 19766635; 19747489; 19542275 |
| NT08MM12776 | 9 | 9 | 0.81 | 20110695; 18760846; 18640292; 18485779; 18031367; 17997341; 17719200; 17199921; 17116638; 17089889 |
| NT08MM12852 | 19 | 19 | 0.96 | 19533311; 11535801; 17159214; 16828055; 4018385; 16307283; 15721281; 11106394; 14660638; 12963347 |
| NT08MM12892 | 19 | 19 | 0.94 | 20639341; 20547868; 20547742; 20541116; 20519506; 20507232; 20498265; 20483773; 20454607; 20398413 |
| NT08MM12903 | 12 | 12 | 0.96 | 20847219; 20813965; 20807195; 20800791; 20709850; 20686566; 20638688; 20637599; 20622013; 20576301 |
| NT08MM12998 | 9 | 9 | 0.53 | 19788655; 19267463; 19107395; 19103481; 18422622; 18340545; 17170116; 15170232; 12324347; 12213937 |
| NT08MM13055 | 12 | 12 | 0.77 | 20837023; 20817268; 20815828; 20800597; 20799349; 20718420; 20713106; 20709893; 20707600; 20704569 |
| NT08MM13072 | 19 | 19 | 0.53 | 12531907; 9575203; 9268317; 6370994; 8366125; 1323052; 3027079 |
| NT08MM13165 | 15 | 18 | 0.52 | 20644138; 20571957; 20562310; 20530967; 20437149; 20430815; 20420522; 20372024; 20237769; 20100857 |
| NT08MM13169 | 12 | 12 | 0.95 | 20838651; 20812964; 20805355; 20670659; 20660367; 20650005; 20615555; 20595064; 20592604; 20565753 |
| NT08MM13189 | 6 | 6 | 0.95 | 20146237; 20140205; 20071747; 20071371; 18931376; 19074649; 15725673; 17914173; 18944491; 18835275 |
| NT08MM13193 | 9 | 9 | 0.99 | 19854834; 17360388; 19472174; 18804030; 12221980; 10222271; 16392678; 16710404; 16441657; 16161997 |
| TERTU_0042 | 2 | 2 | 0.58 | 20194361; 11160087; 12975365; 9765565; 9767567; 8159699; 4345352 |
| TERTU_0081 | 8 | 8 | 0.93 | 20618079; 20067779; 19496430; 19138661; 19049884; 18783734; 18772325; 17936644; 16968457; 16857210 |
| TERTU_0103 | 4 | 15 | 0.33 | 19851727; 19965501; 18454128; 14681412; 10744996; 9381988 |
| TERTU_0108 | 6 | 6 | 0.66 | 20706627; 20663713; 20571955; 20503379; 20398669; 20300437; 20227423; 20122942; 20018860; 19934045 |
| TERTU_0115 | 12 | 12 | 0.96 | 20846373; 20845933; 20843543; 20843011; 20839237; 20839050; 20837714; 20837108; 20836498; 20832960 |
| TERTU_0129 | 18 | 19 | 0.53 | 19682075; 19608743; 19504047; 19478450; 19386706; 19258047; 107827; 15214846; 17270212; 17238236 |
| TERTU_0149 | 19 | 19 | 0.93 | 18224282; 17103163; 17074899; 17013558; 17005007; 16912849; 16739943; 10419961; 10387084; 7493964 |
| TERTU_0168 | 15 | 4 | 0.64 | 16928682; 12015987; 12948827; 11525919; 2223758; 9073570; 8797819; 8087856 |
| TERTU_0176 | 17 | 13 | 0.84 | 20360175; 20348137; 20155482; 19745807; 7482698; 10496227; 18226598; 18604630; 10937990; 18575231 |
| TERTU_0183 | 19 | 19 | 0.90 | 20739278; 20686915; 20562312; 20552260; 20039646; 20039037; 19959580; 19923748; 19884762; 19744498 |
| TERTU_0264 | 12 | 12 | 0.77 | 15044551; 12051826; 11698405; 8025348; 3488815 |
| TERTU_0323 | 15 | 15 | 0.30 | 20623571; 20158269; 19924301; 18767159; 17950490; 1482126; 17698249; 17617795; 10828056; 17323948 |
| TERTU_0342 | 8 | 8 | 0.35 | 20419722; 19690371; 17900149; 17477549; 15180983; 12667082; 12631275; 12237219; 12089147; 12048188 |
| TERTU_0353 | 19 | 19 | 0.96 | 20686915; 20046832; 19935670; 19608743; 19060394; 18998121; 18824139; 18806757; 18796009; 18755688 |
| TERTU_0369 | 18 | 18 | 0.90 | 19841275; 7716527; 16735753; 18600791; 18163621; 12629550; 17347852; 16943493; 16777221; 11751241 |
| TERTU_0436 | 18 | 18 | 0.99 | 20843810; 20561529; 20230813; 20213545; 19919002; 19785523; 19734145; 19691360; 19638364; 19230850 |
| TERTU_0486 | 4 | 15 | 0.25 | 20844759; 20727793; 20720110; 20713515; 20708580; 20701076; 20691900; 20684236; 20683035; 20679435 |
| TERTU_0517 | 19 | 12 | 0.46 | 20228279; 16096273; 2394317; 14739293; 11867639; 11414739; 2522930 |
| TERTU_0570 | 17 | 6 | 0.44 | 20823118; 20655920; 20582318; 20561953; 20512989; 20484300; 20482591; 20399225; 19789459; 20385966 |
| TERTU_0607 | 19 | 19 | 0.93 | 20686915; 20609364; 19966417; 19923748; 19683032; 19646996; 19639231; 19122660; 19520709; 19508204 |
| TERTU_0621 | 13 | 9 | 0.73 | 20599697; 20308079; 20186809; 20111596; 19920185; 19880178; 19834068; 19756806; 19737935; 19631310 |
| TERTU_0656 | 15 | 6 | 0.54 | 20827598; 20622062; 20553494; 20477875; 20302647; 20153530; 20012992; 19836336; 19762509; 19744932 |
| TERTU_0676 | 19 | 19 | 0.90 | 20686915; 20682344; 20622875; 20552260; 20444687; 20227065; 20138890; 20096384; 20081828; 20070105 |
| TERTU_0729 | 14 | 6 | 0.83 | 20484015; 20305636; 20181740; 20102606; 19943803; 19916558; 19901957; 19884123; 19728742; 19639316 |
| TERTU_0738 | 4 | 15 | 0.55 | 20363938; 18282004; 10708670; 39891; 15100255; 14763974; 14707129; 12421313; 11929534; 11134414 |
| TERTU_0798 | 4 | 4 | 0.49 | 20842533; 20840079; 20839801; 20829884; 20828386; 20827334; 20826812; 20818499; 20807727; 20806971 |
| TERTU_0806 | 4 | 18 | 0.93 | 20463878; 20188576; 20103563; 20003133; 19933359; 19373193; 19324881; 19252335; 19816142; 19100867 |
| TERTU_0811 | 19 | 19 | 0.43 | 19923748; 19682075; 19506077; 19306878; 19201751; 19133500; 18796009; 107827; 18237743; 11535801 |
| TERTU_0867 | 2 | 2 | 0.93 | 20576686; 14679236; 18186650; 10531521; 16905099; 226119 |
| TERTU_0899 | 8 | 8 | 0.41 | 15358357; 3083215; 3929016; 6755169; 6118271; 776934; 4850204; 14168690; 6993236 |
| TERTU_0900 | 8 | 8 | 0.82 | 12087099; 3519582; 9490067; 9030266; 8917463; 7499307; 7663384; 8241179; 8100227; 3426633 |
| TERTU_0947 | 9 | 9 | 0.99 | 19854834; 17360388; 19472174; 18804030; 12221980; 10222271; 16392678; 16710404; 16441657; 16161997 |
| TERTU_1027 | 12 | 12 | 0.99 | 19913481; 19241474; 17074076; 17241197; 16923819; 16805830; 15866516; 15772085; 15544571; 12888578 |
| TERTU_1043 | 3 | 3 | 0.99 | 20714501; 20584910; 20466766; 20050159; 19891507; 19799915; 19630233; 19508558; 19103276; 18083807 |
| TERTU_1088 | 18 | 18 | 0.33 | 20398235; 20375110; 20088899; 20041953; 19766561; 19766570; 19734434; 19732351; 19689950; 18266918 |
| TERTU_1091 | 17 | 17 | 0.73 | 19906695; 19863111; 19630750; 19627501; 19617368; 19458082; 19361424; 19215773; 19161842; 19004832 |
| TERTU_1112 | 18 | 18 | 0.83 | 20847002; 20825484; 20816983; 20815083; 20799927; 20799348; 20797400; 20739286; 20731403; 20720015 |
| TERTU_1115 | 19 | 19 | 0.85 | 20811938; 20519512; 20118281; 20134466; 20005985; 19923747; 19807868; 19807181; 10708363; 19684178 |
| TERTU_1132 | 1 | 1 | 0.80 | 20693595; 19968637; 19768502; 19703537; 19502657; 19400493; 19196710; 18831335; 18761051; 10701688 |
| TERTU_1201 | 15 | 15 | 0.87 | 20837582; 20459395; 20303927; 20233795; 20123786; 20110267; 19920112; 19770273; 19747167; 19741198 |
| TERTU_1204 | 4 | 4 | 0.86 | 18697747; 18081840; 10708670; 16171380; 15909991; 15387825; 15175278; 14617139; 11590001; 9209056 |
| TERTU_1205 | 15 | 17 | 0.93 | 20398205; 20154128; 19700407; 18587410; 18375553; 17766240; 16077134; 16701502; 16319496; 8675034 |
| TERTU_1221 | 4 | 4 | 0.64 | 19324687; 6257658; 10466731; 15186426; 11243808; 14744139; 12368460; 12119291; 11243808; 10760170 |
| TERTU_1225 | 4 | 4 | 0.86 | 19376867; 11528005; 17238927; 8943245; 10972838; 10225855; 2407720; 10712687; 8849449; 3536871 |
| TERTU_1282 | 8 | 8 | 0.81 | 20828554; 20828325; 20724146; 20676633; 20560783; 20535465; 20518787; 20507888; 20477771; 20472739 |
| TERTU_1314 | 4 | 4 | 0.85 | 20534509; 20097760; 19903545; 19717585; 16377617; 12904785; 19019140; 18778283; 12366401; 15731070 |
| TERTU_1343 | 4 | 4 | 0.83 | 15170402; 17908686; 18774298; 17908686; 11279165; 16469702; 15546616; 10500847; 15100991; 14993307 |
| TERTU_1352 | 4 | 4 | 0.85 | 18753783; 15817382; 10940035; 6327646; 10361710; 9286988; 3492489; 2002011; 1551848; 2982790 |
| TERTU_1363 | 17 | 17 | 0.95 | 19969540; 17947550; 12384591; 15057484; 2199327; 12044670; 8604139; 1323536; 1748284; 9168617 |
| TERTU_1371 | 4 | 18 | 0.40 | 20615634; 20487285; 20459678; 20309544; 20217167; 20107991; 20099411; 20059692; 20027221; 19788805 |
| TERTU_1376 | 4 | 12 | 0.22 | 20830571; 20818486; 20732953; 20732466; 20729323; 20691396; 20667000; 20666990; 20647113; 20622123 |
| TERTU_1401 | 19 | 19 | 0.96 | 18499663; 18210176; 15552059; 15226299; 15165190; 14646108; 12686644; 11937332; 11846551; 10610795 |
| TERTU_1402 | 19 | 19 | 0.57 | 20816060; 20513249; 20382766; 20309546; 20220136; 20008114; 19850926; 19700415; 19514945; 19507629 |
| TERTU_1469 | 12 | 12 | 0.72 | 14999401; 11477070; 12939276; 12914946; 2184035 |
| TERTU_1475 | 4 | 8 | 0.84 | 19172265; 15294836; 17514677; 3243438; 9683494; 10438748; 12670689; 10589735; 2231712; 9634695 |
| TERTU_1518 | 5 | 5 | 0.78 | 20797998; 20221733; 20221547; 20121157; 20053297; 19967421; 19863729; 19805110; 19663502; 19514721 |
| TERTU_1562 | 5 | 8 | 0.78 | 20723231; 20562311; 19936986; 19904831; 19827797; 19648251; 19286809; 18355323; 10618234; 17407181 |
| TERTU_1594 | 5 | 5 | 0.69 | 15485884; 9514861; 1631147; 2615765; 2643516; 3062178; 2826155 |
| TERTU_1615 | 12 | 12 | 0.76 | 20799977; 20610410; 20433838; 20158612; 19535917; 19238259; 19101567; 18524928; 20076671; 9523453 |
| TERTU_1626 | 12 | 12 | 0.97 | 20828137; 20826753; 20825413; 20818167; 20812737; 20811381; 20801545; 20707733; 20696895; 20695487 |
| TERTU_1636 | 14 | 8 | 0.55 | 20647036; 20516613; 20462199; 20448188; 19899807; 19632469; 19577540; 19082475; 19061340; 19046875 |
| TERTU_1664 | 4 | 18 | 0.30 | 20054881; 19395482; 15276320; 18524912; 18289922; 11062129 |
| TERTU_1673 | 18 | 18 | 0.99 | 20495348; 20387456; 20116460; 20081036; 19663511; 19307713; 19295548; 19270115; 19202098; 19199920 |
| TERTU_1674 | 18 | 19 | 0.52 | 20709893; 20562284; 20496884; 20372022; 20159017; 19830421; 19597812; 19478450; 19459957; 19258047 |
| TERTU_1729 | 6 | 6 | 0.89 | 20410261; 20308066; 20200253; 20146237; 20122942; 20097223; 20071747; 20054126; 20023031; 20015968 |
| TERTU_1744 | 5 | 5 | 0.53 | 19744922; 16328785; 16289027; 11569804; 12070175; 8636094; 1848010 |
| TERTU_1878 | 8 | 8 | 0.82 | 20466730; 20050916; 18687332; 18174132; 17623665; 12421312; 16920107; 10841975; 11744735; 9914305 |
| TERTU_1900 | 18 | 19 | 0.45 | 20686915; 20227408; 20173060; 19520709; 19478450; 19349750; 19258047; 17922847; 11150609; 17238236 |
| TERTU_1906 | 12 | 8 | 0.46 | 20707389; 20384326; 20352460; 19662334; 19459971; 15754057; 14741712; 12135760; 11061233 |
| TERTU_1916 | 15 | 4 | 0.25 | 19238258; 10811905; 9379903; 15287751; 10805562; 12122010; 11069676; 9370259; 236308; 7984109 |
| TERTU_1926 | 2 | 2 | 0.42 | 18801628; 2204419; 16171773; 16123074; 16100107; 15349715; 14702393; 11820920; 12646567; 11282657 |
| TERTU_1931 | 4 | 4 | 0.76 | 20439729; 20355710; 19966007; 19923210; 19705835; 19646451; 19581362; 19502407; 15170402; 19256549 |
| TERTU_1935 | 4 | 8 | 0.76 | 20669954; 20593814; 20546754; 20419722; 20406405; 20387782; 20359206; 20353187; 20348090; 20334431 |
| TERTU_1946 | 4 | 6 | 0.39 | 20713134; 20623179; 20599698; 20596676; 20132728; 18467468; 19258348; 19238258; 18978732; 18656956 |
| TERTU_1950 | 18 | 8 | 0.63 | 20214873; 19155212; 19082940; 18943005; 18817763; 18486594; 17899367; 17229387; 17114648; 16973619 |
| TERTU_1951 | 18 | 8 | 0.94 | 19259137; 19155212; 19060309; 19006332; 18817763; 9593879; 18486594; 18455500; 17899367; 17894548 |
| TERTU_1957 | 8 | 8 | 0.50 | 20600382; 20299454; 19725101; 19446033; 18972197; 17524345; 17453174; 16814740; 16599556; 16080701 |
| TERTU_1961 | 4 | 4 | 0.43 | 20607520; 9379903; 11298285; 9484881; 8973341; 8022259 |
| TERTU_1967 | 12 | 13 | 0.53 | 19440203; 18266307; 3049606; 17216634; 10368282; 16977663; 9407082; 8798470; 7657641; 6387371 |
| TERTU_1990 | 4 | 4 | 0.24 | 20696392; 20666435; 20589823; 20588302; 20444870; 20348291; 20203700; 20154113; 19651421; 19619887 |
| TERTU_1994 | 4 | 9 | 0.91 | 20176020; 20057061; 19095065; 19052370; 18565285; 11373295; 17604051; 16413022; 8564546; 16699188 |
| TERTU_2014 | 19 | 19 | 0.48 | 20498856; 20228279; 20097759; 19509290; 19351539; 19304844; 19179337; 18996339; 18716755; 18678939 |
| TERTU_2039 | 14 | 14 | 0.84 | 19219570; 18596164; 17893650; 17635220; 15479670; 15059910; 14614079; 14522901; 14517346; 12419845 |
| TERTU_2142 | 12 | 12 | 0.98 | 19889088; 10966643; 15047720; 14760686; 14637004; 12177052; 11351281; 11172723; 10715137; 9383148 |
| TERTU_2171 | 5 | 8 | 0.30 | 20604542; 20453147; 20415463; 20397966; 20375234; 20299628; 20193759; 20160052; 20144715; 20110259 |
| TERTU_2206 | 4 | 9 | 0.40 | 20659683; 20534346; 20432424; 20430898; 20332208; 20304931; 20148945; 20067617; 19860832; 19801377 |
| TERTU_2237 | 12 | 18 | 0.48 | 20841380; 20823220; 20810540; 20797862; 20739542; 20736485; 20736288; 20727848; 20712629; 20698859 |
| TERTU_2248 | 18 | 18 | 0.64 | 19682536; 18355966; 17632081; 17628207; 17555702; 17494632; 17002596; 16541252; 11790133; 16129486 |
| TERTU_2254 | 9 | 9 | 0.99 | 20431990; 20204203; 20131019; 20086206; 19797200; 19762802; 19760663; 19473107; 19193980; 18791145 |
| TERTU_2293 | 4 | 6 | 0.98 | 20837538; 20810656; 20810653; 20798359; 20735989; 20729138; 20716525; 20705645; 20703330; 20689233 |
| TERTU_2371 | 4 | 8 | 0.56 | 20842310; 20690630; 20529840; 20471485; 20460582; 20359485; 20359206; 20353187; 20340105; 20163155 |
| TERTU_2372 | 4 | 8 | 0.83 | 20447827; 16342964; 12726929; 11945122; 11559360; 11124904; 10848975; 10385636; 9278141; 9211271 |
| TERTU_2393 | 6 | 6 | 0.96 | 19625490; 12947199; 18931437; 3006102; 6251160; 10390354; 18164032; 10373579; 17439637; 11302791 |
| TERTU_2403 | 4 | 15 | 0.50 | 19293428; 18757857; 9210342; 1656497; 6350604; 773686; 5883923 |
| TERTU_2456 | 4 | 4 | 0.46 | 20660083; 20507997; 20472940; 20395279; 19930663; 11931551; 19578062; 19368887; 18292935; 18087702 |
| TERTU_2465 | 18 | 8 | 0.24 | 20818890; 20732973; 20729930; 20720104; 20718717; 20708580; 20702717; 20701077; 20700687; 20691835 |
| TERTU_2477 | 12 | 11 | 0.32 | 18647502; 18335753; 18320592; 18082675; 17332923; 16009011; 15147195; 10612659; 14630798; 12548635 |
| TERTU_2478 | 2 | 2 | 0.49 | 18256491; 15452112; 12686115; 12269807; 12206776; 11286891; 6388637; 6996720; 6773563 |
| TERTU_2529 | 8 | 8 | 0.57 | 20837639; 20804614; 20817117; 20807773; 20807714; 20802119; 20802081; 20801909; 20738517; 20721682 |
| TERTU_2540 | 18 | 12 | 0.42 | 20519396; 17943122; 18093808; 15026344; 7797266 |
| TERTU_2553 | 8 | 8 | 0.83 | 20847215; 20846527; 20844025; 20124521; 20357281; 20357281; 20839223; 20838623; 20836540; 20835842 |
| TERTU_2560 | 17 | 17 | 0.72 | 20399182; 19690099; 19011043; 16946718; 16216978; 16157481; 15152027; 15037810; 12013525; 1756730 |
| TERTU_2598 | 13 | 13 | 0.99 | 20184321; 19400805; 18789337; 18786544; 18032607; 16857584; 18094627; 17932046; 15472854; 10829287 |
| TERTU_2600 | 8 | 8 | 0.42 | 20026409; 16963438; 15978585; 12600198; 11245795; 2204423 |
| TERTU_2633 | 13 | 13 | 0.91 | 20360392; 20228783; 20132829; 20128644; 20104869; 20810525; 19682871; 19644880; 19549788; 19505149 |
| TERTU_2637 | 14 | 14 | 0.89 | 20821213; 20801890; 20738708; 20713695; 20711856; 20684612; 20649941; 20622504; 20606293; 20602252 |
| TERTU_2684 | 18 | 19 | 0.53 | 20414771; 20173060; 20172492; 19710471; 20076727; 18553392; 16957894; 17985078; 3596237; 17322304 |
| TERTU_2703 | 19 | 8 | 0.60 | 20567601; 20498856; 20382127; 20227362; 20179248; 20558376; 20127462; 20117116; 20097759; 20082315 |
| TERTU_2717 | 12 | 12 | 0.37 | 19498284; 18831998; 18713752; 18028193; 17896114; 17682820; 17513741; 11373684; 17136388; 17107951 |
| TERTU_2725 | 2 | 2 | 0.88 | 18973344; 18811645; 18762421; 12615349; 4926602; 6127606; 8626063; 1663748; 2209599; 3516220 |
| TERTU_2729 | 2 | 1 | 0.51 | 20807338; 20705362; 20532401; 20487026; 20359225; 20170126; 20079748; 19996170; 19816125; 18602859 |
| TERTU_2767 | 19 | 19 | 0.90 | 20140934; 20179984; 19384899; 10575553; 18700763; 18599029; 18596046; 17350598; 18330718; 18206726 |
| TERTU_2771 | 4 | 4 | 0.88 | 20585511; 19804784; 19636618; 17725563; 17408365; 18833010; 18789328; 10675323; 17997135; 17725563 |
| TERTU_2811 | 13 | 13 | 0.99 | 20557293; 20179335; 20045102; 20010690; 19903477; 19702327; 19668194; 19661429; 19549823; 19497723 |
| TERTU_2835 | 2 | 2 | 0.90 | 20627615; 19728152; 9026; 16528563; 11389720 |
| TERTU_2865 | 4 | 2 | 0.23 | 20696925; 20577996; 20516619; 20368332; 20359185; 20225828; 20399179; 20121951; 19923722; 19923209 |
| TERTU_2866 | 4 | 19 | 0.35 | 20846931; 20682343; 20667976; 20656688; 20628353; 20615874; 20605911; 20594961; 20540760; 20525860 |
| TERTU_2871 | 8 | 1 | 0.93 | 18481057; 16960959; 15526543; 12670956; 7491952; 11032894; 9565331; 8933102; 8832974; 7813517 |
| TERTU_2873 | 9 | 8 | 0.40 | 19066401; 11457035; 17535816; 17439396; 17158889; 16042397; 15173618; 11110912; 12379131; 10580280 |
| TERTU_2949 | 2 | 2 | 0.97 | 19754882; 19267692; 19154787; 18603790; 17460889; 16489629; 16289918; 15983414; 15522295; 14595395 |
| TERTU_2971 | 13 | 13 | 0.99 | 19914242; 19894713; 19837083; 19772352; 19706454; 19675636; 19668194; 19596773; 19502729; 19478446 |
| TERTU_2987 | 8 | 2 | 0.81 | 19864628; 19647806; 19554628; 19355893; 19270703; 19249205; 18508649; 18344983; 18178143; 17642516 |
| TERTU_3025 | 18 | 18 | 0.75 | 20713660; 20385866; 19749294; 19617367; 19151142; 18619818; 12655047; 1091862; 6785365; 18042252 |
| TERTU_3029 | 12 | 12 | 0.43 | 20799349; 20599573; 20593478; 20564558; 20537141; 20486678; 20415450; 20375062; 20232307; 20219826 |
| TERTU_3030 | 2 | 14 | 0.95 | 20206212; 20047307; 20047306; 19399913; 6268428; 19165623; 19138656; 18321072; 18157947; 17868694 |
| TERTU_3031 | 3 | 3 | 0.49 | 20545853; 20530728; 20361050; 20233397; 20118249; 19889099; 19486641; 11703657; 12065472; 10537190 |
| TERTU_3046 | 4 | 4 | 0.93 | 20460179; 20363951; 20345660; 20066037; 19880599; 19880597; 19737354; 19635793; 19542290; 19376877 |
| TERTU_3051 | 19 | 19 | 0.90 | 20024979; 19403924; 19369074; 19198900; 19014883; 19007109; 18704940; 18557704; 18259126; 17507028 |
| TERTU_3055 | 19 | 19 | 0.68 | 20580675; 20395215; 20087619; 19995973; 19697870; 19542290; 19249177; 9364911; 18800621; 18786147 |
| TERTU_3065 | 18 | 18 | 0.98 | 20822503; 20598093; 20519092; 20466723; 20430762; 20424298; 20411246; 20346358; 20303857; 20188062 |
| TERTU_3080 | 9 | 9 | 0.99 | 20725044; 20616867; 20140210; 19965770; 7984417; 18557770; 17266990; 10716717; 16707089; 16276872 |
| TERTU_3085 | 6 | 15 | 0.88 | 20633229; 20395367; 20156994; 20140235; 20132305; 20118249; 20039181; 19825646; 19633076; 19560425 |
| TERTU_3139 | 8 | 17 | 0.52 | 20822113; 20731347; 20720002; 20718947; 20696927; 20672157; 20655755; 20518555; 20518483; 20390103 |
| TERTU_3170 | 3 | 4 | 0.24 | 19486161; 16987180; 12453228; 12410832; 10026202; 10051592; 10200956; 9224886 |
| TERTU_3172 | 3 | 3 | 0.99 | 20616068; 20472801; 20455262; 20439474; 20348252; 20080557; 20070257; 19857646; 19646999; 19486161 |
| TERTU_3238 | 12 | 6 | 0.74 | 19798519; 9145897; 16437107; 8804555; 12897787; 12853139; 12834867; 10783239; 8050351 |
| TERTU_3240 | 9 | 8 | 0.37 | 20421508; 19592593; 19343258; 19207562; 19142932; 19128318; 19116691; 19037261; 18670176; 18247576 |
| TERTU_3253 | 19 | 19 | 0.77 | 19813101; 18981178; 17340109; 17074760; 16133657; 15489502; 15278289; 14659747; 9537320; 12738774 |
| TERTU_3266 | 13 | 13 | 0.98 | 20844073; 20726009; 20679220; 20679238; 20679238; 20675580; 20625112; 20620951; 20670883; 20593023 |
| TERTU_3286 | 3 | 18 | 0.82 | 20847002; 20846957; 20844758; 20844462; 20843996; 20843347; 20832899; 20832396; 20831471; 20831404 |
| TERTU_3341 | 19 | 8 | 0.32 | 20699582; 20691225; 20680577; 20680564; 20598292; 20416864; 19850285; 19795066; 19699401; 19691080 |
| TERTU_3347 | 19 | 19 | 0.45 | 19351587; 5031119; 107827; 15214846; 11053376; 11535801; 17922847; 17340109; 17225100; 17103163 |
| TERTU_3356 | 19 | 19 | 0.94 | 20467237; 19846561; 19601664; 19193638; 18718911; 18653706; 18434414; 17947240; 16697989; 7972516 |
| TERTU_3375 | 13 | 13 | 0.42 | 20824822; 20709901; 20644968; 20561953; 20525238; 20418380; 20410300; 20360394; 20202168; 20122156 |
| TERTU_3379 | 1 | 15 | 0.63 | 20811983; 20810664; 20736484; 20720110; 20705954; 20685879; 20681595; 20677833; 20659428; 20655927 |
| TERTU_3439 | 15 | 15 | 0.85 | 20639578; 20600859; 20532736; 20532735; 20521335; 20493877; 20479260; 20385843; 20363937; 20304991 |
| TERTU_3485 | 5 | 8 | 0.40 | 15796980; 15090490; 12640627; 12559615; 9247670; 8697577; 1569960; 1802034; 3896120; 3916727 |
| TERTU_3572 | 15 | 15 | 0.61 | 20622061; 20231439; 19695263; 19416883; 19170727; 18485075; 17241199; 17153922; 17020586; 17008065 |
| TERTU_3575 | 1 | 8 | 0.45 | 20559153; 19481951; 18537471; 18528747; 18354250; 17189831; 16571593; 15367131; 12494465; 12470673 |
| TERTU_3576 | 1 | 8 | 0.46 | 20630732; 20592025; 20201932; 19943898; 19555410; 19344330; 18945221; 13129625; 16820760; 15912497 |
| TERTU_3605 | 19 | 19 | 0.76 | 20609364; 19682075; 19046944; 17602905; 18806757; 18457422; 17503147; 17287210; 17139091; 8797842 |
| TERTU_3657 | 11 | 11 | 0.99 | 20197135; 17028591; 15268934; 10545172; 11877443; 10985748; 7830551; 1660923; 10574452; 10207011 |
| TERTU_3683 | 12 | 12 | 0.98 | 18479146; 18375803; 10383971; 10966643; 11115120; 1645840; 9854064; 11463916; 9383148; 10832972 |
| TERTU_3699 | 2 | 2 | 0.92 | 19888457; 13990617; 12376536; 16497163; 11104108; 338917; 8522961; 8619301; 7982968; 2542220 |
| TERTU_3768 | 12 | 12 | 0.99 | 20833209; 20826445; 20348419; 20306218; 19804410; 19696631; 19553544; 19417147; 11598093; 19349421 |
| TERTU_3781 | 8 | 8 | 0.87 | 20652669; 20025846; 19862563; 19843523; 19736979; 19616011; 19616009; 19464996; 19325183; 19234303 |
| TERTU_3787 | 6 | 6 | 0.89 | 20208147; 20139184; 20080107; 19948952; 19482923; 11889127; 19341290; 19007414; 15199127; 16407906 |
| TERTU_3796 | 2 | 2 | 0.86 | 19878958; 17962451; 19392660; 15597200; 15356000 |
| TERTU_3823 | 15 | 15 | 0.95 | 20738805; 20716276; 20675572; 20643148; 20639578; 20639319; 20618761; 20600859; 20578795; 20573193 |
| TERTU_3853 | 13 | 13 | 0.99 | 20197408; 20234387; 15980571; 12716898; 18391411; 18342886; 18247350; 1470174; 17619020; 17516842 |
| TERTU_3889 | 18 | 18 | 0.39 | 20481466; 20154644; 20060739; 19761223; 17401378; 18227070; 17355973; 16467350; 16364320; 16216272 |
| TERTU_3903 | 18 | 4 | 0.40 | 20825450; 20452626; 15800628; 17379715; 19082748; 19027126; 18666543; 11482991; 18023847; 16527314 |
| TERTU_3928 | 17 | 15 | 0.50 | 20639338; 19940143; 16332890; 19700528; 9647800; 14652736; 19255944; 19167516; 18988696; 18639631 |
| TERTU_3962 | 1 | 1 | 0.96 | 20445230; 20065117; 19923721; 19858196; 19568767; 19405093; 19350404; 19253050; 19157014; 18379776 |
| TERTU_4004 | 8 | 8 | 0.85 | 20813101; 20534481; 20525825; 20511503; 20466976; 20370610; 20335826; 20185795; 20178638; 20064527 |
| TERTU_4039 | 4 | 9 | 0.98 | 20304657; 20221630; 19968566; 19884012; 19863661; 19836235; 19665020; 19664929; 19595597; 19407376 |
| TERTU_4080 | 9 | 9 | 0.36 | 20226373; 19455508; 19286472; 19143636; 18760322; 11316811; 18422860; 18042941; 17233594; 1538787 |
| TERTU_4087 | 18 | 18 | 0.95 | 20601471; 20102440; 19911130; 19451245; 19144921; 11872840; 10087167; 18086184; 10383757; 10522227 |
| TERTU_4090 | 19 | 19 | 0.72 | 19129644; 18579426; 18239434; 8979350; 7493964; 2115772; 8375377 |
| TERTU_4097 | 18 | 18 | 0.87 | 20100283; 20010822; 19631523; 12522116; 18687947; 17931609; 16445289; 11449397 |
| TERTU_4117 | 19 | 19 | 0.97 | 20594961; 20345654; 20300605; 20199110; 20173067; 20118281; 10708363; 19627504; 19447130; 19422893 |
| TERTU_4160 | 4 | 8 | 0.53 | 18796334; 10801492; 17964868; 14645730; 16871797; 9864324; 15667296; 15100998; 14573954; 13680069 |
| TERTU_4216 | 18 | 18 | 0.97 | 20846531; 20846323; 20844750; 20844252; 20844245; 20844147; 20838602; 20836083; 20835844; 20833221 |
| TERTU_4225 | 19 | 19 | 0.94 | 20155484; 20118379; 19847382; 19836729; 19817688; 19801419; 19756576; 19702863; 19697020; 19652954 |
| TERTU_4353 | 15 | 15 | 0.86 | 1584789; 3323813; 13331868; 7476184; 7584049; 8000527; 1334233; 6321442; 3062173; 3135464 |
| TERTU_4394 | 4 | 19 | 0.23 | 20844958; 20844906; 20833807; 20830571; 20829194; 20826221; 20818486; 20816046; 20807881; 20806226 |
| TERTU_4506 | 19 | 19 | 0.79 | 20812985; 20622875; 20556855; 20444687; 20154127; 20138890; 20070105; 20054127; 20046832; 20039037 |
| TERTU_4511 | 19 | 6 | 0.34 | 20605777; 17486406; 17403048; 16859842; 15990253; 15186455; 15109730; 9673232; 9004408; 11976290 |
| TERTU_4597 | 18 | 19 | 0.69 | 20686915; 20682344; 19884762; 19504047; 19400841; 18836907; 18678939; 18049859; 11150609; 17340109 |
| TERTU_4599 | 15 | 15 | 0.69 | 20584146; 19919539; 19332833; 18294208; 16754873; 2172217; 16541134; 10559158; 15818467 |
| TERTU_4626 | 3 | 3 | 0.46 | 10633125; 10430571; 15993836; 15838638; 8955641; 10510235; 10972813; 9799225; 9623911; 1180526 |
| TERTU_4676 | 19 | 19 | 0.90 | 20686915; 20682344; 20532756; 20154127; 19935670; 19884762; 19813101; 19122660; 19508204; 19504047 |
| TERTU_4690 | 5 | 8 | 0.92 | 20689707; 20643656; 20493843; 20458400; 20444232; 20335404; 19754882; 19562269; 19415759; 19194001 |
| TERTU_4691 | 8 | 8 | 0.69 | 20643656; 20493843; 20444232; 20434234; 20025616; 19562269; 19484273; 19415759; 19285953; 19269345 |
