# Supplementary material for: Automatic Assignment of Prokaryotic Genes to Functional Categories Using Literature Profiling
Source: PLoS One. 2012 Oct 15;7(10):e47436. doi: 10.1371/journal.pone.0047436 (PMC3471813; doi:10.1371/journal.pone.0047436)
Supplement: Table S3 — Genes classified by LitProf - Gene Classifier which were previously assigned to ‘Unknown function’ and ‘Unclassified’ categories of JCVI-CMR. (DOC) [file pone.0047436.s005.doc]

GENES CLASSIFIED BY LITPROF – GENE CLASSIFIER WICH WERE PREVIOUSLY ASSIGNED TO ‘UNKNOWN FUNCTION’ AND ‘UNCLASSIFIED’ CATEGORIES OF JCVI-CMR

| **Gene** | **Category Assigned** | **Classification Confidence** | **PubMed IDs** |
| --- | --- | --- | --- |
| AAur_0162 | 19 | 0.86 | 21378199; 21369973; 21369910; 21357626; 21357429; 21324659; 21311881; 21307589; 21297160 |
| AAur_0170 | 4 | 0.75 | 20507997; 20117010; 19770041; 19695338; 19135125; 15695811; 17270289; 10940244; 1638630; 12824352 |
| AAur_0185 | 6 | 0.87 | 21402758; 21398634; 21383239; 21382109; 21372093; 21369983; 21362510; 21362508; 21354180; 21353876 |
| AAur_0248 | 8 | 0.73 | 21357486; 21322495; 21322489; 21319715; 21299212; 21277857; 21277289; 21220625; 21216999; 21209222 |
| AAur_0267 | 8 | 0.89 | 21380735; 21319715; 21216999; 21193665; 21183637; 21178073; 21105363; 21102601; 21087208; 21070747 |
| AAur_0343 | 12 | 0.80 | 20032457; 11751634; 17504491; 17855452; 10569626; 16127432; 16109378; 15249048; 15195946; 15157086 |
| AAur_0717 | 8 | 0.96 | 21336929; 21296661; 21169495; 21168410; 21132340; 21126961; 21063702; 20937090; 20818429; 20808820 |
| AAur_0789 | 8 | 0.73 | 21091201; 20514241; 19011745; 12003933; 18422649; 17350704; 15680231; 14965227; 12974644; 12388585 |
| AAur_0952 | 8 | 0.80 | 20154419; 2854198; 12368463; 16006525; 15186772; 17628145; 17046835; 10089316; 16268782; 16097891 |
| AAur_1184 | 6 | 0.91 | 21248859; 21241792; 21193392; 20800503; 17126598; 19747545; 12517334; 12626685; 10986228; 17029241 |
| AAur_1577 | 2 | 0.83 | 20861021; 10829079; 16830073; 8631762; 11283592; 12605683; 12480890; 10622725; 9615466; 1324835 |
| AAur_1601 | 6 | 0.83 | 17704061; 10647931; 17449473; 10319867; 16973605; 16955407; 10559976; 16425234; 16419127; 16045469 |
| AAur_1922 | 8 | 0.81 | 21075841; 20944229; 20643958; 20558746; 20405215; 19908864; 17850165; 18957412; 19102705; 17355973 |
| AAur_2239 | 13 | 0.88 | 21330151; 19423627; 19803466; 11054294; 17110332; 16584863; 15186354; 11489118; 8757728; 7596295 |
| AAur_2312 | 8 | 0.83 | 21216999; 21070747; 20580617; 20540056; 20413550; 20370825; 20236932; 20212162; 20206639; 20178464 |
| AAur_2535 | 19 | 0.82 | 5160755; 20601509; 20156187; 19923723; 19840758; 18345354; 19574167; 18067544; 19149682; 19081844 |
| AAur_2633 | 18 | 0.78 | 20581474; 20038140; 7860587; 14521953; 17562318; 16275737; 11592120; 10493532; 2404009; 9614122 |
| AAur_2777 | 18 | 0.97 | 19711106; 15358235; 19027009; 19008001; 12455987; 17687501; 18330469; 16930316; 12960109; 11771758 |
| AAur_3084 | 12 | 0.93 | 21241743; 20944219; 20650005; 19139197; 19460310; 19900580; 10913103; 19523070; 18602403; 18593905 |
| AAur_3101 | 8 | 0.80 | 18757814; 17669423; 10091591; 10025942; 8448176; 1633822; 6310323 |
| AAur_3370 | 12 | 0.78 | 21348431; 21072166; 20980587; 20798394; 20798164; 20615878; 20068039; 20040538; 19846560; 18794886 |
| AAur_3537 | 8 | 0.80 | 20934402; 19465092; 18945673; 18757814; 18216065; 18000703; 10467097; 17944363; 17669423; 16820168 |
| AAur_3674 | 19 | 0.90 | 19081844; 18352857; 16700076; 10542272; 15522073; 15053875; 12584002; 11738085; 11287126 |
| AAur_3738 | 18 | 0.78 | 21357396; 21336990; 21315718; 21315686; 21311724; 21221131; 21177413; 21168419; 21148206; 21122131 |
| AAur_3917 | 8 | 0.87 | 1998338; 15551868; 17086172; 15551868; 2985470; 14506266; 9722945; 9417084; 1656234 |
| AAur_4029 | 8 | 0.86 | 19099183; 17654627; 17218763; 17208234; 9171435; 9070288; 2675311; 1605723; 1894599; 2007581 |
| AAur_pTC10040 | 6 | 0.89 | 21354425; 21239581; 20595381; 20233304; 20132442; 19961412; 2540407; 19040634; 12415300; 18957591 |
| AAur_pTC10081 | 18 | 0.98 | 17302441; 20004175; 19858398; 19561086; 15189142; 19362642; 19359404; 19286470; 19250938; 16665774 |
| AAur_pTC10141 | 12 | 0.93 | 21360624; 21329657; 21241743; 21193412; 21148378; 21078856; 21041443; 20952387; 20944219; 20856818 |
| AAur_pTC20107 | 8 | 0.76 | 21296885; 21281627; 21081498; 20944230; 20838375; 20577996; 20577142; 20558438; 6100313; 20364820 |
| AAur_pTC20237 | 15 | 0.99 | 21403465; 21402777; 21402151; 21401918; 21401619; 21399626; 21398614; 21398611; 21398607; 21398516 |
| AAur_pTC20271 | 19 | 0.83 | 19400771; 16530188; 12765834; 12765833; 11278568; 9804788; 1645282 |
| ACIRA0001_0048 | 12 | 0.97 | 21339709; 21338355; 21305563; 21268078; 21268016; 21219955; 21193014; 21184441; 21178177; 21156051 |
| ACIRA0001_0181 | 19 | 0.70 | 19538395; 19502509; 18461320; 3335502; 16096274; 15280033; 15159628; 14684172; 14518977; 10517585 |
| ACIRA0001_0240 | 12 | 0.82 | 21266255; 21183667; 20960078; 20547872; 20482308; 20477872; 20405048; 12654915; 20184891; 20135153 |
| ACIRA0001_0283 | 19 | 0.90 | 21097635; 20497501; 20497501; 20400548; 18554861; 18490448; 8830709; 10419957; 10206711; 14617646 |
| ACIRA0001_0356 | 18 | 0.95 | 21394759; 21394603; 21394086; 21394083; 21393072; 21392824; 21392501; 21392495; 21392268; 21391832 |
| ACIRA0001_0427 | 19 | 0.93 | 21397493; 21342541; 21233160; 21126315; 21106106; 21098513; 21070855; 21040749; 20980614 |
| ACIRA0001_0453 | 18 | 0.88 | 21339705; 21385703; 21303910; 21297161; 21274617; 21245269; 21148420; 21147776; 21111787; 20843792 |
| ACIRA0001_0482 | 8 | 0.70 | 21377399; 21270770; 21253866; 21228234; 21190582; 21179234; 21166653; 20944230; 20944006; 20813101 |
| ACIRA0001_0507 | 8 | 0.78 | 21324682; 21272045; 21236692; 20528775; 19807880; 19467932; 2535522; 16740637; 18045419; 12517448 |
| ACIRA0001_0552 | 8 | 0.94 | 20601217; 20419722; 20186410; 11470912; 19692330; 19583210; 15811344; 16675288; 11702082; 8814218 |
| ACIRA0001_0601 | 19 | 0.94 | 21397493; 21342541; 21367879; 21349845; 21307941; 21297907; 21295693; 21221632; 21182826 |
| ACIRA0001_0616 | 19 | 0.74 | 21400688; 21400235; 21398593; 21393228; 21392988; 21390508; 21390257; 21388958; 21388880; 21388433 |
| ACIRA0001_0629 | 18 | 0.84 | 21400700; 21398536; 21396409; 21389053; 21385840; 21382104; 21380641; 21378973; 21378164; 21377446 |
| ACIRA0001_0681 | 8 | 0.81 | 20826164; 20639368; 19839648; 19664062; 10515912; 17373777; 17353140; 16814740; 942051; 12577265 |
| ACIRA0001_0691 | 9 | 0.95 | 21393855; 21216247; 21145452; 20956350; 20944213; 20886116; 20860408; 20729810; 20643655; 20634289 |
| ACIRA0001_0755 | 19 | 0.85 | 21270273; 21255725; 21248844; 21247091; 21243821; 21229888; 21228185; 21212461; 21207622; 21195054 |
| ACIRA0001_0804 | 2 | 0.79 | 10383756; 2113383; 10075431; 8885414; 791939; 14217462; 14438352; 1097404; 6276219 |
| ACIRA0001_0805 | 9 | 0.72 | 21393855; 21371424; 21370880; 21370215; 21279997; 21272378; 21269277; 21216247; 21195640; 21145452 |
| ACIRA0001_1077 | 19 | 0.87 | 21400235; 21388958; 21380775; 21378160; 21369973; 21369910; 21367878; 21367655; 21357626; 21357429 |
| ACIRA0001_1106 | 18 | 0.94 | 21398536; 21299248; 21279410; 21221942; 21178960; 21062372; 20981744; 20865003; 20583998; 20554571 |
| ACIRA0001_1190 | 4 | 0.71 | 20507997; 20117010; 19770041; 19695338; 19135125; 15695811; 18005663; 17270289; 10940244; 1638630 |
| ACIRA0001_1244 | 15 | 0.86 | 21393212; 21390327; 21386983; 21383239; 21378189; 21375706; 21369994; 21369825; 21364304; 21355787 |
| ACIRA0001_1323 | 12 | 0.89 | 21247409; 16854568; 18989571; 18291114; 18174901; 10436050; 16905115; 10352394; 11723117; 1531448 |
| ACIRA0001_1329 | 5 | 0.98 | 21269549; 20865198; 20863310; 20843788; 20445243; 20387539; 20382221; 20359198; 20353153; 20307537 |
| ACIRA0001_1376 | 15 | 0.80 | 9701816; 15797726; 12604154; 6759876; 10803021; 364941; 9282733; 8742358; 7766893; 1400253 |
| ACIRA0001_1494 | 18 | 0.93 | 20093292; 18611376; 17952093; 3017428; 16777062; 10464217; 16335991; 5432063; 12142471; 11081577 |
| ACIRA0001_1788 | 18 | 0.72 | 20624215; 20154147; 20083401; 19887446; 18656546; 19748349; 19619244; 19351326; 19143621; 16030141 |
| ACIRA0001_1835 | 18 | 0.87 | 21387181; 20180907; 18939857; 18754784; 18641391; 18599313; 18338226; 17964932; 17555765; 17174333 |
| ACIRA0001_1933 | 18 | 0.80 | 18446650; 10617594; 15630182; 15509761; 11595321; 11127834; 11030518; 16347571; 7893473; 8081496 |
| ACIRA0001_2042 | 9 | 0.99 | 21314018; 21211036; 21090686; 21075355; 20957036; 20924576; 20920594; 20874804; 20858216; 20850803 |
| ACIRA0001_2115 | 9 | 0.83 | 20494124; 20481475; 20463021; 20334431; 20055482; 19639238; 19470521; 19673949; 19061855; 16430210 |
| ACIRA0001_2195 | 18 | 0.99 | 21398536; 21393450; 21391724; 21350490; 21346352; 21325274; 21253903; 21249122; 21211813; 21178960 |
| ACIRA0001_2270 | 18 | 0.84 | 21354237; 21297957; 21256155; 21241361; 21229398; 21223391; 21161330; 21063387; 21036780; 20936810 |
| ACIRA0001_2546 | 11 | 0.90 | 20949106; 20875907; 20802084; 20627350; 20554775; 17138868; 20007650; 19937113; 19663564; 19572425 |
| ACIRA0001_2577 | 18 | 0.96 | 18063795; 19039701; 18321634; 17217066; 16857941; 16835234; 7592942; 16269006; 10898947; 10686131 |
| ACIRA0001_2832 | 8 | 0.87 | 20833784; 20601046; 20488895; 20487290; 20385101; 19383527; 17532339; 19147103; 18845259; 18675788 |
| ACIRA0001_3147 | 18 | 0.70 | 21207455; 21143936; 21081547; 21044875; 20881245; 20877283; 20870764; 20730247; 20656779; 20626869 |
| ACIRA0001_3164 | 18 | 0.79 | 21349974; 21295952; 21056572; 21028901; 20553812; 20382203; 20363295; 20138126; 20026386; 19719512 |
| ACIRA0001_3240 | 12 | 0.78 | 21348431; 21072166; 20980587; 20798394; 20798164; 20615878; 20068039; 20040538; 19846560; 18794886 |
| AF_0185 | 8 | 0.72 | 20297722; 19888992; 19826863; 17639348; 17506525; 18314963; 18300158; 17959596; 17893805; 17891922 |
| AF_0522 | 12 | 0.91 | 20819954; 20610394; 19734688; 18550540; 18629030; 17362992; 17210228; 17121880; 16584181; 15927344 |
| AF_0886 | 8 | 0.73 | 15517349; 19196433; 19021765; 1208211; 18024178; 17917424; 17097637; 16901478; 15882061; 15488656 |
| AF_1297 | 18 | 0.88 | 21339705; 21385703; 21303910; 21297161; 21274617; 21245269; 21148420; 21147776; 21111787; 20843792 |
| AF_1498 | 9 | 0.95 | 19550039; 19101548; 17906131; 15498941; 12406578; 8468280; 7883013; 1917873; 1527048; 1508151 |
| AF_1970 | 8 | 0.72 | 21378414; 21266482; 21153002; 20862316; 20455268; 20016946; 19011896; 16894550; 18620420; 15869386 |
| AF_2269 | 18 | 0.94 | 21393174; 21393072; 21392379; 21390069; 21389634; 21383919; 21383019; 21378167; 21372393; 21372131 |
| AFE_0012 | 14 | 0.96 | 20047307; 19683539; 18536021; 17425969; 17384901; 17191912; 999839; 17143579; 16928426; 16714288 |
| AFE_0169 | 18 | 0.75 | 20387456; 20041993; 19731368; 15223320; 18462159; 19432807; 19153809; 8830709; 18043643; 17997964 |
| AFE_0444 | 18 | 0.84 | 21342126; 21321137; 21319959; 21291474; 21284934; 21279999; 21271607; 21265097; 21262925; 21241819 |
| AFE_0501 | 8 | 0.89 | 21376015; 21038895; 20609914; 19549597; 18991392; 18981569; 18945673; 18930846; 2542902; 18472393 |
| AFE_0568 | 19 | 0.83 | 21377170; 21362626; 21349544; 21346816; 21340723; 21329659; 21327158; 21317879; 21302608; 21277938 |
| AFE_0846 | 12 | 0.97 | 20064164; 19383693; 18331405; 2170332; 18064454; 17229735; 16448505; 16407185; 10652088; 11922668 |
| AFE_0949 | 6 | 0.82 | 21383239; 21382109; 21372093; 21369983; 21362510; 21362508; 21354180; 21353876; 21347346; 21339843 |
| AFE_1070 | 8 | 0.85 | 20876192; 20675101; 10987289; 17196981; 7181513; 16126223; 12604241; 11306094; 11277923; 9133741 |
| AFE_1264 | 1 | 0.95 | 20961734; 20871626; 18481057; 16960959; 15526543; 12670956; 8719248; 11032894; 9565331; 8933102 |
| AFE_1316 | 8 | 0.80 | 20934402; 19465092; 18945673; 18757814; 18216065; 18000703; 10467097; 17944363; 17669423; 16820168 |
| AFE_1433 | 8 | 0.85 | 20030377; 19959582; 19368556; 18298089; 17203313; 16962969; 15485884; 10518782; 12605683; 11951047 |
| AFE_1823 | 6 | 0.92 | 21087076; 21079245; 21169503; 21046176; 20929866; 20962275; 20858457; 20826341; 20817635; 20805881 |
| AFE_1962 | 8 | 0.72 | 9630213; 18991392; 18393449; 12517450; 15654874; 10427749; 9852122; 8980517; 7557016; 7479692 |
| AFE_2041 | 19 | 0.93 | 21397493; 21342541; 21233160; 21126315; 21106106; 21098513; 21070855; 21040749; 20980614 |
| AFE_2097 | 8 | 0.92 | 19923715; 19574215; 19110079; 18216065; 10725534; 17313403; 17131148; 16919403; 15362852; 15302401 |
| AFE_2163 | 12 | 0.87 | 20704592; 19477906; 1946424; 9642219; 18310028; 17937616; 17213679; 16135093; 16081041; 15823018 |
| AFE_2230 | 8 | 0.72 | 20977213; 20705057; 20673834; 20653766; 20636266; 20614874; 20592025; 20558724; 20538584; 20518024 |
| AFE_2231 | 2 | 0.74 | 20030377; 19952527; 19122276; 18716757; 16407262; 18390908; 18298089; 17894815; 17676770; 17649978 |
| AFE_2232 | 8 | 0.76 | 12198487; 19082940; 18639656; 16154088; 18279395; 18216065; 17848555; 17669423; 17466607; 17094969 |
| AFE_2251 | 8 | 0.75 | 21345329; 21324338; 21323982; 21317335; 21286787; 21219654; 21216959; 21178073; 20946603; 20943535 |
| AFE_2290 | 8 | 0.81 | 21195229; 8416294; 10715138; 9813031; 9572839; 9308890; 8591049; 3374614; 891918; 3612800 |
| AFE_2601 | 8 | 0.79 | 21333622; 20929256; 20673213; 20364820; 19405028; 19399763; 11779234; 16049196; 16236509; 18803552 |
| AFE_2789 | 1 | 0.70 | 21077527; 20961734; 20832591; 20056897; 19812443; 19604246; 19601701; 19235869; 17579023; 19100381 |
| AFE_2911 | 9 | 0.90 | 19324564; 6997270; 18400514; 17328767; 17249420; 9634230; 14708602 |
| AFE_3006 | 19 | 0.96 | 21097635; 21070413; 21040511; 20724636; 20708437; 20610395; 20829379; 20497501; 20497501; 20497501 |
| AFE_3093 | 15 | 0.91 | 9838063; 17968423; 18974048; 16428390; 17310401; 12142437; 15458412; 11916257; 6096360 |
| AFE_3094 | 2 | 0.87 | 20923139; 16230336; 12753928; 11435415; 10428762 |
| AFE_3118 | 13 | 0.90 | 16245325; 18426891; 19591841; 19446527; 19407389; 18565343; 1735704; 11532950; 16403873; 16245325 |
| AHA_0454 | 8 | 0.83 | 20064493; 18987457; 16483840; 15963890; 15753113; 11432745; 7464065; 10816096; 3192527; 7540767 |
| AHA_0464 | 8 | 0.77 | 18416820; 7809062; 17298562; 15470127; 15114416; 12904832 |
| AHA_0466 | 2 | 0.93 | 20823222; 20543190; 12864856; 4345352; 8012595; 3663113; 9217019; 9163953; 8621563; 7607249 |
| AHA_0492 | 5 | 0.85 | 21378286; 21369825; 21362161; 21356310; 21351076; 21347431; 21342037; 21334454; 21326216; 21319344 |
| AHA_0547 | 12 | 0.97 | 21397184; 21397183; 21395266; 21385720; 21384831; 21382499; 21379584; 21379326; 21378058; 21375464 |
| AHA_0648 | 19 | 0.74 | 20197408; 20234387; 10824085; 16873721; 18247350; 12100553; 15766258; 10449423; 1100429 |
| AHA_0861 | 2 | 0.76 | 20861021; 8631762; 11283592; 12605683; 9615466; 1324835; 9182554; 7782304; 3529824; 2747620 |
| AHA_0862 | 15 | 0.77 | 21257773; 20971871; 19785462; 18792053; 19220743; 18279347; 16740922; 11961556; 9818746; 11728710 |
| AHA_0979 | 19 | 0.80 | 21159160; 21097664; 20948902; 20927641; 20302895; 20068354; 20484982; 19822229; 19699767; 19665502 |
| AHA_1029 | 2 | 0.72 | 20601509; 18606475; 19305405; 19016879; 17469805; 17368066; 11578927; 16221578; 10212987; 15610037 |
| AHA_1205 | 12 | 0.82 | 21397717; 21354180; 21183666; 21097470; 20923350; 19055692; 19706509; 20368341; 12068309; 20160719 |
| AHA_1312 | 8 | 0.73 | 21362485; 21354471; 21335525; 21332407; 21329881; 21322032; 21299470; 21278127; 21226334; 21181421 |
| AHA_1314 | 12 | 0.87 | 21183673; 21178363; 20944232; 20837014; 20233921; 10612322; 10085027; 11371196; 18086211; 18715016 |
| AHA_1392 | 6 | 0.91 | 21354867; 21263027; 21214869; 21211720; 21111787; 21090726; 21081488; 21050475; 21059914; 20937877 |
| AHA_1495 | 8 | 0.72 | 19583210; 1708392; 15063311; 11466310; 9149148 |
| AHA_1541 | 4 | 0.72 | 19735955; 19412608; 17971082; 17908227; 15509585; 10521424; 2087222; 9988687; 3321061 |
| AHA_1575 | 13 | 0.71 | 20926388; 19707755; 15700423; 11497461; 10090755; 8360919 |
| AHA_1576 | 15 | 0.90 | 20870771; 20592028; 17559414; 17434492; 16573700; 16000707; 5432063; 10338008; 11158442; 11121759 |
| AHA_1639 | 15 | 0.75 | 20594941; 20133363; 16945692; 16953632; 4352175; 18245243; 18604637; 18093135; 18022383; 12358600 |
| AHA_1865 | 4 | 0.92 | 19788546; 18391964; 9495771; 17360038; 17259614; 10648549; 11973144; 9252185 |
| AHA_2172 | 2 | 0.88 | 20861021; 20690630; 19122276; 18006324; 7815950; 8010978; 2628174 |
| AHA_2180 | 6 | 0.78 | 21398294; 21397848; 21393220; 21389271; 21387407; 21383776; 21382709; 21378182; 21365485; 21364035 |
| AHA_2226 | 18 | 0.75 | 21357734; 21123545; 21091436; 21052783; 20980406; 20875863; 20807531; 20525694; 12409616; 20423832 |
| AHA_2452 | 18 | 0.84 | 20008538; 2150414; 10926494; 7592901; 11574548; 11238741; 3159677; 808529 |
| AHA_2970 | 18 | 0.93 | 19425588; 1715858; 19166984; 12107133; 14714867; 10092468; 12791142; 11104814 |
| AHA_3098 | 19 | 0.83 | 21397215; 21393864; 21393839; 21390509; 21385872; 21367878; 21360139; 21359176; 21346408; 21327387 |
| AHA_3216 | 6 | 0.86 | 21389349; 21385054; 21383065; 21378168; 21367819; 21163962; 21349834; 21339843; 21316665; 21311766 |
| AHA_3305 | 12 | 0.79 | 21393845; 21384999; 21383483; 21367893; 21365622; 21364762; 21359008; 21339167; 21332393; 21323891 |
| AHA_3362 | 15 | 0.96 | 21252292; 20176937; 20032313; 15537533; 12388300; 11108719; 7939686; 1655112; 2847147 |
| AHA_3398 | 9 | 0.81 | 21199673; 20925342; 20662933; 20562282; 20557983; 20304657; 20132826; 20018879; 19874137; 19427006 |
| AHA_3498 | 4 | 0.82 | 21278288; 20472726; 19396940; 11325945; 17965153; 11687520; 15660156; 17630974; 10222208; 16751195 |
| AHA_3769 | 18 | 0.76 | 21080032; 18462159; 15994322; 14763978; 14731273; 11418569; 9545377; 11731159; 11524163; 2254026 |
| AHA_3790 | 6 | 0.80 | 20950419; 20837709; 20713467; 20518483; 20512115; 20423318; 20129927; 20018860; 19779462; 19608746 |
| AHA_4139 | 1 | 0.90 | 20961734; 20180123; 20056897; 19922747; 19912437; 19601701; 19471856; 7901896; 18555930; 18414142 |
| AHA_4252 | 4 | 0.78 | 21339705; 21272169; 21203349; 21187329; 21183954; 21173114; 21173113; 21124864; 21118147; 21117236 |
| ANA_0192 | 8 | 0.79 | 18804703; 16185084; 15349715; 12061812; 11004530; 15299543; 10049651; 9701462; 9519408; 9454603 |
| ANA_0242 | 6 | 0.80 | 20233727; 20225159; 15538360; 20026132; 15935758; 19199647; 17032235; 10322435; 15351786; 12243751 |
| ANA_0283 | 8 | 0.83 | 20528775; 19807880; 17215140; 17080620; 12226404; 8300616 |
| ANA_0293 | 4 | 0.75 | 20348251; 10998175; 19131424; 11144419; 10952626; 10524961; 14673771; 10529352; 1838574 |
| ANA_0348 | 18 | 0.91 | 21106004; 20507120; 20450914; 20411915; 20401926; 20226783; 17280684; 2007592; 10952578; 19931508 |
| ANA_0405 | 6 | 0.96 | 21363965; 21360615; 21330526; 21306445; 21268715; 21266475; 21261070; 21215703; 21154877; 21151113 |
| ANA_0411 | 8 | 0.95 | 21117708; 21074578; 21042417; 20943545; 20823222; 20808932; 20676631; 20620150; 15987803; 20603809 |
| ANA_0554 | 8 | 0.90 | 21166653; 20889786; 20851994; 20632934; 20171064; 19921179; 19395484; 19824993; 19588068; 19506862 |
| ANA_0608 | 4 | 0.71 | 20507997; 20117010; 19770041; 19695338; 19135125; 15695811; 18005663; 17270289; 10940244; 1638630 |
| ANA_0622 | 18 | 0.93 | 19911131; 16286358; 1008746; 9720051; 9133319; 3896791; 2674131; 14907713 |
| ANA_0736 | 8 | 0.73 | 21178285; 20811135; 19966484; 19546514; 19424620; 19200041; 17291445; 16402358; 16221991; 15996793 |
| ANA_0762 | 19 | 0.95 | 20865175; 20210661; 11320139; 15687380; 18173801; 17426021; 12468728; 15032825; 14997492; 11748726 |
| ANA_0786 | 9 | 0.98 | 21204864; 21098489; 21092192; 20736083; 20662770; 20941840; 20136099; 20013982; 19619887; 19362635 |
| ANA_0819 | 1 | 0.76 | 18062262; 16921527; 16078071; 1849480; 2824247; 2836080 |
| ANA_0979 | 5 | 0.96 | 20532909; 20460772; 20386573; 20359902; 20156691; 20124698; 20095968; 20095967; 20013010; 19997761 |
| ANA_0986 | 1 | 0.87 | 20847010; 20648599; 12631323; 15223311; 14629006; 12525491; 2422596 |
| ANA_0997 | 18 | 0.73 | 21400689; 21391228; 21389615; 21389046; 21388220; 21384913; 21384882; 21383163; 21377658; 21370880 |
| ANA_1073 | 19 | 0.82 | 20921376; 20863279; 20208062; 20147450; 19763417; 19671924; 19571171; 11119490; 19302485; 8293964 |
| ANA_1091 | 12 | 0.95 | 21366318; 21122159; 20946650; 20616104; 20002189; 11279114; 19439009; 12207705; 18954360; 18832305 |
| ANA_1161 | 2 | 0.75 | 20955688; 20593767; 20560533; 11180061; 10961912; 19129660; 19055484; 16666457; 18693754; 6134288 |
| ANA_1235 | 8 | 0.72 | 21345790; 20736169; 20607749; 20418438; 20410293; 20302306; 20089292; 17847094; 20055482; 20049620 |
| ANA_1245 | 18 | 0.97 | 21385839; 21362161; 21356339; 21350490; 21347707; 21308399; 21296088; 21170876; 21272644; 21251105 |
| ANA_1300 | 19 | 0.98 | 21117235; 20700743; 20197075; 20164147; 20045480; 20043969; 19884781; 19576307; 19428473; 18174188 |
| ANA_1334 | 6 | 0.86 | 21265761; 20671029; 20456941; 20451531; 20420913; 20233727; 20225159; 17189189; 15538360; 20109556 |
| ANA_1381 | 12 | 0.97 | 11750807; 7761092; 16361710; 9015299; 10412982 |
| ANA_1591 | 15 | 0.90 | 20144684; 10629763; 18649864; 8402178; 1528892; 1633609; 1828858; 2449695; 1669444; 2270287 |
| ANA_1667 | 8 | 0.73 | 21173220; 20960097; 20951673; 20920510; 20632369; 20551992; 20503330; 20498024; 20484225; 20430815 |
| ANA_1668 | 8 | 0.73 | 21166997; 20969756; 17205209; 20097860; 19214514; 11717466; 18765295; 10406803; 18433447; 18160053 |
| ANA_1710 | 18 | 0.93 | 8606194; 17657347; 16889884; 16388822; 16245943; 15794653; 10781104; 15485658; 11226186; 10473554 |
| ANA_1936 | 18 | 0.99 | 21398536; 21398480; 21395979; 21395556; 21395536; 21394083; 21392495; 21391724; 21391691; 21388881 |
| ANA_1976 | 13 | 0.80 | 21376540; 21114012; 21078604; 20873482; 20852270; 20823289; 20614151; 19232867; 20000576; 19808234 |
| ANA_2044 | 11 | 0.84 | 19733517; 19479257; 18941994; 16330101; 17191617; 16233453; 15591786; 10671461; 14667822; 11798281 |
| ANA_2059 | 18 | 0.87 | 21389396; 21365963; 21359570; 21314574; 21256497; 21256492; 21227764; 21221632; 20958204; 20929469 |
| ANA_2316 | 6 | 0.96 | 21278727; 21245169; 21238786; 21234761; 21229606; 21187428; 21187328; 21187070; 21141724; 21115420 |
| ANA_2394 | 18 | 0.79 | 21369825; 21245145; 21209283; 21197505; 21191290; 21122813; 21115658; 21090806; 21059948; 21059651 |
| ANA_2404 | 14 | 0.88 | 12023031; 9598121; 7945284; 1659312; 2713269; 6587184 |
| ANA_2436 | 18 | 0.75 | 21190215; 21159967; 21048938; 20739949; 20686232; 20643189; 20628025; 18004396; 20606385; 20603149 |
| ANA_2439 | 8 | 0.72 | 20473443; 3545499; 19361226; 18692066; 18331844; 18093543; 17956189; 17675287; 16179963; 15894171 |
| ANA_2503 | 4 | 0.85 | 20413229; 19172878; 18084895; 6303219; 12393925; 15886207; 10536008; 15178486; 11222594; 15105503 |
| ANA_2597 | 18 | 0.75 | 20876117; 20573782; 20562312; 20543096; 19455095; 19274344; 7523951; 6777774; 1698394 |
| APH_0311 | 8 | 0.85 | 21397737; 21265776; 21057504; 21041678; 21038477; 20970991; 20929961; 20869368; 20831589; 20826164 |
| APH_1096 | 13 | 0.83 | 21241052; 15657145; 19661429; 7482695; 6358898; 19351057; 19161851; 1406490; 321008; 17924654 |
| ATORI0001_0017 | 2 | 0.93 | 21206014; 21156797; 21081498; 20861021; 20690630; 20112869; 20039101; 19946146; 19479286; 19330765 |
| ATORI0001_0741 | 8 | 0.92 | 21393833; 21391225; 21388960; 21387410; 21386928; 21386095; 21384112; 21383242; 21383078; 21383058 |
| ATORI0001_0824 | 6 | 0.86 | 21396120; 21385054; 21338660; 21320783; 21310715; 21289264; 21268059; 21245904; 21220303; 21220120 |
| ATORI0001_0915 | 13 | 0.81 | 20657179; 17237168; 19636801; 19246764; 5326242; 17704055; 10419966; 15189152; 12860123; 10655208 |
| ATORI0001_0946 | 19 | 0.97 | 21369910; 21354463; 21354427; 21318364; 21317267; 21311881; 21304822; 21290547; 21266081; 21265544 |
| ATORI0001_1081 | 8 | 0.70 | 21239486; 21152904; 21285043; 20643204; 20564235; 20458544; 20399845; 19743417; 19534905; 19292873 |
| ATORI0001_1346 | 1 | 0.93 | 18481057; 17575227; 17135341; 15803651; 16840914; 2610349; 16428494; 14742742; 12939586; 10684605 |
| BA_0704 | 18 | 0.89 | 21310956; 21281657; 21097502; 21041667; 21035104; 20881245; 20826817; 20797682; 20797400; 20696931 |
| BA_2057 | 8 | 0.73 | 21357486; 21322495; 21322489; 21319715; 21299212; 21277857; 21277289; 21220625; 21216999; 21209222 |
| BA_2405 | 19 | 0.86 | 21378199; 21369973; 21369910; 21357626; 21357429; 21324659; 21311881; 21307589; 21297160 |
| BA_4366 | 15 | 0.90 | 17533171; 11020243; 9756934; 4983359; 1657879; 2666673; 1103845; 2835662 |
| BA_4502 | 2 | 0.90 | 20534554; 20172995; 19907096; 19861490; 19021881; 18211084; 17214985; 16480720; 15978585; 15792953 |
| BA_4943 | 13 | 0.72 | 21320742; 20696925; 14627813; 17868095; 20052680; 10880568; 19894214; 19851001; 19720141; 14622403 |
| BAA_0386 | 19 | 0.72 | 20032185; 18760362; 18342249; 16368098; 16203204 |
| BAA_0409 | 8 | 0.88 | 20609914; 19549597; 18991392; 18930846; 18211101; 17385893; 2172928; 5700707; 16584639; 9000629 |
| BAA_0418 | 15 | 0.85 | 21315771; 21257771; 21219466; 21078995; 21050859; 21037181; 20966074; 20946846; 20835487; 20823675 |
| BAA_0491 | 6 | 0.74 | 20656798; 20434457; 20167799; 20044377; 20018860; 20010811; 10600563; 8505306; 19747545 |
| BAA_0624 | 19 | 0.75 | 21393206; 21356201; 21163680; 21044268; 20974151; 20965199; 20870765; 20849416; 20580675; 20453092 |
| BAA_0672 | 2 | 0.96 | 21190337; 20863064; 20861021; 20453090; 20040598; 20038499; 15680760; 19415239; 19006273; 16535143 |
| BAA_0888 | 12 | 0.86 | 21183667; 20135153; 17108957; 17428499; 16488470; 15805776; 10757787; 10543950; 11994302; 11709175 |
| BAA_1185 | 19 | 0.88 | 20833576; 20447285; 20439716; 20188068; 16407994; 20383071; 19737363; 19730766; 19423628; 11371196 |
| BAA_1532 | 13 | 0.98 | 19211306; 10679383; 1535557; 11526482; 12860121; 12437189; 12054647; 12047221; 11866091; 11710515 |
| BAA_1760 | 4 | 0.71 | 15916611; 9426140; 8200538; 8421316; 1474584; 14220656; 1527488; 7353999; 7056757; 3039149 |
| BAA_1928 | 8 | 0.91 | 21400100; 21397737; 21396889; 21396131; 21394044; 21393444; 21393246; 21393237; 21388872; 21387012 |
| BAA_1944 | 15 | 0.98 | 20833311; 20511236; 18757803; 17335289; 10632884; 16629668; 16604267; 16436431; 15289568; 12777072 |
| BAA_1991 | 19 | 0.79 | 21226054; 20963614; 20931864; 20604568; 20586063; 20554800; 20435896; 20396964; 20304922; 357 |
| BAA_2232 | 8 | 0.74 | 21383138; 21349748; 21345800; 21319793; 21265777; 21259411; 21247928; 21210640; 21198648; 21166638 |
| BAA_2371 | 15 | 0.70 | 21402878; 21402705; 21402151; 21402149; 21401527; 21400516; 21399626; 21398611; 21396111; 21393855 |
| BAA_2901 | 8 | 0.89 | 19700851; 17079028; 15667938; 11243797; 10333487; 1849605; 2643092; 6352260 |
| BAA_3060 | 15 | 0.81 | 21318274; 21315771; 21283542; 21257771; 21219466; 21078995; 21062468; 21051490; 21050859; 21047084 |
| BAA_3468 | 8 | 0.82 | 20647035; 20103717; 18493020; 19121282; 18558362; 18066402; 18043863; 17185023; 17157446; 16607506 |
| BAA_3530 | 8 | 0.84 | 18560155; 18027933; 12455953; 15667305; 8033912; 14646063; 12444973; 2504284; 3843705; 9504047 |
| BAA_3696 | 19 | 0.94 | 21054166; 20868765; 20666458; 20656870; 20469649; 20382819; 20223653; 20201406; 19948399; 19596709 |
| BAA_4344 | 6 | 0.91 | 19399991; 15876366; 14756795; 11428897; 10568806; 1406951; 9714164 |
| BAA_4387 | 15 | 0.90 | 17533171; 11020243; 9756934; 4983359; 1657879; 2666673; 1103845; 2835662 |
| BAA_4952 | 13 | 0.72 | 21320742; 20696925; 14627813; 17868095; 20052680; 10880568; 19894214; 19851001; 19720141; 14622403 |
| BAA_5007 | 18 | 0.70 | 21394800; 21279681; 21196952; 21157892; 20826681; 20739287; 20595047; 14963042; 20530577; 20421303 |
| BAA_5555 | 18 | 0.97 | 21262457; 20863830; 20812950; 20709745; 19805253; 19711106; 19351728; 17673911; 17329346; 17085564 |
| BAA_5671 | 18 | 0.98 | 21396912; 21123563; 21044582; 20880843; 20823525; 20709747; 20601156; 20605797; 20550899; 20524211 |
| BAA_A0073 | 4 | 0.87 | 17651447; 17980467; 16194238; 12721629; 14715750; 15246505; 11032859; 1688844; 14575176; 10190106 |
| BAA_A0075 | 4 | 0.93 | 12721629; 10708363; 12174044; 19211015; 14493304 |
| BAA_A0102 | 4 | 0.80 | 20597981; 11084608; 10328075; 10708363; 14651647 |
| BAA_A0103 | 4 | 0.85 | 15837378; 8994830; 3926644; 16984418; 14997491 |
| BAA_A0129 | 19 | 0.98 | 20863317; 20008039; 19686342; 19332829; 19302304; 16840511; 12055294; 10376820; 9856344 |
| BAA_B0084 | 18 | 0.98 | 21362400; 21349151; 21346128; 21328631; 21266201; 21264952; 21224231; 21192096; 21187401; 21159064 |
| BAAU0058 | 13 | 0.83 | 20954242; 19894713; 19837083; 19772352; 16758500; 19668194; 19478446; 19258353; 12946347; 18842003 |
| BAAU0078 | 4 | 0.84 | 15231773; 11283287; 9784122; 10842753; 8784208 |
| BAAU0124 | 17 | 0.96 | 21248844; 21233849; 21205867; 21187417; 21183718; 21134269; 21126947; 21124318; 21114873; 21107319 |
| BAAU0171 | 4 | 0.82 | 21398556; 19332816; 3080407; 12010551; 10376819; 9846746 |
| BAAU0189 | 12 | 0.95 | 21084296; 20941418; 19379766; 19303951; 17587577; 18366325; 18979629; 18930847; 18690782; 18590836 |
| BAAU0364 | 1 | 0.71 | 20101025; 19946895; 1476141; 1929327; 17522047; 17372354; 16665431; 14684902; 10939241; 15463143 |
| BAAU0404 | 2 | 0.98 | 12410317; 16912043; 15709744; 10891066; 10350464; 8610181; 791939; 2223790 |
| BAAU0459 | 11 | 0.96 | 21185326; 20890845; 20829283; 20586190; 20473284; 20406289; 9384377; 17900620; 15175280; 19797917 |
| BAAU0554 | 8 | 0.73 | 21178285; 20811135; 19966484; 19546514; 19424620; 19200041; 17291445; 16402358; 16221991; 15996793 |
| BAAU0642 | 8 | 0.92 | 20795494; 16872401; 15316720; 12427946; 10830505; 1310545; 10548510; 9480821; 9022686 |
| BAAU0703 | 19 | 0.80 | 21252271; 21181148; 20861017; 20825156; 20720016; 20622130; 20500083; 10672182; 20173067; 20141481 |
| BAAU0725 | 18 | 0.87 | 20797386; 18675571; 2537813; 11902719; 8031825 |
| BAAU0779 | 4 | 0.83 | 21398556; 21389112; 21378197; 21378181; 21352236; 21299880; 21232149; 21221837; 21208192; 21199192 |
| BAAU0790 | 8 | 0.73 | 21178285; 20811135; 19966484; 19546514; 19424620; 19200041; 17291445; 16402358; 16221991; 15996793 |
| BAAU0797 | 8 | 0.73 | 21178285; 20811135; 19966484; 19546514; 19424620; 19200041; 17291445; 16402358; 16221991; 15996793 |
| BAAU0846 | 4 | 0.80 | 21393196; 20400784; 20127470; 18838593; 18388112; 19353380; 18515487; 18200532; 18096812; 364573 |
| BAAU0902 | 9 | 0.79 | 20511508; 19213219; 18281324; 15458418; 15235808; 10756203; 3136142; 11796109; 10944342; 10769118 |
| BAAU1133 | 4 | 0.83 | 21398556; 21389112; 21378197; 21378181; 21352236; 21299880; 21232149; 21221837; 21208192; 21199192 |
| BAAU1164 | 9 | 0.82 | 20221630; 19595597; 19407376; 19185501; 14523010; 12549938; 11286890; 8655562; 11029699; 10733911 |
| BAAU1190 | 5 | 0.92 | 21223465; 9385560; 16301812; 16233726; 16233126; 12951510; 11488932; 11006082 |
| BAAU1266 | 4 | 0.78 | 20601499; 15652293; 16384799; 12644503; 11222602; 15231773; 11884407; 10949920; 6786720 |
| BAAU1468 | 8 | 0.82 | 15047692; 2033048; 1069307; 18422485; 1310666; 15047692; 12637552 |
| BAAU1469 | 8 | 0.86 | 20050916; 19682263; 16825793; 10844653; 10386372; 9395519 |
| BAAU1580 | 6 | 0.88 | 21087930; 21044871; 20551906; 20347402; 2946419; 12140549; 8808633; 17965729; 17614965; 8336719 |
| BAAU1804 | 9 | 0.94 | 20221630; 19595597; 19407376; 12549938; 11286890; 8655562; 11029699; 10733911; 1551888; 7766878 |
| BAAU1886 | 18 | 0.91 | 19653651; 18355966; 17632081; 17171805; 12851823; 11756511; 10828993 |
| BAAU1903 | 8 | 0.73 | 21296217; 20557447; 20369854; 20230247; 20219383; 19881207; 19759613; 19552509; 19217387; 16985102 |
| BAAU2123 | 8 | 0.74 | 19387485; 17233747; 11939777; 9022686; 1830217 |
| BAAU2636 | 4 | 0.89 | 17651447; 16194238; 12721629; 14715750; 15246505; 11032859; 14575176; 10190106; 6096131 |
| BAAU2884 | 5 | 0.71 | 19770075; 18198899; 17289450; 11893063; 12646375; 15882995; 12770504; 12445781; 11750815; 11734883 |
| BAAU2885 | 18 | 0.91 | 21126504; 20498258; 20018840; 11207551; 2939801; 9401119; 17272682; 15228545; 17074317; 16804679 |
| BAAU2887 | 18 | 0.93 | 11207551; 10648151; 15228545; 8932698; 12829270; 12711602; 10940652; 10480865; 10774736; 8113187 |
| BAAU2894 | 2 | 0.83 | 21041678; 19915531; 19915539; 19232050; 17679145; 15581364; 15450493; 15014152; 14739262; 14501115 |
| BAAU3047 | 19 | 0.78 | 21329659; 21217812; 21212461; 21212096; 20975832; 20945913; 20689156; 20595232; 20426787; 20385771 |
| BAAU3093 | 11 | 0.99 | 16181782; 17078817; 10722135; 10986230; 10556026; 9393713; 2548993; 10760133; 11958563; 11907683 |
| BAAU3105 | 4 | 0.70 | 21378197; 21378181; 16332823; 10476029; 17573930; 12670969; 16242862; 10348844; 10348844; 10411756 |
| BAAU3368 | 8 | 0.74 | 19055545; 11018134; 16634330; 10960103; 12693814; 12612960; 11293064; 10757039; 10463157; 10403335 |
| BAAU3558 | 4 | 0.83 | 21398556; 21389112; 21378197; 21378181; 21352236; 21299880; 21232149; 21221837; 21208192; 21199192 |
| BAAU3869 | 13 | 0.80 | 21037010; 20817755; 20106954; 10196363; 19712588; 19664587; 12228251; 18424795; 18069966; 18039703 |
| BAAU3914 | 9 | 0.84 | 20201588; 12237320; 18384517; 14696379; 12737796; 12737802 |
| BAAU4013 | 8 | 0.73 | 21178285; 20811135; 19966484; 19546514; 19424620; 19200041; 17291445; 16402358; 16221991; 15996793 |
| BAAU4047 | 11 | 0.96 | 21244827; 21062824; 20586190; 16012093; 20473284; 20406289; 20030720; 19223640; 19806374; 18776010 |
| BAAU4121 | 8 | 0.81 | 18354500; 16606613; 16915354; 16176272; 15840565; 15120624; 15103625; 12866046; 12730244; 12423349 |
| BAAU4138 | 4 | 0.84 | 7704255; 16561900; 291033; 6801021; 3931691; 18945; 942051; 5432063 |
| BAAU4690 | 6 | 0.93 | 10606644; 16879643; 6288254; 15629722; 15503140; 10489355; 11821933; 11254128; 10431172 |
| BAAU4834 | 13 | 0.95 | 21367972; 21335519; 21327035; 21320742; 21278229; 21270472; 21255615; 21255425; 21220307; 21219871 |
| BAAU4844 | 19 | 0.88 | 20616068; 20593184; 20522493; 20121195; 10825529; 19414173; 14576423; 19251430; 8680309; 19111640 |
| BAAU4934 | 8 | 0.83 | 21307570; 19373895; 10409645; 9407083; 1995594; 9013577; 8702766; 9144969; 1995594; 1588303 |
| BAAU5116 | 6 | 0.80 | 21087076; 20805881; 20686482; 20675723; 19733176; 230505; 2025413; 18765690; 17346206; 18310346 |
| BAAU5138 | 9 | 0.98 | 19213219; 18281324; 15235808; 3136142; 10387003; 9813046; 7988741; 8253773; 8399347; 8386511 |
| BAAU5151 | 18 | 0.75 | 21388683; 21385431; 21363889; 21359212; 21349546; 21346223; 21339697; 21281699; 21281611; 21278408 |
| BAAU5570 | 18 | 0.96 | 21204798; 20650859; 20519092; 20421284; 20411246; 20219637; 19574231; 19393632; 19292758; 18703537 |
| BAAU5628 | 15 | 0.80 | 21219466; 17994770; 17628151; 10745001; 10331874; 15306019; 1482126 |
| BAAU5629 | 15 | 0.92 | 21219466; 17994770; 17628151; 10745001; 10331874; 16030236; 15306019; 1482126 |
| BACN2286 | 13 | 0.99 | 17061034; 16427818; 14690419; 14672708; 11717415; 11173477; 10460155; 9748544; 4339100; 7590355 |
| BACN3349 | 18 | 0.85 | 9237995; 12529317; 16385119; 12007797; 12436256; 11607110; 7679480 |
| BACN4367 | 12 | 0.78 | 21348431; 21072166; 20980587; 20798394; 20798164; 20615878; 20068039; 20040538; 19846560; 18794886 |
| BAKB0242 | 13 | 0.94 | 18775898; 11283358; 2426258; 18425141; 15845537; 17194940; 16734420; 15882062; 15826650; 15790858 |
| BAMEG_0875 | 18 | 0.72 | 20663878; 20437261; 20060908; 10811910; 9482716; 18271245; 18187053; 10611227; 15840809; 15918885 |
| BAMEG_1802 | 19 | 0.90 | 21052875; 20795767; 19267677; 18723026; 18490894; 18420186; 16868531; 15929981; 15590674; 14907713 |
| BAMEG_2716 | 15 | 0.98 | 20946846; 20817745; 20447287; 20148929; 20127467; 19411418; 20019083; 17183208; 6550579; 12614149 |
| BAMEG_2916 | 4 | 0.77 | 21334427; 21198360; 20803137; 20420917; 20160049; 19951364; 19937156; 19673409; 19549172; 17158472 |
| BAMEG_4527 | 17 | 0.78 | 21335605; 21220056; 21192819; 21078997; 20822113; 20731347; 20718947; 20696927; 20672157; 20655755 |
| BAMEG_5573 | 18 | 0.91 | 21315686; 21311724; 21177413; 21168419; 21122131; 21111784; 21090806; 21078855; 21071707; 21059948 |
| BAMEG_5707 | 15 | 0.96 | 21124821; 20942908; 20870771; 20855615; 20722734; 20639324; 20553556; 20521955; 20348251; 20093290 |
| BAMEG_A0107 | 12 | 0.97 | 11750807; 7761092; 16361710; 9015299; 10412982 |
| BAMEG_A0110 | 18 | 0.82 | 21398432; 21393220; 21392185; 21385626; 21384128; 21383012; 21381722; 21378199; 21378194; 21371898 |
| BARBAKC583_0081 | 12 | 0.81 | 21343416; 21321669; 21311096; 21292744; 21231916; 21204021; 21106534; 20889486; 20856808; 20847048 |
| BARBAKC583_0182 | 8 | 0.77 | 19200363; 19824402; 19815019; 9194175; 7640264; 1577742; 18571669; 18406599; 18077463; 11114333 |
| BARBAKC583_0426 | 19 | 0.93 | 21357534; 21356201; 21341753; 21327035; 21247889; 21241420; 21188584; 21178498; 21167155; 21111546 |
| BARBAKC583_0479 | 8 | 0.73 | 20497500; 20375021; 19299230; 18031345; 17348683; 9918669; 16450403; 16353254; 16051266; 16042597 |
| BARBAKC583_0505 | 12 | 0.87 | 21327044; 20970503; 20946100; 20920237; 20813447; 20598079; 20504072; 19932182; 19866485; 18047580 |
| BARBAKC583_0620 | 9 | 0.83 | 20562282; 20557983; 20304657; 20018879; 19874137; 16436705; 18824113; 6796563; 18574592; 18420136 |
| BARBAKC583_0726 | 18 | 0.91 | 21385703; 21303910; 21297161; 21274617; 21245269; 21148420; 21147776; 21111787; 20843792; 20654624 |
| BARBAKC583_0805 | 8 | 0.86 | 21402159; 21377525; 21372178; 21364950; 21359175; 21354471; 21342093; 21329881; 21322032; 21304598 |
| BARBAKC583_0848 | 6 | 0.77 | 20713134; 19785038; 16357870; 3305486; 1515677; 12646158; 12504018; 11727040; 7606784; 8969238 |
| BARBAKC583_1106 | 8 | 0.77 | 18452539; 18408355; 18088303; 16388583; 15928858; 15726348; 15668249; 12925912; 11307944; 11231268 |
| BARBAKC583_1223 | 19 | 0.92 | 21307593; 21282527; 21146533; 21054875; 20973991; 20944232; 20937888; 20920201; 20885997; 20877647 |
| BARBAKC583_1270 | 9 | 0.79 | 20555380; 20305019; 11217410; 17997080; 2814500; 9417034; 1302187; 7565588 |
| BARBAKC583_1369 | 6 | 0.80 | 20736911; 20613712; 20372103; 20527066; 20107077; 17588928; 18477667; 16157887; 16804709; 12364793 |
| BAWN0075 | 2 | 0.71 | 21282208; 20208151; 20064433; 18451506; 10089455; 18047786; 16321944; 17442674; 17081012; 15649375 |
| BB_0592 | 12 | 0.95 | 20388806; 11750807; 7761092; 16750535; 16361710; 9015299; 10412982; 11343912 |
| BB_G32 | 6 | 0.80 | 20923786; 20716382; 20675469; 20484375; 20130679; 19614620; 17324440; 19053250; 19001846; 17895243 |
| BCA_1407 | 6 | 0.88 | 21306995; 21248859; 21078962; 20800503; 20541511; 17126598; 19747545; 12517334; 18642930; 12798682 |
| BCE_0415 | 19 | 0.99 | 20980996; 20586063; 20384696; 18818215; 20190047; 20127467; 20036252; 10438616; 19493008; 19351587 |
| BCE_1173 | 12 | 0.91 | 20188787; 19556294; 18515364; 12823802; 9004508; 7556197 |
| BCE_1206 | 19 | 0.73 | 21398539; 20943180; 20880355; 2248769; 7494007; 19039666; 18547376; 15486203; 18034862; 10361306 |
| BCE_5541 | 15 | 0.98 | 20946846; 20817745; 20447287; 20148929; 20127467; 19411418; 20019083; 17183208; 6550579; 12614149 |
| BCE_A0221 | 15 | 0.95 | 21183667; 20110293; 19170879; 11831459; 17268768; 12651949; 16941243; 15966722; 15870478; 15702929 |
| BMA_0331 | 8 | 0.80 | 20511298; 18346472; 15210349; 12909015; 11900549; 11563694; 3011794; 8145647 |
| BMA_1342 | 12 | 0.73 | 21347346; 21294679; 21293192; 21276198; 21262504; 21246635; 21241231; 21217173; 21228468; 21221970 |
| BMA_1373 | 8 | 0.71 | 19170879; 15340796; 12393925; 16272392; 13757654; 2020552 |
| BMA_1621 | 1 | 0.80 | 648533; 12715888; 12173932; 11832514; 2515994; 9343344; 8112347 |
| BMA_1801 | 1 | 0.96 | 21305278; 21153519; 21102469; 20883697; 20853201; 20548065; 20199593; 20192272; 20189107; 20052993 |
| BMA_1804 | 8 | 0.81 | 21375708; 21365755; 20967536; 20002588; 19043737; 15299926; 18554861; 12368463; 18154727; 18041955 |
| BMA_2006 | 8 | 0.89 | 21329681; 21265764; 19932956; 19900530; 5432063; 19082766; 18850096; 17640279; 17485854; 17433574 |
| BMA_2724 | 18 | 0.98 | 21369825; 20888343; 20855510; 20656493; 20132828; 19996093; 12642662; 19919676; 19847921; 19733245 |
| BMA_3296 | 12 | 0.76 | 21368759; 21347827; 21163262; 21147776; 21095592; 21062893; 20961849; 20957402; 20947006; 20834233 |
| BMA_A0212 | 1 | 0.84 | 18587583; 17660691; 16458324; 12488095; 11570896; 11389597; 7934817; 10229948; 10213630; 9655946 |
| BMA_A0320 | 8 | 0.94 | 21398106; 21393861; 21392547; 21390523; 21387444; 21387033; 21376957; 21366264; 21364629; 21358763 |
| BMA_A0488 | 12 | 0.84 | 21365649; 21330608; 21233422; 21175594; 21147776; 20849853; 20819954; 20697605; 20691256; 20669962 |
| BMA_A1399 | 8 | 0.74 | 21310479; 21278273; 21197843; 21169482; 20965335; 20923481; 20860559; 20855003; 20838866; 20808573 |
| BMA_A2037 | 9 | 0.98 | 21334330; 21035730; 20851904; 20348430; 20214591; 20214478; 20154153; 19634011; 19391105; 19345228 |
| BMA10229_0318 | 8 | 0.73 | 21091201; 20514241; 19011745; 12003933; 18422649; 17350704; 15680231; 14965227; 12974644; 12388585 |
| BMA10229_0430 | 12 | 0.82 | 19188362; 20388806; 11750807; 7761092; 2050108; 16750535; 16361710; 9015299; 1317006; 10704442 |
| BMA10229_0499 | 8 | 0.85 | 20030377; 19959582; 19368556; 18298089; 17203313; 16962969; 15485884; 10518782; 12605683; 11951047 |
| BMA10229_0659 | 8 | 0.71 | 21383138; 21371503; 21346186; 21340633; 21323543; 21322492; 21307872; 21288331; 21282602 |
| BMA10229_0664 | 6 | 0.75 | 21300889; 21262293; 21220025; 21139085; 21127984; 21124823; 21067515; 21060799; 21052090; 20972222 |
| BMA10229_0679 | 1 | 0.90 | 21305854; 21235381; 21140474; 21088147; 20980339; 20973559; 20890947; 20842630; 20797690; 20738517 |
| BMA10229_0855 | 12 | 0.99 | 20064164; 19383693; 18331405; 2170332; 18064454; 17229735; 16448505; 16407185; 10652088; 15225600 |
| BMA10229_1315 | 8 | 0.79 | 21345800; 21265776; 21265736; 21198553; 21179168; 21073414; 21068394; 21041678; 21030590; 20970991 |
| BMA10229_1393 | 8 | 0.85 | 21111035; 20566695; 19156262; 18678259; 18417118; 16763093; 16501008; 16463101; 16204462; 15720833 |
| BMA10229_1840 | 8 | 0.75 | 21345329; 21324338; 21323982; 21317335; 21286787; 21219654; 21216959; 21178073; 20946603; 20943535 |
| BMA10229_1932 | 2 | 0.86 | 19268473; 2126155; 10622725; 9428706; 9353296 |
| BMA10229_2063 | 15 | 0.78 | 17470586; 1279374; 14680702; 10400588; 2005813; 2823881; 9701813; 1340475; 3027048 |
| BMA10229_A0659 | 14 | 0.80 | 21317337; 19689430; 19114044; 7534998; 18475929; 17723319; 17574432; 17307970; 16780358; 9721275 |
| BMA10229_A0849 | 18 | 0.91 | 21385703; 21303910; 21297161; 21274617; 21245269; 21148420; 21147776; 21111787; 20843792; 20654624 |
| BMA10229_A1117 | 2 | 0.70 | 20815377; 20498089; 19477904; 17121859; 17442677; 17372774; 15379587 |
| BMA10229_A1171 | 18 | 0.95 | 20553812; 20382203; 19283345; 17910062; 16463182; 15610971; 15336429; 12697769; 11955012; 10201393 |
| BMA10229_A1272 | 19 | 0.78 | 21074048; 18759117; 17244817; 16476725; 10379365; 9336672; 15130128; 14617152; 8494882; 11171944 |
| BMA10229_A1610 | 8 | 0.93 | 19923715; 19574215; 19110079; 18216065; 17313403; 17131148; 16919403; 15362852; 15302401; 14530261 |
| BMA10229_A1744 | 9 | 0.99 | 21272378; 20860408; 20634289; 1409538; 14609334; 18550529; 17451746; 11585904; 16740134; 16613860 |
| BMA10229_A1966 | 8 | 0.76 | 21265776; 20847256; 20628895; 20419722; 20226790; 20130031; 20070127; 648533; 19692330; 19688823 |
| BMA10229_A2137 | 4 | 0.77 | 20118266; 19732341; 19332819; 18424518; 10234819; 16949608; 16630628; 16246842; 15817382; 10712687 |
| BMA10229_A2316 | 18 | 0.89 | 21328712; 21262832; 21106122; 21087868; 20976528; 20946596; 20940308; 20890540; 20828128; 20819068 |
| BMA10229_A2442 | 8 | 0.92 | 20956531; 20511503; 20064527; 19883128; 18539730; 10878009; 18560155; 15595725; 11841199; 10430865 |
| BMA10229_A2943 | 2 | 0.84 | 21255303; 21173279; 17584754; 17448684; 16835730; 14612231; 12917443; 12686546; 11215515; 12196147 |
| BMA10247_0048 | 9 | 0.93 | 20601073; 20484448; 10570187; 20385867; 20190408; 19834458; 19546342; 19328666; 19322663; 17909855 |
| BMA10247_0925 | 9 | 0.88 | 10482673; 12454267; 10828604; 9892232; 9804328; 9748261; 9165098; 7668351; 8441459 |
| BMA10247_A2160 | 9 | 0.72 | 20937134; 20725044; 20230056; 19914215; 14766011; 18704941; 18297087; 18272421; 17717183; 16480719 |
| BMA10247_A2176 | 6 | 0.93 | 21184731; 20969882; 19747627; 18776347; 18645645; 17632194; 17119468; 16637637; 15259699; 15045055 |
| BMA10247_A2187 | 15 | 0.78 | 21346779; 21217642; 20654625; 18639517; 17951380; 20487285; 20370801; 20059692; 19949103; 17989689 |
| BMA10247_A2312 | 8 | 0.87 | 21375505; 20190087; 19923715; 19740324; 19574215; 19402045; 19160971; 19110079; 18216065; 10725534 |
| BMA10247_A2338 | 2 | 0.87 | 21372092; 21357486; 21190337; 21120472; 21081498; 21075928; 21072368; 21031595; 20977213; 20863064 |
| BMASAVP1_0289 | 12 | 0.86 | 20802227; 20801112; 20704592; 20540495; 19906845; 19900472; 19477906; 19152375; 18270848; 18032064 |
| BMASAVP1_A2390 | 6 | 0.70 | 21391986; 21251613; 21198747; 21153851; 21145476; 21095589; 21076780; 21037103; 20970991; 20970942 |
| BR_0018 | 9 | 0.93 | 21120858; 21030508; 20947019; 20725044; 20616867; 20615689; 20555365; 15632194; 20379951; 20230056 |
| BR_0140 | 2 | 0.76 | 17639348; 18314963; 17893805; 16162504; 15964837; 15952888; 14993221; 12203014; 12196163; 9813017 |
| BR_0366 | 12 | 0.98 | 21289038; 20064164; 19732346; 19473260; 16381856; 2170332; 18064454; 17229735; 17215254; 16448505 |
| BR_0556 | 17 | 0.71 | 21124318; 11606592; 16337964; 15294156; 15294154; 8286946; 12914690; 9698562; 9512710; 9054416 |
| BR_0622 | 6 | 0.90 | 20823514; 19393175; 11462031; 8948651; 12403791; 15959518; 12018403; 10962003; 9886294; 8366089 |
| BR_0844 | 8 | 0.82 | 19019008; 11732896; 10537203; 10080900; 9846747; 9819231; 2061291; 8026764; 8223657 |
| BR_0853 | 9 | 0.98 | 21370994; 21309865; 21199673; 21135931; 21113689; 21081696; 20943853; 20925342; 20662933; 20594840 |
| BR_0870 | 6 | 0.71 | 21345332; 21343909; 21306995; 21231972; 21229398; 21301438; 21178304; 21156129; 21132514; 21123434 |
| BR_1204 | 18 | 0.90 | 21278256; 21220775; 21190440; 20615634; 20487285; 17601489; 20309544; 20217167; 20107991; 20099411 |
| BR_1286 | 8 | 0.90 | 21091725; 21074979; 20863064; 20846340; 20643656; 20335404; 20116460; 19840105; 19191338; 19171117 |
| BR_1292 | 8 | 0.91 | 21380735; 21319715; 21216999; 21193665; 21183637; 21178073; 21105363; 21102601; 21070747; 20977974 |
| BR_1678 | 13 | 0.70 | 20882017; 20190074; 10587438; 17943273; 17024311; 16164567; 15748981; 15301531; 12796482; 11574542 |
| BR_1868 | 12 | 0.79 | 19116772; 12717624; 17591721; 17464060; 10684935; 9150870; 14651272; 12751333; 12741815; 7984417 |
| BR_1875 | 12 | 0.80 | 20032457; 11751634; 17504491; 17855452; 10569626; 16127432; 16109378; 15249048; 15195946; 15157086 |
| BR_1885 | 4 | 0.75 | 21364304; 21205308; 21035278; 12066186; 20467813; 20363791; 4020112; 17630835; 20172996; 16595014 |
| BR_1962 | 8 | 0.75 | 20494936; 20038611; 19285066; 18334484; 17536015; 16725155; 16705405; 16216576; 15946951; 15665306 |
| BR_2076 | 18 | 0.99 | 20643145; 20202360; 2185139; 18039771; 19373193; 12221291; 10878258; 18006522; 17942072; 17682720 |
| BR_2177 | 8 | 0.75 | 20445235; 15026039; 15284489; 11155166; 11120357; 7831789; 8055948; 8349682 |
| BR_A0239 | 1 | 0.74 | 18062777; 17617175; 14672649; 9047371; 8980513; 8098035; 1906065 |
| BR_A0547 | 19 | 0.93 | 21378034; 21367655; 21342516; 21273341; 21268113; 21255377; 21126522; 21112302; 20971639; 20944403 |
| BR_A0715 | 18 | 0.86 | 19357618; 6767702; 18310071; 15854658; 17384423; 16278237; 8524391; 15271785; 15270511; 11524371 |
| BR_A1190 | 18 | 0.75 | 20387456; 20041993; 19731368; 15223320; 18462159; 19432807; 19153809; 8830709; 18043643; 17997964 |
| BTH_I0079 | 12 | 0.73 | 21347346; 21294679; 21293192; 21276198; 21262504; 21246635; 21241231; 21228468; 21221970; 21213586 |
| BTH_I0274 | 6 | 0.71 | 21362064; 21360181; 21304599; 21299643; 21295415; 21267402; 21257771; 21249192; 21245528; 21243338 |
| BTH_I0275 | 15 | 0.82 | 21315771; 21300840; 21284862; 21257771; 21219466; 21217003; 21216906; 21193607; 21108067; 21097618 |
| BTH_I0505 | 11 | 0.75 | 21402865; 21393370; 21375706; 21368277; 21356525; 21350632; 21335390; 21323982; 21310067; 21277379 |
| BTH_I0619 | 8 | 0.73 | 21372295; 21294649; 21286403; 21285510; 21272192; 21258713; 21215399; 21208358; 21195602; 21193059 |
| BTH_I0719 | 8 | 0.91 | 21402726; 21402159; 21400100; 21397686; 21389620; 21389104; 21388532; 21385584; 21382272; 21382109 |
| BTH_I0812 | 15 | 0.95 | 21375718; 17630784; 10941799; 1588910; 7838735; 8195089; 1532388 |
| BTH_I0820 | 2 | 0.75 | 21255303; 21173279; 19081974; 17584754; 17448684; 16835730; 14612231; 12917443; 12686546; 11215515 |
| BTH_I1035 | 8 | 0.79 | 20920450; 20890095; 20879962; 20865387; 20834161; 20805024; 20739172; 20702080; 20676631; 20673625 |
| BTH_I1329 | 19 | 0.87 | 21397493; 21385202; 21342541; 21347376; 21317159; 21203384; 21166709; 21126315; 21106106; 21102601 |
| BTH_I1359 | 19 | 0.78 | 21397493; 21394312; 21388311; 21385202; 21383969; 21378187; 21371037; 21342541; 21367879; 21366639 |
| BTH_I1475 | 4 | 0.73 | 21397846; 21391297; 21390252; 21390146; 21390130; 21390126; 21389676; 21389279; 21387154; 21383206 |
| BTH_I1477 | 19 | 0.93 | 21279381; 21174346; 20920201; 20672277; 20636376; 3611062; 20383007; 11752303; 20199575; 20124190 |
| BTH_I1482 | 19 | 0.96 | 18499663; 18210176; 15552059; 15226299; 15165190; 14646108; 12686644; 11937332; 11846551; 10610795 |
| BTH_I1577 | 18 | 0.90 | 21329359; 21179522; 21156129; 21080032; 20675494; 20387456; 20095050; 20495348; 20041993; 19731368 |
| BTH_I1925 | 8 | 0.73 | 21178285; 20811135; 19966484; 19546514; 19424620; 19200041; 17291445; 16402358; 16221991; 15996793 |
| BTH_I2100 | 8 | 0.73 | 21178285; 20811135; 19966484; 19546514; 19424620; 19200041; 17291445; 16402358; 16221991; 15996793 |
| BTH_I2101 | 8 | 0.73 | 21178285; 20811135; 19966484; 19546514; 19424620; 19200041; 17291445; 16402358; 16221991; 15996793 |
| BTH_I2283 | 15 | 0.90 | 21347346; 21205010; 21039781; 20965974; 20851895; 20533077; 20497226; 20482591; 20370825; 20116460 |
| BTH_I2340 | 18 | 0.84 | 21187464; 21168419; 21149736; 20554613; 21115899; 21104926; 21077936; 21062971; 21046154; 20935265 |
| BTH_I2343 | 18 | 0.96 | 21393174; 21389634; 21372393; 21366542; 21362586; 21360409; 21351087; 21349151; 21347827; 21345797 |
| BTH_I2358 | 9 | 0.93 | 21253498; 21036334; 20942458; 20834152; 19996150; 19332293; 19324564; 6997270; 18553138; 18400514 |
| BTH_I2463 | 18 | 0.97 | 21392495; 21369825; 21347827; 21304833; 21287809; 21276099; 21261938; 20607690; 21254069; 21262457 |
| BTH_I2464 | 18 | 0.99 | 21402852; 21402783; 21402586; 21401500; 21398536; 21398480; 21395979; 21395556; 21395536; 21394083 |
| BTH_I2554 | 8 | 0.93 | 21354098; 21296938; 21253866; 21238944; 21237726; 21168410; 20960712; 20848047; 20833784; 20815787 |
| BTH_I2658 | 12 | 0.93 | 20368502; 19175361; 19091155; 18938683; 18638487; 18096126; 17987630; 17982898; 17607295; 17400722 |
| BTH_I2691 | 8 | 0.73 | 21178285; 20811135; 19966484; 19546514; 19424620; 19200041; 17291445; 16402358; 16221991; 15996793 |
| BTH_I2692 | 8 | 0.73 | 21178285; 20811135; 19966484; 19546514; 19424620; 19200041; 17291445; 16402358; 16221991; 15996793 |
| BTH_I2962 | 8 | 0.73 | 21178285; 20811135; 19966484; 19546514; 19424620; 19200041; 17291445; 16402358; 16221991; 15996793 |
| BTH_I3119 | 15 | 0.73 | 21403627; 21401619; 21399658; 21398577; 21398529; 21393212; 21392585; 21392507; 21390242; 21390240 |
| BTH_II0085 | 18 | 0.97 | 21392495; 21369825; 21347827; 21304833; 21287809; 21276099; 21261938; 20607690; 21254069; 21262457 |
| BTH_II0120 | 8 | 0.73 | 21178285; 20811135; 19966484; 19546514; 19424620; 19200041; 17291445; 16402358; 16221991; 15996793 |
| BTH_II0289 | 9 | 0.97 | 21135213; 20725044; 20616867; 20379951; 20355329; 1355089; 19097787; 19552511; 1905927; 15968460 |
| BTH_II0293 | 15 | 0.73 | 21124051; 20190084; 19389781; 19307761; 1368202; 18485072; 18219120; 942051; 1956285; 12368433 |
| BTH_II0294 | 15 | 0.88 | 21390327; 21386983; 21385202; 21383239; 21379585; 21375706; 21369825; 21347346; 21340608; 21335458 |
| BTH_II0314 | 18 | 0.95 | 21383014; 21376056; 21346187; 21327105; 21327084; 21304920; 21298091; 21291396; 21285351; 21285349 |
| BTH_II0320 | 2 | 0.85 | 17335870; 16206477; 12736664; 11816029; 10094680; 9055989; 8640549 |
| BTH_II0392 | 12 | 0.87 | 20883740; 20858292; 20495785; 16290006; 20375344; 20083022; 20067781; 20017734; 19966416; 10986670 |
| BTH_II0478 | 9 | 0.96 | 21377632; 21034488; 20622065; 16121256; 19395506; 18032383; 18407998; 18171025; 15904873; 15705744 |
| BTH_II0552 | 19 | 0.91 | 21256827; 21235239; 21220027; 21068446; 21044320; 21042030; 21040561; 20977442; 20938646; 20937835 |
| BTH_II0655 | 1 | 0.71 | 20924576; 20818520; 827241; 19798672; 16857674; 17922758; 17567742; 11495997; 17185548; 17031048 |
| BTH_II0929 | 8 | 0.90 | 20160912; 17923481; 17157320; 15082001; 14992577; 14644451; 12761172; 11955070; 10767328; 10656808 |
| BTH_II1017 | 11 | 0.95 | 12504012; 16859902; 15476403; 16009540; 12535534; 11045004; 1851088; 10873751; 10393199; 10231489 |
| BTH_II1159 | 18 | 0.92 | 21369825; 20855510; 20132828; 19416927; 11756427; 8932698; 16135226; 7934817; 9332349; 642007 |
| BTH_II1291 | 18 | 0.72 | 10470043; 12662939; 9521785; 1738310; 2722818; 271968; 1791754; 1994034; 2184029; 2981624 |
| BTH_II1293 | 18 | 0.92 | 21393174; 21389634; 21372393; 21366542; 21362586; 21360409; 21352852; 21351087; 21349151; 21348297 |
| BTH_II1304 | 8 | 0.73 | 21178285; 20811135; 19966484; 19546514; 19424620; 19200041; 17291445; 16402358; 16221991; 15996793 |
| BTH_II1318 | 2 | 0.72 | 20386493; 20107425; 19858780; 19839929; 19652507; 19571519; 19420703; 16702348; 18283461; 18025275 |
| BTH_II1346 | 8 | 0.73 | 21178285; 20811135; 19966484; 19546514; 19424620; 19200041; 17291445; 16402358; 16221991; 15996793 |
| BTH_II1472 | 8 | 0.82 | 21377632; 21364005; 21362018; 21349860; 21347589; 21345790; 21343423; 21329681; 21325261; 21324174 |
| BTH_II1756 | 4 | 0.75 | 20507997; 20117010; 19770041; 19695338; 19135125; 15695811; 17270289; 10940244; 1638630; 12824352 |
| BTH_II1819 | 15 | 0.83 | 20862323; 19781550; 19699748; 12730324; 18174133; 17569992; 10536136; 11779944; 10081580; 9927482 |
| BTH_II2158 | 8 | 0.90 | 20727352; 20407804; 19916920; 19904831; 19827797; 17468253; 19132060; 18998409; 18355273; 17546202 |
| BTH_II2215 | 18 | 0.89 | 21347827; 21226332; 21210849; 21195340; 21177413; 21157037; 21136441; 21130497; 20554613; 21104926 |
| BTH_II2252 | 4 | 0.83 | 19162196; 17640273; 15576792; 16309817; 9675890; 10361275; 9642082; 8152377; 1453957 |
| BURPS1106A_0044 | 18 | 0.92 | 20952699; 20803137; 20679507; 20470049; 20466765; 20381608; 20351243; 20299401; 20217167; 20199110 |
| BURPS1106A_0239 | 4 | 0.75 | 20582761; 20188560; 19684019; 19243689; 18424518; 8757861; 12236604; 11309126; 18060666; 14614826 |
| BURPS1106A_0485 | 2 | 0.94 | 21081693; 20920246; 11604448; 15347679; 16463102; 10737137; 10022081; 16661655 |
| BURPS1106A_0497 | 8 | 0.88 | 17576516; 15388943; 10437801; 7584858; 6546423; 6288108; 6254527 |
| BURPS1106A_0784 | 2 | 0.94 | 21367973; 21268892; 21117240; 21114499; 20968298; 20956531; 20845493; 20817725; 20684226; 20674574 |
| BURPS1106A_1328 | 1 | 0.82 | 21264378; 21263039; 21188077; 20973559; 20953603; 20693595; 20675481; 20656866; 20656783; 20565114 |
| BURPS1106A_1524 | 5 | 0.87 | 21381661; 21208463; 20957999; 20736171; 20673205; 11387201; 7836277; 19957302; 19580157; 18700747 |
| BURPS1106A_2173 | 8 | 0.80 | 15667938; 11243797; 10333487; 1849605; 2643092 |
| BURPS1106A_2543 | 12 | 0.94 | 20944232; 20837014; 20233921; 11371196; 18086211; 18715016; 17957108; 16381862; 11114514 |
| BURPS1106A_3196 | 18 | 0.77 | 21227585; 18670623; 20154136; 19851727; 1500854; 19640852; 19405026; 19118354; 17030797; 11162101 |
| BURPS1106A_3246 | 15 | 0.85 | 17537210; 17504243; 12675803; 7623377; 9099861; 7961507; 7770032; 1527488 |
| BURPS1106A_3482 | 8 | 0.79 | 18188553; 18063573; 15060078; 16348073; 12188192; 3156376 |
| BURPS1106A_3560 | 2 | 0.85 | 21296186; 19774399; 17876813; 7287745; 10960098; 17977822; 17601793; 17130255; 10865958; 16787928 |
| BURPS1106A_4038 | 9 | 0.86 | 21332878; 21185305; 21181422; 21144863; 21128943; 21106527; 21080948; 21079819; 20944206; 20933624 |
| BURPS1106A_A0203 | 18 | 0.79 | 21156129; 21098297; 20978537; 20947511; 20942504; 20937947; 20890124; 20847098; 20835844; 20739286 |
| BURPS1106A_A0453 | 9 | 0.97 | 16430210; 14728675; 11524729; 10869041; 15146484; 14752098; 12962497; 11872165; 11812788; 11700068 |
| BURPS1106A_A0678 | 12 | 0.92 | 21385872; 21383012; 21375706; 21369989; 21366963; 21365755; 21352460; 21352200; 21334441; 21334427 |
| BURPS1106A_A0838 | 8 | 0.76 | 21216999; 20723539; 20675471; 20460824; 20236932; 20206639; 20147703; 20067464; 19706729; 19546342 |
| BURPS1106A_A0922 | 8 | 0.91 | 19184973; 17887777; 18365259; 17321558; 16339744; 14960302; 8606174; 9864312; 10491136; 8931553 |
| BURPS1106A_A0978 | 8 | 0.88 | 21326935; 21300042; 21277289; 21270753; 21214177; 21185270; 21135126; 21134280; 21113494; 21112834 |
| BURPS1106A_A1240 | 2 | 0.91 | 21367973; 21366574; 21342411; 21288652; 21286325; 21268892; 21247799; 21135073; 21117240; 21117170 |
| BURPS1106A_A1457 | 12 | 0.91 | 21296967; 21296878; 21245388; 21195694; 21166997; 21072667; 21041298; 20926695; 20882138; 20877006 |
| BURPS1106A_A1712 | 9 | 0.97 | 16430210; 14728675; 11524729; 10869041; 15146484; 14752098; 12962497; 11872165; 11812788; 11700068 |
| BURPS1106A_A1713 | 9 | 0.95 | 21193572; 20534481; 20452448; 20378648; 20334431; 20151199; 20150517; 19954230; 19395484; 19693513 |
| BURPS1106A_A2116 | 12 | 0.98 | 21303942; 21261463; 20871837; 20713620; 20679205; 20667621; 19763421; 19729222; 19696109; 19553530 |
| BURPS1106A_A2341 | 4 | 0.72 | 20878241; 19441223; 19385994; 19220744; 18852008; 10582163; 18164725; 18069746; 17222936; 10355354 |
| BURPS1710b_A0125 | 8 | 0.73 | 21178285; 20811135; 19966484; 19546514; 19424620; 19200041; 17291445; 16402358; 16221991; 15996793 |
| BURPS1710b_A0469 | 18 | 0.77 | 21394612; 21394086; 21393453; 21388224; 21385714; 21383316; 21382200; 21378553; 21378313; 21375769 |
| BURPS1710b_A0722 | 8 | 0.81 | 21333651; 21108829; 21041373; 20878365; 20864674; 20519277; 20388558; 20335176; 20136502; 19659575 |
| BURPS1710b_A0895 | 18 | 0.89 | 14560025; 15797382; 15618217; 14981507; 9644977; 10367886; 7556166; 9367739 |
| BURPS1710b_A1872 | 19 | 0.77 | 21158480; 21118988; 21104698; 20871989; 20669918; 20552260; 20522493; 20444687; 20227065; 20159465 |
| BURPS1710b_A1907 | 14 | 0.85 | 21273634; 21207045; 21204885; 21122056; 21103868; 20883226; 20825306; 20673591; 20639015; 20580963 |
| BURPS1710b_A2393 | 8 | 0.76 | 21389623; 21382335; 21380809; 21380594; 21376665; 21376267; 21365530; 21345193; 21341543; 21340422 |
| BURPS1710b_A2581 | 12 | 0.87 | 15832497; 15483401; 11533066; 10824104; 9783261; 3375068 |
| BURPS1710b_B0333 | 9 | 0.93 | 21235239; 20696925; 19797355; 17199298; 19118366; 11036020; 15865210; 14675432; 12664133; 12140293 |
| BURPS1710b_B0529 | 8 | 0.73 | 21178285; 20811135; 19966484; 19546514; 19424620; 19200041; 17291445; 16402358; 16221991; 15996793 |
| BURPS1710b_B0531 | 19 | 0.72 | 21378199; 21357626; 21297160; 21282527; 21273488; 21256135; 21245531; 21238925; 21228360 |
| BURPS1710b_B0596 | 1 | 0.93 | 20973559; 20953603; 20693595; 20675481; 20656866; 20656783; 20565114; 20214682; 20190091; 20683430 |
| BURPS1710b_B1619 | 18 | 0.89 | 21366555; 21257186; 21153644; 21070948; 20955520; 20865392; 20848165; 20838661; 20736046; 20585949 |
| BURPS1710b_B1638 | 9 | 0.99 | 15690397; 10531379; 12788067; 8384302; 9817842; 11027523; 9382842; 7500386; 8302582; 1832554 |
| BURPS1710b_B2342 | 8 | 0.87 | 21265776; 21068394; 18399988; 18323662; 18237633; 16364235; 11300770; 11213485; 10828978; 9610381 |
| BURPS1710b_B2693 | 8 | 0.73 | 21178285; 20811135; 19966484; 19546514; 19424620; 19200041; 17291445; 16402358; 16221991; 15996793 |
| BURPS668_0105 | 11 | 0.71 | 20543074; 17367389; 15469518; 2402433; 12082109; 10969077; 9862965; 1099217; 7625275; 2947238 |
| BURPS668_0486 | 4 | 0.81 | 21178435; 21046450; 21040514; 21038480; 20876224; 20607764; 20446763; 20416426; 20118250; 20109152 |
| BURPS668_1435 | 8 | 0.86 | 21059110; 20075073; 19953240; 17600048; 19654341; 19542013; 11257125; 19265687; 18398174; 10802060 |
| BURPS668_1913 | 8 | 0.92 | 21300042; 21212945; 20654618; 19895910; 19766037; 19694383; 19393548; 19337691; 19303876; 6684148 |
| BURPS668_2114 | 19 | 0.70 | 21372094; 18667582; 16809100; 16481518; 15927297; 15652425; 15296426; 15292190; 15129439; 12585481 |
| BURPS668_2226 | 12 | 0.86 | 21298303; 21250657; 21199674; 21073469; 21062041; 21049911; 20873968; 20717665; 20499648; 20434514 |
| BURPS668_3535 | 8 | 0.91 | 15987803; 20533395; 20303962; 20217243; 15347752; 20085745; 19760337; 19038229; 19026783; 18614015 |
| BURPS668_A0759 | 19 | 0.93 | 21357479; 21307593; 21240541; 21167943; 21125394; 21085702; 21070667; 20976701; 20873227; 20829286 |
| BURPS668_A1007 | 8 | 0.79 | 16546999; 15520003; 9696761; 12605683; 9020134; 8194605; 1905152 |
| BURPS668_A1336 | 8 | 0.91 | 21389620; 21389104; 21388532; 21385584; 21377525; 21355852; 21353756; 21346809; 21346101; 21345799 |
| BURPS668_A1742 | 8 | 0.93 | 21388533; 21335099; 21212945; 21146502; 20889786; 20873171; 20834176; 20717664; 20627615; 20519920 |
| BURPS668_A1779 | 8 | 0.89 | 15389597; 19115036; 17294170; 16027951; 14705036; 7826011; 14638414; 12855725; 11566129; 2971647 |
| BURPS668_A2404 | 8 | 0.80 | 21397737; 21041678; 21030590; 20866105; 20849886; 20809650; 20727152; 20089849; 20629071; 20486243 |
| BURPS668_A2765 | 19 | 0.90 | 20976701; 18550550; 16099208; 18342249; 18314348; 17521358; 17074909; 17007421; 16233217; 15251431 |
| BURPS668_A2850 | 13 | 0.94 | 21102444; 20974854; 20965064; 20947765; 20890290; 20876129; 20807316; 20724456; 20712004; 20670889 |
| BURPS668_A3143 | 12 | 0.94 | 21125380; 20847219; 20812717; 20599707; 20487024; 20485744; 20333370; 20064164; 19799526; 19778964 |
| BURPS668_A3229 | 19 | 0.95 | 21183069; 20843347; 20497333; 20192190; 6351730; 17646652; 9238107; 18539032; 10564478; 17938168 |
| CAMGR0001_0051 | 8 | 0.80 | 21117708; 19799467; 19734121; 19478453; 19367707; 19361226; 18945673; 1310666; 4209578; 8522504 |
| CAMGR0001_0113 | 18 | 0.87 | 21323665; 21199668; 20678548; 20713719; 20346991; 20213554; 19614867; 19467704; 19077146; 16902062 |
| CAMGR0001_0181 | 18 | 0.90 | 21084292; 20971896; 20546934; 20504255; 20498067; 20472555; 20460828; 20438316; 20153493; 20127029 |
| CAMGR0001_0182 | 12 | 0.98 | 20064164; 20010851; 19863457; 19383693; 18331405; 2170332; 18064454; 17229735; 16466653; 16448505 |
| CAMGR0001_0561 | 5 | 0.82 | 20851900; 9838063; 19337310; 18657428; 15755455; 17469805; 11487696; 16684499; 15583009; 14506286 |
| CAMGR0001_0860 | 18 | 0.97 | 20192961; 10827169; 19711123; 15069191; 16304597; 12368463; 17652430; 16968739; 14505932; 10404179 |
| CAMGR0001_1500 | 19 | 0.84 | 21030539; 20707404; 18258263; 16040347; 18499663; 18325534; 18210176; 16829524; 16794327; 15820665 |
| CAMGR0001_2019 | 8 | 0.71 | 20405215; 19908864; 18957412; 18160405; 15581577; 10852721; 10368269; 9490068; 1529349 |
| CAMRE0001_0308 | 19 | 0.81 | 21327035; 21241420; 20971910; 20971903; 20497501; 20497501; 20487285; 20199110; 20059692; 19348786 |
| CAMRE0001_0880 | 13 | 0.99 | 21404088; 21403627; 21402928; 21402779; 21402715; 21402132; 21400704; 21400228; 21399643; 21398531 |
| CAMRE0001_2212 | 18 | 0.82 | 21357485; 21245269; 21210168; 21187326; 21173115; 20693326; 20565992; 17442372; 20484375; 20472799 |
| CAMRE0001_2392 | 4 | 0.77 | 21282416; 20410299; 12162953; 11739521; 16668369; 10377992; 7581999; 7043737; 2067020 |
| CAMRE0001_2433 | 11 | 0.84 | 21364304; 21349326; 21318311; 21241792; 21214923; 21205308; 21192634; 21176936; 21144870; 21138844 |
| CAMSH0001_0493 | 4 | 0.76 | 21369489; 21282416; 21063169; 20826163; 20580470; 20561590; 16487743; 19217396; 20410299; 20368723 |
| CAMSH0001_1355 | 19 | 0.82 | 21311024; 21244730; 21183069; 21183069; 21182591; 21177880; 21167155; 21159796; 21131496; 21129777 |
| CAMSH0001_1361 | 19 | 0.95 | 21326923; 21162957; 21057010; 20799091; 20699433; 20688825; 11386933; 20624909; 20622068; 20619088 |
| CAMSH0001_2010 | 18 | 0.90 | 21397717; 21378194; 21369825; 21365259; 21265778; 21233160; 21192796; 21183643; 21139203; 21122159 |
| CAPGI0001_0163 | 19 | 0.75 | 21357626; 21333648; 21301097; 21247902; 21148142; 21060781; 21059179; 20941418; 20931864; 20872163 |
| CAPGI0001_0640 | 9 | 0.89 | 20889131; 20605918; 20597103; 20308079; 20186809; 13671378; 19756806; 16481150; 18375835; 17901542 |
| CAPGI0001_0671 | 19 | 0.81 | 20491912; 20308541; 16736587; 12876312; 15136574 |
| CAPGI0001_0988 | 5 | 0.76 | 21330782; 21329685; 21318891; 21318872; 21219895; 21208503; 21187074; 21156160; 21152090; 21082203 |
| CAPGI0001_1313 | 18 | 0.77 | 21397556; 21382378; 21357485; 21353727; 21346155; 21331260; 21329510; 21318561; 21262962; 21245269 |
| CAPGI0001_1544 | 19 | 0.73 | 21398539; 20943180; 20880355; 2248769; 7494007; 19039666; 18547376; 15486203; 18034862; 10361306 |
| CAPGI0001_1738 | 8 | 0.78 | 21264378; 20615089; 20600873; 20438784; 20479750; 20118281; 20035319; 19160513; 942051; 19617350 |
| CAPGI0001_1877 | 8 | 0.86 | 21383078; 21082361; 21067517; 20581474; 20405931; 19845618; 7860587; 15269332 |
| CAPGI0001_2026 | 19 | 0.83 | 21167155; 20594157; 19923747; 19246764; 18096506 |
| CAPGI0001_2472 | 14 | 0.98 | 16154080; 15299374; 8836106; 8008020; 11188695; 9088991; 7000783; 2643092; 7030616 |
| CAPSP0001_0167 | 15 | 0.98 | 20480360; 20378989; 20205655; 18761686; 19246748; 18757803; 10500846; 17709334; 17294699; 12601011 |
| CAPSP0001_0946 | 8 | 0.77 | 21184730; 21167537; 21076590; 20930473; 20889231; 20695896; 20675102; 20450196; 12186604; 20210935 |
| CAPSP0001_1039 | 19 | 0.94 | 21299880; 21215963; 21097495; 21062783; 20868765; 20804758; 20685646; 20656867; 20632938; 20503105 |
| CAPSP0001_1076 | 19 | 0.90 | 20884352; 20713411; 20686915; 20681989; 20562284; 20392438; 20383018; 20206499; 20070105; 20054127 |
| CAPSP0001_1150 | 19 | 0.73 | 21215963; 21146160; 21094645; 21069723; 20872041; 20686915; 20619350; 20562312; 20547381; 11482451 |
| CAPSP0001_1460 | 18 | 0.84 | 21111787; 20621843; 20410287; 20371770; 20298201; 20146669; 19887446; 14561776; 19523112; 16321991 |
| CBU_0084 | 5 | 0.97 | 21369825; 21362161; 21318560; 20722631; 20722014; 20679209; 20036737; 19900405; 19806451; 19726673 |
| CBU_0176 | 12 | 0.99 | 21369989; 21352460; 21332448; 21329491; 21327044; 21320870; 21316765; 21315829; 21310956; 21301787 |
| CBU_0638 | 8 | 0.95 | 21253866; 21036145; 10672375; 20022530; 9324032; 17667915; 17635929; 8798399; 17303087; 16293764 |
| CBU_0640 | 8 | 0.88 | 20655923; 20416309; 20160912; 19996100; 16531404; 14742428; 14644451; 12603319; 12031470; 11955070 |
| CBU_0692 | 8 | 0.81 | 20655923; 20160912; 18398875; 17923481; 17157320; 15082001; 14992577; 14742428; 14644451; 12761172 |
| CBU_0787 | 2 | 0.87 | 21357626; 21299212; 21290549; 21191944; 21183648; 21173115; 21102595; 21068446; 21067235; 20945983 |
| CBU_1189 | 18 | 0.91 | 21385703; 21303910; 21297161; 21148420; 21147776; 21111787; 20654624; 20351179; 20332122; 20130685 |
| CBU_1689 | 18 | 0.89 | 19903372; 19711106; 19410333; 19389778; 15358235; 19027009; 19008001; 19002783; 18788527 |
| CBU_1835 | 8 | 0.90 | 21296217; 20889709; 20557447; 20369854; 20305273; 20230247; 20219383; 20101264; 20079438; 19881207 |
| CBU_1924 | 13 | 0.90 | 16245325; 18426891; 19591841; 19446527; 19407389; 18565343; 1735704; 11532950; 16403873; 16245325 |
| CBU_2045 | 19 | 0.96 | 21390509; 21307593; 21278271; 21240541; 21182289; 21138570; 20879840; 20829286; 20730758; 20724636 |
| CC_0140 | 8 | 0.91 | 21186730; 20833976; 19111879; 20433422; 20237237; 20227511; 20118250; 19781542; 19764814; 19638315 |
| CC_0168 | 12 | 0.70 | 21291858; 21212608; 21182594; 21151953; 21113270; 21111810; 21046238; 20857408; 20837138; 20807766 |
| CC_0315 | 13 | 0.79 | 10419966; 17616600; 18833940; 16428430; 10419966; 4934072; 15836769; 16518617; 11160812; 16343434 |
| CC_1405 | 8 | 0.90 | 21261469; 21210973; 21239558; 21051545; 21037180; 20934402; 20869472; 20652619; 5420057; 20511510 |
| CC_1907 | 4 | 0.78 | 18451864; 4598031; 15817382; 9168127; 7867059; 9063647; 2129533; 8299954 |
| CC_2134 | 12 | 0.93 | 18684711; 10089876; 15221451; 11940587; 11516154; 11054575; 10021343; 8626673; 9488737; 9328291 |
| CC_2183 | 3 | 0.98 | 18523167; 17468768; 16162504; 12410843; 12172800; 10400570; 8096341; 10375640; 9786181; 9282737 |
| CC_3454 | 9 | 0.96 | 21193572; 21057504; 20929961; 20816087; 20727852; 20707002; 20534481; 18476984; 20370610; 20334431 |
| CC_3569 | 8 | 0.72 | 21266406; 21033739; 20935402; 20884079; 20880712; 20820888; 20819069; 20819067; 20738256; 20728216 |
| CCA_00906 | 5 | 0.94 | 17891922; 12207230; 9503607; 8840505; 7947754; 8492805; 2664422 |
| CCC13826_0958 | 2 | 0.93 | 21206014; 21156797; 21081498; 20861021; 20690630; 20112869; 20039101; 19946146; 19479286; 19330765 |
| CCV52592_0568 | 4 | 0.83 | 21369489; 21326935; 21325038; 21306444; 21298091; 21283546; 21282416; 21199832; 21198360; 21155910 |
| CCV52592_0848 | 2 | 0.86 | 21081498; 20863064; 20861021; 20709836; 20690630; 20636266; 20219465; 20112869; 20063894; 20039101 |
| CCV52592_0927 | 17 | 0.98 | 21389150; 21378166; 21346410; 21326886; 21326359; 21310960; 21254160; 21221925; 21214942; 21159079 |
| CCV52592_1087 | 19 | 0.80 | 21377772; 21372526; 21191041; 21098931; 20826445; 20537545; 20385753; 20233306; 20118540; 20111805 |
| CCV52592_1161 | 6 | 0.96 | 16125908; 1451796; 1387640; 1386387; 1826850 |
| CFF8240_0129 | 8 | 0.94 | 19195398; 7108955; 9585000; 7628554; 3481022 |
| CFF8240_0366 | 18 | 0.97 | 20192961; 10827169; 19711123; 15069191; 16304597; 12368463; 17652430; 16968739; 14505932; 10404179 |
| CFF8240_1382 | 19 | 0.87 | 21394312; 21383503; 21366909; 21354605; 21347376; 21342283; 21327212; 21286806; 21273509; 21267505 |
| CFF8240_1521 | 18 | 0.90 | 21397717; 21378194; 21369825; 21365259; 21265778; 21233160; 21192796; 21183643; 21139203; 21122159 |
| CFF8240_1801 | 8 | 0.92 | 19923715; 19574215; 19110079; 18216065; 10725534; 17313403; 17131148; 16919403; 15362852; 15302401 |
| CHAB381_0749 | 5 | 0.90 | 21369825; 20394677; 19602058; 19195484; 18704836; 18604641; 17951579; 17949251; 17913701; 12740388 |
| CHY_0099 | 6 | 0.90 | 21193388; 21080372; 21059412; 21042723; 20949080; 20938339; 21058426; 20930037; 20920744; 20874390 |
| CHY_0621 | 2 | 0.70 | 12590921; 11325967; 9757107; 16668194; 10498704; 7815950 |
| CHY_0717 | 13 | 0.70 | 20882017; 20190074; 10587438; 17943273; 17024311; 16164567; 15748981; 15301531; 12796482; 11574542 |
| CHY_1096 | 8 | 0.82 | 21226510; 21148632; 21130735; 21080067; 20705057; 20629071; 20457604; 20435060; 20236937; 20121244 |
| CHY_1849 | 19 | 0.91 | 20976701; 20202763; 19762441; 17400891; 12397186; 18550550; 16099208; 18342249; 18314348; 17533641 |
| CHY_1889 | 12 | 0.89 | 20869404; 19888572; 19263521; 12824332; 18814301; 18095937; 17786425; 16627468; 12577270; 1704930 |
| CHY_1900 | 19 | 0.97 | 21301102; 21111546; 21040514; 21040511; 20851903; 20847002; 20843347; 20713082; 20594961; 20349278 |
| CHY_2150 | 12 | 0.91 | 21289038; 20807195; 20077115; 19619611; 12815105; 17606910; 16030141; 10051596; 18631244; 18189304 |
| CHY_2491 | 12 | 0.95 | 21401523; 21397190; 21385871; 21385438; 21383919; 21383012; 21371431; 21369989; 21368759; 21364999 |
| CJA_0371 | 18 | 0.90 | 21398536; 21299248; 21178960; 21062372; 20865003; 20583998; 20554571; 19342493; 20497225; 20488669 |
| CJA_0392 | 13 | 0.99 | 20943400; 20617848; 20601684; 19879905; 18957446; 17533454; 19481543; 18252769; 19106621; 18817520 |
| CJA_1183 | 6 | 0.91 | 21109751; 16989934; 9783263; 9609952; 8627883; 7763245; 8588247; 8485224; 1302503; 1663344 |
| CJA_1284 | 4 | 0.82 | 9603889; 19775244; 19696109; 8081742; 18243686; 6761544; 17302795; 10419957; 16089397; 16049010 |
| CJA_1632 | 6 | 0.93 | 8488562; 19527752; 18824234; 7592430; 18227240; 10099689; 17683397; 17619258; 17363056; 15708977 |
| CJA_1656 | 19 | 0.95 | 21369910; 21304822; 21290547; 21193820; 21106368; 20938774; 20813002; 20798166; 20718293; 20567817 |
| CJA_1869 | 9 | 0.97 | 20834152; 19996150; 18553138; 12364542; 17119955; 16600467; 9634230; 15927887; 14499609 |
| CJA_2203 | 4 | 0.72 | 20600507; 10592175; 16790025; 16309817; 12927080; 12521307; 12354237; 10361282; 9491412; 9258332 |
| CJA_2274 | 18 | 0.90 | 21397012; 21394083; 21392501; 21392495; 21391832; 21359551; 21353704; 21353305; 21346813; 21335528 |
| CJA_2788 | 18 | 0.88 | 21399923; 21353727; 21318561; 21265748; 21258844; 21242164; 21216906; 21191678; 21187401; 21177244 |
| CJA_2991 | 6 | 0.87 | 21402758; 21398634; 21383239; 21382109; 21372093; 21369983; 21362510; 21362508; 21354180; 21353876 |
| CJA_3323 | 12 | 0.77 | 21073315; 21037178; 20699090; 19439009; 19378128; 10503534; 19059780; 15558012; 14726457; 17466013 |
| CJE_0306 | 5 | 0.90 | 21369825; 20394677; 19602058; 19195484; 18704836; 18604641; 17951579; 17949251; 17913701; 12740388 |
| CJE_0882 | 1 | 0.82 | 21264378; 21263039; 21188077; 20973559; 20953603; 20693595; 20675481; 20656866; 20656783; 20565114 |
| CJE_1419 | 6 | 0.82 | 21383239; 21382109; 21372093; 21369983; 21362510; 21362508; 21354180; 21353876; 21347346; 21339843 |
| CJJ81176_pTet0025 | 4 | 0.76 | 20427671; 402693; 2231712; 19205798; 18205809; 10438968; 10322172; 16782401; 15358353; 10417188 |
| CLB_0585 | 8 | 0.92 | 21376015; 21068394; 20932062; 20652669; 20513347; 20159007; 19821612; 18723024; 19626710; 2294092 |
| CLB_1294 | 6 | 0.73 | 21255377; 21203403; 21123384; 21092102; 21059708; 20970942; 20929867; 20854105; 20837039; 20810535 |
| CLB_1337 | 5 | 0.97 | 21156797; 16924483; 18429691; 18096847; 17163967; 12471504; 11823455; 11781802; 9671511; 2615765 |
| CLB_1351 | 19 | 0.98 | 21388880; 21357429; 21347309; 21339299; 21327741; 21307593; 21304822; 21300542; 21282527; 21269444 |
| CLB_1932 | 8 | 0.78 | 20679207; 20597606; 19889946; 17108241; 19379783; 18455501; 17609257; 16469539; 16453288; 16377227 |
| CLB_2125 | 9 | 0.90 | 19324564; 6997270; 18400514; 17328767; 17249420; 9634230; 14708602 |
| CLB_2231 | 6 | 0.84 | 21301859; 21245343; 21228759; 21173259; 21107010; 21087930; 21044871; 20950625; 20929870; 20854105 |
| CLB_2520 | 8 | 0.75 | 20298739; 20155950; 20124708; 19053231; 20013028; 19266512; 7765483; 17406963; 17876515; 17406963 |
| CLB_2593 | 4 | 0.80 | 21091499; 20889747; 20886115; 20586063; 16487743; 20471399; 20451645; 20444091; 20439729; 20427320 |
| CLB_2622 | 19 | 0.77 | 21378188; 21081498; 20385136; 19836342; 19783652; 19684306; 19666032; 12401806; 11038364; 12220406 |
| CLB_3673 | 8 | 0.85 | 17217963; 15026039; 11120357; 10679197; 8055948; 8349682; 1840590 |
| CLC_0953 | 4 | 0.75 | 21390130; 21357426; 21319192; 21295525; 21291275; 21289174; 21266713; 21245169; 21235489; 21221131 |
| CLC_2143 | 5 | 0.76 | 21231969; 21212358; 21038112; 20843032; 20709836; 20429505; 20411554; 20221547; 20221528; 20221527 |
| CLC_2781 | 8 | 0.92 | 20609914; 19549597; 18991392; 18981569; 18930846; 18211101; 12936980; 17385893; 2172928; 5700707 |
| CLD_0046 | 8 | 0.90 | 21387033; 21360829; 21340633; 21338421; 21333632; 21332407; 21323543; 21322492; 21320074; 21296885 |
| CLD_1688 | 19 | 0.88 | 21400235; 21388958; 21378160; 21367655; 21357626; 21342516; 21333648; 21332624; 21331044; 21307593 |
| CLI_2425 | 6 | 0.83 | 21303910; 21146514; 21113140; 20351180; 20179009; 18602413; 20110566; 19878744; 17065615; 19573535 |
| CLJ_0235 | 19 | 0.93 | 2248769; 18547376; 15486203; 17153919; 12196390; 16391697; 15470114; 15255185; 14758475; 12791139 |
| CLJ_0248 | 6 | 0.93 | 21214869; 20186688; 3217442; 15750208; 18414490; 19039682; 1791753; 18561205; 17932351; 17542913 |
| CLJ_B0320 | 8 | 0.70 | 21383138; 21349748; 21345800; 21327819; 21259411; 21229992; 21210640; 21205212; 21205174; 21148731 |
| CLJ_B0516 | 19 | 0.75 | 21097580; 20668486; 20642807; 18818215; 20127467; 20118250; 17888883; 11069242; 19093149; 19025571 |
| CLJ_B0581 | 19 | 0.74 | 21097580; 21062783; 20668486; 20642807; 18818215; 20127467; 20118250; 18236038; 17888883; 19422893 |
| CLJ_B0661 | 13 | 0.99 | 21278229; 21151095; 21124459; 20972222; 20954242; 20890638; 20696925; 20617848; 20566627; 20452364 |
| CLJ_B1237 | 8 | 0.77 | 16611136; 16600650; 9827558; 8531024; 7727530 |
| CLJ_B2050 | 19 | 0.81 | 21327035; 21241420; 20971910; 20971903; 20497501; 20497501; 20487285; 20199110; 20059692; 19348786 |
| CLJ_B2342 | 15 | 0.90 | 1906870; 19959660; 17935691; 18205812; 17054778; 9286980; 3862129; 3403505; 8282725; 12175495 |
| CLJ_B2541 | 12 | 0.96 | 21347827; 21320870; 21315771; 21278271; 21259244; 21235526; 21233422; 21222627; 21216232; 21205794 |
| CLJ_B2734 | 15 | 0.87 | 21397844; 21385202; 21364902; 21347487; 21318274; 21315771; 21300840; 21284862; 21283542; 21283517 |
| CLJ_B2813 | 9 | 0.95 | 21035724; 20305019; 19766092; 19522564; 15014080; 11038526; 12237320; 3289117; 9244288; 2814500 |
| CLJ_B2881 | 4 | 0.78 | 18753783; 15817382; 10712687; 7601828; 10536136; 2407720; 2002011; 1999391; 8415608; 3155514 |
| CLJ_B2983 | 1 | 0.73 | 20180123; 19471856; 19402116; 7901896; 18481057; 18210886; 18007551; 17589636; 17362731; 17139593 |
| CLJ_B3084 | 8 | 0.74 | 21393246; 21322495; 21322493; 21322492; 21167156; 21138528; 21094149; 21075841; 21073881; 21068394 |
| CLL_A0091 | 4 | 0.85 | 15699190; 786255; 13999018; 15470121; 10198031; 12662922; 12624213; 1729247; 9353931; 8755863 |
| CLL_A0670 | 4 | 0.70 | 20507997; 19770041; 19695338; 19135125; 17270289; 10940244; 12824352; 11344133; 9520405; 15695811 |
| CLL_A0880 | 18 | 0.83 | 21393072; 21379326; 21357745; 21385703; 21328529; 21325636; 21322032; 21314926; 21310790; 21303910 |
| CLL_A1044 | 8 | 0.79 | 388439; 9179845; 3323848; 2981635; 6094487; 127785; 6282417; 6792201; 5809587 |
| CLL_A1307 | 18 | 0.82 | 17306760; 10377394; 12627883; 12582158; 12427139; 10201006; 12133831; 12098491; 11165372; 10825438 |
| CLL_A2334 | 2 | 0.86 | 18163882; 18043855; 11795479; 11535779; 11435118; 11419946; 2271518; 1737042 |
| CLL_A2524 | 18 | 0.92 | 9586241; 9641984; 8132345; 9245444; 12393194; 8161535; 8349639; 1401036; 1848243; 2684986 |
| CLL_A3272 | 2 | 0.94 | 21342411; 21288652; 21286325; 21268892; 21117170; 21073422; 20968298; 20956531; 20817725; 20739284 |
| CLL_A3436 | 18 | 0.85 | 21393841; 20581474; 20038140; 20026078; 18691556; 17803910; 18321243; 18227270; 14521953; 12167852 |
| CORAM0001_0083 | 8 | 0.73 | 21358191; 21354629; 21318381; 21307286; 21268708; 21262951; 21238431; 21106325; 21083040; 21082267 |
| CORAM0001_0133 | 15 | 0.99 | 21364902; 21320584; 21315771; 21295603; 21239493; 21216996; 21216906; 21193607; 21183673; 21178479 |
| CORAM0001_0270 | 4 | 0.84 | 20979333; 20545868; 20020285; 19954209; 17298391; 19016879; 19016840; 17926093; 11731481; 10708368 |
| CORAM0001_0309 | 8 | 0.87 | 21199936; 21090815; 21082862; 21081474; 21057456; 21046342; 21038895; 21030914; 20937819; 20934416 |
| CORAM0001_0396 | 19 | 0.70 | 19538395; 19502509; 18461320; 3335502; 16096274; 15280033; 15159628; 14684172; 14518977; 10517585 |
| CORAM0001_1331 | 8 | 0.93 | 20858453; 20837795; 20826797; 20818589; 20654728; 20636270; 20627642; 20528950; 20505714; 20505246 |
| CORAM0001_1374 | 19 | 0.91 | 20971878; 20709844; 20656867; 20622122; 20562284; 20511418; 20382819; 20382811; 20348303; 20228117 |
| CORAM0001_1546 | 9 | 0.94 | 21041072; 20975997; 20889131; 20709688; 20605918; 20599697; 20597103; 20308079; 20219900; 20186809 |
| CORAM0001_1664 | 15 | 0.98 | 21320584; 21315771; 21295603; 21239493; 21216996; 21216906; 21193607; 21183673; 21178479; 21173175 |
| CORAM0001_1872 | 18 | 0.78 | 20581474; 20038140; 7860587; 14521953; 17562318; 16275737; 11592120; 10493532; 2404009; 9614122 |
| CORAM0001_1945 | 9 | 0.92 | 21135213; 20648527; 20355329; 1355089; 18079405; 19603180; 19560433; 15572765; 19263251; 10913250 |
| CORMA0001_0016 | 12 | 0.76 | 10777535; 10436088; 2963963; 1944231; 6270337 |
| CORMA0001_0381 | 8 | 0.82 | 19409596; 19274741; 19038292; 19020988; 18845259; 18557388; 17483518; 17927979; 17891475; 16781452 |
| CORMA0001_1264 | 2 | 0.86 | 20667822; 20218726; 19460095; 18983854; 18163882; 17372673; 7852378; 10438743; 11327837; 6379600 |
| CORMA0001_2380 | 15 | 0.98 | 21320584; 21315771; 21295603; 21239493; 21216996; 21216906; 21193607; 21183673; 21178479; 21173175 |
| CORMA0001_2444 | 15 | 0.98 | 21183673; 20870771; 20855615; 20639324; 20521955; 20093290; 6550579; 7010115; 12614149; 15980437 |
| CORMA0001_2662 | 12 | 0.71 | 21266780; 21292960; 21143848; 21143527; 21135213; 21106943; 21073735; 21062737; 21044074; 21041661 |
| CORTU0001_0221 | 12 | 0.88 | 20852022; 20799957; 20696265; 20645085; 20616713; 20522495; 20510344; 20496585; 20457124; 16086015 |
| CORTU0001_1858 | 8 | 0.74 | 21138528; 21029451; 20889970; 20705057; 20635418; 20592025; 20502966; 20405215; 19908864; 10766431 |
| CORTU0001_1892 | 4 | 0.82 | 20502438; 20453092; 19552710; 19552709; 1628843; 10400593 |
| COXBU7E912_0698 | 5 | 0.87 | 21384112; 21357626; 21310492; 20545351; 20506478; 20429582; 20129923; 20118238; 20036737; 20026605 |
| COXBU7E912_1480 | 8 | 0.81 | 21299470; 21235502; 21190518; 21078302; 21044985; 20960080; 20929858; 20862491; 20833709; 20682242 |
| COXBU7E912_1824 | 8 | 0.78 | 21343423; 21329681; 21185310; 21078123; 21073854; 20931090; 20833539; 20809899; 20806931; 20736169 |
| CP_0130 | 13 | 0.85 | 20554247; 20353943; 20127467; 16245325; 17645794; 18426891; 19591841; 19446527; 15358762; 19407389 |
| CP_0320 | 8 | 0.87 | 21209092; 20516622; 20453144; 20375021; 20364333; 18086808; 18469270; 18941301; 18845259; 9398220 |
| CP_0902 | 9 | 0.71 | 19539490; 7961456; 17726007; 17014493; 16524713; 16243729; 15926258; 9618564; 10924502; 9748349 |
| CP_0972 | 8 | 0.74 | 20656472; 9525901; 15896461; 11102521; 9151972; 8473316; 1393838; 3485632 |
| CPF_0408 | 18 | 0.85 | 21343426; 20674666; 19567876; 1946465; 19816142; 17950313; 18599498; 16162665; 17710533; 8300209 |
| CPF_0631 | 18 | 0.83 | 21368759; 21385703; 21303910; 21297161; 21249125; 21182296; 21148420; 21147776; 21111787; 21039781 |
| CPF_0753 | 15 | 0.97 | 21402151; 21401077; 21398634; 21396120; 21393212; 21386983; 21385724; 21383239; 21381030; 21379585 |
| CPF_1073 | 12 | 0.91 | 21356365; 21344393; 21335558; 21289093; 21288331; 21199726; 21196491; 21188449; 21252992; 21075784 |
| CPF_2301 | 4 | 0.87 | 21346797; 21339285; 21284980; 21272569; 21255377; 21204254; 21190525; 21182296; 21178952 |
| CPF_2406 | 18 | 0.90 | 21398536; 21350490; 21350473; 21349348; 21337355; 21333939; 21322478; 21308846; 21299248; 21268857 |
| CPF_2841 | 6 | 0.85 | 21035733; 19217392; 10545127; 10564466; 15837199; 14985111; 10369758; 11301257; 11137007; 10717390 |
| CPF_2852 | 18 | 0.89 | 20106928; 16230336; 12060404; 11320094; 11257260; 9878413; 9092582 |
| CPR_1064 | 12 | 0.87 | 16290006; 19553544; 18594935; 18294721; 17646170; 17475206; 15823018; 15713660; 15147205; 14512418 |
| CPR_1266 | 15 | 0.92 | 21131084; 20968238; 20938090; 20825427; 20626716; 20560130; 20493201; 20445005; 20430001; 20113547 |
| CPS_0523 | 19 | 0.88 | 21400235; 21388958; 21378160; 21367655; 21357626; 21342516; 21333648; 21332624; 21331044; 21307593 |
| CPS_0688 | 2 | 0.91 | 21367973; 20968298; 14675766; 12651851; 8144536; 8380721; 2187098 |
| CPS_1187 | 9 | 0.82 | 21385872; 21369973; 21295455; 21279344; 21161801; 21131515; 21114629; 21108127; 21084450; 21081692 |
| CPS_2656 | 19 | 0.86 | 20708437; 20643653; 20384696; 19493008; 19400771; 11371196; 19170887; 12192412; 17991683; 18506095 |
| CPS_2838 | 18 | 0.93 | 21089447; 19903372; 19711106; 19410333; 19389778; 15358235; 19027009; 19008001; 19002783; 18788527 |
| CPS_2946 | 6 | 0.89 | 21349825; 21327070; 21303910; 21149390; 21146514; 21113140; 21081662; 21061633; 20688172; 20674516 |
| CPS_3252 | 19 | 0.95 | 11443096; 11487696; 11270405; 12761115; 8717524; 12464616; 9266683; 334154; 640738; 2493441 |
| CPS_3627 | 19 | 0.90 | 20981454; 20735851; 6808412; 20552686; 20538863; 20435741; 20189107; 20175955; 20106978; 20086163 |
| CPS_3870 | 8 | 0.85 | 20030377; 19959582; 19368556; 18298089; 17203313; 16962969; 15485884; 10518782; 12605683; 11951047 |
| CPS_4233 | 2 | 0.87 | 19878958; 8910497; 19392660; 15597200; 15356000 |
| CPS_4237 | 6 | 0.84 | 21170359; 21111748; 21059676; 20826341; 20739603; 20711169; 20705653; 19182789; 20639533; 20600238 |
| CPS_4482 | 12 | 0.71 | 21367852; 21364604; 21350558; 21333377; 21319795; 21245196; 21245169; 21213249; 21209419; 21203533 |
| CSE45_0089 | 2 | 0.93 | 21190337; 21120472; 21081498; 20861021; 20690630; 20666399; 20112869; 20040598; 20039101; 20030377 |
| CSE45_0129 | 6 | 0.91 | 21245167; 21350155; 21126517; 20875449; 20705645; 11071951; 20192920; 20118257; 19234526; 1830580 |
| CSE45_0944 | 18 | 0.82 | 21232206; 20678811; 20554850; 20667930; 20375246; 20167774; 20038587; 20036857; 18079405; 19643810 |
| CSE45_0967 | 18 | 0.83 | 21256134; 21228468; 21117233; 21069910; 20920237; 20870776; 20813447; 20693671; 20675494; 20487285 |
| CSE45_1006 | 8 | 0.81 | 18396129; 18061151; 16870178; 16788776; 15559763; 15317750; 15300772; 15115179; 15056672; 14740892 |
| CSE45_1035 | 12 | 0.89 | 21270253; 21268308; 21258846; 21194485; 21177833; 21060781; 21055618; 20964795; 20940419; 20920545 |
| CSE45_1055 | 12 | 0.85 | 19853572; 17305560; 16573693; 15882410; 15037243; 12214059; 2408275; 8106505 |
| CSE45_1902 | 8 | 0.86 | 21073854; 20515113; 19428350; 19177216; 19140015; 19011745; 18850316; 18422649; 17516063; 17203966 |
| CSE45_2097 | 12 | 0.71 | 20574001; 19825830; 19254684; 18399917; 18260106; 9757107; 10500301; 16318855; 10600770; 12684048 |
| CSE45_2112 | 2 | 0.88 | 18156677; 19013460; 11795479; 6752142; 1737042 |
| CSE45_2289 | 8 | 0.83 | 19454319; 19083092; 18599075; 18028427; 17350870; 15914052; 15720386; 15565272; 15211513; 9389475 |
| CSE45_2345 | 8 | 0.78 | 20562284; 19888806; 19336532; 19184767; 19117726; 18318839; 17876960; 16874542; 16698295; 16368748 |
| CSE45_2404 | 8 | 0.75 | 2142875; 19118407; 7181513; 10091591; 14216418 |
| CSE45_2426 | 8 | 0.91 | 21393249; 15069080; 11866469; 10876657; 10444331; 9839943; 9590030; 1710985; 13694597; 321333 |
| CSE45_2445 | 12 | 0.81 | 20007811; 15272232; 14502125; 9512662; 7871388; 1388724 |
| CSE45_2514 | 19 | 0.96 | 21030539; 20824214; 20595206; 20707404; 20201406; 19883124; 19825675; 19414810; 19298858; 9736698 |
| CSE45_2526 | 6 | 0.84 | 21242293; 21211720; 21143968; 20973964; 20952393; 20929865; 20864450; 20813592; 20713449; 20733069 |
| CSE45_2625 | 12 | 0.78 | 21291857; 21187289; 21040728; 20947360; 20806075; 20643210; 20493688; 20452224; 20417280; 15968468 |
| CSE45_2650 | 18 | 0.72 | 20624215; 20154147; 20083401; 19887446; 18656546; 19748349; 19619244; 19351326; 19143621; 16030141 |
| CSE45_2707 | 9 | 0.86 | 21397712; 21145868; 20881054; 20398751; 19564404; 19716469; 19575662; 19207211; 19029290; 18450749 |
| CSE45_2741 | 19 | 0.73 | 21342516; 21046402; 20971878; 20619350; 20504027; 20503105; 20227065; 19919544; 19784808; 19652374 |
| CSE45_2946 | 8 | 0.77 | 21138528; 20889970; 20502966; 20405215; 19908864; 10766431; 16603772; 18957412; 18421771; 18160405 |
| CSE45_3043 | 8 | 0.81 | 10775262; 18061151; 16526093; 16218961; 16023078; 15317750; 15056672; 15004020; 14727190; 12657050 |
| CSE45_3272 | 2 | 0.93 | 21190337; 21120472; 21081498; 20861021; 20690630; 20666399; 20112869; 20040598; 20039101; 20030377 |
| CSE45_3304 | 8 | 0.71 | 20405215; 19908864; 18957412; 18160405; 15581577; 10852721; 10368269; 9490068; 1529349 |
| CSE45_3366 | 8 | 0.80 | 19659575; 18368854; 17259611; 8702795; 15946826; 10400577; 1787796; 9493364; 7836293; 8205477 |
| CSE45_3859 | 1 | 0.93 | 18481057; 17575227; 17135341; 15803651; 16840914; 2610349; 16428494; 14742742; 12939586; 10684605 |
| CSE45_4586 | 8 | 0.92 | 20956531; 20449117; 20015072; 19894084; 10700270; 19397993; 19027887; 18755150; 18248595; 17906130 |
| CSE45_4738 | 8 | 0.78 | 19943898; 12892110; 18192696; 17547421; 16995898; 16817906; 16228571; 15848194; 14645228; 12196153 |
| CSE45_4835 | 15 | 0.84 | 18393619; 8922118; 10748066; 15528657; 15165250; 2824447; 14638413; 10727938; 10792733; 11310734 |
| CSE45_5057 | 12 | 0.72 | 21398603; 21397717; 21376116; 21375730; 21364925; 21354366; 21354180; 21349976; 21343359; 21342099 |
| CSE45_5342 | 8 | 0.95 | 21167813; 16274230; 12409197; 12165429; 9546395; 9756865; 7746153; 1657982; 16345624 |
| CSE45_5429 | 4 | 0.71 | 20507997; 20117010; 19770041; 19695338; 19135125; 15695811; 18005663; 17270289; 10940244; 1638630 |
| CSE45_5431 | 4 | 0.78 | 18688112; 18198179; 16904399; 12393925; 16542815; 10536008; 14527802; 12761216; 10569923; 11914371 |
| CSE45_5455 | 5 | 0.89 | 21357626; 21089447; 21043483; 20692275; 20394677; 19995333; 19937589; 7926754; 19571171; 19551860 |
| CT_1923 | 4 | 0.80 | 11325945; 15769466; 1846149; 11298275; 1832208 |
| CT_2006 | 4 | 0.82 | 21383142; 21368902; 21368045; 21347721; 21347234; 21346797; 21339285; 21339284; 21330432; 21326934 |
| CYA_1029 | 8 | 0.87 | 21167158; 21075841; 20944229; 20643958; 20558746; 20405215; 19908864; 17850165; 18957412; 19102705 |
| CYA_1819 | 5 | 0.98 | 19334767; 18314963; 2037604; 15485884; 9514861; 2836706; 2615765; 2643516; 3062178; 2826155 |
| CYA_2629 | 19 | 0.87 | 21256135; 21206027; 21126522; 20962348; 20954236; 20932833; 20883454; 20865528; 20798166; 20724227 |
| CYB_1554 | 8 | 0.72 | 21329667; 21073414; 20861021; 20334431; 19679655; 19624733; 16817897; 18421771; 18154320; 17074519 |
| CYB_1596 | 18 | 0.89 | 21314091; 21187068; 21135135; 20616256; 19403823; 19915095; 19548526; 19540251; 19323989; 19187093 |
| CYB_1827 | 2 | 0.88 | 21081498; 20861021; 20690630; 20112869; 20039101; 19330765; 19122276; 18716757; 16407262; 18522945 |
| CYB_2441 | 8 | 0.97 | 20466541; 20124701; 19395484; 19744988; 1445195; 19184529; 16665774; 12107140; 10772893; 18323627 |
| CYB_2593 | 2 | 0.91 | 21206014; 21081498; 20861021; 20709836; 20690630; 20378651; 20219465; 20112869; 20063894; 19946146 |
| DET_0071 | 6 | 0.77 | 9305914; 7590261; 16298340; 14990586; 10970884; 11031099; 10910076; 8371988; 9748214; 9614128 |
| DET_0769 | 12 | 0.91 | 21397065; 21391917; 21387310; 21385871; 21383919; 21383012; 21375706; 21372759; 21372510; 21368759 |
| DET_1111 | 18 | 0.78 | 21357396; 21336990; 21315718; 21315686; 21311724; 21221131; 21177413; 21168419; 21148206; 21122131 |
| DNO_0020 | 8 | 0.95 | 21272045; 20406696; 20352460; 20091071; 20002867; 19807880; 19778366; 19597319; 19582719; 19523599 |
| DNO_0060 | 13 | 0.97 | 10937989; 9294008; 9044258; 8722036; 354966; 1742360; 173425; 2665813; 2653827; 3884043 |
| DNO_0136 | 18 | 0.99 | 20516589; 17206386; 15901729; 16718600; 15901729; 2055470 |
| DNO_0367 | 8 | 0.73 | 21091201; 20514241; 19011745; 12003933; 18422649; 17350704; 15680231; 14965227; 12974644; 12388585 |
| DNO_0396 | 18 | 0.78 | 21204798; 20974900; 20826674; 20676104; 20709936; 20707984; 20683865; 20650859; 20576798; 20547976 |
| DNO_0398 | 18 | 0.94 | 21360409; 21348297; 21347827; 21217751; 21206157; 21184741; 21175590; 21173549; 21130455; 21106767 |
| DNO_0399 | 18 | 0.77 | 21393072; 21392379; 21383919; 21368759; 21366672; 21360512; 21360409; 21357396; 21348297; 21347827 |
| DNO_0423 | 13 | 0.85 | 21068099; 8619477; 18926798; 17980389; 2011739; 9857203; 16962594; 15994936; 14970222; 12559567 |
| DNO_0494 | 18 | 0.80 | 21321143; 21130927; 20952578; 20836765; 20665474; 20533493; 20450883; 20093292; 19929574; 17054063 |
| DNO_0525 | 13 | 1.00 | 20400559; 18037435; 12368106; 8628231; 8722036; 8504167 |
| DNO_0526 | 13 | 0.99 | 20132446; 15100988; 12068810; 9419243; 8980544; 8725011; 7739034; 8207023; 1875917; 2116407 |
| DNO_0545 | 8 | 0.91 | 21253866; 21036145; 10672375; 20160912; 20022530; 19594830; 19464573; 19383527; 17532339; 9324032 |
| DNO_0552 | 8 | 0.96 | 21271858; 21247899; 21229706; 21183445; 21179059; 21143473; 21071057; 21064266; 20960122; 20952576 |
| DNO_0557 | 4 | 0.83 | 21209210; 19915636; 19016754; 16606633; 16213196; 15917183; 15630518; 12804011; 12605684; 12601812 |
| DNO_0697 | 12 | 0.80 | 20819954; 20610394; 19734688; 18550540; 18629030; 17916556; 17362992; 17210228; 17121880; 16584181 |
| DNO_0745 | 11 | 0.71 | 21071053; 20973964; 15518819; 10729119; 12374837; 13677051; 12706095; 2988951; 11827470; 11501993 |
| DNO_0763 | 6 | 0.96 | 21398521; 21389352; 21351580; 21349825; 21312235; 21280222; 21265901; 21241813; 21241729; 21239155 |
| DNO_0798 | 2 | 0.79 | 20955688; 20593767; 20560533; 11180061; 19559030; 19433215; 10961912; 19129660; 19055484; 16666457 |
| DNO_0819 | 15 | 0.96 | 18573169; 16385057; 18031348; 16979633; 16430694; 12867461; 10197994; 12777072; 11101667; 10627039 |
| DNO_0830 | 9 | 0.95 | 20846931; 20836570; 20470824; 20110695; 20047909; 19695650; 19686777; 7551052; 19549600; 19170545 |
| DNO_0834 | 2 | 0.88 | 21236255; 21099135; 20822158; 20460376; 20097860; 19946146; 17350958; 17350000; 11578927; 16455656 |
| DNO_0851 | 4 | 0.84 | 15819627; 15735307; 12791143; 12777805; 10931298 |
| DNO_0865 | 17 | 0.96 | 21126315; 20382767; 768986; 17234211; 15554979; 15554978; 12181321; 9933592; 9642084; 8610017 |
| DNO_0890 | 3 | 0.99 | 17985115; 17468768; 17482513; 15100690; 9159491; 1683004; 8550520; 7642131; 1588814 |
| DNO_0900 | 15 | 0.94 | 21320584; 21317318; 21315771; 21295603; 21284862; 21239493; 21217003; 21216996; 21216906; 21193607 |
| DNO_0945 | 2 | 0.76 | 21301101; 21220764; 21030587; 20685834; 20672281; 20209896; 20051244; 12068013; 19103300; 19101474 |
| DNO_1002 | 9 | 0.94 | 21041072; 20975997; 20889131; 20709688; 20605918; 20599697; 20597103; 20308079; 20219900; 20186809 |
| DNO_1007 | 13 | 0.97 | 19646947; 7731806; 18318121; 17850309; 17022624; 12003491; 15544333; 11014182; 10997906; 8730873 |
| DNO_1088 | 18 | 0.91 | 18341998; 12890666; 6329717; 11863361; 11585831; 11250194; 10491184; 10398342; 1830924; 2138512 |
| DNO_1157 | 18 | 0.74 | 21360476; 21160910; 21047280; 20949524; 20441802; 14651849; 20427472; 20395263; 20219227; 20217429 |
| DNO_1165 | 6 | 0.88 | 18007608; 10600744; 12667053; 11937058; 10778854; 10669596; 6325431 |
| DNO_1263 | 13 | 0.97 | 9757107; 10937989; 8174557; 2664451; 6178424 |
| DNO_1266 | 13 | 0.98 | 10561594; 7916699; 1772592; 2438658; 3926498 |
| DNO_1284 | 13 | 0.97 | 20399793; 9169555; 15989950; 10805779; 10747797; 8123703; 1764524; 6360687; 7049235; 6125208 |
| DNO_1310 | 4 | 0.75 | 21330432; 14960717; 10640603; 9224882; 8998979; 8830266; 8196543 |
| DR_0629 | 4 | 0.78 | 10572301; 14659047; 11591668; 9428770; 16535483 |
| DR_0646 | 13 | 0.90 | 21354313; 21330151; 21240995; 20604745; 20524863; 20434458; 20430825; 20350999; 20188109; 20088793 |
| DR_1191 | 17 | 0.90 | 10551881; 14592988; 16514143; 10371038; 11727826; 7688127; 7568114 |
| DR_1751 | 17 | 0.70 | 18952495; 16787914; 15723801; 15342628; 9850147; 1429873; 1984662; 3045116; 3021184; 14104603 |
| DR_1855 | 4 | 0.80 | 11325945; 15769466; 1846149; 11298275; 1832208 |
| DR_1988 | 8 | 0.76 | 20662890; 19691502; 8282725; 10871608; 12762842 |
| DR_2094 | 15 | 0.91 | 20577782; 9838063; 3148836; 17293416; 17968423; 18974048; 16428390; 17310401; 12142437; 15458412 |
| DR_2248 | 12 | 0.96 | 20415690; 19748062; 18973761; 16704418; 15786906; 15770466; 11282022; 11173531; 7999781; 7915920 |
| DR_2389 | 19 | 0.94 | 21397493; 21342541; 21367879; 21349845; 21307941; 21297907; 21295693; 21221632; 21182826 |
| DR_2567 | 19 | 0.93 | 20586063; 8662184; 20127467; 20036252; 19389766; 19025870; 15973422; 18440165; 18338579; 17957108 |
| DVU_0171 | 4 | 0.73 | 21375706; 20847516; 20636974; 20528175; 20376562; 2592553; 19106014; 19103467; 18156272; 18620975 |
| DVU_2330 | 18 | 0.95 | 21291474; 20712617; 20645920; 20627997; 20846098; 20601550; 20426950; 20231161; 6501214; 20103563 |
| DVU_2475 | 8 | 0.77 | 18452539; 18408355; 18088303; 16388583; 15928858; 15726348; 15668249; 12925912; 11307944; 11231268 |
| DVU_2548 | 9 | 0.92 | 20064615; 20047918; 19900769; 17904577; 17198382; 16107329; 11583992; 9305965; 3549716; 14320488 |
| DVU_2563 | 9 | 0.90 | 20192828; 3537305; 19550039; 19427006; 19370064; 14499468; 18381272; 17906131; 16213523; 15803418 |
| DVU_A0074 | 5 | 0.88 | 21357626; 21339297; 21156230; 20816216; 20798316; 20602915; 20601060; 20460947; 20429582; 20391625 |
| EcE24377A_2937 | 19 | 0.95 | 21294843; 21104698; 20871989; 20669918; 20552260; 12175810; 20522493; 20444687; 20442679; 18760333 |
| EcE24377A_4262 | 18 | 0.84 | 21111787; 20621843; 20410287; 20371770; 20298201; 20146669; 19887446; 14561776; 19523112; 16321991 |
| EcE24377A_4411 | 9 | 0.98 | 21199673; 20304931; 19801377; 10593897; 16921353; 12239217; 11535054; 11404016; 9857025; 8226759 |
| EcE24377A_4768 | 9 | 0.74 | 21388423; 21378393; 21375722; 21375706; 21375498; 21337322; 21318289; 21310443; 21307094; 21296915 |
| ECH_0063 | 8 | 0.72 | 20826797; 19766092; 10716729; 10824085; 7287745; 18246068; 17977822; 17601793; 17583799; 17292333 |
| ECH_0184 | 8 | 0.88 | 20074573; 19959836; 18614015; 18690890; 16982728; 18054490; 17964535; 17936786; 17521330; 16979134 |
| ECH74115_0331 | 2 | 0.86 | 20861021; 20690630; 19122276; 18006324; 7876088; 7815950; 8010978; 2628174 |
| ECH74115_0550 | 15 | 0.78 | 12730182; 15009896; 18001134; 16891058; 271968; 9683479; 1658572; 8836921; 388439; 7934907 |
| ECH74115_1086 | 19 | 0.94 | 21111546; 21040514; 21040511; 20851903; 20594961; 20497501; 20345654; 18818215; 20199110; 20173067 |
| ECH74115_2507 | 15 | 0.70 | 21386087; 21372178; 21367753; 21364651; 21362065; 21351730; 21331044; 21327630; 21326202; 21320329 |
| ECH74115_3302 | 8 | 0.70 | 20599671; 20511298; 18346472; 15210349; 12909015; 12147680; 11900549; 11563694; 3011794; 8145647 |
| ECH74115_3562 | 11 | 0.96 | 12441108; 11470529; 18623024; 7926832; 3516881; 8163171; 8460474; 1433286; 1838127; 2143557 |
| ECH74115_4150 | 15 | 0.81 | 16762026; 12791847; 9719000; 2556400; 2944600; 2525131; 2844626 |
| ECH74115_4401 | 8 | 0.88 | 21384861; 21370283; 21362632; 21289251; 21285336; 21275244; 21220430; 21193039; 21187465; 21130106 |
| ECH74115_4476 | 9 | 0.91 | 21397712; 21145868; 20925360; 20881054; 20690035; 20543850; 20444975; 20398751; 20363355; 20213544 |
| ECH74115_5326 | 8 | 0.79 | 18188553; 18063573; 15060078; 16348073; 12188192; 3156376 |
| ECH74115_B0092 | 6 | 0.78 | 20709099; 19640422; 19399991; 15876366; 17932625; 15479795; 17331555; 14756795; 16930703; 11428897 |
| EcSMS35_4211 | 18 | 0.87 | 21398536; 21397717; 21396452; 21390217; 21381205; 21369825; 21355897; 21350490; 21347851; 21338480 |
| EcSMS35_A0006 | 6 | 0.91 | 18035357; 7559580; 14508492; 9382804; 1610820; 8939893; 2334423; 3816761 |
| EF_0259 | 13 | 0.94 | 19616559; 10093218; 15078091; 11727977; 10093218; 9353189; 8858155; 8553584; 8722015; 7559430 |
| EF_0759 | 12 | 0.80 | 19911130; 16925552; 12453210; 9752999; 8050611; 8034015 |
| EF_1334 | 15 | 0.80 | 19708918; 18335939; 1658572; 15734745; 9631538 |
| EF_1649 | 13 | 0.85 | 20547974; 20008464; 16245325; 18426891; 19591841; 19446527; 19407389; 18565343; 1735704; 17652764 |
| EF_2452 | 6 | 0.91 | 21193388; 21080372; 21042723; 20949080; 20938339; 21058426; 20930037; 20920744; 20874390; 20864530 |
| EF_2655 | 2 | 0.87 | 19902973; 19683078; 15530362; 15530362; 15014152; 14686929; 12906818; 12140293; 11923312; 11278255 |
| EF_2933 | 6 | 0.82 | 21383239; 21382109; 21372093; 21369983; 21362510; 21362508; 21354180; 21353876; 21347346; 21339843 |
| EF_3028 | 13 | 0.98 | 20696925; 18258260; 17674047; 15078106; 8534844; 6230156; 2016746; 1828448; 3323524 |
| EF_3060 | 9 | 0.82 | 21385872; 20979348; 20799957; 15888936; 20192961; 16349198; 17428787; 19005622; 18705871; 14617657 |
| EF_3317 | 8 | 0.77 | 21365755; 21263039; 21224340; 21221530; 21117708; 21053013; 20937141; 20879029; 20803137; 20693323 |
| ENHAE0001_0045 | 4 | 0.90 | 21399666; 21330526; 21324671; 21321206; 21219460; 21172651; 21151113; 21117236; 21111234; 21124307 |
| ENHAE0001_0070 | 12 | 0.99 | 21368222; 21323990; 21303407; 21251800; 21221694; 21167902; 21042721; 14510993; 20703245; 20676769 |
| ENHAE0001_0705 | 8 | 0.80 | 21378188; 21356514; 21346153; 21333632; 21332878; 21306992; 21305032; 21296885; 21291520; 21291258 |
| ENHAE0001_0728 | 18 | 0.91 | 19469713; 17592720; 12960168; 10950931; 10748025 |
| ENHAE0001_1027 | 1 | 0.91 | 9398223; 6370397; 17113026; 16618097; 9398304; 16083849; 12032090; 3023184; 2988449 |
| ENHAE0001_1379 | 8 | 0.82 | 21310683; 21152904; 21139018; 21075928; 21051570; 21035307; 20958965; 20561692; 20555380; 15599400 |
| ENHAE0001_1900 | 8 | 0.90 | 20361926; 19904831; 19817865; 12893945; 15466509; 18440023; 18426211; 18421771; 17331467; 17244482 |
| ENHAE0001_2239 | 19 | 0.97 | 20707404; 19298858; 18499663; 18210176; 16829524; 16794327; 15820665; 15555940; 15552059; 15226299 |
| ENHAE0001_2253 | 8 | 0.92 | 21399817; 21399655; 21397646; 21397009; 21396649; 21396439; 21396131; 21394845; 21394802; 21394293 |
| ENTFA0001_0267 | 9 | 0.89 | 20708515; 20685329; 20669373; 19107742; 16972177; 16236283; 15304746; 14580194; 12451678; 10759845 |
| ENTFA0001_1641 | 18 | 0.88 | 21371898; 21369825; 21310270; 21308399; 21170876; 21261641; 21256461; 21247492; 21245145; 21238925 |
| ENTFA0001_1797 | 8 | 0.93 | 21252517; 21131394; 21063115; 21062895; 20971862; 20880984; 20880213; 20811656; 20809899; 20804650 |
| ENTFA0001_2378 | 19 | 0.84 | 21290547; 21193828; 21193820; 21184415; 20813002; 20720016; 20718293; 20639136; 20467831; 20372022 |
| ENTFA0001_2531 | 18 | 0.75 | 21356448; 21296133; 21287352; 21284755; 21276774; 21238591; 21226776; 21223646; 21218510; 21209185 |
| FSU0488 | 17 | 0.96 | 21316373; 20952386; 20595001; 20553390; 20025658; 20070531; 18995832; 19847776; 19843221; 19843219 |
| FSU0615 | 8 | 0.83 | 21181421; 21109633; 21164002; 21041373; 20952576; 20888800; 20869947; 20869366; 20808844; 20805357 |
| FSU1000 | 8 | 0.90 | 18818364; 17977556; 16570183; 16418167; 12833515; 12270713; 11525742; 9287302; 3571278 |
| FSU1028 | 15 | 0.80 | 15263010; 19016841; 17303566; 8039908; 15583171; 15312768; 15263010; 12499568; 12125823; 10632888 |
| FSU1612 | 6 | 0.88 | 20484375; 12837750; 11136247; 8196650; 9493270 |
| FSU1682 | 19 | 0.84 | 21377658; 21314954; 21220037; 21126522; 20935120; 20823521; 20730755; 20680265; 20676918; 20654622 |
| FSU1729 | 19 | 0.88 | 20686915; 20562312; 20372022; 20139185; 19766588; 18998121; 18976664; 18931445; 8540419; 18759236 |
| FSU1831 | 6 | 0.90 | 21340633; 21288492; 21187428; 21123384; 21110832; 21094162; 21052000; 20924116; 20888795 |
| FSU1995 | 9 | 0.89 | 21378159; 21351321; 21347309; 21308848; 21256127; 21148142; 21093408; 21087632; 21080625; 21029287 |
| FSU2595 | 19 | 0.84 | 21297666; 21234373; 21151099; 20940366; 20940307; 20925002; 20888398; 20881132; 20864106; 20858757 |
| FSU2610 | 19 | 0.75 | 19783787; 19293368; 19121397; 18335278; 17286574; 17190185; 16431915; 7823963; 15274921; 14715651 |
| FSU3242 | 19 | 0.94 | 20843801; 3611062; 20364833; 20188057; 20077550; 20030628; 19929855; 19720067; 19277539; 19245333 |
| GBAA0352 | 8 | 0.92 | 20609914; 19549597; 18991392; 18981569; 18930846; 18211101; 12936980; 17385893; 2172928; 5700707 |
| GBOORF1093 | 12 | 0.71 | 20026006; 9009197; 3049578; 17272709; 10406942; 7913473; 7929183; 8166717; 8132503 |
| GBOORF1490 | 18 | 0.86 | 21397193; 21370995; 21339618; 21339577; 21338570; 21317284; 21315086; 21304597; 21215441; 21195087 |
| GBOORF1828 | 15 | 0.89 | 10852884; 9701816; 19271533; 8939696; 18080141; 18051268; 17473887; 16732458; 16385114; 16232284 |
| GBOORF2106 | 12 | 0.96 | 21352096; 21258033; 20624488; 19925415; 19508204; 19479740; 19038301; 18585473; 18575784; 9473502 |
| GBOORFA0202 | 13 | 0.97 | 20587522; 18948268; 10196363; 19664587; 17900615; 15247100; 15135053; 16943774; 15103084; 16712869 |
| GBOORFA0241 | 8 | 0.78 | 15059967; 11959456; 11422376; 11029287; 9774405; 9559562; 9443819; 8106447; 8281942; 8224215 |
| GBOORFA1041 | 13 | 0.97 | 19889645; 19106621; 11348589; 10811628; 10518522; 15614972; 12521300; 12009902 |
| GFRORF0559 | 8 | 0.90 | 20400563; 20337711; 20085751; 2538471; 18485344; 19285953; 15189131; 18162174; 18074176; 17655961 |
| GFRORF1023 | 1 | 0.79 | 21348779; 21319195; 21273246; 21161522; 21128730; 20871599; 20932060; 20876533; 20847010; 20812356 |
| GFRORF1537 | 8 | 0.73 | 16739977; 15292518; 14505379; 12236732; 11902903; 8563639; 10922373; 10682863; 10486127; 9149148 |
| GFRORF1595 | 18 | 0.71 | 21377658; 21360185; 21332878; 21315829; 21303543; 21268154; 21261630; 21256135; 21255651; 21250658 |
| GFRORF1661 | 19 | 0.80 | 18499663; 18494783; 17259976; 17420245; 17407266; 1826034; 12618437; 12480526; 12139940; 11545590 |
| GFRORF1747 | 12 | 0.97 | 21310089; 21296967; 21272581; 21254169; 21252346; 21237196; 21235002; 21224067; 21203407; 21182001 |
| GFRORF1974 | 1 | 0.89 | 20816894; 20714314; 20648599; 10470851; 20418392; 20390338; 19858196; 19596042; 10091662; 18831049 |
| GFRORF2065 | 4 | 0.71 | 21385871; 21376122; 21357485; 21354407; 21332941; 21319192; 21307569; 21306995; 21277337; 21264238 |
| GFRORF2209 | 4 | 0.85 | 20817757; 8039908; 10322028; 15583171; 12007411; 12223298; 12180929; 11830939; 2694934; 10754235 |
| GFRORF2222 | 19 | 0.84 | 21377658; 21314954; 21220037; 21126522; 20935120; 20823521; 20730755; 20680265; 20676918; 20654622 |
| GFRORF2359 | 12 | 0.77 | 21238935; 21233420; 21223741; 21220330; 21204942; 21098708; 21093502; 21076469; 20957652; 20810007 |
| GGOORF_0157 | 15 | 0.96 | 19202108; 11114919; 12686116; 11158353; 1318499; 9889976; 9389448; 9168623; 7582014; 4896022 |
| GGOORF_0470 | 17 | 0.84 | 21400116; 21399665; 21398637; 21398630; 21398531; 21398520; 21398469; 21398463; 21398445; 21398443 |
| GGOORF_0649 | 18 | 0.80 | 21393861; 21393174; 21393072; 21392379; 21391900; 21390069; 21389634; 21383919; 21383163; 21381755 |
| GGOORF_0851 | 6 | 0.71 | 20675469; 17324440; 18366438; 17906129; 17893144; 9731775; 16879868; 16624405; 16095750; 16023671 |
| GGOORF_1064 | 17 | 0.96 | 21398520; 21396820; 21387412; 21387281; 21379585; 21378190; 21375705; 21372179; 21371608; 21367571 |
| GGOORF_1232 | 18 | 0.84 | 21130728; 21098279; 20861461; 20810664; 20729329; 20690173; 20601156; 20530578; 20510935; 20509626 |
| GGOORF_1903 | 15 | 0.71 | 21398555; 21394897; 21385720; 21382349; 21381030; 21376736; 21376459; 21372139; 21362065; 21360678 |
| GGOORF_2683 | 8 | 0.80 | 20337711; 2538471; 18485344; 15189131; 9772177; 11313346; 10651802; 1310089; 8534850; 7782304 |
| GGOORF_3278 | 18 | 0.85 | 20199110; 942051; 10493868; 14763978; 11684686 |
| GGOORF_3414 | 18 | 0.75 | 21270798; 21268845; 21166718; 21164545; 21148012; 21147986; 21146149; 21110956; 21105363; 21093432 |
| GGOORF_3826 | 13 | 0.87 | 21152913; 21102444; 20947765; 20890290; 20838377; 20807316; 20797626; 20670889; 20659448; 20505071 |
| GGOORF_4318 | 8 | 0.81 | 21216999; 21070747; 20580617; 20413550; 20370825; 20236932; 20212162; 20206639; 20178464 |
| GGOORF_4339 | 5 | 0.80 | 20961069; 20943194; 20183076; 19429462; 19308726; 19058962; 18625336; 18288656; 17113870; 16860560 |
| GGOORF_5074 | 18 | 0.96 | 21266546; 20519548; 19887446; 19619244; 18701882; 10559939; 16563799; 16169010; 10200558; 15803409 |
| GGOORF_5521 | 6 | 0.85 | 21245038; 21228759; 2196557; 20663713; 2946419; 19576228; 15254239; 16150475; 17942449; 16407406 |
| GGOORF_5683 | 17 | 0.81 | 17690690; 14722088; 16513815; 12574165; 12504017; 10508665; 11369601; 9604891; 9099738; 8575880 |
| GGOORF_6442 | 12 | 0.94 | 21278271; 21130733; 21106832; 21040514; 21040511; 21037554; 20947023; 20930480; 20921220; 20877571 |
| GGOORF_7503 | 9 | 0.97 | 21389045; 21386062; 21376757; 21375498; 21368116; 21344388; 21342606; 21342605; 21327327; 21324704 |
| GGOORF_7962 | 6 | 0.74 | 1861990; 20633226; 9177283; 10476041; 17533171; 1482047; 12748189; 12427949; 12409632; 11020243 |
| GGOORF_8571 | 18 | 0.84 | 21347618; 21341536; 21257037; 21110011; 21063405; 21048306; 21306258; 21029887; 20970996; 20926601 |
| GGOORF_8613 | 2 | 0.72 | 19481404; 19359015; 16508242; 7327860; 7239796 |
| GKPORF_0008 | 6 | 0.82 | 20464017; 19462083; 17376083; 17376066; 17376071; 17317110; 16950091; 16814717; 16238625; 12142478 |
| GKPORF_0048 | 6 | 0.84 | 21242293; 21211720; 21143968; 20973964; 20952393; 20929865; 20864450; 20813592; 20713449; 20733069 |
| GKPORF_B0831 | 8 | 0.87 | 21329667; 20861021; 20831907; 20718865; 20690630; 20600130; 20592025; 20457604; 20334431; 1324665 |
| GKPORF_B1244 | 8 | 0.80 | 20013980; 19744988; 12514037; 17051653; 15672171; 15491145; 8246842; 1791759; 9056848; 3038334 |
| GKPORF_B1342 | 15 | 0.96 | 20347390; 18715143; 18328832; 10586519; 10347192; 11761187; 9829921; 2997779 |
| GKPORF_B1628 | 8 | 0.95 | 17680234; 16128611; 15112989; 7515059; 8185833; 3110160 |
| GKPORF_B1660 | 13 | 0.83 | 21394738; 21317554; 21282640; 21267462; 21209200; 21199936; 21195735; 21172307; 21163263; 21152913 |
| GKPORF_B1724 | 19 | 0.80 | 21393228; 21317946; 21166726; 20869941; 20862280; 20394845; 20208169; 20183598; 19646414; 19445596 |
| GKPORF_B1741 | 5 | 0.96 | 17408830; 10364262; 11823455; 8387524; 10564803; 2155202; 2455457; 2644218; 1429737; 1817706 |
| GKPORF_B1841 | 18 | 0.84 | 21342126; 21321137; 21319959; 21291474; 21284934; 21279999; 21271607; 21265097; 21262925; 21241819 |
| GKPORF_B1900 | 18 | 0.81 | 21183689; 21098000; 21056089; 21048979; 20975041; 20844141; 20737591; 20708685; 20691277; 20622037 |
| GKPORF_B2467 | 8 | 0.77 | 20831589; 20601217; 20419722; 20186410; 19931102; 19688823; 19665595; 18463136; 15583382; 18433620 |
| GKPORF_B2644 | 6 | 0.89 | 21034467; 20952573; 20622062; 20595230; 20570733; 20435136; 20211899; 18515342; 20132443; 19889099 |
| GKPORF_B2752 | 5 | 0.91 | 21226054; 17618313; 9384533; 9398217; 9371250; 8999955; 7775463 |
| GKPORF_B2895 | 12 | 0.91 | 18850720; 10829079; 14999401; 8626288; 16380269 |
| GKPORF_B3023 | 13 | 0.75 | 20606270; 1325384; 17623669; 19279227; 12717624; 18193200; 16890019; 8206848; 10076042; 15716138 |
| GKPORF_B3182 | 11 | 0.75 | 12558182; 9455479; 2530136; 6099311; 8406039 |
| GKPORF_B3510 | 11 | 0.97 | 17367389; 17302814; 17078817; 9889980; 10760133; 11907683; 10556026; 10878126; 10760133; 10320583 |
| GKPORF_B3647 | 2 | 0.93 | 20823222; 20543190; 12864856; 4345352; 8012595; 3663113; 9217019; 9163953; 8621563; 7607249 |
| GKPORF_B3771 | 4 | 0.76 | 15876366; 18756740; 11679348; 8282725; 3038334; 6094016; 3540312; 6304324 |
| GKPORF_B4119 | 6 | 0.94 | 21139085; 21067515; 20622854; 20403335; 15814643; 20208542; 19010785; 20057358; 9182762; 19047520 |
| GKPORF_B4228 | 18 | 0.72 | 21225311; 21114521; 21075922; 20924357; 20697693; 11580842; 19628558; 19420727; 2939801; 18029785 |
| GKPORF_B4349 | 18 | 0.94 | 21273394; 21191377; 21187464; 21107414; 21104926; 21062372; 21057057; 20971900; 20970441; 20960970 |
| GKPORF_B4391 | 3 | 0.95 | 21299650; 20498260; 20145101; 20086145; 19151141; 4041428; 18485865; 18400183; 8820654; 18179420 |
| GKPORF_B5477 | 19 | 0.76 | 20562306; 19356131; 10503534; 9644208; 9395316; 9182997; 9094436; 8797842; 1452020 |
| GRAORF_0001 | 19 | 0.97 | 21253720; 20944403; 20851126; 19544083; 9195887; 10069079; 17947240; 17823855; 271968; 17346264 |
| GRAORF_0175 | 4 | 0.80 | 20097452; 20096385; 12951241; 18165184; 15974590; 15492014; 14572387; 12007843; 1513876; 16887510 |
| GRAORF_0320 | 12 | 0.80 | 21183644; 11849553; 16027164; 12087102; 10904406; 9312074; 8595395; 8074654; 8025682 |
| GRAORF_0426 | 11 | 0.97 | 16181782; 18227257; 17367389; 10722135; 10986230; 9393713; 2548993; 10760133; 11958563; 11907683 |
| GRAORF_0666 | 19 | 0.94 | 21170548; 21054875; 21031187; 20801759; 20680572; 20357138; 19907056; 17970751; 17875553; 17349635 |
| GRAORF_0772 | 5 | 0.76 | 16932909; 10809706; 14694078; 11781802; 11668346; 10564803; 10217509; 1378430; 7876209; 2559312 |
| GRAORF_1010 | 13 | 0.98 | 20882017; 20606262; 20601684; 20541532; 20499650; 20457752; 20156451; 19914242; 18957446; 19770500 |
| GRAORF_1191 | 8 | 0.79 | 21265776; 20847256; 20498362; 20460582; 20406405; 20334431; 19948738; 18485344; 19035832; 7248267 |
| GRAORF_1254 | 12 | 0.79 | 21199021; 20695486; 12459185; 20110296; 20101515; 16091041; 19133232; 18201799; 14762173; 16427218 |
| GRAORF_1281 | 8 | 0.90 | 21391703; 21311031; 21281627; 21237294; 21156811; 21118710; 21113737; 20966073; 20963423; 20961996 |
| GRAORF_1622 | 13 | 0.99 | 21404088; 21403627; 21402928; 21402779; 21402715; 21402132; 21400704; 21400228; 21399643; 21398531 |
| GRAORF_2117 | 6 | 0.94 | 19573535; 15040448; 17535249; 16831867; 12077349; 10555141; 10970884; 11522779; 11262242; 8371988 |
| GRAORF_3210 | 6 | 0.91 | 21390502; 21356311; 21343179; 21322094; 21315812; 21291352; 21254323; 21220122; 21166493; 21104133 |
| GRAORF_3336 | 18 | 0.93 | 20855510; 20154192; 20070462; 19252923; 3060089; 19054109; 18463798; 17434477; 10361139; 16407176 |
| GRAORF_4777 | 18 | 0.98 | 21196294; 21191290; 21185261; 21126579; 21122131; 21087598; 21044960; 20923632; 20881245; 20824073 |
| GSU_0891 | 12 | 0.98 | 20639575; 17942541; 20173418; 19696109; 19544039; 17644627; 19152799; 18430084; 17786425; 17600056 |
| GSU_1090 | 12 | 0.75 | 21128782; 21061175; 20878062; 20596584; 20460647; 20442305; 20145614; 20128820; 20036255; 20029996 |
| GSU_1502 | 2 | 0.87 | 19878958; 8910497; 19392660; 15597200; 15356000 |
| GSU_1587 | 13 | 0.74 | 18948268; 16141453; 3049533; 10556305; 16943774; 15752202; 15581897 |
| GSU_1908 | 9 | 0.94 | 571431; 14651609; 16667073; 12093819; 9498570; 9370338; 9301097; 16887524; 8407984 |
| GSU_2345 | 15 | 0.88 | 20558511; 15263010; 17303566; 11316371; 12125823; 6323313 |
| GSU_2387 | 18 | 0.72 | 21117239; 20675482; 12477867; 18315685; 17073449; 15665647; 12468528; 12091127; 10429202; 9497386 |
| GSU_2436 | 8 | 0.77 | 20160912; 17923481; 17157320; 14992577; 14742428; 14644451; 12031470; 11955070; 11507102; 11448970 |
| GSU_2977 | 8 | 0.85 | 20883732; 20600873; 20148428; 19160513; 18801356; 12364791; 17221198; 16570322; 11297390; 11553768 |
| GSU_3019 | 8 | 0.71 | 17375285; 17367808; 15033367; 14742428; 14644451; 12031470; 11955070; 11168412; 10187829; 9546032 |
| GSU_3377 | 6 | 0.81 | 20974931; 20637419; 20410075; 17618295; 17707226; 18518821; 10497258; 15572765; 17912758; 10331874 |
| GSYN0006 | 6 | 0.85 | 21301105; 21254166; 21087076; 21169503; 20952390; 20962275; 20870749; 20852981; 20844218; 20833633 |
| GSYN0055 | 4 | 0.78 | 21183069; 20580675; 20497333; 17233825; 16146521; 1628843; 2681146; 18759781; 10671449; 18394147 |
| GSYN0164 | 18 | 0.74 | 21398614; 21398541; 21395625; 21393654; 21389774; 21389113; 21387010; 21386897; 21378199; 21378183 |
| GSYN0362 | 6 | 0.89 | 20693660; 19815694; 20530453; 20504867; 20499678; 20410075; 20140303; 20067765; 20032005; 19962393 |
| GSYN0632 | 19 | 0.90 | 21229881; 21030539; 20707404; 20347067; 20188057; 20161474; 15639242; 18258263; 16040347; 11500481 |
| GSYN0654 | 1 | 0.89 | 19383689; 19370061; 17624493; 10735883; 9531508; 14503880; 12948640; 12845493; 12715888; 12101301 |
| GSYN0726 | 4 | 0.87 | 21401077; 21322032; 21302907; 21287625; 21242720; 21209210; 21120619; 21075149; 21057456; 21051700 |
| GSYN0732 | 19 | 0.95 | 21183069; 20843347; 20497333; 20192190; 6351730; 17646652; 9238107; 18539032; 10564478; 17938168 |
| GSYN0802 | 12 | 0.79 | 21205202; 20000742; 19661058; 17038124; 15513925; 6259126; 7651187; 8051048; 8344936; 2033085 |
| GSYN0814 | 8 | 0.73 | 21178285; 20811135; 19966484; 19546514; 19424620; 19200041; 17291445; 16402358; 16221991; 15996793 |
| GSYN0928 | 12 | 0.89 | 17280684; 12369934; 11121403; 9276481; 3087629 |
| GSYN1256 | 14 | 0.75 | 21128170; 20048065; 17919281; 18776470; 18487606; 17004709; 14992575; 10688532; 10419637; 8617773 |
| GSYN1280 | 6 | 0.84 | 17493798; 18477668; 17569599; 12960407; 12882965; 10455195; 7605583; 1391611; 1717843; 7188181 |
| GSYN1298 | 8 | 0.88 | 20823527; 20190087; 20163155; 19923715; 19574215; 19402045; 10494852; 19110079; 18685206; 18482980 |
| GSYN1307 | 18 | 0.74 | 20566650; 19636250; 17855354; 18991398; 18032408; 16870614; 16077079; 9742136; 15066148; 12857732 |
| GSYN1663 | 2 | 0.74 | 21327093; 20871101; 20685834; 20209896; 20051244; 19103300; 18422659; 17473883; 16896603; 16262699 |
| GSYN1775 | 18 | 0.76 | 20711710; 11750807; 16999837; 16092522; 9922273; 12890034; 11709306; 12011037; 11754746; 7525269 |
| GSYN2023 | 8 | 0.75 | 21062748; 20716859; 20567911; 20059486; 19842439; 19830588; 19704083; 19674121; 19534905; 19231875 |
| GSYN2504 | 12 | 0.97 | 21247409; 21238933; 21098233; 21072173; 20961427; 20960971; 20950468; 20861008; 20852886; 20740538 |
| GSYN2618 | 12 | 0.92 | 20805355; 20410297; 20112455; 17194835; 19730897; 10931273; 19505802; 19249287; 17435169; 19152643 |
| GSYN3036 | 1 | 0.94 | 18481057; 18007551; 17589636; 17139593; 11309139; 8331070; 1644759; 8797099; 2681782 |
| GSYN3072 | 18 | 0.94 | 20006708; 9055073; 17499216; 11912227; 9575142; 8057356 |
| HI_0410 | 17 | 0.80 | 21379585; 21039781; 20624215; 19286133; 19843219; 19818022; 19426742; 18433632; 17572072; 10679470 |
| HI_0433 | 4 | 0.76 | 15357215; 10348751; 11770120; 7840574; 11044368; 11027804; 10873522; 10589984; 6572966; 2985470 |
| HI_0669 | 6 | 0.76 | 17316685; 16963438; 16882299; 16298387; 15588821; 12940821; 10482494; 10692383; 10913144; 10844646 |
| HI_1278 | 8 | 0.74 | 19132060; 18765906; 9786194; 18438923; 17377573; 14551431; 16686476; 16442642; 16232917; 16232847 |
| HIMB114_0706 | 2 | 0.76 | 21390227; 21343300; 20499837; 20443544; 17706543; 20347989; 19385043; 20153287; 19914215; 19560433 |
| HIMB114_1261 | 8 | 0.72 | 8639028; 3162770; 7764511; 1512562; 6641901 |
| HMPREF0554_0361 | 4 | 0.72 | 20955520; 20637762; 20144450; 11880056; 19703106; 18022332; 16842351; 19165133; 18852008; 18582518 |
| HMPREF0554_0370 | 8 | 0.94 | 17891922; 16762453; 10581550; 15604729; 15164997; 15056475; 14555654; 12875742; 12619155; 11358527 |
| HMPREF0554_0624 | 13 | 0.96 | 20584901; 20472640; 15766524; 10559285; 8757728 |
| HMPREF0554_0680 | 19 | 0.89 | 21388958; 21380775; 21378160; 21369973; 21369910; 21367878; 21367655; 21357626; 21357429; 21354463 |
| HMPREF0554_0781 | 19 | 0.93 | 21388958; 21378160; 21367655; 21342516; 21332624; 21307593; 21299880; 21297160; 21273341; 21268113 |
| HMPREF0554_1519 | 8 | 0.88 | 15009198; 11896456; 11390499; 8183345; 8661051; 10647888; 9647736 |
| HMPREF0554_1529 | 8 | 0.84 | 21391839; 21388699; 21388533; 21380803; 21376710; 21376665; 21372401; 21367494; 21359855; 21355597 |
| HMPREF0554_1616 | 8 | 0.86 | 21382340; 20118261; 19826007; 19318624; 19109241; 18268842; 762020; 18093907; 12914700; 17603507 |
| HMPREF0554_1840 | 8 | 0.74 | 21347362; 21339362; 21282377; 21184731; 21110129; 21073729; 20631153; 17989133; 20517649; 20208542 |
| HMPREF0554_2471 | 1 | 0.91 | 9398223; 6370397; 17113026; 16618097; 9398304; 16083849; 12032090; 3023184; 2988449 |
| HMPREF0890_0471 | 18 | 0.96 | 21393174; 21369825; 21366542; 21351087; 21347827; 21342117; 21325265; 21216355; 21210849; 21209459 |
| HMPREF0890_0661 | 19 | 0.92 | 21241053; 21199899; 21166709; 21057010; 21031309; 20832870; 20801880; 20801103; 20799091; 20699433 |
| HMPREF0890_0972 | 6 | 0.83 | 21290819; 21261070; 21258395; 21255731; 21242293; 21211720; 21187328; 21183718; 21172662; 21167178 |
| HMPREF0890_1050 | 19 | 0.90 | 21354427; 20886903; 21243086; 21212461; 21183069; 21172308; 21168384; 21154671; 21134281; 21126315 |
| HMPREF0890_1062 | 8 | 0.82 | 21336929; 21236692; 21228466; 21106768; 20971845; 20952574; 20889786; 20876337; 20862513; 20851994 |
| HMPREF0890_1464 | 19 | 0.84 | 20608745; 18784082; 18155726; 18077448; 11489886; 1465423; 8939710; 6783617; 3920658; 6825689 |
| HNE_0086 | 4 | 0.81 | 20302012; 9603889; 4595206; 19696109; 7885225; 17724598; 14973022; 17081641; 11222602; 11230141 |
| HNE_0714 | 8 | 0.86 | 20887732; 20516592; 19947964; 10759516; 19091740; 16348073; 12534290; 10800688; 18584170; 18391442 |
| HNE_1148 | 2 | 0.71 | 21081698; 21058708; 20184790; 20158163; 19931317; 19883126; 19915530; 2007585; 19342798; 18776677 |
| HNE_1410 | 18 | 0.83 | 21338516; 21190440; 19959581; 20387456; 20116460; 20081036; 19663511; 19307713; 19295548; 11895295 |
| HNE_2118 | 8 | 0.83 | 10320398; 1939137; 6386468; 8241149; 8508789; 1939137 |
| HNE_2798 | 12 | 0.74 | 21400585; 21398595; 21376040; 21360678; 21344733; 21331044; 21302808; 21290823; 21288162; 21258665 |
| HNE_2821 | 12 | 0.99 | 21362485; 21188150; 21042721; 20667819; 20630859; 20540960; 20526895; 20363282; 20206694; 20069636 |
| JJD26997_0551 | 12 | 0.70 | 21398045; 21396418; 21391719; 21390324; 21390270; 21388195; 21386061; 21385576; 21384867; 21384112 |
| KVP40_0007 | 12 | 0.94 | 21368759; 20637416; 20462489; 20427067; 20416323; 20068042; 19767395; 19726681; 19635595; 19557795 |
| LACJE0001_0077 | 8 | 0.80 | 21075928; 20885442; 20693323; 20521619; 20399798; 20097860; 2231712; 19672561; 1532388; 19391076 |
| LACJE0001_0246 | 19 | 0.85 | 21378160; 21342516; 21299880; 21206049; 21193824; 21193067; 21177247; 21174947; 21118988; 21063757 |
| LACJE0001_1432 | 19 | 0.76 | 20802066; 20576315; 20331423; 19783787; 19590984; 18838391; 19422833; 18366639; 18258263; 15178325 |
| LACJE0001_1439 | 6 | 0.84 | 21169356; 20920291; 20724443; 17980389; 16164551; 17986081; 1332053; 17233826; 5341484; 10348866 |
| LMOf2365_0134 | 11 | 0.78 | 11752276; 10669353; 12224521; 18298295; 18179387; 17078814; 16103125; 9287942; 11418146; 14585681 |
| LMOf2365_0398 | 2 | 0.98 | 18314961; 15501823; 11158296; 9748345; 8683340; 8335646; 8344439; 2551297; 13278344; 3040103 |
| LMOf2365_0530 | 18 | 0.86 | 17974510; 12601730; 15258141; 15153772; 12202490; 8662938; 9705652; 9551558; 9484231; 2495266 |
| LMOf2365_0659 | 18 | 0.86 | 17974510; 12601730; 15258141; 15153772; 12202490; 8662938; 9705652; 9551558; 9484231; 2495266 |
| LMOf2365_0729 | 4 | 0.77 | 20439729; 16249258; 17443716; 19361426; 19358329; 358191; 18955157; 10468575; 17085552; 17015641 |
| LMOf2365_1103 | 19 | 0.94 | 20709894; 20603129; 20554644; 20118281; 19737355; 19652332; 8743712; 11173485; 19210622; 18156677 |
| LMOf2365_2093 | 18 | 0.80 | 21299248; 20865003; 20519567; 20451502; 19932772; 19695261; 19669174; 19570948; 19557345; 10778972 |
| LMOf6854_0411.2 | 15 | 0.86 | 21393212; 21390327; 21386983; 21383239; 21378189; 21375706; 21369994; 21369825; 21364304; 21355787 |
| LMOf6854_2352.6 | 11 | 0.88 | 19895817; 17534690; 3000073; 271968; 6335533 |
| LMOh7858_0411.2 | 15 | 0.86 | 19000608; 17293407; 10612281; 17555437; 2120234; 16451192; 10430882; 7986004; 10903438; 8025679 |
| LMOh7858_0663.1 | 18 | 0.99 | 21402783; 21402586; 21401500; 21398536; 21398480; 21395979; 21395556; 21395536; 21394083; 21392495 |
| LMOh7858_1819.1 | 18 | 0.97 | 21103969; 21040688; 20979388; 20626656; 2017436; 20953582; 20362640; 20204475; 20103563; 20074572 |
| LMOh7858_2043.1 | 8 | 0.72 | 21393181; 21356593; 21353684; 21349442; 21325251; 21313732; 21262107; 21184744; 21130864; 21121160 |
| LMOh7858_2524.1 | 18 | 0.70 | 21207455; 21143936; 21081547; 21044875; 20881245; 20877283; 20870764; 20730247; 20656779; 20626869 |
| LMOh7858_2944 | 18 | 0.98 | 21220218; 21037069; 18349697; 18310123; 18303017; 16763184; 6185509; 15579558; 12949075; 12546731 |
| MAV_0118 | 4 | 0.74 | 21350664; 21233299; 21162678; 21084462; 20837474; 20815241; 20018382; 19880448; 12218036; 19602151 |
| MAV_0765 | 18 | 0.94 | 8830274; 15221452; 8843165; 3943911; 1447208 |
| MAV_0772 | 9 | 1.00 | 21389045; 21388747; 21383846; 21375498; 21371360; 21366865; 21369993; 21365447; 21362034; 21357772 |
| MAV_0857 | 8 | 0.92 | 20379701; 10367948; 18667222; 18606554; 17728257; 17185221; 15380647; 12895106; 12589497; 2024119 |
| MAV_1097 | 2 | 0.95 | 20861021; 1532388; 19415239; 19368556; 3322275; 15572765; 10829079; 11004177; 10978347; 17201004 |
| MAV_1316 | 8 | 0.80 | 21385584; 21369832; 21368150; 21364293; 21361388; 21350473; 21349979; 21348864; 21345474; 21335525 |
| MAV_1622 | 2 | 0.88 | 21081498; 20861021; 20690630; 20112869; 20039101; 19330765; 19122276; 18716757; 16407262; 18522945 |
| MAV_1769 | 6 | 0.78 | 21219854; 21205014; 21124948; 21079801; 20861182; 20813592; 20666462; 20628184; 20334433; 20223211 |
| MAV_1962 | 8 | 0.78 | 20727352; 20508930; 20504108; 20407804; 20083491; 19916920; 19855959; 19851725; 16432167; 17468253 |
| MAV_2075 | 8 | 0.71 | 20041954; 8034727; 19302486; 17509919; 16330045; 12486057; 16205910; 16187334; 10464202; 1625581 |
| MAV_2166 | 9 | 0.97 | 20334431; 19470521; 16430210; 14728675; 18005338; 17900701; 11524729; 10849007; 10869041; 9890906 |
| MAV_2710 | 19 | 0.72 | 20834142; 20702566; 20686913; 20680265; 20487018; 20414771; 6100313; 19818022; 19785462; 9133663 |
| MAV_2719 | 12 | 0.70 | 21329698; 20501794; 15843375; 19646995; 18519635; 18804477; 18346739; 16709864; 9767238; 16225851 |
| MAV_2987 | 8 | 0.88 | 21384861; 21370283; 21362632; 21289251; 21285336; 21275244; 21220430; 21193039; 21187465; 21130106 |
| MAV_2994 | 8 | 0.72 | 19405028; 18754683; 16818382; 12242011; 11236090; 10387114; 10080900; 9831644; 1613797; 2546007 |
| MAV_3169 | 9 | 0.96 | 21318295; 21258134; 21212523; 21045059; 20931591; 20924576; 20846931; 20836570; 20547355; 20522559 |
| MAV_3258 | 19 | 0.85 | 20919961; 20872591; 10362118; 20540529; 20443562; 20679605; 20369747; 20192807; 14367272; 19936627 |
| MAV_3510 | 8 | 0.81 | 19172265; 15814313; 17514677; 2199796; 15066784; 10438748; 12670689; 10589735; 9683494; 9634695 |
| MAV_3511 | 8 | 0.86 | 20980054; 20953859; 20837989; 20684326; 20632934; 20566993; 20195856; 20182771; 20173089; 20036650 |
| MAV_3905 | 19 | 0.90 | 19081844; 18352857; 16700076; 10542272; 15522073; 15053875; 12584002; 11738085; 11287126 |
| MAV_4287 | 12 | 0.82 | 21386798; 21378161; 21328541; 21283563; 21203395; 21136602; 21112838; 21078859; 21061425; 20980042 |
| MAV_5187 | 12 | 0.80 | 21398173; 21397570; 21397480; 21397320; 21397065; 21395221; 21394468; 21391724; 21391593; 21390227 |
| MCA_0030 | 12 | 0.86 | 20192920; 10421373; 9398514; 10887181; 8051170 |
| MCA_0089 | 3 | 0.99 | 21148726; 20722599; 20629752; 20363933; 20348257; 20338182; 19747550; 19423631; 19347566; 9542072 |
| MCA_0223 | 5 | 0.84 | 21393052; 21338415; 21088107; 21046332; 21036337; 20965680; 20847585; 20797998; 20221733; 20221547 |
| MCA_0272 | 6 | 0.94 | 1741244; 8903506; 16038930; 15713456; 10844674; 6094968 |
| MCA_0404 | 6 | 0.94 | 1741244; 15713456; 10844674; 9642063; 6094968 |
| MCA_1092 | 2 | 0.90 | 21206014; 21081498; 20709836; 20690630; 20606263; 20378651; 20219465; 20112869; 20063894; 19946146 |
| MCA_1288 | 19 | 0.96 | 20980996; 20586063; 20384696; 18818215; 19493008; 19210622; 18599819; 18558341; 18343340; 18343337 |
| MCA_1305 | 12 | 0.85 | 21326941; 21281590; 21210767; 20966518; 20890740; 20847048; 20559014; 20549191; 19000036; 20153593 |
| MCA_1639 | 9 | 0.99 | 21282470; 21163262; 21130179; 20886305; 20865380; 20512324; 20507988; 20472257; 20452984; 20146059 |
| MCA_1716 | 14 | 0.97 | 17701900; 18409506; 12083723; 10547847; 15470112; 7591066; 11751055; 11683362; 11101685; 10514564 |
| MCA_2306 | 17 | 0.90 | 10551881; 14592988; 16514143; 10371038; 11727826; 7688127; 7568114 |
| MCA_2731 | 8 | 0.76 | 21265776; 21068394; 18323662; 18237633; 11300770; 10828978 |
| MCA_2837 | 8 | 0.76 | 19759613; 17881660; 12893987; 12214664; 10089528; 8954574; 7720101; 8374037 |
| MCA_3096 | 8 | 0.81 | 2854198; 17628145; 17046835; 16268782; 15662561; 15022647; 12586342; 12498792; 11827481; 11746679 |
| MG_018 | 6 | 0.77 | 9305914; 7590261; 16298340; 14990586; 10970884; 11031099; 10910076; 8371988; 9748214; 9614128 |
| MJ_0765 | 8 | 0.76 | 15299589; 1336462; 1339351; 1511014; 1318833; 1318832 |
| MJ_0963 | 12 | 0.77 | 16630933; 16604481; 16602714; 6712611; 16135898; 16043025; 15782410; 15166985; 13221568; 15024734 |
| MJ_1121 | 6 | 0.86 | 19400780; 9917385; 10339816; 14507368; 6790143 |
| MSMEG_0122 | 18 | 0.96 | 20855615; 20855510; 20132828; 7010115; 18383009; 18310026; 17064364; 12610720; 10583393; 9492267 |
| MSMEG_0451 | 8 | 0.82 | 19409596; 19274741; 19038292; 19020988; 18845259; 18557388; 17483518; 17927979; 17891475; 16781452 |
| MSMEG_0475 | 19 | 0.84 | 5420057; 20525998; 18486535; 18057039; 17827659; 17329255; 15660207; 15383535; 15134748; 9257704 |
| MSMEG_0740 | 19 | 0.98 | 20802066; 20656870; 20562284; 20382819; 18820840; 8070556; 18309273; 17641888; 15614968; 12754107 |
| MSMEG_0875 | 18 | 0.86 | 19822337; 8257103; 18399798; 17764658; 17202864; 16118285; 15917580; 15818560; 10544289; 9644260 |
| MSMEG_0986 | 13 | 0.77 | 21320180; 10681447; 17245409; 14592990; 11223267; 12493913; 12519988; 11743721; 9254694; 7745684 |
| MSMEG_2237 | 8 | 0.83 | 21097588; 21037180; 20453150; 7984417; 17521419; 19608925; 16699521; 16034418; 9245800 |
| MSMEG_2462 | 8 | 0.91 | 21275368; 21247090; 21176053; 21130022; 21068776; 20979125; 20956531; 20828392; 20694853; 20659147 |
| MSMEG_2477 | 8 | 0.80 | 10940013; 10375171; 9193789; 9061190; 8639709; 8789834; 18613021; 3159722; 7052137 |
| MSMEG_3289 | 6 | 0.70 | 21129204; 2466840; 19700405; 18462391; 18221264; 17179933; 17174325; 15909989; 15897200; 2822257 |
| MSMEG_3304 | 8 | 0.87 | 21371425; 21303655; 20696867; 20639325; 20576519; 20570675; 20548048; 20363598; 20304328; 20304089 |
| MSMEG_3346 | 8 | 0.82 | 19019008; 11732896; 10537203; 10080900; 9846747; 9819231; 2061291; 8026764; 8223657 |
| MSMEG_4217 | 4 | 0.82 | 21036658; 20622015; 20502438; 20453092; 17326815; 20178847; 19903201; 19737354; 19698693; 1925029 |
| MSMEG_4375 | 9 | 0.75 | 20590527; 20370610; 20178986; 8406042; 20099411; 20080211; 7045078; 637839; 19686777; 13595904 |
| MSMEG_4419 | 8 | 0.79 | 20920450; 20890095; 20879962; 20865387; 20834161; 20805024; 20739172; 20702080; 20676631; 20673625 |
| MSMEG_4521 | 12 | 0.94 | 21125380; 20847219; 20812717; 20599707; 20487024; 20485744; 20333370; 20064164; 19799526; 19778964 |
| MSMEG_4914 | 4 | 0.72 | 18450221; 17952731; 15574480; 3149758; 11467949; 10774259; 1720665; 10197038; 6345791; 1399951 |
| MSMEG_5720 | 9 | 0.82 | 21075928; 21034488; 20835703; 20638476; 20378648; 20017484; 19947964; 19638338; 19366370; 19088433 |
| MSMEG_6026 | 8 | 0.74 | 21310479; 21278273; 21197843; 21169482; 20965335; 20923481; 20860559; 20855003; 20838866; 20808573 |
| MSMEG_6213 | 4 | 0.82 | 21185807; 19201019; 20233930; 2404518; 20055466; 19933836; 19495574; 19129167; 18284847; 17994727 |
| MSMEG_6242 | 8 | 0.89 | 15389597; 19115036; 17294170; 16027951; 14705036; 7826011; 14638414; 12855725; 11566129; 2971647 |
| MSMEG_6319 | 19 | 0.88 | 21393206; 21216228; 21183069; 20974151; 20961110; 20870765; 20849416; 20580675; 20347848 |
| MSMEG_6712 | 8 | 0.88 | 20359211; 20054138; 19684066; 8550433; 19242755; 19149164; 16963440; 12107140; 18331335; 37837 |
| MT_0381 | 8 | 0.71 | 20008709; 19523468; 11779234; 18505728; 15279962; 18085563; 17955389; 17900823; 17576770; 17559390 |
| MT_1073 | 12 | 0.81 | 20602853; 20510023; 20120222; 19658200; 19628562; 17604188; 17069638; 16598647; 16520377; 16401476 |
| MT_1169 | 9 | 0.93 | 20043999; 19659733; 15345482; 15033994; 11915938; 10972423; 1673680; 8547181; 7883054; 7881821 |
| MT_1322 | 8 | 0.76 | 20819067; 18167490; 19036964; 19360879; 20483300; 17697045; 16234806; 17219437; 16609906; 15590750 |
| MT_2041 | 19 | 0.80 | 19117793; 12071845; 3678489; 15082932; 14499588; 10198752; 11755056; 11222941; 10939251; 942051 |
| MT_3512 | 12 | 0.92 | 20965153; 20873853; 16365159; 20448898; 20345980; 10896954; 19707939; 19539899; 12626583; 18684973 |
| MT_3820 | 19 | 0.78 | 21288904; 20586063; 8662184; 20127467; 20036252; 19389766; 19025870; 15973422; 18440165; 18338579 |
| MXAN_0140 | 17 | 0.87 | 21164032; 21156400; 21149580; 21139582; 21124318; 21114873; 21078673; 21060858; 21060794; 21041632 |
| MXAN_0837 | 8 | 0.85 | 21301108; 21226315; 21178087; 21159649; 21129192; 21090237; 21058063; 21036757; 20977478; 20952389 |
| MXAN_1713 | 12 | 0.92 | 20230833; 19526727; 16388577; 10677216; 10824098; 10544281 |
| MXAN_2888 | 8 | 0.80 | 19944166; 16218869; 16159891; 15450488; 15194705; 14605502; 10209742; 9493381; 9454777; 1990004 |
| MXAN_3210 | 19 | 0.91 | 21393206; 20974151; 20870765; 20849416; 20580675; 20206184; 20013255; 19807868; 17646652; 845124 |
| MXAN_4126 | 8 | 0.79 | 21385584; 21348864; 21335525; 21310654; 21306142; 21284395; 21265508; 21256041; 21228494; 21225390 |
| MXAN_5165 | 8 | 0.93 | 19923715; 19574215; 19110079; 18216065; 17313403; 17131148; 16919403; 15362852; 15302401; 14530261 |
| MXAN_5247 | 19 | 0.97 | 21193828; 20960239; 20720016; 20703955; 20562284; 20467831; 19883124; 19783787; 19527927; 18997327 |
| MXAN_5557 | 8 | 0.83 | 21051545; 20652619; 20347849; 17280684; 20025616; 19876765; 19811920; 2294092; 1555572; 18355283 |
| MXAN_6016 | 8 | 0.82 | 21336929; 21236692; 21228466; 21106768; 20971845; 20952574; 20889786; 20876337; 20862513; 20851994 |
| MXAN_6630 | 8 | 0.80 | 20861021; 20593880; 20334431; 19904831; 19850617; 18581061; 15466509; 1137083; 15897978; 17074519 |
| MXAN_6642 | 18 | 0.84 | 21080032; 20969756; 20966097; 20807204; 19645445; 19624751; 18539730; 17245413; 18582596; 16139844 |
| NEIFL0001_0930 | 6 | 0.83 | 21336542; 21312235; 21265762; 21219465; 21193392; 21148395; 21204301; 21044871; 20975905; 20937909 |
| NMB_0846 | 19 | 0.71 | 17169333; 15356101; 12843783; 11716961; 11141174 |
| NMB_1220 | 18 | 0.93 | 19032151; 536918; 17942620; 16101677; 12122055; 11435687; 10993673; 10742096; 9931417; 9074782 |
| NT01AA0085 | 5 | 0.91 | 21318879; 21318878; 21303766; 21295581; 21295540; 21155529; 21041039; 20861309; 20838441; 20798393 |
| NT01AA0255 | 18 | 0.95 | 21197505; 21147627; 21140912; 21044958; 21037554; 21036245; 20949136; 20926757; 20876384; 20851114 |
| NT01AA0437 | 19 | 0.92 | 20356840; 942051; 18498310; 18205357; 16458488; 16262785; 15637732; 10585410; 12417409; 11739642 |
| NT01AA0472 | 2 | 0.94 | 1141859; 12393194; 7982968; 2542220; 791939; 14217462; 1097404 |
| NT01AA0525 | 4 | 0.81 | 21399697; 21399666; 21399624; 21394085; 21392133; 21389114; 21385724; 21368915; 21368139; 21359677 |
| NT01AA0608 | 3 | 0.93 | 21142157; 21130745; 21115127; 21036658; 20971868; 20738375; 20714501; 20708667; 20656905; 20616068 |
| NT01AA0654 | 9 | 0.76 | 21081696; 20594840; 20562282; 20557983; 20547590; 20418430; 20304657; 20221630; 20153183; 20135119 |
| NT01AA0755 | 8 | 0.87 | 21209092; 20516622; 20453144; 20375021; 20364333; 18086808; 18469270; 18941301; 18845259; 9398220 |
| NT01AA0841 | 18 | 0.83 | 20111865; 15571508; 10608770; 10519401; 8411712 |
| NT01AA0856 | 19 | 0.86 | 18755141; 11251840; 1790305; 1790304; 1720755 |
| NT01AA0859 | 19 | 0.97 | 21367879; 21315310; 21272313; 21250655; 21206755; 21147851; 20958982; 20870880; 20854854; 20843801 |
| NT01AA0860 | 5 | 0.93 | 20890097; 20851900; 20586061; 20466643; 20382111; 20374528; 20042022; 17835129; 12637555; 19770499 |
| NT01AA0867 | 4 | 0.82 | 21387009; 21079963; 20414301; 20414307; 20373248; 10353518; 16983090; 9278503; 19935887; 19923603 |
| NT01AA0870 | 18 | 0.98 | 21232060; 21135102; 21118978; 21041493; 20978004; 20888343; 20876358; 20859232; 20856239; 20855510 |
| NT01AA0937 | 8 | 0.77 | 11741948; 10387078; 9037110; 8866660; 7811234; 8468468; 5926184 |
| NT01AA0975 | 18 | 0.99 | 21328631; 21282456; 21134356; 21081547; 21062372; 21042264; 20955466; 20951729; 20882639; 20855745 |
| NT01AA0998 | 18 | 0.72 | 10704219; 18639631; 12016214; 11300785; 10748025; 3192527 |
| NT01AA1007 | 4 | 0.83 | 19162196; 17640273; 15576792; 16309817; 9675890; 10361275; 9642082; 8152377; 1453957 |
| NT01AA1010 | 5 | 0.98 | 21369825; 21317335; 21261641; 21074515; 20855745; 20821001; 20501439; 20498339; 20087755; 20039042 |
| NT01AA1044 | 8 | 0.81 | 21387033; 21315823; 21310654; 21251101; 21132328; 21112414; 20971198; 20943545; 20922418; 20876714 |
| NT01AA1058 | 6 | 0.71 | 21386365; 21367911; 21265761; 21239155; 21228632; 21178304; 21129204; 21102582; 21094707; 21084480 |
| NT01AA1086 | 8 | 0.97 | 21382338; 21324314; 21239558; 21179059; 21109418; 21092633; 21042417; 20924414; 20854917; 20737854 |
| NT01AA1100 | 8 | 0.73 | 8325373; 9593850; 10750708; 9588024; 8673340; 1463844 |
| NT01AA1101 | 8 | 0.90 | 21387033; 21329181; 21303200; 21132328; 20808932; 20620150; 15987803; 20551160; 20545001; 20454804 |
| NT01AA1185 | 9 | 0.94 | 20956528; 20528952; 20455949; 20434430; 20185506; 19330542; 8863531; 18759116; 18691575; 11025668 |
| NT01AA1191 | 13 | 0.91 | 19914359; 19446023; 12730326; 16315110; 15621578; 15378713; 14662309; 14586115; 12055297; 11453072 |
| NT01AA1242 | 18 | 0.95 | 21357299; 21344396; 21089447; 21084795; 20974830; 20954009; 20935265; 20876745; 20855745; 20718869 |
| NT01AA1274 | 8 | 0.77 | 21397687; 21397458; 21390464; 21378552; 21377832; 21377831; 21377830; 21377321; 21358537; 21354808 |
| NT01AA1370 | 9 | 0.98 | 21392131; 21389266; 21385214; 21382219; 21378135; 21376780; 21375498; 21374590; 21372039; 21371432 |
| NT01AA1374 | 19 | 0.77 | 21205672; 21188072; 21092180; 21054963; 21030539; 20724435; 15695395; 20473714; 20388532; 20331963 |
| NT01AA1428 | 5 | 0.73 | 21147779; 20378984; 20361933; 20153593; 20143161; 20069462; 19730970; 19616092; 12603203; 19421892 |
| NT01AA1563 | 8 | 0.74 | 21236260; 21126315; 21074536; 21063756; 20974739; 20957753; 20849415; 20709031; 5420057; 20458737 |
| NT01AA1626 | 9 | 0.72 | 10446428; 1716161; 10329729; 3692487; 8005353; 8323974; 1571364; 1860841; 3196740; 3622518 |
| NT01AA1664 | 18 | 0.90 | 20797386; 20570749; 15757906; 18471079; 11878472; 17632081; 17628207; 16166347; 15175298; 10835424 |
| NT01AA1674 | 14 | 1.00 | 15641804; 9551557; 1447788; 1797375; 1781372; 6348505 |
| NT01AA1707 | 6 | 0.94 | 10677682; 1987126; 8918248; 8068630; 7517004; 3932331; 1267736 |
| NT01AB0549 | 9 | 0.70 | 21392988; 21383171; 21375532; 21372125; 21365225; 21355555; 21351219; 21347292; 21341672; 21341516 |
| NT01AB0897 | 15 | 0.73 | 21278272; 21257773; 21256953; 21237626; 21181144; 21178479; 21151497; 20979333; 20971871; 20831412 |
| NT01AB1274 | 6 | 0.95 | 21343909; 21078962; 20736290; 20708016; 20657659; 20568999; 20484375; 20472641; 20451470; 20443711 |
| NT01AB1359 | 9 | 0.96 | 20725044; 20616867; 19423627; 1355089; 15514159; 18557770; 17266990; 10716717; 16707089; 16276872 |
| NT01AB1391 | 6 | 0.82 | 21362551; 21338660; 21303393; 21243721; 21227473; 21189471; 21086481; 21052759; 21045305; 20978746 |
| NT01AB2105 | 6 | 0.91 | 21393220; 21347446; 21317560; 21301087; 21229398; 21193392; 21178304; 21172664; 21152448; 21071401 |
| NT01AB2656 | 13 | 0.96 | 21102444; 20965064; 20947765; 20890290; 20876129; 20807316; 20724456; 20712004; 20670889; 20561528 |
| NT01AB2898 | 19 | 0.73 | 18461319; 17561300; 2744487; 14907713; 11710008; 3611052 |
| NT01AD0374 | 13 | 0.99 | 21402928; 21399665; 21399643; 21394717; 21392374; 21378123; 21368145; 21364490; 21351069; 21347434 |
| NT01AD0860 | 6 | 0.77 | 20624486; 19528180; 11526167; 18281457; 11445083; 12940949; 16322989; 15778718; 14985346; 11136769 |
| NT01AD1317 | 14 | 0.96 | 21333632; 19948730; 19666465; 15611115; 14614131; 18804703; 3877056; 18056801; 15304656; 18804703 |
| NT01AD2781 | 8 | 0.92 | 20951673; 19466416; 17716833; 19103151; 10406803; 17933581; 17092311; 12167658; 11891134; 3959077 |
| NT01AD2795 | 4 | 0.88 | 21091512; 10785634; 2450866; 8698506; 15889074; 10627048; 1439759; 1707541; 1435261 |
| NT01AD2953 | 8 | 0.72 | 21362632; 21359959; 21343247; 21276778; 21256041; 21238579; 21187465; 21130106; 21117316; 21113494 |
| NT01AD3054 | 18 | 0.88 | 10542219; 19927326; 19847921; 19158789; 19016846; 18540842; 18315685; 17073449; 17068171; 17011224 |
| NT01AD3392 | 8 | 0.91 | 21346809; 21329684; 21317765; 21306562; 21296056; 21277289; 21277203; 21276783; 21276435; 21255593 |
| NT01AD3694 | 8 | 0.91 | 21400100; 21397737; 21396889; 21396131; 21394044; 21393444; 21393246; 21393237; 21388872; 21387012 |
| NT01AD4103 | 9 | 0.89 | 8082824; 20013982; 19954230; 19777301; 19733180; 18451049; 18319060; 11036020; 17237222; 16793520 |
| NT01AE0073 | 15 | 0.72 | 20952573; 20942908; 2265755; 20507519; 10633114; 13376513; 8780507; 319344 |
| NT01AE0074 | 15 | 0.89 | 21051490; 16000707; 13129619; 11497997; 7913926 |
| NT01AE0088 | 8 | 0.96 | 20653766; 20350544; 15347752; 16348351; 18983854; 18973623; 18197582; 16662666; 17720337; 17418088 |
| NT01AE0096 | 15 | 0.84 | 21402729; 21337578; 21267420; 21173260; 21116826; 21070394; 21051490; 21045860; 21218576; 21036163 |
| NT01AE0155 | 6 | 0.92 | 16298340; 14990586; 10970884; 8371988; 9025290; 1312673 |
| NT01AE0226 | 8 | 0.73 | 21178285; 20811135; 19966484; 19546514; 19424620; 19200041; 17291445; 16402358; 16221991; 15996793 |
| NT01AE0317 | 19 | 0.96 | 18499663; 18210176; 16829524; 16794327; 15820665; 15555940; 15552059; 15226299; 15161861; 12758148 |
| NT01AE0375 | 4 | 0.85 | 21118484; 2985470; 16487743; 20199603; 20118266; 2030670; 19961544; 8707053; 8071222; 2853689 |
| NT01AE0383 | 8 | 0.73 | 21178285; 20811135; 19966484; 19546514; 19424620; 19200041; 17291445; 16402358; 16221991; 15996793 |
| NT01AE0386 | 18 | 0.99 | 21357299; 21315686; 21182225; 21090806; 21089447; 21084795; 21081547; 21037109; 20974830; 20938527 |
| NT01AE0487 | 1 | 0.77 | 21393246; 21383205; 21372135; 21219974; 21210868; 21185009; 21138988; 21081499; 21079812; 20971073 |
| NT01AE0540 | 15 | 0.97 | 20497226; 20056703; 19818022; 19570137; 19138193; 17711452; 18310026; 14561776; 11179370; 9148780 |
| NT01AE0578 | 8 | 0.78 | 21375708; 21365755; 20967536; 20002588; 19043737 |
| NT01AE0761 | 12 | 0.86 | 21388519; 21385063; 21352228; 21349154; 21334374; 21326357; 21322006; 21316457; 21300156; 21289117 |
| NT01AE0785 | 18 | 0.70 | 21399938; 21398549; 21398292; 21397696; 21389131; 21387181; 21385715; 21385626; 21385335; 21383014 |
| NT01AE0980 | 15 | 0.81 | 21205010; 21039781; 20965974; 20851895; 20497226; 19843219; 19570137; 18599839; 19138193; 19076234 |
| NT01AE1058 | 19 | 0.91 | 18210176; 15820665; 15552059; 12686644; 10521532 |
| NT01AE1066 | 18 | 0.92 | 21393174; 21389634; 21372393; 21366542; 21362586; 21360409; 21352852; 21351087; 21349151; 21348297 |
| NT01AE1071 | 18 | 0.97 | 21385839; 21362161; 21356339; 21350490; 21347707; 21315728; 21308399; 21296088; 21170876; 21272644 |
| NT01AE1088 | 18 | 0.99 | 21221942; 21041493; 20348251; 20100880; 19574656; 19254725; 19166984; 18177365; 17893146; 15855496 |
| NT01AE1123 | 8 | 0.73 | 10698784; 16780798; 12445643; 1310545; 1385030 |
| NT01AE1132 | 12 | 0.88 | 20368502; 20409632; 2646153; 18988691; 2231712; 11419950; 17453174; 15012905; 16542876; 16530268 |
| NT01AE1141 | 9 | 0.83 | 20590527; 20370610; 20178986; 8406042; 20099411; 20080211; 19686777; 19571038; 19525201; 15461798 |
| NT01AE1152 | 18 | 0.72 | 21403916; 21403643; 21402783; 21402762; 21402707; 21402114; 21402067; 21401840; 21401756; 21401713 |
| NT01AE1200 | 8 | 0.73 | 21178285; 20811135; 19966484; 19546514; 19424620; 19200041; 17291445; 16402358; 16221991; 15996793 |
| NT01AE1203 | 8 | 0.73 | 21178285; 20811135; 19966484; 19546514; 19424620; 19200041; 17291445; 16402358; 16221991; 15996793 |
| NT01AE1205 | 8 | 0.73 | 21178285; 20811135; 19966484; 19546514; 19424620; 19200041; 17291445; 16402358; 16221991; 15996793 |
| NT01AE1271 | 18 | 0.84 | 20815017; 20628435; 20460875; 20460828; 20413720; 20147439; 20133363; 19833843; 19806079; 19497955 |
| NT01AE1302 | 8 | 0.78 | 10611284; 2384085; 2384085; 7607243; 7607242; 1633822; 2176602; 2384085; 2591380; 2591379 |
| NT01AE1305 | 18 | 0.88 | 21393247; 21386735; 21362444; 21355107; 21346352; 21336588; 21311139; 21255364; 21224231; 21220496 |
| NT01AE1338 | 18 | 0.91 | 21315686; 21311724; 21177413; 21122131; 21111784; 21090806; 21078855; 21071707; 21059948; 21059651 |
| NT01AE1435 | 15 | 0.95 | 21393367; 21385202; 21362064; 21342462; 21330435; 21320584; 21315771; 21306428; 21304896; 21295603 |
| NT01AE1591 | 5 | 0.97 | 21392199; 21336695; 21175893; 21075841; 21072861; 20473684; 20139159; 20022138; 15579666; 17600048 |
| NT01AE1657 | 12 | 0.89 | 21397717; 21378058; 21357721; 21345358; 21343416; 21338918; 21338912; 21335977; 21326941; 21321669 |
| NT01AE1747 | 18 | 0.77 | 12731863; 11741943; 11717516; 1463743; 2294058; 1544915; 2102832 |
| NT01AE1799 | 15 | 0.92 | 21205010; 21039781; 20965974; 20862323; 20851895; 20497226; 20056703; 19843219; 19818022; 2316135 |
| NT01AE1928 | 12 | 0.79 | 21269876; 20847219; 20691708; 19153597; 20547206; 20594290; 20488436; 4877784; 20466848; 20433886 |
| NT01AE1934 | 8 | 0.73 | 20370825; 20138824; 19932528; 19874915; 19660473; 19552984; 19342073; 19151966; 7248267; 18448193 |
| NT01AE1939 | 8 | 0.85 | 20158523; 19557348; 5056224; 19284767; 18435559; 16217726; 15066784; 7793958; 14618567; 12687299 |
| NT01AE1952 | 8 | 0.70 | 18452539; 18408355; 18088303; 16388583; 15668249; 9095201; 8798648; 7893703; 8137935; 8120891 |
| NT01AE2036 | 11 | 0.75 | 21402865; 21393370; 21375706; 21368277; 21356525; 21350632; 21335390; 21323982; 21310067; 21277379 |
| NT01AE2126 | 13 | 0.94 | 20598281; 20197408; 20234387; 10656263; 19463886; 10824085; 11388898; 18391411; 18342886; 16873721 |
| NT01AE2181 | 8 | 0.92 | 21168410; 20868295; 20600801; 20213442; 20209439; 1324665; 20006584; 19899808; 19761232; 7173200 |
| NT01AE2220 | 8 | 0.84 | 19833508; 16200391; 12962329; 12851403; 12651584; 10791819; 8605224; 7901008; 786161; 35513 |
| NT01AE2229 | 15 | 0.81 | 10517579; 10915804; 1494353; 9141695; 8153625; 1708380 |
| NT01AE2230 | 8 | 0.71 | 21399882; 21396977; 21395315; 21390324; 21389278; 21386917; 21381885; 21377535; 21377464; 21377369 |
| NT01AE2232 | 9 | 0.83 | 21362022; 21352884; 21134903; 21257607; 21258118; 20956318; 20804609; 20825197; 20601511; 20541526 |
| NT01AE2286 | 15 | 0.98 | 20946846; 20817745; 20447287; 19411418; 17183208; 6550579; 12614149; 19460100; 17908933; 16345414 |
| NT01AE2425 | 15 | 0.91 | 20643656; 20012281; 8830274; 10746760; 12783863; 11418552; 2781284; 1480115; 11129050; 10217494 |
| NT01AE2478 | 19 | 0.73 | 21178073; 20688825; 8386125; 19211098; 18833547; 18459313; 11371519; 17880933; 10699503; 15795227 |
| NT01AE2482 | 8 | 0.81 | 2854198; 17628145; 17046835; 16268782; 15662561; 15022647; 12586342; 12498792; 11827481; 11746679 |
| NT01AE2484 | 4 | 0.88 | 20822537; 9917404; 15945374; 15854646; 2615761; 11890555; 11741897; 11454201; 10873460; 10196173 |
| NT01AE2488 | 18 | 0.97 | 20957165; 20737137; 20704181; 20639324; 20561140; 20395595; 20185911; 16099468; 6467192; 19775248 |
| NT01AE2489 | 18 | 0.96 | 20737137; 20704181; 20658302; 20639324; 20561140; 20395595; 19775248; 19761223; 18606475; 19428657 |
| NT01AE2565 | 8 | 0.77 | 21125567; 20974948; 20854995; 20837989; 20824049; 20731849; 20718298; 20601070; 20595031; 20445323 |
| NT01AE2610 | 12 | 0.86 | 21388519; 21385063; 21352228; 21349154; 21334374; 21326357; 21322006; 21316457; 21300156; 21289117 |
| NT01AE2641 | 19 | 0.77 | 20202763; 17400891; 12397186; 18342249; 17007421; 15927751; 12650935 |
| NT01AE2778 | 15 | 0.90 | 21057008; 20833804; 20954302; 20512483; 18052041; 2180908; 19220749; 10745001; 18818215; 1380671 |
| NT01AE2836 | 18 | 0.96 | 21349151; 21265893; 21226332; 21175590; 21168419; 21135102; 21134393; 21130497; 21122131; 21090806 |
| NT01AE2851 | 18 | 0.87 | 20862303; 20581474; 20405931; 20188062; 18931440; 18691556; 18451056; 648533; 12167852; 17562318 |
| NT01AE2870 | 8 | 0.89 | 21398391; 21388533; 21388532; 21378355; 21367571; 21364306; 21359858; 21357267; 21355870; 21354561 |
| NT01AE2927 | 9 | 0.77 | 21386062; 21375498; 21342605; 21332878; 21327327; 21320492; 21320485; 21296915; 21294661; 21284260 |
| NT01AE2943 | 18 | 0.87 | 21399724; 21396366; 21395556; 21395542; 21395536; 21393626; 21393212; 21393173; 21391724; 21389634 |
| NT01AE2988 | 8 | 0.73 | 20813141; 20803137; 20472738; 20447326; 20133363; 19943898; 19659660; 19545523; 19486386; 19430792 |
| NT01AE3122 | 8 | 0.73 | 21178285; 20811135; 19966484; 19546514; 19424620; 19200041; 17291445; 16402358; 16221991; 15996793 |
| NT01AE3246 | 18 | 0.93 | 21122131; 21090806; 21071707; 21059948; 21059651; 20935265; 20921309; 20870774; 20855510; 20838378 |
| NT01AE3285 | 12 | 0.94 | 21378284; 21372727; 21352200; 21351429; 21331046; 21327682; 21327498; 21310076; 21304989; 21303546 |
| NT01AE3334 | 15 | 0.88 | 21390327; 21389340; 21386983; 21385202; 21383239; 21379585; 21375706; 21369825; 21347346; 21340608 |
| NT01AE3369 | 15 | 0.78 | 21347346; 21205010; 21039781; 20851895; 20497226; 20482591; 20370825; 19843219; 19245942; 19138193 |
| NT01AE3374 | 8 | 0.78 | 21376550; 20632934; 19543983; 12743764; 2985470; 612708; 344137; 5838388 |
| NT01AE3513 | 19 | 0.95 | 18258263; 16040347; 18499663; 18325534; 18210176; 16829524; 16794327; 15820665; 15552059; 15226299 |
| NT01AE3543 | 8 | 0.91 | 21221570; 21184752; 21140150; 20737003; 20718298; 20699116; 20551082; 20457944; 20359890; 20190094 |
| NT01AE3556 | 9 | 0.97 | 16430210; 14728675; 11524729; 10869041; 15146484; 14752098; 12962497; 11872165; 11812788; 11700068 |
| NT01AE3578 | 8 | 0.89 | 21398391; 21388533; 21388532; 21378355; 21367571; 21364306; 21359858; 21357267; 21355870; 21354561 |
| NT01AE3598 | 17 | 0.78 | 21378185; 21321231; 21139222; 21126315; 21045543; 20956557; 20944626; 20935500; 20844015; 20705241 |
| NT01AE3637 | 15 | 0.87 | 21404442; 21403627; 21403626; 21403465; 21403389; 21402798; 21402785; 21402777; 21402776; 21402769 |
| NT01AE3747 | 8 | 0.73 | 21178285; 20811135; 19966484; 19546514; 19424620; 19200041; 17291445; 16402358; 16221991; 15996793 |
| NT01AE3806 | 8 | 0.78 | 21327819; 20593880; 17815852; 15466509; 17630784; 15897978 |
| NT01AE3868 | 12 | 0.88 | 21394206; 21390501; 21375693; 21352228; 21301927; 21256965; 21247063; 21214543; 21196474; 21143798 |
| NT01AE3931 | 18 | 0.89 | 21369825; 21204689; 21122131; 21090806; 21071707; 21059948; 21059651; 20955543; 20954009; 20938339 |
| NT01AE3991 | 6 | 0.84 | 21399806; 21399673; 21395071; 21390259; 21389155; 21384253; 21383776; 21383132; 21378315; 21378122 |
| NT01AE4042 | 18 | 0.87 | 21400686; 21400247; 21399882; 21399724; 21399631; 21399613; 21399568; 21399389; 21398536; 21398509 |
| NT01AE4144 | 12 | 0.99 | 21189343; 21135574; 21115495; 21095572; 21075926; 20872041; 12382110; 20566871; 20522495; 20419406 |
| NT01AE4190 | 8 | 0.86 | 21387444; 21364306; 21358763; 21357773; 21354350; 21331250; 21327408; 21315475; 21311031; 21297942 |
| NT01AE4327 | 19 | 0.93 | 21279381; 21174346; 20920201; 20672277; 20636376; 3611062; 20383007; 11752303; 20199575; 20124190 |
| NT01AE4348 | 18 | 0.96 | 21393174; 21392495; 21389634; 21389261; 21383772; 21382175; 21378036; 21375706; 21374643; 21372393 |
| NT01AE4380 | 8 | 0.90 | 21400100; 21389620; 21389104; 21388532; 21385584; 21377525; 21355852; 21353756; 21346809; 21346101 |
| NT01AE4387 | 9 | 0.96 | 21397998; 21390227; 21370994; 21366529; 21362022; 21358050; 21357626; 21357487; 21333381; 21318901 |
| NT01AE4394 | 19 | 0.76 | 20976701; 20202763; 19762441; 17400891; 12397186; 18550550; 18342249; 17007421; 15961396; 15927751 |
| NT01AE4499 | 12 | 0.84 | 21394206; 21390501; 21389245; 21375693; 21365756; 21352228; 21309581; 21301927; 21256965; 21247063 |
| NT01AEA0065 | 18 | 0.87 | 21397717; 21369825; 21265778; 21059948; 20972419; 20947023; 20855615; 20855510; 20851903; 20847002 |
| NT01AEA0072 | 18 | 0.97 | 21399938; 21399583; 21398553; 21398536; 21398489; 21398480; 21397847; 21397696; 21397337; 21397298 |
| NT01AEA0076 | 18 | 1.00 | 21369825; 20855510; 20662775; 20408914; 20154136; 20132828; 10827169; 19715704; 19416927; 11805094 |
| NT01AEA0099 | 8 | 0.73 | 21178285; 20811135; 19966484; 19546514; 19424620; 19200041; 17291445; 16402358; 16221991; 15996793 |
| NT01AEB0109 | 8 | 0.73 | 21178285; 20811135; 19966484; 19546514; 19424620; 19200041; 17291445; 16402358; 16221991; 15996793 |
| NT01AL0317 | 8 | 0.83 | 20620948; 20131297; 19442247; 17693399; 18393679; 18464067; 18455306; 17616665; 15175153; 14534320 |
| NT01AL0621 | 4 | 0.87 | 18418076; 8943288; 12847084; 16494844; 2298810; 10411757; 12360284; 11973272; 11175772; 6321297 |
| NT01AL0737 | 19 | 0.88 | 21400235; 21388958; 21378160; 21367655; 21357626; 21342516; 21333648; 21332624; 21331044; 21307593 |
| NT01AL0787 | 15 | 0.78 | 21173231; 21124861; 20837997; 20149645; 4351805; 18660808; 19262507; 19155176; 18817723; 16557263 |
| NT01AL1423 | 8 | 0.72 | 15311941; 11178972; 10368269; 10329709; 9490068; 8199774 |
| NT01AM0022 | 8 | 0.92 | 21336929; 21317262; 21290824; 21197843; 21168410; 21146502; 21144833; 21132340; 21119085; 21117708 |
| NT01AM0050 | 18 | 0.88 | 21081547; 20881245; 20202763; 3162770; 19825636; 19697161; 19665072; 19577910; 18039771; 10931319 |
| NT01AM0085 | 8 | 0.88 | 20160912; 19996100; 16531404; 14644451; 12603319; 11955070; 11935326; 11781147; 11708858; 11673873 |
| NT01AM0296 | 9 | 0.97 | 20418430; 20178986; 8406042; 19664929; 19472174; 2580220; 19095065; 19083031; 17555433; 18824113 |
| NT01AM0446 | 15 | 0.98 | 21315771; 21284862; 21257771; 21217003; 21193607; 21078995; 21037181; 20951027; 20946847; 20946846 |
| NT01AM0906 | 18 | 0.93 | 20647000; 20217167; 20111865; 2285283; 17159924; 19416927; 16979625; 18533835; 12783268; 8300209 |
| NT01AM0995 | 17 | 0.91 | 9582370; 20429864; 20403971; 19309454; 15808855; 18434412; 6409691; 18386297; 18288197; 10551881 |
| NT01AM1042 | 14 | 1.00 | 20980241; 20831412; 11157898; 20082641; 20045992; 16322525; 19900465; 1644750; 17392337; 17919281 |
| NT01AM1089 | 8 | 0.73 | 21178285; 20811135; 19966484; 19546514; 19424620; 19200041; 17291445; 16402358; 16221991; 15996793 |
| NT01AO0344 | 19 | 0.86 | 21378199; 21369973; 21369910; 21357626; 21357429; 21324659; 21311881; 21307589; 21297160 |
| NT01AO0357 | 5 | 0.78 | 21031595; 20429505; 20030377; 19772975; 19006326; 18616587; 18351402; 18043616; 17975078; 17959596 |
| NT01AO1133 | 2 | 0.96 | 19754882; 19267692; 19154787; 16489629; 15522295; 14595395; 11215515; 12055304; 9689094; 10585141 |
| NT01AO1255 | 12 | 0.74 | 21399329; 21398603; 21398524; 21398495; 21398423; 21397980; 21397664; 21396985; 21396939; 21395509 |
| NT01AO1288 | 8 | 0.79 | 18367206; 9757107; 10320398; 10955993; 10433701; 1939137; 2174243; 6386468; 8241149; 8508789 |
| NT01AO1811 | 12 | 0.91 | 21403683; 21402859; 21402719; 21402511; 21402045; 21401044; 21399329; 21398603; 21398524; 21398495 |
| NT01AO1852 | 12 | 0.88 | 21392578; 21368744; 21357900; 21345977; 21345437; 21341174; 21338617; 21300545; 21298052; 21292998 |
| NT01AO1934 | 19 | 0.88 | 21393206; 21216228; 21183069; 20974151; 20961110; 20870765; 20849416; 20580675; 20347848 |
| NT01AO2355 | 8 | 0.79 | 21383078; 21067517; 20405931; 20303981; 20038140; 19845618; 7860587; 15269332; 10788346; 14521953 |
| NT01AO2375 | 8 | 0.90 | 20977213; 20628895; 20400563; 20381373; 20340124; 20202935; 20067522; 19584533; 18485344; 8385603 |
| NT01AO2491 | 8 | 0.90 | 20383014; 19397993; 10491097; 17704896; 15280650; 16917525; 16796018; 15928986; 12011980; 11825616 |
| NT01AO2797 | 1 | 0.73 | 20180123; 19471856; 19402116; 7901896; 18481057; 18210886; 18007551; 17589636; 17362731; 17139593 |
| NT01AP0074 | 12 | 0.81 | 21403392; 21391720; 21379584; 21374551; 21373891; 21364669; 21357544; 21349328; 21345219; 21338617 |
| NT01AP0126 | 6 | 0.76 | 21400534; 21398560; 21396975; 21396734; 21392732; 21388065; 21385603; 21380782; 21377274; 21377232 |
| NT01AP0136 | 2 | 0.84 | 21075928; 20861021; 20677756; 1532388; 19415239; 19368556; 3322275; 15572765; 10829079; 11602359 |
| NT01AP0203 | 14 | 0.94 | 21045288; 20874647; 20676924; 20473969; 20377130; 20066562; 20054825; 12209152; 19370069; 18202078 |
| NT01AP0260 | 18 | 0.71 | 20453143; 19118352; 12007399; 18312576; 3301822; 8162415; 12042068; 322281; 16181352; 10496909 |
| NT01AP0266 | 6 | 0.74 | 7558017; 6366519; 2184031; 8387143; 1741034; 2666673; 6329717; 3015599 |
| NT01AP0391 | 2 | 0.96 | 21368171; 21255426; 21231969; 21206014; 21142117; 21081498; 21072368; 21071388; 21038112; 20971904 |
| NT01AP0397 | 8 | 0.94 | 21403907; 21392498; 21387444; 21381897; 21364298; 21357464; 21357306; 21346867; 21335009; 21334407 |
| NT01AP0413 | 13 | 0.77 | 21399665; 21396915; 21395050; 21385875; 21383689; 21375771; 21368180; 21367972; 21349877; 21334712 |
| NT01AP0514 | 18 | 0.87 | 21282456; 21081547; 20855745; 20610168; 20573661; 20525733; 20508090; 20399647; 20111865; 20017731 |
| NT01AP0759 | 14 | 0.96 | 20859109; 20801890; 19765547; 19524543; 11827479; 12797828; 18602116; 18556440; 15163660; 18343960 |
| NT01AP0776 | 18 | 0.77 | 10581369; 15711318; 14523988; 11422294; 11162526; 10903843; 10564813; 10506149; 10381896; 9920925 |
| NT01AP0809 | 6 | 0.83 | 15656976; 12638184; 11737776; 11732635; 10915862; 8621533; 10666338; 1373853; 9784390; 8294433 |
| NT01AP0856 | 12 | 0.87 | 21349806; 21256111; 21254169; 21228234; 21224428; 21224036; 21204251; 21199564; 21270777; 21136954 |
| NT01AP0900 | 12 | 0.96 | 21395946; 21387162; 21383917; 21383194; 21383012; 21375947; 21364294; 21354219; 21352778; 21340536 |
| NT01AP0952 | 8 | 0.77 | 21216999; 20943182; 20847256; 20629071; 20595007; 20593880; 20460582; 20334431; 19932189; 19827797 |
| NT01AP1060 | 18 | 0.96 | 21085989; 20118641; 9787636; 19168988; 18702072; 18343670; 17251264; 16887318; 16751629; 16730200 |
| NT01AP1070 | 1 | 0.74 | 11708855; 16881711; 11259585; 18398873; 18266921; 17680699; 11119723; 17261589; 10393538; 7108955 |
| NT01AP1106 | 6 | 0.86 | 20081198; 11572866; 19664060; 16040599; 18655840; 9564050; 17556804; 12748298; 17286578; 16793392 |
| NT01AP1122 | 18 | 0.99 | 21118978; 20855510; 20643901; 20639324; 20408914; 20132828; 19594831; 8830274; 12775704; 19362642 |
| NT01AP1132 | 9 | 0.95 | 19550039; 19101548; 17906131; 15498941; 12406578; 8468280; 7883013; 1917873; 1527048; 1508151 |
| NT01AP1201 | 8 | 0.78 | 20661960; 19579240; 19115296; 18215050; 17927915; 2834341; 3276516; 9047371; 16302255; 15959893 |
| NT01AP1252 | 18 | 0.96 | 21369825; 21122131; 21111784; 21090806; 21071707; 21059948; 21059651; 20974818; 20955543; 20952574 |
| NT01AP1265 | 17 | 0.96 | 21164032; 21057108; 21046623; 20978146; 20952539; 20952386; 20870727; 20862319; 20844218; 20837538 |
| NT01AP1382 | 8 | 0.76 | 19374041; 18494623; 15028871; 12853152; 8214582; 10816601; 12515465; 11293413; 10048486; 9720302 |
| NT01AP1429 | 8 | 0.71 | 21371789; 21346092; 21323512; 21226315; 21222161; 21178087; 21163840; 21161404; 21141123; 21129192 |
| NT01AP1483 | 2 | 0.82 | 17548827; 12119022; 11939774; 9446573; 7929373; 8344936; 6312261 |
| NT01AP1496 | 18 | 0.96 | 21168419; 21149736; 21104926; 20876799; 20851982; 20823549; 20720015; 20693325; 20678502; 20668054 |
| NT01AP1542 | 8 | 0.82 | 17993624; 17981801; 15809865; 15666206; 9426612; 8830251; 8223576; 1688555 |
| NT01AP1635 | 18 | 0.91 | 21350158; 21265952; 21200028; 21135173; 21131949; 21130773; 21098712; 21097526; 21080215; 21063095 |
| NT01AP1735 | 2 | 0.93 | 18028398; 17174261; 15967475; 15614489; 11527707; 10891066; 10383756; 10075431; 9700068; 8885414 |
| NT01AP1814 | 18 | 0.91 | 21315686; 21311724; 21177413; 21122131; 21111784; 21090806; 21078855; 21071707; 21059948; 21059651 |
| NT01AS2535 | 15 | 0.82 | 21393212; 21386983; 21385724; 21383239; 21357299; 21347346; 21338417; 21330130; 21312005; 21310052 |
| NT01AS2562 | 8 | 0.75 | 21166461; 2144454; 2684979; 3308853; 3365097 |
| NT01AS2697 | 18 | 0.95 | 21367787; 21362118; 21352605; 21349178; 21324969; 21318884; 21310956; 21047224; 21302256; 21282431 |
| NT01AS2730 | 12 | 0.98 | 21397065; 21395067; 21394739; 21394468; 21392058; 21388873; 21388559; 21388136; 21387310; 21385872 |
| NT01AS2874 | 9 | 0.94 | 21393229; 21301088; 21166831; 21035728; 20818586; 20727773; 20698542; 20583783; 20519910 |
| NT01AS2914 | 4 | 0.80 | 20696876; 20618074; 20543056; 20487023; 20065060; 20056773; 19913366; 19900770; 19808235; 16424339 |
| NT01AS2993 | 9 | 0.98 | 20547355; 18704089; 16707707; 9250661; 18702504; 17609944; 16061252; 15751987; 15638818; 15380819 |
| NT01AS2998 | 4 | 0.81 | 21178435; 21046450; 21040514; 21038480; 20876224; 20607764; 20446763; 20416426; 20118250; 20109152 |
| NT01AS3063 | 9 | 0.94 | 21400110; 21398697; 21397942; 21397586; 21396142; 21393852; 21393244; 21392972; 21392566; 21392564 |
| NT01AS3133 | 8 | 0.97 | 21403907; 21400553; 21397011; 21392546; 21392498; 21387444; 21381897; 21374662; 21370474; 21364298 |
| NT01AS3191 | 8 | 0.85 | 15193308; 17854388; 16689938; 10564790; 8161284; 7814412; 8444151; 8297211; 189692; 1784339 |
| NT01AS3217 | 15 | 0.94 | 21338611; 21151646; 21149585; 20729367; 20711606; 20685939; 10850980; 20139635; 20086039; 20049751 |
| NT01AS3326 | 18 | 0.91 | 21288822; 21276099; 21226332; 21209370; 21187898; 21175590; 21168419; 21134393; 21134356; 21130497 |
| NT01AT0239 | 8 | 0.74 | 16292556; 10947204; 10686279; 9787093; 9590057; 9590035; 6546423; 7609453; 3117077; 6418146 |
| NT01AT1028 | 18 | 0.78 | 19236456; 10644771; 10500249; 9295337; 8021279; 7932715; 8130334; 1365829 |
| NT01AT1294 | 18 | 0.94 | 17071331; 2840346; 9704093; 15203903; 12217875 |
| NT01AT1478 | 11 | 0.93 | 19503942; 765483; 11452024; 16956798; 16273039; 16125724; 12467643; 11823865; 11095248; 10756710 |
| NT01AT1728 | 18 | 0.99 | 21168419; 20154136; 20132828; 15673787; 17322901; 17171468; 11756427; 16445940; 16135226; 6051349 |
| NT01AT2182 | 12 | 0.94 | 2248769; 10693756; 17051148; 16321976; 12225666; 12214059; 11793244; 11672525; 8915840 |
| NT01AT2469 | 15 | 0.98 | 21315771; 21284862; 21257771; 21217003; 21193607; 21078995; 21037181; 20951027; 20946847; 20946846 |
| NT01AT2699 | 8 | 0.73 | 18323095; 18515344; 9175718; 8554504; 9210251; 9203161; 9013822; 8876969; 2171428; 2753874 |
| NT01AT2893 | 18 | 0.97 | 21385839; 21347707; 21343424; 21151096; 21150129; 21123563; 21104370; 21044049; 20936698; 20860483 |
| NT01AT3152 | 2 | 0.92 | 21367973; 21335095; 21315197; 21268892; 21217003; 21214021; 21148731; 21117240; 21071847; 21029241 |
| NT01AT3345 | 2 | 0.93 | 21288652; 21247799; 20649840; 20484298; 20382023; 19197387; 19507290; 19490098; 19167902; 1754394 |
| NT01ATA0161 | 18 | 0.93 | 21122131; 21090806; 20955543; 20870774; 17302441; 20382765; 20233492; 20154136; 20004175; 10839820 |
| NT01ATA0414 | 18 | 0.99 | 21168419; 20888343; 20808924; 7500034; 20203106; 20154136; 20132828; 18535149; 19400778; 19056738 |
| NT01ATA0494 | 4 | 0.76 | 21375141; 21083620; 21030434; 20822309; 20501965; 20450002; 19912343; 19747126; 19722842; 19702960 |
| NT01ATA1671 | 9 | 0.93 | 20725044; 17570335; 12121720; 14997352; 14766918; 9973333; 12048195; 10470036; 10589718; 8868440 |
| NT01ATC0321 | 8 | 0.85 | 18510336; 7778974; 15242297; 14630048; 11114944; 2971647; 11165359; 8245826; 10744688; 8254303 |
| NT01ATC0365 | 18 | 0.96 | 21276099; 21209370; 21187898; 21175590; 21134356; 21122131; 21090806; 20959127; 20956569; 20955543 |
| NT01ATC0589 | 15 | 0.98 | 20946846; 20817745; 20148929; 20127467; 19411418; 17183208; 6550579; 12614149; 17908933; 7989325 |
| NT01ATC0732 | 6 | 0.72 | 21369766; 21319196; 21293478; 21266827; 21122797; 20966874; 20926398; 20735435; 20670616; 20652500 |
| NT01BA0171 | 8 | 0.92 | 21254627; 21057504; 20980952; 20974925; 20971856; 20959113; 20926707; 20858599; 20858453; 20856881 |
| NT01BA0173 | 8 | 0.88 | 20116362; 20059731; 20049706; 20025615; 19716389; 19520722; 19364480; 19097788; 18956753; 18849900 |
| NT01BA0242 | 4 | 0.95 | 20870765; 20807205; 20644139; 20363951; 17326815; 2002065; 19635793; 8181761; 18394147; 12634424 |
| NT01BA0353 | 12 | 0.91 | 20945359; 20419408; 20170640; 20146530; 11101515; 10679227; 19569227; 16179007; 10698927; 11597451 |
| NT01BA0358 | 4 | 0.75 | 10785634; 17492271; 14617189; 15817382; 12940991; 1937792; 10320579; 9302021; 8200538 |
| NT01BB0413 | 12 | 0.73 | 21080085; 20558052; 20557879; 1577847; 20392990; 19838948; 19755172; 19729827; 19461054; 19224338 |
| NT01BB0665 | 15 | 0.94 | 20718955; 12236604; 15528647; 11310739; 10439393; 371775; 1545709; 1840643; 2695393 |
| NT01BB0675 | 14 | 0.87 | 20813786; 19935661; 16333637; 16114032; 14506266; 12630318; 11441057; 16665292; 3038334; 10506947 |
| NT01BB0890 | 18 | 0.82 | 20519548; 19711043; 19629169; 19524532; 19217392; 19217392; 12529635; 18628306; 8345525; 17878235 |
| NT01BB1076 | 9 | 0.91 | 20590527; 20178986; 8406042; 7045078; 19686777; 19095065; 18977209; 18691538; 18569341; 18201766 |
| NT01BB1111 | 15 | 0.94 | 19830761; 3507689; 11059492; 8704152; 3162770; 8152373 |
| NT01BB1350 | 12 | 0.79 | 8087850; 6384729; 8462840; 2679887; 8387148; 1588819; 2088168; 2041472; 2170107; 2249673 |
| NT01BB1385 | 2 | 0.94 | 18154309; 15632135; 15269205; 15247236; 15159566; 12583917; 12504674; 12006571; 11554796; 11428898 |
| NT01BB1513 | 8 | 0.82 | 18025549; 12925808; 12821154; 12596860; 240767; 3275662 |
| NT01BB1530 | 18 | 0.80 | 21097626; 21059656; 20970503; 20705129; 20558583; 10986670; 19906183; 19664604; 12829268; 19576903 |
| NT01BB1531 | 15 | 0.91 | 12236604; 10049400; 10564505; 10439393; 371775; 9781878; 9503622; 1444258; 2201955; 8022282 |
| NT01BB1726 | 15 | 0.75 | 21068394; 20659289; 20562308; 21261917; 18005413; 18331470; 1715407; 17711452; 10714982; 10982867 |
| NT01BB1994 | 4 | 0.76 | 18224398; 4360948; 12122010; 9696767; 1622933 |
| NT01BB2092 | 2 | 0.97 | 19818021; 18959753; 17686778; 7047497; 15306815; 16205910; 15632135; 15269205; 15247236; 12719427 |
| NT01BB2465 | 12 | 0.90 | 20393890; 20061471; 19639232; 12082169; 12686301; 16937283; 9843504; 15333588; 14643002; 12833515 |
| NT01BB2806 | 18 | 0.80 | 20192961; 19335424; 10879525; 18041813; 14559180 |
| NT01BB3171 | 19 | 0.73 | 14769872; 15518577; 14529267; 8987972; 8961949; 8672489; 8672475 |
| NT01BB3348 | 8 | 0.83 | 17618087; 16508165; 12410826; 11429466; 7926834 |
| NT01BB3594 | 15 | 0.85 | 20718955; 20219606; 19728107; 10811905; 10515934; 16140031; 15055769; 15690348; 7925310; 11932447 |
| NT01BB3608 | 8 | 0.82 | 20970504; 20924576; 20818520; 20383024; 16876450; 16133321; 856801; 10482501; 10648513; 10952301 |
| NT01BB3652 | 8 | 0.78 | 19801601; 18239430; 17787008; 17684016; 17137300; 16260293; 16119842; 14706841; 14655000; 10482501 |
| NT01BB3671 | 12 | 0.98 | 21095572; 21075926; 12382110; 20566871; 20419406; 20419402; 20417202; 20207389; 20185723; 20100489 |
| NT01BB3734 | 15 | 0.80 | 21345178; 20874755; 20068355; 10745001; 9511756; 16359331; 1677357; 15713883; 12753201; 2557454 |
| NT01BB3946 | 18 | 0.99 | 21345797; 21344946; 21336875; 21107372; 21059948; 20959448; 20959127; 20937813; 20888473; 20888319 |
| NT01BB4022 | 12 | 0.75 | 9933597; 9931490; 9882712; 9409753; 9141502; 8621556; 8021926; 8203164; 8509389; 1537805 |
| NT01BB4051 | 17 | 0.83 | 20453093; 19458654; 14636572; 12730324; 16469698; 10564483; 15561138; 377280; 11703665; 11495995 |
| NT01BB4248 | 5 | 0.89 | 20207756; 10194322; 10368287; 14749331; 11015224; 7565414; 10403130; 9524276; 3558364; 7565414 |
| NT01BB4373 | 4 | 0.77 | 10692373; 10409682; 10048040; 15165230; 16416128; 15752189; 11544226; 11763238; 11430412; 10819322 |
| NT01BB4394 | 15 | 0.91 | 15937186; 3038334; 8830274; 7816030; 8078459; 1714035; 1094240; 6381964; 3054125; 2456446 |
| NT01BB4409 | 19 | 0.77 | 21382014; 21298035; 19797705; 10571240; 19232405; 19168951; 19133276; 10319815; 18835273; 17588582 |
| NT01BB4470 | 8 | 0.83 | 18825405; 17936114; 17853358; 17261587; 15720402; 12480900; 11527960; 10495709; 2080068; 3132906 |
| NT01BB4629 | 8 | 0.92 | 21253866; 21036145; 10672375; 20160912; 20022530; 19464573; 19383527; 17532339; 9324032; 17667915 |
| NT01BB4897 | 6 | 0.96 | 21270834; 20633769; 8851040; 17965588; 16713978; 15786493; 1303259; 12408864; 12101424; 9606182 |
| NT01BC0150 | 18 | 0.77 | 21216906; 21123754; 21072985; 20966097; 20714501; 11972039; 20589834; 20511716; 10785634; 20202763 |
| NT01BC0353 | 11 | 0.96 | 21205308; 21176936; 21097580; 21079776; 20954012; 20802084; 20709082; 20662890; 20224894; 16595014 |
| NT01BC0483 | 12 | 0.72 | 21220484; 21123754; 21109561; 20876432; 7968073; 20580496; 20467255; 20385758; 20217167; 20212185 |
| NT01BC0544 | 8 | 0.72 | 21078123; 21073854; 19616102; 19589965; 19307254; 19191964; 18765916; 18390572; 17188300; 17146529 |
| NT01BC0556 | 4 | 0.80 | 16778019; 10931886; 20507476; 16696176; 16496698; 10471709; 11111904; 1126938; 9272861; 7483850 |
| NT01BC0691 | 4 | 0.78 | 21378181; 12721630; 12101297; 11964118; 10086841 |
| NT01BC0752 | 18 | 0.77 | 20883740; 15659379; 20031922; 17256083; 16632906; 7190579; 16044265; 14729147; 14713188; 12761179 |
| NT01BC0770 | 12 | 0.74 | 20711794; 19170879; 14999401; 10749923; 16380269; 11114201; 2025413; 12939276; 12914946; 12886009 |
| NT01BC0983 | 13 | 0.98 | 15540167; 15528667; 12185248; 9733638; 3305487; 3856321; 3911025; 6376124; 6345153 |
| NT01BC1041 | 4 | 0.81 | 20580869; 19735749; 19573697; 19269568; 18802102; 9393812; 17259600; 10066533; 10586027; 12608714 |
| NT01BC1149 | 8 | 0.81 | 20037925; 18956756; 12702311; 10216875; 16496400; 16473032; 15502322 |
| NT01BC1181 | 4 | 0.78 | 15652293; 16384799; 12644503; 11222602; 10949920; 6786720 |
| NT01BC1772 | 6 | 0.90 | 21160097; 20663915; 19702328; 19001846; 4625747; 10716976; 16226853; 9780838; 14676427; 12774115 |
| NT01BC1963 | 8 | 0.92 | 21291916; 20952574; 20809899; 20676631; 20620150; 5420057; 20378648; 11290745; 20133360; 20093189 |
| NT01BC2236 | 6 | 0.77 | 21300638; 21190558; 21131491; 21118802; 21078407; 20724226; 20733069; 20639336; 15020458; 9214645 |
| NT01BC2237 | 12 | 0.74 | 18323453; 16386933; 16078697; 11238984; 15670163; 15000685; 12359093; 11948788; 1616713; 10588904 |
| NT01BC2280 | 12 | 0.92 | 21385872; 21383012; 21375706; 21369989; 21366963; 21365755; 21352460; 21352200; 21334441; 21334427 |
| NT01BC2321 | 1 | 0.96 | 19887726; 5325263; 2610349; 15961950; 15792811; 14756555; 11752249; 12660328; 12376828; 1496924 |
| NT01BC2518 | 11 | 0.78 | 4977981; 9514719; 6427474; 18377930; 17602902; 10656821; 10612727; 9806496; 8744563; 8973529 |
| NT01BC2919 | 9 | 0.95 | 16105701; 15711796; 15629687; 10433720; 1886522; 12324476; 10848999; 405386 |
| NT01BC3088 | 4 | 0.90 | 15281929; 10421756; 10521664; 2231712; 438126 |
| NT01BC3280 | 18 | 0.92 | 21081547; 20881245; 20202763; 20150239; 3162770; 12773059; 19825636; 19697161; 19665072; 18039771 |
| NT01BC4291 | 8 | 0.72 | 21398106; 21393225; 21345801; 21342105; 21302259; 21268631; 21225092; 21182010; 21168331; 21093499 |
| NT01BC4928 | 12 | 0.90 | 19013180; 18504625; 15049693; 17596826; 16183637; 14739250; 14698441; 14573600; 11472061; 11432789 |
| NT01BC5290 | 8 | 0.73 | 21141812; 21138528; 21029451; 20889970; 20705057; 20635418; 20592025; 20502966; 20429505; 20405215 |
| NT01BF0128 | 19 | 0.70 | 21396969; 21375716; 21341524; 21333724; 21331046; 21205637; 21326935; 21324191; 21317344; 21306444 |
| NT01BF0462 | 8 | 0.76 | 20823222; 19845869; 19656447; 18845277; 7629135; 12859407; 12556518; 12114526; 11929049; 9862501 |
| NT01BF0905 | 14 | 0.89 | 21205085; 16778368; 19775280; 11895911; 16491070; 18839780; 18773877; 18255096; 15324697; 17602053 |
| NT01BF0943 | 19 | 0.98 | 21117235; 20700743; 20197075; 20164147; 20045480; 20043969; 19884781; 19576307; 19428473; 18174188 |
| NT01BF0947 | 19 | 0.71 | 21335550; 21208504; 21194112; 21092141; 21085571; 21068446; 20940307; 20940044; 20702566; 20615089 |
| NT01BF0978 | 4 | 0.94 | 19788546; 18391964; 9495771; 17360038; 10648549; 9252185 |
| NT01BF1078 | 8 | 0.80 | 21221530; 20304968; 17804429; 16295523; 11222635; 10757980; 10561613; 10329019; 10101253; 10027965 |
| NT01BF1156 | 14 | 0.97 | 20851133; 20799866; 20427701; 20208582; 19740110; 19647757; 19478237; 19386496; 10801977; 18669260 |
| NT01BF1308 | 18 | 0.77 | 21386968; 21369991; 21369988; 21331094; 21327741; 21308845; 21306769; 21283549; 21278256; 21276440 |
| NT01BF1716 | 18 | 0.76 | 21308845; 21183598; 21117233; 21060243; 20976191; 20950448; 20934507; 20920237; 20820947; 2139617 |
| NT01BF1913 | 12 | 0.78 | 16051065; 14683426; 12111749; 12087102; 11170590; 9891971; 9519865; 9356240; 8806674; 8002015 |
| NT01BF2217 | 1 | 0.71 | 21264378; 21263039; 20675481; 20133363; 19596042; 19595734; 19342798; 18761051; 17425614; 17388807 |
| NT01BF2279 | 6 | 0.90 | 21388532; 21378185; 21365542; 21350762; 21343909; 21336027; 21325134; 21321231; 21216906; 21145896 |
| NT01BF2373 | 15 | 0.98 | 20946846; 20817745; 17183208; 6550579; 12614149; 17908933; 18048908; 10924135; 10500846; 17650500 |
| NT01BF2563 | 18 | 0.93 | 20647000; 20217167; 20111865; 2285283; 17159924; 19416927; 16979625; 18533835; 12783268; 8300209 |
| NT01BF2780 | 13 | 0.96 | 21285181; 21241052; 21115117; 20942128; 1625572; 20362064; 20179335; 20156976; 20059602; 7397478 |
| NT01BF2784 | 18 | 0.86 | 21369825; 21204689; 21168419; 21122131; 21090806; 21071707; 21059948; 21059651; 21037298; 20955543 |
| NT01BF2855 | 2 | 0.90 | 20955518; 20798996; 20543028; 20223208; 19646414; 9588025; 19568953; 19557521; 18846290; 18790730 |
| NT01BF3021 | 6 | 0.83 | 18929140; 18945840; 11296256; 11292780; 17107651; 16431373; 16361113; 16325764; 15989726; 15168598 |
| NT01BF3136 | 8 | 0.90 | 20400563; 20337711; 20085751; 2538471; 18485344; 19285953; 15189131; 18162174; 18074176; 17655961 |
| NT01BF3344 | 18 | 0.99 | 21357299; 21315686; 21182225; 21090806; 21089447; 21084795; 21081547; 21037109; 20974830; 20938527 |
| NT01BF3376 | 19 | 0.87 | 20858733; 20699399; 20590984; 20502893; 18292091; 20153640; 20014851; 19945280; 19923741; 19918887 |
| NT01BF4453 | 12 | 0.89 | 19864425; 18637841; 17141585; 16330176; 16272315; 16137693; 15467394; 15352382; 15187416; 12919329 |
| NT01BF4456 | 15 | 0.71 | 21388494; 21383171; 21355038; 21347346; 21338417; 21329791; 21322102; 21311748; 21282189; 21276270 |
| NT01BF4515 | 4 | 0.79 | 20854656; 20231275; 20218337; 19819087; 3679545; 19596862; 19500523; 19023039; 19136695; 18227266 |
| NT01BG0093 | 18 | 0.88 | 21187401; 21187088; 21179061; 21173275; 21172605; 21149736; 21060012; 21037553; 21031171; 21030403 |
| NT01BG0144 | 4 | 0.75 | 21390509; 21369489; 21363982; 21362288; 21358760; 21350342; 21349136; 21335384; 21334822; 21334427 |
| NT01BG0199 | 12 | 0.70 | 20599668; 19853572; 19804735; 16307111; 17766116; 18387365; 18339324; 17980516; 16834335; 16573693 |
| NT01BG0320 | 12 | 0.85 | 20450495; 11142374; 10196305; 18413314; 18584290; 17957113; 10485658; 16630058; 14526118; 10690410 |
| NT01BG0371 | 11 | 0.74 | 21177765; 20974536; 20514991; 19150986; 19771572; 19571721; 19165083; 19135482; 19026554; 18956682 |
| NT01BG0569 | 12 | 0.88 | 21360678; 21270901; 17619951; 20171112; 10322418; 19948822; 19879746; 18627868; 19697319; 19769784 |
| NT01BG0667 | 12 | 0.78 | 21269063; 21258250; 21246601; 21245096; 21187068; 21107839; 21037231; 20805362; 20667740; 20661135 |
| NT01BG0674 | 12 | 0.90 | 21360678; 21280121; 21270901; 21255413; 21205158; 21104721; 21072667; 20962594; 20926695; 20877006 |
| NT01BH0037 | 18 | 0.81 | 21394800; 21388232; 21364742; 21355614; 21355045; 21350490; 21339296; 21337355; 21323310; 21322478 |
| NT01BH0277 | 18 | 0.98 | 21210849; 21199020; 21127384; 21103974; 20846144; 20804728; 20712617; 20601550; 20598550; 20587391 |
| NT01BH0907 | 9 | 0.98 | 21094633; 20931591; 20924576; 20846931; 20353815; 20332534; 20237302; 20223213; 20110695; 20067470 |
| NT01BH1225 | 4 | 0.76 | 21398607; 21393613; 21393550; 21392571; 21392139; 21391817; 21390329; 21389637; 21389095; 21388954 |
| NT01BH1226 | 12 | 0.80 | 20032457; 11751634; 17504491; 17855452; 10569626; 16127432; 16109378; 15249048; 15195946; 15157086 |
| NT01BH1373 | 19 | 0.96 | 21097635; 21070413; 21040511; 20724636; 20708437; 20610395; 20829379; 20497501; 20497501; 20497501 |
| NT01BH1597 | 19 | 0.89 | 20188057; 18499663; 16891347; 15820665; 15552059; 15226299; 12758148; 11846551; 10521532; 9192738 |
| NT01BH1626 | 4 | 0.71 | 20507997; 20117010; 19770041; 19695338; 19135125; 15695811; 18005663; 17270289; 10940244; 1638630 |
| NT01BH2494 | 4 | 0.83 | 21398556; 21389112; 21378197; 21378181; 21352236; 21299880; 21232149; 21221837; 21208192; 21199192 |
| NT01BH3446 | 6 | 0.76 | 21394101; 21393072; 21392397; 21391904; 21390132; 21389894; 21389547; 21389348; 21389131; 21388532 |
| NT01BH3568 | 19 | 0.90 | 21396764; 21393568; 21392970; 21389598; 21388656; 21381086; 21378034; 21377658; 21377361; 21377353 |
| NT01BH3584 | 6 | 0.75 | 18366438; 15909989; 15530361; 10669611; 11412119; 10972842; 10572298; 10480949; 8702746; 8842771 |
| NT01BH3854 | 5 | 0.98 | 9988768; 10196134; 11811992; 11811991; 8655146; 9988767; 8900198 |
| NT01BJ0095 | 18 | 0.95 | 21369825; 21366542; 21041497; 21034832; 20888343; 20855510; 20808924; 20805402; 20662775; 20656493 |
| NT01BJ0668 | 2 | 0.83 | 21047120; 20959560; 20692224; 20458544; 18849445; 19491146; 19234759; 10075836; 18215430; 17375528 |
| NT01BJ1077 | 12 | 0.88 | 21118527; 20877571; 20714719; 20371367; 20363282; 20206694; 15659660; 19616577; 12615915; 19497371 |
| NT01BJ1842 | 3 | 0.96 | 21404359; 21115127; 20616068; 20444090; 20348257; 20199591; 19942854; 1683704; 19895819; 19820722 |
| NT01BJ1844 | 3 | 0.90 | 21404359; 21398554; 21398549; 21284804; 21265748; 21264317; 21199258; 21192786; 21112248; 21098028 |
| NT01BJ2579 | 15 | 0.90 | 20056196; 19882243; 19601916; 17001076; 14600241; 10864496; 1377899; 2851488; 1346263; 2573733 |
| NT01BJ3684 | 18 | 0.91 | 21204932; 21074515; 20930480; 20921220; 20876538; 20695528; 20507120; 20401926; 20226783; 20025662 |
| NT01BJ4371 | 15 | 0.88 | 20236932; 19400806; 18607790; 10830969; 11320135; 15670593; 15590657; 13129621; 11988525; 10708580 |
| NT01BJ5763 | 18 | 0.89 | 21397696; 21389119; 21287570; 21203343; 21134354; 21104926; 21103971; 21103968; 21103967; 21070944 |
| NT01BJ6771 | 18 | 0.97 | 21383162; 21354532; 21168419; 21143427; 21118978; 21091503; 21073695; 21051487; 21040305; 20971898 |
| NT01BJ8347 | 12 | 0.89 | 21397660; 21397653; 21395069; 21394206; 21393250; 21391210; 21390501; 21390217; 21388519; 21385063 |
| NT01BJ9927 | 8 | 0.92 | 21404260; 21401388; 21400100; 21397737; 21396889; 21396131; 21394044; 21393444; 21393246; 21393237 |
| NT01BL0016 | 12 | 0.79 | 19393241; 15371479; 11724574; 10801886; 10344253 |
| NT01BL0369 | 12 | 0.97 | 11750807; 7761092; 16361710; 9015299; 10412982 |
| NT01BL1266 | 15 | 0.72 | 21220736; 21095445; 20962268; 20837138; 20803129; 20797529; 20726779; 20724523; 20713918; 20713515 |
| NT01BM0171 | 18 | 0.94 | 21349151; 20826817; 20737472; 20716818; 20616104; 20550927; 17382878; 20463659; 20395270; 20307498 |
| NT01BM0903 | 2 | 0.84 | 20038586; 19267692; 18281969; 16907720; 15522295; 14595395; 11215515; 12196144; 11750128; 7723011 |
| NT01BM1763 | 19 | 0.94 | 21056982; 20871101; 20870764; 20805402; 12740933; 20561581; 20410291; 8862584; 20061477; 19798745 |
| NT01BMA0190 | 4 | 0.88 | 21382340; 21273249; 21273248; 16166542; 18842098; 1711027; 17887963; 16091049; 7984417; 8330068 |
| NT01BMA1051 | 8 | 0.76 | 20739284; 20693675; 19566721; 11976112; 15899413; 15158259 |
| NT01BMA1159 | 8 | 0.87 | 15225600; 15028871; 8214582; 10816601; 11881834; 11293413; 9720302; 8243476; 2170125 |
| NT01BQ0011 | 6 | 0.82 | 21362551; 21338660; 21303393; 21243721; 21227473; 21189471; 21086481; 21052759; 21045305; 20978746 |
| NT01BQ0059 | 12 | 0.94 | 21352096; 21348480; 21343292; 21258033; 21233422; 21229878; 21189019; 21140098; 21130903; 21110709 |
| NT01BQ0069 | 15 | 0.96 | 21315771; 21284862; 21217003; 21216906; 21193607; 21078995; 21037181; 20979345; 20951027; 20946856 |
| NT01BQ0244 | 13 | 0.97 | 21159796; 18421856; 16678201; 10026269; 9585521 |
| NT01BQ0275 | 8 | 0.73 | 21116622; 21081062; 21071492; 20971847; 20952576; 20937896; 20802042; 20798065; 20705660; 20697695 |
| NT01BQ0603 | 8 | 0.91 | 20655923; 20160912; 18398875; 17923481; 17157320; 15082001; 14992577; 14644451; 12761172; 11955070 |
| NT01BQ0647 | 19 | 0.81 | 19754149; 7961456; 16755996; 11679076; 10092655; 1732206; 10092655; 9197543; 9099672; 8662613 |
| NT01BQ0648 | 8 | 0.97 | 20837989; 20833713; 20806931; 20568895; 20173089; 20149621; 20082374; 19571038; 19437978; 18986377 |
| NT01BQ0825 | 9 | 0.96 | 15539300; 10960477; 8388033; 3013315; 6067194; 6824716; 7236695 |
| NT01BQ0843 | 8 | 0.94 | 21398106; 21393861; 21392547; 21390523; 21387444; 21387033; 21376957; 21366264; 21364629; 21358763 |
| NT01BQ1100 | 13 | 0.70 | 21368151; 21264994; 21051506; 20852270; 20817755; 20678576; 12032344; 20184321; 20067766; 9149153 |
| NT01BQ1176 | 5 | 0.88 | 21386987; 21372632; 21358122; 21354142; 21318889; 21318880; 21318879; 21318878; 21312326; 21303959 |
| NT01BQ1442 | 2 | 0.81 | 21042033; 19197387; 19438713; 13990617; 14684885; 2268373; 18356241; 1141859; 16497163; 16170809 |
| NT01BQ1573 | 18 | 0.86 | 21362202; 21296666; 21273404; 21258766; 21043032; 20920577; 20815976; 20808673; 20709983; 20691752 |
| NT01BS0038 | 4 | 0.75 | 21037003; 20802044; 1347044; 19587469; 19497328; 18761709; 18208527; 3029030; 15715676; 15381338 |
| NT01BS0495 | 12 | 0.91 | 21403683; 21402859; 21402719; 21402511; 21402045; 21401044; 21399329; 21398603; 21398524; 21398495 |
| NT01BS0641 | 12 | 0.90 | 21390130; 21386517; 21375771; 21354421; 21344627; 21322055; 21300879; 21282582; 21276852; 21269272 |
| NT01BS0669 | 6 | 0.77 | 21300638; 21190558; 21131491; 21118802; 21078407; 20724226; 20733069; 20639336; 15020458; 9214645 |
| NT01BS0813 | 18 | 0.75 | 19201869; 10464268; 16790421; 16301646; 14759738; 10644735; 10318843; 9677430; 9401921; 8022784 |
| NT01BS0964 | 6 | 0.88 | 21323543; 21302907; 21251613; 21212468; 21167174; 21156276; 21148036; 21145476; 21095589; 21076780 |
| NT01BS1040 | 8 | 0.80 | 18786405; 18433622; 15694381; 15667300; 1280998; 12627946; 12586421; 11751865; 12101220; 11751865 |
| NT01BS1081 | 15 | 0.93 | 21335452; 19526422; 16267314; 10368139; 10939244 |
| NT01BS1086 | 19 | 0.74 | 21400688; 21400235; 21398593; 21393228; 21392988; 21390508; 21390257; 21388958; 21388880; 21388433 |
| NT01BS1980 | 18 | 0.81 | 16945692; 17517373; 17417881; 16750162; 1505735 |
| NT01BS2225 | 19 | 0.96 | 21030539; 20707404; 20201406; 19279143; 19883124; 19825675; 2125350; 19414810; 18237819; 12582168 |
| NT01BS2518 | 19 | 0.88 | 21183069; 20547785; 20497333; 20308379; 20194704; 19736537; 17646652; 19366363; 18809265; 1592809 |
| NT01BS2545 | 18 | 0.72 | 20663878; 20437261; 20060908; 10811910; 9482716; 18271245; 18187053; 10611227; 15840809; 15918885 |
| NT01BS2981 | 13 | 0.75 | 20606270; 1325384; 17623669; 19279227; 12717624; 18193200; 16890019; 8206848; 10076042; 15716138 |
| NT01BS3278 | 5 | 0.79 | 17308574; 10206712; 11741847; 11038360; 15272571; 11390694 |
| NT01BS3347 | 19 | 0.79 | 19528283; 1744050; 16218944; 15044829; 8953214 |
| NT01BS3551 | 15 | 0.95 | 20543043; 11751817; 9585513; 9988472; 11435478; 2116363; 10994983; 10972833; 8117074; 149110 |
| NT01BS3836 | 19 | 0.85 | 20720312; 19118632; 17452789; 16151197; 12070074; 9235953; 8355611 |
| NT01BS4503 | 15 | 0.86 | 21219466; 17994770; 17628151; 17499766; 10745001; 10331874; 16030236; 15306019; 1482126 |
| NT01BT0109 | 5 | 0.73 | 19073607; 10713131; 2463635; 12557188; 8425191; 3080424 |
| NT01BT0663 | 8 | 0.92 | 20400563; 10766431; 16677314; 15797248; 15496591; 15135079; 14669076; 12553798; 12484761; 11713686 |
| NT01BT1083 | 9 | 0.90 | 20879029; 20192961; 20058052; 15514001; 17032652; 12536298; 15337759; 11240125 |
| NT01BT1963 | 8 | 0.75 | 19478450; 18353294; 12368439; 14642815; 11999422; 5432063; 11073941; 7507269; 10990025; 10959081 |
| NT01BT2219 | 19 | 0.97 | 20798166; 20493247; 19517808; 19478450; 18359051; 18318839; 17955189; 17681998; 16914364; 16171396 |
| NT01BT2259 | 8 | 0.90 | 20400563; 20337711; 20085751; 2538471; 18485344; 19285953; 15189131; 18162174; 18074176; 17655961 |
| NT01BT2263 | 8 | 0.75 | 20519568; 20447408; 20299676; 19942659; 19923219; 19859980; 19850285; 18795799; 12206759; 18428423 |
| NT01BT2265 | 8 | 0.70 | 21220625; 21193572; 21037180; 20952114; 20887713; 20876192; 20852032; 20844868; 20829361; 20826812 |
| NT01BT2739 | 19 | 0.83 | 15292578; 10464298; 4563441; 8486688; 1826463 |
| NT01BT2946 | 6 | 0.96 | 20823514; 20724832; 20699270; 20628060; 20575528; 20434457; 20410075; 20377204; 20230692; 20088964 |
| NT01BT3212 | 19 | 0.87 | 21193067; 16926507; 12052060; 11853483; 11267646; 9511752; 7515357 |
| NT01BT3399 | 6 | 0.87 | 17660407; 16740122; 16684535; 16038930; 15948708; 15254896; 12917398; 3074010; 10438614; 10490383 |
| NT01BT4643 | 9 | 0.99 | 19330542; 18691575; 11025668; 8892527; 6305424 |
| NT01BW1205 | 19 | 0.77 | 21158480; 21118988; 21104698; 20871989; 20669918; 20552260; 20522493; 20444687; 20227065; 20159465 |
| NT01BW2624 | 2 | 0.86 | 21367973; 19013460; 18163882; 18043855; 16810234; 11795479; 11535779; 11435118; 11419946; 2271518 |
| NT01BW3388 | 8 | 0.81 | 19428350; 16897483; 15623532; 9784233; 7770449; 7948919 |
| NT01BW4622 | 13 | 0.94 | 18824261; 18765894; 16982740; 10902565; 10093218 |
| NT01BW4961 | 9 | 0.91 | 21388804; 21378051; 21351250; 21049984; 20658340; 20630605; 20503262; 20416968; 20410100; 20301160 |
| NT01BW5447 | 12 | 0.93 | 21395696; 20584115; 20181846; 19783165; 19307770; 11136446; 18488627; 17681481; 12765833; 16607952 |
| NT01BX0069 | 2 | 0.81 | 21307574; 20516609; 19111641; 18388403; 16824480; 15502327; 15226311; 15047724; 12005047; 10705982 |
| NT01BX0549 | 8 | 0.85 | 17449009; 2271232; 8645313; 8300532; 16345266 |
| NT01BX0552 | 8 | 0.94 | 21311752; 20614849; 20547844; 20490713; 20080351; 20036412; 9752724; 19623929; 19595660; 19303470 |
| NT01BX0622 | 8 | 0.90 | 21402210; 20041643; 19746965; 18804699; 17760503; 12102556; 15280650; 16466742; 15344930; 3009411 |
| NT01BX1258 | 12 | 0.78 | 21393425; 21360678; 21354993; 21348431; 21332026; 21308747; 21288162; 21246635; 21218781; 21210099 |
| NT01BX1464 | 8 | 0.97 | 21393249; 21163947; 20616209; 20434969; 19943898; 19899738; 19562840; 19212391; 2363500; 18406615 |
| NT01BX1601 | 9 | 0.99 | 16430210; 12893945; 14728675; 11524729; 10869041; 10212238; 15316083; 15146484; 14752098; 14577613 |
| NT01BX1645 | 8 | 0.80 | 21367571; 20813141; 18216065; 16919403; 16315011; 16233666; 9916801; 12906115; 12378587; 11841569 |
| NT01BX1724 | 8 | 0.82 | 17336131; 10766745; 12127981; 11494316; 3292726 |
| NT01BX1842 | 8 | 0.87 | 21068394; 21051545; 21029046; 20978135; 20932062; 20889970; 20737532; 20652619; 20606288; 20558724 |
| NT01BX2180 | 8 | 0.94 | 17891922; 16762453; 10581550; 15604729; 15164997; 15056475; 14555654; 12875742; 12619155; 11358527 |
| NT01BX2459 | 8 | 0.88 | 15667938; 11846565; 11243797; 10333487; 1995341; 2643092 |
| NT01BX2740 | 6 | 0.77 | 21085632; 8986766; 19918932; 18039855; 18497818; 18769921; 15723711; 18396468; 11376695; 18176792 |
| NT01BX2798 | 9 | 0.89 | 8082824; 20013982; 19954230; 19777301; 19733180; 18451049; 18319060; 11036020; 17237222; 16793520 |
| NT01BX2954 | 8 | 0.92 | 19636558; 8890739; 17083668; 12191769; 12899636; 12482588; 9724528; 12196158; 11502169; 11442063 |
| NT01BXA0197 | 8 | 0.91 | 21185288; 20807532; 20673834; 20543059; 20511510; 20498375; 20400563; 20340124; 20221534; 20154103 |
| NT01BXA0341 | 8 | 0.89 | 20920594; 19825426; 19304791; 19048328; 7248267; 7678352; 17241123; 17045293; 10788346; 16630630 |
| NT01BXA0655 | 6 | 0.83 | 21036838; 20739480; 19556570; 19139811; 17207152; 11014263; 2839109; 4091534 |
| NT01BXA0698 | 9 | 0.98 | 20043225; 18214656; 17996401; 17708576; 16380223; 16284748; 15908751; 15249192; 15033536; 14673522 |
| NT01CA0237 | 6 | 0.92 | 21399697; 21357694; 21344755; 21317926; 21281628; 21278727; 21222484; 21193302; 21190322; 21187477 |
| NT01CA0669 | 18 | 0.89 | 21358271; 21339577; 21315799; 21310270; 21308399; 21288822; 21170880; 21170874; 21271856; 21268855 |
| NT01CA1103 | 12 | 0.92 | 21385872; 21383012; 21375706; 21369989; 21366963; 21365755; 21352460; 21352200; 21334441; 21334427 |
| NT01CA1125 | 19 | 0.91 | 20047819; 11535797; 10986464; 9453150; 10419503; 2066344; 9174213; 2118867; 7379792 |
| NT01CA1138 | 8 | 0.79 | 20628895; 19888992; 16780790; 18293927; 17188684; 16969669; 16677314; 16645316; 15811511; 15221484 |
| NT01CA1275 | 6 | 0.91 | 10600744; 12667053; 11955076; 11810227; 11804608; 10377891; 6224021; 3025722; 3023634; 9062700 |
| NT01CA1697 | 19 | 0.97 | 21193828; 20562284; 11856846; 10217435; 9753433; 8612648 |
| NT01CA1731 | 19 | 0.95 | 20865175; 20210661; 11320139; 15687380; 18173801; 17426021; 12468728; 15032825; 14997492; 11748726 |
| NT01CA1856 | 15 | 0.82 | 21357744; 21338417; 21310052; 21278922; 21252224; 21249136; 21207455; 21199371; 21193612; 21192786 |
| NT01CA2047 | 8 | 0.79 | 20831589; 20504979; 20139414; 19919002; 19624733; 11528392; 19465084; 16405730; 19279361; 19238379 |
| NT01CA2087 | 11 | 0.80 | 12646614; 15808743; 17506728; 10502728; 12519996; 7685997; 15979387; 15777728; 15744573; 10645945 |
| NT01CA2550 | 19 | 0.72 | 20032185; 18760362; 18342249; 16368098; 16203204 |
| NT01CA2666 | 15 | 0.75 | 17085583; 15374661; 11980491; 7607244; 8365476; 3691501 |
| NT01CA2922 | 19 | 0.98 | 20826344; 20708437; 20682344; 20643653; 20571035; 20522493; 20068354; 19807868; 19807181 |
| NT01CA3062 | 4 | 0.75 | 20639339; 12470264; 10837475; 10084122; 7542800; 2201574; 8112597; 5432063; 1905718 |
| NT01CA4017 | 15 | 0.89 | 20932844; 9466251; 20498088; 20231408; 20196071; 20146748; 19850005; 1630316; 19400808; 19101563 |
| NT01CAA0057 | 3 | 0.92 | 21398553; 21385202; 21375257; 21364296; 21349988; 21327475; 21310062; 21282975; 21265756; 21258149 |
| NT01CAA0174 | 12 | 0.79 | 20842817; 20677810; 20451518; 20376786; 3878707; 20105403; 12778127; 17651389; 18756517; 18450759 |
| NT01CAA0183 | 17 | 0.83 | 21317330; 21131491; 20624215; 19715148; 18812514; 18631157; 19452595; 19439445; 19019196; 17090527 |
| NT01CD0024 | 19 | 0.96 | 21378160; 21365786; 21339299; 21333726; 21332624; 21331585; 21313853; 21310393; 21307368; 21306246 |
| NT01CD0165 | 19 | 0.91 | 21361872; 21356201; 21307941; 21284263; 21148159; 21059201; 20953949; 20942953; 20932078; 20889748 |
| NT01CD0170 | 19 | 0.76 | 21385362; 21344374; 21080432; 20946627; 20846498; 20798522; 20572857; 20491945; 20197185; 20156260 |
| NT01CD0331 | 18 | 0.88 | 21385839; 21381019; 21378197; 21378181; 21369424; 21357901; 21354347; 21347707; 21343931; 21343424 |
| NT01CD0426 | 8 | 0.71 | 18092946; 11827481; 10958932; 9512715; 9489669; 8805587; 8634242; 7929373; 1326547; 1313012 |
| NT01CD0599 | 4 | 0.80 | 21390252; 21390126; 21383206; 21370283; 21370068; 21368822; 21368055; 21364632; 21355420; 21352907 |
| NT01CD0618 | 18 | 0.99 | 18039771; 19105722; 11356926; 18590228; 10706290; 15813727; 15480787; 15240840; 6095266; 12410826 |
| NT01CD0822 | 9 | 0.79 | 20511508; 19213219; 18281324; 15458418; 15235808; 10756203; 3136142; 11796109; 10944342; 10769118 |
| NT01CD0848 | 5 | 0.99 | 21281472; 21224284; 21188624; 21187059; 21123867; 20978009; 20946949; 10398147; 20621683; 20566371 |
| NT01CD1098 | 1 | 0.87 | 20847010; 20648599; 12631323; 15223311; 14629006; 12525491; 2422596 |
| NT01CD1274 | 12 | 0.80 | 19295650; 10593889; 11929547; 11591389; 11422364; 9857014; 9660752 |
| NT01CD1378 | 1 | 0.91 | 18260104; 16078071; 15596430; 15336409; 12906820; 12906831; 12624088 |
| NT01CD1643 | 19 | 0.79 | 17335103; 12589763; 11398928; 11124264; 14907713; 7926844; 2269304 |
| NT01CD1920 | 2 | 0.92 | 11180061; 18553504; 15716108; 11098133; 12137537; 10896663; 16578835; 7275937; 8294001; 8250898 |
| NT01CD2007 | 8 | 0.73 | 21178285; 20811135; 19966484; 19546514; 19424620; 19200041; 17291445; 16402358; 16221991; 15996793 |
| NT01CD2072 | 19 | 0.95 | 21183069; 20608745; 20497333; 19646181; 17646652; 19209901; 19063962; 10692378; 18408890; 10564478 |
| NT01CD2099 | 12 | 0.82 | 18093586; 10531249; 16325969; 9545337; 15377669; 12972178; 12919480; 11526112; 11390277; 11208991 |
| NT01CD2132 | 8 | 0.73 | 21178285; 20811135; 19966484; 19546514; 19424620; 19200041; 17291445; 16402358; 16221991; 15996793 |
| NT01CD2138 | 8 | 0.73 | 21178285; 20811135; 19966484; 19546514; 19424620; 19200041; 17291445; 16402358; 16221991; 15996793 |
| NT01CD2211 | 8 | 0.85 | 6336730; 11173520; 8520120; 7813883; 2222122 |
| NT01CD2212 | 1 | 0.87 | 20847010; 20648599; 12631323; 15223311; 14629006; 12525491; 2422596 |
| NT01CD2273 | 8 | 0.83 | 18579827; 17855635; 16971696; 17510911; 12117714; 17374725; 17332886; 16873929; 16873928; 10518522 |
| NT01CD2451 | 17 | 0.93 | 10666455; 18226598; 18604630; 10551881; 14592988; 16514143; 10371038; 15590678; 15304219; 7482698 |
| NT01CE0347 | 19 | 0.88 | 21376799; 21279381; 21278296; 21241472; 21229881; 21205103; 21196935; 21188072; 21174346; 21161258 |
| NT01CE0350 | 8 | 0.90 | 1445195; 18436321; 16633561; 16292529; 9165069 |
| NT01CE0765 | 12 | 0.73 | 15870397; 15218044; 15201200; 10454615; 10411901; 11756428; 11288717; 9514212; 10913914; 10679295 |
| NT01CE1439 | 9 | 0.98 | 21193572; 20534481; 20334431; 19395484; 16667981; 12893945; 18446411; 8300518; 10849007; 16828988 |
| NT01CE1688 | 2 | 0.83 | 21306229; 19068481; 17561959; 16042605; 15525640; 12869542; 12196155; 10777198; 8590279; 9331403 |
| NT01CE1753 | 8 | 0.91 | 21310052; 21300879; 21295699; 21228259; 21173572; 21153002; 21148288; 21098265; 21067517; 20977550 |
| NT01CE1788 | 8 | 0.84 | 20888212; 20688172; 20675294; 20518346; 20353187; 19943898; 19899738; 19751796; 19651103; 18388293 |
| NT01CE2386 | 2 | 0.75 | 20955688; 20593767; 20560533; 11180061; 10961912; 19129660; 19055484; 16666457; 18693754; 6134288 |
| NT01CE3347 | 9 | 0.71 | 21051554; 18167308; 1657935; 17850764; 17726007; 17675291; 16847310; 16620760; 16243729; 16150824 |
| NT01CE3659 | 6 | 0.92 | 16011798; 10518615; 9454710; 17148461; 10656812; 12942776; 8223468; 9795213; 10593256; 9493381 |
| NT01CE3724 | 19 | 0.74 | 18366074; 15680239; 8093697; 7902527; 8093697; 1355454; 1495475 |
| NT01CF0013 | 17 | 0.71 | 21326893; 21243353; 21189325; 21079674; 21075884; 21051490; 20979336; 20971916; 20955556; 20923236 |
| NT01CF0112 | 8 | 0.72 | 20297722; 19888992; 19826863; 17639348; 17506525; 18314963; 18300158; 17959596; 17893805; 17891922 |
| NT01CG0031 | 15 | 0.82 | 21398516; 21397356; 21396644; 21395887; 21395007; 21393861; 21387177; 21386087; 21385720; 21385331 |
| NT01CG0070 | 18 | 0.98 | 16857941; 16049012; 15713785; 15353332; 15240109; 14514697; 11430413; 10844693; 10423704; 9918676 |
| NT01CG0155 | 8 | 0.73 | 21178285; 20811135; 19966484; 19546514; 19424620; 19200041; 17291445; 16402358; 16221991; 15996793 |
| NT01CG0496 | 18 | 0.96 | 21367787; 21362118; 21352605; 21349178; 21324969; 21318884; 21047224; 21302256; 21282431; 21281657 |
| NT01CG0638 | 6 | 0.87 | 21393072; 21304489; 21271694; 21255115; 21251107; 21131907; 21131361; 21074452; 20977236; 20961081 |
| NT01CG0661 | 1 | 0.71 | 20924576; 20818520; 827241; 19798672; 16857674; 17922758; 17567742; 11495997; 17185548; 17031048 |
| NT01CG0697 | 1 | 0.94 | 20818520; 827241; 17922758; 17185548; 11807258; 13058835 |
| NT01CG1070 | 1 | 0.87 | 20847010; 20648599; 12631323; 15223311; 14629006; 12525491; 2422596 |
| NT01CG1346 | 6 | 0.89 | 15537632; 15049692; 9390555; 11790092; 11054459; 10882092; 10514496; 9526698; 9264032; 9215631 |
| NT01CG1383 | 13 | 0.99 | 20223217; 20160120; 19749248; 19737557; 19625150; 17953511; 19492814; 19285947; 19267673; 19201563 |
| NT01CG1460 | 2 | 0.93 | 10383756; 10075431; 8885414; 791939; 14217462 |
| NT01CG1680 | 12 | 0.77 | 21394826; 21383147; 21374984; 21353370; 21345219; 21342228; 21338338; 21325107; 21307284; 21303943 |
| NT01CG2020 | 13 | 0.88 | 20525789; 18384044; 15248782; 15165845; 14517984; 12504684; 11406626; 12499565; 11763972; 10094308 |
| NT01CG2238 | 6 | 0.81 | 20963614; 20686482; 20675469; 20591822; 20122937; 19733176; 19465049; 320003; 4565086; 18373065 |
| NT01CG2409 | 1 | 0.87 | 20847010; 20648599; 12631323; 15223311; 14629006; 12525491; 2422596 |
| NT01CG2414 | 1 | 0.97 | 20140469; 19428471; 17168900; 18285355; 18208521; 18184691; 18050920; 16535512; 17981822; 5277076 |
| NT01CG2561 | 2 | 0.73 | 11342140; 17826740; 10760138; 16554727; 16326705; 12657046; 16132864; 15581578; 10940244 |
| NT01CG2749 | 9 | 0.90 | 21248857; 20652826; 20622122; 20528952; 20086012; 20025994; 19898564; 19850005; 19656950; 11528392 |
| NT01CG3019 | 8 | 0.81 | 20826164; 20639368; 19839648; 19664062; 10515912; 17373777; 17353140; 16814740; 942051; 12577265 |
| NT01CJ1612 | 18 | 0.97 | 18799096; 11771758; 11557130; 10955852; 9034323 |
| NT01CK0016 | 8 | 0.70 | 21327819; 21073414; 20889740; 20889709; 20861021; 20858599; 20836848; 20697198; 20650894; 20593880 |
| NT01CK0131 | 12 | 0.73 | 21397184; 21364279; 21362486; 21324305; 21313852; 21304496; 21285396; 21285349; 21267636; 21246635 |
| NT01CK0220 | 13 | 0.91 | 4927947; 19620372; 19013277; 18523188; 17693713; 16803595; 15378702; 11342105; 11341315; 9810686 |
| NT01CK0440 | 6 | 0.81 | 3603026; 11029422; 12145201; 12742016; 8163161; 11916378; 11078647; 9813262; 9396829; 7988894 |
| NT01CK0536 | 12 | 0.77 | 20466747; 17526840; 10320580; 16861792; 9735342; 9240461; 8954128; 7811295 |
| NT01CK0591 | 4 | 0.79 | 20413065; 1445195; 18710024; 18436321; 18307039; 18299074; 18078731; 17369938; 17364433; 16967205 |
| NT01CK0814 | 8 | 0.90 | 20600565; 19443720; 17885090; 19081388; 16098460; 15581639; 12191769; 15292214; 15109257; 15078221 |
| NT01CK0846 | 8 | 0.74 | 21082361; 21067517; 20581474; 20405931; 20303981; 20038140; 10788346; 18179423; 16275737; 16151213 |
| NT01CK0993 | 18 | 0.96 | 20516589; 18270203; 18563621; 17206386; 15901729; 16718600; 15901729; 2055470 |
| NT01CK1604 | 17 | 0.85 | 21214942; 18669632; 10369763; 17891922; 16891062; 15476402; 15388715; 15143026; 12845423; 12381573 |
| NT01CK1874 | 4 | 0.71 | 19017496; 18957605; 17989942; 16168580; 15641784; 8429550; 12520362; 11688801; 9335544; 8879569 |
| NT01CK2619 | 5 | 0.84 | 21393052; 21338415; 21088107; 21046332; 21036337; 20965680; 20847585; 20797998; 20221733; 20221547 |
| NT01CK3583 | 8 | 0.80 | 21327819; 21159175; 21071046; 20889740; 20889709; 20861021; 20858599; 20843096; 20836848; 20831907 |
| NT01CKA0038 | 11 | 0.81 | 21185326; 21129204; 21129203; 20890845; 20889742; 20823229; 20662890; 10744977; 17900620; 19878744 |
| NT01CN0055 | 9 | 0.97 | 19550039; 17906131; 15668256; 9804793; 8075395 |
| NT01CN0481 | 19 | 0.91 | 20848588; 20729089; 20726530; 20595390; 14367272; 20179984; 19384899; 10575553; 11418550; 18599029 |
| NT01CN0614 | 5 | 0.84 | 12656626; 17845818; 16784786; 16495101; 15924422; 11781802; 11029694; 9102457; 8601431; 9305970 |
| NT01CN0859 | 6 | 0.81 | 20843803; 20732909; 19616486; 19542005; 15249553; 8706136; 11106395; 6997501; 12226667; 8234293 |
| NT01CN0901 | 14 | 0.98 | 21394111; 21366529; 21348499; 21307941; 21279421; 21197954; 21169694; 21126315; 21112359; 21091150 |
| NT01CN0921 | 19 | 0.88 | 21131496; 21097630; 21092259; 21061584; 21044320; 21041486; 20965319; 20923118; 20921143; 20889748 |
| NT01CN1212 | 18 | 0.94 | 21383000; 21362624; 21360610; 21356241; 21349697; 21348820; 21346156; 21343600; 21334314; 21333664 |
| NT01CN1338 | 2 | 0.77 | 20194361; 19919179; 17178720; 16606627; 16218869; 15967800; 15450488; 11578923; 12975365; 12114526 |
| NT01CN1388 | 6 | 0.79 | 17588338; 10863370; 12753365; 12425851; 11155162; 10665535; 10635221; 1629152; 1282354; 3017638 |
| NT01CN1612 | 12 | 0.96 | 21397717; 21378058; 21338918; 21338912; 21335977; 21326941; 21242965; 21231916; 21151970; 21109561 |
| NT01CN1659 | 2 | 0.72 | 18476984; 11153263; 10498773; 12547821; 2231712; 9753692; 9261082; 8916230; 8430515 |
| NT01CN1825 | 1 | 0.95 | 20137911; 19508381; 18243023; 18080813; 18069696; 17216463; 17176071; 17115705; 17087051; 15753077 |
| NT01CN1983 | 1 | 0.86 | 20381632; 17333167; 16983384; 12570844; 8919551; 11101437; 10508663; 9131624; 10222581; 9521647 |
| NT01CN2018 | 6 | 0.84 | 21390502; 21356311; 21343179; 21322094; 21315812; 21291352; 21254323; 21253737; 21235684; 21220122 |
| NT01CN2112 | 2 | 0.89 | 20405048; 9843405; 14757766; 11443125; 10781607; 10722656; 2231712; 1768149; 9632726; 7519593 |
| NT01CN2218 | 4 | 0.87 | 21112337; 17317110; 11016400; 1576406; 3162770 |
| NT01CN2251 | 15 | 0.93 | 16430694; 11101667; 10627039; 13890303; 8969513; 7746146; 8497200; 1846145 |
| NT01CN2312 | 6 | 0.78 | 21219854; 21205014; 21124948; 21079801; 20861182; 20813592; 20666462; 20628184; 20334433; 20223211 |
| NT01CP0058 | 6 | 0.74 | 20600108; 8164689; 16896524; 10486005; 1502852; 15537659; 15494304; 10378680; 10672022; 9697843 |
| NT01CP0159 | 6 | 0.76 | 21394101; 21390132; 21390131; 21388263; 21386365; 21385932; 21383063; 21383020; 21378982; 21378394 |
| NT01CP0347 | 13 | 0.95 | 21278155; 21253384; 21091502; 21051357; 21037421; 21081960; 21081960; 20873214; 20688916; 20348441 |
| NT01CP0349 | 6 | 0.89 | 16840531; 10383760; 12791149; 1552845; 2957272; 8969512; 8733228; 7830722; 8253680; 7517004 |
| NT01CP0399 | 19 | 0.92 | 21323991; 20562225; 20512054; 20448101; 20373113; 20197075; 20034619; 19414173; 19407477; 15686522 |
| NT01CP0417 | 9 | 0.80 | 21393229; 21316961; 21301088; 21185310; 21171605; 21143326; 21092192; 21078859; 21078387; 21035728 |
| NT01CP0946 | 6 | 0.92 | 21144832; 21097613; 20156448; 18703019; 18206906; 8663104; 16677303; 11839499; 1718867; 8308039 |
| NT01CP0971 | 9 | 0.94 | 20956528; 20528952; 20455949; 20434430; 20185506; 19330542; 8863531; 18759116; 18691575; 11025668 |
| NT01CR1169 | 12 | 0.79 | 20230833; 17971396; 12437884; 16547004; 16388577; 10479292; 10677216; 9831660; 7505578 |
| NT01CS1392 | 12 | 0.78 | 20711794; 18382667; 11114201; 2025413; 14616127; 12939276; 12761214 |
| NT01CS1497 | 8 | 0.72 | 21092725; 21075259; 20840762; 20810540; 20673214; 20662781; 20652542; 20642449; 20634336; 20629583 |
| NT01CS2045 | 12 | 0.85 | 19853572; 17305560; 16573693; 15882410; 15037243; 12214059; 2408275; 8106505 |
| NT01CS2059 | 9 | 0.91 | 21397712; 21145868; 20925360; 20881054; 20690035; 20543850; 20444975; 20398751; 20363355; 20213544 |
| NT01CS2540 | 18 | 0.86 | 21388343; 21340537; 21333139; 21314147; 21297084; 21295624; 21294250; 21286757; 21269934; 21219737 |
| NT01CT0059 | 8 | 0.91 | 21253866; 21036145; 10672375; 20160912; 20022530; 19594830; 19464573; 19383527; 17532339; 9324032 |
| NT01CT0097 | 6 | 0.79 | 21336311; 20980099; 20967229; 15897176; 18662996; 15660136; 9278512; 17715138; 19205745; 17102632 |
| NT01CT0114 | 6 | 0.92 | 21178863; 21110984; 21063410; 21044965; 21044966; 20888338; 20848659; 20816984; 20725929; 20724227 |
| NT01CT0239 | 8 | 0.74 | 20850431; 20679233; 19713214; 9614136; 10545161; 19111326; 18851973; 18511453; 18156349; 17973598 |
| NT01CT0257 | 8 | 0.91 | 21253866; 21036145; 20400973; 10672375; 20106611; 20022530; 19874026; 18787477; 19739093; 19688725 |
| NT01CT0279 | 19 | 0.75 | 21097580; 20668486; 20642807; 18818215; 20127467; 20118250; 17888883; 11069242; 19093149; 19025571 |
| NT01CT0524 | 14 | 0.78 | 21385632; 21374069; 21371039; 21354216; 21336542; 21331762; 21315198; 21295139; 21282572; 21155784 |
| NT01CT0749 | 18 | 0.88 | 21397193; 21377909; 21365874; 21338570; 21337475; 21317284; 21315086; 21304597; 21292166; 21267063 |
| NT01CT0769 | 5 | 0.94 | 17891922; 12207230; 9503607; 8840505; 7947754; 8492805; 2664422 |
| NT01CT0822 | 9 | 0.93 | 21370994; 21135931; 21113689; 20943853; 20925342; 20662933; 20594840; 20562282; 20557983; 20547590 |
| NT01CV0107 | 18 | 0.81 | 21183689; 21098000; 21056089; 21048979; 20975041; 20844141; 20737591; 20708685; 20691277; 20622037 |
| NT01CV0134 | 8 | 0.80 | 20577996; 20383025; 20363943; 18997324; 18186475; 16614860; 17641651; 17290794; 16403639; 16333337 |
| NT01CV0184 | 2 | 0.84 | 20054111; 19946146; 2156133; 11891227; 11135669; 8514783 |
| NT01CV0366 | 4 | 0.77 | 21398530; 21329738; 20662368; 20350525; 20129765; 20086292; 19943887; 19902840; 19874484; 11553605 |
| NT01CV0557 | 19 | 0.88 | 20564039; 17690022; 16469515; 15604744; 15221226; 15144887; 15048569; 14662300; 11403575; 12175020 |
| NT01CV0702 | 12 | 0.71 | 21309470; 21292975; 21281954; 21255722; 21255181; 21248121; 21245143; 21212461; 21209222; 21187044 |
| NT01CV0909 | 8 | 0.73 | 20693355; 19967469; 19807880; 19725876; 19597319; 15685292; 19523599; 19464998; 16565040; 19280224 |
| NT01CV1276 | 8 | 0.88 | 20823527; 20190087; 20163155; 19923715; 19574215; 19402045; 10494852; 19110079; 18685206; 18482980 |
| NT01CV1403 | 1 | 0.73 | 21121258; 20930473; 20663040; 18492492; 11709194; 17989071; 17355287; 16902948; 16435204; 16379566 |
| NT01CV1613 | 9 | 0.87 | 20823776; 20691134; 20512102; 20594942; 20403458; 20188875; 20086206; 20042190; 20007335; 19884765 |
| NT01CV1701 | 18 | 0.96 | 21077936; 20861184; 20738254; 20553499; 20552428; 20498340; 20487690; 20487268; 20479131; 20504754 |
| NT01CV1874 | 12 | 0.89 | 20030056; 19863457; 18979362; 17389905; 15075326; 12210987; 11776310; 8624513; 8661051; 10647888 |
| NT01CV2180 | 18 | 0.82 | 20566650; 19636250; 17855354; 18032408; 16870614; 16077079; 9742136; 15066148; 12857732; 12556450 |
| NT01CV2194 | 18 | 0.72 | 21041493; 20737137; 17725565; 12563288; 10231495; 689734; 8807793; 9643536; 10231495; 2984176 |
| NT01CV2195 | 18 | 0.99 | 21041493; 20737137; 20335169; 10747959; 9473041; 18535817; 17725565; 17295050; 11967085; 12563288 |
| NT01CV2404 | 9 | 0.97 | 20042190; 20034331; 19884765; 19846138; 6099397; 18842780; 18838020; 17701238; 17393230; 17284757 |
| NT01CV2409 | 18 | 0.95 | 21145416; 21103975; 20805575; 20805402; 21082744; 20732973; 20524699; 20658906; 20419433; 19325113 |
| NT01CV2812 | 15 | 0.95 | 21378195; 19016871; 3033433; 16872401; 7783615; 11358176; 2556636; 11607193; 2160938; 9244251 |
| NT01CV3147 | 6 | 0.79 | 21278781; 18432238; 16174769; 17823664; 17201058; 17132930; 16860792; 16793366; 16482161; 10869041 |
| NT01CV3254 | 19 | 0.76 | 8309940; 17504469; 2446923; 2256682; 14606945; 11191810; 8238872; 10816581; 10564766; 10361281 |
| NT01CV3444 | 8 | 0.76 | 20157809; 19924449; 19574301; 19366710; 15572765; 17451240; 15264822; 18572191; 18508968; 18365077 |
| NT01CV3447 | 19 | 0.84 | 21307593; 21282899; 21248255; 21240541; 21167943; 21131525; 21125394; 21115741; 21060843; 21052875 |
| NT01CV3454 | 18 | 0.81 | 18767164; 14962940; 15274913; 15141299; 4290867; 1995346 |
| NT01CV3671 | 15 | 0.79 | 10708364; 8750236; 2793824; 7896073; 1365888; 333433; 8391103 |
| NT01DG1935 | 6 | 0.70 | 21224401; 20972751; 20936170; 20868484; 20860693; 17971348; 20574440; 20562439; 20546858; 20007604 |
| NT01DG2374 | 8 | 0.73 | 21324604; 19781556; 19043737; 18803552; 17897734; 17576516; 17468884; 10764795; 10691985; 17024352 |
| NT01DH0392 | 8 | 0.71 | 20405215; 19908864; 18957412; 18160405; 15581577; 10852721; 10368269; 9490068; 1529349 |
| NT01DH2514 | 18 | 0.83 | 20930480; 16307111; 18182167; 17581156; 12968029; 11167132; 11159550 |
| NT01DH2654 | 8 | 0.81 | 20653766; 19368556; 17891922; 17690995; 16762453; 17091562; 17074894; 16546999; 16268586; 15520003 |
| NT01DH3198 | 14 | 0.75 | 17145955; 17020885; 16161567; 14657601; 8973304 |
| NT01DH4186 | 5 | 0.78 | 21031595; 20429505; 20030377; 19772975; 19006326; 18616587; 18351402; 18043616; 17975078; 17959596 |
| NT01DO0051 | 8 | 0.79 | 21217638; 18948093; 16239142; 15458408; 16732897; 11470801; 11056483; 9201999; 8953379; 2521219 |
| NT01DO0653 | 5 | 0.76 | 21231969; 21212358; 21038112; 20843032; 20709836; 20429505; 20411554; 20221547; 20221528; 20221527 |
| NT01DO1350 | 18 | 0.83 | 20672819; 20602459; 19843215; 19751213; 19708718; 19609573; 19539611; 19378562; 19090784; 7912814 |
| NT01DO1522 | 8 | 0.93 | 21167813; 12605683; 9546395; 9808754; 9756865; 9579062; 8529639; 2985470; 7567953; 7746153 |
| NT01DO1720 | 8 | 0.85 | 21266480; 21103375; 20932478; 20014342; 16890522; 12702667; 9427680; 18671723; 18372636; 17309779 |
| NT01DO1767 | 8 | 0.83 | 20580433; 18028869; 17321223; 16962276; 16675853; 15036329; 12409197; 11532445; 11527963; 11405622 |
| NT01DO2057 | 18 | 0.79 | 21227921; 21102408; 20960006; 3003395; 20212038; 20130683; 20085762; 19887527; 19772918; 19711043 |
| NT01DO2156 | 8 | 0.73 | 21377422; 21376574; 21364295; 21360229; 21358763; 21333622; 21320350; 21307348; 21297352; 21294156 |
| NT01DO2517 | 18 | 0.72 | 21193550; 21131438; 21129149; 21106074; 20857203; 20851956; 20724635; 20608983; 20547810; 20505076 |
| NT01DO2565 | 15 | 0.91 | 20577782; 9838063; 3148836; 17293416; 17968423; 18974048; 16428390; 17310401; 12142437; 15458412 |
| NT01DO3046 | 8 | 0.81 | 15338111; 15316720; 9254694; 11889481; 11820782; 10548510; 9729445; 9467914; 9425311; 9022686 |
| NT01DP0107 | 12 | 0.84 | 20709901; 20023146; 8759852; 17929834; 9862121; 16561900; 7894706 |
| NT01DP0173 | 18 | 0.97 | 20937798; 20863830; 20815017; 20737137; 20704181; 20698911; 20679205; 20662775; 20628015; 20625982 |
| NT01DP0368 | 18 | 0.98 | 21400686; 21399389; 21398609; 21397717; 21395595; 21393174; 21392495; 21389634; 21389261; 21383772 |
| NT01DP0551 | 8 | 0.85 | 7854413; 19928857; 16381856; 17028282; 17995952; 17938909; 17668201; 17588123; 11751810; 16808345 |
| NT01DP0570 | 8 | 0.85 | 20598080; 20367468; 6501214; 19904424; 19878267; 10585965; 19480945; 9233812; 12710666; 18400755 |
| NT01DP0600 | 3 | 0.99 | 21255118; 21115127; 21098028; 20656905; 20616068; 20472801; 20455262; 20439474; 20348252; 20345659 |
| NT01DP0630 | 8 | 0.78 | 21382015; 21307643; 21225390; 21209665; 21095201; 21091725; 21043576; 20846340; 20822098; 20808844 |
| NT01DP0662 | 9 | 0.97 | 19550039; 17906131; 15668256; 9804793; 8075395 |
| NT01DP0717 | 19 | 0.94 | 20843801; 20718423; 20595206; 20421293; 20400566; 11752303; 20201406; 19906649; 19896471; 19857612 |
| NT01DP0724 | 18 | 0.86 | 20936704; 20450883; 15294909; 12043833; 10455198; 10192807; 1830665; 9218724; 8953116; 8748033 |
| NT01DP0903 | 18 | 0.97 | 21397717; 21393220; 21385872; 21385626; 21384128; 21378194; 21377371; 21375706; 21371898; 21366702 |
| NT01DP0913 | 18 | 0.92 | 21396420; 21373771; 21372499; 21358696; 21347255; 21341987; 21335086; 21292027; 21170889; 21170872 |
| NT01DP0971 | 6 | 0.74 | 11278070; 15563835; 3555843; 12872134; 10860755; 11866515; 11183780; 10930420; 10465790; 10570977 |
| NT01DP1143 | 4 | 0.78 | 21292743; 21242066; 21162553; 21131490; 21108067; 20923659; 20863296; 20959858; 20738376; 20693676 |
| NT01DP1198 | 8 | 0.73 | 21298264; 21266406; 21120027; 21033739; 20880712; 20819069; 20819062; 20738256; 20728216; 20691944 |
| NT01DP1225 | 8 | 0.78 | 21228234; 21106768; 20809899; 20620150; 5420057; 20516620; 20479254; 20435888; 20171064; 20096472 |
| NT01DP1266 | 12 | 0.90 | 21357508; 21279407; 20977881; 20890740; 20540562; 20303406; 19088197; 20159564; 20132728; 20091748 |
| NT01DP1369 | 8 | 0.73 | 21178285; 20811135; 19966484; 19546514; 19424620; 19200041; 17291445; 16402358; 16221991; 15996793 |
| NT01DP1508 | 18 | 0.95 | 21103969; 11048718; 20188576; 20103563; 20003133; 17493798; 19373193; 18208836; 19252335; 19816142 |
| NT01DP1558 | 9 | 0.88 | 10482673; 12454267; 10828604; 9892232; 9804328; 9748261; 9165098; 7668351; 8441459 |
| NT01DP1806 | 18 | 0.84 | 21400700; 21398536; 21396409; 21389053; 21385840; 21382104; 21380641; 21378973; 21378164; 21377446 |
| NT01DP1811 | 8 | 0.91 | 21387012; 21377190; 21358763; 21178975; 21119085; 21082227; 21056772; 20966878; 20957679; 20957220 |
| NT01DP1813 | 12 | 0.80 | 20032457; 11751634; 17504491; 17855452; 10569626; 16127432; 16109378; 15249048; 15195946; 15157086 |
| NT01DP2029 | 15 | 0.78 | 21388403; 21372131; 21371473; 21365689; 21354350; 21352825; 21344481; 21342606; 21333795; 21331030 |
| NT01DP2068 | 8 | 0.97 | 20688826; 20498375; 17975082; 16529396; 16274230; 12486057; 14576151; 12714601; 12605683; 12409197 |
| NT01DP2070 | 8 | 0.93 | 21167813; 12605683; 9546395; 9808754; 9756865; 9579062; 8529639; 2985470; 7567953; 7746153 |
| NT01DP2207 | 15 | 0.94 | 21057008; 20834167; 20833804; 20954302; 20363229; 18052041; 12368235; 19220749; 18818215; 18789936 |
| NT01DP2265 | 15 | 0.89 | 21303547; 21288895; 20576608; 20400540; 16211538; 19996382; 18052041; 11252893; 18846291; 17908933 |
| NT01DP2319 | 18 | 0.95 | 21383019; 21368759; 21343303; 21297161; 21286403; 21254069; 21182082; 21106767; 21086519; 21078855 |
| NT01DP2465 | 8 | 0.80 | 21253866; 21036145; 20798971; 10672375; 20160912; 20022530; 9324032; 17667915; 17635929; 8798399 |
| NT01DP2486 | 5 | 0.97 | 21392199; 21152399; 21112120; 20946949; 20938698; 20935495; 20890097; 20851900; 20839006; 20586061 |
| NT01DP2600 | 8 | 0.89 | 21334908; 21273120; 21236692; 21209090; 21178163; 21152271; 21114358; 21105905; 21098488; 21095085 |
| NT01DP2629 | 2 | 0.89 | 21364293; 21236255; 21099135; 20873749; 20822158; 20815377; 20734996; 20709756; 20681987; 20601509 |
| NT01DP2734 | 1 | 0.90 | 18266921; 11852094; 3534538; 9353942; 7502581 |
| NT01DP2814 | 9 | 0.98 | 21358050; 21357626; 21235239; 20797606; 20696925; 20563648; 20406823; 11251294; 3537305; 20013982 |
| NT01DP2910 | 15 | 0.72 | 21390248; 21352825; 21338338; 21335064; 21312339; 21311889; 21248136; 21211289; 21189160; 21187486 |
| NT01DP3241 | 3 | 0.87 | 20455262; 17468768; 16390451; 15170403; 9157238; 12040098; 11886754; 10026202; 11401692; 10972813 |
| NT01DP3355 | 19 | 0.95 | 21354427; 21311881; 21304822; 21290547; 21221939; 21193820; 21184415; 21131522; 21106368; 21094645 |
| NT01DP3418 | 19 | 0.93 | 17517613; 16049679; 10552901; 9620799; 9644208; 4927838; 1712067; 2983993 |
| NT01DP3455 | 18 | 0.99 | 21179522; 20805337; 20227482; 18694716; 19894125; 11029592; 18973471; 18535817; 18462159; 16139844 |
| NT01DP3471 | 8 | 0.89 | 21357619; 21167813; 20688826; 20498375; 20447995; 19959573; 860983; 18716757; 18086437; 17975082 |
| NT01DP3484 | 9 | 0.89 | 21387258; 21386960; 21385844; 21369973; 21366729; 21347706; 21343303; 21342076; 21293475; 21268534 |
| NT01DR0774 | 19 | 0.94 | 21299880; 21215963; 21097495; 21062783; 20868765; 20804758; 20685646; 20656867; 20632938; 20503105 |
| NT01DS0233 | 15 | 0.91 | 20577782; 9838063; 3148836; 17293416; 17968423; 18974048; 16428390; 17310401; 12142437; 15458412 |
| NT01DS0610 | 8 | 0.87 | 21387012; 21339825; 21220430; 21190551; 21120593; 21035093; 20868692; 20846527; 20802042; 20649538 |
| NT01DS0809 | 6 | 0.80 | 21288492; 21274582; 21123178; 21111748; 20884785; 20844047; 20719949; 20711169; 20705653; 20675469 |
| NT01DS1119 | 8 | 0.93 | 20400563; 18485344; 16546999; 11313346; 11115640; 11054105; 10985736; 9683494; 8471182 |
| NT01DS1391 | 4 | 0.83 | 21403855; 21398556; 21398543; 21394468; 21393140; 21389148; 21389112; 21387406; 21383169; 21380604 |
| NT01DS1523 | 14 | 0.97 | 11157898; 1644750; 18359660; 17919281; 1729199; 19210675; 19093079; 18757797; 7463489; 8107083 |
| NT01DS1598 | 9 | 0.96 | 20725044; 20616867; 19423627; 1355089; 15514159; 18557770; 17266990; 10716717; 16707089; 16276872 |
| NT01DS1600 | 9 | 0.99 | 21389045; 21376757; 21342605; 21327327; 21284260; 21216135; 21209665; 21204617; 21111594; 21076105 |
| NT01EC0026 | 13 | 0.72 | 16218899; 19841848; 3132458; 17501926; 9554847; 16697013; 15388946; 14672940; 14596614; 12783542 |
| NT01EC0049 | 12 | 0.95 | 20870944; 20647553; 20628058; 20529839; 20492473; 20456758; 20451919; 18813375; 20121198; 20121197 |
| NT01EC0050 | 8 | 0.79 | 15386115; 9804883; 14702404; 6751257; 10026281; 271968; 13895406; 8599534; 8525056; 7473063 |
| NT01EC0051 | 8 | 0.85 | 20814302; 12521268; 15386115; 9804883; 11297752; 8599534; 1850088; 2503674; 772161; 881736 |
| NT01EC0140 | 8 | 0.81 | 21195720; 20807773; 20607720; 20354189; 19781588; 19520720; 18162174; 17973442; 17920150; 15317876 |
| NT01EC0142 | 5 | 0.95 | 20124698; 20095968; 16515461; 16176926; 15150268; 9862121; 15096507; 12600205; 12463749; 12403622 |
| NT01EC0147 | 8 | 0.71 | 21400553; 21398595; 21398434; 21397806; 21397649; 21397531; 21396919; 21395333; 21392498; 21389623 |
| NT01EC0178 | 18 | 0.98 | 19708689; 17956229; 19252278; 387415; 17927700; 17925389; 16928679; 10850805; 10348879; 16430693 |
| NT01EC0179 | 18 | 0.98 | 16928679; 10850805; 2259338; 16336924; 16158230; 16142938; 2066336; 15215626; 12213932; 10746769 |
| NT01EC0485 | 18 | 0.94 | 21177413; 20566641; 20179178; 20164366; 20032990; 19725576; 18039771; 9043118; 16453883; 18055536 |
| NT01EC0572 | 6 | 0.77 | 21144832; 21097613; 18703019; 8663104; 16677303; 11839499; 1718867; 8308039; 1667219; 3456159 |
| NT01EC0620 | 15 | 0.84 | 21193605; 21187094; 20699632; 10470851; 20337533; 20219606; 20178842; 20167774; 20093293; 19931317 |
| NT01EC0725 | 18 | 0.89 | 20335169; 17447029; 12747420; 9643536; 10348879; 7885234; 1479347; 1838574; 1787794 |
| NT01EC0768 | 9 | 0.88 | 20853818; 20826347; 20593270; 20491126; 20175558; 20173094; 19769329; 11844786; 18563089; 19280380 |
| NT01EC0860 | 15 | 0.89 | 20932844; 9466251; 20498088; 20231408; 20196071; 20146748; 19850005; 1630316; 19400808; 19101563 |
| NT01EC0862 | 18 | 0.95 | 11129050; 1741458; 12459492; 11984815; 1587485; 10551842; 8302219; 8626559; 7888828; 2286635 |
| NT01EC0888 | 8 | 0.87 | 21272569; 21268708; 21173220; 21083040; 21082267; 21081313; 20980436; 20951673; 20920510; 20842377 |
| NT01EC0915 | 18 | 0.99 | 15388932; 8422996; 10403413; 9654141; 8033999; 3943911; 7018904 |
| NT01EC0938 | 18 | 0.99 | 21315686; 21122131; 20855510; 20704181; 20662775; 12867445; 20132520; 20070462; 19484827; 19389779 |
| NT01EC0975 | 18 | 0.94 | 20870774; 20467255; 9787636; 19095018; 17551219; 17159201; 17064369; 12563288; 16549425; 16524964 |
| NT01EC1015 | 2 | 0.96 | 21206014; 21075928; 19818021; 15680760; 18959753; 17686778; 17509837; 7047497; 17374639; 15306815 |
| NT01EC1063 | 8 | 0.73 | 21377371; 21322491; 21298162; 21239558; 21216904; 21147635; 21087381; 21063632; 21059948; 21053013 |
| NT01EC1073 | 18 | 0.92 | 21397717; 21375706; 21369825; 21366702; 21315686; 21311724; 21310787; 21300772; 21282456; 21217007 |
| NT01EC1081 | 18 | 0.83 | 19784448; 16040611; 15470119; 12438693; 12393891; 8386371; 2470676; 2989179; 8969509; 1310666 |
| NT01EC1199 | 19 | 0.99 | 21307367; 21261077; 21199899; 21190553; 21183069; 21183069; 21174953; 21167155; 21149736; 21149550 |
| NT01EC1217 | 12 | 0.88 | 21148698; 20812717; 20688826; 20615351; 20064164; 19754117; 19049517; 19013157; 17040909; 2227449 |
| NT01EC1225 | 15 | 0.72 | 20718459; 18724706; 17709748; 18313075; 17224607; 9493270; 15728912; 10331874; 15588829; 15447145 |
| NT01EC1262 | 8 | 0.80 | 19184529; 2293096; 10663127; 16339959; 13719300; 12127981; 12081029; 2271542; 10684634; 10518796 |
| NT01EC1294 | 8 | 0.84 | 20888212; 20688172; 20675294; 20518346; 20353187; 19943898; 19899738; 19751796; 19651103; 18388293 |
| NT01EC1300 | 8 | 0.84 | 21395221; 21385868; 21323311; 21299470; 21275844; 21235502; 21222452; 21210868; 21194355; 21190518 |
| NT01EC1492 | 12 | 0.71 | 21401077; 21397717; 21357544; 21338417; 21319304; 21304995; 21245269; 21221131; 21139434; 21125667 |
| NT01EC1546 | 9 | 0.97 | 17899070; 17891922; 16388583; 15708363; 15668256; 12770824; 9388293; 9438344; 8341260; 1547954 |
| NT01EC1585 | 8 | 0.73 | 18983993; 18705875; 12538805; 11741210; 9682270; 9615466; 9600061; 5432063; 9182554; 9022693 |
| NT01EC1598 | 18 | 0.95 | 20942908; 20601430; 20592244; 20543096; 20511219; 20305274; 15770687; 20004175; 19803484; 19762344 |
| NT01EC1625 | 15 | 0.74 | 4927203; 10651904; 8982091; 8093697; 7642533 |
| NT01EC1637 | 11 | 0.86 | 21244532; 21087627; 21062824; 19919677; 19809245; 19718510; 19268511; 19268511; 17021227; 19085081 |
| NT01EC1802 | 18 | 0.92 | 18851927; 12563288; 8752321; 15175298; 10767421; 8574415; 7711833; 1519451; 6757669 |
| NT01EC1809 | 18 | 0.92 | 21369940; 21368142; 21262798; 21216025; 21203343; 21153767; 21151096; 21148207; 21130072; 21124885 |
| NT01EC1868 | 18 | 0.95 | 10952578; 19931508; 10336456; 19380587; 10953001; 17702946; 11162101; 17764658; 10230402; 17380425 |
| NT01EC1949 | 15 | 0.90 | 21124821; 17660406; 14523117; 12398213; 10433720; 1608976; 9802029; 8596440; 7932717; 7584034 |
| NT01EC1950 | 6 | 0.82 | 20464017; 19462083; 17376083; 17376066; 17376071; 17317110; 16950091; 16921356; 16814717; 16238625 |
| NT01EC2004 | 6 | 0.84 | 21388532; 21365542; 21350762; 21343909; 21336027; 21325134; 21321231; 21091440; 21086493; 21078962 |
| NT01EC2089 | 8 | 0.73 | 20935102; 20599671; 20511298; 19486643; 18346472; 17543971; 17459874; 16467323; 16807925; 16289704 |
| NT01EC2159 | 8 | 0.79 | 16212227; 15997113; 11535415; 10449319; 10666297; 1898369; 10564799; 10549852; 10548452; 10228449 |
| NT01EC2410 | 18 | 0.95 | 10952578; 19931508; 10336456; 19380587; 10953001; 17702946; 11162101; 17764658; 10230402; 17380425 |
| NT01EC2496 | 19 | 0.93 | 21279381; 21174346; 20920201; 20672277; 20636376; 3611062; 20383007; 11752303; 20199575; 20124190 |
| NT01EC2532 | 8 | 0.74 | 21388699; 21377527; 21376957; 21277857; 21253895; 21184218; 21166979; 21152904; 21117456; 21071844 |
| NT01EC2537 | 8 | 0.71 | 21294156; 21080034; 21042030; 20406700; 20363943; 20148428; 20138225; 20026236; 19968635; 19160513 |
| NT01EC2571 | 15 | 0.87 | 15699211; 10708580; 9685006; 9636707; 1729202 |
| NT01EC3076 | 8 | 0.83 | 20372835; 18793820; 1137083; 16880594; 15128296; 10873522; 9716718; 9346300; 7922040; 9183016 |
| NT01EC3109 | 15 | 0.75 | 20971899; 2179676; 19889087; 12011022; 18682280; 18619465; 7590326; 17827157; 17536848; 9554854 |
| NT01EC3268 | 8 | 0.90 | 19784641; 18760354; 15211509; 18483816; 17309679; 16166538; 15122035; 16930323; 16791965; 16134116 |
| NT01EC3418 | 19 | 0.70 | 21400715; 21400698; 21400681; 21400661; 21400660; 21400651; 21400585; 21400575; 21400481; 21400228 |
| NT01EC3427 | 18 | 0.97 | 21398536; 21394084; 21393832; 21393450; 21393247; 21393174; 21392495; 21392072; 21391724; 21390324 |
| NT01EC3496 | 6 | 0.93 | 21306445; 21303535; 21205011; 21187148; 21170359; 21087930; 20600282; 20465561; 20457749; 20304994 |
| NT01EC3509 | 9 | 0.96 | 21368116; 21294903; 21209024; 21060891; 21030508; 21084710; 20957029; 20923423; 20725044; 20690600 |
| NT01EC3610 | 18 | 0.85 | 21385626; 21384128; 21375716; 21373893; 21335525; 21295699; 21294757; 21290218; 21285405; 21170872 |
| NT01EC3626 | 18 | 0.88 | 21204810; 21163614; 21049012; 21040760; 20880839; 20875871; 20802201; 20689940; 20678983; 20649599 |
| NT01EC3634 | 8 | 0.84 | 20924198; 18467858; 18188555; 18060402; 17587673; 10867230; 12149117; 8617755; 11934292; 11931561 |
| NT01EC3716 | 9 | 0.93 | 20622065; 11591162; 11514237; 8993540; 11060310; 10841782; 9448727; 9346310; 8344281; 7968274 |
| NT01EC4025 | 18 | 0.71 | 21264209; 21122159; 20696859; 20647000; 20525687; 20466765; 20453832; 12177321; 20345654; 20192961 |
| NT01EC4174 | 18 | 0.74 | 15917616; 10940570; 9461423; 7922040; 9274047; 7765086; 8254318 |
| NT01EC4415 | 19 | 0.88 | 8862584; 12535074; 11069912; 10398745; 8606157; 7854127; 7934840; 3155716; 4590475; 2168379 |
| NT01EC4419 | 19 | 0.77 | 18822381; 18984595; 10829079; 19201821; 16871971; 16773832; 8370744; 12427940; 9675370; 11521078 |
| NT01EC4513 | 8 | 0.75 | 21362632; 21294640; 21254817; 21238579; 21187465; 21176082; 21099366; 20980392; 20837989; 20823560 |
| NT01EC4556 | 18 | 0.93 | 21197505; 21196294; 21140912; 21044958; 21037554; 21036245; 20949136; 20926757; 20876384; 20851114 |
| NT01EC4705 | 18 | 0.91 | 20881245; 8202535; 18798051; 18343875; 17079719; 11045621; 15927004; 15589830; 10871608; 12576593 |
| NT01EC4716 | 15 | 0.97 | 10510236; 9393850; 9179845; 2040302; 1658569; 1556120; 1094240; 3012272; 3038334; 3316209 |
| NT01EC4887 | 15 | 0.81 | 19709076; 2450098; 19230635; 15757897; 10948194; 9634699; 9511734; 9268370; 9043061; 7939631 |
| NT01EC5028 | 15 | 0.97 | 21402151; 21401077; 21398634; 21396120; 21393212; 21386983; 21385724; 21383239; 21381030; 21379585 |
| NT01EC5164 | 4 | 0.80 | 21336656; 20935126; 20022964; 15489417; 10483722; 10339816; 17623030; 17416361; 16803593; 15882409 |
| NT01EC5189 | 18 | 0.98 | 21034864; 20961850; 20921224; 20602459; 20512981; 20497190; 20423050; 20414585; 19917612; 19902387 |
| NT01EC5352 | 15 | 0.76 | 20952573; 18793199; 18375801; 11121424; 16207917; 10485902; 12177351; 2540407; 8913298; 7934818 |
| NT01EL0495 | 8 | 0.94 | 21397031; 21371425; 21369577; 21349860; 21338723; 21305026; 21303655; 21209920; 21197843; 21185811 |
| NT01EL0738 | 6 | 0.82 | 20875449; 20192920; 18754681; 17586464; 15509558; 15230094; 9757107; 12473678; 11972788; 11886555 |
| NT01EL1848 | 18 | 0.82 | 21382378; 21357485; 21353727; 21346155; 21331260; 21329510; 21318561; 21262962; 21245269; 21238925 |
| NT01EL2029 | 4 | 0.86 | 20138126; 19826764; 19719512; 16861227; 15758237; 11683355; 9735283; 8709848 |
| NT01EL2309 | 18 | 0.76 | 21389348; 21368759; 21359673; 21358755; 21358125; 21357745; 21354408; 21352491; 21351879; 21350489 |
| NT01EL2697 | 8 | 0.75 | 21362632; 21294640; 21254817; 21238579; 21187465; 21176082; 21099366; 20980392; 20837989; 20823560 |
| NT01EL2975 | 1 | 0.76 | 12966138; 14964871; 7711027; 8012909; 8002714; 8098035; 1906928; 2153658; 2981222; 2834822 |
| NT01EL2983 | 8 | 0.93 | 21393237; 21387012; 21339825; 21334908; 21310150; 21290824; 21268347; 21254627; 21253496; 21226204 |
| NT01ER0115 | 2 | 0.74 | 19916033; 18997428; 18047786; 15379557; 15215589; 12639570; 12595740; 12029484 |
| NT01ER0371 | 13 | 0.97 | 18926798; 17980389; 2011739; 16962594; 15994936; 14970222; 11983710 |
| NT01ER0979 | 6 | 0.72 | 21211912; 20678074; 8807206; 7494037; 17476576; 16207839; 11354623; 15068247; 14621319; 12140246 |
| NT01FA0139 | 19 | 0.94 | 21367611; 21366294; 21355999; 21341216; 21287229; 21258004; 21253720; 21223393; 21193007; 21188612 |
| NT01FA0889 | 9 | 1.00 | 8406042; 637839; 13595904; 17401135; 14580958; 10380806; 11163788; 1551888; 1562581; 16665177 |
| NT01FA1065 | 8 | 0.72 | 20449117; 20015072; 19894084; 10700270; 19397993; 19027887; 18755150; 18248595; 17906130; 16978355 |
| NT01FA1571 | 9 | 0.79 | 20925342; 20562282; 20557983; 20304657; 20018879; 19874137; 19427006; 16436705; 18824113; 18574592 |
| NT01FA2725 | 8 | 0.83 | 21051545; 20652619; 20347849; 17280684; 20025616; 19876765; 19811920; 2294092; 1555572; 18355283 |
| NT01FA3328 | 8 | 0.80 | 21397737; 21041678; 21030590; 20866105; 20849886; 20809650; 20727152; 20089849; 20629071; 20486243 |
| NT01FA3636 | 8 | 0.92 | 21392498; 21389623; 21388699; 21382707; 21382335; 21381206; 21380809; 21380594; 21376665; 21376562 |
| NT01FA3646 | 1 | 0.70 | 19427006; 19271978; 19101548; 15829503; 17922758; 11208803; 17906131; 17524985; 17185548; 10849007 |
| NT01FA3899 | 19 | 0.72 | 21167155; 20965199; 20594157; 20462494; 20453092; 20400545; 20223804; 19923747; 19246764; 17488738 |
| NT01FA4306 | 8 | 0.74 | 21073414; 20889709; 20650894; 20574584; 20193780; 20168325; 19624733; 18348; 12913124; 18096502 |
| NT01FA4563 | 8 | 0.82 | 21333648; 21255444; 21217202; 21207565; 21199794; 21193412; 21177970; 21122813; 21119630; 21109751 |
| NT01FA4692 | 4 | 0.74 | 19350532; 9157240; 18339368; 11677609; 11463916; 4822122; 11287152; 14763981; 14617184; 11737645 |
| NT01FA5557 | 8 | 0.78 | 21038477; 20534465; 20075070; 11081790; 18434308; 970945; 1925561; 16218963; 16088215; 16048999 |
| NT01FA5983 | 19 | 0.96 | 21378160; 21365786; 21339299; 21333726; 21332624; 21331585; 21313853; 21310393; 21307368; 21306246 |
| NT01FA6585 | 4 | 0.82 | 20974846; 20728562; 20582873; 20164561; 20088490; 20076970; 20022137; 19969519; 19815002; 19635794 |
| NT01FA6732 | 12 | 0.87 | 20487289; 20059688; 19339102; 18995833; 18394994; 2137128; 16147871; 16076845; 14970903; 14673507 |
| NT01FJ0179 | 12 | 0.74 | 21262840; 21241743; 20823542; 10871752; 10913103; 19530167; 16987329; 18421280; 11030354; 18187213 |
| NT01FJ0822 | 19 | 0.92 | 21380775; 21369910; 21367878; 21367655; 21345211; 21342516; 21339299; 21299880; 21269444; 21255377 |
| NT01FJ0824 | 19 | 0.84 | 21146160; 20681989; 20662765; 20622018; 20540109; 19840850; 19640850; 19234795; 19154353; 19111640 |
| NT01FJ0826 | 19 | 0.94 | 20622122; 20570296; 20349118; 20169343; 20153658; 19706595; 19683032; 19605459; 12045105; 19504047; 20811723; 20686915; 20681989; 20622122; 20532756; 20427274; 20227362; 20075633; 20044921; 10952578 |
| NT01FJ0850 | 6 | 0.94 | 21139085; 21067515; 20622854; 20403335; 15814643; 20208542; 19010785; 20057358; 9182762; 19047520 |
| NT01FJ1662 | 19 | 0.87 | 21273341; 21249355; 21174947; 21152915; 21097495; 21036948; 20932833; 20872041; 20812985; 18280679 |
| NT01FJ1667 | 19 | 0.92 | 21307593; 21174947; 21056976; 21036948; 20852022; 20826810; 20739278; 20709893; 20686915; 20682344 |
| NT01FJ2389 | 18 | 0.83 | 21397012; 21396938; 21395536; 21393220; 21391095; 21389137; 21388960; 21388812; 21381693; 21381059 |
| NT01FJ3339 | 19 | 0.74 | 21181156; 20919961; 20681989; 20637827; 20477891; 20438826; 20336338; 20228117; 20208168; 20087845 |
| NT01FJ4235 | 19 | 0.96 | 17139091; 16330537; 15659099; 8444792; 68686 |
| NT01FJ4519 | 12 | 0.93 | 20802071; 20800580; 20723343; 20718289; 19819358; 19782652; 19631209; 19545519; 19416551; 11136446 |
| NT01FJ4703 | 18 | 0.96 | 20038594; 19425588; 19361527; 1715858; 19166984; 12107133; 18179581; 17420217; 10858345; 12878498 |
| NT01FJ5291 | 19 | 0.89 | 20713411; 20673219; 20570296; 20547381; 20426480; 20385553; 20169343; 20153658; 20036247; 19883701 |
| NT01FM0218 | 12 | 0.80 | 20032457; 11751634; 17504491; 17855452; 10569626; 16127432; 16109378; 15249048; 15195946; 15157086 |
| NT01FM1165 | 8 | 0.74 | 21334308; 20884367; 20803144; 20631991; 20471587; 20028436; 19897917; 19834705; 1905518; 19060392 |
| NT01FM1440 | 4 | 0.73 | 21397846; 21391297; 21390252; 21390146; 21390130; 21390126; 21389676; 21389279; 21387154; 21383206 |
| NT01FN0297 | 18 | 0.74 | 21315686; 21214909; 21209283; 21190940; 21177413; 21144872; 21059948; 21059651; 20976528; 20861263 |
| NT01FN0307 | 8 | 0.82 | 21293192; 21216999; 21208358; 21149440; 20952574; 20850485; 20809899; 20675490; 20653766; 20632378 |
| NT01FN0322 | 18 | 0.75 | 20675482; 20421419; 15736970; 16091943; 15723527; 11470438; 10700278; 9655823; 9383187; 270744 |
| NT01FN0342 | 11 | 0.96 | 8556862; 10476039; 9648746; 2170815; 3894006 |
| NT01FN0406 | 18 | 0.79 | 20515934; 19796645; 17041044; 17302825; 12860693; 15151995; 14726537; 9006035; 9006035; 11163480 |
| NT01FN0796 | 18 | 0.74 | 20031922; 17256083; 16632906; 7190579; 16044265; 12761179; 12706816; 11271498; 10366717; 10216867 |
| NT01FN1081 | 2 | 0.78 | 21371789; 21059411; 21038477; 20843822; 20534592; 20222145; 20158163; 20106978; 19917609; 19791805 |
| NT01FN1669 | 12 | 0.89 | 8143162; 16700049; 11524383; 15870898; 11292347; 11021970 |
| NT01FS0339 | 12 | 0.79 | 21246635; 21221970; 21109561; 20946650; 20886060; 20870766; 20807319; 20718868; 20698901; 20547176 |
| NT01FS0459 | 4 | 0.85 | 21122120; 20223806; 15933011; 10517576; 16954040; 16232707; 12833261; 10464216; 10464216; 10913872 |
| NT01FS0778 | 8 | 0.81 | 21268708; 21262951; 21059645; 20885442; 20707865; 20705058; 19768395; 11717466; 16665774; 10896219 |
| NT01FS0843 | 12 | 0.86 | 20927194; 20522495; 20149826; 20146860; 19376213; 18256178; 18819916; 1409590; 11310352; 18227262 |
| NT01FS0915 | 6 | 0.84 | 19425495; 18838147; 17069852; 16649659; 15554191; 12669426; 10511538; 12500545; 10686096; 9608939 |
| NT01FS1618 | 19 | 0.80 | 21397493; 21342541; 20876582; 20831592; 20628005; 20624909; 20439478; 20236121; 20048165 |
| NT01FS1791 | 12 | 0.84 | 21037302; 20826214; 20616713; 19139197; 18444997; 20007980; 18794289; 19914916; 19777228; 19776420 |
| NT01FS3203 | 6 | 0.73 | 21360524; 21306759; 21278145; 21254357; 21227392; 21189161; 21187396; 21151910; 21125561; 21120523 |
| NT01FS3498 | 18 | 0.91 | 21194120; 20936291; 20858756; 20831043; 20827334; 20703447; 21226590; 20576528; 20540435; 20529095 |
| NT01FS4876 | 8 | 0.71 | 21385856; 21378481; 21364283; 21354057; 21352713; 21352711; 21344764; 21306609; 21288332; 21258047 |
| NT01GK0229 | 8 | 0.77 | 15780999; 11989713; 8836445; 8605224; 9035688; 8308022; 8308020; 8515235; 8398079; 14179673 |
| NT01GK0265 | 9 | 0.71 | 20023301; 8624514; 19179086; 18808455; 18088304; 17932115; 16824521; 16762032; 16009708; 15277243 |
| NT01GK0310 | 15 | 0.96 | 19202108; 11114919; 12686116; 11158353; 1318499; 9889976; 9389448; 9168623; 7582014; 4896022 |
| NT01GK0391 | 4 | 0.73 | 20639340; 19926649; 19903372; 2077343; 18931428; 17967850; 17850346; 10419969; 15336432; 15225313 |
| NT01GK0451 | 2 | 0.83 | 11796116; 11132637; 8674784; 8706691; 8537361 |
| NT01GK0524 | 19 | 0.97 | 20824214; 20707404; 19825675; 18597864; 18508770; 18043952; 17955483; 17934909; 15608179; 16158237 |
| NT01GK0543 | 8 | 0.79 | 20920450; 20890095; 20879962; 20865387; 20834161; 20805024; 20739172; 20702080; 20676631; 20673625 |
| NT01GK0818 | 8 | 0.73 | 15518552; 12651118; 12095640; 10096082; 8412675; 8969175; 1319216; 1908789; 2253629 |
| NT01GK0837 | 8 | 0.91 | 21364306; 21359858; 21339386; 21336645; 21332529; 21329681; 21328323; 21297347; 21295965; 21291872 |
| NT01GK0949 | 4 | 0.83 | 21398543; 21375591; 21344251; 21092197; 20863881; 20621133; 20601499; 20551082; 20545323; 20451384 |
| NT01GK0984 | 19 | 0.91 | 18210176; 15820665; 15552059; 12686644; 10521532 |
| NT01GK1100 | 8 | 0.76 | 21223593; 21166389; 21141474; 20962905; 20931881; 20802042; 20737074; 20692227; 20673625; 20669254 |
| NT01GK1114 | 19 | 0.74 | 21400688; 21400235; 21398593; 21393228; 21392988; 21390508; 21390257; 21388958; 21388880; 21388433 |
| NT01GK1146 | 9 | 0.83 | 20590527; 20370610; 20178986; 8406042; 20099411; 20080211; 19686777; 19571038; 19525201; 15461798 |
| NT01GK1183 | 8 | 0.92 | 21036145; 20675489; 10672375; 20022530; 19874026; 18787477; 19594830; 19464573; 19264849; 17532339 |
| NT01GK1209 | 8 | 0.95 | 21042417; 20808761; 20807714; 20650322; 20586425; 17525339; 20532892; 20450929; 20429690; 20335176 |
| NT01GK1400 | 13 | 0.96 | 4868216; 16825789; 15692745; 15185964; 12628255; 11824762; 10360176 |
| NT01GK1690 | 4 | 0.83 | 21398543; 21375591; 21344251; 21092197; 20863881; 20621133; 20601499; 20551082; 20545323; 20451384 |
| NT01GK1712 | 4 | 0.83 | 21398543; 21375591; 21344251; 21092197; 20863881; 20621133; 20601499; 20551082; 20545323; 20451384 |
| NT01GK1847 | 4 | 0.79 | 20795493; 12692562; 17486317; 16545948; 15712669; 14507368; 10217485; 9799102 |
| NT01GK1880 | 9 | 0.92 | 21398488; 21384891; 21382465; 21354350; 21296885; 21263204; 21187145; 21155016; 21110176; 21106133 |
| NT01GK2083 | 9 | 0.95 | 21397998; 21185310; 20864541; 20800575; 20698542; 20533312; 20410062; 20335945; 20303926; 8406042 |
| NT01GK2206 | 9 | 0.94 | 21389045; 21349883; 21317262; 21306440; 21296915; 21284260; 21276087; 21183032; 21125681; 21122111 |
| NT01GK2221 | 8 | 0.74 | 20026072; 16499623; 16023116; 12186751; 11004571; 9443811; 9417993; 8786138; 7584606; 1449496 |
| NT01GK2384 | 12 | 0.82 | 21398432; 21357544; 21266308; 21255413; 21158868; 21148414; 21148206; 21125667; 21118527; 21104177 |
| NT01GK2467 | 8 | 0.79 | 21400553; 21398445; 21397652; 21397649; 21381313; 21378135; 21376826; 21371425; 21360752; 21353684 |
| NT01GK2496 | 2 | 0.82 | 3032913; 4874308; 14898026; 9446573; 7929373; 8425548 |
| NT01GK2516 | 15 | 0.89 | 21091510; 18662309; 15158709; 15504593; 10972836; 8999810; 9000055; 10438769; 10972837; 10972836 |
| NT01GK2730 | 18 | 0.78 | 21400508; 21400108; 21398567; 21397510; 21397191; 21396766; 21396747; 21395869; 21395220; 21395069 |
| NT01GK2765 | 4 | 0.79 | 20382772; 357; 17376078; 17420574; 17376078; 16959571; 15882622; 15752199; 11886548; 9287012 |
| NT01GK2826 | 6 | 0.94 | 19740104; 7528109; 17851501; 9413993; 8055176; 16378748; 15363844; 9419227; 9499421; 11927535 |
| NT01GK3029 | 8 | 0.73 | 21178285; 20811135; 19966484; 19546514; 19424620; 19200041; 17291445; 16402358; 16221991; 15996793 |
| NT01GK3043 | 18 | 0.86 | 21375706; 21369825; 21366702; 21315686; 21311724; 21310787; 21300772; 21282456; 21217007; 21204689 |
| NT01GK3216 | 8 | 0.89 | 15389597; 19115036; 17294170; 16027951; 14705036; 7826011; 14638414; 12855725; 11566129; 2971647 |
| NT01GK3242 | 18 | 0.71 | 21143936; 21130726; 21078125; 20952578; 21209049; 20937701; 20881245; 20873853; 20817733; 20729555 |
| NT01GK3396 | 4 | 0.73 | 21397846; 21391297; 21390252; 21390146; 21390130; 21390126; 21389676; 21389279; 21387154; 21383206 |
| NT01GK3480 | 19 | 0.92 | 20829291; 20538266; 16980456; 17524545; 13971270; 16859842; 16044265; 15990253; 12700273; 10930752 |
| NT01GK3546 | 8 | 0.84 | 21397011; 21374662; 21338421; 21334408; 21297247; 21253866; 21238944; 21209092; 21252179; 21136949 |
| NT01GK3561 | 15 | 0.99 | 21375718; 17630784; 19118356; 18375800; 10094626; 16519689; 3275606; 8455551; 10092812; 11750821 |
| NT01GK3679 | 8 | 0.95 | 20610779; 18651753; 15126303; 12914915; 10549856; 9952163 |
| NT01GK3765 | 8 | 0.95 | 21353684; 21351093; 21343247; 21336929; 21303655; 21296058; 21272640; 21272045; 21244052; 21238579 |
| NT01GK3841 | 4 | 0.77 | 21204254; 19779460; 12586834; 17425940; 12813085; 4185146; 11889108; 2105300; 11606741; 7768847 |
| NT01GM0477 | 19 | 0.82 | 19836342; 12220406; 16540473; 15788406; 10480912; 11545592; 1312094; 8810902; 7918629; 8200457 |
| NT01GM1167 | 8 | 0.75 | 20562308; 20467754; 3006051; 18302536; 18283514; 17233767; 16531087; 15975858; 12634328; 12586382 |
| NT01GM1177 | 19 | 0.94 | 21111546; 21040514; 21040511; 20851903; 20594961; 20497501; 20345654; 18818215; 20199110; 20173067 |
| NT01GM1211 | 12 | 0.97 | 20944232; 20837014; 18715016; 16381862; 11114514 |
| NT01GM1595 | 8 | 0.75 | 18056997; 18956890; 17612488; 17256840; 9933463; 15100020; 14672930; 12809486; 11960460; 10727220 |
| NT01GM2087 | 19 | 0.95 | 20957510; 19332829; 16002992; 12055294; 9748211; 9792849; 9570402; 1834675 |
| NT01GM2621 | 18 | 0.96 | 21357299; 21221942; 20981744; 20979388; 20883496; 20865003; 20696879; 20583998; 20488669; 20051474 |
| NT01GM2683 | 8 | 0.82 | 19099183; 17654627; 17218763; 17208234; 9070288; 2675311; 1605723; 1894599; 2007581; 2380260 |
| NT01GM2869 | 15 | 0.92 | 21183673; 21150091; 21124821; 20942908; 20870771; 20855615; 20722734; 20639324; 20553556; 20521955 |
| NT01GM2964 | 5 | 0.97 | 21075841; 20473684; 20022138; 15579666; 17600048; 19746730; 19744922; 19678707; 19548980; 19372431 |
| NT01GM3158 | 4 | 0.82 | 21339285; 21323645; 21293029; 21215261; 21085634; 20971913; 20968139; 20947574; 20926497; 20849693 |
| NT01GM3365 | 8 | 0.93 | 5465052; 19094961; 19038229; 18694756; 3009448; 17597094; 17641188; 17109834; 16480720; 16157265 |
| NT01GM3382 | 9 | 0.95 | 21257793; 20077421; 20042190; 19915585; 19298700; 6099397; 17853487; 17393226; 16061200; 15854218 |
| NT01GO0052 | 4 | 0.71 | 21204254; 21178435; 20979332; 20946243; 20937821; 20935100; 20815357; 20708040; 20707000; 20693575 |
| NT01GO0702 | 19 | 0.80 | 20421293; 20347067; 20194587; 19929855; 19404677; 5821838; 2440807; 7655074; 7638195; 11792630 |
| NT01GO0813 | 18 | 0.74 | 20855745; 20399647; 20370506; 19910518; 2679354; 19713238; 6033613; 19592704; 19513693; 19494346 |
| NT01GO1645 | 8 | 0.83 | 19636558; 10406803; 17349966; 17074894; 16540118; 12473105; 11181712; 11004459; 10586875; 10491161 |
| NT01GO1826 | 4 | 0.78 | 21393220; 20975905; 20935126; 20487277; 20442221; 19481568; 1511879; 11591683; 19210620; 18042450 |
| NT01GO2395 | 13 | 0.94 | 21220307; 21082745; 20943400; 20882017; 20876530; 20856873; 20829344; 20617848; 20606262; 20601684 |
| NT01GO2498 | 8 | 0.76 | 19609583; 18281432; 17688437; 17668019; 17139509; 17030441; 11399090; 15882939; 15770479; 15548307 |
| NT01GO2555 | 8 | 0.73 | 21178285; 20811135; 19966484; 19546514; 19424620; 19200041; 17291445; 16402358; 16221991; 15996793 |
| NT01GO2902 | 8 | 0.78 | 21343423; 21329681; 21185310; 21078123; 21073854; 20931090; 20833539; 20809899; 20806931; 20736169 |
| NT01GOA0120 | 18 | 0.71 | 20949065; 17040490; 10920394; 11433449; 10805992; 6498172 |
| NT01GOA0141 | 12 | 0.92 | 21346419; 21334890; 21196781; 20809634; 20798596; 20727011; 20622895; 20202940; 20199576; 20148929 |
| NT01GT0558 | 9 | 0.89 | 21301088; 21185310; 21045289; 21035728; 20800575; 20583783; 20519910; 20443682; 20410062; 20303926 |
| NT01GT1985 | 8 | 0.95 | 19119913; 17904517; 16739990; 16343420; 16237515; 15115179; 10357231; 12605255; 11599178; 11334785 |
| NT01GT2033 | 8 | 0.73 | 20547751; 20516592; 20410293; 20370610; 20302306; 17847094; 20035104; 10995752; 19874713; 17213657 |
| NT01GT2473 | 9 | 0.84 | 20044264; 15819633; 11446992; 11171175; 9782513; 7579178; 8408076; 2049482; 16664382; 1054842 |
| NT01GT2680 | 19 | 0.90 | 942051; 12224521; 18266855; 17697984; 17420590; 17376078; 17310083; 17038794; 12626683; 16675219 |
| NT01GT3023 | 9 | 1.00 | 16506788; 16473891; 14766307; 10418145; 2066335; 1556094 |
| NT01GT3364 | 8 | 0.83 | 21397737; 21345800; 21338251; 21308351; 21306142; 21275052; 21265776; 21265736; 21247928; 21221720 |
| NT01GT3769 | 8 | 0.88 | 21393246; 21378180; 21349979; 21322495; 21322493; 21322492; 21310949; 21235502; 21190518; 21167156 |
| NT01GV0756 | 12 | 0.97 | 21320871; 21276865; 21258033; 21185091; 21054848; 20829225; 20599902; 20420910; 20377816; 19969024 |
| NT01GV1440 | 18 | 0.88 | 20081036; 3065452; 12646254; 10832632; 2168373 |
| NT01GV1664 | 1 | 0.73 | 21148420; 20851918; 20656812; 20562308; 18572426; 18176015; 17257267; 16301313; 12054251; 11034445 |
| NT01GV1835 | 8 | 0.82 | 18681889; 18237273; 17892308; 16820168; 16491912; 15900210; 15063311; 9660187; 9374858 |
| NT01GV2030 | 12 | 0.98 | 20451170; 18494037; 17761692; 17018519; 16175637; 15969742; 15128837; 10068683; 14670919; 12638128 |
| NT01GV2126 | 6 | 0.87 | 21380727; 21333322; 21314979; 21251231; 21037379; 21076181; 20565110; 20549720; 19506576; 11955436 |
| NT01GV2567 | 12 | 0.75 | 20596027; 19889619; 18420585; 18204430; 15782142; 11864566; 10611969; 11223033; 10644755; 10437795 |
| NT01GV3570 | 18 | 0.80 | 20395849; 20069550; 18471079; 17983207; 16584200; 16306165; 15904891; 1294062; 11749115; 11570890 |
| NT01GV3914 | 19 | 0.78 | 18854951; 12757950; 11841812; 8935665; 9144435; 8935665; 1658561; 8439670 |
| NT01GV4209 | 9 | 0.90 | 21248857; 20652826; 20622122; 20528952; 20086012; 20025994; 19898564; 19850005; 19656950; 11528392 |
| NT01GV4275 | 13 | 0.72 | 20400571; 19824612; 19787775; 19623961; 18957606; 17646390; 18957606; 16333325; 16269262; 15978086 |
| NT01HB0097 | 12 | 0.98 | 12382110; 20615993; 20595040; 20558234; 20467052; 20039036; 19725589; 19557795; 19425607; 18460472 |
| NT01HB0175 | 13 | 0.86 | 21354313; 21330537; 21330151; 21303906; 21285948; 21283762; 21265749; 21255212; 21245655 |
| NT01HB0281 | 12 | 0.79 | 20698901; 20546914; 19482134; 19003349; 16672363; 10499919; 10908637; 17609416; 17587813; 15654820 |
| NT01HB0363 | 13 | 0.94 | 21403913; 21403678; 21399665; 21385875; 21383696; 21378162; 21373201; 21349877; 21326952; 21326211 |
| NT01HB0482 | 13 | 0.74 | 12110595; 18552770; 17900615; 10722656; 16343540; 15210688; 11295541 |
| NT01HB0584 | 8 | 0.94 | 21199936; 21057456; 20849982; 20833784; 20804509; 20609064; 20378984; 20345183; 20235561; 20143804 |
| NT01HB0700 | 8 | 0.96 | 19943898; 16820168; 16027125; 8529639; 7543100; 7798224 |
| NT01HB0716 | 6 | 0.79 | 21288492; 21274582; 21245665; 21145896; 21123178; 21111748; 20884785; 20844047; 20719949; 20711169 |
| NT01HB0737 | 5 | 0.90 | 18991405; 18758731; 3896121; 15258168; 3309559; 17853355; 17519237; 17352498; 10613872; 16981690 |
| NT01HB0840 | 8 | 0.95 | 19119913; 17904517; 16739990; 16343420; 16237515; 15115179; 10357231; 12605255; 11599178; 11334785 |
| NT01HB1124 | 8 | 0.87 | 16343420; 12009405; 11480181; 9490067; 8902625 |
| NT01HB1125 | 8 | 0.87 | 16343420; 12009405; 11480181; 9490067; 8902625 |
| NT01HB1459 | 3 | 0.92 | 17355860; 16999833; 10359757; 10645945; 15049811; 9452389; 7715446; 11084348; 12407192; 10377148 |
| NT01HC0311 | 8 | 0.82 | 21362632; 21359959; 21276778; 21256041; 21238579; 21187465; 21037009; 20951799; 20942797; 20837989 |
| NT01HC1237 | 8 | 0.89 | 20655316; 19000608; 6684148; 17938906; 12604242; 12009906; 11514550; 393250; 4366025; 5674056 |
| NT01HC2620 | 12 | 0.71 | 16427708; 11802713; 8292665; 2917565; 3687922 |
| NT01HC3005 | 1 | 0.91 | 20389118; 9511756; 7731798; 9282744; 2527331 |
| NT01HC3391 | 15 | 0.90 | 21393369; 21320584; 21317318; 21315771; 21295603; 21284862; 21250657; 21246512; 21239493; 21217003 |
| NT01HC3514 | 8 | 0.93 | 16028369; 10592178; 15062771; 11012673; 9019138; 3109450 |
| NT01HC3538 | 13 | 0.79 | 8034696; 17614351; 17024311; 14741355; 16082410; 15581577; 15470111; 15301531; 11500486; 12818206 |
| NT01HC6230 | 18 | 0.87 | 21197505; 20949136; 20926757; 20876384; 20718731; 20671071; 20139708; 19827860; 19822435; 19729596 |
| NT01HD0504 | 8 | 0.78 | 20604566; 19833097; 19309100; 16788055; 16754998; 16691474; 15533835; 12417030; 10340844; 7706221 |
| NT01HD0734 | 18 | 0.98 | 16649212; 10802061; 9830000; 9774393; 9680959; 9304817; 9319211; 7789797; 7762614; 7496388 |
| NT01HD1496 | 18 | 0.94 | 20870774; 20467255; 9787636; 19095018; 17551219; 17159201; 17064369; 12563288; 16549425; 16524964 |
| NT01HH0964 | 8 | 0.72 | 21371603; 21371600; 21247988; 21033679; 20841500; 236638; 20071464; 16804059; 19300989; 18346472 |
| NT01HH1336 | 6 | 0.72 | 20974931; 20628285; 19004346; 17707226; 15572765; 10699505; 17912758; 17693399; 17327258; 11591160 |
| NT01HH1834 | 13 | 0.70 | 16237448; 9335267; 15213438; 10844675; 12429100; 12360533 |
| NT01HMA0089 | 8 | 0.89 | 20929961; 20413803; 20231855; 18719115; 17921179; 19520722; 19437546; 19348888; 19210957; 19038229 |
| NT01HMA0513 | 6 | 0.97 | 21273742; 21150323; 21098111; 21090780; 21078407; 21040652; 21035408; 20871811; 11842222 |
| NT01HMA1705 | 8 | 0.81 | 20831859; 20380465; 9504925; 20233940; 20184858; 20136502; 19961168; 19576290; 19248790; 10517866 |
| NT01HMA1883 | 8 | 0.73 | 21178285; 20811135; 19966484; 19546514; 19424620; 19200041; 17291445; 16402358; 16221991; 15996793 |
| NT01HMA1913 | 6 | 0.71 | 12741826; 16274984; 12953074; 12953085; 12805288; 12785867; 12717837; 12694127; 12663512; 12637151 |
| NT01HMA2134 | 4 | 0.77 | 21334427; 21198360; 20803137; 20420917; 20160049; 19951364; 19937156; 19673409; 19549172; 17158472 |
| NT01HMA2274 | 12 | 0.70 | 21329698; 20501794; 15843375; 19646995; 18519635; 18804477; 18346739; 16709864; 9767238; 16225851 |
| NT01HMA2458 | 8 | 0.86 | 21389104; 21385868; 21377462; 21371429; 21323311; 21300479; 21299470; 21282103; 21275844; 21252495 |
| NT01HMA2579 | 6 | 0.74 | 21384253; 21354178; 21353648; 21345667; 21310716; 21291520; 21278688; 21256960; 21241689; 21225639 |
| NT01HMA2590 | 14 | 1.00 | 20005278; 19735955; 4077987; 19299651; 18260100; 10074353; 17907270; 17439323; 10716711; 16985054 |
| NT01HMA2641 | 18 | 0.97 | 21397717; 21393220; 21385872; 21385626; 21384128; 21378194; 21377371; 21375706; 21371898; 21366702 |
| NT01HMA2723 | 4 | 0.72 | 19735955; 19412608; 17971082; 17908227; 15509585; 10521424; 2087222; 9988687; 3321061 |
| NT01HMA2751 | 8 | 0.94 | 19446023; 10103179; 10849007; 9704093; 15987839; 15949980; 12127981; 10509019; 2271542; 10679197 |
| NT01HMA2863 | 8 | 0.96 | 19586787; 19260967; 12795594; 11724561; 10767328; 9863634; 1908789; 9089910; 8504804; 1730230 |
| NT01HMA2894 | 12 | 0.97 | 20522495; 19696105; 19539664; 2646153; 11254569; 10096074; 9723914; 17208043; 8805079; 15223057 |
| NT01HMA3004 | 12 | 0.83 | 20826562; 19721756; 18759060; 16769036; 16395289; 16081615; 16024107; 1348724; 1971619; 10206887 |
| NT01HMA3099 | 18 | 0.97 | 21392495; 21369825; 21347827; 21304833; 21287809; 21276099; 21261938; 20607690; 21254069; 21262457 |
| NT01HMB0095 | 18 | 0.98 | 1741458; 16364320; 3156376; 8214582; 37402; 10447888; 6546423 |
| NT01HMB0142 | 17 | 0.75 | 21388532; 21126315; 20026132; 10656815; 18439859; 17449473; 15375644; 14690591; 1122947; 12435362 |
| NT01HMB0162 | 4 | 0.75 | 21364304; 21205308; 21035278; 12066186; 20467813; 20363791; 4020112; 17630835; 20172996; 16595014 |
| NT01HMB0192 | 8 | 0.87 | 21371425; 21303655; 20696867; 20639325; 20576519; 20570675; 20548048; 20363598; 20304328; 20304089 |
| NT01HMC0018 | 8 | 0.73 | 21178285; 20811135; 19966484; 19546514; 19424620; 19200041; 17291445; 16402358; 16221991; 15996793 |
| NT01HMC0039 | 18 | 0.95 | 21073695; 21051487; 20942908; 20623800; 20601430; 20596658; 20543096; 20470049; 20457836; 15770687 |
| NT01HMC0075 | 8 | 0.88 | 21388532; 21203490; 21194623; 20659890; 20405473; 20399281; 20378991; 20307095; 19367707; 19361226 |
| NT01HMC0096 | 8 | 0.70 | 21277857; 21184218; 20372835; 18793820; 15211509; 1137083; 17266949; 17137322; 16880594; 15803389 |
| NT01HMC0219 | 18 | 0.93 | 21082744; 18037401; 18559527; 10470083; 18300232; 17210706; 17074913; 11850253; 16756973; 16132345 |
| NT01HMC0350 | 8 | 0.90 | 20885442; 16665774; 12063017; 17891922; 16762453; 10581550; 15604729; 15164997; 15056475; 14555654 |
| NT01HP0464 | 12 | 0.87 | 21403131; 21399922; 21398604; 21394468; 21394287; 21391788; 21387509; 21385515; 21383917; 21378275 |
| NT01HP1698 | 18 | 0.76 | 21271607; 21216355; 21189132; 21078981; 21097885; 21106757; 20975670; 20937798; 20937233; 20812950 |
| NT01HS0062 | 12 | 0.70 | 21329698; 20501794; 15843375; 19646995; 18519635; 18804477; 18346739; 16709864; 9767238; 16225851 |
| NT01HS0087 | 12 | 0.84 | 21341515; 21313758; 21268712; 21265340; 21259244; 20890187; 21239051; 21239053; 21228176; 21226910 |
| NT01HS0120 | 12 | 0.95 | 20478922; 19243813; 18592176; 10545125; 16413316; 8625980; 10652445; 10574782; 10444550; 7669292 |
| NT01HS0245 | 12 | 0.78 | 19402045; 9603842; 17846036; 11836320; 18799682; 16926147; 10638745; 16617437; 10341140; 10637627 |
| NT01HS0500 | 14 | 0.89 | 21311411; 21295473; 21139397; 21076373; 21070810; 21062060; 21040798; 21035731; 20934342; 20848821 |
| NT01HS0717 | 19 | 0.80 | 18763123; 17368198; 9895306; 14695374; 12885960; 12626403; 10908670; 16668996; 10438455; 13835291 |
| NT01HS0867 | 13 | 0.74 | 20525789; 2265611; 10094308; 4556577; 9047363; 13221549; 7599277; 7689113; 2478711; 2471265 |
| NT01HS1058 | 13 | 0.98 | 21350339; 21143677; 21078665; 20978817; 20566885; 20528918; 20460460; 20363226; 20163168; 20101230 |
| NT01HS1605 | 18 | 0.87 | 21359673; 21187326; 21103346; 20663085; 20581866; 19497384; 19362814; 15189445; 14519844; 10542411 |
| NT01HS1613 | 13 | 0.96 | 20870726; 20835228; 20663952; 20696925; 11773626; 19734148; 18385375; 19439209; 10102815; 18816837 |
| NT01HS1769 | 8 | 0.77 | 11741948; 10387078; 9037110; 8866660; 7811234; 8468468; 5926184 |
| NT01HS1965 | 8 | 0.73 | 21178285; 20811135; 19966484; 19546514; 19424620; 19200041; 17291445; 16402358; 16221991; 15996793 |
| NT01HSB0181 | 1 | 0.77 | 20639325; 1478671; 16384793; 11461712; 18495640; 19183960; 16668324; 18600542; 18398873; 17355287 |
| NT01HW0431 | 8 | 0.86 | 21207115; 19889091; 19835359; 19432488; 14500511; 18944940; 17469189; 17240979; 16914555; 16036918 |
| NT01HW0595 | 18 | 0.88 | 9512653; 9651403; 10066480; 16734750; 10940251; 15820982; 10949309; 14734158; 14643200; 12968475 |
| NT01HW0634 | 8 | 0.86 | 21370474; 21188449; 21169447; 21115698; 21079674; 21075597; 21047399; 20866111; 20810510; 20739123 |
| NT01HW0683 | 8 | 0.75 | 20557447; 20369854; 20230247; 20219383; 19881207; 19759613; 19552509; 19217387; 16985102; 19276413 |
| NT01HW1222 | 18 | 0.72 | 21210973; 19966839; 17689557; 18512850; 15355342; 9576944; 12896996; 8588931 |
| NT01HW1507 | 8 | 0.74 | 20863064; 20690630; 20629638; 19762442; 19484273; 17676770; 17130127; 16900298; 15134887; 10672190 |
| NT01HW1761 | 6 | 0.96 | 21347786; 21172664; 20658645; 20634321; 20007604; 20298197; 20233728; 20227372; 20203129; 17684138 |
| NT01HW2458 | 8 | 0.73 | 21358191; 21354629; 21318381; 21307286; 21268708; 21262951; 21238431; 21106325; 21083040; 21082267 |
| NT01HW2793 | 6 | 0.92 | 21092102; 20587501; 20581194; 3074013; 19304752; 20054126; 17202163; 18931376; 11479932; 18931437 |
| NT01IH0548 | 8 | 0.74 | 19725515; 18579519; 18326045; 15659166; 15046979; 12634336; 7574488; 12423369; 7849603; 11744691 |
| NT01IL0040 | 1 | 0.79 | 20570618; 19478949; 18761702; 7476868; 10348851; 10563018; 12926249; 12736771; 9689222; 7502583 |
| NT01IL0101 | 15 | 0.87 | 20453093; 15522091; 18451049; 10579532; 11679669; 18096021; 18068682; 17712603; 10940041; 17320105 |
| NT01IL0118 | 18 | 0.89 | 20888343; 20888319; 20705604; 20639324; 20626554; 1658572; 20049509; 9353044; 6853475; 18771089 |
| NT01IL0181 | 8 | 0.85 | 20844759; 17375285; 17057342; 15647825; 12457410; 11955070; 11945122; 11642366; 11448970; 9501264 |
| NT01IL0191 | 8 | 0.82 | 20571026; 19862803; 19711960; 19628049; 17881823; 17291766; 1310545; 16917525; 16718678; 16448504 |
| NT01IL0308 | 8 | 0.73 | 21178285; 20811135; 19966484; 19546514; 19424620; 19200041; 17291445; 16402358; 16221991; 15996793 |
| NT01IL0416 | 12 | 0.91 | 21228211; 21178793; 21137246; 20967558; 20863401; 20861839; 20858750; 21080615; 20806045; 20722631 |
| NT01IL0478 | 18 | 0.88 | 20487273; 20111865; 17493798; 19581367; 1741458; 11831459; 10377096; 18297791; 9712811; 17119290 |
| NT01IL0594 | 8 | 0.73 | 21178285; 20811135; 19966484; 19546514; 19424620; 19200041; 17291445; 16402358; 16221991; 15996793 |
| NT01IL0597 | 14 | 1.00 | 20980241; 20831412; 11157898; 20082641; 20045992; 16322525; 19900465; 1644750; 17392337; 17919281 |
| NT01IL0714 | 8 | 0.91 | 21400100; 21397737; 21396889; 21396131; 21394044; 21393444; 21393246; 21393237; 21388872; 21387012 |
| NT01IL0800 | 18 | 0.94 | 20727012; 20679507; 18849422; 20220788; 20153847; 20111865; 20028819; 19843215; 8834871; 19713238 |
| NT01IL0940 | 9 | 0.96 | 21193572; 21057504; 20929961; 20866105; 20816087; 20727852; 20534481; 18476984; 20370610; 20334431 |
| NT01IL1060 | 9 | 0.86 | 19854834; 10840036; 19472174; 17555433; 11154287; 9634230; 10476972; 16710404; 16441657; 16161997 |
| NT01IL1194 | 15 | 0.87 | 19118355; 8982457; 1639496; 8200538; 8235653; 1527488; 6094484 |
| NT01IL1202 | 18 | 0.92 | 21397717; 21375706; 21369825; 21366702; 21315686; 21311724; 21310787; 21300772; 21282456; 21217007 |
| NT01IL1541 | 6 | 0.72 | 20935500; 20569003; 20472641; 20225163; 20225155; 20166751; 20116367; 20026132; 20016128; 18701081 |
| NT01IL1544 | 8 | 0.73 | 21178285; 20811135; 19966484; 19546514; 19424620; 19200041; 17291445; 16402358; 16221991; 15996793 |
| NT01IL1770 | 8 | 0.77 | 8989877; 12581215; 9933572; 1599458; 1550340; 6999791 |
| NT01IL1821 | 9 | 0.84 | 20506522; 20213520; 19922427; 19025871; 18546026; 17784853; 17669530; 17321147; 16314965; 15883872 |
| NT01IL2024 | 18 | 0.86 | 21371926; 21325274; 21147064; 20971850; 20804453; 20735480; 20718490; 20647000; 20639340; 20599691 |
| NT01IL2126 | 18 | 0.91 | 21315686; 21311724; 21177413; 21122131; 21111784; 21090806; 21078855; 21071707; 21059948; 21059651 |
| NT01IL2195 | 18 | 0.81 | 21315728; 21131908; 20937906; 20881245; 20879691; 20877283; 20870764; 20855745; 20828170; 20826447 |
| NT01IL2414 | 8 | 0.87 | 21296180; 21289278; 21242068; 21163439; 21048875; 20931523; 20831907; 20736162; 20697932; 20695200 |
| NT01IL2434 | 8 | 0.74 | 19132060; 18765906; 9786194; 18438923; 17377573; 14551431; 16686476; 16442642; 16232917; 16232847 |
| NT01IL2442 | 12 | 0.88 | 20659466; 17956229; 18034795; 17368855; 2263456; 10747959 |
| NT01IL2482 | 4 | 0.71 | 19695338; 19135125; 18709443; 9023936; 16763149; 15695811; 15222878; 10207070 |
| NT01IL2511 | 15 | 0.96 | 20874755; 20068355; 2435709; 8425216; 12907708; 13129941; 17884222; 17307737; 16487683; 1677357 |
| NT01JM0767 | 18 | 0.91 | 20519548; 19887446; 18701882; 16563799; 10464259 |
| NT01JM1078 | 12 | 0.87 | 20487289; 20059688; 19339102; 18995833; 18394994; 2137128; 16147871; 16076845; 14970903; 14673507 |
| NT01JM1116 | 18 | 0.98 | 21134356; 21103975; 21082379; 21062372; 20981744; 20938527; 20817945 |
| NT01JM1158 | 18 | 0.82 | 11309111; 10619994; 17221235; 8932698; 15667283; 37402; 6806240 |
| NT01JM2773 | 9 | 0.97 | 20334431; 19470521; 16430210; 14728675; 18005338; 17900701; 11524729; 10849007; 10869041; 9890906 |
| NT01JM3540 | 3 | 0.96 | 20616068; 20455262; 19423631; 1253620; 11163218; 10993081; 15105427; 17140412; 17007878; 16162504 |
| NT01JM3697 | 12 | 0.84 | 21080964; 21048804; 20707391; 16086015; 15072156; 19494339; 18421280; 15355989; 10411236; 17116701 |
| NT01JM3734 | 2 | 0.93 | 21190337; 21120472; 21081498; 20861021; 20690630; 20666399; 20112869; 20040598; 20039101; 20030377 |
| NT01JS0413 | 19 | 0.95 | 19617350; 8386125; 12519941; 11069912; 8358670; 7499229; 8748024; 1382035 |
| NT01JS0450 | 6 | 0.91 | 21245167; 21350155; 21126517; 20875449; 20705645; 11071951; 20192920; 20118257; 19234526; 1830580 |
| NT01JS0925 | 8 | 0.92 | 21068394; 21051545; 20690702; 20652669; 20652619; 20576606; 20099820; 19954240; 19904564; 19626710 |
| NT01JS1506 | 2 | 0.97 | 20968298; 20674574; 19222039; 20106967; 19883445; 19703421; 17207784; 19490096; 19476486; 19364324 |
| NT01JS2838 | 12 | 0.79 | 21254358; 21080431; 20815724; 20662919; 10423782; 20097680; 16388802; 19864597; 19657184; 17651389 |
| NT01KR0518 | 8 | 0.76 | 21054786; 20862316; 20832725; 20678977; 20498017; 20354154; 11444821; 19625634; 19498158; 19498162 |
| NT01KR0890 | 8 | 0.72 | 20233924; 20219606; 18969812; 17289415; 16634330; 15640082; 15182181; 12859020; 12480102; 11400062 |
| NT01KR1315 | 4 | 0.84 | 19635803; 20220788; 20217167; 20202936; 20015628; 19917674; 19557308; 19332830; 11386365; 10972825 |
| NT01KR2059 | 8 | 0.76 | 12198487; 19082940; 18639656; 16154088; 18279395; 18216065; 17848555; 17669423; 17466607; 17094969 |
| NT01KR2250 | 8 | 0.94 | 19195398; 7108955; 9585000; 7628554; 3481022 |
| NT01KR2616 | 19 | 0.74 | 21400688; 21400235; 21398593; 21393228; 21392988; 21390508; 21390257; 21388958; 21388880; 21388433 |
| NT01KR3073 | 8 | 0.85 | 21277857; 21228482; 21105363; 21042417; 20803137; 8923858; 20676631; 20632378; 20526482; 20361235 |
| NT01KR4037 | 8 | 0.85 | 21295699; 21259244; 21148142; 21148036; 21147988; 21217638; 21106951; 21106820; 21095589; 21087632 |
| NT01LA0045 | 18 | 0.96 | 21393174; 21369825; 21366542; 21351087; 21347827; 21342117; 21325265; 21216355; 21210849; 21209459 |
| NT01LA0401 | 15 | 0.98 | 11244084; 10865958; 10550204; 16207915; 8830275 |
| NT01LA0430 | 18 | 0.76 | 21392742; 21373967; 21217078; 21193742; 21187090; 21147991; 20973063; 20883206; 20857511; 20821325 |
| NT01LA0465 | 6 | 0.81 | 21129204; 21079776; 20170733; 20133362; 19921332; 19563117; 19217392; 16952955; 18057007; 9097419 |
| NT01LA0743 | 6 | 0.86 | 21326810; 20622468; 20584746; 20571792; 20554496; 20546727; 20499638; 20442410; 20420913; 20337945 |
| NT01LA0752 | 19 | 0.87 | 21400235; 21388958; 21380775; 21378160; 21369973; 21369910; 21367878; 21367655; 21357626; 21357429 |
| NT01LA0753 | 19 | 0.87 | 21400235; 21388958; 21380775; 21378160; 21369973; 21369910; 21367878; 21367655; 21357626; 21357429 |
| NT01LA0970 | 8 | 0.73 | 21400100; 21322493; 21322492; 21298284; 21277857; 21272640; 21271858; 21255723; 21233389; 21212468 |
| NT01LA0990 | 19 | 0.83 | 20863279; 20675481; 19671924; 19571171; 16505006; 16984883; 1060637; 15501784; 17032646; 16857734 |
| NT01LA1053 | 8 | 0.84 | 21391839; 21388699; 21388533; 21380803; 21376710; 21376665; 21372401; 21367494; 21359855; 21355597 |
| NT01LA1183 | 8 | 0.75 | 21396482; 21375592; 21357619; 21357486; 21338421; 21334970; 21333574; 21320074; 21319715; 21309870 |
| NT01LA1419 | 2 | 0.85 | 17335870; 16206477; 12736664; 11816029; 10094680; 9055989; 8640549 |
| NT01LB0124 | 19 | 0.89 | 21097635; 21040511; 20497501; 20497501; 20400548; 20173067; 18236038; 19170887; 18761696; 18490448 |
| NT01LB1641 | 8 | 0.92 | 20961996; 20957753; 19962210; 19853595; 19798499; 19294702; 19223009; 18584286; 18419176; 17027164 |
| NT01LB1899 | 15 | 0.91 | 21268903; 21225241; 21097630; 20935102; 20873228; 20709896; 20644332; 20543070; 20487268; 20435893 |
| NT01LB2165 | 8 | 0.76 | 21299839; 21163439; 20687335; 20654618; 20639204; 20552355; 20420880; 20361235; 20305000; 20213632 |
| NT01LC0104 | 9 | 0.98 | 21266581; 21238479; 21029305; 20919932; 20863951; 20846129; 20836903; 20833149; 20705608; 20687875 |
| NT01LC0746 | 8 | 0.80 | 21396917; 21366233; 21329684; 21310233; 21307643; 21300042; 21296056; 21281621; 21256830; 21245201 |
| NT01LC1869 | 8 | 0.85 | 21222452; 21148108; 20960080; 20862491; 20620191; 20610843; 20351055; 1687097; 20074091; 20036764 |
| NT01LC2865 | 12 | 0.84 | 20709901; 20023146; 8759852; 17929834; 9862121; 16561900; 7894706 |
| NT01LD0579 | 4 | 0.87 | 18506577; 16009133; 10982486; 7984101; 8412657 |
| NT01LD0857 | 15 | 0.94 | 21385202; 21347487; 21315771; 21246634; 21216906; 21193607; 21173175; 21039781; 21030435; 20979345 |
| NT01LD1005 | 1 | 0.96 | 21161522; 20352489; 9881164; 20102721; 20032584; 19963421; 19899082; 19747453; 19545238; 3933573 |
| NT01LD1285 | 1 | 0.76 | 12966138; 14964871; 7711027; 8012909; 8002714; 8098035; 1906928; 2153658; 2981222; 2834822 |
| NT01LD1569 | 14 | 0.81 | 16778368; 19775280; 18255096; 16551864; 16180016; 15504034; 12938148; 12771412; 12488542; 12406912 |
| NT01LH0164 | 18 | 0.90 | 20554841; 20552439; 19846751; 18419130; 15585581; 10051606; 17960831; 17255476; 12411949 |
| NT01LH0228 | 12 | 0.84 | 21325030; 20931338; 20800573; 20650410; 20232238; 20225247; 20206623; 20094994; 20044523; 19917054 |
| NT01LH1312 | 8 | 0.81 | 21270363; 21266482; 21247820; 21210071; 21177481; 21169389; 21161404; 21156199; 21147775; 21114891 |
| NT01LH1394 | 3 | 0.92 | 17355860; 16999833; 10359757; 10645945; 15049811; 9452389; 7715446; 11084348; 12407192; 10377148 |
| NT01LH1408 | 12 | 0.87 | 21074531; 19367726; 18989956; 12668614; 17604023; 17322486; 15136875; 15129268; 14584897; 14531822 |
| NT01LH1654 | 3 | 0.99 | 19884777; 17462920; 15682975; 16925785; 16631357; 15675069; 9074504; 10867658; 8828205; 8878042 |
| NT01LH1794 | 8 | 0.73 | 21265776; 20847256; 12368463; 18177021; 10467097; 16268586; 15789405; 9757107; 15260491; 14592717 |
| NT01LH2135 | 9 | 0.98 | 1355089; 15155740; 16212603; 10704200; 3556162; 1526981 |
| NT01LI0122 | 11 | 0.78 | 4977981; 9514719; 6427474; 18377930; 17602902; 10656821; 10612727; 9806496; 8744563; 8973529 |
| NT01LI0125 | 11 | 0.72 | 20963614; 20665904; 19009320; 2275851; 15669111; 15110522; 14507382; 11040123; 7873585 |
| NT01LI0217 | 12 | 0.77 | 21347827; 19901547; 19325157; 8962160; 17122039; 11427569; 20476959; 18357523; 17557115; 17259602 |
| NT01LI0232 | 19 | 0.79 | 19504047; 18036614; 17970751; 15470100; 15287736; 15283239; 15252718; 14981297; 12502707; 376499 |
| NT01LI0445 | 19 | 0.94 | 20798166; 19478450; 17955189; 16914364; 12645269; 11118302 |
| NT01LI0928 | 15 | 0.85 | 21362065; 19923733; 16209347; 10322028; 12950928; 11298281; 10383961; 2540676; 9786189; 8830262 |
| NT01LI1147 | 8 | 0.86 | 21245529; 21239588; 21103360; 20870711; 18332146 |
| NT01LI1647 | 6 | 0.91 | 19414020; 10652786; 11350954; 10652786; 3517933; 1097700; 7716189; 1374847 |
| NT01LI2189 | 8 | 0.94 | 20385644; 17158010; 10968285; 9805393; 9421252; 16453491; 1862930 |
| NT01LI2376 | 18 | 0.96 | 20737137; 20704181; 20658302; 20639324; 20561140; 20395595; 19775248; 19761223; 18606475; 19428657 |
| NT01LIA0004 | 6 | 0.92 | 21402158; 21245167; 21229291; 21189471; 21170958; 21151114; 21110832; 21073875; 21061089; 20979427 |
| NT01LJ0069 | 11 | 0.96 | 21118541; 17092825; 11076021; 16181782; 15604724; 15574470; 12948647; 12838611; 10835106; 9737923 |
| NT01LJ0071 | 15 | 0.87 | 21078847; 20622066; 20190050; 19589829; 10966457; 18954352; 18785057; 17418637; 10672179; 16764842 |
| NT01LJ0095 | 18 | 0.78 | 20395534; 19853682; 19063603; 18584243; 16702222; 16166347; 9389475; 12434407; 12388190; 11133151 |
| NT01LJ0274 | 18 | 0.88 | 21168419; 20837701; 19390537; 19290483; 19274306; 18650262; 2674121; 10839820; 17887176; 17520177 |
| NT01LJ0320 | 11 | 0.91 | 9384377; 17900620; 17540587; 17526845; 17322187; 16457869; 10408954; 4522805; 12634337; 15839401 |
| NT01LJ0438 | 2 | 0.95 | 10880976; 10545188; 9756625; 9748348; 7629164; 6413254 |
| NT01LJ0621 | 19 | 0.84 | 8309940; 17504469; 2446923; 2256682; 14606945; 11191810; 8238872; 10816581; 10361281; 9682471 |
| NT01LJ0695 | 4 | 0.94 | 19788546; 18391964; 9495771; 17360038; 10648549; 9252185 |
| NT01LJ1286 | 18 | 0.97 | 21378181; 21373771; 21373769; 21371522; 21369940; 21368142; 21367787; 21366347; 21366300; 21362118 |
| NT01LJ1318 | 18 | 0.73 | 21175590; 20428776; 20103563; 20072950; 20020128; 19885582; 19654441; 19643774; 19548284; 12079203 |
| NT01LJ1571 | 11 | 0.83 | 19921332; 17034960; 9421491; 10079521; 8224896 |
| NT01LJ1899 | 18 | 0.77 | 20925249; 20810280; 20656486; 20053560; 19328290; 18984587; 18644203; 18641186; 17852870; 17603058 |
| NT01LL0261 | 15 | 0.83 | 9584201; 20400852; 17555441; 20064064; 17591613; 11918807; 8946956; 17317630; 19246615; 10400604 |
| NT01LL1100 | 18 | 0.99 | 21369825; 21366542; 21347827; 21304833; 21247706; 21262457; 21187898; 21163262; 21098265; 21090806 |
| NT01LL1542 | 19 | 0.94 | 20843801; 3611062; 20364833; 20188057; 20077550; 20030628; 19929855; 19720067; 19277539; 19245333 |
| NT01LL2318 | 18 | 0.96 | 21187068; 20826797; 9680201; 19826225; 18190691; 18430260; 17646927; 17583799; 17437113; 17266728 |
| NT01LL2465 | 6 | 0.72 | 21369766; 21319196; 21293478; 21266827; 21122797; 20966874; 20926398; 20735435; 20670616; 20652500 |
| NT01LL2704 | 19 | 0.92 | 21284754; 21111546; 21040511; 20851903; 20829286; 20825156; 20680265; 20676630; 20637315; 20610395 |
| NT01LP0642 | 12 | 0.74 | 21372759; 21320869; 21289624; 21287809; 21279994; 21270253; 21268566; 21266412; 21259244; 21228176 |
| NT01LP0649 | 11 | 0.80 | 20802084; 19904424; 18078973; 8756719; 2495760; 11384223; 6403500; 7998420; 7932776 |
| NT01LP0672 | 15 | 0.82 | 20889551; 20821243; 20732878; 20711226; 20576316; 12802337; 20498137; 20417603; 20354764; 20150914 |
| NT01LP1260 | 6 | 0.89 | 11048718; 19840758; 19735921; 18977316; 10861942; 1495996; 11773415; 17150583; 15246274; 16359314 |
| NT01LP1514 | 1 | 0.86 | 20961734; 19922747; 18007551; 17139593; 20507450; 16163487; 10533754; 11319580; 11223945; 8922917 |
| NT01LP1563 | 18 | 0.84 | 21398557; 21398489; 21396912; 21394477; 21393845; 21393450; 21391724; 21385992; 21383000; 21379337 |
| NT01LP2989 | 14 | 0.98 | 8902269; 8558524; 517739; 2044194; 2026165; 3262341 |
| NT01LS0257 | 8 | 0.88 | 19191966; 18811651; 17259621; 9572874; 9930981; 9467910; 9311140; 9168623; 4896022; 8241148 |
| NT01LS0579 | 4 | 0.71 | 21331631; 21128945; 20807374; 20507936; 20375108; 20358223; 20348054; 20211220; 19947981; 19880399 |
| NT01LS1325 | 19 | 0.75 | 17721845; 17567447; 17017480; 16836671; 12729795; 12465432; 12220067; 12086211; 12026535; 11450148 |
| NT01LS1417 | 13 | 0.70 | 16237448; 9335267; 15213438; 10844675; 12429100; 12360533 |
| NT01LS1649 | 4 | 0.72 | 21241361; 16956068; 17623030; 14680711; 12931342; 8650219; 12123459; 7542800; 9579381 |
| NT01LW0013 | 8 | 0.83 | 19734178; 3148839; 12578386; 12530544; 10563797; 9737851; 9493381; 9428682; 9298948; 9219517 |
| NT01LW0019 | 9 | 0.92 | 21393244; 21387258; 21369973; 21344529; 21343303; 21342713; 21308848; 21274617; 21267636; 21255302 |
| NT01LW0338 | 12 | 0.75 | 21379326; 21247889; 21242965; 21231916; 21209184; 21205201; 21147937; 21125667; 21121894; 21113270 |
| NT01LW0379 | 18 | 0.90 | 21397044; 21358085; 21355371; 21335451; 21318893; 21299645; 21278273; 21262549; 21256773; 21253597 |
| NT01LW0380 | 18 | 0.90 | 21397044; 21358085; 21355371; 21335451; 21318893; 21299645; 21278273; 21262549; 21256773; 21253597 |
| NT01LW0669 | 4 | 0.78 | 20132451; 8071222; 18957286; 10995478; 17090391; 16720646; 10089417; 15699192; 15276839; 15136044 |
| NT01LW0893 | 17 | 0.97 | 21277915; 20979331; 20439472; 16113269; 18061613; 19575568; 19478430; 19014275; 18757816; 9721294 |
| NT01LW0965 | 12 | 0.86 | 21388432; 21386961; 21375177; 21372759; 21365808; 21360755; 21321349; 21347511; 20979473; 21344734 |
| NT01LW0971 | 13 | 0.76 | 20106954; 10196363; 19712588; 19664587; 18424795; 18069966; 7721763; 17466622; 12655133; 17900615 |
| NT01LW1026 | 6 | 0.71 | 21362064; 21360181; 21304599; 21299643; 21295415; 21267402; 21257771; 21249192; 21245528; 21243338 |
| NT01LW1051 | 8 | 0.96 | 21374701; 21354098; 21296938; 21253866; 21238944; 21237726; 21188974; 21168410; 21057948; 20960712 |
| NT01LW1159 | 8 | 0.74 | 21037291; 20832411; 20724492; 20665426; 20656883; 20228169; 20139187; 20044564; 19782502; 19765784 |
| NT01LW1290 | 19 | 0.84 | 8309940; 17504469; 2446923; 2256682; 14606945; 11191810; 8238872; 10816581; 10361281; 9682471 |
| NT01LW1379 | 8 | 0.77 | 21107318; 20933183; 20456655; 20124719; 20089767; 19860829; 19084582; 18581728; 18549703; 17993624 |
| NT01LW1610 | 12 | 0.94 | 9847218; 7763298; 10455123; 7628708; 3510201 |
| NT01LW2005 | 6 | 0.84 | 21388532; 21365542; 21350762; 21343909; 21336027; 21325134; 21321231; 21091440; 21086493; 21078962 |
| NT01LW2066 | 15 | 0.96 | 21315771; 21284862; 21283517; 21257771; 21235644; 21050859; 21039781; 20946846; 20942908; 20929957 |
| NT01LW2302 | 6 | 0.73 | 21278727; 16430210; 17175108; 10878254; 17291766; 6337994; 16570853; 16482161; 352392; 2544809 |
| NT01LW2631 | 13 | 0.96 | 11141312; 20171175; 15016989; 18850066; 993776; 17493334; 16337202; 15846068; 15357103; 6760806 |
| NT01LX0161 | 6 | 0.76 | 21394635; 21389894; 21389349; 21383990; 21383065; 21381982; 21379330; 21378168; 21373969; 21372752 |
| NT01LX0538 | 8 | 0.75 | 17314104; 16365091; 11945122; 11368334; 11168412; 10832633; 10385636; 9546032; 8652022; 7896739 |
| NT01LX0888 | 19 | 0.94 | 21367611; 21366294; 21355999; 21341216; 21287229; 21258004; 21253720; 21223393; 21193007; 21188612 |
| NT01LX1356 | 19 | 0.82 | 21399861; 21399604; 21398658; 21393344; 21392539; 21392092; 21389981; 21388709; 21386062; 21385868 |
| NT01LX1370 | 8 | 0.87 | 21393235; 21375708; 21365755; 21324604; 21237294; 21136951; 21131059; 21045562; 20967536; 21080591 |
| NT01LX1706 | 18 | 0.81 | 21335451; 21278108; 21204808; 21147915; 21104926; 21077204; 21073695; 21062372; 21051487; 21050923 |
| NT01LX2392 | 19 | 0.97 | 21193828; 20960239; 20720016; 20703955; 20562284; 20467831; 19883124; 19783787; 19527927; 18997327 |
| NT01MB0097 | 8 | 0.86 | 21187144; 21185288; 1849603; 19720045; 18458828; 18335216; 17669368; 17668201; 11751810; 16776699 |
| NT01MB1293 | 6 | 0.88 | 20211667; 19115962; 18977316; 11158121; 9311918; 16585648; 15338545 |
| NT01MB1842 | 12 | 0.83 | 21142294; 20489149; 20369790; 20188882; 19656111; 19255851; 18729835; 18713951; 18260911; 17884933 |
| NT01MB2559 | 8 | 0.82 | 21077373; 19777264; 19581367; 19523599; 18482575; 16524901; 18952156; 18602174; 18311572; 18272530 |
| NT01MB2629 | 19 | 0.98 | 20802066; 20562284; 20057070; 19626289; 19280987; 16653123; 18559265; 18337693; 18309273; 17234634 |
| NT01MB2755 | 18 | 0.75 | 21322495; 21223949; 21192931; 21106832; 21090806; 21059651; 21049012; 20886313; 20846911; 20829431 |
| NT01MB2972 | 13 | 0.72 | 20400571; 19824612; 19787775; 19623961; 18957606; 17646390; 18957606; 16333325; 16269262; 15978086 |
| NT01MB3448 | 8 | 0.79 | 20959113; 20926707; 18545270; 19154484; 19006332; 16608357; 18499056; 18355283; 17964535; 15039328 |
| NT01MC0019 | 13 | 0.99 | 21357927; 21321019; 21222438; 21098258; 21149735; 20942128; 20504770; 20387531; 20160114; 20059602 |
| NT01MC0103 | 15 | 0.96 | 21357485; 21310787; 21303432; 21183673; 21124821; 21098029; 20942908; 20870771; 20855615; 20847938 |
| NT01MC0191 | 8 | 0.71 | 20716364; 20601085; 20586183; 6121318; 20026209; 19537206; 19454243; 19219570; 19043360; 18793820 |
| NT01MC0220 | 18 | 0.91 | 21387012; 21334054; 21170879; 21278129; 21268658; 21262798; 21236240; 21216025; 21187068; 21153767 |
| NT01MC0355 | 18 | 0.77 | 21228159; 21187398; 21129771; 21129209; 21107421; 21104093; 20860462; 20858774; 20858777; 20825633 |
| NT01MC0383 | 6 | 0.97 | 21387457; 21386659; 21374739; 21364188; 21347226; 21346195; 21326362; 21317888; 21301224; 21288002 |
| NT01MC0422 | 6 | 0.91 | 21047769; 18262734; 16510450; 16390238; 16122992; 15850673; 15578504; 10383961; 12974384; 12869651 |
| NT01MC0427 | 4 | 0.72 | 21062468; 18081840; 4957395; 11705377; 10672186; 10524254; 10510225; 9643537; 9765799; 7724572 |
| NT01MC0447 | 12 | 0.88 | 21315085; 21118527; 20947885; 20874569; 20817636; 20732391; 20639698; 20607520; 20604530; 20601056 |
| NT01MC0464 | 8 | 0.72 | 12892110; 17547421; 17185537; 16995898; 15870478; 14645228; 12147345; 11863431; 10400577; 11523998 |
| NT01MC0488 | 15 | 0.97 | 21169497; 21124821; 20946846; 20870771; 20817745; 20805330; 20385539; 20160049; 20148929; 20093290 |
| NT01MC0582 | 15 | 0.96 | 21217003; 21173175; 18516236; 15033981; 15599505; 12650455; 9585513; 9776758 |
| NT01MC0691 | 13 | 0.84 | 21336932; 21282426; 21205898; 20974979; 20498377; 20190824; 16218899; 20163021; 19841848; 16277600 |
| NT01MC0839 | 18 | 0.83 | 16043697; 19874424; 19841502; 9182806; 19580841; 16354698; 19074639; 17546510; 18239147; 18042255 |
| NT01MC0880 | 19 | 0.76 | 21070855; 20609359; 20163137; 19623961; 10545329; 11418550; 10542091; 1885544; 18173803; 17910958 |
| NT01MC0882 | 18 | 0.98 | 21378036; 21351087; 21347827; 21336797; 21304833; 21301930; 21300772; 21293047; 21283517; 21282456 |
| NT01MC0900 | 19 | 0.80 | 15977277; 14761630; 11096116; 10521660; 10411277; 11553351; 11517612; 7574484; 11065359; 10895688 |
| NT01MC0914 | 15 | 0.91 | 21385202; 21124821; 21062468; 20974818; 20946846; 20942908; 20817745; 20629796; 20501872; 20447287 |
| NT01MC0969 | 15 | 0.87 | 21350472; 21264306; 21114961; 21036245; 21030591; 20924200; 20883745; 20864477; 20702764; 20691271 |
| NT01MC1074 | 15 | 0.96 | 21357485; 21310787; 21303432; 21183673; 21124821; 21098029; 20942908; 20870771; 20855615; 20847938 |
| NT01MC1086 | 1 | 0.70 | 20660776; 20381632; 20372887; 19897476; 18565285; 18470486; 5700707; 18006325; 17981822; 17909870 |
| NT01MC1094 | 15 | 0.72 | 20376102; 15937186; 18248418; 16154092; 11591683; 11703664; 1459461; 10517599; 10448677; 10089538 |
| NT01MC1148 | 11 | 0.85 | 17556756; 16816399; 16233010; 16228364; 16134325; 15384490; 12938040; 8801430 |
| NT01MC1230 | 8 | 0.74 | 21317262; 16790015; 15386115; 11167002; 11006847; 3141411; 9079890; 8736535; 1938911; 7030314 |
| NT01MC1253 | 15 | 0.82 | 21385202; 20942908; 19832907; 7989325; 18391440; 10500846; 20507528; 17435999; 16773556; 16573700 |
| NT01MC1284 | 9 | 0.99 | 18585998; 15982005; 12896875; 10960477; 10508782; 9714757; 9115637; 8557688; 8824210; 8858564 |
| NT01MC1304 | 11 | 0.90 | 15733918; 12781722; 8794874; 8175794; 3159906 |
| NT01MC1336 | 1 | 0.81 | 20086163; 19153452; 19072614; 18216013; 17927957; 9721288; 16708165; 16313180; 16232856; 16216079 |
| NT01MC1377 | 15 | 0.98 | 16483137; 14984021; 11554742; 8755540; 10544147; 2764573; 10209742; 9648849; 9278440; 8750828 |
| NT01MC1405 | 15 | 0.86 | 21393212; 21390327; 21386983; 21383239; 21378189; 21375706; 21369994; 21369825; 21364304; 21355787 |
| NT01MC1438 | 15 | 0.89 | 10966457; 19748334; 10673004; 12657056; 11844754; 11937029; 9813128; 10500846; 8969172; 9878429 |
| NT01MC1455 | 14 | 0.97 | 20544509; 20382023; 20380929; 17701900; 19918830; 19754463; 19186537; 19161981; 19131690 |
| NT01MC1511 | 15 | 0.88 | 18977359; 14561776; 11179370; 14651344; 12650455; 12460564; 11931559; 11931556; 11873906; 11748231 |
| NT01MC1525 | 12 | 0.93 | 19650643; 10485712; 10383442; 9428517; 9601038; 9067252; 7713911; 8027082; 2197275; 3049606 |
| NT01MC1531 | 6 | 0.98 | 20889748; 18726173; 16303848; 19587535; 8387604; 11124263; 18186484; 17980605; 17728255; 1324925 |
| NT01MC1611 | 12 | 0.77 | 21399659; 21385720; 21382349; 21376040; 21368888; 21364884; 21360476; 21324900; 21317537; 21308747 |
| NT01MC1681 | 18 | 0.72 | 10470043; 12662939; 9521785; 1738310; 2722818; 271968; 1791754; 1994034; 2184029; 2981624 |
| NT01MC1687 | 4 | 0.71 | 21139141; 21084450; 21080032; 20883793; 20858866; 20845092; 20799341; 20708625; 20603107; 9632527 |
| NT01MC1699 | 12 | 0.80 | 8087850; 10582129; 6384729; 8462840; 2679887; 8387148; 1588819; 2088168; 2041472; 2170107 |
| NT01MC1708 | 8 | 0.74 | 19081846; 18652881; 18266760; 17594457; 17290794; 16798780; 16341707; 16228901; 19262876; 15120114 |
| NT01MC1723 | 19 | 0.98 | 20831592; 20688825; 20622068; 20566690; 20056708; 10829079; 19332829; 19201821; 17517879; 18833547 |
| NT01MC1778 | 15 | 0.91 | 20963440; 15937186; 12855715; 8830274; 10866802; 3086314 |
| NT01MC1779 | 15 | 0.81 | 20963440; 15937186; 18248418; 8830274; 16385125; 4604283; 1447208; 8335256; 8432742; 2241934 |
| NT01MC1783 | 18 | 0.85 | 3329281; 17196220; 3549717; 11535604; 10589712; 8900068; 8628229; 1917927; 1956288; 2676709 |
| NT01MC1785 | 19 | 0.76 | 21395597; 20436292; 19015727; 17706299; 19073578; 18756098; 18346723; 18210176; 1008746; 16797542 |
| NT01MC1834 | 8 | 0.88 | 21326935; 21300042; 21277289; 21270753; 21214177; 21185270; 21135126; 21134280; 21113494; 21112834 |
| NT01MC1972 | 2 | 0.95 | 11461190; 7635153; 8590013; 7763270; 8001680; 7923811; 8188266; 8444860 |
| NT01MC2077 | 6 | 0.71 | 21298832; 21076033; 21169503; 20921377; 20952417; 20940181; 20962275; 20890847; 20890836; 20846831 |
| NT01MC2114 | 12 | 0.94 | 18342411; 17239953; 16573693; 16076848; 15170231; 12081643; 11860552; 6194404 |
| NT01MC2150 | 6 | 0.71 | 21193388; 18931376; 18838147; 18331470; 18062262; 9473031; 17660407; 17218011; 17180708; 16972870 |
| NT01MC2218 | 6 | 0.86 | 21326810; 20622468; 20584746; 20571792; 20554496; 20546727; 20499638; 20442410; 20420913; 20337945 |
| NT01MC2299 | 8 | 0.90 | 19650882; 18606812; 15637070; 15592456; 11798281; 12180974; 11443117; 11171647; 11064128; 10426373 |
| NT01MC2301 | 12 | 0.89 | 21395536; 21387826; 21376122; 21369703; 21344411; 21342114; 21339174; 21328067; 21307569; 21307147 |
| NT01MC2393 | 9 | 0.79 | 20418430; 20178986; 19664929; 19472174; 2580220; 19095065; 19083031; 17555433; 18824113; 18721141 |
| NT01MC2423 | 8 | 0.78 | 20383618; 20093047; 19819302; 19596066; 18498061; 10913250; 7689641; 12736690; 17927566; 17709548 |
| NT01MC2455 | 2 | 0.75 | 18083805; 3032913; 4874308; 15943815; 14898026; 9446573; 7929373; 8425548 |
| NT01MC2461 | 2 | 0.94 | 19754882; 19267692; 16289918; 15522295; 11215515; 11215515; 10579529; 10217486; 2077690; 9778368 |
| NT01MC2467 | 2 | 0.92 | 18391406; 16866557; 15522295; 9665173; 9660189; 9331403; 9224567; 7606163; 1731915; 8405386 |
| NT01MC2508 | 19 | 0.71 | 6295886; 17120230; 17004716; 1569582; 15299374; 16236703; 15200939; 14659546; 12757953; 12628682 |
| NT01MC2568 | 12 | 0.90 | 21125796; 21094166; 21051851; 20950979; 20844218; 20631311; 20631135; 10859029; 20383012; 20299243 |
| NT01MC2626 | 19 | 0.79 | 20661555; 20638314; 20589904; 20512975; 20507884; 19235233; 19122187; 18824121; 18690721; 18664520 |
| NT01MC2644 | 3 | 0.99 | 20971868; 20738375; 20345659; 19103276; 10944393; 9786194; 18599841; 1060637; 772168; 17468768 |
| NT01MC2657 | 15 | 0.92 | 21183673; 21150091; 21124821; 20942908; 20870771; 20855615; 20722734; 20639324; 20553556; 20521955 |
| NT01MC2662 | 8 | 0.85 | 19459782; 17568678; 16869557; 174718; 12459896; 11021927; 7781769; 8396649; 2831975; 3030432 |
| NT01MC2664 | 4 | 0.81 | 21367479; 21257033; 21203423; 21125383; 20950979; 20935127; 20368405; 20233932; 20199592; 20185542 |
| NT01MC2683 | 6 | 0.94 | 21092102; 21030504; 20622503; 20587501; 3074013; 1741244; 2555265; 17126598; 19304752; 15006762 |
| NT01MC2690 | 8 | 0.94 | 16450109; 18245085; 15491156; 10320675; 12427946; 12196025; 1380014; 10830505; 10571051; 9748316 |
| NT01MC2692 | 8 | 0.84 | 11274111; 15316720; 1310545; 10548510; 9729445; 9022686; 7664887; 7883189; 7765251; 1468562 |
| NT01MC2715 | 6 | 0.97 | 21319705; 21310243; 21254781; 21229879; 21205687; 21177431; 21177427; 21173034; 21169356; 21143350 |
| NT01MC2764 | 12 | 0.73 | 21352861; 21324157; 21270265; 21257463; 21240922; 21228165; 21228159; 21197403; 21179864; 21172613 |
| NT01MC2862 | 12 | 0.80 | 20668094; 20335479; 15221451; 14991425; 10358092; 9559550; 9372188; 9325105; 1679318; 1349602 |
| NT01MC2865 | 18 | 0.90 | 21397717; 21378194; 21369825; 21365259; 21265778; 21233160; 21192796; 21183643; 21139203; 21122159 |
| NT01MC2896 | 4 | 0.73 | 21332625; 21325038; 21273249; 21253572; 21219416; 21204905; 21109564; 21094508; 20965571; 20868693 |
| NT01MC3024 | 14 | 0.82 | 19935919; 19921932; 19900465; 19575694; 18392760; 17053145; 17686772; 6538402; 17434429; 16755134 |
| NT01MC3081 | 15 | 0.91 | 21385202; 21124821; 21062468; 20974818; 20946846; 20942908; 20817745; 20629796; 20501872; 20447287 |
| NT01MC3102 | 18 | 0.92 | 21397717; 21375706; 21369825; 21366702; 21315686; 21311724; 21310787; 21300772; 21282456; 21217007 |
| NT01MC3134 | 18 | 0.84 | 21276097; 21179522; 21157514; 20802073; 20799747; 16704341; 20420522; 20227482; 19919671; 10209752 |
| NT01MC3135 | 18 | 0.91 | 21276097; 21179522; 20802073; 20799747; 20420522; 20227482; 19919671; 10209752; 19199920; 19119856 |
| NT01MC3144 | 9 | 0.91 | 21115502; 20110695; 18760846; 18640292; 18485779; 18031367; 17997341; 17719200; 17199921; 17116638 |
| NT01MC3145 | 9 | 0.88 | 10482673; 12454267; 10828604; 9892232; 9804328; 9748261; 9165098; 7668351; 8441459 |
| NT01MC3148 | 6 | 0.78 | 21400651; 21390146; 21389155; 21387901; 21384194; 21383530; 21371140; 21370012; 21369955; 21368204 |
| NT01MC3253 | 13 | 0.73 | 19627989; 10810734; 17083917; 15822125; 12731872; 12697167; 12533518; 10810734; 10739928; 9766225 |
| NT01MC3296 | 5 | 0.83 | 21231969; 19840103; 10613860; 16756317; 18027933; 10506203; 17163967; 16932909; 15213441; 12892890 |
| NT01MC3306 | 5 | 0.93 | 21393366; 21393052; 21392197; 21382037; 21375387; 21365232; 21357454; 21338415; 21334214; 21322495 |
| NT01MC3316 | 5 | 0.93 | 19772354; 18697949; 18314963; 16228203; 9418236; 9236774; 7954845; 8393789; 8316214; 2162195 |
| NT01MC3333 | 2 | 0.82 | 20057142; 20051244; 19508223; 19409940; 8755509; 2991702; 9559985; 18050926; 17473883; 17401144 |
| NT01MC3350 | 15 | 0.98 | 21398634; 21396957; 21393212; 21375718; 21375708; 21354180; 21344396; 21339296; 21325273; 21284756 |
| NT01MC3477 | 8 | 0.81 | 19783691; 19412686; 19282820; 17301062; 16977511; 16920413; 16359119; 16325144; 15897195; 12768344 |
| NT01MC3542 | 18 | 0.76 | 21294449; 21126349; 20973408; 20884691; 20662775; 20442959; 20136973; 19894125; 17965213; 19659660 |
| NT01MC3545 | 5 | 0.88 | 20635345; 20631318; 20207756; 10194322; 7565414; 10368287; 16404152; 2211515; 14981304; 14749331 |
| NT01MC3599 | 8 | 0.86 | 21073854; 20515113; 19428350; 19177216; 19140015; 19011745; 18850316; 18422649; 17516063; 17203966 |
| NT01MC3637 | 6 | 0.95 | 21389547; 21389352; 21388532; 21388492; 21387067; 21386365; 21380757; 21378962; 21375739; 21372173 |
| NT01MC3646 | 19 | 0.72 | 21341175; 21331046; 21205637; 21302401; 21280564; 21279994; 21277993; 21273067; 21269535; 21256827 |
| NT01MC3662 | 1 | 0.96 | 21161522; 20352489; 9881164; 20102721; 20032584; 19963421; 19899082; 19747453; 19545238; 3933573 |
| NT01MC3697 | 15 | 0.83 | 18045853; 17911379; 15797652; 11180822; 7473772; 8229239; 1848270; 1974845; 6087123; 6245715 |
| NT01MC3778 | 18 | 0.79 | 20857686; 19091722; 20051028; 20029328; 19639865; 19184417; 19182356; 19118356; 19008136; 18719175 |
| NT01MC3822 | 11 | 0.88 | 21244532; 21087627; 21062824; 17379577; 16024079; 15165236; 3000073; 11320127; 1396573; 9141688 |
| NT01MC3911 | 19 | 0.82 | 21193672; 20871101; 20553325; 20061477; 20030726; 19682076; 18312393; 5323018; 15980069; 14651342 |
| NT01MC3925 | 19 | 0.81 | 21288267; 21273488; 21168419; 21127404; 21070836; 21068446; 21056541; 21047786; 21044320; 21040561 |
| NT01MC3943 | 19 | 0.87 | 21397493; 21385202; 21342541; 21347376; 21317159; 21203384; 21166709; 21126315; 21106106; 21102601 |
| NT01MC4078 | 8 | 0.74 | 1148211; 12628239; 8846223; 1943775; 8574415; 8533474; 1659648 |
| NT01MC4172 | 15 | 0.98 | 21380435; 21362064; 21336656; 21320584; 21300775; 21295603; 21255106; 21250657; 21219471; 21216996 |
| NT01MC4179 | 6 | 0.79 | 16083981; 9799358; 11809766; 10219083; 10097083; 10751307; 10219083; 2192864; 1657977; 2569765 |
| NT01MC4187 | 15 | 0.98 | 20946846; 20817745; 20148929; 20127467; 19411418; 17183208; 6550579; 12614149; 17908933; 7989325 |
| NT01MC4252 | 18 | 0.98 | 15388932; 10403413; 8033999; 3943911; 7018904 |
| NT01MC4301 | 1 | 0.78 | 21114891; 21070756; 21048403; 20861582; 20853870; 20853735; 20724492; 20662904; 20558164; 20549016 |
| NT01MC4332 | 8 | 0.90 | 20557574; 20202167; 16880; 19705487; 15685292; 19505081; 2611230; 15536523; 19170242; 15642712 |
| NT01MC4375 | 8 | 0.76 | 20053183; 16232605; 15308491; 15086813; 15032819; 14756538; 14754917; 14719043; 12936925; 1598191 |
| NT01MC4393 | 8 | 0.93 | 21400238; 21393052; 21390036; 21384925; 21380435; 21370354; 21368150; 21367862; 21365722; 21365080 |
| NT01MC4418 | 12 | 0.73 | 21347346; 21294679; 21293192; 21276198; 21262504; 21246635; 21241231; 21217173; 21228468; 21221970 |
| NT01MC4535 | 12 | 0.80 | 21400101; 21399567; 21399492; 21393844; 21388709; 21385615; 21383157; 21382368; 21381077; 21380642 |
| NT01MC4638 | 14 | 0.71 | 21187411; 20929557; 20605980; 20574830; 20498911; 20473969; 20452222; 20441167; 20424759; 20369850 |
| NT01MC4652 | 13 | 0.94 | 21148395; 21135037; 21030830; 20937566; 20807199; 20736336; 20598889; 20598766; 20507976; 20363226 |
| NT01MC4660 | 14 | 0.93 | 18388293; 17506728; 17023639; 16865708; 16544324; 15566465; 15060594; 9328467; 1398079; 8224889 |
| NT01MC4691 | 9 | 0.95 | 21389045; 21284260; 21204617; 21034554; 20707865; 20655933; 20625797; 20610563; 20601073; 20600859 |
| NT01MC4719 | 15 | 0.98 | 20870771; 20855615; 20722734; 20639324; 20521955; 20348251; 20093290; 6550579; 7010115; 12614149 |
| NT01MC4786 | 6 | 0.81 | 21211815; 21183667; 21174981; 20724137; 20586430; 20442958; 20178784; 20154125; 14976242; 20110293 |
| NT01MC4806 | 15 | 0.93 | 20656781; 20118252; 10966457; 18792681; 12453229; 10781568; 16949866; 9781871; 12123461; 10094700 |
| NT01MC4864 | 6 | 0.92 | 21388139; 21383100; 21354180; 21350762; 21338149; 21329318; 21318561; 21301105; 21298832; 21292748 |
| NT01MC4886 | 2 | 0.95 | 20861021; 1532388; 19415239; 19368556; 3322275; 15572765; 10829079; 11004177; 10978347; 17201004 |
| NT01MC4899 | 2 | 0.82 | 21335425; 21335099; 21321112; 21308763; 21290987; 21287164; 21224049; 21160477; 21122070; 21104984 |
| NT01MG0328 | 5 | 0.88 | 12207230; 9503607; 7947754; 8492805; 2664422 |
| NT01MG0519 | 9 | 0.98 | 20226507; 20552428; 19325113; 18234676; 16415354; 15890647; 12888572; 11013079; 12055623; 11870854 |
| NT01MK0307 | 8 | 0.77 | 20831589; 20601217; 20419722; 20186410; 19931102; 19688823; 19665595; 18463136; 15583382; 18433620 |
| NT01MK0441 | 6 | 0.77 | 20164056; 19173707; 10393331; 17588178; 10706821; 11839498; 11500378; 12379279; 8477448; 11707072 |
| NT01MK0827 | 6 | 0.93 | 21326897; 21211722; 21118997; 20534568; 20505338; 20374520; 20232936; 1846780; 20083442; 9230309 |
| NT01MK0979 | 18 | 0.99 | 21347707; 21179061; 21103969; 20935164; 20858838; 20808807; 20709029; 20597505; 20595678; 20594231 |
| NT01MK0991 | 6 | 0.79 | 21067376; 20541396; 20482652; 20488783; 20167366; 20150365; 19796694; 19631206; 19548140; 21261927 |
| NT01MK1652 | 19 | 0.83 | 11596651; 15913610; 12200473; 10786796; 8692962; 9461254; 8585318; 8050708; 7856404; 2620065 |
| NT01ML0007 | 6 | 0.97 | 21254166; 21229879; 21098118; 21079245; 21074444; 21063406; 21062978; 21056033; 21030584; 20968093 |
| NT01ML0240 | 12 | 0.79 | 19753541; 19031360; 16785239; 14510716; 10852914; 9660831; 2851488; 9261179; 9049355; 7979996 |
| NT01ML0296 | 6 | 0.92 | 17493798; 15367703; 11023544; 8805338; 7692268; 2696875; 3047400; 3748047; 6533049 |
| NT01ML0496 | 9 | 0.98 | 20145252; 20132453; 19456874; 15169766; 19072541; 11260139; 1851153; 18204094; 6316353; 6427211 |
| NT01ML0941 | 6 | 0.78 | 2153220; 11214325; 17921483; 17674145; 16984922; 2041477; 15322083; 11532933; 3442830; 14506243 |
| NT01ML1231 | 8 | 0.72 | 21400585; 21400553; 21400515; 21400237; 21399654; 21399615; 21398595; 21398490; 21398445; 21398434 |
| NT01ML1346 | 12 | 0.71 | 21307369; 21265898; 21060254; 20124512; 18827014; 17922265; 17356065; 17118947; 16771841; 15297378 |
| NT01ML1548 | 11 | 0.99 | 16181782; 17078817; 10722135; 10986230; 10556026; 9393713; 2548993; 10760133; 11958563; 11907683 |
| NT01ML1948 | 8 | 0.87 | 21389620; 21385584; 21382272; 21380518; 21377525; 21375472; 21372134; 21368419; 21365006; 21364950 |
| NT01ML2024 | 18 | 0.96 | 19960428; 18573480; 10473554; 16789882; 14706849; 14583680; 9630701 |
| NT01ML2064 | 15 | 0.90 | 20144684; 10629763; 18649864; 8402178; 1528892; 1633609; 1828858; 2449695; 1669444; 2270287 |
| NT01ML2195 | 8 | 0.73 | 21357486; 21322495; 21322489; 21319715; 21299212; 21277857; 21277289; 21220625; 21216999; 21209222 |
| NT01ML2200 | 1 | 0.88 | 19249065; 942051; 17937657; 11495997; 14705949; 12596860; 11390676; 10769117; 9817848; 8170390 |
| NT01ML2336 | 8 | 0.81 | 18056994; 19787709; 19661995; 19434406; 10859321; 19056199; 18850694; 16500709; 18473959; 18363338 |
| NT01ML2415 | 8 | 0.88 | 16344854; 16179381; 14672739; 12646198; 11469812; 11306067; 8550838; 1748675; 10084958; 9188497 |
| NT01ML2427 | 8 | 0.92 | 21272324; 20392690; 15150239; 19684063; 19019148; 18673073; 17305364; 17108290; 15470119; 15013751 |
| NT01ML3033 | 8 | 0.73 | 21178285; 20811135; 19966484; 19546514; 19424620; 19200041; 17291445; 16402358; 16221991; 15996793 |
| NT01ML3055 | 8 | 0.83 | 20433942; 16267304; 17849411; 17090916; 16318918; 12686116; 12480887; 7783618; 13345830; 9325289 |
| NT01ML4242 | 19 | 0.83 | 20684602; 20447077; 20428816; 20331643; 20178374; 19896717; 19541294; 18953688; 9670976; 18498310 |
| NT01ML4550 | 18 | 0.93 | 21220721; 21159965; 21139282; 21107313; 21103975; 21081491; 21075931; 21060855; 21044569; 21036669 |
| NT01ML4569 | 6 | 0.93 | 21187477; 21127267; 21030352; 20974932; 20969882; 20964789; 20963646; 20955519; 20952393; 20929586 |
| NT01ML4958 | 12 | 0.80 | 21373826; 21356360; 21238917; 21235061; 21216416; 21209184; 21205201; 21191678; 21187371; 21184302 |
| NT01ML5069 | 4 | 0.72 | 18710261; 16954321; 18032424; 16369542; 11943863; 16390137; 8089067; 12773148; 12161746; 11590162 |
| NT01MM0060 | 18 | 0.92 | 17933550; 16135777; 12073031; 11891667; 10517864; 9473333; 8480190 |
| NT01MM0965 | 12 | 0.85 | 21338465; 21256137; 21273475; 21138942; 21115803; 21085989; 21029479; 20957186; 20944837; 20890268 |
| NT01MM1219 | 19 | 0.92 | 15486203; 18045411; 17503147; 17031028; 17003269; 15886040; 15821912; 11549021; 12883866; 12646689 |
| NT01MM1585 | 19 | 0.87 | 21394312; 21383503; 21366909; 21354605; 21347376; 21342283; 21327212; 21286806; 21273509; 21267505 |
| NT01MM1697 | 6 | 0.71 | 16296883; 16042414; 15978576; 15554704; 12729751; 9826182; 3118156; 8100262; 8422927; 1652772 |
| NT01MM1993 | 18 | 0.99 | 15149971; 14507379; 12163501; 11160807; 10931328; 1828528; 2252588; 9813059; 7108955; 8282685 |
| NT01MM2367 | 19 | 0.87 | 21330522; 21175888; 21097635; 21087652; 21070413; 21070404; 21040511; 20980680; 20843785; 20724636 |
| NT01MM2761 | 19 | 0.93 | 21058504; 21038111; 20798166; 20718293; 20714719; 20703955; 20666458; 20639136; 20591661; 20576315 |
| NT01MM3601 | 12 | 0.76 | 20846863; 20824169; 20819954; 20670659; 19692335; 19402045; 19356619; 19301871; 19241474; 19075822 |
| NT01MM4323 | 18 | 0.82 | 21134399; 20805831; 20554699; 20430082; 20206165; 19915555; 19822200; 15113752; 19082842; 15101088 |
| NT01MP0084 | 8 | 0.84 | 20597606; 17108241; 19379783; 18455501; 17609257; 16469539; 16453288; 16377227; 16352674; 15988697 |
| NT01MP0330 | 8 | 0.73 | 21178285; 20811135; 19966484; 19546514; 19424620; 19200041; 17291445; 16402358; 16221991; 15996793 |
| NT01MP0379 | 8 | 0.71 | 20519396; 20304492; 19164483; 16729314; 18696218; 18445831; 9751768; 18164766; 18162076; 17207117 |
| NT01MP0440 | 8 | 0.92 | 21253866; 21036145; 10672375; 20160912; 20022530; 9324032; 17667915; 17635929; 8798399; 16293764 |
| NT01MP0611 | 6 | 0.91 | 17184904; 9349725; 7628707; 7867950; 7748939; 7748938; 8515442; 1825651; 1879696; 2265752 |
| NT01MP0655 | 12 | 0.95 | 20420854; 20350528; 19552712; 19288213; 19118453; 10829079; 18205165; 10829079; 111762; 16529946 |
| NT01MS0087 | 8 | 0.73 | 21178285; 20811135; 19966484; 19546514; 19424620; 19200041; 17291445; 16402358; 16221991; 15996793 |
| NT01MS0245 | 8 | 0.73 | 21178285; 20811135; 19966484; 19546514; 19424620; 19200041; 17291445; 16402358; 16221991; 15996793 |
| NT01MS0292 | 8 | 0.90 | 21296048; 21287605; 21265782; 21192786; 21109633; 21164002; 20675088; 20669924; 20629638; 20622453 |
| NT01MS0299 | 13 | 0.95 | 3902358; 8509334; 8389364; 1400344; 2671655; 2828880; 6991870 |
| NT01MS0461 | 18 | 0.84 | 21125597; 21098514; 20824416; 12740933; 20599691; 20583998; 20581201; 20510648; 20211899; 20160052 |
| NT01MS0476 | 13 | 0.74 | 7677746; 16959352; 15378702; 11491085; 12533145; 10524762; 9868784; 1856227; 8372100; 1856227 |
| NT01MS0492 | 6 | 0.96 | 21206470; 21187148; 21145792; 20558179; 20497334; 20465561; 20386912; 20297719; 20138014; 9363943 |
| NT01MS0593 | 6 | 0.94 | 21368455; 21360615; 21359151; 21342502; 21330432; 21297161; 21284673; 21267641; 21264493; 21245388 |
| NT01MS0673 | 14 | 0.94 | 21385632; 20936320; 20711856; 20575762; 17341192; 20053586; 19923730; 19878436; 19843229; 19607848 |
| NT01MS0898 | 18 | 0.82 | 21072340; 19603679; 19602147; 18953667; 15057514; 18415938; 14617043; 10498699; 17326661; 7838735 |
| NT01MS0912 | 19 | 0.88 | 18761435; 11418550; 17910958; 16475802; 15586827; 15366937; 11790124; 11775545; 11084598; 10684641 |
| NT01MS0938 | 17 | 0.90 | 20700605; 20482591; 20211899; 10594833; 18426891; 19168258; 17542919; 17040898; 18191205; 10551881 |
| NT01MS0981 | 8 | 0.79 | 21029451; 20851693; 20696493; 20587739; 12615923; 19586916; 19389383; 15952888; 19348026; 17630784 |
| NT01MS1042 | 1 | 0.95 | 19731276; 17874165; 2985470; 12207706; 10579528; 10564784; 10356262; 9851916; 9846749; 9011040 |
| NT01MS1088 | 18 | 0.81 | 21389771; 21346101; 21139810; 21086908; 20971155; 20865166; 20806826; 20502413; 20388533; 20017440 |
| NT01MS1098 | 18 | 0.81 | 21388950; 21382336; 21372504; 21371592; 21362064; 21360618; 21352502; 21351734; 21322593; 21322544 |
| NT01MS1111 | 8 | 0.77 | 21373748; 21364653; 21359673; 21333634; 21328570; 21315832; 21308999; 21285290; 21283809; 21272662 |
| NT01MS1204 | 12 | 0.93 | 11163206; 18173131; 15182186; 16002085; 15720562; 15606774; 15560777; 14991425; 8460142; 10411757 |
| NT01MS1350 | 8 | 0.72 | 21236318; 20821193; 20675489; 19452213; 18706942; 18675788; 18667562; 18594130; 17938909; 17890844 |
| NT01MS1397 | 8 | 0.76 | 20601473; 20525825; 18190263; 10234841; 17318639; 16899629; 16463078; 16292529; 16135233; 15090508 |
| NT01MS1399 | 8 | 0.73 | 21357486; 21322495; 21322489; 21319715; 21299212; 21277857; 21277289; 21220625; 21216999; 21209222 |
| NT01MS1508 | 19 | 0.92 | 19577637; 17113996; 11260463; 10986272; 15727837; 11929520; 10678915; 10398745; 8595593; 1655711 |
| NT01MS1603 | 13 | 0.97 | 17537210; 12114023; 12236604; 18186482; 18052890; 1766869; 16053518; 2405162; 17010380; 16208181 |
| NT01MS1633 | 12 | 0.75 | 20713651; 20599668; 19853572; 1619456; 18387365; 17556688; 17269451; 16834335; 16573693; 16389450 |
| NT01MS1676 | 19 | 0.84 | 19052362; 10698988; 15948948; 15347760; 14188160; 8662022; 8125347; 1993184; 2841972 |
| NT01MS1731 | 8 | 0.73 | 21178285; 20811135; 19966484; 19546514; 19424620; 19200041; 17291445; 16402358; 16221991; 15996793 |
| NT01MS1793 | 19 | 0.73 | 21274977; 20095006; 19737507; 18972347; 18224673; 772168; 16278308; 15837433; 15709142; 15117398 |
| NT01MS1882 | 18 | 0.81 | 21362453; 21347244; 21339605; 21324980; 21322614; 21300787; 21297961; 21280048; 21277866; 21276808 |
| NT01MS1921 | 19 | 0.73 | 21372094; 21367878; 21330526; 21311881; 21302624; 21270457; 21237173; 21234787; 21206029; 21205968 |
| NT01MS2188 | 15 | 0.83 | 21364902; 21257775; 21239559; 20487301; 16689796; 2118274; 18792684; 11741852; 18469109; 17478425 |
| NT01MS2213 | 3 | 0.90 | 21349988; 21327475; 20467248; 20431909; 20229202; 20208444; 20133413; 20067320; 19884777; 19808679 |
| NT01MS2245 | 4 | 0.76 | 15357215; 10348751; 11770120; 7840574; 11044368; 11027804; 10873522; 10589984; 6572966; 2985470 |
| NT01MT0145 | 8 | 0.79 | 18544072; 15516593; 11959447; 10422581; 9748275; 8572689; 9297469; 3170343; 2178138; 2009287 |
| NT01MT0358 | 19 | 0.87 | 20602334; 20532300; 20331963; 19912589; 19217283; 19214747; 18563288; 3053713; 18393820; 18387370 |
| NT01MT0722 | 1 | 0.72 | 19351325; 19334594; 17942358; 18498255; 794063; 10445884; 9665716 |
| NT01MT0809 | 8 | 0.85 | 21378306; 21278127; 21130735; 21117647; 20920593; 20717703; 20705057; 20457604; 20435060; 20236937 |
| NT01MT0903 | 6 | 0.87 | 20977236; 20961081; 20828134; 20705454; 20696762; 20657578; 20614917; 20600091; 20532362; 20444223 |
| NT01MT1129 | 8 | 0.72 | 21266589; 21090696; 21033580; 20934393; 20877805; 20857468; 20835839; 20735712; 20707311; 20597483 |
| NT01MT1226 | 19 | 0.94 | 21054166; 20868765; 20666458; 20656870; 20469649; 20382819; 20223653; 20201406; 19948399; 19596709 |
| NT01MT1401 | 8 | 0.78 | 20661960; 19579240; 19115296; 18215050; 17927915; 2834341; 3276516; 9047371; 16302255; 15959893 |
| NT01MT1568 | 15 | 0.97 | 21070938; 21070950; 20875081; 20025661; 17639608; 19236354; 18415079; 9037050; 17456186; 15590692 |
| NT01MT1860 | 18 | 0.90 | 18692508; 18156179; 12923181; 12354616; 11756453; 11726714; 11248195; 10521259; 8045426; 9693722 |
| NT01NA0096 | 8 | 0.76 | 21374779; 21064170; 20526519; 20396613; 19943268; 19891489; 19664062; 19016851; 20396603; 18847184 |
| NT01NA0751 | 19 | 0.91 | 21193672; 21173313; 20871101; 20600864; 20030726; 19682076; 19473944; 19246750; 18753038; 5323018 |
| NT01NA2416 | 19 | 0.91 | 19793137; 17277884; 14761997; 1474649; 15888348; 3413113; 10347049; 11098143; 10964959; 7844147 |
| NT01NA3012 | 8 | 0.75 | 21404308; 21404260; 21404018; 21403916; 21403907; 21403660; 21403421; 21403409; 21402901; 21402494 |
| NT01NE0059 | 8 | 0.71 | 21294156; 21080034; 21042030; 20406700; 20363943; 20148428; 20138225; 20026236; 19968635; 19160513 |
| NT01NE1204 | 4 | 0.80 | 21300638; 20540056; 20445252; 19921332; 12864863; 15572765; 11046155; 17168829; 17110979; 16403649 |
| NT01NE1292 | 12 | 0.79 | 20103594; 18617647; 17690102; 17109403; 17088474; 16893192; 13130121; 11930615; 10712407; 10388479 |
| NT01NE1368 | 12 | 0.93 | 21042721; 20667819; 20630859; 20363282; 20206694; 20069636; 19995574; 19717562; 19616577; 19497371 |
| NT01NE1658 | 4 | 0.75 | 21343356; 21216994; 20831592; 20828376; 11752303; 20030726; 20019083; 10856226; 19302523; 9597130 |
| NT01NE2126 | 8 | 0.75 | 18097650; 15316720; 11889481; 10705449; 9425311 |
| NT01NE2277 | 4 | 0.88 | 21339296; 20624225; 20585119; 20233934; 20036749; 19843215; 19711106; 19710230; 19582378; 12927971 |
| NT01NFA0131 | 6 | 0.82 | 21078962; 19840190; 18786551; 18419580; 18234224; 17895243; 16887145; 10652347; 16425234; 16176273 |
| NT01NFA0344 | 12 | 0.71 | 20167799; 20014030; 19889088; 19843523; 19767395; 19635595; 16381856; 19161851; 16780827; 19016865 |
| NT01NFA0406 | 8 | 0.73 | 20629071; 20093189; 18485344; 19332830; 18416536; 18343668; 37837; 11591691; 7544094; 16611136 |
| NT01NFA0693 | 12 | 0.71 | 21209222; 21102654; 21087465; 20886100; 20807235; 20806779; 20594967; 20570161; 20423461; 20207139 |
| NT01NFA0868 | 12 | 0.98 | 21243083; 21080956; 21075844; 21045264; 20952758; 20938207; 20874501; 20800088; 20798590; 20723242 |
| NT01NFA1036 | 12 | 0.84 | 21349274; 21309515; 21287358; 21187650; 21159160; 21130728; 20973643; 20888865; 20879038; 20816878 |
| NT01NFA1387 | 9 | 0.99 | 21034488; 18171025; 10464321; 1504080; 9684854; 9452501; 9403065; 942051 |
| NT01NFA1520 | 19 | 0.78 | 21369910; 21311881; 21256135; 20920201; 20714719; 20703955; 20686913; 20571959; 20562284; 20383007 |
| NT01NFA1540 | 8 | 0.82 | 21059110; 21044985; 20861021; 20826812; 20673055; 20629071; 20444427; 2050109; 20399281; 20378991 |
| NT01NFA1826 | 19 | 0.95 | 21278296; 21161258; 3611062; 20045693; 19637158; 19245333; 19143835; 18341480; 18184612; 17892446 |
| NT01NFA2004 | 8 | 0.89 | 21291916; 20549192; 5420057; 11290745; 19996487; 19935882; 19854152; 19781588; 2401567; 18980183 |
| NT01NFA2088 | 8 | 0.79 | 21317335; 21216959; 21171573; 21039937; 20601653; 20302299; 20218714; 20162368; 20147623; 19932076 |
| NT01NFA2220 | 8 | 0.93 | 21396919; 21396482; 21387033; 21381206; 21380499; 21373268; 21364005; 21360535; 21359855; 21353684 |
| NT01NFA2737 | 8 | 0.85 | 18510336; 7778974; 15242297; 14630048; 11114944; 2971647; 11165359; 8245826; 10744688; 8254303 |
| NT01NFA2805 | 8 | 0.85 | 20603809; 19364480; 18614015; 9870699; 12176061; 11841569; 11313346; 7476123; 7715455; 2808350 |
| NT01NFA2808 | 8 | 0.88 | 20959113; 1482115; 19006332; 12368463; 18355283; 17690995; 16844076; 15922336; 15683249; 15352239 |
| NT01NFA2835 | 8 | 0.73 | 21178285; 20811135; 19966484; 19546514; 19424620; 19200041; 17291445; 16402358; 16221991; 15996793 |
| NT01NFA2988 | 18 | 0.86 | 20181730; 19962375; 18799268; 11246165; 16891059; 15804497; 12576674; 1377899; 10082872; 9368839 |
| NT01NFA3860 | 9 | 0.98 | 19405909; 18247525; 16707707; 18576672; 18542924; 18338382; 16386430; 18260643; 17613526; 17524985 |
| NT01NFA3920 | 12 | 0.77 | 11292810; 9757107; 17340635; 17123102; 17112654; 17068342; 11076021; 16513119; 16246027; 16202924 |
| NT01NFA3932 | 8 | 0.82 | 21377632; 21364005; 21362018; 21349860; 21347589; 21345790; 21343423; 21329681; 21325261; 21324174 |
| NT01NFA3943 | 8 | 0.82 | 20171216; 18765233; 15466509; 9105615; 10075418; 7934817; 10647818; 8206829; 2061286 |
| NT01NFA4308 | 19 | 0.95 | 14479179; 19052362; 10698988; 15948948; 15347760; 15302885; 14188160; 8662022; 8807826; 8125347 |
| NT01NFA4349 | 8 | 0.85 | 20675490; 20017731; 16607529; 37837; 12603323; 11432745; 1505666 |
| NT01NFA4389 | 19 | 0.93 | 21193820; 21126522; 20615998; 20079869; 19531602; 19412576; 19245333; 18679678; 18421587; 18379842 |
| NT01NFA4543 | 19 | 0.78 | 21350040; 20617340; 18292091; 20025609; 19574437; 19826227; 18366639; 19037899; 18713381; 18704491 |
| NT01NFA4565 | 14 | 0.89 | 21350654; 20880495; 20810283; 20668078; 20338830; 20440001; 20196672; 20105439; 14651859; 19902562 |
| NT01NFA4730 | 8 | 0.82 | 19840846; 18941994; 10884373; 10785638; 18277387; 15753130; 1174548; 15281945; 14871203; 8335622 |
| NT01NFA4814 | 8 | 0.88 | 20625734; 20406281; 20093189; 19781588; 18391450; 17237047; 16814740; 16522631; 15449607; 12634329 |
| NT01NFA4830 | 8 | 0.92 | 21077373; 21042417; 20705129; 20127467; 19777264; 19545523; 19523599; 19520672; 19405028; 16524901 |
| NT01NFA4831 | 8 | 0.86 | 12191769; 12482588; 10425677; 10216163; 9278503; 1333793 |
| NT01NFA4852 | 12 | 0.98 | 21404362; 21403131; 21397065; 21395067; 21394739; 21394468; 21392058; 21388873; 21388559; 21388136 |
| NT01NFA4947 | 1 | 0.96 | 20210945; 20061483; 19383689; 18451046; 18050920; 17981822; 10595554; 9531508; 15647275; 15576367 |
| NT01NFA5311 | 4 | 0.82 | 21388800; 21384874; 21382703; 21369715; 21347497; 21339256; 21337715; 21320452; 21288457; 21268091 |
| NT01NFA5346 | 8 | 0.81 | 7935603; 12573288; 12091098; 12036956; 7810866 |
| NT01NFA5735 | 8 | 0.94 | 17891922; 16762453; 10581550; 15604729; 15164997; 15056475; 14555654; 12875742; 12619155; 11358527 |
| NT01NFA5751 | 8 | 0.80 | 21336929; 20026072; 16499623; 16023116; 12186751; 11004571; 9443811; 9417993; 8786138; 7584606 |
| NT01NFB0048 | 4 | 0.88 | 21339296; 20624225; 20585119; 20233934; 20036749; 19843215; 19711106; 19710230; 19582378; 12927971 |
| NT01NH0571 | 19 | 0.72 | 21310261; 20715794; 20385411; 20149108; 19834746; 19827773; 19760485; 19735113; 19687346; 19679115 |
| NT01NH2774 | 13 | 0.98 | 21134380; 20920668; 20823541; 20729861; 20699648; 11342140; 20670890; 1735721; 20580719; 20348443 |
| NT01NH2931 | 8 | 0.77 | 21394309; 21393477; 21393229; 21391839; 21389104; 21385862; 21384912; 21384143; 21380725; 21376747 |
| NT01NH3192 | 12 | 0.96 | 17028008; 10491199; 12836705; 12651114; 11517925 |
| NT01NH4272 | 18 | 0.87 | 20111865; 19713238; 12604548; 10943556; 16101295; 10585202; 10543738; 1317381; 10348749; 533264 |
| NT01NM0513 | 12 | 0.98 | 21327044; 21123869; 20959450; 20876094; 20735484; 20716448; 20696826; 20688909; 20655928; 20630471 |
| NT01NM1575 | 18 | 0.95 | 21258844; 20363758; 19576997; 19130269; 18702072; 18048940; 17975309; 10419525; 15161914; 15981243 |
| NT01NM2482 | 15 | 0.75 | 21050451; 20460525; 20345481; 20129639; 19962964; 19818776; 17591613; 19541854; 19170204; 19140155 |
| NT01NO0504 | 8 | 0.73 | 21178285; 20811135; 19966484; 19546514; 19424620; 19200041; 17291445; 16402358; 16221991; 15996793 |
| NT01NO0523 | 8 | 0.70 | 21382104; 21166718; 20941387; 20060836; 19906178; 19822367; 17526500; 19478453; 18997103; 18562317 |
| NT01NO0738 | 12 | 0.78 | 21397717; 20950422; 17560276; 20643949; 12823820; 2076701; 20177791; 20055936; 20096393; 19879896 |
| NT01NO0838 | 18 | 0.73 | 10688190; 15289558; 15174953; 10658645; 1447130; 7565096 |
| NT01NO0945 | 12 | 0.75 | 19809370; 18570439; 17005457; 11551224; 16084398; 6425056 |
| NT01NO1330 | 8 | 0.73 | 21178285; 20811135; 19966484; 19546514; 19424620; 19200041; 17291445; 16402358; 16221991; 15996793 |
| NT01NO1340 | 9 | 0.79 | 21076105; 19138528; 19093250; 19127309; 14584854; 12887206; 12828209; 12512582; 1318499; 11128388 |
| NT01NO1393 | 14 | 0.75 | 21205085; 16778368; 19775280; 11895911; 16491070; 18255096; 17602053; 17416930; 16708051; 16642528 |
| NT01NO1570 | 8 | 0.73 | 21178285; 20811135; 19966484; 19546514; 19424620; 19200041; 17291445; 16402358; 16221991; 15996793 |
| NT01NO2003 | 5 | 0.79 | 21089447; 19995333; 19551860; 17936463; 17519237; 17308787; 10749872; 10681374; 7598806; 7954741 |
| NT01NO2054 | 12 | 0.79 | 21397698; 21382888; 21376094; 21375592; 21357482; 21350490; 21349329; 21347706; 21339300; 21337514 |
| NT01NO2524 | 8 | 0.73 | 21178285; 20811135; 19966484; 19546514; 19424620; 19200041; 17291445; 16402358; 16221991; 15996793 |
| NT01NP0303 | 12 | 0.74 | 20573955; 16565055; 17406022; 16232997; 12860414; 12657051; 11943164; 11580265; 11484476; 11162559 |
| NT01NP0550 | 8 | 0.85 | 9325428; 9151968; 1314088; 7649177; 7851382; 8319675; 8481089; 8215796; 1953299; 1772346 |
| NT01NP0578 | 2 | 0.81 | 21255303; 21173279; 17448684; 16835730; 11215515; 12196147; 9056975 |
| NT01NP1187 | 8 | 0.84 | 21042417; 20943545; 20823222; 20808932; 20620150; 15987803; 20603809; 20589618; 20406883; 19038229 |
| NT01NP1738 | 18 | 0.79 | 21354532; 21256461; 21197505; 21152117; 21094940; 21044960; 20966395; 20949136; 20926757; 20889969 |
| NT01NP1775 | 9 | 0.99 | 20123779; 12378192; 11032827; 8952487; 1453953 |
| NT01NP2072 | 8 | 0.92 | 20956531; 20449117; 20015072; 19894084; 10700270; 19397993; 19027887; 18755150; 18248595; 17906130 |
| NT01NP2110 | 12 | 0.73 | 21392374; 21382340; 21093452; 20935102; 20598080; 20472801; 20226724; 20162616; 15031730; 11932238 |
| NT01NP2179 | 9 | 0.87 | 21392546; 21387444; 21378393; 21377632; 21359855; 21354840; 21354350; 21349883; 21347589; 21347544 |
| NT01NP2291 | 9 | 0.87 | 19766195; 15209520; 17707131; 16819514; 16804052; 12441112; 15604682; 15044020; 11790793; 10801831 |
| NT01NP2590 | 12 | 0.70 | 20457869; 20400202; 19143597; 18850059; 16000332; 14636577; 15279777; 15175154; 14635057; 12470813 |
| NT01NP2633 | 17 | 0.83 | 21126315; 20944626; 20506188; 15037068; 18411269; 17936712; 11390668; 15598798; 18374519; 18325588 |
| NT01NP2669 | 8 | 0.84 | 20675490; 20362274; 7601336; 19118348; 19110078; 15912375; 18973815; 18959761; 18339331; 17244482 |
| NT01NS0548 | 15 | 0.92 | 17434492; 17004655; 16514156; 16102006; 15073296; 11254610; 879805; 10594824; 10524254; 9802029 |
| NT01NS1830 | 11 | 0.88 | 17765537; 11040422; 16304164; 7813017; 2231712; 9274023 |
| NT01NS2847 | 4 | 0.79 | 11759840; 11913779; 9927482; 8200522; 366607; 7683649; 11607099; 1282192; 1404376; 1956294 |
| NT01NS2887 | 19 | 0.84 | 21205103; 19288077; 14999614; 18195661; 8459035; 16687599; 4878433; 1834913; 11421283; 11065358 |
| NT01NS3033 | 19 | 0.90 | 20920201; 20672277; 20636376; 3611062; 20383007; 20199575; 20124190; 19279143; 19720067; 15109786 |
| NT01NS3686 | 14 | 0.92 | 20445243; 18020427; 1435732; 17146584; 16618122; 15138730; 14981288; 9139917; 11730338; 10927027 |
| NT01NS4692 | 15 | 0.79 | 18930705; 17611224; 17294361; 17267442; 16979619; 10473124; 15790965; 10473124; 14633699; 14597169 |
| NT01NS4871 | 2 | 0.85 | 18311927; 12410317; 14592524; 9188462; 1097404 |
| NT01NS5067 | 18 | 0.74 | 21397666; 21396132; 21395360; 21392569; 21392185; 21392049; 21389082; 21388953; 21388792; 21385666 |
| NT01NS5306 | 15 | 0.76 | 9295299; 8939900; 8543151; 8112331; 2160609; 2504497 |
| NT01NSA0176 | 6 | 0.95 | 17416902; 12748189; 1349601; 8107086; 6211591 |
| NT01NW0311 | 11 | 0.96 | 21079776; 20952568; 20875907; 20709901; 20546690; 20467813; 20363791; 20298189; 20211730; 20084428 |
| NT01NW0781 | 8 | 0.73 | 21178285; 20811135; 19966484; 19546514; 19424620; 19200041; 17291445; 16402358; 16221991; 15996793 |
| NT01NW0839 | 8 | 0.73 | 21178285; 20811135; 19966484; 19546514; 19424620; 19200041; 17291445; 16402358; 16221991; 15996793 |
| NT01NW0995 | 8 | 0.73 | 21178285; 20811135; 19966484; 19546514; 19424620; 19200041; 17291445; 16402358; 16221991; 15996793 |
| NT01NW2028 | 18 | 0.86 | 21397193; 21370995; 21339618; 21339577; 21338570; 21317284; 21315086; 21304597; 21215441; 21195087 |
| NT01NW2305 | 6 | 0.86 | 21389614; 21388271; 21385879; 21383078; 21381961; 21376234; 21368822; 21367893; 21366595; 21365649 |
| NT01OA0251 | 6 | 0.73 | 21376705; 21348850; 21270603; 21245378; 21148321; 21132535; 21118960; 21097474; 21070416; 21045111 |
| NT01OA0349 | 8 | 0.93 | 21278273; 20695438; 20570675; 20442401; 20086057; 19056333; 18683078; 18351376; 17854388; 4062906 |
| NT01OA0409 | 8 | 0.80 | 21236367; 20941747; 20865669; 20842623; 20106972; 20071332; 20036864; 15099738; 11830638; 19126643 |
| NT01OA0584 | 9 | 0.74 | 21398484; 21393229; 21377632; 21336932; 21301088; 21280175; 21185310; 21094257; 21045289; 20961859 |
| NT01OA0802 | 9 | 0.79 | 21393229; 21377632; 21301088; 21212097; 21199673; 21166831; 21094257; 21045289; 21035728 |
| NT01OA1665 | 12 | 0.90 | 19887527; 19836138; 17559250; 15264254; 17257322; 17010374; 16425288; 15892698; 15304217; 11521196 |
| NT01OA1809 | 12 | 0.99 | 21368222; 21323990; 21303407; 21251800; 21221694; 21167902; 21042721; 14510993; 20703245; 20676769 |
| NT01OA2661 | 8 | 0.71 | 20979355; 20870790; 19496621; 16156794; 15175326; 15035646; 11728457; 10491142; 8536688; 1605643 |
| NT01OA2664 | 8 | 0.79 | 20979355; 19815558; 16608357; 15943805; 15520003; 15102833; 9718301; 8797851 |
| NT01OA2665 | 8 | 0.81 | 21339825; 20979355; 20870790; 20816962; 20610779; 20558724; 18651753; 20117074; 19815558; 19496622 |
| NT01OA2666 | 8 | 0.76 | 20979355; 20816962; 19815558; 19496622; 15972314; 18454933; 19118365; 16608357; 10649489; 17200125 |
| NT01OA2674 | 8 | 0.78 | 21339825; 20979355; 20870790; 18651753; 19815558; 19496621; 15972314; 17894548; 10649489; 16973619 |
| NT01OAA0546 | 12 | 0.73 | 21081498; 20615351; 20538584; 20437165; 20419412; 20064164; 19754117; 19631648; 19555457; 12634052 |
| NT01OAA1125 | 8 | 0.92 | 20609914; 19549597; 18991392; 18981569; 18930846; 18211101; 12936980; 17385893; 2172928; 5700707 |
| NT01OAA1731 | 8 | 0.92 | 20795494; 16872401; 15316720; 12427946; 10830505; 1310545; 10548510; 9480821; 9022686 |
| NT01OI0562 | 12 | 0.93 | 21404362; 21404269; 21404243; 21404111; 21404103; 21403096; 21403095; 21402841; 21402360; 21401523 |
| NT01OI2292 | 4 | 0.82 | 17838811; 19332816; 10493868; 18042185; 10464206; 17436617; 11737637; 6801017; 4995746; 7926687 |
| NT01OI3286 | 4 | 0.83 | 19082958; 10735848; 18363793; 11418552; 10376844; 15699208; 11823460 |
| NT01OI3306 | 2 | 0.98 | 21182990; 19931406; 19345718; 18071260; 16769720; 16557306; 16353092; 11322938; 11318637; 10642176 |
| NT01OT0047 | 14 | 0.76 | 10747855; 2556396; 10995231; 10375643; 6160384; 8730877; 184817 |
| NT01OT0157 | 12 | 0.77 | 20736484; 20663878; 20106980; 18774967; 9108479; 19394289; 10811910; 9482716; 16580629; 18327258 |
| NT01OT0252 | 18 | 0.91 | 19469713; 17592720; 12960168; 10950931; 10748025 |
| NT01OT0566 | 18 | 0.97 | 21149736; 21059948; 21059651; 20952391; 20937813; 20935152; 20876358; 20847183; 20719938; 20715055 |
| NT01OY0207 | 13 | 0.98 | 19749382; 18320344; 10704215; 12730326; 17150597; 15978085; 15967466; 15596445; 15571069; 15564671 |
| NT01OY0749 | 19 | 0.75 | 21359518; 21112711; 21107761; 20659950; 20484424; 20445711; 20430261; 20369497; 20027119; 20013275 |
| NT01OY0925 | 6 | 0.78 | 21264264; 21245389; 21227759; 21187148; 21087930; 21044871; 21036739; 20972219; 20953064; 20814424 |
| NT01OY1009 | 13 | 0.94 | 21098258; 21149735; 20942128; 20387531; 20018267; 19761773; 19702327; 19502729; 16754626; 19398261 |
| NT01PA0036 | 18 | 0.83 | 21130773; 12930999; 18692508; 18156179; 241020; 12923181; 12354616; 11756453; 11726714; 11248195 |
| NT01PA0069 | 13 | 0.84 | 19720141; 18948503; 15497447; 15053875; 11134326; 11565036; 9182768; 4745379; 10591218; 1531632 |
| NT01PA0070 | 13 | 0.81 | 11939770; 9757106; 3905389; 7916644; 6329735 |
| NT01PA0916 | 14 | 0.73 | 21398490; 21299636; 19523919; 10400604; 8995388; 15599027; 8631326; 12787358; 12062412; 11566360 |
| NT01PA1025 | 15 | 0.72 | 19835966; 15183876; 11162187; 4901868; 7845360; 8455563; 1452195; 1251186 |
| NT01PA1424 | 8 | 0.73 | 21167817; 20614517; 20570573; 20512454; 20451229; 17433650; 20081242; 20081240; 19921097; 19699269 |
| NT01PA1775 | 8 | 0.81 | 21305143; 18371296; 18216016; 12426385; 7721730; 10413356; 17559573; 16508746; 16233230; 15842998 |
| NT01PC0049 | 12 | 0.97 | 21385872; 21375706; 21220481; 21184521; 21122141; 20930480; 20852886; 20805188; 20602853; 20495942 |
| NT01PC0438 | 12 | 0.97 | 20398703; 20345279; 20224549; 20064164; 20010851; 19881308; 19863457; 19732346; 12045105; 19495967 |
| NT01PC0770 | 2 | 0.89 | 21305354; 21255426; 21142117; 20154124; 20038586; 20038499; 18630851; 19122276; 16535143; 18546893 |
| NT01PC0961 | 18 | 0.89 | 20647000; 19663511; 18535817; 17225063; 17177252; 11418569; 10446249; 9545377; 11087740; 2139651 |
| NT01PC2318 | 8 | 0.70 | 21206034; 20423462; 17847094; 1417845; 9310340; 18984032; 15277752; 11388460; 11223945; 10537158 |
| NT01PC2918 | 8 | 0.80 | 3982017; 19299076; 19119913; 17250685; 16637010; 16120446; 15581577; 11146102; 11030559; 1316154 |
| NT01PC3069 | 18 | 0.72 | 21315593; 21044960; 20730379; 21080532; 20457836; 20338165; 20204726; 20127491; 19907129; 19624522 |
| NT01PC3326 | 12 | 0.76 | 21343356; 21130728; 21081693; 21078397; 20886100; 20858537; 20600029; 20491066; 11672528; 19786100 |
| NT01PE0506 | 8 | 0.78 | 21334970; 21165740; 21150112; 20692227; 20618134; 20215780; 20096472; 19950920; 19711069; 21255283 |
| NT01PE1492 | 12 | 0.95 | 20802071; 20221715; 19558324; 12568721; 18590838; 18331590; 18187322; 18051297; 17668255; 17355285 |
| NT01PE2431 | 14 | 0.90 | 17384901; 9332345; 7525561; 7000783; 3265398; 2866187 |
| NT01PE4829 | 1 | 0.96 | 20817725; 2248769; 19193709; 3301814; 17937657; 10716626; 10471790; 11980910; 11751050; 11478886 |
| NT01PE5286 | 19 | 0.91 | 21367879; 21349845; 21221632; 21111225; 21054875; 20941621; 20889584; 20837469; 20829644; 20811656 |
| NT01PF0808 | 8 | 0.82 | 20723231; 19238379; 17428434; 17385904; 16752898; 16611136; 15003265; 12719939; 11583992; 9145444 |
| NT01PF0969 | 2 | 0.80 | 11180061; 19559030; 18693754; 16269131; 12193620; 10424174; 14745175; 10449533; 8479900; 8050991 |
| NT01PF1385 | 19 | 0.83 | 21397215; 21393864; 21393839; 21390509; 21385872; 21367878; 21360139; 21359176; 21346408; 21327387 |
| NT01PF2208 | 6 | 0.71 | 21148310; 21047968; 20949077; 20883816; 20877309; 20824083; 20670893; 20515430; 20417305; 19834905 |
| NT01PF2209 | 6 | 0.73 | 20877309; 19812063; 17976647; 10449415; 7138600; 16428324; 16412704; 10064605; 11239458; 15450732 |
| NT01PH0020 | 12 | 0.76 | 19908377; 18976628; 18846552; 18313393; 18154740; 17151018; 17092293; 15808850; 15305922; 12796498 |
| NT01PH0092 | 14 | 0.89 | 19362594; 16882332; 14674717; 10841757; 10830490; 10816600; 10433693; 9742728; 7721718; 9088991 |
| NT01PH0302 | 12 | 0.97 | 17488738; 15699195; 9809072; 11210516; 10701998; 9415440; 8702780 |
| NT01PH0304 | 8 | 0.81 | 21263039; 21081500; 21053013; 20725133; 20693323; 10978548; 20410231; 20002867; 19882159; 19360009 |
| NT01PH0338 | 19 | 0.83 | 10567375; 7827405; 2819874; 7588788; 7772861; 8179616; 1330104; 1336634; 1656876; 1700792 |
| NT01PH0477 | 8 | 0.81 | 21305143; 18371296; 18216016; 12426385; 7721730; 10413356; 17559573; 16508746; 16233230; 15842998 |
| NT01PH0756 | 15 | 0.92 | 17242400; 10851063; 16337145; 10486559; 15280358; 12821157; 12788093; 12554957; 12419230; 12207886 |
| NT01PH0799 | 18 | 0.99 | 20922376; 20737137; 20693325; 20639324; 20395880; 19937827; 15689108; 17625566; 1302283; 19432486 |
| NT01PH0909 | 4 | 0.75 | 20981284; 20553716; 19202875; 18297791; 17268768; 10788346; 7242389; 11790780; 10206874; 12480902 |
| NT01PH1375 | 18 | 0.94 | 20870774; 20467255; 9787636; 19095018; 17551219; 17159201; 17064369; 12563288; 16549425; 16524964 |
| NT01PH1388 | 18 | 0.80 | 21346154; 21231964; 21212855; 21130773; 20421301; 20376676; 20204435; 20118607; 19751706; 19664553 |
| NT01PH1649 | 8 | 0.78 | 21394524; 21372092; 21358310; 21344271; 21320626; 21316074; 21315380; 21311071; 21310534; 21270901 |
| NT01PH1842 | 18 | 0.88 | 20870774; 20467255; 20033069; 9787636; 14960717; 19095018; 10896219; 16030141; 17551219; 17350859 |
| NT01PH1931 | 15 | 0.87 | 21403627; 21402785; 21402776; 21401619; 21399658; 21398611; 21397653; 21397294; 21394201; 21392585 |
| NT01PI0554 | 6 | 0.88 | 21296012; 21251613; 21225631; 21192664; 21165562; 21350155; 21134274; 21078963; 21073875; 21044074 |
| NT01PI0651 | 19 | 0.86 | 21076971; 20875088; 20852022; 20336338; 19513709; 19500674; 19206107; 19022284; 18944957; 18944674 |
| NT01PI1422 | 8 | 0.78 | 16981725; 16278810; 16038606; 15667255; 15643882; 12615347; 10378274; 12351236; 11956744; 11807566 |
| NT01PI3264 | 8 | 0.95 | 20826660; 20160912; 19586787; 18164639; 17923481; 7569993; 9560191; 17157320; 14558820; 16219034 |
| NT01PI3666 | 1 | 0.75 | 21385584; 21383205; 21362417; 21219974; 21210868; 21138988; 21079812; 20971073; 20969952; 20958953 |
| NT01PI4195 | 8 | 0.80 | 21253866; 21036145; 20798971; 10672375; 20160912; 20022530; 9324032; 17667915; 17635929; 8798399 |
| NT01PL0019 | 11 | 0.74 | 21337470; 21214923; 21204936; 21196936; 21146538; 21041684; 20817773; 20799658; 20739276; 20733148 |
| NT01PL0220 | 15 | 0.92 | 20963440; 15937186; 14617181; 18194341; 18031348; 8830274; 15882427; 12471449; 1478454; 1447208 |
| NT01PL0337 | 15 | 0.86 | 20110300; 17674138; 11115115; 10360097; 11401724; 11115115; 2254248 |
| NT01PL0701 | 2 | 0.84 | 20734996; 11290749; 10829016; 10609891; 9278392; 3994382 |
| NT01PL1417 | 15 | 0.91 | 18511939; 19706608; 15755726; 19007420; 18445167; 18067539; 17238917; 11254632; 10411743; 7925310 |
| NT01PL1969 | 6 | 0.96 | 20724226; 16877383; 18616603; 12404116; 14592985; 17516097; 17416902; 17042786; 9406544; 16428325 |
| NT01PL2125 | 11 | 0.98 | 16621832; 16151213; 9274008; 1579110; 8594337; 7844814; 7753910; 2555940; 1331747; 2175363 |
| NT01PL2363 | 15 | 0.82 | 20522491; 20156450; 12824332; 10525169; 16954205; 12546643; 15667258; 12795406; 12139615; 9733973 |
| NT01PL2462 | 15 | 0.98 | 18355019; 12581359; 11750821; 1588910; 2125052; 1339463; 6281280; 1588910; 1367576; 6330500 |
| NT01PL2594 | 11 | 0.98 | 15731203; 12888597; 10476039; 10418136; 8393825; 1312499; 1090575; 2993811; 2993810; 6308391 |
| NT01PL2683 | 18 | 0.97 | 11043981; 3843705; 9157252; 128553; 8384682; 1552851; 379572; 7984105; 8245841; 8347944 |
| NT01PL2822 | 2 | 0.94 | 20936830; 20857974; 20734996; 20460376; 20054882; 19919618; 16905347; 17452319; 17350958; 17350000 |
| NT01PL2827 | 2 | 0.79 | 20942799; 20460376; 20097860; 19810706; 11983079; 15952888; 1060112; 16603772; 16905347; 2226775 |
| NT01PL3790 | 6 | 0.88 | 21262293; 21127984; 20970991; 20889709; 20739293; 20421419; 20403335; 19010785; 9182762; 12089521 |
| NT01PL4191 | 6 | 0.94 | 21131491; 20603075; 20304994; 20144564; 19925793; 19897571; 17965170; 19018518; 18972020; 18342610 |
| NT01PL4334 | 17 | 0.98 | 20624215; 12519953; 19942657; 12618438; 19843219; 18631157; 19383688; 14712681; 19008221; 18846815 |
| NT01PL4717 | 18 | 0.93 | 19911131; 1008746; 11700350; 9720051; 1379743; 3896791; 2674131 |
| NT01PL4871 | 11 | 0.83 | 20603069; 20167799; 12534466; 12504012; 17709741; 8599117; 17669422; 8675443; 10373363; 16181782 |
| NT01PL4888 | 12 | 0.90 | 21292975; 21248121; 21212461; 21209222; 21185288; 21170051; 21147124; 21119664; 21102654; 21205813 |
| NT01PM0778 | 15 | 0.98 | 21364902; 21357485; 21320584; 21317318; 21310787; 21295603; 21261468; 21216996; 21183673; 21179024 |
| NT01PM0821 | 8 | 0.73 | 19135450; 18284579; 18235971; 12631323; 16228611; 16128245; 13926592; 7502762; 7957557; 1545116 |
| NT01PM1327 | 12 | 0.71 | 10096074; 2129543; 2822665; 10508090; 1809829; 10972813; 8837412; 9668097; 9623911; 3768954 |
| NT01PM1517 | 18 | 0.88 | 20147290; 19808664; 19656948; 19298753; 18703537; 19166841; 19046020; 11050159; 11040062; 17698007 |
| NT01PP0231 | 19 | 0.89 | 20938646; 20225957; 19019161; 11497373; 10984043; 8529890 |
| NT01PP0306 | 8 | 0.90 | 21296885; 21228234; 21166653; 20889786; 20862513; 20809073; 20632934; 20171064; 19921179; 19395484 |
| NT01PP0318 | 8 | 0.74 | 21216999; 21097520; 20938339; 20850485; 20676631; 20675490; 20629071; 20219674; 20208557; 19904831 |
| NT01PP0914 | 8 | 0.73 | 21178285; 20811135; 19966484; 19546514; 19424620; 19200041; 17291445; 16402358; 16221991; 15996793 |
| NT01PP0916 | 8 | 0.73 | 21178285; 20811135; 19966484; 19546514; 19424620; 19200041; 17291445; 16402358; 16221991; 15996793 |
| NT01PP1068 | 4 | 0.90 | 20337712; 19899346; 19842384; 19735197; 18370608; 18243247; 17310071; 16421934; 7608087; 12408815 |
| NT01PP1283 | 15 | 0.84 | 18504019; 17040909; 9813128; 8809780; 2118656 |
| NT01PP1524 | 12 | 0.97 | 21148698; 20812717; 20615351; 20064164; 19754117; 19013157; 16320049; 16221580; 12935879; 9632249 |
| NT01PP1614 | 12 | 0.88 | 20799957; 20408570; 20023722; 16971147; 19066885; 18699780; 18590838; 18302298; 11152613; 17650679 |
| NT01PP1727 | 8 | 0.73 | 21178285; 20811135; 19966484; 19546514; 19424620; 19200041; 17291445; 16402358; 16221991; 15996793 |
| NT01PP1728 | 8 | 0.73 | 21178285; 20811135; 19966484; 19546514; 19424620; 19200041; 17291445; 16402358; 16221991; 15996793 |
| NT01PP1729 | 8 | 0.73 | 21178285; 20811135; 19966484; 19546514; 19424620; 19200041; 17291445; 16402358; 16221991; 15996793 |
| NT01PP1732 | 8 | 0.73 | 21178285; 20811135; 19966484; 19546514; 19424620; 19200041; 17291445; 16402358; 16221991; 15996793 |
| NT01PP1933 | 8 | 0.93 | 21357619; 20498375; 17054778; 16221580; 15924426; 14610638; 12679550; 12605683; 12560990; 12409197 |
| NT01PP2330 | 14 | 0.99 | 21361328; 21257770; 21257642; 21219207; 21131548; 21059659; 20952484; 20877497; 20842824; 20829075 |
| NT01PP2812 | 19 | 0.90 | 21054826; 20533408; 17933909; 19912589; 19833438; 18759077; 18673459; 10966453; 18385336; 17442906 |
| NT01PP2904 | 8 | 0.71 | 21159204; 21136602; 21103042; 21061425; 21055121; 20870774; 20852023; 20659153; 20618078; 20450584 |
| NT01PP3322 | 8 | 0.73 | 21374662; 21296938; 21278273; 21253866; 21236318; 21136949; 21036145; 20821193; 20803137; 20798971 |
| NT01PPA0001 | 4 | 0.76 | 21248157; 20888151; 19244914; 19648344; 19410003; 18786689; 17557077; 18562485; 18514370; 18380802 |
| NT01PPA0335 | 8 | 0.75 | 21207592; 20873810; 20675490; 20499905; 20491454; 20449078; 20378984; 17280684; 20153451; 20148387 |
| NT01PPA0480 | 18 | 0.99 | 21398536; 21398480; 21395979; 21395556; 21395536; 21394083; 21392495; 21391724; 21391691; 21388881 |
| NT01PPA0484 | 8 | 0.91 | 21400100; 21397737; 21396889; 21396131; 21394044; 21393444; 21393246; 21393237; 21388872; 21387012 |
| NT01PPA0491 | 8 | 0.73 | 21178285; 20811135; 19966484; 19546514; 19424620; 19200041; 17291445; 16402358; 16221991; 15996793 |
| NT01PPA0585 | 11 | 0.91 | 10829079; 17601790; 18363236; 17526845; 17379730; 16804187; 11136448; 7790093; 10087920; 10829077 |
| NT01PPA0720 | 1 | 0.93 | 21380856; 21349875; 21288239; 21267457; 21227480; 21191998; 21182990; 21178262; 21163840; 21146842 |
| NT01PPA0883 | 8 | 0.89 | 15629949; 18729428; 11473123; 16645312; 10209742; 3041370; 10671186; 15225600; 15028871; 8214582 |
| NT01PPA0897 | 8 | 0.86 | 21329681; 21120472; 19938610; 15389597; 19383697; 19115036; 17294170; 17222841; 16819729; 16027951 |
| NT01PPA1332 | 8 | 0.82 | 21377632; 21364005; 21362018; 21349860; 21347589; 21345790; 21343423; 21329681; 21325261; 21324174 |
| NT01PPA1386 | 11 | 0.96 | 17957106; 17573936; 12781722; 11410354; 9791169; 8472961; 1995430 |
| NT01PPA1521 | 8 | 0.73 | 21178285; 20811135; 19966484; 19546514; 19424620; 19200041; 17291445; 16402358; 16221991; 15996793 |
| NT01PPA1729 | 8 | 0.87 | 21371425; 21303655; 20696867; 20639325; 20576519; 20570675; 20548048; 20363598; 20304328; 20304089 |
| NT01PT0095 | 18 | 0.93 | 11855834; 1388167; 11096086; 8810286; 7629056; 8203905; 6755463; 2144188; 2163123; 6755463 |
| NT01PT0601 | 8 | 0.91 | 20655923; 20160912; 19996100; 18398624; 18362922; 18253149; 16531404; 14644451; 12603319; 11955070 |
| NT01PT0632 | 18 | 0.93 | 20938718; 18383009; 16817909; 12161466; 2826434; 10506587; 9711545; 8951813; 1447130; 1987150 |
| NT01PT0634 | 18 | 0.86 | 21401502; 21273450; 21262549; 21261465; 21253597; 21236318; 21220781; 21211813; 21207611; 21195693 |
| NT01PT0741 | 19 | 0.75 | 21347309; 21332624; 21292493; 21215778; 20868568; 21149454; 21134383; 21119090; 21064131; 21029479 |
| NT01PT0802 | 8 | 0.80 | 21366819; 21336929; 21309025; 21188613; 21168410; 21132340; 20937090; 20868295; 20818429; 20816746 |
| NT01PT0832 | 18 | 0.89 | 21367787; 21324969; 21248166; 21122131; 21038480; 20881245; 20816053; 20739622; 20709031; 20610400 |
| NT01PT1061 | 18 | 0.94 | 21254069; 21217751; 21134393; 21115899; 21106767; 21103975; 21103974; 21090806; 21087599; 21046154 |
| NT01PT1129 | 8 | 0.89 | 21398391; 21388533; 21388532; 21378355; 21367571; 21364306; 21359858; 21357267; 21355870; 21354561 |
| NT01PT1249 | 18 | 0.94 | 21397997; 21391546; 21390329; 21388519; 21387835; 21387378; 21385601; 21385112; 21383017; 21378974 |
| NT01PT1272 | 8 | 0.94 | 21398106; 21393861; 21392547; 21390523; 21387444; 21387033; 21376957; 21366264; 21364629; 21358763 |
| NT01PT1467 | 8 | 0.90 | 20803137; 15987803; 20433942; 3982017; 20079748; 19952500; 19583970; 19545176; 19389950; 19351325 |
| NT01PT1491 | 8 | 0.92 | 21369832; 21345474; 21323310; 21300282; 21261469; 21239558; 21068394; 21048403; 21037180; 20956531 |
| NT01PT1516 | 8 | 0.97 | 20610779; 19904590; 18454933; 18358763; 17634376; 17244482; 16098512; 12914915; 12615344; 12515529 |
| NT01PT1529 | 14 | 0.86 | 21212540; 21128666; 21079776; 20951473; 20860090; 20833052; 20593468; 20516559; 6254974; 20497505 |
| NT01PT1560 | 19 | 0.97 | 21193820; 21126522; 21058504; 20971639; 20824214; 20816775; 20802066; 20798166; 20720016; 20714719 |
| NT01PT1614 | 8 | 0.97 | 21403907; 21400553; 21397011; 21392546; 21387444; 21381897; 21374662; 21349883; 21346867; 21338644 |
| NT01RD1958 | 12 | 0.98 | 19200026; 17328877; 8951032; 7991596; 7819336; 8102366 |
| NT01RD2951 | 8 | 0.84 | 21383078; 21082361; 21067517; 20581474; 20405931; 20303981; 20038140; 19845618; 7860587; 15269332 |
| NT01RD3678 | 18 | 0.71 | 19914235; 19036317; 12975461; 17255106; 15558041; 11676004; 11375517; 8977098; 14488584; 1279804 |
| NT01RDA0117 | 12 | 0.82 | 21115803; 21029479; 20217243; 2248769; 16543934; 18543065; 18234743; 10693756; 17267054; 17051148 |
| NT01RE0375 | 8 | 0.74 | 21266382; 21167962; 20624914; 20622059; 20436434; 19791729; 19780766; 19772191; 19625251; 19416715 |
| NT01RE1771 | 8 | 0.76 | 21343423; 21185310; 21073854; 20621795; 20547751; 20516592; 20418438; 20370610; 20150517; 20133363 |
| NT01RE2883 | 15 | 0.99 | 21357483; 20133363; 16945692; 16204505; 4352175; 18245243; 18093135; 10679470; 16455652; 16339930 |
| NT01RE2973 | 6 | 0.75 | 19840758; 10861942; 11773415; 16359314; 15878881; 15581572; 15333942; 15133035; 15053875; 12509230 |
| NT01RE3116 | 18 | 0.98 | 21349151; 21320185; 21288822; 21265893; 21227585; 21226332; 21187898; 21175590; 21168419; 21135102 |
| NT01REA0583 | 19 | 0.96 | 18508770; 18184612; 9426598; 15748978; 15552059; 12851288; 11846551; 11551746; 10393171; 11018032 |
| NT01REA0720 | 15 | 0.96 | 21315771; 21284862; 21217003; 21216906; 21193607; 21078995; 21037181; 20979345; 20951027; 20946856 |
| NT01REA1578 | 6 | 0.78 | 11766963; 21362445; 20662778; 16952955; 18959764; 17698626; 18424443; 7929618; 11931626; 18281611; 18177465 |
| NT01REA1740 | 9 | 0.99 | 20433156; 20176020; 10404163; 20064615; 20057061; 19933275; 19733180; 19573516; 18837378; 18770515 |
| NT01REB0330 | 11 | 0.93 | 12951335; 19593027; 19592961; 15528650; 13677658; 9351174; 2825175; 1409650; 8594337; 2555940 |
| NT01RL0052 | 4 | 0.71 | 20566763; 20478990; 20046086; 7486920; 18295586; 11134934; 18095712; 17317190; 16913716; 16131761 |
| NT01RL1095 | 15 | 0.99 | 20946846; 20817745; 19411418; 17183208; 6550579; 12614149; 17908933; 16345414; 10924135; 10500846 |
| NT01RL1489 | 8 | 0.74 | 20600130; 19915006; 19744497; 18440023; 18355283; 18034235; 17892308; 17675148; 17181540; 16893166 |
| NT01RL1597 | 8 | 0.81 | 19428350; 16897483; 15623532; 9784233; 7770449; 7948919 |
| NT01RL1603 | 19 | 0.98 | 20201406; 17934909; 14646108; 11082184; 2445222 |
| NT01RL1638 | 8 | 0.94 | 21249616; 19880789; 19593802; 19567539; 17192572; 8370690; 18252960; 10415113; 9543163; 17673214 |
| NT01RL2272 | 13 | 0.84 | 21364908; 21357738; 21274405; 21273291; 20807211; 20798174; 20691699; 20626565; 20566627; 9626666 |
| NT01RL2731 | 12 | 0.77 | 20388708; 17215254; 9527842; 15732910; 15464143; 14741712; 10945979; 12686540; 8075118; 9765270 |
| NT01RL2837 | 12 | 0.72 | 21398603; 21397717; 21376116; 21375730; 21364925; 21354366; 21354180; 21349976; 21343359; 21342099 |
| NT01RL3196 | 19 | 0.76 | 21146536; 20622465; 20544772; 20507981; 20118260; 20079707; 19952412; 19948209; 19704161; 19553349 |
| NT01RL3317 | 19 | 0.88 | 20724279; 2283426; 12498145; 12484750; 12426223; 10481063; 8789344; 2722748; 1406248; 2311931 |
| NT01RL3538 | 12 | 0.87 | 20594613; 18705851; 11457979; 2535528; 1946465 |
| NT01RL3575 | 8 | 0.77 | 21399654; 21396997; 21370474; 21369715; 21358504; 21338238; 21330949; 21295977; 21294799; 21281628 |
| NT01RL3630 | 19 | 0.86 | 21379570; 21276045; 21255117; 21183069; 21053951; 20961110; 20956585; 20944066; 20870765 |
| NT01RL3647 | 19 | 0.89 | 21388958; 21378160; 21367655; 21350158; 21344734; 21342516; 21332624; 21307593; 21300542; 21299880 |
| NT01RL3714 | 19 | 0.89 | 21369973; 21369910; 21357626; 21357429; 21311881; 21307589; 21297160; 21282527; 21256135; 21245531 |
| NT01RL3740 | 13 | 0.86 | 20402779; 19916630; 16421450; 18279892; 8592456; 10727213; 10811628; 10500847; 16030207; 15856304 |
| NT01RL4143 | 9 | 0.94 | 21382701; 21381205; 21369973; 21347309; 21322560; 21291119; 21279344; 21262713; 21254164; 21253498 |
| NT01RL4380 | 4 | 0.73 | 21182780; 20979332; 20951638; 20884343; 20846031; 20677943; 20675470; 20668846; 20589424; 20546749 |
| NT01RL4914 | 19 | 0.92 | 21307593; 21174947; 21056976; 21036948; 20852022; 20826810; 20739278; 20709893; 20686915; 20682344 |
| NT01RLD0106 | 8 | 0.92 | 10940013; 2029896; 2403367; 6342621; 7159554 |
| NT01RLE0261 | 19 | 0.97 | 21320184; 21301102; 21194491; 21135211; 20956585; 20847002; 345275; 20487272; 20417640; 20400208 |
| NT01RLE0355 | 19 | 0.80 | 9736698; 12094214; 17234634; 9556600; 9358065; 8911985; 8543020 |
| NT01RLE0413 | 19 | 0.78 | 18007245; 15864725; 10074749; 9309739; 1356879; 2560400; 403241 |
| NT01RLE0616 | 12 | 0.94 | 21400094; 21385872; 21381030; 21375706; 21369989; 21355788; 21354644; 21352460; 21346188; 21331075 |
| NT01RLE0652 | 8 | 0.93 | 21335525; 21284395; 21265508; 21219905; 21212358; 21178165; 21138528; 21071046; 21069144; 21068394 |
| NT01RLF0085 | 6 | 0.78 | 10366527; 16893567; 14644498; 12876287; 11948448; 11278349; 9278140; 10899169; 10764793; 2993811 |
| NT01RLF0283 | 19 | 0.92 | 21044320; 20472799; 18835420; 18557786; 9620971; 10375631 |
| NT01RLF0382 | 8 | 0.90 | 20707404; 20508929; 20173089; 19571038; 12899689; 19222584; 19096101; 19000905; 18588320; 18455185 |
| NT01RM0288 | 8 | 0.79 | 20624914; 20622059; 19625251; 19416715; 19373256; 19295170; 18582433; 18045776; 11782424; 17761683 |
| NT01RM1046 | 8 | 0.71 | 21223465; 21153706; 21083422; 21082227; 20857400; 20820784; 20600382; 20196923; 20180116; 20154096 |
| NT01RM2065 | 18 | 0.82 | 21346170; 21342462; 21151985; 21151967; 21076391; 21059846; 21037388; 20963440; 21183694; 20947498 |
| NT01RM2436 | 8 | 0.76 | 20620191; 20610843; 20351055; 1687097; 20074091; 20036764; 19966511; 19958171; 19936779; 19931534 |
| NT01RM3606 | 5 | 0.99 | 21208503; 21095459; 20838441; 20095974; 19523496; 19405119; 19185952; 19023869; 18375607; 18308526 |
| NT01RMB0150 | 12 | 0.79 | 20230833; 19526727; 19159700; 18948221; 17971396; 12437884; 16547004; 16388577; 10479292; 12729748 |
| NT01RP0085 | 18 | 0.87 | 21124774; 20116460; 19486157; 17217961; 16414958; 10323882; 9426139; 1283000 |
| NT01RP0138 | 12 | 0.70 | 20599668; 19853572; 19804735; 16307111; 17766116; 18387365; 18339324; 17980516; 16834335; 16573693 |
| NT01RP0139 | 12 | 0.75 | 20713651; 20599668; 19853572; 1619456; 18387365; 17556688; 17269451; 16834335; 16573693; 16389450 |
| NT01RP0238 | 8 | 0.77 | 20712413; 20522499; 20498481; 20488891; 20381373; 20351055; 20231437; 1687097; 20085751; 19914726 |
| NT01RP0309 | 8 | 0.79 | 21219744; 21046346; 20962902; 20814880; 20695278; 20529851; 20496592; 17438062; 20484676; 20367096 |
| NT01RP0330 | 12 | 0.74 | 21195664; 20426418; 20065028; 20033031; 19436993; 10678941; 2270973; 19054325; 12700266; 15618402 |
| NT01RP0338 | 5 | 0.82 | 21087930; 2138605; 15580782; 10224133; 9647837; 10200269; 17450323; 14585934; 7972072; 7493934 |
| NT01RP0352 | 18 | 0.81 | 21285349; 20870776; 20221740; 19650773; 19519769; 19075020; 18269247; 17982123; 10474183; 3915176 |
| NT01RP0562 | 8 | 0.90 | 21261075; 21186173; 20557574; 20455436; 20227379; 20202167; 16880; 19825618; 19705487; 15685292 |
| NT01RP0771 | 4 | 0.94 | 21321206; 21216997; 21145019; 21097625; 20943430; 20825347; 20807205; 4870278; 20543068; 20497503 |
| NT01RS0152 | 15 | 0.96 | 1312518; 9781878; 16529980; 16353555; 11773048; 12650454; 11929528; 10421756; 11321588; 1447147 |
| NT01RS0252 | 11 | 0.89 | 20154109; 10499270; 9729608; 9737923; 9737923; 9571135; 9141667; 2666844; 3018434; 2540414 |
| NT01RS0338 | 8 | 0.82 | 15047692; 2033048; 1069307; 18422485; 1310666; 15047692; 12637552 |
| NT01RS0396 | 8 | 0.77 | 19290553; 16596388; 15859247; 14747735; 10939523 |
| NT01RS0461 | 18 | 0.96 | 21150302; 21029514; 20980454; 20880843; 20846323; 20601877; 20589886; 20560207; 20543968; 20512921 |
| NT01RS0687 | 3 | 0.92 | 17581122; 16403447; 15101991; 12040098; 9767592; 9224880; 8955641; 7565106; 7783642; 7908398 |
| NT01RS0688 | 3 | 0.83 | 17581122; 15629949; 19508279; 18485000; 15101991; 12040098; 9767592; 7908398 |
| NT01RS0690 | 3 | 0.92 | 17713814; 10984043; 15101991; 12040098; 7783642; 7908398 |
| NT01RS0794 | 8 | 0.74 | 20801213; 7961454; 19212665; 18021062; 8116881 |
| NT01RS1367 | 15 | 0.87 | 2965141; 1729202; 9218420; 7968519; 2493441 |
| NT01RS1402 | 19 | 0.84 | 20144715; 18342249; 17641929; 17056114; 12502707 |
| NT01RS2846 | 4 | 0.81 | 21386816; 20847002; 20729359; 20497333; 16146521; 18832310; 10564478; 17938168; 17185541; 15165230 |
| NT01RS3253 | 8 | 0.76 | 12482588; 10581551; 7939681; 1654798; 1846290 |
| NT01RSA0148 | 9 | 0.88 | 20013982; 19954230; 19777301; 19733180; 18451049; 18319060; 11036020; 17237222; 16793520; 15033238 |
| NT01RSA0368 | 9 | 0.98 | 21358050; 21357626; 21235239; 20797606; 20696925; 20563648; 20406823; 11251294; 3537305; 20013982 |
| NT01RSA0557 | 11 | 0.97 | 16181782; 18227257; 17367389; 10722135; 10986230; 9393713; 2548993; 10760133; 11958563; 11907683 |
| NT01RSA0558 | 11 | 0.99 | 16181782; 17078817; 10722135; 10986230; 10556026; 9393713; 2548993; 10760133; 11958563; 11907683 |
| NT01RSA0568 | 11 | 0.99 | 16181782; 17078817; 10722135; 10986230; 10556026; 9393713; 2548993; 10760133; 11958563; 11907683 |
| NT01RSA0671 | 9 | 0.87 | 21155016; 20826333; 20133127; 19452559; 11208803; 11851409; 15554960; 15476969; 12655555; 9400389 |
| NT01RSA0695 | 8 | 0.71 | 19946124; 2294092; 18384884; 17294609; 17198384; 16450123; 16075385; 12711393; 11469796; 8245962 |
| NT01RSA0991 | 8 | 0.81 | 19880510; 18252249; 17137322; 16388583; 15812642; 15755419; 12914529; 12604214; 12562758; 11533060 |
| NT01RSA1333 | 15 | 0.91 | 21335458; 11018148; 3507689; 9987125; 9632252; 2199796; 9484898; 8246839; 1783189; 1479894 |
| NT01RSA1335 | 15 | 0.91 | 21335458; 11018148; 3507689; 9987125; 9632252; 2199796; 9484898; 8246839; 1783189; 1479894 |
| NT01RSA1616 | 8 | 0.73 | 21178285; 20811135; 19966484; 19546514; 19424620; 19200041; 17291445; 16402358; 16221991; 15996793 |
| NT01RT0218 | 8 | 0.73 | 21178285; 20811135; 19966484; 19546514; 19424620; 19200041; 17291445; 16402358; 16221991; 15996793 |
| NT01RT0337 | 19 | 0.91 | 21376799; 21279381; 21278296; 21196935; 21174346; 21161258; 21057009; 20636376; 20595206; 11251855 |
| NT01RT0557 | 8 | 0.71 | 21159204; 21136602; 21103042; 21061425; 21055121; 20870774; 20852023; 20659153; 20618078; 20450584 |
| NT01RU1258 | 18 | 0.92 | 21099448; 20933223; 20817004; 20718731; 20354142; 20164366; 19894125; 19543710; 19372168; 11129050 |
| NT01RX0258 | 9 | 0.98 | 21294903; 20923423; 20725044; 20690600; 20616867; 20129920; 1355089; 19926919; 10744768; 18992226 |
| NT01RX0547 | 8 | 0.89 | 20879797; 20699381; 19439190; 18978174; 18232632; 17628001; 15769597; 15347626; 2055098; 2675762 |
| NT01RX2063 | 12 | 0.81 | 20486678; 4778946; 19784642; 19426854; 19178938; 19126663; 18207562; 18158172; 16166273; 16144506 |
| NT01RX3213 | 8 | 0.71 | 19117949; 19006332; 18793172; 14740890; 13678276; 12206896; 11860176; 11357863; 10371157; 10220277 |
| NT01SA0215 | 8 | 0.83 | 2123834; 19628714; 11396927; 9397158; 9252455; 8665954; 8612675; 1329869; 7685596; 2222460 |
| NT01SA1428 | 18 | 0.77 | 21228159; 21187398; 21129771; 21129209; 21107421; 21104093; 20860462; 20858774; 20858777; 20825633 |
| NT01SA1549 | 4 | 0.87 | 15819619; 17172014; 16209911; 15458414; 9746358; 9701804; 7565874; 7968523 |
| NT01SA1745 | 8 | 0.96 | 21152918; 21042417; 20958226; 20943545; 20922418; 20858453; 20826797; 20823090; 20808932; 20802042 |
| NT01SA1929 | 12 | 0.76 | 21273509; 8662184; 15528186; 11160090; 19114107; 18673393; 18439585; 18166993; 17592722; 17439951 |
| NT01SA1990 | 12 | 0.90 | 21398423; 21394648; 21394209; 21393220; 21393166; 21389542; 21389146; 21388810; 21375591; 21372091 |
| NT01SA2150 | 11 | 0.85 | 20602451; 20449796; 10868275; 18593511; 18031956; 17609978; 8755548; 11158729; 11691926; 12354653 |
| NT01SF0059 | 12 | 0.97 | 21231916; 20856808; 20847048; 20803087; 20615403; 20494979; 20418496; 20113313; 20111006; 20060408 |
| NT01SF0060 | 13 | 0.92 | 20817755; 17937767; 15215462; 12762017; 1324907 |
| NT01SF0288 | 8 | 0.91 | 21365653; 21222161; 21163840; 21141873; 20652236; 5420057; 20496344; 20163845; 20054120; 20014430 |
| NT01SF0533 | 18 | 0.70 | 20487273; 20111865; 11831459; 10377096; 9712811; 15459199; 10425169; 10743563; 1416876; 7608184 |
| NT01SF0539 | 11 | 0.86 | 21330133; 21124920; 20875143; 20863318; 20817910; 20541928; 20481467; 20478417; 20118935; 20096705 |
| NT01SF0728 | 8 | 0.76 | 21400205; 21398409; 21398019; 21397656; 21396999; 21396437; 21396424; 21395651; 21394524; 21394467 |
| NT01SF0844 | 11 | 0.88 | 21214923; 21041684; 19241380; 12079349; 9925584; 11244082; 16496690; 16453083; 12351820; 10500998 |
| NT01SF0932 | 18 | 0.93 | 21371898; 21315686; 21310270; 21308399; 21170876; 21261641; 21247492; 21245145; 21238925; 21209283 |
| NT01SF0978 | 6 | 0.93 | 17108184; 12071693; 16675461; 12946629; 12239560; 15493325; 15476890; 1552845; 3071688; 11138010 |
| NT01SF1166 | 19 | 0.83 | 20687615; 20600864; 20522722; 16101996; 17761750; 16275672; 16915710; 16049108; 15998313; 12627387 |
| NT01SF1273 | 18 | 0.83 | 21207455; 21143936; 21114408; 21081547; 20881245; 20877283; 20870764; 20730247; 20656779; 20626869 |
| NT01SF1955 | 12 | 0.92 | 21347511; 21207565; 20800743; 20707600; 20597098; 20586483; 20442413; 20208362; 19883788; 19874678 |
| NT01SF2057 | 8 | 0.86 | 21069144; 21305143; 17854180; 17549795; 15902489; 15772818; 12646191; 10024458; 11511200; 11265456 |
| NT01SF2072 | 18 | 0.95 | 21345797; 21163357; 21131438; 21122131; 21111784; 21098040; 21090806; 21057044; 20935152; 20888319 |
| NT01SF2211 | 18 | 0.87 | 21398553; 21397337; 21393457; 21393220; 21386968; 21378194; 21361323; 21353274; 21350490; 21342097 |
| NT01SF2216 | 18 | 0.93 | 21191290; 21122813; 20443692; 20197031; 20091106; 20028803; 19937979; 17493798; 19780400; 19093971 |
| NT01SF2386 | 8 | 0.74 | 21389880; 21348513; 21346758; 21346565; 21339577; 21327766; 21324401; 21321076; 21320164; 21301994 |
| NT01SF2464 | 11 | 0.72 | 20628359; 20606646; 19679697; 16179923; 17015820; 16474888; 12842434; 7483287 |
| NT01SF2571 | 19 | 0.77 | 30701; 12883005; 17524545; 8114723; 15639623; 2436229; 11069242; 1712763; 12726766; 9515923 |
| NT01SF2695 | 9 | 0.81 | 20590527; 20370610; 20178986; 20099411; 20080211; 19686777; 19571038; 19525201; 15461798; 19444866 |
| NT01SF2833 | 18 | 0.91 | 21375706; 21293192; 21291419; 21273531; 21245145; 21238925; 21209283; 21205795; 21191290; 21190950 |
| NT01SF2849 | 12 | 0.88 | 21385872; 21366301; 21212512; 21176936; 21139195; 21056043; 20833209; 20824821; 20667603; 20652663 |
| NT01SF2964 | 19 | 0.96 | 21311881; 21307602; 21273488; 21193346; 21161227; 21114521; 20937888; 20826214; 20820855; 20812131 |
| NT01SF2976 | 15 | 0.89 | 19116917; 18777041; 17287440; 16725275; 16616847; 16000707; 15680700; 15583171; 15183729; 13129619 |
| NT01SF3309 | 8 | 0.83 | 9973347; 12639950; 11835514; 10203754; 9383150; 7894055; 8397187; 2134185 |
| NT01SF3588 | 6 | 0.93 | 21071401; 20944244; 20675469; 20026132; 17827721; 18414490; 12019088; 15550775; 10446921; 12691822 |
| NT01SF3761 | 13 | 0.72 | 21320742; 20696925; 14627813; 17868095; 20052680; 10880568; 19894214; 19851001; 19720141; 14622403 |
| NT01SF3762 | 13 | 0.81 | 21295137; 21282428; 21175197; 21144880; 21042822; 20954242; 20796028; 11342140; 20512599; 20510245 |
| NT01SF3850 | 3 | 0.73 | 18943053; 9786194; 17633193; 17448348; 3022192; 2445686; 6111541 |
| NT01SF4126 | 4 | 0.77 | 19390444; 17159215; 16006488; 11814663; 11178251; 1365398; 10361613; 1715856; 8178371; 8629932 |
| NT01SG0712 | 12 | 0.91 | 21403683; 21402859; 21402719; 21402511; 21402045; 21401044; 21399329; 21398603; 21398524; 21398495 |
| NT01SG0807 | 1 | 0.75 | 19730818; 19139748; 18625006; 16775081; 6803789; 15843022; 15756463; 15210152; 9862121; 8227040 |
| NT01SG0953 | 11 | 0.75 | 19503942; 765483; 11452024; 16956798; 16125724; 12467643; 11823865; 11095248; 10756710; 1724963 |
| NT01SG1029 | 19 | 0.82 | 21193672; 20871101; 20553325; 20061477; 20030726; 19682076; 18312393; 5323018; 15980069; 14651342 |
| NT01SG1571 | 15 | 0.73 | 21385583; 21333636; 21326370; 21284215; 21276270; 21070403; 20877641; 20870898; 20724380; 20678145 |
| NT01SG1674 | 6 | 0.73 | 18832328; 17698626; 17087993; 11359568; 10564465; 15948955; 12634337; 15212787; 15208453; 14584922 |
| NT01SG5003 | 8 | 0.85 | 20421510; 20363758; 20335404; 12874335; 1829523; 18174141; 16861801; 10671461; 15366932; 15233785 |
| NT01SG5808 | 8 | 0.80 | 21377212; 21107143; 20733506; 20719394; 20466433; 19457771; 19731149; 18947880; 18752443; 18589628 |
| NT01SG6711 | 18 | 0.92 | 11855834; 1388167; 11096086; 10089335; 8810286; 7629056; 8203905; 6755463; 2163123; 6755463 |
| NT01SH0109 | 6 | 0.94 | 17446283; 16125907; 12488074; 11092734; 9837717; 9383194; 7909397 |
| NT01SH0953 | 15 | 0.74 | 21223465; 21103340; 19851730; 19708918; 19688348; 19517810; 18335939; 19146538; 19064249; 18713030 |
| NT01SH1643 | 18 | 0.81 | 19933751; 6777774; 11356312; 12893936; 1679551; 14559353; 12856948; 8833341; 10590270; 8916917 |
| NT01SH2054 | 8 | 0.91 | 21400100; 21397737; 21396889; 21396131; 21394044; 21393444; 21393246; 21393237; 21388872; 21387012 |
| NT01SH2165 | 12 | 0.79 | 21367858; 21277964; 21229607; 21229178; 21061271; 21056894; 20976535; 20970105; 20854261; 20844317 |
| NT01SH2312 | 18 | 0.87 | 21289433; 20804584; 20718750; 20454973; 20096615; 20035738; 236308; 19811460; 19713739; 19682108 |
| NT01SH2516 | 19 | 0.84 | 21367514; 21178486; 21176138; 21167286; 21167199; 21085702; 21073231; 20980996; 20980266; 20803062 |
| NT01SM2707 | 6 | 0.95 | 21383100; 21372129; 21332098; 21329318; 21300955; 21300644; 21298832; 21292748; 21281597; 21236644 |
| NT01SM4345 | 6 | 0.95 | 21383776; 21264139; 21261287; 21243571; 21063097; 20972458; 20961055; 20952393; 20951509; 20932474 |
| NT01SM4524 | 18 | 0.87 | 20130896; 19686236; 15928039; 18645191; 17937777; 16702300; 16411015; 16260085; 16184434; 16102820 |
| NT01SMA1359 | 18 | 0.97 | 20957165; 20737137; 20704181; 20639324; 20561140; 20395595; 20185911; 16099468; 6467192; 19775248 |
| NT01SMB0462 | 18 | 0.99 | 10541556; 15694325; 16308273; 16249326; 15014918; 10692314; 12675852; 10964703; 10215934; 9833966 |
| NT01SMB0820 | 6 | 0.95 | 21164569; 20729316; 20662765; 20406687; 20138014; 2087220; 19150358; 2649886; 17965170; 19018518 |
| NT01SMB0998 | 19 | 0.95 | 20957510; 19332829; 16002992; 12055294; 9748211; 9792849; 9570402; 1834675 |
| NT01SMB1187 | 14 | 0.99 | 21361328; 21257770; 21257642; 21219207; 21131548; 21059659; 20952484; 20877497; 20842824; 20829075 |
| NT01SMB1956 | 6 | 0.89 | 21375706; 21369983; 21349326; 21343600; 21301087; 21300948; 21289117; 21287257; 21284850; 21264493 |
| NT01SP0070 | 2 | 0.88 | 21282208; 20189102; 20064433; 16321944; 15840497; 15649375; 11468415; 9701598 |
| NT01SP0277 | 15 | 0.83 | 21371090; 12799345; 16088826; 11136460; 10433581; 17608796; 19202105; 18460666; 17600057; 18824088 |
| NT01SP1384 | 8 | 0.89 | 16664915; 19995806; 19995805; 18667575; 19140471; 18967091; 18676724; 18614804; 19820353; 18490438 |
| NT01SP1449 | 12 | 0.96 | 21397265; 21393861; 21382374; 21379584; 21378058; 21373826; 21342561; 21369706; 21362463; 21357544 |
| NT01SP1505 | 6 | 0.86 | 21386893; 21362621; 21354613; 21342128; 21339608; 21335605; 21333363; 21332166; 21276096; 21266475 |
| NT01SP1530 | 8 | 0.73 | 21178285; 20811135; 19966484; 19546514; 19424620; 19200041; 17291445; 16402358; 16221991; 15996793 |
| NT01SS0008 | 1 | 0.84 | 20731789; 15486256; 9817848; 7676624; 3338988 |
| NT01SS0012 | 5 | 0.96 | 21356439; 21330782; 21312326; 21282323; 21212863; 21192794; 21139196; 21055403; 21043115; 20876533 |
| NT01SS0036 | 18 | 0.86 | 21183643; 21080032; 20805337; 20647000; 20601471; 20308306; 20190043; 20102440; 19911130; 19894125 |
| NT01SS0064 | 6 | 0.87 | 21300638; 20139184; 1715862; 20134056; 15225322; 10481021; 10458907; 8876701; 1657879; 1103845 |
| NT01SS0148 | 19 | 0.98 | 19576307; 18494783; 18051301; 17321685; 16333636; 14996213; 14593480; 10982190; 10571954; 388439 |
| NT01SS0295 | 12 | 0.91 | 17420601; 17213638; 10791715; 15964663; 15207487; 12923788; 1429855; 9159391; 7649995 |
| NT01SS0386 | 13 | 0.96 | 21054500; 20332778; 20185543; 20132446; 19962967; 19366171; 19239245; 19170772; 19167989; 19150742 |
| NT01SS0431 | 19 | 0.72 | 19732381; 942051; 12460945; 19559061; 19321844; 19017269; 18939472; 17986089; 3053713; 18343875 |
| NT01SS0783 | 18 | 0.94 | 21346101; 21229381; 21178077; 21147712; 21062066; 21028901; 20950636; 20930652; 20929212; 20883039 |
| NT01SS0937 | 13 | 0.98 | 21054500; 20132820; 19840122; 9514735; 19035880; 2687846; 18951960; 18758445; 18672904; 11557883 |
| NT01SS0964 | 19 | 0.98 | 20700743; 20197075; 20164147; 20045480; 20043969; 19884781; 19576307; 19428473; 18174188; 19217615 |
| NT01SS0977 | 17 | 0.98 | 21124318; 21073264; 21071440; 21070967; 21057108; 21040729; 20972363; 20956010; 20952386; 20943161 |
| NT01SS1083 | 18 | 0.78 | 21399401; 21397935; 21397641; 21397601; 21396230; 21396226; 21389247; 21383318; 21376599; 21375527 |
| NT01SS1159 | 6 | 0.85 | 19208629; 11839499; 11781105; 8810301; 10199404 |
| NT01SS1242 | 18 | 0.81 | 20237644; 17644517; 16971936; 3403505; 16107340; 16101450; 17185584; 12195740; 9712774; 9575204 |
| NT01SS1250 | 2 | 0.94 | 21141924; 20937134; 19953200; 19821612; 19328201; 19245793; 9920791; 18022560; 10819988; 16099603 |
| NT01SS1276 | 13 | 0.97 | 19889645; 19106621; 11348589; 10811628; 10518522; 15614972; 12521300; 12009902 |
| NT01SS1308 | 19 | 0.75 | 21097580; 20668486; 20642807; 18818215; 20127467; 20118250; 17888883; 11069242; 19093149; 19025571 |
| NT01SS1342 | 17 | 0.72 | 21385588; 21382107; 21378195; 21378190; 21371479; 21347346; 21332625; 21324192; 21316374 |
| NT01SS1346 | 15 | 0.91 | 21071627; 21051490; 20862323; 20626317; 20511506; 20562301; 20148929; 17487300; 17379732; 17213678 |
| NT01SS1572 | 18 | 0.88 | 21304920; 20889743; 20873853; 20799927; 20730534; 8202535; 20496864; 18931304; 20363939; 20100880 |
| NT01SS1578 | 2 | 0.83 | 21047120; 20959560; 20692224; 20458544; 18849445; 19491146; 19234759; 10075836; 18215430; 17375528 |
| NT01SS1656 | 18 | 0.84 | 21273509; 21228780; 21221114; 21220218; 21187401; 21187073; 21139678; 21108950; 21068392; 21068384 |
| NT01SS1696 | 15 | 0.96 | 21385202; 21306428; 21217007; 21183673; 21115262; 20946846; 20870771; 20817745; 20805330; 20722734 |
| NT01SS1792 | 14 | 0.73 | 21394111; 21304254; 21266913; 21246601; 21211508; 21111048; 21071429; 21035468; 20981450; 20956937 |
| NT01SS1942 | 8 | 0.73 | 21357486; 21322495; 21322489; 21319715; 21299212; 21277857; 21277289; 21220625; 21216999; 21209222 |
| NT01SS1978 | 14 | 0.88 | 19683509; 15539408; 12538648; 11916376; 10777501; 1658539 |
| NT01SS2005 | 9 | 0.99 | 21369993; 21307609; 21257056; 21184438; 21173264; 21124062; 21104442; 21083519; 20920595; 10747865 |
| NT01SS2016 | 12 | 0.77 | 20736484; 20583963; 19953351; 18759457; 17942943; 16580629; 18187053; 17942943; 17113038; 16875662 |
| NT01SS2022 | 8 | 0.93 | 21181421; 21081480; 21068384; 20971856; 20952576; 20876281; 20732359; 20624914; 20622059; 20518024 |
| NT01SS2340 | 18 | 0.72 | 19882355; 19358815; 17141570; 16696735; 16549277; 16207882; 15965656; 15540989; 12201039; 10639734 |
| NT01SS2435 | 8 | 0.76 | 21377371; 21357486; 21338950; 21322495; 21322491; 21322489; 21320784; 21319715; 21310270; 21308956 |
| NT01SS2437 | 9 | 0.95 | 21369993; 21362034; 21328464; 21318631; 21310868; 21308896; 21307609; 21222433; 21191990; 21184438 |
| NT01SS2761 | 18 | 0.88 | 21371898; 21369825; 21310270; 21308399; 21170876; 21261641; 21256461; 21247492; 21245145; 21238925 |
| NT01SS2805 | 2 | 0.81 | 21388533; 21379374; 21335601; 21296950; 21266015; 21143723; 20718073; 20660776; 20552454; 20491066 |
| NT01SS3075 | 19 | 0.72 | 19732381; 942051; 12460945; 19559061; 19321844; 19017269; 18939472; 17986089; 3053713; 18343875 |
| NT01SS3187 | 18 | 0.72 | 19882355; 19358815; 17141570; 16696735; 16549277; 16207882; 15965656; 15540989; 12201039; 10639734 |
| NT01SS3224 | 9 | 0.84 | 21389045; 21388747; 21375498; 21371360; 21362034; 21345233; 21331774; 21330637; 21327327; 21315740 |
| NT01SS3275 | 6 | 0.87 | 21402758; 21398634; 21383239; 21382109; 21372093; 21369983; 21362510; 21362508; 21354180; 21353876 |
| NT01SS3469 | 15 | 0.88 | 21108067; 21097618; 21078995; 21050859; 21037181; 20979345; 20971918; 20966074; 20951027; 20946859 |
| NT01SS3474 | 15 | 0.95 | 15179604; 10547699; 11309119; 10422261; 10998244; 9278412 |
| NT01SS3481 | 18 | 0.94 | 20118641; 19168988; 18702072; 18343670; 17251264; 16751629; 16730200; 16394583; 16342953; 15925480 |
| NT01SS3499 | 19 | 0.82 | 21378199; 15009891; 9869563; 10368136; 2842151; 10094630; 4347536; 803965; 3080407 |
| NT01SS3543 | 8 | 0.71 | 21177473; 20960122; 20943284; 19577535; 15817393; 19067956; 18593701; 18543768; 18302324; 18221490 |
| NT01SS3599 | 2 | 0.94 | 19646414; 19291068; 11172074; 15634204; 10437802; 393803; 5432063 |
| NT01SS3609 | 15 | 0.79 | 21311751; 21295603; 21183673; 21169449; 20843371; 20731460; 20671191; 20571952; 17582720; 20233304 |
| NT01SS3739 | 5 | 0.94 | 17891922; 12207230; 9503607; 8840505; 7947754; 8492805; 2664422 |
| NT01SS3764 | 2 | 0.88 | 20838591; 15967443; 18959769; 18490451; 18651751; 3346071; 10891285; 16049659; 15967443; 15937336 |
| NT01SS3765 | 8 | 0.80 | 20952576; 18033741; 17706591; 17501927; 12356333; 12524212; 12421312; 1322289; 11418186; 1840615 |
| NT01SS3926 | 8 | 0.90 | 21296048; 21287605; 21265782; 21192786; 21109633; 21164002; 20675088; 20669924; 20629638; 20622453 |
| NT01ST0130 | 15 | 0.73 | 11750821; 11521084; 9570402; 7642494; 7827509; 1805309; 2687118; 6263857 |
| NT01ST0428 | 18 | 0.96 | 19342493; 20111865; 19361527; 19254725; 19199920; 18977099; 16979625; 18556176; 8808924; 18048926 |
| NT01ST0487 | 6 | 0.93 | 21388430; 21386869; 21383963; 21378048; 21372041; 21364493; 21362549; 21362492; 21348847; 21347226 |
| NT01ST0566 | 18 | 0.99 | 21397717; 21369825; 21059948; 20855510; 20720015; 20132828; 20070462; 19416927; 19302333; 16665774 |
| NT01ST0641 | 14 | 0.82 | 20676924; 12746293; 15955309; 12946365; 7763986; 7766613; 11080587; 10657901; 10597041; 9878395 |
| NT01ST0738 | 8 | 0.83 | 16524901; 12449384; 17991026; 17074894; 15904985; 9696761; 8214582; 10547699; 1556074; 3129571 |
| NT01ST1115 | 11 | 0.97 | 17900620; 2152912; 9332359; 8820638; 8509344 |
| NT01ST1275 | 11 | 0.71 | 20303825; 19501196; 19493006; 10829079; 17601790; 18363236; 17526845; 17379730; 17355601; 16804187 |
| NT01ST1283 | 11 | 0.71 | 20303825; 19501196; 19493006; 10829079; 17601790; 18363236; 17526845; 17379730; 17355601; 16804187 |
| NT01ST1302 | 11 | 0.71 | 20303825; 19501196; 19493006; 10829079; 17601790; 18363236; 17526845; 17379730; 17355601; 16804187 |
| NT01ST1531 | 13 | 0.98 | 10196363; 15103084; 16712869; 15772074; 15581587; 14501142; 12736709; 9829824 |
| NT01ST1649 | 15 | 0.96 | 21402758; 21396937; 21393212; 21390327; 21389340; 21386983; 21385318; 21385202; 21383239; 21379585 |
| NT01ST1798 | 8 | 0.72 | 20502966; 20405215; 19162251; 18421771; 18160405; 10803945; 16143847; 15752704; 15581577; 14967020 |
| NT01ST1824 | 18 | 0.98 | 21393220; 21384128; 21378194; 21371898; 21354532; 21310270; 21308399; 21170876; 21261641 |
| NT01ST1839 | 2 | 0.86 | 20713166; 20565114; 1678137; 11500486; 11322938; 10966576; 10333520; 10089457; 8591031; 4598072 |
| NT01ST2167 | 18 | 0.93 | 21335045; 21197505; 21190577; 21140912; 20949136; 20876384; 20851114; 20802201; 20649599; 20592205 |
| NT01ST2233 | 19 | 0.80 | 21397493; 21342541; 20876582; 20831592; 20628005; 20624909; 20439478; 20236121; 20048165 |
| NT01ST2285 | 18 | 0.84 | 21399638; 21398603; 21398465; 21397717; 21393220; 21383187; 21378194; 21377792; 21376116; 21375730 |
| NT01ST2467 | 4 | 0.76 | 19858188; 10468575; 17085552; 17015641; 16677309; 10468575; 10788321; 7768858; 9774535; 9356251 |
| NT01ST2576 | 6 | 0.89 | 20729809; 20457749; 20304994; 17560275; 16632468; 15707945; 15456277; 12531018; 9450760; 9858579 |
| NT01ST2798 | 11 | 0.88 | 21214923; 21041684; 19241380; 12079349; 9925584; 11244082; 16496690; 16453083; 12351820; 10500998 |
| NT01ST2863 | 8 | 0.70 | 21063111; 20647094; 11592066; 19591863; 19540598; 19500182; 19482183; 19478203; 19466450; 19292985 |
| NT01ST3031 | 19 | 0.98 | 20843347; 14617152; 10880432; 15009891; 11104814; 9428770; 12113939; 9858668; 9923682; 9155041 |
| NT01ST3251 | 11 | 0.71 | 20303825; 19501196; 19493006; 10829079; 17601790; 18363236; 17526845; 17379730; 17355601; 11598044 |
| NT01ST3565 | 15 | 0.87 | 12060687; 17320105; 15522091; 15228517; 8951818; 10995226; 2283426; 10540289; 8704970; 3991809 |
| NT01ST3611 | 18 | 0.72 | 20693325; 20435726; 19911130; 19656046; 19577910; 19423632; 16594065; 19013133; 1741458; 10619994 |
| NT01ST3625 | 19 | 0.81 | 21288267; 21273488; 21168419; 21127404; 21070836; 21068446; 21056541; 21047786; 21044320; 21040561 |
| NT01ST3959 | 18 | 0.96 | 21080032; 20826777; 20737137; 20585060; 2254250; 20418143; 20335169; 20086155; 19696110; 19609963 |
| NT01ST4150 | 18 | 0.96 | 19325113; 20133363; 20004175; 10827169; 15358235; 19024901; 18344567; 18313803; 17961285; 17920277 |
| NT01ST4519 | 19 | 0.93 | 21279381; 20972815; 20955181; 20702566; 20226261; 20089768; 19828160; 19825677; 19743414; 19589393 |
| NT01ST4994 | 8 | 0.80 | 20883732; 20693678; 18188553; 11919723; 17242513; 10361094; 7845354; 9193081 |
| NT01STA0049 | 15 | 0.71 | 21219468; 21067162; 20444101; 20233936; 19520724; 19482927; 18279344; 16677312; 15716451; 11226648 |
| NT01SW2077 | 15 | 0.81 | 20558511; 19016841; 17303566; 11244072; 16319496; 16209347; 8039908; 15583171; 15583165; 10322028 |
| NT01SW2296 | 15 | 0.91 | 19300486; 17660417; 10476035; 15225327; 11851334; 16561900; 9425243; 8459762; 2838724 |
| NT01SW2771 | 3 | 0.79 | 21097635; 20595000; 20487019; 20439474; 20398206; 20338182; 20018629; 17581122; 17355860; 19486161 |
| NT01TA0053 | 8 | 0.89 | 18079120; 11744691; 9603890; 11092943; 2294086 |
| NT01TA0066 | 9 | 0.92 | 21398488; 21384891; 21382465; 21354350; 21296885; 21263204; 21187145; 21155016; 21110176; 21106133 |
| NT01TA0129 | 8 | 0.73 | 21157015; 20799977; 20675477; 1700256; 11799204; 19732150; 19726675; 19452501; 19018016; 16244220 |
| NT01TA0184 | 18 | 0.97 | 20639324; 19533605; 9353044; 10417152; 10203839; 9457862; 16920706; 15918073; 11004167; 7012838 |
| NT01TA0192 | 15 | 0.71 | 21365689; 21354350; 21352825; 21220697; 21211289; 21178285; 21145325; 21127255; 21062968; 21038678 |
| NT01TA0193 | 18 | 0.73 | 20457585; 17473178; 20234002; 20146057; 20037756; 19925602; 11713258; 19366595; 19332362; 18977337 |
| NT01TA0350 | 12 | 0.96 | 20602853; 20510023; 20120222; 19658200; 17069638; 16520377; 16128611; 14706757; 12801932; 12139484 |
| NT01TA0444 | 14 | 0.86 | 21301083; 19723241; 16600626; 16480261; 11080587; 10891080; 9878395; 9776318; 8028028; 8104841 |
| NT01TA0481 | 9 | 0.82 | 21377632; 20877624; 20463028; 19866242; 19452559; 18657232; 15174055; 17326203; 16979414; 16952422 |
| NT01TA0918 | 8 | 0.93 | 10784035; 20148520; 20054534; 19202108; 18719904; 18620082; 17366474; 17097644; 16819729; 16232957 |
| NT01TA0959 | 19 | 0.85 | 20971868; 3304148; 18939472; 16505006; 18649738; 18048019; 10375550; 17032646; 1757461 |
| NT01TA1077 | 12 | 0.92 | 17092293; 15808850; 12796498; 11381270; 10064137; 8521970; 7677788 |
| NT01TA1118 | 2 | 0.84 | 20054111; 19946146; 2156133; 11891227; 11135669; 8514783 |
| NT01TA1274 | 11 | 0.89 | 20921317; 20498309; 12801945; 17172510; 10952608; 14618392; 10496929; 2463208; 1851643; 2843776 |
| NT01TA1284 | 18 | 0.90 | 16594065; 11602359; 16143847; 16135226; 8214582; 10206433; 9006953 |
| NT01TA1387 | 12 | 0.79 | 18846552; 15808850; 12796498; 8521970; 7677788 |
| NT01TA1395 | 6 | 0.83 | 15509785; 15253435; 15210332; 10753973; 11472367; 1346062; 9188783; 9092550; 8812460; 8553702 |
| NT01TA1397 | 18 | 0.85 | 21106037; 20881245; 20799957; 20631011; 20601085; 8202535; 20508090; 20435726; 20383414; 20358596 |
| NT01TA1453 | 18 | 0.97 | 21371898; 21310270; 21308399; 21170876; 21261641; 21256461; 21245145; 21238925; 21209283; 21209222 |
| NT01TC0525 | 19 | 0.93 | 21193820; 21126522; 20615998; 20079869; 19531602; 19412576; 19245333; 18679678; 18421587; 18379842 |
| NT01TD2648 | 18 | 0.77 | 21339607; 21044584; 20978126; 20827720; 20825352; 20600595; 20599688; 20529663; 20513760; 5527561 |
| NT01TE1112 | 6 | 0.82 | 21362257; 21176381; 21172308; 21143678; 21129289; 20880207; 20864644; 20533259; 20163247; 9183743 |
| NT01TE1224 | 2 | 0.91 | 11180061; 19283408; 12427032; 9575211; 10620354; 7665618; 7642565 |
| NT01TE1694 | 8 | 0.71 | 21382037; 21359551; 21325606; 21311030; 21308384; 21147832; 21049565; 21043108; 20960144; 20960142 |
| NT01TE1931 | 9 | 0.99 | 17385315; 16739479; 4623694; 15241633; 10937442; 10848999; 9744093; 9559566; 9231425; 8318516 |
| NT01TE2275 | 18 | 0.88 | 20519162; 19890837; 19780400; 18063795; 8314774; 12628916; 17619822; 16857941; 16595876; 16109844 |
| NT01TF0134 | 17 | 0.75 | 21388532; 21126315; 20026132; 10656815; 18439859; 17449473; 15375644; 14690591; 1122947; 12435362 |
| NT01TF0592 | 12 | 0.95 | 21404259; 21403388; 21402861; 21402761; 21402357; 21402152; 21402150; 21402050; 21401642; 21400099 |
| NT01TF0862 | 13 | 0.93 | 20110296; 17674047; 10660627; 14769948; 15276006; 15185964; 12458201; 11453072; 4349564; 9675891 |
| NT01TF1007 | 6 | 0.78 | 21124948; 19635595; 19563116; 18393679; 19719636; 17452361; 2659796; 17929923; 17881053; 7574479 |
| NT01TF1381 | 8 | 0.86 | 21051545; 21037180; 20652619; 5420057; 20511510; 2050109; 20337711; 20221733; 20056701; 20052967 |
| NT01TF1498 | 6 | 0.85 | 20048165; 14502267; 17048004; 15716268; 8928228; 8209257 |
| NT01TF1694 | 8 | 0.86 | 17508726; 16804169; 14742434; 10416260; 9722670; 9303884; 8486638; 344137 |
| NT01TF1786 | 19 | 0.76 | 20826214; 19793137; 18247030; 17924109; 17341817; 17277884; 17068754; 16990374; 15959723; 10968627 |
| NT01TF1791 | 18 | 0.95 | 21397717; 18088318; 8604299; 14568145; 9016539; 11414327; 11081796; 10347054; 7103660; 10361292 |
| NT01TF2026 | 9 | 0.90 | 19465092; 15987605; 15708363; 2207257; 6725256 |
| NT01TF2478 | 12 | 0.84 | 21037302; 20852022; 20826214; 20812763; 20799957; 20724278; 20696265; 20645085; 20616713; 20522495 |
| NT01TF2585 | 15 | 0.96 | 21124821; 20942908; 20870771; 20855615; 20722734; 20639324; 20553556; 20521955; 20348251; 20093290 |
| NT01TF2777 | 15 | 0.95 | 21383169; 21281728; 21190544; 21131488; 21035346; 20971899; 20935121; 20879840; 20716365; 20709900 |
| NT01TF2819 | 15 | 0.87 | 21350472; 21264306; 21114961; 21036245; 21030591; 20924200; 20883745; 20864477; 20702764; 20691271 |
| NT01TF2860 | 8 | 0.83 | 20123779; 20054534; 19834077; 19815845; 19566114; 10579571; 18620082; 8119488; 18336549; 18224307 |
| NT01TF2962 | 8 | 0.91 | 18694756; 16343420; 10357231; 12605255; 11606230; 11334785; 9737861; 8380721; 9180697; 8902625 |
| NT01TK0663 | 18 | 0.96 | 21209194; 20807201; 20705137; 20204475; 20152063; 20025906; 19843304; 19420709; 19847115; 19194888 |
| NT01TK1356 | 19 | 0.84 | 21367879; 21356201; 21330144; 21284263; 21194247; 21155392; 21088224; 21080475; 21056984; 20977886 |
| NT01TK1774 | 19 | 0.87 | 21367879; 21356201; 21354427; 21330144; 21284263; 21221632; 21194247; 21187397; 21168384; 21166996 |
| NT01TK1830 | 15 | 0.93 | 20012992; 18599838; 18005985; 17320105; 10967285; 15916601; 15702929; 9108148; 10467127; 11136469 |
| NT01TK2021 | 6 | 0.96 | 20877309; 19812063; 16428324; 15204703; 12819197; 11342551; 11081631; 10064605; 10856290; 10449415 |
| NT01TK2052 | 8 | 0.91 | 18694756; 16343420; 10357231; 12605255; 11606230; 11334785; 9737861; 8380721; 9180697; 8902625 |
| NT01TK2167 | 13 | 0.74 | 18552770; 17900615; 10722656; 16343540; 15210688 |
| NT01TL0235 | 19 | 0.74 | 20822875; 20739278; 20570296; 20532763; 20208168; 20159568; 19916085; 19778013; 19756576; 19662397 |
| NT01TL1738 | 8 | 0.76 | 21398604; 21398519; 21398220; 21397190; 21396932; 21393833; 21391225; 21388960; 21387410; 21386928 |
| NT01TP10720 | 18 | 0.70 | 21338571; 21198640; 21124736; 20578993; 20506231; 20478264; 20450889; 19805324; 18614020; 19760522 |
| NT01TP11362 | 19 | 0.85 | 21342516; 21193824; 21120468; 21030826; 20858202; 20812985; 20709893; 20680265; 20622122; 20599513 |
| NT01TP11714 | 19 | 0.88 | 21149454; 20686915; 20681989; 20563857; 20504027; 19807035; 19608744; 19517808; 19478450; 19344716 |
| NT01TT0870 | 18 | 0.91 | 20875429; 20851982; 1429629; 18998542; 18572928; 18470695; 18227241; 18025080; 11577723; 10612281 |
| NT01TT1901 | 2 | 0.94 | 17174261; 1141859; 9700068; 9252577; 7982968; 2542220; 791939; 14217462; 1097404 |
| NT01TT2130 | 18 | 0.75 | 20821001; 19244099; 19114588; 18441515; 17478601; 16675592; 15177457; 15050519; 11856762; 11500505 |
| NT01TT2228 | 4 | 0.87 | 19780824; 18826411; 17536675; 10087920; 3148491; 9106214 |
| NT01TT2567 | 18 | 0.92 | 11162101; 15812005; 10926494; 15808935; 9168605 |
| NT01TT2725 | 12 | 0.80 | 21188561; 21073144; 20609357; 20385758; 20071547; 19729064; 17901467; 8350998; 18524871 |
| NT01TT2758 | 18 | 0.91 | 16889884; 15763514; 15168617; 11829513; 11841238; 9787771; 9264544 |
| NT01TV1157 | 11 | 0.80 | 21073510; 20652620; 20602451; 20543074; 20521842; 20449818; 20363791; 12169598; 20007650; 19961612 |
| NT01TV1182 | 8 | 0.96 | 21370474; 21364298; 21325689; 21318135; 21293295; 21279683; 21278273; 21270427; 21261075; 21253866 |
| NT01TV1330 | 8 | 0.86 | 16844076; 16313413; 14605795; 12678433; 12110677; 11695833; 11695827; 11248188; 10486127; 2172211 |
| NT01TV1440 | 13 | 0.93 | 20399793; 16642040; 19520088; 15766524; 17936990; 17727881; 16330048; 16053518; 16168724; 16008351 |
| NT01UU0613 | 9 | 0.90 | 21248857; 20652826; 20622122; 20528952; 20086012; 20025994; 19898564; 19850005; 19656950; 11528392 |
| NT01VEA2739 | 8 | 0.90 | 21224997; 21209092; 20456655; 20033765; 20026072; 19996100; 19536464; 19013472; 18719905; 10713460 |
| NT01VEA3139 | 3 | 0.71 | 20796283; 19589067; 19423631; 15461798; 10993081; 17713814; 12446837; 17435117; 17140412; 16968224 |
| NT01VEA4375 | 18 | 0.73 | 21343370; 21218445; 21148088; 21139075; 20826668; 20428825; 20392955; 20357117; 19535589; 20053893 |
| NT01VEA4903 | 2 | 0.78 | 19622649; 11399071; 7473709; 7622491; 7814407; 8055941; 2115523; 2106516; 2506544; 3502256 |
| NT01VF0303 | 12 | 0.93 | 20592095; 3936687; 19189954; 19259827; 17565583; 11162110; 16632497; 15988562; 16661430; 15645143 |
| NT01VF0780 | 8 | 0.73 | 21178285; 20811135; 19966484; 19546514; 19424620; 19200041; 17291445; 16402358; 16221991; 15996793 |
| NT01VF1507 | 9 | 0.93 | 21388747; 21330637; 21327327; 21315740; 21269353; 21257793; 21171605; 21148782; 21147110; 21075079 |
| NT01VF1713 | 19 | 0.78 | 20594307; 19962135; 7854376; 19683318; 19646441; 18944395; 18943823; 18943613; 18943537; 18671744 |
| NT01VF1719 | 12 | 0.89 | 20844218; 20194926; 10740299; 17229438; 2129543; 16403873; 16229488; 15546659; 15263011; 15260479 |
| NT01VF2446 | 15 | 0.83 | 20000490; 16919681; 15313602; 15027196; 12744509; 12667072; 12391883; 8527132; 12068544; 11866516 |
| NT01VF2466 | 3 | 0.99 | 19857646; 16556217; 15170403; 9157238; 7565110 |
| NT01VF2578 | 8 | 0.77 | 21067517; 20581474; 20405931; 20303981; 20038140; 7860587; 15269332; 10788346; 14521953; 16275737 |
| NT01VFA0267 | 8 | 0.70 | 21235502; 21190518; 21138528; 21075841; 21068394; 21058656; 21051545; 21029046; 20978135; 20951673 |
| NT01VFA0595 | 15 | 0.89 | 21124821; 20927693; 20874755; 20811812; 20466769; 18076326; 19755120; 19465656; 18270203; 18178468 |
| NT01VFA0983 | 1 | 0.87 | 19585268; 19217306; 4859493; 18299981; 15954777; 15084713; 15035613; 12163143; 11596649; 9804062 |
| NT01VFA1061 | 8 | 0.81 | 21393237; 21220430; 21216999; 21068394; 21046342; 20975995; 20921383; 20868295; 20836540; 20826164 |
| NT01VP0867 | 4 | 0.76 | 20472726; 11325945; 17965153; 17630974; 10222208; 16751195; 15769466; 15558816; 11744713; 1846149 |
| NT01VP0892 | 13 | 0.83 | 16844682; 321008; 15292242; 12571243; 10383414 |
| NT01VP1158 | 18 | 0.96 | 21170879; 21278129; 21262798; 21236240; 21187068; 21151096; 21116569; 21107625; 21077901; 21072772 |
| NT01VP1169 | 4 | 0.86 | 20118256; 10447890; 14617184; 8808934; 11082305; 14171462; 1846779; 291033 |
| NT01VP1280 | 8 | 0.83 | 19382143; 17090920; 8132157; 11073918; 11459836; 11081795; 10537203; 9611813; 9367878; 9299451 |
| NT01VP1663 | 15 | 0.87 | 12060687; 17320105; 15522091; 15228517; 8951818; 10995226; 2283426; 10540289; 8704970; 3991809 |
| NT01VP2241 | 13 | 0.97 | 21320742; 21134380; 20974979; 20882017; 20823541; 20670890; 20696925; 19779558; 19423627; 20064631 |
| NT01VP2506 | 18 | 0.94 | 19325113; 20004175; 10827169; 15358235; 19024901; 18344567; 18313803; 17961285; 17920277; 17687501 |
| NT01VP2794 | 8 | 0.72 | 20601046; 20385101; 19383527; 17532339; 18675788; 18401010; 7007381; 18362922; 17927566; 17571210 |
| NT01VPA0075 | 19 | 0.97 | 19816782; 17944535; 18613689; 18351384; 17221229; 11773048; 12026180; 9891778; 11513090; 11414615 |
| NT01VPA0189 | 8 | 0.84 | 21364306; 21358759; 21354350; 21332878; 21315475; 21297942; 21296915; 21234102; 21194788; 21178163 |
| NT01VPA0605 | 8 | 0.74 | 17451240; 15264822; 18508968; 18365077; 18297793; 17915680; 17883338; 16849108; 16329976; 15568806 |
| NT01VPA1036 | 8 | 0.76 | 21342473; 21309870; 21280023; 21257770; 21252224; 21199252; 21184484; 21170562; 21160512; 21097634 |
| NT01VPA1235 | 18 | 0.98 | 21209370; 21184741; 20807201; 20655873; 20435726; 20178799; 20173761; 19800383; 10827169; 19754365 |
| NT01VV0017 | 18 | 0.87 | 21391682; 21389264; 21389124; 21383100; 21376091; 21372321; 21361312; 21355107; 21354299; 21346101 |
| NT01VV1442 | 8 | 0.79 | 21359175; 21327819; 20693447; 19962946; 19628817; 12140659; 19246762; 9210286; 17485086; 19158662 |
| NT01VV2640 | 4 | 0.81 | 21143895; 20691271; 19994673; 2112747; 18978020; 19210618; 19179756; 19074654; 18713007; 18462070 |
| NT01VVA0768 | 13 | 0.73 | 9844142; 19165584; 17943273; 17380901; 17103135; 17024311; 15748981; 15670165; 15301531; 15297933 |
| NT01WB0311 | 14 | 0.71 | 21392179; 21391975; 21391885; 21390549; 21389787; 21387213; 21386131; 21386086; 21386085; 21385847 |
| NT01WB0408 | 2 | 0.90 | 20936830; 20857974; 20734996; 20460376; 20054882; 19919618; 16905347; 18616280; 17452319; 17350958 |
| NT01WB0413 | 2 | 0.83 | 20942799; 20460376; 20097860; 19810706; 15018306; 11983079; 15952888; 1060112; 16603772; 16905347 |
| NT01WS0389 | 18 | 0.79 | 21369825; 21245145; 21209283; 21197505; 21191290; 21122813; 21115658; 21090806; 21059948; 21059651 |
| NT01WS2088 | 19 | 0.98 | 19400768; 18036201; 11090285; 10566865; 7925310; 11722566; 11398928; 11124264; 10873532; 4030693 |
| NT01XA0009 | 18 | 0.94 | 21399938; 20860484; 20643857; 3045756; 19130262; 18973471; 18629473; 11967085; 15993072; 1748657 |
| NT01XA0245 | 18 | 0.72 | 21392185; 20933601; 20849418; 20540995; 19636966; 19171150; 12192411; 11248124; 18622627; 18602403 |
| NT01XA0673 | 18 | 0.75 | 8957677; 20599705; 18957443; 19754895; 19729311; 19013219; 18452987; 16966422; 10676964 |
| NT01XA1872 | 9 | 0.82 | 21389045; 21388747; 21375498; 21362034; 21345233; 21331774; 21330637; 21327327; 21315740; 21307609 |
| NT01XA2179 | 8 | 0.72 | 20211485; 9269998; 19226621; 11100734; 17174341; 16855872; 16774472; 8752345; 16126232; 15970278 |
| NT01XA2249 | 18 | 0.99 | 20643857; 20047910; 19735464; 3045756; 18629473; 4196588; 17623027; 17385904; 11967085; 7642501 |
| NT01XA2309 | 2 | 0.81 | 20693992; 11159333; 10494632; 8969526; 1445232; 2110099 |
| NT01XA2436 | 19 | 0.95 | 20639136; 20522496; 20426480; 19884778; 19507068; 19466498; 19296676; 19205049; 19129644; 18999999 |
| NT01XA2990 | 15 | 0.72 | 20718459; 18724706; 17709748; 18313075; 17224607; 9493270; 15728912; 10331874; 15588829; 15447145 |
| NT01XA3151 | 18 | 0.84 | 21314091; 21263229; 20806404; 20739542; 20564199; 20463051; 20444574; 20212151; 2249631; 20178461 |
| NT01XA3240 | 6 | 0.82 | 21307601; 21261070; 21187418; 21133394; 21118349; 21070960; 21059649; 21051352; 21037296; 20975945 |
| NT01XA3437 | 19 | 0.73 | 18854951; 18596046; 18304669; 14736729; 14736729; 11841812 |
| NT01XA3445 | 19 | 0.83 | 18854951; 17356870; 12757950; 11841812; 8935665; 9144435; 8935665; 1658561; 8439670 |
| NT01XA3529 | 12 | 0.73 | 2129543; 8655500; 15993836; 15838638; 10508090; 1809829; 10972813; 10699328; 9623911; 271968 |
| NT01XA3937 | 12 | 0.92 | 21400725; 21400693; 21400691; 21400688; 21400619; 21400610; 21400609; 21400565; 21400546; 21400519 |
| NT01XA4063 | 5 | 0.82 | 21087930; 2138605; 15580782; 10224133; 9647837; 10200269; 17450323; 14585934; 7972072; 7493934 |
| NT01XA4735 | 18 | 0.89 | 21369825; 21204689; 21122131; 21090806; 21071707; 21059948; 21059651; 20955543; 20954009; 20938339 |
| NT01XA5718 | 4 | 0.75 | 21347234; 21040514; 20536745; 17555432; 10447890; 16430701; 8707053; 14645278; 15769466; 15661011 |
| NT01XC1213 | 15 | 0.88 | 18313075; 17224607; 12917445; 12475235; 12469338; 10518785; 10092458; 9680958; 9356247; 8733235 |
| NT01XC1840 | 9 | 0.97 | 18791145; 1545801; 18523866; 17934871; 17614770; 17484888; 17444966; 17093216; 16513294; 14981222 |
| NT01XC2530 | 15 | 0.81 | 21178479; 21109564; 20826333; 20636376; 20624216; 12730390; 20545848; 20522500; 20231439; 18485075 |
| NT01XC3218 | 1 | 0.95 | 17288446; 16472303; 16950391; 7896726; 16245858; 12400704; 12005058; 11601612; 11359561; 11337276 |
| NT01XC4821 | 4 | 0.82 | 21237251; 20952574; 20922376; 20704181; 20562102; 12692562; 20432484; 20132828; 20086163; 19151095 |
| NT01XF0009 | 18 | 0.99 | 21399938; 21354401; 21338516; 21329359; 21278293; 21276097; 21190440; 21189348; 21183643; 21179522 |
| NT01XF0032 | 3 | 0.76 | 20738375; 20457291; 20338182; 19817860; 18718522; 10993081; 17322192; 17121595; 15612917; 10564475 |
| NT01XF0069 | 2 | 0.73 | 21371789; 21283685; 21214021; 21163251; 21091475; 21084312; 21081698; 21047785; 21038477; 20972559 |
| NT01XF0346 | 8 | 0.84 | 21397024; 21397009; 21385310; 21367862; 21331762; 21320492; 21319986; 21291916; 21170889; 21269378 |
| NT01XF0384 | 18 | 0.73 | 21073858; 20735204; 20522553; 20521619; 20420430; 19479006; 19708087; 19594131; 19479947; 19463859 |
| NT01XF0388 | 18 | 0.99 | 21397717; 21250599; 21194366; 21148731; 21053951; 21041493; 20855510; 20799927; 20625147; 20599727 |
| NT01XF0389 | 11 | 0.99 | 21262357; 16181782; 18227257; 17367389; 16731525; 10722135; 10986230; 9316915; 9393713; 2548993 |
| NT01XF0390 | 11 | 0.99 | 20547379; 16181782; 17078817; 16731525; 10722135; 10986230; 10556026; 9393713; 2548993; 10760133 |
| NT01XF0408 | 8 | 0.92 | 21370474; 21159175; 21086129; 20535465; 20472739; 20400555; 20054113; 19586914; 19572314; 18845584 |
| NT01XF0439 | 3 | 0.99 | 21398554; 21091863; 20944223; 20797400; 20570733; 20487271; 20086145; 19891507; 8529885; 19507071 |
| NT01XF0481 | 18 | 0.89 | 21396420; 21386061; 21385507; 21377531; 21375706; 21373771; 21371427; 21371038; 21368142; 21367787 |
| NT01XF0485 | 18 | 0.71 | 20532806; 20164445; 19919002; 18606475; 19689950; 16594065; 11006093; 11602359; 17121816; 17090931 |
| NT01XF0512 | 12 | 0.82 | 21372759; 21286385; 20920355; 20735720; 20457869; 20400202; 19694617; 19143597; 18850059; 10617467 |
| NT01XF0563 | 3 | 0.94 | 21371235; 21311024; 21304951; 21256201; 21245931; 21245534; 21234771; 21227987; 21115127; 21097635 |
| NT01XF0670 | 12 | 0.79 | 21371455; 21366963; 21365649; 21348480; 21334801; 21330608; 21329689; 21314960; 21311678; 21288455 |
| NT01XF0685 | 4 | 0.71 | 21402362; 21402352; 21401642; 21396201; 21395450; 21395449; 21393367; 21392132; 21391724; 21390252 |
| NT01XF0695 | 18 | 0.88 | 10829079; 20482592; 20335169; 20299406; 20102440; 19731368; 10504726; 19153809; 3054465; 9453631 |
| NT01XF0820 | 11 | 0.75 | 19326202; 9294425; 16348513; 14019094; 3548034; 3904174; 6702102 |
| NT01XF0850 | 4 | 0.87 | 21404117; 21402769; 21402762; 21402761; 21402760; 21402759; 21402758; 21402357; 21402352; 21401642 |
| NT01XF1009 | 18 | 0.98 | 20519162; 12867445; 19890837; 19780400; 18063795; 8314774; 12628916; 17651435; 17619822; 15077103 |
| NT01XF1079 | 11 | 0.98 | 20467813; 20363791; 19493003; 19183283; 18497485; 16769696; 15215404; 15347755; 14553929; 12726763 |
| NT01XF1146 | 9 | 0.74 | 15221226; 12680762; 1459244; 11099866; 10991936; 10945972; 10427752; 7607251; 6345794 |
| NT01XF1190 | 18 | 0.84 | 21187464; 21168419; 21149736; 20554613; 21115899; 21104926; 21077936; 21062971; 21046154; 20935265 |
| NT01XF1351 | 13 | 0.93 | 21102444; 20947765; 20890290; 20838377; 20807316; 20670889; 20659448; 20505071; 11879651; 20226789 |
| NT01XF1514 | 17 | 0.92 | 21205867; 20952386; 9446751; 20595001; 20553390; 20545305; 20678145; 20453093; 20354151; 20227844 |
| NT01XF1653 | 18 | 0.99 | 21392495; 21372393; 21369825; 21347851; 21347827; 21345797; 21341745; 21336797; 21332314; 21325265 |
| NT01XF1684 | 8 | 0.78 | 18317695; 15917234; 8335628; 11807567; 10984484; 16666683; 9761836; 7569952; 8399175; 8360156 |
| NT01XF1736 | 2 | 0.96 | 21368171; 21255426; 21231969; 21206014; 21142117; 21081498; 21075928; 21072368; 21071388; 21038112 |
| NT01XF1769 | 4 | 0.86 | 21397711; 19852988; 18945840; 19372158; 19368775; 17629557; 17033047; 15033238; 15567279; 12871230 |
| NT01XF1812 | 8 | 0.86 | 21371432; 21315380; 21248490; 21185308; 21136597; 21122807; 21115336; 21104949; 21093100; 20841500 |
| NT01XF1928 | 8 | 0.82 | 21358763; 21325689; 21248489; 21216240; 21185256; 21127473; 21126600; 21115169; 21068209; 21064166 |
| NT01XF1978 | 18 | 0.96 | 21178960; 21030403; 20978133; 20932953; 20869350; 20713918; 20713739; 20696265; 20691655; 20680571 |
| NT01XF2049 | 4 | 0.85 | 21335456; 21252222; 20730544; 20463026; 20139188; 19897918; 12060747; 19763738; 19332821; 19082958 |
| NT01XF2125 | 4 | 0.74 | 21279427; 21081474; 21030914; 20237573; 19886864; 19753378; 19167407; 19129167; 19106090; 18722362 |
| NT01XF2150 | 12 | 0.82 | 21205036; 20798503; 20132020; 20061385; 19758361; 19478443; 18199413; 19251047; 18037263; 16945147 |
| NT01XF2177 | 18 | 0.92 | 20929736; 20970482; 21030639; 20921224; 20838342; 20686069; 20634426; 20513761; 20483324; 20185828 |
| NT01XF2194 | 3 | 0.95 | 21398554; 21255118; 21142157; 21115127; 21098028; 21036658; 20971868; 20738375; 20708667; 20656905 |
| NT01XF2207 | 8 | 0.72 | 21301099; 21294712; 21255311; 21193677; 20823547; 20797453; 20721539; 20606284; 20527958; 20118260 |
| NT01XF2270 | 18 | 0.96 | 21153767; 20736165; 20592244; 20337714; 20203292; 20163190; 20150244; 20042604; 19922410; 19857597 |
| NT01XF2343 | 18 | 0.90 | 21264094; 21205015; 21173183; 21149619; 20921306; 20883496; 19342493; 17493798; 19574656; 19558960 |
| NT01XF2408 | 18 | 0.96 | 21179061; 21059656; 20929736; 20978133; 20932953; 21030639; 20869350; 20844539; 20826797; 20720542 |
| NT01XF2543 | 12 | 0.99 | 20553980; 19000094; 18591879; 18547641; 18508466; 18286570; 17981718; 9974389; 17161345; 16782851 |
| NT01XF2553 | 18 | 0.79 | 20881245; 19429920; 19102629; 18628769; 12622816; 11039922; 17713961; 8096630; 8849412; 11045621 |
| NT01XF2586 | 6 | 0.75 | 21163962; 21347417; 21315607; 21287394; 21274469; 21263241; 21249131; 21238786; 21229971 |
| NT01XF2638 | 12 | 0.87 | 21378058; 20955556; 20862304; 20847048; 20727857; 20648511; 20552259; 20504766; 20466747; 20448033 |
| NT01XF2656 | 19 | 0.73 | 18854951; 18596046; 18304669; 14736729; 14736729; 11841812 |
| NT01XF2685 | 4 | 0.73 | 17425668; 16390454; 16203150; 15383161; 14678166; 12209001; 10582867; 9837987; 10188253 |
| NT01XF2691 | 19 | 0.90 | 20351104; 20042300; 19762441; 10992472; 8636107; 17680768; 16950533; 8743704; 10411730; 15491367 |
| NT01XF2748 | 18 | 0.86 | 20190787; 21191377; 21187464; 21149736; 20554613; 21115899; 21104926; 21103977; 21094854; 21077936 |
| NT01XF2760 | 18 | 0.95 | 21347851; 21122131; 21118089; 21110944; 20888343; 20888319; 20828733; 20705604; 20643857; 20639324 |
| NT01XF2846 | 15 | 0.96 | 21320584; 21315771; 21295603; 21250657; 21239493; 21216996; 21216906; 21193607; 21178479; 21149619 |
| NT01XF3005 | 18 | 0.79 | 21393247; 21393174; 21384090; 21359593; 21354366; 21342117; 21328631; 21321274; 21308736; 21287651 |
| NT01XF3045 | 6 | 0.92 | 20587501; 3074013; 20418439; 1741244; 20140025; 20134230; 19304752; 19758331; 19232054; 1812789 |
| NT01XFA0066 | 6 | 0.85 | 21362621; 21354613; 21333363; 21276096; 21263027; 21262234; 21196264; 21195035; 21188165; 21156963 |
| NT01XFB0001 | 6 | 0.94 | 21403392; 21402598; 21402428; 21402113; 21402084; 21401918; 21401783; 21401563; 21399625; 21399624 |
| NT01XO0974 | 6 | 0.71 | 21394295; 21382659; 21380616; 21377995; 21375672; 21370284; 21364307; 21362556; 21361792; 21357694 |
| NT01XO1274 | 19 | 0.92 | 21357429; 21339299; 21307593; 21304822; 21282527; 21269444; 21146533; 21054875; 20973991; 20944232 |
| NT01XO1349 | 15 | 0.94 | 12686116; 11158353; 1318499; 9844742; 9495022; 9467911; 9168623; 8392137 |
| NT01XO1571 | 18 | 0.80 | 21358545; 21357734; 21331626; 21314928; 21312224; 21311889; 21309733; 21307845; 21302669; 21273422 |
| NT01XO1919 | 6 | 0.93 | 21392582; 21364582; 21352556; 21321020; 21306759; 21304883; 21303649; 21296759; 21283726 |
| NT01XO3990 | 3 | 0.86 | 20953948; 20331639; 20233936; 10993081; 20070368; 9393554; 10993081; 17322192; 10564475; 8899718 |
| NT01XO4239 | 8 | 0.85 | 21199936; 21168410; 21093782; 21082862; 21081474; 21068394; 21046342; 21038895; 21030914; 20976072 |
| NT01XO4741 | 18 | 0.85 | 21149716; 21059661; 20708613; 20688187; 18004396; 20613726; 20592205; 20483361; 11825133; 20393197 |
| NT01XO5075 | 19 | 0.88 | 21273488; 21067711; 21051539; 20967294; 20847102; 20823521; 20795726; 20735086; 20709852; 20708927 |
| NT01XV0202 | 12 | 0.92 | 21320498; 21048103; 17257854; 16055222; 6699152; 15618353; 15581796; 15204311; 15110334; 15070833 |
| NT01XV2520 | 19 | 0.70 | 21400715; 21400698; 21400681; 21400661; 21400660; 21400651; 21400585; 21400575; 21400481; 21400228 |
| NT01XV2557 | 18 | 0.85 | 20923416; 20829798; 11976294; 17322201; 1498688; 15686563; 15327991; 15183890; 14872322; 14629018 |
| NT01XV4182 | 19 | 0.75 | 21169496; 21041303; 20977193; 20920201; 20554962; 15695454; 20213317; 20180804; 18045787; 19996150 |
| NT01XV4962 | 12 | 0.76 | 21332624; 21292493; 21229878; 21087457; 21069157; 21062896; 21037108; 20936527; 20870437; 20860530 |
| NT01YE0902 | 11 | 0.70 | 21228534; 21062506; 20813133; 20056615; 20016040; 17428497; 18753784; 6283090; 10465774; 11917032 |
| NT01YE1236 | 18 | 0.76 | 19822337; 8257103; 18399798; 17202864; 16118285; 10544289; 9644260; 9139716; 1329093; 7809621 |
| NT01YE1427 | 8 | 0.76 | 21398595; 21397011; 21387033; 21376774; 21374701; 21374662; 21358191; 21357619; 21354629; 21354350 |
| NT01YE1896 | 6 | 0.90 | 21364324; 21310712; 21291520; 21224630; 21224584; 21205839; 21187428; 21110832; 21092102 |
| NT01YE2054 | 11 | 0.90 | 15066036; 9220002; 9159521; 7645241; 7632676; 7799432; 4578100; 2973176; 2965251; 2961121 |
| NT01YE2454 | 11 | 0.82 | 21364304; 20662890; 20467813; 15953930; 19921332; 11524133; 18279340; 10688204; 9097419; 16199584 |
| NT01YE2698 | 9 | 0.74 | 21388423; 21378393; 21375722; 21375706; 21375498; 21337322; 21318289; 21310443; 21307094; 21296915 |
| NT01YE3544 | 19 | 0.82 | 20622122; 20570296; 20349118; 20169343; 20153658; 19706595; 19683032; 19605459; 12045105; 19504047 |
| NT01YE4009 | 12 | 0.80 | 20032457; 11751634; 17504491; 17855452; 10569626; 16127432; 16109378; 15249048; 15195946; 15157086 |
| NT01YE4023 | 9 | 0.96 | 20725044; 20616867; 19423627; 1355089; 15514159; 18557770; 17266990; 10716717; 16707089; 16276872 |
| NT01YE4299 | 19 | 0.81 | 21258004; 12949092; 9707537; 7568131; 11297407; 11260463; 1522059 |
| NT01YP0672 | 15 | 0.72 | 21034467; 20148929; 16882028; 18630346; 10348866; 15912452; 12694615; 12657056; 1745240 |
| NT01YP2162 | 18 | 0.76 | 21265778; 21091602; 20472799; 1326356; 15035043; 8971712; 16763151; 17986187; 16928682; 16417500 |
| NT01YP2676 | 12 | 0.74 | 21398480; 21221937; 21184302; 21141460; 21125383; 21090243; 20959988; 20926695; 20925060; 20880591 |
| NT01YP2776 | 2 | 0.90 | 20936830; 20857974; 20460376; 20054882; 9927721; 16905347; 17350958; 16431905; 16364320; 11158531 |
| NT01YP2920 | 4 | 0.72 | 16944097; 16521149; 7661448; 11700361; 9244278; 8288518; 2999794; 2691873; 2716520 |
| NT01YPA0035 | 6 | 0.85 | 20798162; 19303899; 17698626; 2694936; 12624212; 12207703 |
| NT01ZM0004 | 5 | 0.81 | 19770499; 16387658; 1318389; 12426581; 12007424; 11724564; 11389593; 8274013; 2545683; 2824486 |
| NT01ZM0597 | 18 | 0.83 | 17493798; 19840771; 9287004; 8125105; 7836312; 18272295; 16211368; 16030212; 16722228; 15319545 |
| NT01ZM0810 | 8 | 0.87 | 21059110; 20847256; 20645352; 19943898; 19717732; 19496430; 19060411; 19038229; 18395192; 17891922 |
| NT01ZM0811 | 8 | 0.84 | 19923715; 18216065; 16919403; 11566130; 10565547 |
| NT01ZM0882 | 2 | 0.81 | 21389612; 21092138; 20832326; 20817265; 20722615; 20711197; 20552672; 20539033; 20450493; 20393702 |
| NT01ZM1122 | 19 | 0.93 | 15256257; 11554469; 7189755; 9973330; 10395827; 12713541; 12514238; 12492837; 9445479; 12393017 |
| NT01ZM1336 | 8 | 0.84 | 19923715; 18216065; 16919403; 11566130; 10565547 |
| NT01ZM1371 | 18 | 0.88 | 20870774; 20467255; 20033069; 9787636; 14960717; 19095018; 10896219; 16030141; 17551219; 17350859 |
| NT01ZM1372 | 18 | 0.88 | 20870774; 20467255; 20033069; 9787636; 14960717; 19095018; 10896219; 16030141; 17551219; 17350859 |
| NT01ZM1381 | 18 | 0.91 | 20870773; 19400808; 17974510; 17803963; 16079355; 10559156; 15258141; 10319814; 14766911; 12867744 |
| NT01ZM1413 | 8 | 0.71 | 21341803; 21186120; 21126006; 20975902; 20873618; 20715795; 20660227; 20564611; 20354759; 19476485 |
| NT01ZM1463 | 19 | 0.86 | 21378199; 21369973; 21369910; 21357626; 21357429; 21324659; 21311881; 21307589; 21297160 |
| NT01ZM1665 | 8 | 0.76 | 21398391; 21388532; 21378355; 21367571; 21364306; 21359858; 21355870; 21339386; 21339137; 21338521 |
| NT01ZM1830 | 12 | 0.98 | 21277783; 21216092; 21212976; 21173228; 21139689; 21130737; 21118346; 21109192; 21095563; 21093407 |
| NT01ZM1894 | 8 | 0.90 | 21305608; 21271855; 21271853; 21183445; 21072380; 21068384; 21064266; 21029720; 20960122; 20952576 |
| NT01ZM1934 | 18 | 0.96 | 20737137; 20639324; 15689108; 19430763; 16049196; 9353044; 19132541; 18982279; 18096847; 3308853 |
| NT01ZM2087 | 8 | 0.82 | 21358763; 21325689; 21248489; 21216240; 21185256; 21127473; 21126600; 21115169; 21068209; 21064166 |
| NT02AB1708 | 8 | 0.88 | 20160912; 19996100; 16531404; 14644451; 12603319; 11955070; 11935326; 11781147; 11708858; 11673873 |
| NT02AB2229 | 13 | 0.99 | 20952379; 20923763; 20629144; 19749248; 19656186; 18719118; 19187230; 19179530; 19179361; 18775898 |
| NT02AB2320 | 18 | 0.92 | 20846937; 20802073; 20696823; 20647000; 10829079; 20352420; 20335169; 20299406; 20164862; 20161024 |
| NT02AB4008 | 18 | 0.76 | 21359673; 21357485; 21302294; 21266406; 21265761; 21263029; 21212072; 21187432; 21187400; 21183954 |
| NT02AC0662 | 19 | 0.96 | 21317267; 21221939; 20571959; 20382819; 20223653; 20109094; 3696175; 19883124; 12962235; 19645671 |
| NT02AC0663 | 19 | 0.76 | 21377658; 21290602; 21174947; 21038354; 20935120; 20873227; 20834142; 20811983; 20795726; 20730755 |
| NT02AC1568 | 19 | 0.91 | 21054166; 20868765; 20666458; 20469649; 20223653; 20201406; 19596709; 19442228; 19246207; 18467126 |
| NT02AE0506 | 9 | 0.99 | 4324203; 20379720; 19889625; 16484204; 4374474; 17651922; 15258140; 15028711; 14663079; 11904150 |
| NT02AE1731 | 19 | 0.96 | 21030539; 20824214; 20595206; 20707404; 20201406; 19883124; 19825675; 19414810; 19298858; 9736698 |
| NT02AT0270 | 1 | 0.86 | 18481057; 11216661; 16669068; 10966576; 11213 |
| NT02AT0953 | 8 | 0.76 | 20889970; 20718446; 20652619; 20606288; 20547883; 20362339; 19804777; 15859246; 1639790; 17964805 |
| NT02AT1399 | 15 | 0.77 | 10592234; 15170488; 11180822; 8704152; 2107277 |
| NT02AT2435 | 8 | 0.73 | 21091201; 20514241; 19011745; 12003933; 18422649; 17350704; 15680231; 14965227; 12974644; 12388585 |
| NT02AT2516 | 4 | 0.93 | 14659047; 10400593; 1400163; 9240446; 1528267; 14237063; 7637778; 3323803; 7689140; 17246400 |
| NT02AV1074 | 4 | 0.82 | 20546773; 20513630; 3136011; 14657018; 17066651; 12752683; 15246989; 12830385; 12590300; 12223550 |
| NT02AV1232 | 8 | 0.79 | 17128979; 15342248; 12527201; 12517343; 10840044; 10329704; 10231530; 9890977; 9593854; 8993342 |
| NT02AV2125 | 18 | 0.77 | 21339577; 21321143; 21315184; 21067451; 21063116; 21029429; 20959616; 20949000; 20936704; 20851747 |
| NT02AV2373 | 8 | 0.96 | 21315823; 21303200; 21152918; 21061628; 21029720; 20958226; 20802042; 20636270; 20613764; 20601013 |
| NT02AV3061 | 8 | 0.75 | 21390523; 21366264; 21317262; 21278308; 21272045; 21184752; 21146502; 21040753; 21035426; 20980458 |
| NT02AV3303 | 1 | 0.84 | 21128869; 19549189; 17896178; 17595805; 17573062; 16820168; 15865426; 15840047; 11371182; 11169018 |
| NT02AV3564 | 8 | 0.89 | 19882159; 17562223; 16228362; 11553768; 11443110; 2491675 |
| NT02AV5020 | 8 | 0.76 | 21366264; 21271977; 21236328; 21084058; 20541869; 20516587; 20399532; 20224939; 19926682; 19825612 |
| NT02BA0024 | 6 | 0.90 | 21193388; 21080372; 21059412; 21042723; 20949080; 20938339; 21058426; 20930037; 20920744; 20874390 |
| NT02BB0162 | 18 | 0.95 | 20308306; 3054465; 18402979; 19636864; 11737642; 9025287; 8951808; 6226306; 8437515; 1406279 |
| NT02BB0316 | 18 | 0.91 | 19423632; 19013133; 18248418; 10619994; 17221235; 16477356; 16418175; 8932698; 15961260; 15948956 |
| NT02BB0617 | 12 | 0.81 | 21343416; 21321669; 21311096; 21292744; 21231916; 21204021; 21106534; 20889486; 20856808; 20847048 |
| NT02BB0630 | 8 | 0.73 | 21178285; 20811135; 19966484; 19546514; 19424620; 19200041; 17291445; 16402358; 16221991; 15996793 |
| NT02BB0709 | 18 | 0.97 | 21369825; 21304833; 21059948; 20804453; 20720015; 20659291; 20212112; 20208152; 8407802; 9529892 |
| NT02BB1019 | 8 | 0.73 | 21178285; 20811135; 19966484; 19546514; 19424620; 19200041; 17291445; 16402358; 16221991; 15996793 |
| NT02BB1165 | 12 | 0.90 | 21303942; 21261463; 21232591; 21153812; 20874089; 20871837; 20833217; 20713620; 20679205; 20675003 |
| NT02BB1242 | 18 | 0.75 | 21241388; 21207455; 21143936; 21114408; 21034599; 20937124; 20886638; 20881245; 20877283; 20706981 |
| NT02BB1297 | 18 | 0.87 | 19268700; 18627004; 17541839; 16364310; 16212599; 15927675; 15192086; 14655754; 9933992; 12444043 |
| NT02BB1394 | 14 | 0.95 | 18067538; 18054556; 17962997; 4604641; 15280220; 15130468; 12903193; 12324998; 12244448; 11494055 |
| NT02BB1509 | 12 | 0.85 | 21333629; 21327044; 21320469; 21262232; 21219594; 21193663; 21193577; 21129186; 21125388; 21120455 |
| NT02BB1561 | 1 | 0.76 | 21297164; 20704760; 20592025; 19501038; 19286931; 17175447; 16518696; 8027034; 18032385; 17452319 |
| NT02BB1591 | 5 | 0.98 | 19531743; 19770499; 19730970; 1525856; 16049108; 15882565; 15065880; 12716056; 11921096; 10806350 |
| NT02BB1808 | 8 | 0.83 | 20967536; 20628047; 2033048; 19021509; 9374473; 11520617; 10215894; 11069671; 1840615; 10672190 |
| NT02BB2080 | 19 | 0.71 | 15691006; 11017768; 9858642; 9850421; 9111134; 7852863; 8076653; 8201220; 8509426; 1835319 |
| NT02BB2378 | 18 | 0.78 | 21384763; 21383851; 21376776; 21375531; 21347350; 21333710; 21325403; 21299244; 21285319; 21283768 |
| NT02BB2408 | 8 | 0.73 | 21178285; 20811135; 19966484; 19546514; 19424620; 19200041; 17291445; 16402358; 16221991; 15996793 |
| NT02BB2462 | 9 | 0.92 | 21377632; 21354350; 21347589; 21347544; 21344271; 21341335; 21333574; 21320492; 21318295; 21317262 |
| NT02BB2483 | 18 | 0.96 | 20933199; 20445069; 20493455; 20217497; 20053100; 19955484; 19747469; 19694789; 19655111; 19654142 |
| NT02BB2526 | 2 | 0.89 | 20961145; 12873140; 19438611; 9920791; 10436161; 14505379; 14705938; 10819988; 16897469; 16533066 |
| NT02BB3095 | 12 | 0.83 | 21130733; 20877571; 20858456; 20824169; 20670659; 20544843; 20486760; 19158376; 11356520; 19908197 |
| NT02BC0193 | 8 | 0.82 | 18289689; 8226720; 1543699; 2687259; 3214167; 6166606 |
| NT02BC0201 | 8 | 0.88 | 21329681; 21265764; 19932956; 19900530; 19472401; 19450714; 5432063; 19082766; 18850096; 17640279 |
| NT02BC0694 | 8 | 0.92 | 17433574; 11021946; 8496150; 8385013; 1446828; 1427101; 1510560; 1597190; 2007567; 2252897 |
| NT02BC0878 | 8 | 0.89 | 21329681; 21265764; 19932956; 19900530; 5432063; 19082766; 18850096; 17640279; 17485854; 17433574 |
| NT02BC0973 | 6 | 0.97 | 21090726; 20638361; 20552646; 20482310; 19767856; 19491084; 19056144; 15574326; 18394656; 18307725 |
| NT02BC1050 | 18 | 0.89 | 21358271; 21339577; 21332893; 21315799; 21310270; 21308399; 21288822; 21170880; 21170874; 21271856 |
| NT02BC1051 | 8 | 0.92 | 21362632; 21238579; 21187465; 20837989; 20681784; 20545351; 17381106; 20424179; 20373852; 20361926 |
| NT02BC1172 | 4 | 0.94 | 21255377; 19801546; 18334534; 17581123; 15277670; 12950930; 11722737; 9535081; 8412687 |
| NT02BC1198 | 12 | 0.76 | 21030958; 20600941; 20038533; 19597489; 19432418; 18474220; 17991743; 11381114; 16887800; 16455668 |
| NT02BC1254 | 4 | 0.83 | 21398543; 21375591; 21344251; 21092197; 20863881; 20621133; 20601499; 20551082; 20545323; 20451384 |
| NT02BC1257 | 18 | 0.78 | 21385869; 21385839; 21381019; 21378197; 21378181; 21369424; 21357901; 21354347; 21347707; 21343931 |
| NT02BC1281 | 8 | 0.89 | 21376015; 21038895; 20609914; 19549597; 18991392; 18981569; 18945673; 18930846; 2542902; 18472393 |
| NT02BC1285 | 8 | 0.98 | 21358763; 21119085; 20795369; 20395268; 20353438; 20331426; 20299454; 18820069; 19915948; 19887598 |
| NT02BC1315 | 8 | 0.81 | 21296058; 21216231; 21166653; 20955166; 20933050; 20837989; 20809073; 20806931; 19965430; 20632934 |
| NT02BC1730 | 19 | 0.81 | 21193820; 20868765; 20601267; 9572842; 20403753; 20212162; 20194690; 20172492; 20045693; 19962718 |
| NT02BC2232 | 8 | 0.90 | 21303655; 1662759; 2088175; 17480102; 15606778; 15358267; 12208142; 9600061; 7606170; 1610409 |
| NT02BC2285 | 19 | 0.70 | 19538395; 19502509; 18461320; 3335502; 16096274; 15280033; 15159628; 14684172; 14518977; 10517585 |
| NT02BC2471 | 4 | 0.73 | 21365081; 21278754; 21225062; 21159796; 20833577; 12007800; 20639314; 20615929; 20566763; 20478990 |
| NT02BC2549 | 4 | 0.75 | 20713681; 17923393; 17726620; 17532190; 17520806; 17350183; 16911866; 15059283; 15834559; 15574480 |
| NT02BC2827 | 18 | 0.91 | 21126504; 20498258; 20018840; 11207551; 2939801; 9401119; 17272682; 15228545; 17074317; 16804679 |
| NT02BC2873 | 18 | 0.89 | 21358271; 21339577; 21332893; 21315799; 21310270; 21308399; 21288822; 21170880; 21170874; 21271856 |
| NT02BC3046 | 4 | 0.83 | 21398556; 21389112; 21378197; 21378181; 21352236; 21299880; 21232149; 21221837; 21208192; 21199192 |
| NT02BC3104 | 6 | 0.97 | 21386365; 21292986; 21285356; 21280222; 21274582; 21250850; 21112256; 21078962; 21070408; 21037296 |
| NT02BC3283 | 8 | 0.92 | 21362632; 21238579; 21187465; 20837989; 20681784; 20545351; 17381106; 20424179; 20373852; 20361926 |
| NT02BC3323 | 9 | 0.97 | 19166906; 18614287; 16650384; 15469937; 12366803; 7948012; 8027063; 8477967; 1455165; 7115321 |
| NT02BC3369 | 2 | 0.79 | 21110975; 20955518; 12472699; 18818314; 18846290; 18846282; 11967097; 17472958; 16915519; 16021340 |
| NT02BC3481 | 4 | 0.83 | 21398556; 21389112; 21378197; 21378181; 21352236; 21299880; 21232149; 21221837; 21208192; 21199192 |
| NT02BC3689 | 8 | 0.84 | 20924198; 18467858; 18188555; 18060402; 17587673; 10867230; 12149117; 8617755; 11934292; 11931561 |
| NT02BC3726 | 19 | 0.98 | 21388880; 21357429; 21347309; 21339299; 21327741; 21307593; 21304822; 21300542; 21282527; 21269444 |
| NT02BC5242 | 1 | 0.89 | 20947662; 20830571; 20696910; 20658158; 20541568; 20494107; 20379751; 20164178; 20086163; 15208350 |
| NT02BC5309 | 9 | 0.75 | 21377632; 21325261; 21211036; 21170680; 21130127; 21106133; 21071855; 20937244; 20923481; 20801191 |
| NT02BC5358 | 19 | 0.98 | 21388880; 21357429; 21347309; 21339299; 21327741; 21307593; 21304822; 21300542; 21282527; 21269444 |
| NT02BF0220 | 12 | 0.88 | 9870699; 8087850; 6384729; 8462840; 2679887; 8387148; 2088168; 2041472; 2249673 |
| NT02BF0440 | 8 | 0.83 | 9973347; 12639950; 11835514; 10203754; 9383150; 7894055; 8397187; 2134185 |
| NT02BF0505 | 5 | 0.89 | 20207756; 10194322; 10368287; 14749331; 11015224; 7565414; 10403130; 9524276; 3558364; 7565414 |
| NT02BM0346 | 12 | 0.72 | 21220484; 21123754; 21109561; 20876432; 7968073; 20580496; 20467255; 20385758; 20217167; 20212185 |
| NT02BM0524 | 12 | 0.72 | 20974857; 20965153; 20952389; 20873853; 20736789; 20714129; 20668211; 20644913; 20633031; 20602988 |
| NT02BM0925 | 4 | 0.80 | 21267607; 20637256; 20356456; 20173000; 20078128; 19298085; 19229492; 18355320; 17069977; 16040186 |
| NT02BM0931 | 5 | 0.75 | 21212358; 20977213; 20718463; 20709836; 20558750; 20411554; 20400946; 20221547; 20221527; 20219465 |
| NT02BM1132 | 8 | 0.83 | 20630744; 19777228; 12655455; 12057198; 2082825; 11717506; 9666313; 9322760; 9168611; 7952195 |
| NT02BM1982 | 15 | 0.87 | 21346155; 21296585; 21175428; 11581164; 5937781; 11181018; 17937768; 9162900; 10329189; 16155226 |
| NT02BMA0411 | 19 | 0.79 | 21327035; 21241420; 21111546; 21097635; 20973806; 20971910; 20971903; 16597618; 20594157; 20497501 |
| NT02BP1559 | 4 | 0.73 | 19361928; 8755888; 1404383; 1828466; 1905667; 3076089; 3007433 |
| NT02BP1560 | 4 | 0.85 | 17015641; 8755888; 10468575; 8107139; 8308888; 1404383; 1551848; 1370543; 1828466; 1905667 |
| NT02BS1877 | 9 | 0.78 | 19815542; 18047786; 15539300; 8706906; 8925405; 7636910; 7565640; 8408590; 3027079; 13271455 |
| NT02BS1896 | 8 | 0.87 | 21068394; 21051545; 20966083; 20889970; 20737579; 20718446; 20690702; 20652669; 20652619; 20606288 |
| NT02BS2179 | 9 | 0.91 | 21388804; 21378051; 21351250; 21049984; 20658340; 20630605; 20503262; 20416968; 20410100; 20301160 |
| NT02BSA0633 | 8 | 0.93 | 21349979; 21345474; 21323388; 21317262; 21306561; 21293046; 21275058; 21265508; 21255865; 21241658 |
| NT02BSA0961 | 8 | 0.75 | 12200544; 9538249; 4091279; 6547672; 6292658; 7358641 |
| NT02BSA1664 | 8 | 0.94 | 21376015; 21352799; 21094149; 21091510; 21068394; 21051545; 21029046; 20978135; 20966083; 20952574 |
| NT02BSA3306 | 6 | 0.86 | 20929586; 10210213; 20036200; 19800300; 19393175; 6307121; 19074850; 19060414; 18971960; 8663612 |
| NT02BSB0203 | 9 | 0.71 | 21365262; 21347282; 21316679; 21303701; 21283740; 21252068; 21155670; 21081177; 20937151; 20864671 |
| NT02BSB0296 | 8 | 0.76 | 20620150; 18680749; 8743581; 15809331; 11306094; 10978349; 9059644; 8898089; 2157439; 2326309 |
| NT02BT1362 | 1 | 0.94 | 20056607; 19449898; 16943623; 16292576; 5432063; 12624751; 12589472; 405455; 11163791; 11080625 |
| NT02BT1521 | 19 | 0.73 | 21097635; 20497501; 20497501; 20400548; 18490448; 8830709; 10419957; 10369758; 15943817; 10496936 |
| NT02BT2153 | 18 | 0.73 | 21310951; 21177345; 21175430; 21124864; 21068307; 21064033; 21060781; 21037580; 20980587; 20937842 |
| NT02BT2228 | 8 | 0.86 | 20676631; 19560494; 18619846; 10715008; 19399347; 9135111; 187575; 6388497; 9254694; 12921536 |
| NT02BT2607 | 19 | 0.91 | 21393206; 20974151; 20870765; 20849416; 20580675; 20206184; 20013255; 19807868; 17646652; 845124 |
| NT02BT3388 | 8 | 0.75 | 21229467; 21194928; 21188075; 21160204; 21137052; 21117708; 21103092; 20943168; 20876192; 20678796 |
| NT02BT4034 | 4 | 0.92 | 21092197; 20432948; 8655545; 17634510; 7885225; 17381738; 17040240; 16907802; 16553729; 16049010 |
| NT02BT4315 | 12 | 0.93 | 21395946; 21384360; 21280569; 21240566; 21076486; 21067677; 20976223; 20873796; 20870311; 20833629 |
| NT02BT5114 | 15 | 0.75 | 21375718; 12800502; 15720552; 17293407; 18292804; 17177879; 16579459; 12850135; 10094627; 9889978 |
| NT02BTA0009 | 4 | 0.89 | 21371137; 21216948; 20597981; 19502400; 19709333; 19493277; 15837378; 17651447; 10328075; 18326183 |
| NT02BTA0071 | 6 | 0.76 | 21394101; 21393072; 21392397; 21391904; 21390132; 21389894; 21389547; 21389348; 21389131; 21388532 |
| NT02BV0300 | 6 | 0.90 | 21355971; 20875449; 20833787; 7968535; 20640526; 20581194; 20026194; 19783470; 19766657; 19725510 |
| NT02BV0524 | 12 | 0.94 | 20926695; 20668094; 20348249; 20171112; 19953351; 15948963; 19250902; 19195484; 19133079; 19084538 |
| NT02BV2428 | 9 | 0.91 | 20846931; 20656688; 20605911; 20332208; 2771945; 17957779; 17709268; 10571059; 12369917; 12007602 |
| NT02BV2682 | 17 | 0.93 | 19621586; 18851833; 16455663; 15953538; 15674325 |
| NT02BVB1252 | 8 | 0.75 | 20334431; 19737939; 19055322; 19038292; 7248267; 9509248; 12646371; 12176061; 11855936; 11827546 |
| NT02BVE0234 | 6 | 0.80 | 20864034; 20627952; 20473270; 20432434; 20351180; 20179009; 16951256; 17064419; 19343651; 14966270 |
| NT02CB0148 | 12 | 0.98 | 21397065; 21368222; 21347827; 21320870; 21319259; 21315771; 21314817; 21299121; 21268879; 21262231 |
| NT02CB0936 | 15 | 0.74 | 21115395; 20878781; 20869472; 20134056; 16840531; 8076591; 9555905; 10829079; 12427949; 10760155 |
| NT02CB1226.1 | 4 | 0.73 | 20418388; 8456299; 16213082; 18001143; 17609136; 17286419; 15306010; 14636056; 14621982; 6379600 |
| NT02CB1326 | 19 | 0.98 | 21357429; 21282527; 21169496; 21040305; 20937888; 20920201; 20852022; 20851967; 20843347; 20829286 |
| NT02CB1760 | 8 | 0.78 | 20679207; 20597606; 19889946; 17108241; 19379783; 18455501; 17609257; 16469539; 16453288; 16377227 |
| NT02CG2450 | 6 | 0.84 | 21306995; 21248859; 21241792; 21193392; 21078962; 20800503; 20734974; 20541511; 17126598; 8595885 |
| NT02CG3162 | 8 | 0.92 | 19818736; 18497090; 18361512; 18023619; 19157549; 16274879; 16128611; 15388933; 15229897; 15090746 |
| NT02CH0069 | 19 | 0.86 | 20213317; 20079869; 12429703; 10962023; 10029552; 1398087 |
| NT02CH0373 | 19 | 0.88 | 21311881; 20576315; 20570296; 20372022; 19883701; 19645671; 19625389; 19382702; 12110300; 18224282 |
| NT02CH0532 | 8 | 0.95 | 21120593; 20802042; 20633693; 15987803; 19616643; 8486629; 19259137; 19128036; 19097788; 14988572 |
| NT02CH0581 | 8 | 0.96 | 21288652; 21123035; 21091667; 21057948; 20833784; 20649840; 20601046; 20488895; 20487290; 20385101 |
| NT02CH0797 | 9 | 0.98 | 20379951; 15968460; 15155740; 16707089; 16460018; 14960587; 12048195; 11883946; 11346647; 10821865 |
| NT02CH0843 | 9 | 0.98 | 21094633; 20931591; 20924576; 20846931; 20353815; 20332534; 20237302; 20223213; 20110695; 20067470 |
| NT02CH1052 | 8 | 0.72 | 20655986; 8550433; 12369917; 7813460; 11053834; 16348446; 1362236; 7582169; 1409539 |
| NT02CH1224 | 19 | 0.96 | 21054875; 20638050; 20153658; 20039037; 19962718; 19777228; 19682300; 18987878; 18705851; 18624642 |
| NT02CH1729 | 1 | 0.77 | 21393246; 21383205; 21372135; 21219974; 21210868; 21185009; 21138988; 21081499; 21079812; 20971073 |
| NT02CH1766 | 8 | 0.94 | 20633693; 20628895; 15987803; 20485385; 19959836; 9680610; 19616643; 8486629; 19348883; 19128036 |
| NT02CH2079 | 6 | 0.90 | 21132514; 21030321; 20876134; 20805320; 20693977; 20519779; 20516198; 20491765; 20447876; 20224553 |
| NT02CH2332 | 14 | 0.89 | 21205085; 16778368; 19775280; 11895911; 16491070; 18839780; 18773877; 18255096; 15324697; 17602053 |
| NT02CH2343 | 12 | 0.82 | 21248119; 21187486; 21187062; 21173516; 21140473; 21116918; 21098883; 21098492; 21081040; 21077523 |
| NT02CH2418 | 8 | 0.94 | 21386030; 21370474; 21338644; 21329996; 21325606; 21324682; 21323163; 21315475; 21307384; 21294661 |
| NT02CJ0035 | 12 | 0.94 | 2248769; 10693756; 17051148; 16321976; 12225666; 12214059; 11793244; 11672525; 8915840 |
| NT02CP0327 | 9 | 0.99 | 21390227; 21069965; 20693992; 18824113; 20418430; 20406823; 20370610; 20226757; 20201588; 20176020 |
| NT02CP0585 | 18 | 0.79 | 21378188; 21366542; 21349151; 21317537; 21315686; 21310741; 21291544; 21284393; 21281708; 21279359 |
| NT02CP0847 | 18 | 0.85 | 21092325; 20816073; 20653412; 315944; 18276250; 20126988; 20101631; 20010300; 19850449; 19751420 |
| NT02CT0330 | 14 | 0.86 | 21398490; 21396949; 21396482; 21395333; 21342445; 21324702; 21320556; 21317409; 21311411; 21299636 |
| NT02CT0459 | 12 | 0.87 | 17436086; 16565075; 9716100; 15288868; 11978727; 9851654; 9792826; 1281457 |
| NT02CT0797 | 18 | 0.97 | 21371898; 21369825; 21354532; 21310270; 21308399; 21170876; 21261641; 21256461; 21247492 |
| NT02CT1122 | 12 | 0.92 | 21400725; 21400693; 21400691; 21400688; 21400619; 21400610; 21400609; 21400565; 21400546; 21400519 |
| NT02CT1223 | 18 | 0.78 | 21385869; 21385839; 21381019; 21378197; 21378181; 21369424; 21357901; 21354347; 21347707; 21343931 |
| NT02CT1607 | 18 | 0.88 | 21296744; 21057623; 20878713; 20458187; 20420920; 19719510; 20304922; 20189481; 20171266 |
| NT02CT1819 | 5 | 0.74 | 21318560; 20722631; 20036737; 19806451; 15687489; 15657036; 15634687; 14973553; 10521484; 9099729 |
| NT02CT1888 | 12 | 0.77 | 20448065; 19847098; 14534577; 18381780; 17352256; 10886369; 10476719; 15727257; 12745921; 12704195 |
| NT02CT2314 | 8 | 0.74 | 21037291; 20832411; 20724492; 20665426; 20656883; 20228169; 20139187; 20044564; 19782502; 19765784 |
| NT02CT2551 | 8 | 0.93 | 12165429; 5432063; 1935949; 1900194; 6816896 |
| NT02DA0735 | 8 | 0.84 | 21324916; 21323331; 21296913; 21275244; 21272918; 21266166; 21257036; 21247988; 21240476; 21198961 |
| NT02DA1389 | 18 | 0.82 | 20704181; 20147287; 16875436; 19761223; 19004000; 18793179; 18252722; 17600077; 17168567; 17148438 |
| NT02DA1896 | 18 | 0.94 | 21392495; 21343459; 21338466; 21303921; 21299248; 21257298; 21251927; 21237168; 21216355; 21194365 |
| NT02DA2401 | 18 | 0.78 | 19684063; 19558963; 17054778; 17120758; 16913910; 2001402; 16207915; 15517343; 12882966; 1625581 |
| NT02DA2425 | 6 | 0.84 | 11069656; 1946387; 17207624; 16793378; 16202661; 11259647; 10535734; 12509298; 12509239; 12459439 |
| NT02DA2679 | 9 | 0.92 | 18982870; 17717280; 17606858; 17491019; 16179798; 2172217; 15265042; 9804184; 9485310; 7639527 |
| NT02DA3060 | 12 | 0.92 | 17400722; 16148011; 15893634; 12832290; 1705030; 10452886; 9092528; 7969132; 7766636; 2823875 |
| NT02DA3074 | 8 | 0.73 | 21178285; 20811135; 19966484; 19546514; 19424620; 19200041; 17291445; 16402358; 16221991; 15996793 |
| NT02DA3368 | 8 | 0.72 | 21165740; 17259621; 11349275; 17005560; 10366508; 8782398; 7548156; 8180195; 8241148; 1702437 |
| NT02DA3538 | 15 | 0.95 | 19632156; 11831459; 8051064; 17185537; 12651949; 16158234; 3109911; 8231807 |
[truncated: 237,099 more chars]
